# Incidence of Lower Respiratory Tract Infections and Atopic Conditions in Boys and Young Male Adults: Royal College of General Practitioners Research and Surveillance Centre Annual Report 2015-2016

**DOI:** 10.2196/publichealth.9307

**Published:** 2018-04-30

**Authors:** Simon de Lusignan, Ana Correa, Richard Pebody, Ivelina Yonova, Gillian Smith, Rachel Byford, Sameera Rankiri Pathirannehelage, Christopher McGee, Alex J Elliot, Mariya Hriskova, Filipa IM Ferreira, Imran Rafi, Simon Jones

**Affiliations:** ^1^ Department of Clinical and Experimental Medicine University of Surrey Guildford United Kingdom; ^2^ Respiratory Diseases Department National Infection Service Public Health England London United Kingdom; ^3^ Real-time Syndromic Surveillance Team National Infection Service Public Health England Birmingham United Kingdom; ^4^ Research and Surveillance Centre Clinical Innovation and Research Centre Royal College of General Practitioners London United Kingdom; ^5^ Clinical Innovation and Research Centre Royal College of General Practitioners London United Kingdom

**Keywords:** population surveillance, medical record system, Computerized, general practice, pharyngitis, common cold, rhinitis, bronchitis, conjunctivitis, asthma, urinary tract infections, gastroenteritis

## Abstract

**Background:**

The Royal College of General Practitioners Research and Surveillance Centre comprises more than 150 general practices, with a combined population of more than 1.5 million, contributing to UK and European public health surveillance and research.

**Objective:**

The aim of this paper was to report gender differences in the presentation of infectious and respiratory conditions in children and young adults.

**Methods:**

Disease incidence data were used to test the hypothesis that boys up to puberty present more with lower respiratory tract infection (LRTI) and asthma. Incidence rates were reported for infectious conditions in children and young adults by gender. We controlled for ethnicity, deprivation, and consultation rates. We report odds ratios (OR) with 95% CI, *P* values, and probability of presenting.

**Results:**

Boys presented more with LRTI, largely due to acute bronchitis. The OR of males consulting was greater across the youngest 3 age bands (OR 1.59, 95% CI 1.35-1.87; OR 1.13, 95% CI 1.05-1.21; OR 1.20, 95% CI 1.09-1.32). Allergic rhinitis and asthma had a higher OR of presenting in boys aged 5 to 14 years (OR 1.52, 95% CI 1.37-1.68; OR 1.31, 95% CI 1.17-1.48). Upper respiratory tract infection (URTI) and urinary tract infection (UTI) had lower odds of presenting in boys, especially those older than 15 years. The probability of presenting showed different patterns for LRTI, URTI, and atopic conditions.

**Conclusions:**

Boys younger than 15 years have greater odds of presenting with LRTI and atopic conditions, whereas girls may present more with URTI and UTI. These differences may provide insights into disease mechanisms and for health service planning.

## Introduction

The Royal College of General Practitioners (RCGP) Research and Surveillance Centre (RSC) publishes an annual report highlighting trends in respiratory, infectious, and some noninfectious conditions in England [1,2]. RCGP RSC data are extracted weekly from the computerized medical records (CMR) of >150 representative general practices in England, a sentinel network covering a population of over 1.5 million patients, that is, 3% of the population. RCGP RSC is one of the oldest sentinel networks; it was established as a Weekly Returns Service (WRS) in 1964, and it has just completed its 50^th^ season of influenza surveillance [3,4]. The WRS reports continue to this day; they are available on the Web or individuals can sign up to receive them [2]. The network continues to conduct influenza surveillance and review vaccine effectiveness as part of a longstanding collaboration with Public Health England [5], which extends into broader public health interests, including promoting winter wellness [6]. RCGP RSC also contributes to European surveillance and studies of vaccine effectiveness [7,8].

RCGP RSC data are representative of the national population [1] in terms of the following:

1. *Age and gender of the population*: This is largely representative, although we have a slightly higher proportion of people aged 25 to 44 years and a lower proportion of people aged >75 years.
2. *Ethnicity*: The majority of patients in the RCGP RSC network were of white ethnicity (84.35%, 543,452/644,273). We have a slightly higher (though within 1%) prevalence of Asian and black ethnicities.
3. *Deprivation*: We have a slight over-representation of the less deprived using the Index of Multiple Deprivation (IMD). The mean deprivation score for the RCGP RSC population was 19.8 (SD 0.01), compared with the English population score of 21.8 (SD 0.0005).
4. *Other factors*: We have a geographical distribution of practices across England and actively recruit where we have gaps. A comparison of our practices with national pay-for-performance data, the quality and outcomes framework (QOF), and prescribing data suggests our data are representative. Our practices generally perform better at QOF (proportion of QOF targets achieved is 97.4% [529.58/559; SD 0.02%] compared with 94.7% [544.45/559; SD 0.0006%] for non-RCGP RSC practices). We also actively provide feedback to practices about recording of infections, especially encouraging accurate recording of episode type, to differentiate first or new (incident) cases from ongoing (prevalent) cases. We also extract data from all the different brands of the CMR system.

In addition to its weekly report, RCGP RSC produces an Annual Report (Multimedia Appendix 1) [9]. This report contains incidence data for 37 conditions or groups of conditions that are included in the WRS report. This report generally has a theme alongside these weekly incidence reports. In 2014/15, the theme was contrasting conditions that have different seasonal patterns [10]. In 2015/16, we explore gender differences in the presentation of respiratory conditions and infections in children and young adults. The network director hypothesized that in over 3 decades as a general practitioner (GP), he had seen more boys with lower respiratory conditions and asthma than girls, and this is the theme of the 2015/16 report.

This study explores differences, by age and gender, of respiratory conditions and infections presenting to general practice.

## Methods

We extracted data from 155 participating practices who are members of RCGP RSC; a cohort of 1,589,702 patients registered during the period of 4 May 2015 to 8 May 2016. The data extracted were anonymized and encrypted; we only extracted coded data, not free text. Data were coded with Read version 2 or Clinical Terms version 3 [11].

RCGP RSC practices should have good data quality, particularly for influenza-like illness (ILI), acute infections, and respiratory conditions. RCGP RSC practices are encouraged to record the most likely diagnosis as a problem title and also assign an “episode type” to differentiate first or new presentations from ongoing care. Most of the data quality feedback to RSC member practices focuses on data quality for ILI, acute respiratory infection, and respiratory conditions. Since its inception, RCGP RSC has encouraged participating GPs to record valid and reliable diagnostic data; these approaches have been in place for some decades [12]. More recently, we have introduced financially incentivized training and practice-specific comparative feedback, which are modeled on the principles of audit-based education [13].

### Data Processing and Analysis

We used the age bands that have been used long term by RCGP RSC to facilitate historic comparisons. These age bands were as follows: <1 year, 1-4 years, 5-14 years, and 15-24 years.

**Figure 1:**
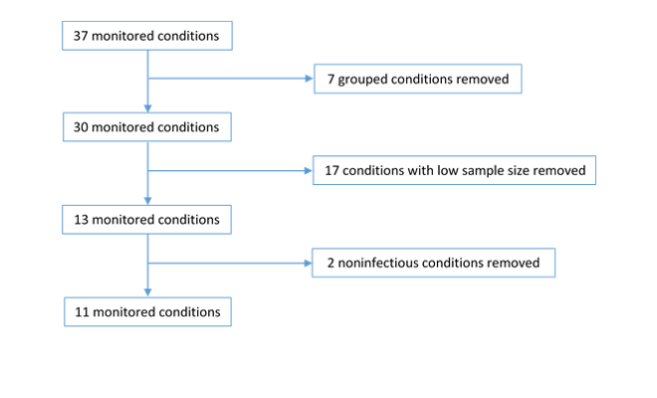
Selection of 13 conditions from the 37 items included in the Weekly Returns Service report and Annual Report of the Royal College of General Practitioners Research and Surveillance Centre for detailed investigation.

The WRS and Annual reports have traditionally reported 37 conditions or groups of conditions. On this basis, we excluded 5 because they were aggregates of several illnesses (but we kept lower respiratory tract infections [LRTIs] and upper respiratory tract infections [URTIs] as conditions of interest), 2 conditions that were not respiratory or infections, and 17 conditions that did not have sufficient sample size for our model (Figure 1). This left 13 conditions of the 37 items recorded, for which we explored gender differences in depth.

We grouped together LRTI and ILI, as influenza generally involves both upper and lower respiratory tracts, and diseases involving the lower respiratory tract are more clinically significant. We also grouped together URTI and conjunctivitis, as the latter is generally secondary to nasal obstruction. Finally, we grouped together asthma and allergic rhinitis (AR) as atopic respiratory conditions.

This left 13 conditions to consider in detail. Those were as follows: acute bronchitis and ILI (which we also grouped together with LRTI); acute tonsillitis, common cold, sinusitis, acute otitis media (AOM), and conjunctivitis (which we grouped together with URTI); asthma and AR, which we grouped together as atopic conditions; and finally, urinary tract infection (UTI) and intestinal infectious disease. Although these 13 conditions have been given a single-disease label, we group together a number of codes that fit with that disease concept using an ontological approach [14]. For example, hay fever would be included within AR codes. A full list is provided in Multimedia Appendix 2.

To assess gender differences by age group, adjusting for other demographic variables, we developed 56 multivariate logistic regression models. The data were subset into 4 age bands used in RCGP RSC data (<1 year, 1-4 years, 5-14 years, and 15-24 years). Moreover, 14 conditions, including atopy (ie, AR or asthma), each measured per each age band, gave us 56 models. The outcome variable was whether the patient presented with each of the conditions above, and the explanatory variables included the following:

1. Gender (female was the reference group)
2. Ethnicity (white ethnicity was reference, and we divided into Asian (A), black (B), mixed (M), other (O), and unclassified (U) ethnicities) [15]
3. Deprivation was reported by the IMD quintile (quintile 1, the most deprived quintile, was used as reference)
4. Propensity to consult using consultation rate by decile band (band 1, the decile with the lowest consultation rate, was the reference, and band 6 was used in the models where there were no events in the consultation band 1; please refer to the information below for a description of this variable).

We also calculated the crude probability of males presenting with each condition in their respective age band and the adjusted probability based on the logistic model using the variables above.

### Propensity to Consult

We assigned each patient a consultation rate. We then divided these rates into deciles, as a measure of propensity to consult. We calculated consultation rates in the following manner:

1. We summed up the number of visits (consultations or presentations) by patients to a GP as the number of consultations that they had in a year to find the numerator.
2. The denominator was 365, that is, the number of days in a year.
3. We divided the number of consultations per person in a year by 365 and multiplied the result by 100 to find the percentage of days in a year in which they consulted a GP.

The rate was defined as the number of different days that person attended their practice over 365 days. We conducted a sensitivity analysis, building 56 new regression models with an alternative measure for propensity to consult (annual rate of consultations without an “action” taken, in the form of a prescription or referral). This sensitivity analysis was conducted to determine whether removing consultations for patients who visited their GP without any action had an effect on the model.

The outcome variable and the other explanatory variables remained the same for these new regression models. The only changes were the different approaches to the “propensity to consult” explanatory variable. It must be noted that age, gender, and ethnicity may be confounding variables with consultation rates.

We report the results of the gender variable in this paper. For gender, we derived the odds ratio (OR), 95% CI, and probability (p) from the multivariate logistic regression [16]. An OR of >1 implies greater odds of a male/boy presenting with the condition; an OR of <1 suggests lower odds of a male/boy presenting, adjusting for other variables in the model. We created an aggregated table showing those conditions, with significant results denoted in italics (Table 1). Given the large number of models, we applied a Bonferroni-Šidák correction to the significance level of <.05, resulting in a new level of <.001 [17,18].

Additionally, the forest plots for each of the 13 conditions and the 4 age bands were reported separately (Multimedia Appendix 3). For each condition and each analysis, we quote OR, 95% CI, *P* value, and probability (p). Probability is calculated from the coefficients of logistic regression. We also include forest plots of the gender results of the 13 conditions closely studied, by age band. All the statistical analyses were conducted using functions in the statistical software R. The results of the sensitivity analysis were presented in a similar aggregated format (Table 2). We exclude a number of results in certain age subsets because of low numbers of events per predictor variables; we used a minimum of 20 events per variable as the threshold for exclusion [19].

The analysis presented in the Annual Report (Multimedia Appendix 1) includes the following:

1. Map of the national distribution of RCGP RSC practices
2. Summary tables showing the conditions we monitor, which is a new addition to the 2015/16 report:
  - Median age (using horizontal box-whisker plots)
  - Gender distribution of our monitored conditions
  - Ethnicity distribution comparing white and nonwhite
  - Median IMD (again using a horizontal box-whisker plot)
3. Week-by-week incidence of the conditions monitored by RCGP RSC—these data are published annually by RCGP RSC. Population denominators were based on the population registered in the participating practices for the study period. The weeks are numbered according to the International Organization for Standardization, ranging from week 1 to 52 in a single year [20].
4. An age-sex profile comparing the distribution of the disease with the national population, the distribution of the condition’s deprivation score compared with the rest of RCGP RSC, and similarly for ethnicity. The level of deprivation was determined using IMD [21], scored from 0.5 (least deprived) to 92.6 (most deprived), based on each patient’s Lower Super Output Area, which is determined from their postcode [22]. Ethnic groups, based on the 2011 English census categories, were assigned using an algorithm that incorporated proxy markers for ethnicity, such as language spoken [14]. This is also a new addition to the 2015/16 report.

### Ethical and Data Sharing Considerations

Disease surveillance is part of standard health service activity, and therefore, no specific ethical approval was needed. The Health and Social Care Act 2012 includes the Secretary of State’s duty to act to protect public health. RCGP RSC data extraction and analytics hub provides Public Health England with disease surveillance and vaccine effectiveness data. Data are pseudonymized as close to a source as possible, and no personal identifiers are held on the RCGP RSC secure network at the University of Surrey. We did not process the data of patients who had an “opt out” code.

Data are shared in a way which safeguards the confidentiality and anonymity of participants. Requests for access to data should be addressed to the data custodian of this study, Professor Simon de Lusignan.

## Results

### Lower Respiratory Tract Infections

Acute bronchitis was the single LRTI with the highest odds of presentation in boys up to the age of 15 years (Table 1). The greater ORs of boys attending were as follows: 1.59 (95% CI 1.35-1.87, *P*<.001) for those aged under 1 year, 1.13 (95% CI 1.05-1.21, *P*<.001) for those aged 1-4 years, and 1.20 (95% CI 1.09-1.32, *P*<.001) for those aged 5-14 years. After the age of 15 years, there are no significant gender differences (OR 1.11, 95% CI 1.00-1.23; *P*=.05).

**Figure 2:**
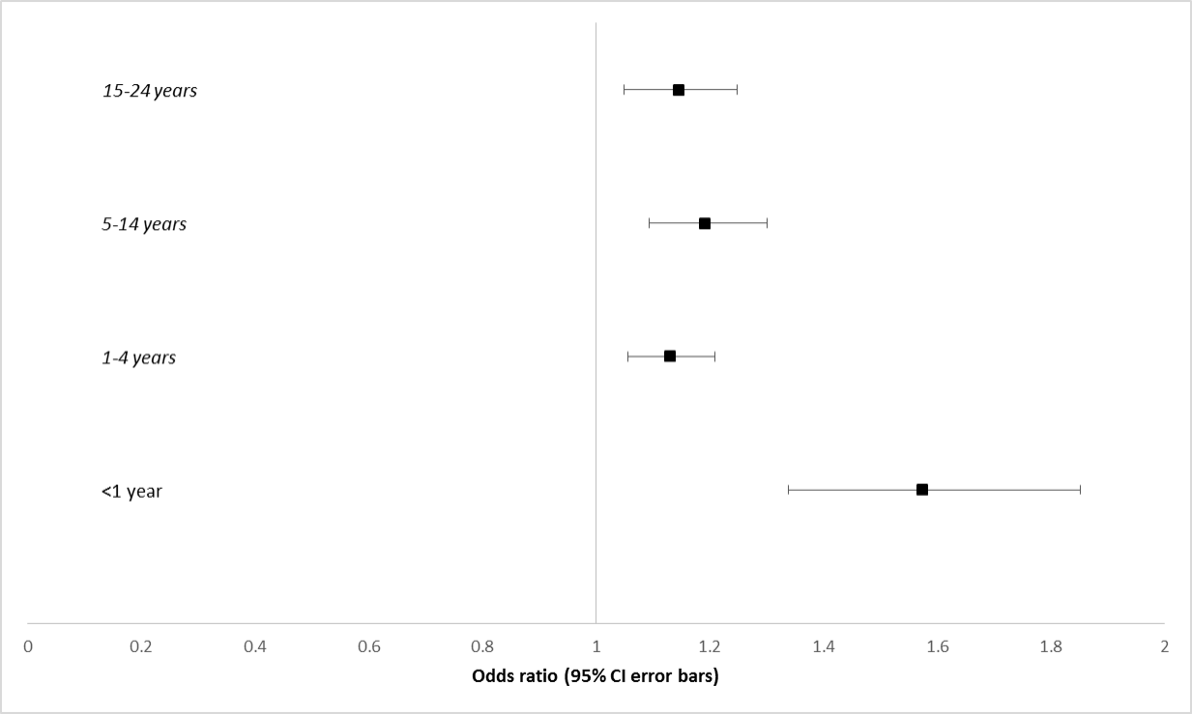
Forest plot of odds ratios of males younger than 25 years presenting with lower respiratory tract infection and influenza-like illness. Ages with statistically significant results are in italics. LRTI: lower respiratory tract infection.

Boys younger than 15 years had greater odds of presenting with LRTI and ILI, although much of this effect was due to acute bronchitis (Figure 2). For boys younger than 1 year, OR was 1.57 (95% CI 1.34-1.87; *P*<.001), for those aged 1-4 years, OR was 1.12 (95% CI, 1.05-1.20; *P*<.001), and for boys aged 5 to 14 years, OR was 1.20 (95% CI 1.10-1.31, *P*<.001). After the age of 15 years, no significant gender differences appear (OR 1.13, 95% CI 1.03-1.23; *P*=.01).

### Upper Respiratory Tract Infections

**Figure 3:**
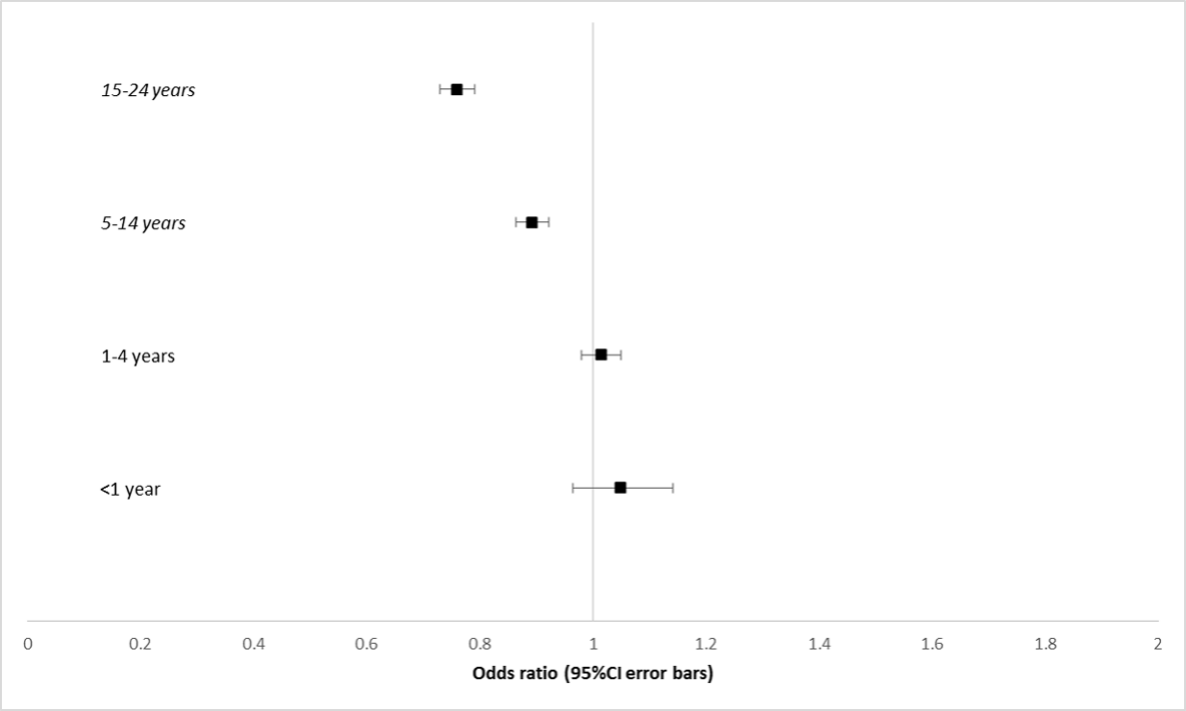
Forest plot of odds ratios of males younger than 25 years presenting with upper respiratory tract infection and conjunctivitis. Ages with statistically significant results are in italics. URTI: upper respiratory tract infection.

Boys older than 5 years had lower odds of presenting with URTIs, with OR decreasing with age (Figure 3). For boys aged 5-14 years, OR was 0.89 (95% CI 0.86-0.92, *P*<.001), whereas for males aged 15-24 years, OR was 0.76 (95% CI 0.73-0.80, *P*<.001). Under the age of 5 years, there were no significant gender differences (<1 year: OR 1.04, 95% CI 0.96-1.13; *P*=.37 and 1-4 years: OR 0.99, 95% CI 0.95-1.02; *P*=.44).

Out of this grouping, tonsillitis had the lowest odds of presentation for boys aged 5-14 years (OR 0.81, 95% CI 0.76-0.85; *P*<.001) compared with common cold (OR 0.93, 95% CI 0.89-0.97; *P*<.001); the results for sinusitis showed no difference (Table 2). For males aged 15-24 years, sinusitis had the lowest odds of presentation (OR 0.60, 95% CI 0.52-0.70; *P*<.001), followed by tonsillitis (OR 0.73, 95% CI 0.69-0.77; *P*<.001) and common cold (OR 0.80, 95% CI 0.75-0.86; *P*<.001). The results for children aged 0-4 years were not significant, or, as in the case of sinusitis, the sample size was too small for analysis.

### Atopic Respiratory Conditions

**Figure 4:**
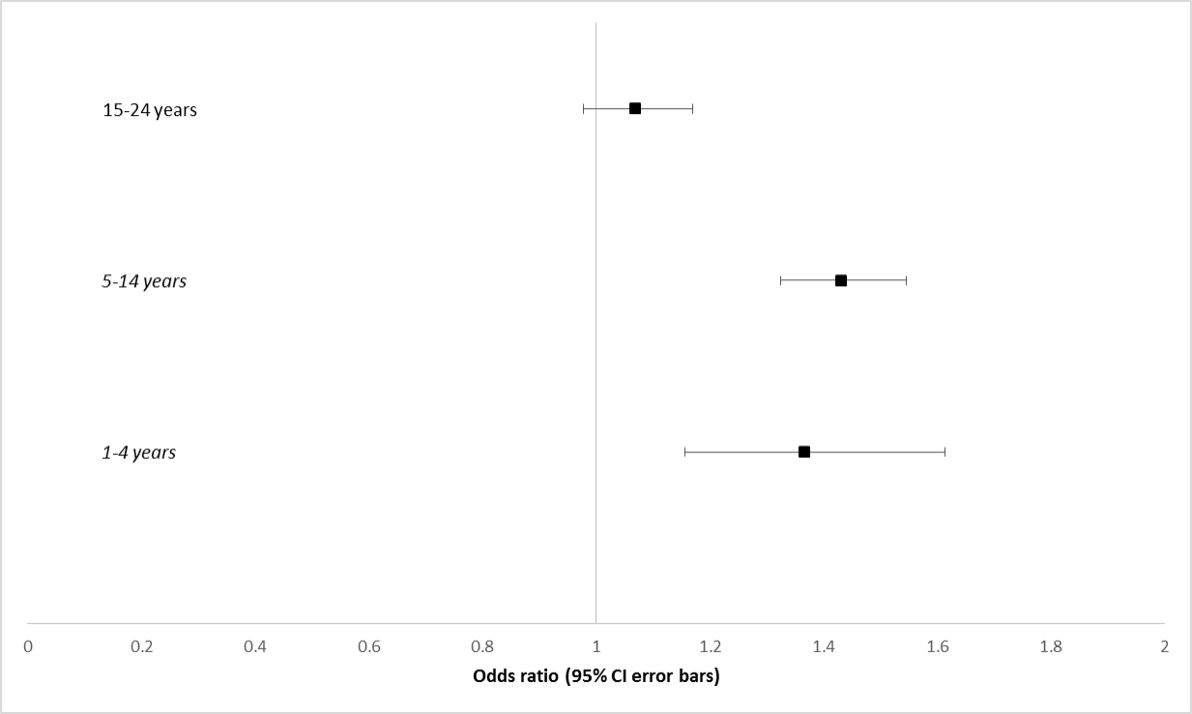
Forest plot of odds ratios of males younger than 25 years presenting with atopic respiratory conditions. Ages with statistically significant results are in italics. Age band under 1 has been excluded because of low sample size.

Compared with girls, boys aged 1-14 years had significantly greater odds of presenting with atopic respiratory conditions of AR and asthma (Figure 4). Under the age of 1 year, the sample was too small to conduct a regression (Table 3). For boys aged 1-4 years, OR of presenting with atopic respiratory conditions, compared with girls, was 1.37 (95% CI 1.16-1.61, *P*<.001); for boys aged 5-14 years, OR was 1.43 (95% CI 1.25-1.62, *P*<.001). For each individual condition, the gender differences were only significant for boys aged 5-14 years: OR 1.52 (95% CI 1.37-1.68, *P*<.001) for AR and OR 1.31 (95% CI 1.17-1.48, *P*<.001) for asthma.

The remaining conditions did not have significant results, with the exception of UTIs, where boys older than 1 year were less likely than girls to present with this infection (Table 4). Under the age of 1 year, the sample size was too low to run a regression. For boys aged 1-4 years, OR is 0.30 (95% CI 0.23-0.38, *P*<.001); for boys aged 5-14 years, OR is 0.14 (95% CI 0.11-0.18, *P*<.001); and for boys aged 15-24 years, OR is 0.04 (95% CI 0.03-0.06, *P*<.001).

Just under 1 in 20 boys <1 year presented with acute bronchitis (Table 1). The probability of a boy presenting with acute bronchitis decreased to about a third with each increasing age band (<1 year: 4.16%, 1-4 years: 1.42%, 5-14 years: 0.41%). For every 7 boys aged <1 year, 1 boy (15%) presented with a URTI (Table 2). There was a relatively little decrease until 5 years of age: 13.85%, 4.70%, and 2.45% for the 1-4 year, 5-14 year, and 15-24 year age bands, respectively. Although grouped with URTIs, conjunctivitis had a similar pattern of presentation to acute bronchitis, 4.48% in boys aged <1 year and 2.66% in boys aged 1-4 years. The probability of presenting with AOM peaked in the 1-4 year age band: incidence 2.51%.

**Table 1:** Odds ratios, 95% CI, *P* values, and probabilities of young males presenting with acute bronchitis, influenza-like illness (ILI), and lower respiratory tract infections and ILI (N=474,548).

Patients: age band and gender (N)		Acute bronchitis	ILI^a^	LRTI^b^+ILI
**<1year male, ref=Female (N=14,066)**				
	Patients, n	723	9	730
	OR	1.59	0.56	1.57
	95% CI	1.21-2.08	0.05-6.03	1.20-2.06
	*P* value	<.001^d^	.43	<.001^d^
	Adjusted probability, %	4.16	0.16	4.35
**1-4 years male, ref=Female (N=75,011)**				
	Patients, n	3794	187	3991
	OR	1.13	1.04	1.13
	95% CI	1.01-1.27	0.63-1.73	1.01-1.26
	*P* value	<.001^d^	.79	<.001^d^
	Adjusted probability, %	1.42	0.18	1.52
**5-14 years male, ref=Female (N=211,752)**				
	Patients, n	1699	716	2426
	OR	1.20	1.22	1.19
	95% CI	1.02-1.40	0.86-1.72	1.03-1.38
	*P* value	<.001^d^	.06	<.001^d^
	Adjusted probability, %	0.41	0.31	0.47
**15-24 years male, ref=Female (N=173,719)**				
	Patients, n	1835	380	2230
	OR	1.11	1.18	1.14
	95% CI	0.93-1.32	0.91-1.53	0.99-1.32
	*P* value	.05	.04	.02
	Adjusted probability, %	0.41	0.43	0.55

^a^ILI: influenza-like illness.

^b^LRTI: lower respiratory tract infection.

^c^OR: odds ratio.

^d^Indicates statistically significant, *P*<.001.

**Table 2:** Odds ratios, 95% CI, *P* values, and probabilities of young males presenting with upper respiratory tract infections and conjunctivitis (N=474,548).

Age band and gender (N)		Tonsillitis	Common cold	Sinusitis	Conjunctivitis	Acute otitis media	URTI^a^+conjunctivitis
**<1year male, ref=Female (N=14,066)**							
	Patients, n	98	2607	1	1144	251	3527
	OR^b^	1.22	1.09	N/A^c^	0.96	1.11	1.05
	95% CI	0.61-2.45	0.94-1.27	N/A	0.78-1.19	0.71-1.72	0-91-1.21
	*P* value	.34	.06	N/A	.55	.46	.26
	Adjusted probability. %	0.05	11.00	N/A	4.48	0.78	14.81
**1-4 years male, ref=Female (N=75,011)**							
	Patients, n	4144	15391	25	4592	5093	23654
	OR	1.05	0.97	0.44	1.06	1.07	1.01
	95% CI	0.95-1.18	0.91-1.04	0.11-1.76	0.96-1.18	0.97-1.18	0.96-1.07
	*P* value	.11	.14	.05	.07	.03	.41
	Adjusted probability, %	5.16	7.57	0.00	2.66	2.51	13.85
**5-14 years male, ref=Female (N=211,752)**							
	Patients, n	5609	4598	942	937	931	12363
	OR	0.81	0.93	0.92	1.05	0.93	0.89
	95% CI	0.74-0.88	0.86-1.00	0.58-1.44	0.89-1.24	0.82-1.04	0.85-0.94
	*P*-value	<.001^d^	<.001^d^	.54	.38	.03	<.001^d^
	Adjusted probability, %	5.65	1.68	0.10	0.51	0.89	4.70
**15-24 years male, ref=Female (N=173,719)**							
	Patients, n	6031	9036	221	1616	3468	2230
	OR	0.73	0.80	0.60	0.96	0.83	0.76
	95% CI	0.66-0.80	0.72-0.90	0.47-0.77	0.76-1.21	0.66-1.05	0.71-0.81
	*P* value	<.001^d^	<.001^d^	<.001^d^	.58	.01	<.001^d^
	Adjusted probability, %	2.98	0.77	0.20	0.15	0.29	2.45

^a^URTI: upper respiratory tract infection.

^b^OR: odds ratio.

^c^N/A: not applicable.

^d^Indicates statistically significant, *P*<.001.

The probability of a male child of 1 year or over presenting as an incident case of an atopic respiratory condition remains similar across age bands, just under 1 in 100 (0.80%-0.89%) each year (Table 3). Atopic incident cases are broadly equally split between AR and asthma, with the incidence of around 1 in 200 in these conditions individually. For the remaining conditions, the probabilities of boys presenting are under 1%.

Our sensitivity analysis produced very similar results to the original analysis (Multimedia Appendix 3). The only key differences were that OR of males aged 15 to 24 years attending with otitis media became significant (forest plots of the individual conditions are shown in Multimedia Appendix 4).

**Table 3:** Odds ratios, 95% CI, *P* value, and probabilities of young males presenting with atopic respiratory conditions of allergic rhinitis and asthma (N=474,548).

Age band and gender (N)		Allergic rhinitis	Asthma	Atopic respiratory disease
**<1year male, ref=Female (N=14,066)**				
	Patients, n	4	1	5
	OR^a^	N/A^b^	N/A	N/A
	95% CI	N/A	N/A	N/A
	*P* value	N/A	N/A	N/A
	Adjusted probability, %	N/A	N/A	N/A
**1-4 years male, ref=Female (N=75,011)**				
	Patients, n	277	368	636
	OR	1.32	1.39	1.37
	95% CI	0.87-1.99	0.97-2.00	1.04-1.80
	*P* value	.03	.003	<.001^c^
	Adjusted probability, %	0.30	0.40	0.80
**5-14 years male, ref=Female (N=211,752)**				
	Patients, n	1361	946	2280
	OR	1.52	1.31	1.43
	95% CI	1.29-1.79	1.08-1.59	1.26-1.63
	*P* value	<.001^c^	<.001^c^	<.001^c^
	Adjusted probability, %	0.50	0.50	0.89
**15-24 years male, ref=Female (N=173,719)**				
	Patients, n	1773	1243	2962
	OR	1.14	1.00	1.07
	95% CI	0.94-1.37	0.79-1.26	0.92-1.24
	*P* value	.02	.95	.14
	Adjusted probability, %	0.50	0.30	0.83

^a^OR: odds ratio.

^b^N/A: not applicable.

^c^Indicates statistically significant, *P*<.001.

**Table 4:** Odds ratios, 95% CI, *P* values, and probabilities of young males presenting with urinary tract infection and intestinal infectious disease (N=474,548).

Age band and gender (N)		UTI^a^	IID^b^
**<1year male, ref=Female (N=14,066)**			
	Patients, n	35	152
	OR^c^	0.71	0.88
	95% CI	0.22-2.27	0.50-1.53
	*P* value	.33	.45
	Adjusted probability, %	0.28	0.67
**1-4 years male, ref=Female (N=75,011)**			
	Patients, n	349	1088
	OR	0.30	0.95
	95% CI	0.19-0.45	0.77-1.17
	*P* value	<.001^d^	.44
	Adjusted probability, %	0.05	0.62
**5-14 years male, ref=Female (N=211,752)**			
	Patients, n	1495	511
	OR	0.14	1.07
	95% CI	0.09-0.21	0.83-1.37
	*P* value	<.001^d^	.41
	Adjusted probability, %	0.04	0.14
**15-24 years male, ref=Female (N=173,719)**			
	Patients, n	572	700
	OR	0.04	1.05
	95% CI	0.03-0.08	0.77-1.44
	*P* value	<.001^d^	.60
	Adjusted probability, %	0.03	0.10

^a^UTI: urinary tract infection.

^b^IID: intestinal infectious disease.

^c^OR: odds ratio.

^d^Indicates statistically significant, *P*<.001.

## Discussion

### Summary

Boys and young men were more likely to consult for LRTI and atopic conditions. Generally, the odds of presentation become less in older age bands, even if they remain significantly different. Our sensitivity analysis, excluding consultation where no action was taken, produced similar findings. In broad terms, the hypothesis that boys present more with LRTI and asthma up to puberty was supported by RCGP RSC data.

### Strengths and Limitations

This paper used the data collected for RCGP RSC’s WRS and Annual Report for the 2015-2016 season. RCGP RSC should have as good data quality as possible for infections, recognizing that routine data collected in the 10-min consultation has limitations [23]. Notwithstanding, the data quality of RCGP RS is considered the gold standard in primary care surveillance. This richness of research data available within a surveillance network can promptly produce initial insights into the epidemiology of a given disease, leading to more in-depth research.

Although the RCGP RSC network only covered 2.8% of the English population, it has been shown to be representative [1]. RCGP RSC is smaller than many of the other widely known UK primary care databases available for research. These include the Clinical Practice Research Data-link [24], the Health Improvement Network [25], and QResearch [26]. All have similarities, including the potential to link to other data such as hospital records and death data.

RCGP RSC differs in several ways. First, in their original form, most of the other databases were derived from a single brand of CMR system, whereas RCGP RSC extracts data from all the clinical systems. This creates issues around difference in version of coding system between brands [27] as well as related to their degree of problem orientation, which we adjusted for in our analysis [28]. Second, RCGP RSC data are probably the freshest of the data sources, perhaps inevitably so, given its surveillance function. Data extracted up to the end of the previous week are analyzed by Wednesday noon and are presented in the public domain by 2 PM on Thursday of the following week.

### Comparison With Existing Literature

Although there is an overall propensity for males to consult less than females [29]; we did not see this in children or young adults. Generally, the literature around gender differences supports the hypothesis that boys are more prone to respiratory conditions and other infectious diseases, rather than this being a sociological phenomenon. There appears to be evidence that young males may be more susceptible to respiratory infections, respiratory symptoms, and hospitalization.

Acute respiratory infections, including tonsillitis, resulting in hospitalization of boys, have been shown to be 2.4 times higher in India [30]. An interesting study of wheeze from birth to adolescence showed a U-shaped difference in presenting with wheeze between boys and girls, with similar levels of presentation with wheeze of girls near birth and late adolescents. This is compatible with our findings that boys aged 1-14 years have an excess of asthma, a condition generally associated with wheeze [31]. Additionally, a questionnaire study reported that asthma and AR were more prevalent in boys aged 6 to 7 years, but with the exception of asthma, most health symptoms were more prevalent in girls aged 13 to 14 years [32]. A further study also described gender differences that were not explained by the presence of atopy [33]. A study of AR also reported a male excess in childhood, with females “growing into” AR in adolescence [34]. Our findings are also reinforced by a study of AR and asthma (in Brazil) in 6 and 7 year olds, reporting an excess of males [35].

The excess in infections in boys and young men may be reflected through into hospital admissions. Male vulnerability to infection, in terms of more boys admitted, has been seen across many disease categories (in Singapore) in which they report disparities of presentation to primary care [36]. A Danish study also showed disparity in admission in favor of boys, but that the reverse applied in adolescents and adults aged 15-25 years [37].

Not all studies support our findings. A study of URTIs including AR for ambulatory ear, nose, and throat practice found no gender difference in presentation [38]. Similarly, a study that followed up 294 children for a year showed no difference in rates of URTI or between genders [39].

The mechanism for such differences has only been hypothesized, with hormonal and epigenetic mechanisms having been proposed [40,41]. Differences in inflammatory markers might provide some insight. A study reported differences in inflammatory markers; white cell responses were longer in boys, whereas duration of fever was longer in girls [42].

### Implications for Research and Practice

If it can be shown that boys and young adult males respond differently to infections than females and have different patterns of presentation; this will have important implications in health care provision across genders.

The preponderance of male presentations did not go away when we adjusted for the consultation rate (as a proxy of propensity to consult), nor when we adjusted for consultations at which no action was taken, as well as other key demographic variables. We see disease presentation as a complex bio-psychosocial phenomenon [43,44], and we conclude that although there may be important sociological contributions, it is more likely that genetic and hormonal reasons account for these differences in consultation rates.

Further research is needed at the specific disease level to explore this.

### Conclusions

RCGP RSC has a long history as a surveillance network providing data about infections and respiratory illness. The Annual Report (Multimedia Appendix 1) contains important details about presentation across a wide range of conditions; it is extended in the 2015/16 report to include more details about the age-sex differences in disease presentation. Boys and young male adults appear to have greater odds of presenting with some infections and respiratory conditions. This phenomenon has not been reported from a substantial population group, such as RCGP RSC; confirmation of this observation and understanding its mechanism may enable us to tailor guidelines to gender differences.

## Appendix

Multimedia Appendix 1 This Annual Report is based on the data that we extract from more than 150 GP practices and draws together the principal elements of our work—disease surveillance, virological sampling, and vaccine effectiveness.

Multimedia Appendix 2 Read2 and CTV3 read code lists.

Multimedia Appendix 3 Odds ratios, 95% CI, P values, and probabilities of males presenting with infections and respiratory conditions by age band. This table is adjusted for whether any action was taken at the consultation. The * indicates statistically significant, *P*<.001.

Multimedia Appendix 4 Forest plots of the individual conditions; statistically significant results are in italics.

## Abbreviations

AR: allergic rhinitis
AOM: acute otitis media
CMR: computerized medical records
GP: general practitioner
IID: intestinal infectious disease
ILI: influenza-like illness
IMD: Index of Multiple Deprivation
LRTI: lower respiratory tract infection
OR: odds ratios
QOF: quality and outcomes framework
RCGP: Royal College of General Practitioners
RSC: Research and Surveillance Centre
URTI: upper respiratory tract infection
UTI: urinary tract infection
WRS: Weekly Returns Service
