# Supplementary material for: Incidence of Lower Respiratory Tract Infections and Atopic Conditions in Boys and Young Male Adults: Royal College of General Practitioners Research and Surveillance Centre Annual Report 2015-2016
Source: JMIR Public Health Surveill. 2018 Apr 30;4(2):e49. doi: 10.2196/publichealth.9307 (PMC5952117; doi:10.2196/publichealth.9307)
Supplement: Multimedia Appendix 1 [file publichealth_v4i2e49_app1.pdf]

REGISTERED
CHARITY

NUMBER 223106

RCGP

Royal College of General Practitioners

Annual Report 2015-2016

Research & Surveillance Centre

Weekly Returns Service Annual Report 2015-2016

Content

1. Introduction 3
2. What we do – and why 4
   Measuring vaccine effectiveness 4
   Monitoring disease incidence 4
   Collecting samples of viruses and disease-causing organisms 4
   Identifying disparities 5
   Helping to change clinical practice 5
3. The RSC dataset 6
   Our data extraction process and information governance 6
   The dataset used for the RSC Annual Report 2015/16 6
   Reporting period 6
4. Sample population 7
   Age Sex Breakdown, National 7
   Age Sex Breakdown by NHSRegion 7
   Age Sex Profile 7
   Distribution of RSC practices among NHS regions in England 8
5. Influenza 9
   Swabbing programme 9
   Vaccine uptake 10
   Vaccine effectiveness 11
6. Disparities 13
   Age 13
   Gender 13
   Ethnicity 13
   Deprivation 14
   Disparity graphs for all conditions 15
7. Disease incidence 19
8. Episode typing – key part of data quality 21
9. Current Projects 22
   European Projects 22
   Other Projects 22
10. RCGP Publications 2015/16 23
11. Participating practices 24
12. Contributors 29

13. Appendix A: Weekly disease incidence graphs 30
14. Appendix B: Demographic distribution by condition 50
15. Appendix C: Data tables 88

1. Introduction

Welcome to the Royal College of General Practitioners (RCGP) Research and Surveillance Centre's (RSC) Annual Report for 2015-16. This is written as we enter our 50th year of surveillance.

This Annual Report is based on the data that we extract from more than 150 GP practices and draws together the principal elements of our work – disease surveillance, virological sampling, and vaccine effectiveness. It covers the period from the beginning of May 2015 to the end of April 2016, which includes the whole of the winter flu season 2015-16.

We would like to thank all the practices who form our network, and of course all their patients, without whose data (which we utilise in an anonymised format) our work would be impossible. Sharing of medical data has become a controversial topic over recent years, but without the support of our member practices and the use of their patients' data we would not be able to carry out our crucial work of monitoring disease rates, assuring the effectiveness of national vaccination programmes, and participating in other research programmes.

Influenza surveillance is the biggest part of our work. This year, we are also involved in research testing enhanced surveillance for gastroenteritis, within a Wellcome Trust/Department of Health funded project called Integrate[1]. This involves the near real-time detection of many of the causative organisms for this condition. We recruited over 60 practices into this study. We are hoping that gastroenteritis surveillance might become an activity of the network.

The RCGP RSC has had a couple of years of immense change including a re-design and rebuilding of its data extraction systems and creation of a new data and analytics hub at University of Surrey. This report is based on an extract taken in 2016; the report therefore includes a larger population. In May 2016, we improved the data quality process to import data – the data extracted for this report has been processed in this way, which means that the incidence graphs might differ slightly from the weekly surveillance reports during the 2015-2016 season.

The RCGP RSC works in close collaboration with Public Health England (PHE). The principal source of funding of the RCGP RSC is from PHE as their principal primary care surveillance element. This surveillance contributes to decisions about when flu starts to circulate in the community. The Chief Medical Officer can then state whether there is circulating influenza, which triggers permission being given to GPs to prescribe oral medication for influenza, where they feel appropriate. The RCGP RSC is also looking out for any signs of an influenza pandemic, or any other unexpected rise across a range of diseases.

The innovation we have added to this year's report are new graphics displaying how the age, gender, ethnicity and deprivation varies across the conditions we monitor (See pages 15 to 18). We hope these graphics will provide insights into patterns of health and disease. The accompanying paper in the British Journal of General Practice describes how boys and young adults present more to primary care for many infective and atopic conditions.

[1] http://www.integrateproject.org.uk/

2. What we do – and why

For nearly 50 years, the RCGP's Research and Surveillance Centre has used data from GP practices across England to monitor disease, measure vaccine effectiveness, and for research. The RCGP RSC is the principal primary care research surveillance system for England; for example, its data makes a major contribution to the announcement by the Chief Medical Officer that there is circulating influenza. This announcement provides the cue to practitioners that they can prescribe oral antiviral treatments for influenza. The robust evidence that we produce enables public health agencies and others to make the best possible decisions in preventing disease and responding to epidemics; alongside this, we contribute to a range of research studies.

Measuring vaccine effectiveness

Vaccination helps protect populations from disease and saves lives every year. Data from RCGP RSC practices is crucial for ensuring that vaccines are as effective as possible:

- The childhood flu vaccination programme was introduced in 2013/14 following successful pilots. This is also known as the live attenuated influenza vaccine (LAIV) programme. Data from RCGP RSC practices provided evidence that the pilot scheme should be rolled out across the country, and is being used to monitor the ongoing effectiveness of the scheme. (See p. 9.)
- Data from RCGP RSC practices also helps to monitor the effectiveness of the seasonal flu vaccine for adults. (See p. 11.)
- Disease does not stop at national borders – international collaboration is key in maximising vaccine effectiveness. We are involved in two projects to put into place European frameworks that will help health professionals, regulatory agencies, public health institutions, vaccine manufacturers, and the general public make more informed decisions on the benefits and risks of vaccines. (See p. 22.)

Monitoring disease incidence

We monitor 37 communicable and respiratory diseases on a weekly basis. Our data feeds into PHE's surveillance system and gives an early warning of epidemics or unusual patterns of disease. Our monitoring warns of seasonal events that place pressure on health services, such as the start of the flu season, and allow services to be planned accordingly.

Collecting samples of viruses and disease-causing organisms

RSC practices take samples from patients that are used to increase our understanding of disease and how to combat it:

- Flu virology swabs, taken within 7 days of onset, to establish which flu strain is dominant in the current season; and also to infer the strain likely to be dominant the following flu season, so that decisions can be made on how the flu vaccine can be improved in order to target these specific strains. (See p. 9.)
- Analysis of stool samples from the Integrate Gastroenteritis Project will enable much more advanced and rapid identification of organisms responsible for vomiting and diarrhoea. (See p. 22.)

Identifying disparities

The richness of clinical data allows us to identify demographic factors around clinical presentations. For this report, we present the distribution of age, gender, ethnicity, and deprivation for each condition, compared to the larger RSC population. The aim of this is to understand any disparities in clinical presentation, concerning key demographic characteristics. This should guide clinical practice in identifying at-risk groups, and provides a strong basis for public health interventions.

Helping to change clinical practice

The RCGP RSC relies on practices agreeing to share their anonymised patient data with us – and, in return, their aggregated data provides evidence for research that is used to improve clinical practice and patient outcomes. The Diabetes Real World Evidence (RWE) Centre, for instance, has explored adherence to and persistence with different classes of anti-diabetes medicines and the thresholds at which different clinicians implement injectable therapy in type 2 diabetes (T2DM)[2 3 4 5 6]. Currently, the work of the RWE centre is focused around demographic disparities in the care of people with T2DM, and targeted interventions around the risk of macrovascular complications.

---

[2] McGovern A, Hinton W, Tippu Z, Whyte M, de Lusignan S. Ethnic disparities in medication persistence in type 2 diabetes: Non-whites have reduced persistence [Abstract]. Diabetes (June 2016). 65 (Supplement 1): A23

[3] McGovern A, Hinton W, Munro NM, Whyte MB, de Lusignan S. Do persistence rates vary between dipeptidylpeptidase-4 inhibitors? [Abstract]. Diabetes (June 2016). 65 (Supplement 1): A576

[4] McGovern A, Hinton W, van Vlymen J, Munro N, Whyte M, de Lusignan S. Real world evidence on the prescribing trends in sodium glucose co-transporter 2 inhibitors in UK primary care [Abstract]. Diabetic Medicine (March 2016). 33 (Supplement 1): P165.

[5] Hinton W, McGovern A, van Vlymen J, Munro N, Whyte M, de Lusignan S. Real world evidence on the prescribing trends of glucagon-like peptide-1 agonists in UK primary care [Abstract]. Diabetic Medicine (March 2016). 33 (Supplement 1): P165.

[6] McGovern A, Hinton W, van Vlymen J, Munro N, Whyte M, de Lusignan S. Real world evidence on the disparities of prescribing of dipeptidyl peptidase-4 inhibitors in UK primary care [Abstract]. Diabetic Medicine (March 2016). 33 (Supplement 1): P183.

3. The RSC dataset

Our data extraction process and information governance

Data are extracted twice weekly from practice systems by Apollo Medical Systems, a well-established data extraction company, on the RCGP's behalf. Patients who have withheld consent for data sharing, for any reason, are excluded from our analyses. These patients are identified through an opt-out code, which the automatic extraction process uses to exclude them.

Data are pseudonymised (this is a process that scrambles any strong identifiers such as name, NHS number, and date of birth) as close to source as possible. The pseudonymised data are held on secure servers at the RCGP RSC data and analytics hub in the Section of Clinical Medicine and Ageing at the University of Surrey. Both Apollo and the University of Surrey are fully compliant with NHS data governance rules.

The dataset used for the RSC Annual Report 2015/16

The dataset used in this report was extracted in May 2016. It includes 1,589,702 patients from 155 practices. This includes all patients who were registered for at least one week during the reporting period, within a practice from the RCGP RSC network for which we received data. There is one exception to this: the dataset used to calculate influenza vaccine coverage. Swabbing results and vaccine effectiveness were defined differently for the specific purposes of the end of influenza season report.

For the week-by-week disease incidence graphs, we include episodes of disease in the numerator if the patient was validly registered with a practice within the RCGP RSC network at the time of the episode. The denominator include all registered patients for that particular week.

Reporting period

This report covers the period 4 May 2015 – 8 May 2016 (International Standards Organisation (ISO) Week 19 of 2015 to ISO Week 18 of 2016). It covers this period, rather than a calendar year, in order to ensure that one complete summer season and one complete winter season are included in our analysis. This year, there were 53 weeks instead of 52; this meant that for our four-weekly reporting in the Appendix C tables, we have a period (W1) with 5 weeks.

4. Sample population

This section shows the demographic (age and gender) breakdown of our sample population, and how it compares with that of England as a whole. The sample population includes all patients with a registration date prior to 4 May 2015, and either no deregistration date or a deregistration date after 8 May 2016 (i.e. those patients registered throughout the data period covered by this report).

Age Sex Breakdown, National

Gender	<1yr	1-4yrs	5-14yrs	15-24yrs	25-44yrs	45-64yrs	65-74yrs	75-84yrs	85+yrs
Female	6,898	36,856	85,020	108,259	235,369	189,439	73,532	45,580	26,320
Male	7,168	38,155	88,699	103,493	231,595	193,002	68,857	37,240	14,220

Age Sex Breakdown by NHSRegion

NHS Region	Gender	<1yr	1-4yrs	5-14yrs	15-24yrs	25-44yrs	45-64yrs	65-74yrs	75-84yrs	85+yrs
North	Female	2,071	10,383	25,308	39,649	65,010	63,038	25,789	15,841	8,564
	Male	2,190	10,935	26,307	37,145	67,487	64,157	24,500	13,040	4,555
South	Female	1,584	8,152	20,119	27,219	49,594	47,855	19,721	12,270	7,525
	Male	1,596	8,579	21,245	27,310	50,539	48,180	18,665	10,050	4,049
Midlands And East	Female	1,236	8,665	21,117	20,265	48,153	47,581	19,517	12,065	7,478
	Male	1,288	8,814	22,104	20,863	46,692	48,006	18,301	9,999	3,991
London	Female	2,007	9,656	18,476	21,126	72,612	30,965	8,505	5,404	2,753
	Male	2,094	9,827	19,043	18,175	66,877	32,659	7,391	4,151	1,625
National	Female	6,898	36,856	85,020	108,259	235,369	189,439	73,532	45,580	26,320
	Male	7,168	38,155	88,699	103,493	231,595	193,002	68,857	37,240	14,220

We work very hard to ensure that the demographics of our sample population closely resemble those of the country as a whole. This correspondence can be seen in the age-sex profile below; the bars show the number of patients within our dataset, broken down by gender and age-band, and the lines show the distribution of the corresponding population of England as a whole. The 0-5 age group tends to be underrepresented as registration does not generally take place until a little while after birth.

Age Sex Profile

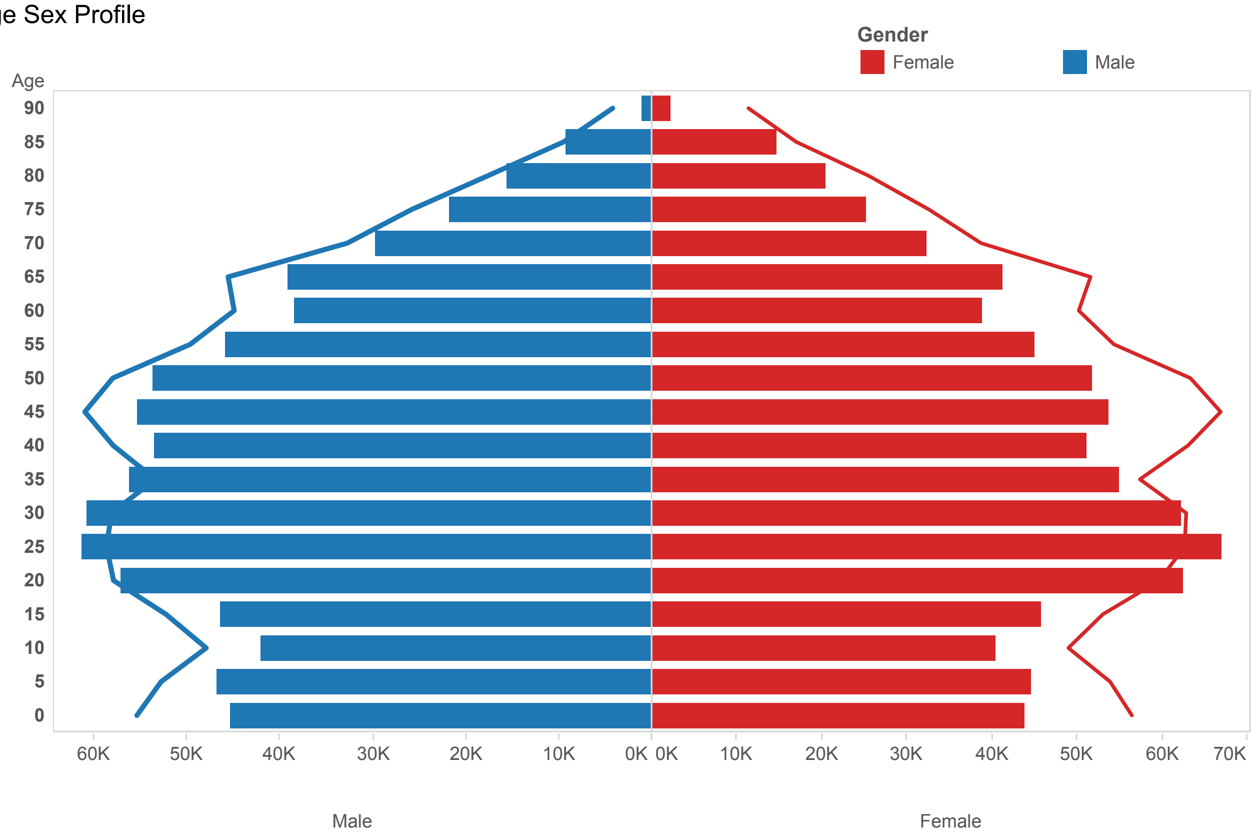

As can be seen from the map below, practices within the Network are spread across England in order to reflect the distribution of the population as a whole. We vary our priorities for recruiting to try to ensure an even spread of practices. We largely recruit new practices through the RCGP Research Ready network.

Distribution of RSC practices among NHS regions in England

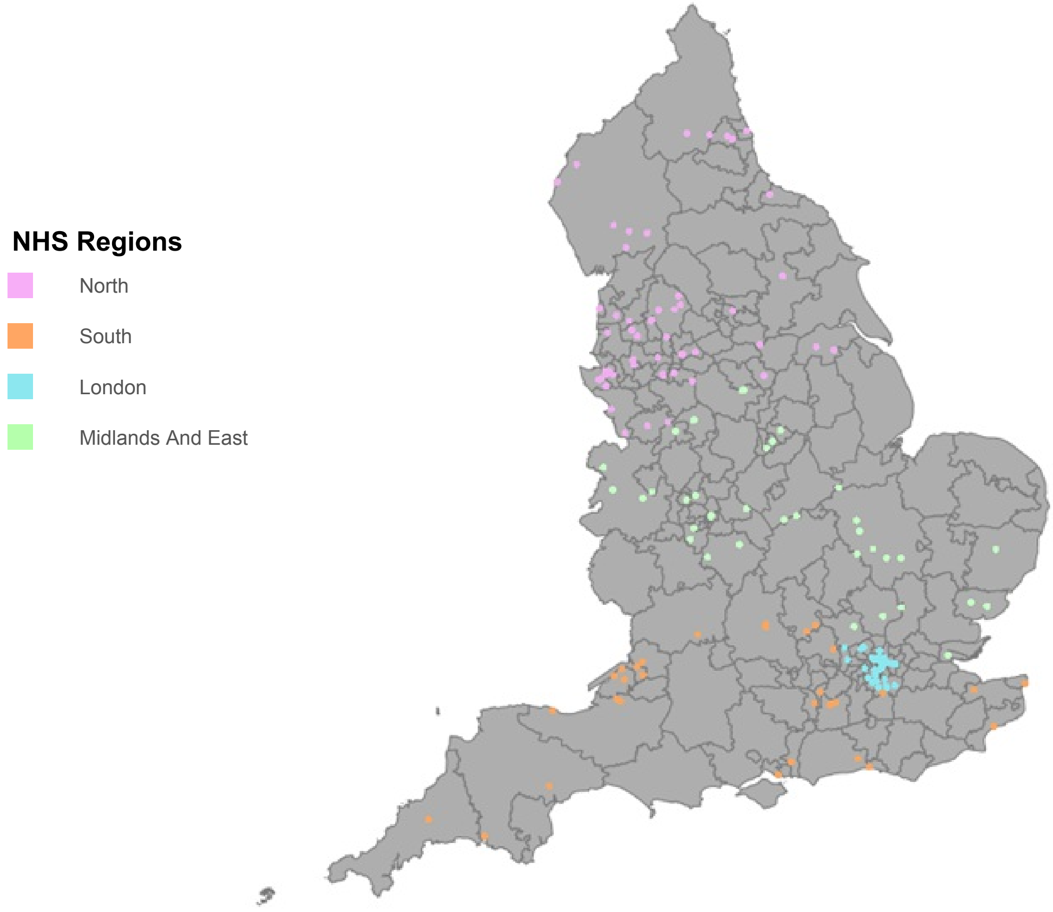

5. Influenza

Work on influenza is a central part of the RCGP RSC's activity. In addition to surveillance of weekly incidence of influenza-like illness (ILI), we conduct a programme of virological swabbing throughout the flu season and conduct analysis of vaccine uptake and effectiveness.

Swabbing programme

A total of 2,414 swabs were taken during the season. These are summarised in the table below, which shows positivity rates by age band.

Positivity rate by age band

Age Band	Total Samples	Positive for H3	Positive for Flu B	Positive for any virus
**<1**	41 (2%)	0 (0%)	1 (2%)	26 (63%)
**01-4**	168 (7%)	1 (1%)	11 (7%)	94 (56%)
**05-14**	227 (9%)	2 (1%)	42 (19%)	65 (29%)
**15-24**	284 (12%)	7 (2%)	54 (19%)	62 (22%)
**25-44**	799 (33%)	5 (1%)	64 (8%)	221 (28%)
**45-64**	616 (26%)	5 (1%)	20 (3%)	181 (29%)
**65-74**	168 (7%)	0 (0%)	10 (6%)	34 (20%)
**75-84**	92 (4%)	3 (3%)	1 (1%)	15 (16%)
**85+**	15 (1%)	1 (7%)	0 (0%)	3 (20%)
**Unknown**	4 (0%)	0 (0%)	0 (0%)	1 (25%)
**Total**	2414 (100%)	24 (1%)	203 (8%)	702 (29%)

The graph below summarises the season's virology coupled with all-age ILI incidence[7], and allows a comparison between this season and last season's incidence rates.

The number of swabs peaked in Week 11, with 171, when the ILI rate was at its highest. Both Influenza A(H1N1)[8] and Influenza B[9] were in circulation during the season, with Influenza B showing a later peak.

[7] Incidence is the number of new cases per 100,000 population registered with RCGP RSC practices.
[8] H1N1 is a subtype of influenza A known to cause pandemics in humans.
[9] Influenza B infections are of one subtype, generally influenza B follows influenza A each winter season.

Influenza Swabbing Surveillance

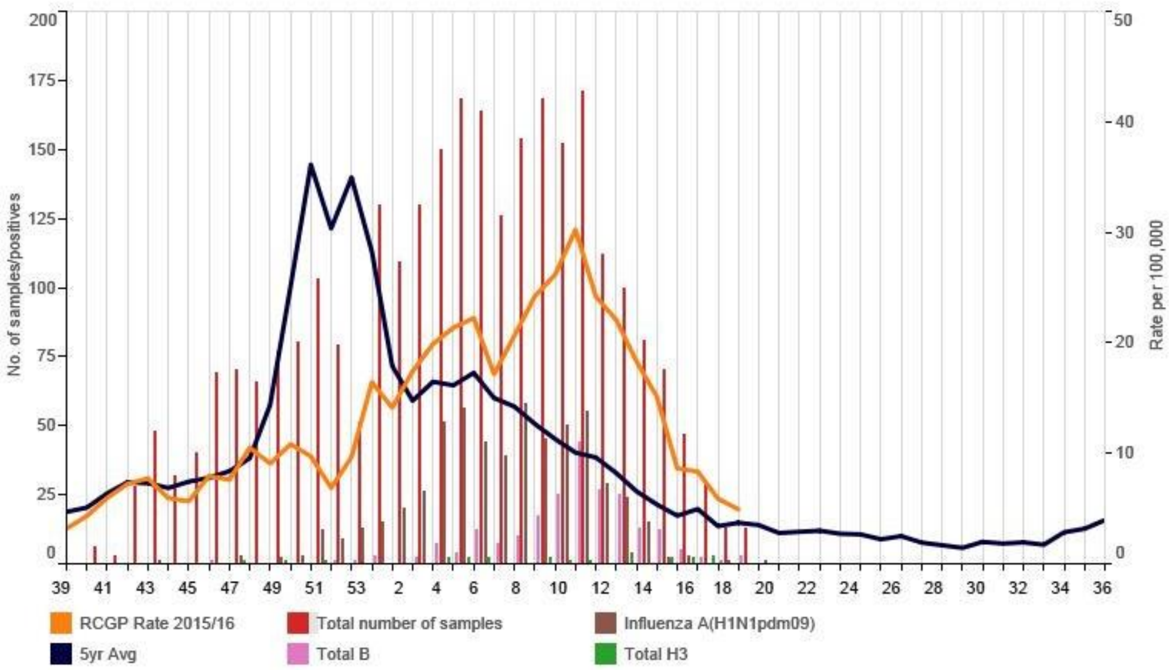

Vaccine uptake

The table below shows influenza vaccine coverage, as observed in our dataset. In addition to vaccine coverage across all patients, we have measured vaccine coverage among the following key populations, who were offered the influenza vaccination in 2015/16:

- Children aged two, three or four eligible for the Live Attenuated Influenza Vaccine (LAIV) at the GP surgery[10]. Additionally, we are including children in Year 1-2 (aged five to seven) who are eligible for LAIV vaccine at their school[11].
- Those aged 65 and over
- Patients aged under 65 in a clinical risk group (as defined below)

Our results show different vaccine coverage rates than those reported by Public Health England (PHE).[12] In line with PHE's reporting, uptake increases with age and is greatest among the older population.

[10] LAIV is a nasal spray flu vaccine. All LAIV is designed to protect against four different influenza viruses: Influenza A (H1N1), A (H3N2) and two influenza B viruses.
[11] This is a new change in the service specification for 2015/2016, and may not be recorded within the GP data, as the scheme operates in primary schools.
[12] Public Health England (2016) Influenza immunisation programme for England: GP patient groups. Data collection survey, Season 2015 to 2016 https://www.gov.uk/government/uploads/system/uploads/attachment_data/file/526033/Seasonal_flu_GP_patient_groups_annual_report_2015_2016.pdf

Influenza vaccine coverage

	Total eligible population	Vaccine coverage
**All patients**	1,276,590	22%
**Children eligible for LAIV (children aged 2-7)**	90,528	31%
**Patients aged under 65 in risk groups**	126,632	42%
**65 and over**	213,623	73%

This analysis is based on a sample of 138 practices for which we were able to extract data at the end of the flu season. Because the extract was taken earlier in the year than that used elsewhere in this Annual Report, our vaccine coverage figures are based on a smaller sample than the analysis in the rest of this report.

The risk factors included in this table are:

- Asplenia or Dysfunction of the spleen
- Asthma
- Chronic Heart Disease (CHD)
- Chronic Kidney Disease (CKD)
- Chronic Liver Disease
- Chronic neurological disease (including Stroke/Transient Ischaemic Attack, cerebral palsy and Multiple Sclerosis)
- Chronic Respiratory Conditions
- Diabetes
- Immuno-suppression

It should also be noted that vaccinations administered in settings other than GP practices are likely to be inconsistently recorded in the GP record. Therefore, the vaccine coverage data given here is likely to be a slight underestimate of the true rate.

Vaccine effectiveness

RCGP RSC data was one of the primary data sources for PHE's evaluation of influenza vaccine effectiveness (VE) during the 2015/16 season. The text below is taken from the published evaluations.

'End-of-season vaccine effectiveness (VE) showed an overall adjusted VE of 52.4% (95% confidence interval (CI) 41.0, 61.6) against influenza confirmed primary care consultation; VE of 54.5% (95% CI: 41.6, 64.5) against A(H1N1)pdm09 and 54.2% (95% CI 33.1, 68.6) against influenza B. In 2-17 year olds, adjusted VE for LAIV against any influenza was 57.4% (95% CI -8.0, 68.5); 81.4% (39.7, 94.3) against influenza B and 41.5% (-8.5, 68.5) against influenza A(H1N1)pdm09. These estimates demonstrate moderate to good levels of protection, particularly against influenza B in children, though less against A(H1N1)pdm09, which in the light of recent US reports needs further investigation. Despite influenza B lineage mismatch in the trivalent IIV, younger adults <65 years

were still protected against influenza B. These results provide reassurance for the UK to continue its current influenza immunisation programme planned for 2016/17.' [13]

[13] Pebody R, Warburton F, Ellis J, Andrews N, Potts A, Cottrell S, Johnston J, Reynolds A, Gunson R, Thompson C, Galiano M, Robertson C, Byford R, Gallagher N, Sinnathamby M, Yonova I, Pathirannehelage S, Donati M, Moore C, de Lusignan S, McMenamin J, Zambon M. Effectiveness of seasonal influenza vaccine for adults and children in preventing laboratory-confirmed influenza in primary care in the United Kingdom: 2015/16 end-of-season results. Euro Surveill. 2016Sep 22;21(38):pii=30013. http://www.eurosurveillance.org/ViewArticle.aspx?ArticleId=22592

6. Disparities

We explored disparities by condition, and in comparison to the overall RSC population. The network has been previously shown to be representative of the English population[14]. We examined whether people who presented with a given condition in the previous year had different demographic characteristics (age, gender, ethnicity, deprivation) than the general population. We present the distribution of these variables, with confidence intervals, for each individual condition in the appendix B graphs. We also include these variables for all conditions in the graphs below.

Age

We obtained the age for each registered patient on May 1st, 2016. We found the following patterns:

- Common childhood conditions, such as chicken pox and acute otitis media, had a lower median than the general population.
- Common conditions seen in the elderly, such as herpes zoster and pneumonia, had a higher median than the general population.
- The median age for influenza-like illness had a similar median age as that of the general population.
- Common cold, although a condition that affects all ages, had a median age below the population median, probably indicating that it is a condition with a high consultation rate for the young.

Gender

Gender is recorded by the GP on each patient's electronic record, when they first register in a practice. We found the following patterns:

- Overall, more women presented with conditions than men, reflecting their higher propensity to consult.
- Women presented more often than men with urinary tract infections and herpes simplex.
- Men presented more often than women with viral hepatitis and mumps, although the sample sizes were smaller.
- Almost 60% of influenza-like illness consultations were for women.

Ethnicity

Ethnicity is recorded on a proportion of patients. We have developed an algorithm, which incorporates languages spoken and other recorded fields, to generate the most probable ethnicity of

[14] Correa A, Hinton W, McGovern A, van Vlymen J, Yonova I, Jones S, de Lusignan S. Royal College of General Practitioners Research and Surveillance Centre (RCGP RSC) sentinel network: a cohort profile. BMJ Open. 2016 Apr 20;6(4):e011092. doi: 10.1136/bmjopen-2016-011092. PubMed PMID: 27098827; PubMed Central PMCID: PMC4838708.

a patient[15]. The ethnic groups used are those in the 2011 census (asian, black, mixed, other, white). We found the following patterns:

- More non-white patients presented with allergic rhinitis and chicken pox, compared to the overall population.
- More white patients presented with herpes zoster, infectious mononucleosis, and pleurisy, compared to the overall population.
- There were slightly more influenza-like illness consultations from non-white people, compared to the overall population.

Deprivation

We determined deprivation using the Index of Multiple Deprivation (IMD)[16], which assigns a score to each Lower Super Output Area (LSOA) in England[17]. When we extract our data, each patient's postcode is converted to LSOA. The lower the score, the least deprived the area where the patient lived. We found the following patterns:

- Patients presenting with viral hepatitis and scabies had a higher median IMD score (more deprived) than the overall population.
- Patients presenting with herpes zoster and infectious mononucleosis had a lower median IMD score (less deprived) than the overall population.
- Patients presenting with influenza-like illness had a slightly higher median IMD score (more deprived) than the overall population.

[15] Tippu Z, Liyanage H, Correa A, Burleigh D, McGovern A, Jones S, de Lusignan S. (2016) Ontologies to improve the identification of ethnicity in people with type 2 diabetes [Poster]. Diabetes UK Professional Conference. Glasgow, UK. March 2-4th, 2016.
[16] Department for Communities and Local Government (2015). The English Indices of Deprivation 2015. Available at: https://www.gov.uk/government/statistics/english-indices-of-deprivation-2015
[17] Office of National Statistics (2011). Postcodes (Enumeration) to output areas to lower layer SOA to middle layer SOA to local authority districts E+W lookup. Available at: https://geoportal.statistics.gov.uk/Docs/Lookups/Postcodes_(Enumeration)_(2011)_to_output_areas_(2011)_to_lower_layer_SOA_(2011)_to_middle_layer_SOA_(2011)_to_local_authority_districts_(2011)_E+W_lookup.zip

Disparity graphs for all conditions

Median Age

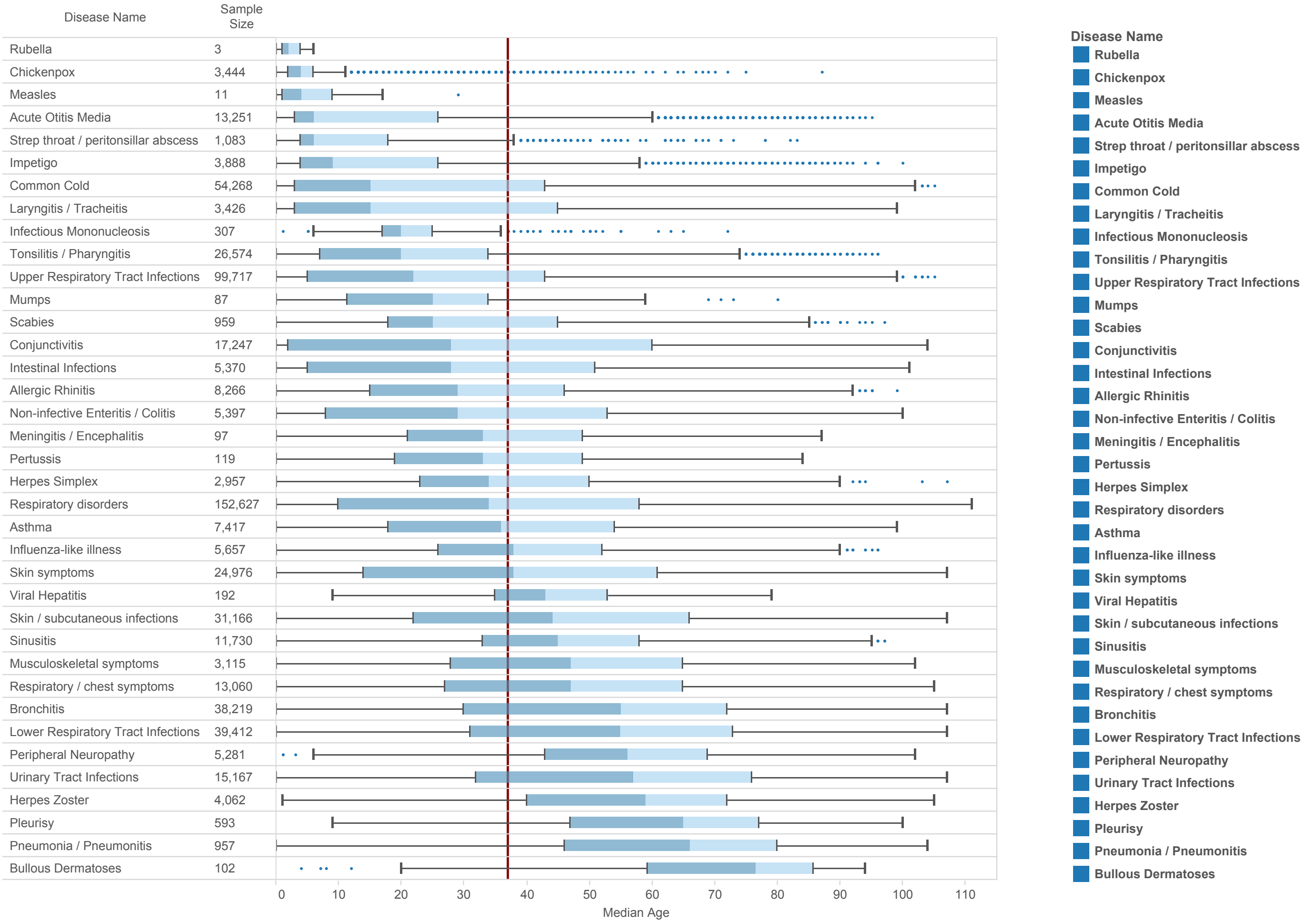

Grouped Gender

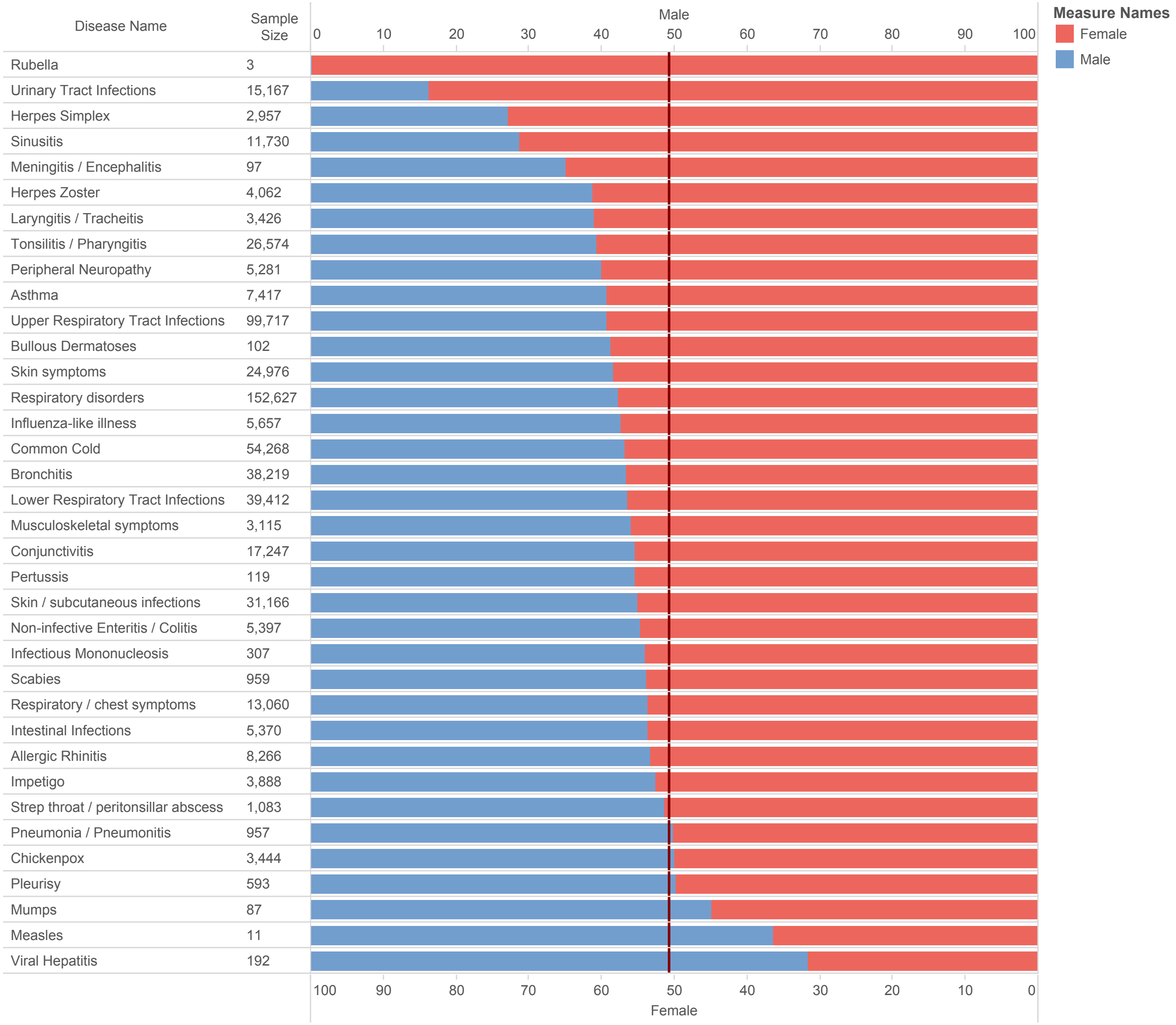

Grouped Ethnicity

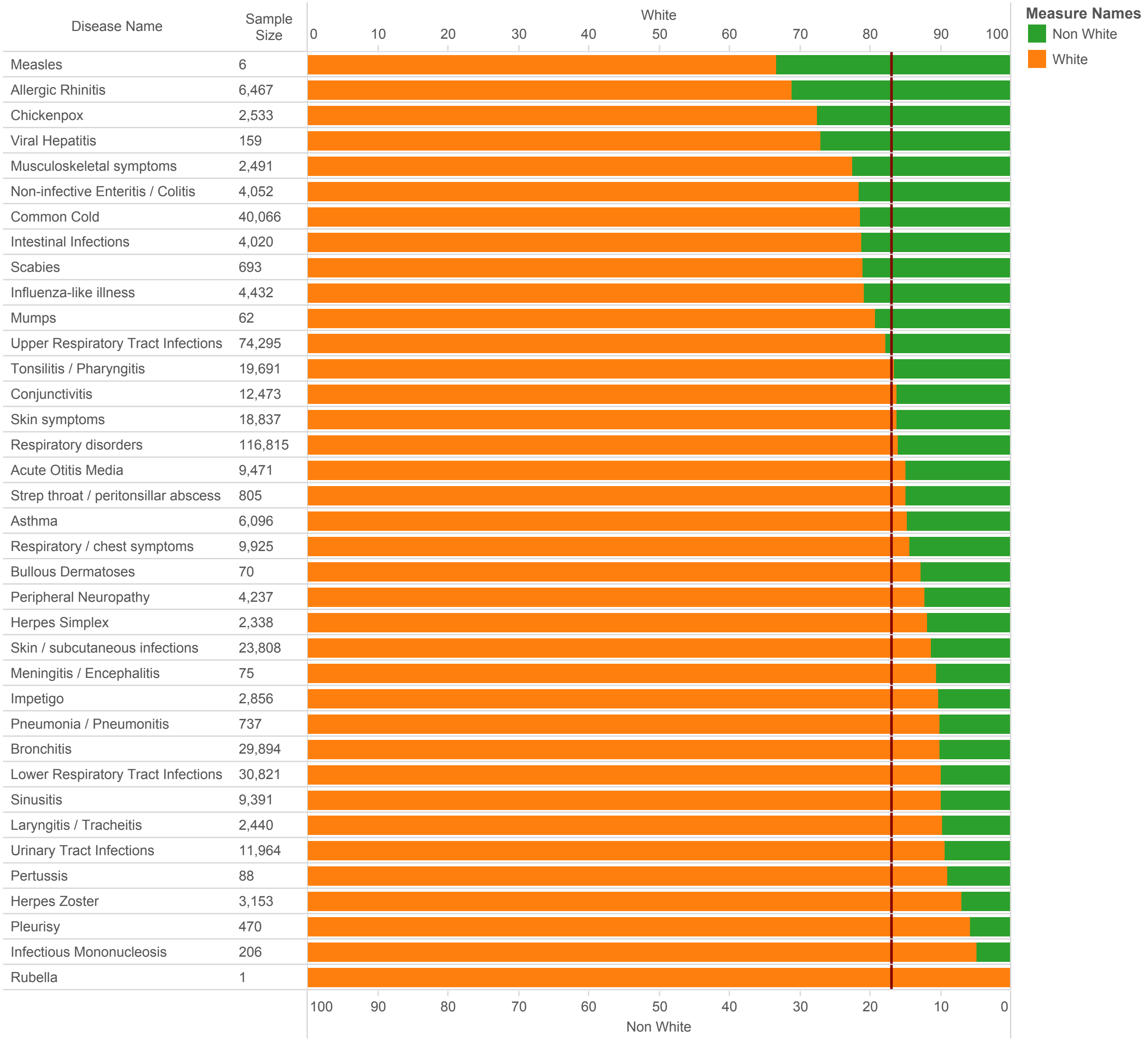

Median IMD

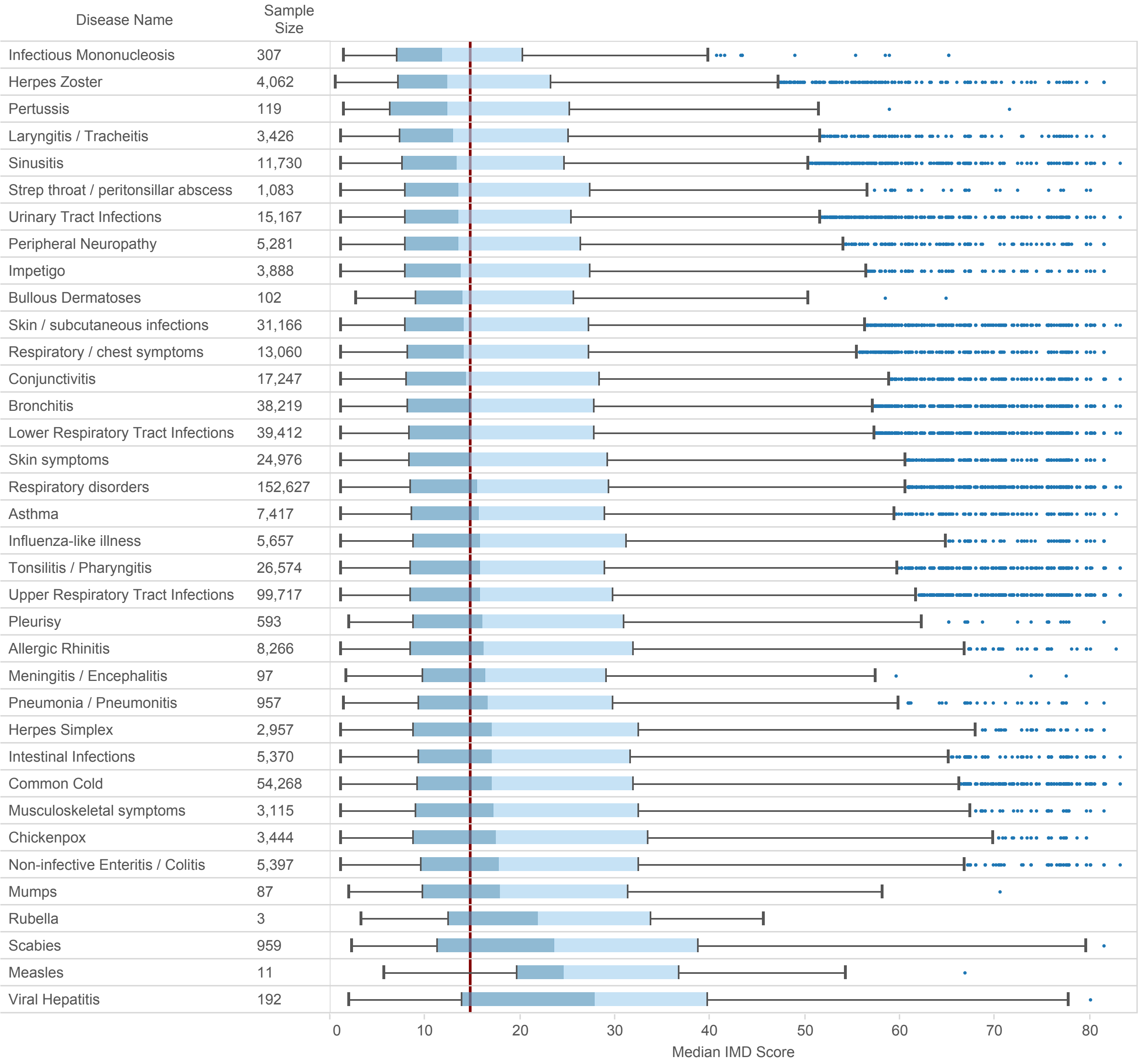
Disease Name
Sample Size
Infectious Mononucleosis 307
Herpes Zoster 4,062
Pertussis 119
Laryngitis / Tracheitis 3,426
Sinusitis 11,730
Strep throat / peritonsillar abscess 1,083
Urinary Tract Infections 15,167
Peripheral Neuropathy 5,281
Impetigo 3,888
Bullous Dermatoses 102
Skin / subcutaneous infections 31,166
Respiratory / chest symptoms 13,060
Conjunctivitis 17,247
Bronchitis 38,219
Lower Respiratory Tract Infections 39,412
Skin symptoms 24,976
Respiratory disorders 152,627
Asthma 7,417
Influenza-like illness 5,657
Tonsilitis / Pharyngitis 26,574
Upper Respiratory Tract Infections 99,717
Pleurisy 593
Allergic Rhinitis 8,266
Meningitis / Encephalitis 97
Pneumonia / Pneumonitis 957
Herpes Simplex 2,957
Intestinal Infections 5,370
Common Cold 54,268
Musculoskeletal symptoms 3,115
Chickenpox 3,444
Non-infective Enteritis / Colitis 5,397
Mumps 87
Rubella 3
Scabies 959
Measles 11
Viral Hepatitis 192
0
10
20
30
40
50
60
70
80
Median IMD Score

7. Disease incidence

We monitor the incidence of 37 conditions in our weekly disease surveillance reports, both nationally and for the four English NHS regions, in comparison with the 10-year average. Graphs showing weekly incidence of all monitored diseases are included in Appendix A.

These graphs are presented in the same format as in the RCGP RSC weekly surveillance report[18]. However, the graphs presented here are based on a single data extract taken after the end of the reporting period, which contains a larger sample of practices than it was possible to use in the weekly extracts. Additionally, we improved the data quality process to import data in May 2016, and the data extracted for this report has been processed in this way. Although the overall trends are the same, the rates for individual weeks may therefore show some variation from those published throughout the year.

Like the weekly surveillance report, the data are presented in this report by disease chapter. The key points for each chapter are:

- **Water and food-borne disorders.** Presentations of intestinal infectious diseases were at or below seasonal averages, with the exception of some small peaks in week 45 and weeks 9-10. Presentations of non-infective enteritis and colitis varied from lower incidence in the winter, and higher incidence in the summer. For both of these conditions, incidence was higher in London than other regions.
- **Environmentally-sensitive disorders.** Presentations of asthma remained high for most of the winter period, with incidence higher than seasonal expectations between January and March. The peak of allergic rhinitis was later than expected during the summer of 2015, rather than in the spring.
- **Respiratory infections.** Most respiratory infections had a peak later in the year than expected. Despite the temporal discrepancy, most peak rates did not exceed the 5 year average, with the exception of acute tonsillitis/pharyngitis, laryngitis/tracheitis, and strep throat/peritonsillar abscess. Presentations of strep sore throat and peritonsillar abscess were above expected levels for some of the year, and in particular towards the end of the reporting period. The incidence of influenza-like illness (ILI) peaked at 29 cases per 100,000 in week 11 2016; incidence in the South peaked a week later at 38 cases per 100,000. Incidence of common cold was consistently higher in London than other regions.
- **Vaccine-sensitive disorders.** Measles, mumps and rubella all showed low incidence throughout the year.
- **Skin contagions.** Chickenpox had a higher incidence than seasonally expected in the summer of 2015, with a lower incidence during the winter. Most other conditions remained at seasonally expected levels.
- **Disorders affecting the nervous system.** Presentations of peripheral nervous disease were at seasonally expected levels throughout the year.
- **Genitourinary system disorders.** Presentations of urinary tract infections were below the expected rate throughout the year.

[18] RCGP RSC Weekly Report. URL: http://www.rcgp.org.uk/clinical-and-research/our-programmes/research-and-surveillance-centre.aspx

As noted above, considerable regional variation can be seen for some diseases. For environmentally-sensitive disorders such as allergic rhinitis, this variation may reflect differing weather patterns across the country.

We will publish an accompanying paper exploring disease incidence in boys and young males of 11 selected conditions. Controlling for other key variables (ethnicity, deprivation, and propensity to consult), we examined the probability of boys in a certain age band presenting with:

- **Acute tonsillitis:** Across the 0-24 year age band, males had higher odds of presenting with tonsillitis – the probability was greater for the 1-4 years age band (2.2%).
- **Common cold:** Across the 0-24 year age band, males had higher odds of presenting with common cold – the probability was greater for the 1-4 years age band (7.7%).
- **Allergic rhinitis:** Across the 1-24 year age band, males had higher odds of presenting with allergic rhinitis – the probability was greater for the 5-14 years age band (0.9%).
- **Conjunctivitis:** Across the 1-14 year age band, males had higher odds of presenting with conjunctivitis – the probability was greater for the 1-4 years age band (3.1%).
- **Asthma:** Across the 1-14 year age band, males had higher odds of presenting with asthma – the probability was greater for the 5-14 years age band (0.9%).
- **Acute bronchitis:** Across the <1 year old and 5-14 year age band, males had higher odds of presenting with acute bronchitis – the probability was greater for the <1 years age band (2.6%).
- **Urinary Tract Infection (UTI):** Across the 0-4 year age band, males had higher odds of presenting with UTI – their combined probability (being male and young), however, was not particularly large; in the 15-24 age band males were less likely to present.
- **Acute Otitis Media (AOM):** Across the <1 year age band, males had higher odds of presenting with AOM – the probability was 2.7%.
- **Sinusitis:** Across the 5-14 year age band, males had higher odds of presenting with sinusitis – their combined probability, however, was not particularly large.
- **Intestinal Infectious Disease (IID):** Across the 1-14 year age band, males had lower odds of presenting with IID – the combined probability for these patients was nonetheless over 0.3%.
- **Influenza-like Illness (ILI):** There were no statistically significant results for young males related to ILI.

8. Episode typing – key part of data quality

Recording episode type is the only way that we can differentiate incident (first and new) from prevalent cases (ongoing care/reviews). RCGP RSC practices get constant feedback and reminders about the importance of recording whether a clinical consultation is the first time a patient presents the condition or whether it is a follow up appointment. Different conditions will have varying ratios of incident (first or new) to prevalent cases (reviews). We expect:

- Only a small proportion of people with influenza or influenza-like illness will be seen more than once by their GP. We therefore expect there to be very few follow-ups.
- A higher proportion of people with acute bronchitis and/or bronchiolitis will be followed up.
- Most people with asthma (a long term condition) will be seen for follow-up/review. In asthma we anticipate there will be many more reviews than new cases.

The chart below shows the mean rate of First and New episodes against all episodes for the seven key conditions for which we provide monthly feedback to practices:

- Bronchitis
- Allergic Rhinitis
- Asthma
- Infectious Intestinal Diseases
- Influenza-like illness
- Urinary Tract Infections

Episode typing by disease

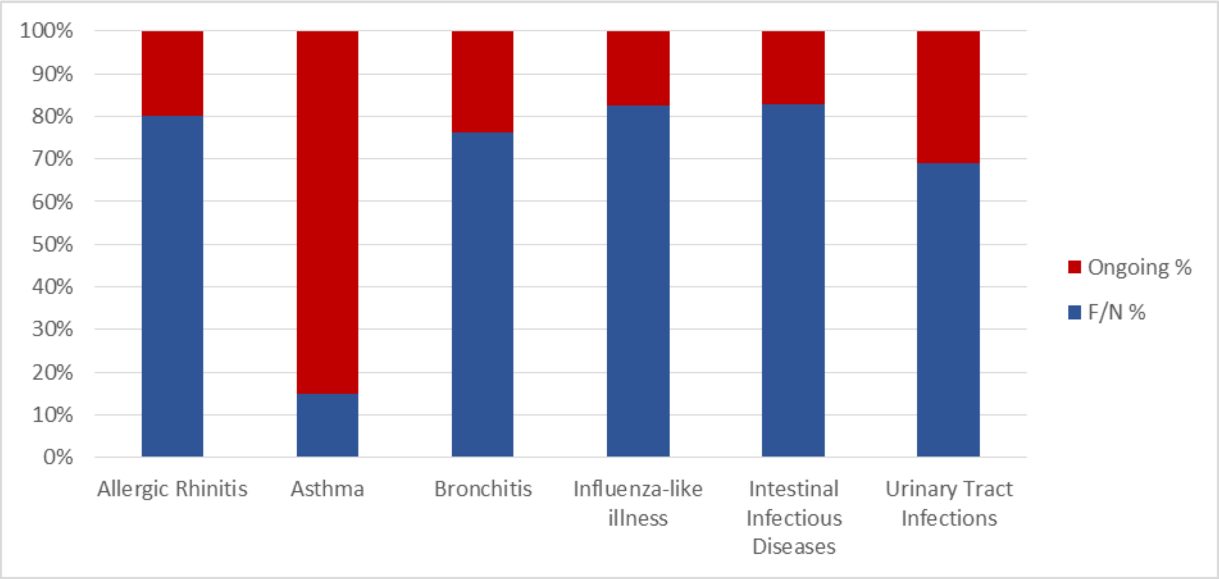

9. Current Projects

The RSC has been involved in the following projects during 2015/16.

European Projects

- **I-MOVE+:** RCGP RSC works in collaboration with leading European Public Health Institutes and Universities. It aims at measuring and comparing the effectiveness (VE) and impact (VI) of influenza and Pneumococcal vaccines and vaccination strategies a in the elderly population in Europe. The goal is to develop a sustainable platform of primary care practices, hospitals and laboratory networks that share validated methods to evaluate post marketing vaccine performances.
- **ADVANCE:** The RSC is involved in The Accelerated Development of Vaccine benefit-risk Collaboration in Europe (ADVANCE) project funded by the Innovative Medicines Initiative (IMI). The project is focused on more rapidly generating best evidence on vaccine benefits and risks. It aims to establish a blueprint for a validated and tested best practice framework that could rapidly provide robust data to support accelerated decision making.

Other Projects

- **The live-attenuated influenza vaccination (LAIV) project:** The RSC is working with Public Health England and the University of Surrey on the pilot of a universal childhood (LAIV) programme. The UK initiated a universal childhood immunisation programme with a newly licensed intransally-administered trivalent LAIV in the 2013/14 influenza season.
- **Integrate Project (Enhanced Gastroenteritis Surveillance):** This is a Wellcome-Department of Health funded research study led by Prof Sarah O'Brien at Liverpool University. The objective is to create enhanced surveillance systems for gastrointestinal illness. The surveillance system will include veterinary surveillance and much more advanced and rapid identification of organisms responsible for vomiting and diarrhoea. The recruitment process has been finalised with a network of 60 practices providing regular stool samples for pathogen surveillance.
- **Flu virology scheme:** Approximately 90 of the RSC practices are included in the flu virology specimen scheme. These practices provide nasal and throat specimens during the flu season (between the International Standards Organisation (ISO) week 40 and ISO week 20) each year from patients clinically suspected of having flu. This helps to establish which strain of the flu is dominant in the current season and is likely to be dominant the following flu season and how the flu vaccine can be improved in order to target these specific strains.
- **Diabetes Real World Evidence Centre:** The RSC is providing data, subject to ethical approval for studies looking at adherence to different types of diabetes medicines and the thresholds at which different clinicians implement injectable therapy in Type 2 diabetes.

10. RCGP Publications 2015/16

Correa A, Hinton W, McGovern A, van Vlymen J, Yonova I, Jones S, de Lusignan S. Royal College of General Practitioners Research and Surveillance Centre (RCGP RSC) sentinel network: a cohort profile. BMJ Open. 2016 Apr 20;6(4):e011092. doi: 10.1136/bmjopen-2016-011092. PubMed PMID: 27098827; PubMed Central PMCID: PMC4838708.

Pebody R, Warburton F, Ellis J, Andrews N, Potts A, Cottrell S, Johnston J, Reynolds A, Gunson R, Thompson C, Galiano M, Robertson C, Mullett D, Gallagher N, Sinnathamby M, Yonova I, Moore C, McMenamin J, de Lusignan S, Zambon M. Effectiveness of seasonal influenza vaccine in preventing laboratory-confirmed influenza in primary care in the United Kingdom: 2015/16 mid-season results. Euro Surveill. 2016 Mar 31;21(13). doi: 10.2807/1560-7917.ES.2016.21.13.30179. PubMed PMID: 27074651.

Botting J, Correa A, Duffy J, Jones S, de Lusignan S. Safety of community-based minor surgery performed by GPs: an audit in different settings. Br J Gen Pract. 2016 May;66(646):e323-8. doi: 10.3399/bjgp16X684397. Epub 2016 Mar 10. PubMed PMID: 26965026; PubMed Central PMCID: PMC4838444.

McGovern A, Tippu Z, Hinton W, Munro N, Whyte M, de Lusignan S. Systematic review of adherence rates by medication class in type 2 diabetes: a study protocol. BMJ Open. 2016 Feb 29;6(2):e010469. doi: 10.1136/bmjopen-2015-010469. PubMed PMID: 26928029; PubMed Central PMCID: PMC4780063.

McGovern AP, Hine J, de Lusignan S. Infection risk in elderly people with reduced glycaemic control. Lancet Diabetes Endocrinol. 2016 Apr;4(4):303-4. doi: 10.1016/S2213-8587(16)00043-7. Epub 2016 Feb 16. PubMed PMID: 26895714.

Mold F, de Lusignan S. Patients' Online Access to Their Primary Care Electronic Health Records and Linked Online Services: Implications for Research and Practice. J Pers Med. 2015 Dec 4;5(4):452-69. doi: 10.3390/jpm5040452. PubMed PMID: 26690225; PubMed Central PMCID: PMC4695865.

de Lusignan S, Crawford L, Munro N. Creating and using real-world evidence to answer questions about clinical effectiveness. J Innov Health Inform. 2015 Nov 4;22(3):368-73. doi: 10.14236/jhi.v22i3.177. PubMed PMID: 26577427.

11. Participating practices

This list includes all practices who were members of the RSC network at any point during the period covered by this report. For technical reasons, we were unable to extract data from all of the practices below for use in this report; therefore, the report is based on 155 of these 202 practices. However, we would like to thank all practices for their participation in the work of the RSC.

**Practice Name**	**Address**
Aberfeldy Practice	London
Adderlane Surgery	Northumberland
Adelaide Medical Centre	London
Albert Road And Britannia Village Surgery	London
Alcester Health Centre	Warwickshire
Alconbury & Brampton Surgeries	Cambridgeshire
Aldershot Health Centre	Hampshire
Ampthill Square Medical Centre	London
Ash Tree House Surgery	Lancashire
Aspartia Medical Group	Cumbria
Avisford Medical Group	West Sussex
Axbridge & Wedmore M.P.	Somerset
Aylestone Surgery (Sahdev)	Leicester
Backwell Medical Centre	Bristol
Bangor Street Health Centre	Blackburn
Barlby Surgery	London
Barlow Medical Centre	Manchester
Barnoldswick Medical Centre	Lancashire
Barrington Medical Centre	Cheshire
Baslow Health Centre	Derbyshire
Beacon View Med Ctr, Dr Gj Penrice	Tyne & Wear
Bishopgate Medical Centre	County Durham
Block Lane Surgery	Oldham
Bloomsbury Health Centre	Birmingham
Bloxwich Medical Practice	West Midlands
Bodriggy Health Centre	Cornwall
Bottisham Medical Practice	Cambridgeshire
Bretton Medical Practice	Cambridgeshire
Broadway Surgery	Preston
Brownlow Health (Brownlow Group Practice)	Liverpool
Burn Brae Medical Group	Northumberland
Cassidy Medical Centre	London
Castle Street Medical Centre	Derbyshire
Cator Medical Centre	Kent
Cedar Brook Practice	Middlesex

Cheddar Medical Centre	Somerset
Cherry Hinton Surgery	Cambridge
Church Street Practice	Oxfordshire
Church Street Surgery	Hertfordshire
City Road Medical Centre	London
Clarendon Lodge	Warwickshire
Clevedon Riverside Group	North Somerset
Cleveleys Group Practice	Lancashire
Collingham Medical Centre	Nottinghamshire
Comberton Surgery	Cambridgeshire
Cotterils Lane Surgery	Birmingham
Creffield Medical Centre	Essex
Debenham Group Practice	Suffolk
Devon Square Surgery	Devon
Dr Ij Moodies Practice	Blackburn
Dr Maclennan & Partners	Oxfordshire
Dr Scriven, Lee, Hopkins & Sissons	North Staffordshire
Dr Slade & Partners	Somerset
Dronfield Medical Centre	Derbyshire
Dr'S Mackinnon Chande & Chappell	Bury
East Park Medical Centre	West Midlands
Eastham Group Practice	Wirral
Eaton Socon Health Centre	Cambridgeshire
Ecclesfield Group Practice	Sheffield
Edith Cavell Surgery	London
Ellenbrook Medical Centre	Manchester
Elm House Surgery	Kent
Farnborough Bank House Surgery	Kent
Fieldway Medical Centre	Surrey
Fosse Medical Centre	Leicester
Framlingham Medical Practice	Suffolk
Frankley Health Centre	Birmingham
Garswood Surgery	Wigan
Glenpark Medical Centre	Tyne & Wear
Gosforth Valley Med Practice	Derbyshire
Gosport Medical Centre	Hampshire
Greens Norton & Weedon Practice	Northamptonshire
Greenway Community Practice	Bristol
Grove Medical Group	Tyne & Wear
Guardian Street Medical Centre	Cheshire
Guildowns Group Practice	Guildford
Haslemere Health Centre	Surrey
Haydock Medical Centre	St Helens
Headley Drive Surgery	Croydon

Heatherside Surgery	Camberley
Highlands Surgery	Essex
Husbands Bosworth Surgery	Leicestershire
Immeary Street Surgery	South Shields
James Street Group Practice	Cumbria
Killick Street Health Centre	London
Kiltearn Medical Centre	Cheshire
Kings Cross Road Surgery	London
Kings Road Medical Centre	London
Knockin Medical Centre	Shropshire
Lache Health Centre	Cheshire
Laurel Bank Surgery	Leeds
Laurel Bank Surgery	Cheshire
Lawrence House Surgery	London
Lordswood House Practice	Birmingham
Lowdham Medical Centre	Nottinghamshire
Lowfield Medical Centre	Dartford
Market Harbororugh Med. Centre	Leicestershire
Market Surgery	Norwich
Maywood Surgery	West Sussex
Medwyn Surgery	Dorking
Mendip Vale Medical Practice	Bristol
Merepark Medical Park	South Cheshire
Mill Hill Surgery	London
Millway Medical Practice	London
Minchinhampton Surgery	Gloucestershire
Mitchinson Road Surgery	London
Monkspath Surgery	West Midlands
Mount Chambers Surgery	Essex
Mountwood Surgery	Middlesex
Much Wenlock Practice	Shropshire
Myhealth	York
Nettleham Medical Centre	Lincoln
New Hayesbank Surgery	Kent
New Inn Surgery	Guildford
New Road Surgery Bromsgrove	Worcestershire
Newton Place Surgery	Kent
Nightingale Valley Pract	Bristol
Normanby Medical Centre	Cleveland
North Road West Medical Centre	Plymouth
Northfield Health Centre	Birmingham
Oak Lodge Medical Centre	Middlesex
Oak Vale Medical Centre	Liverpool
Oaklands Health Centre	Kent

Oakwood Surgery	Leeds
Palacci & Partners (Castelnau)	London
Papworth Surgery	Cambridgeshire
Parkside Group Practice	Surrey
Parkway Health Centre	Surrey
Pendle View Medical Centre	East Lancashire
Petersfield Surgery	Essex
Phoenix Surgery	Gloucestershire
Pontesbury Medical Practice	Shropshire
Portishead Medical Group	Bristol
Portslade Health Centre	East Sussex
Priory Medical Centre	Liverpool
Prospect Medical Practice	Norwich
Queens Road Surgery	London
Regent House Surgery	Lancashire
Richmond Hill Practice	East Lancashire
Ridgacre House Surgery	Birmingham
Riverside Surgery	North Lincolnshire
Saddleworth Medical Practice	Oldham
Sandy Lane Surgery	Lancashire
Sedbergh Health Centre (Lumb)	Cumbria
Shebbear Surgery	Devon
Silverlock Medical Centre	London
South Chadderton Health Centre	Oldham
St Fillans Medical Centre	Lancashire
St Gabriels Medical Centre	Manchester
St James Health Centre	Liverpool
St Leonard'S Practice	Devon
St Paul'S Medical Centre	Blackpool
Station House Surgery	Cumbria
Stoneleigh Surgery	Cumbria
Streatham High Practice	London
Streatham Place Surgery	London
Summertown Health Centre	Oxfordshire
Swan Lane Medical Centre	Greater Manchester
Thamesmead Health Centre	London
The Calverton Practice	Nottingham
The Carnewater Practice	Cornwall
The Church Lane Practice	London
The Eden Surgeries	West Essex
The Fairfields Practice	Nottingham
The Grange Medical Centre	Warwickshire
The Grange Practice	Thanet
The Hall Practice	Buckinghamshire

The Hambleden Clinic	London
The Hollies	Essex
The Lakeside Surgery	Northamptonshire
The Manor Health Centre- Dr Curran & Partners	London
The Manor Health Centre- Dr Sheila Santamaria	London
The Marshside Surgery	Sefton
The New Sheepmarket Surgery	Lincolnshire
The Schoolhouse Surgery	Cheshire
The Stokes Medical Centre	Bristol
The Uppingham Surgery	Leicestershire
The Valley Surgery	Nottinghamshire
The Whipton Surgery	Exeter
Thornton Road Surgery	Surrey
Trowbridge Surgery	London
Valley Park Surgery	Surrey
Vauxhall Primary Health Centre	Liverpool
Victoria Park Practice	Birkenhead
Villa Med Ctr	Wirral
Village Surgery	Rotherham
Warlingham Green Med Practice	Surrey
Wellington House Surgery	Buckinghamshire
Wellside Surgery	Cambridgeshire
West Common Lane Medical Ctr	Scunthorpe
West Timperley Medical Centre	Trafford
Westongrove Partners	Aylesbury
Whalley Medical Centre	Blackburn
White Rose Surgery	West Yorkshire
Whitechapel Health Centre	London
Windermere And Bowness Med Practice	Cumbria
Winterton Medical Practice	South Humberside
Wonford Green Surgery	Devon
Woodbridge Hill Surgery	Surrey
Worden Medical Centre	Lancashire
Yardley Green Medical Centre	Birmingham
Yaxley Group Practice	Peterborough

12. Contributors

Contributors

Simon de Lusignan

Ana Correa

Sameera Pathirannehelage

Rachel Byford

Ivelina Yonova

Filipa Ferreira

Imran Rafi

Contact details

RCGP Research & Surveillance Centre
University of Surrey
Clinical Medicine & Ageing
GUILDFOFD
GU2 7PX
Tel: +44 (0)1483 684802

Medical Director: Professor Simon de Lusignan
MedicalDirectorRSC@rcgp.org.uk

Practice Liaison Officer: Ivelina Yonova
i.yonova@surrey.ac.uk
Tel: +44 (0)1483 682758

13. Appendix A: Weekly disease incidence graphs

13. APPENDIX A : Weekly Disease Incidence Graphs

1. Water and Food Borne Disorders:

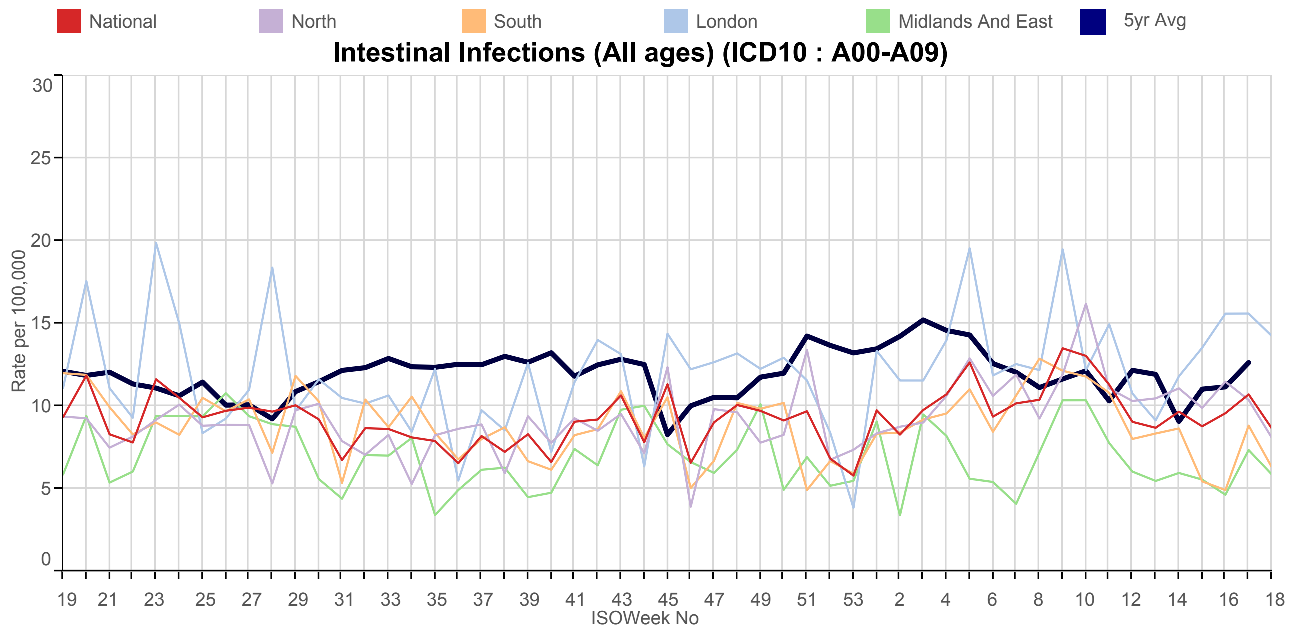

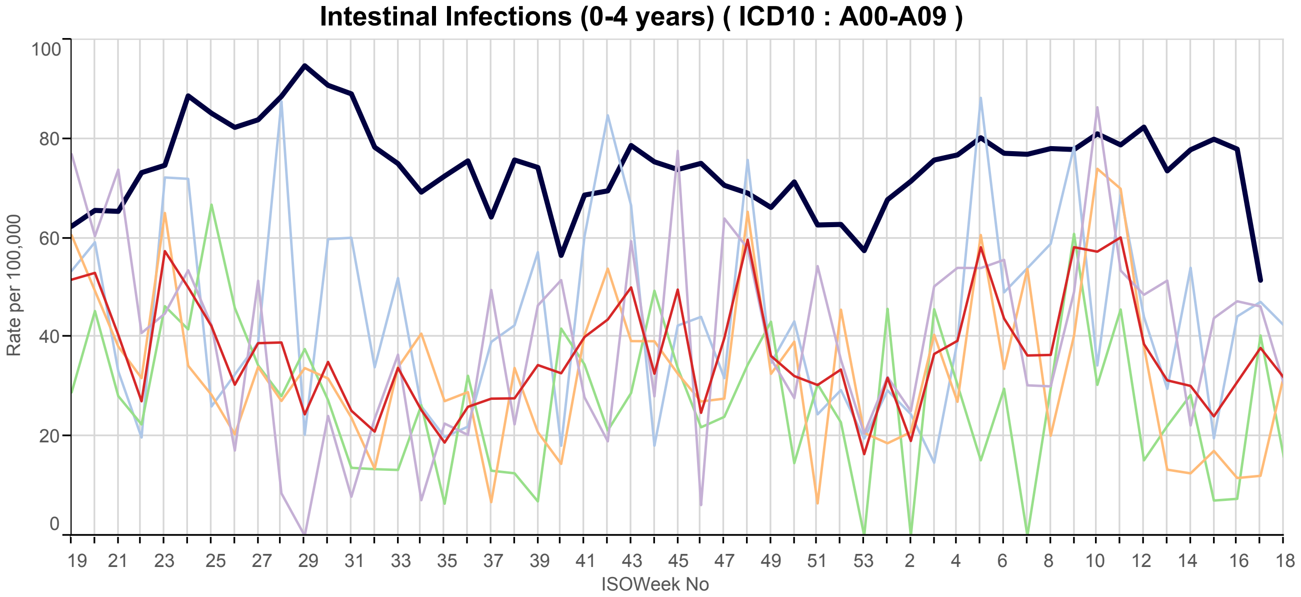

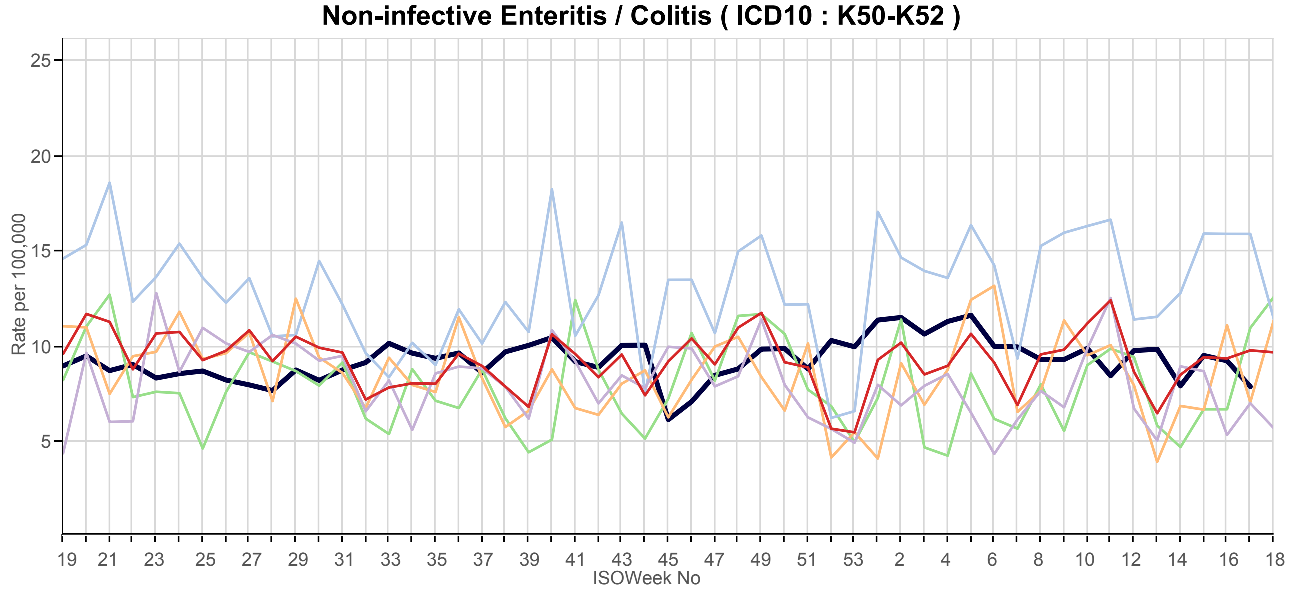

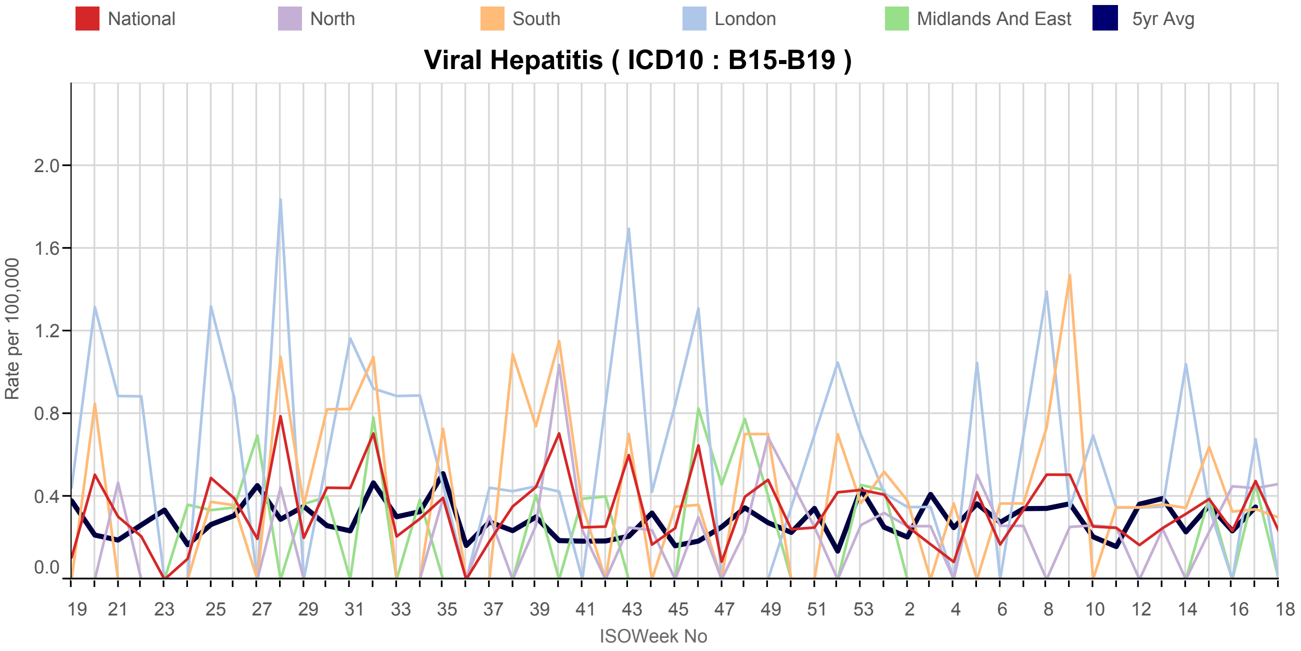
National
North
South
London
Midlands And East
5yr Avg
Viral Hepatitis (ICD10 : B15-B19)
Rate per 100,000
2.0
1.6
1.2
0.8
0.4
0.0
19
21
23
25
27
29
31
33
35
37
39
41
43
45
47
49
51
53
2
4
6
8
10
12
14
16
18
ISOWeek No

2. Environmentally Sensitive Disorders :

National North South London Midlands And East 5yr Avg

Asthma (ICD10 : J45 - J46)

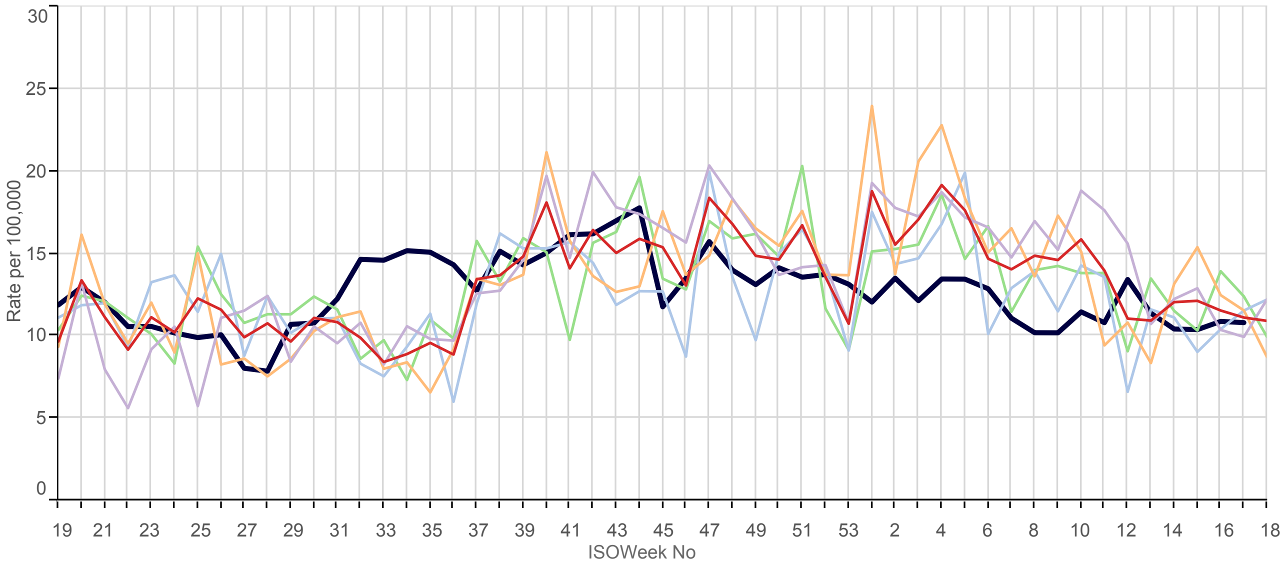

Conjunctivitis (ICD10 : H10 - H13)

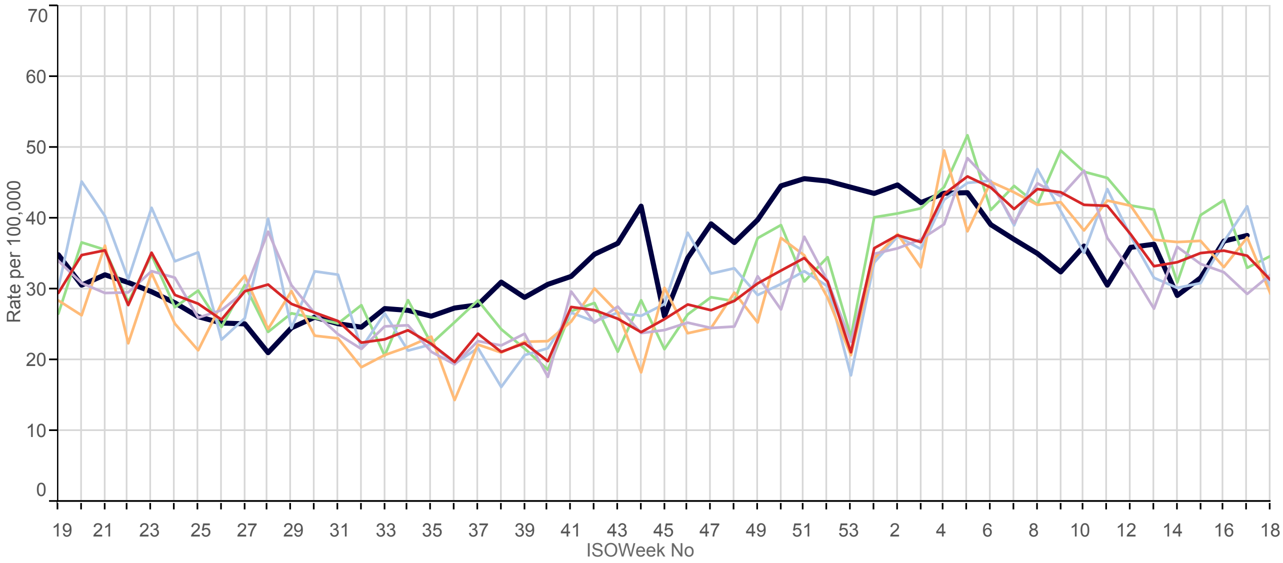

Hayfever/Allergic Rhinitis (ICD10: J30)

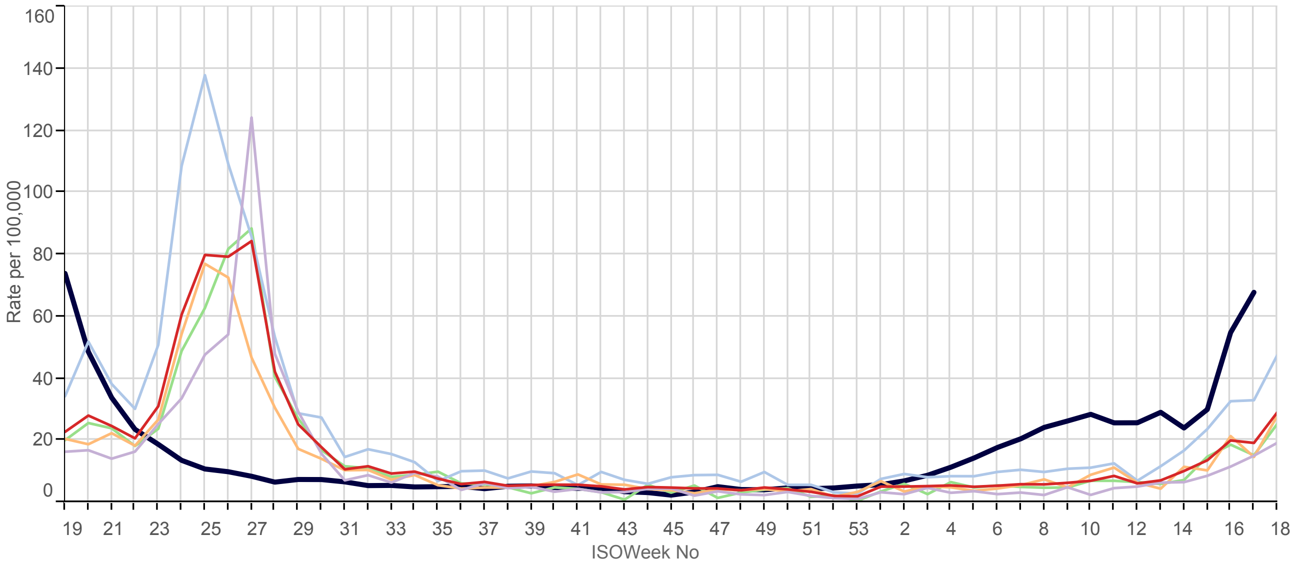

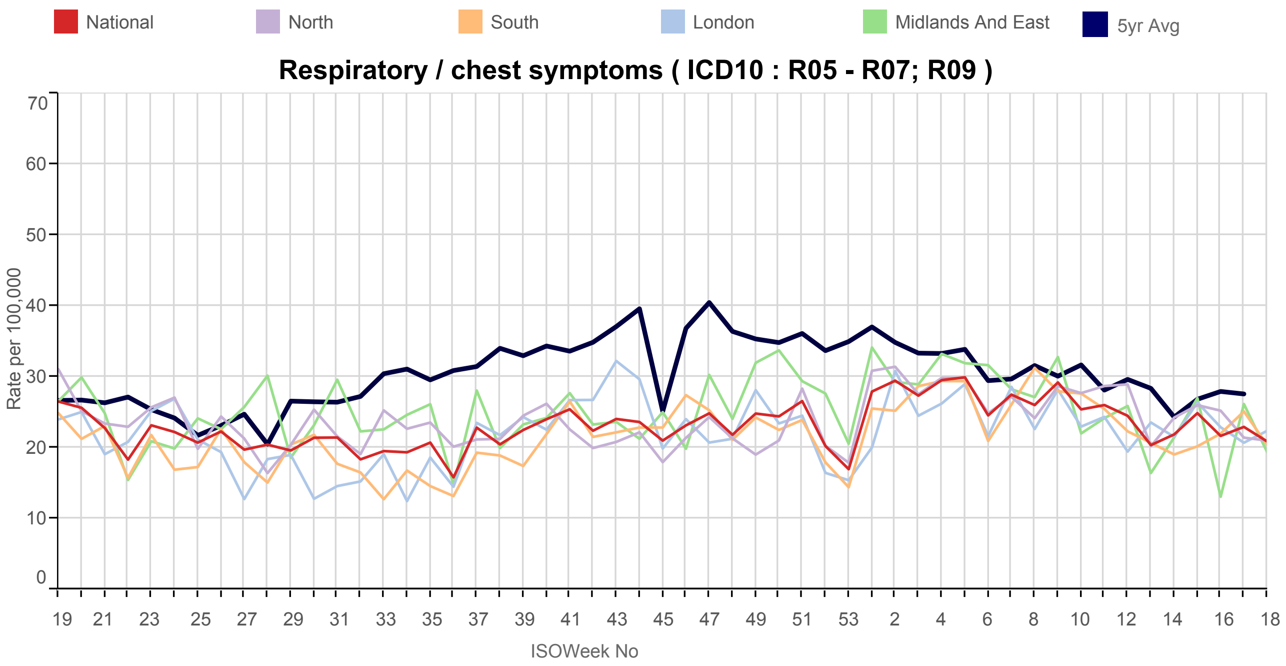
National
North
South
London
Midlands And East
5yr Avg
Respiratory / chest symptoms (ICD10 : R05 - R07; R09)
Rate per 100,000
70
60
50
40
30
20
10
0
19
21
23
25
27
29
31
33
35
37
39
41
43
45
47
49
51
53
2
4
6
8
10
12
14
16
18
ISOWeek No

3. Respiratory Infections :

National North South London Midlands And East 5yr Avg

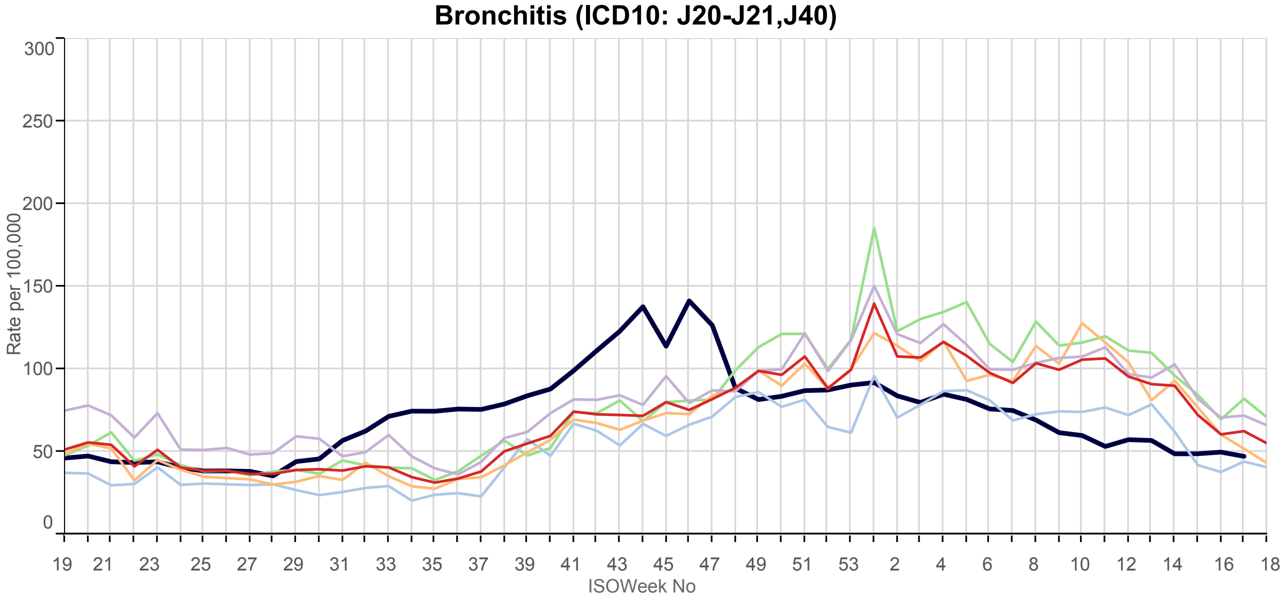

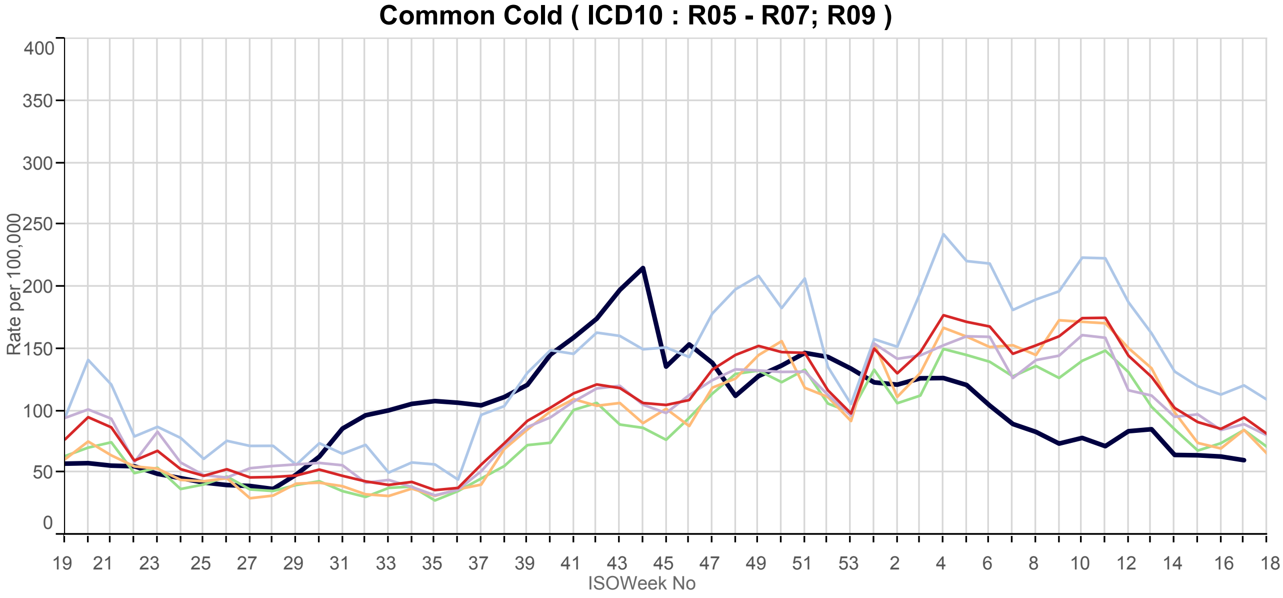

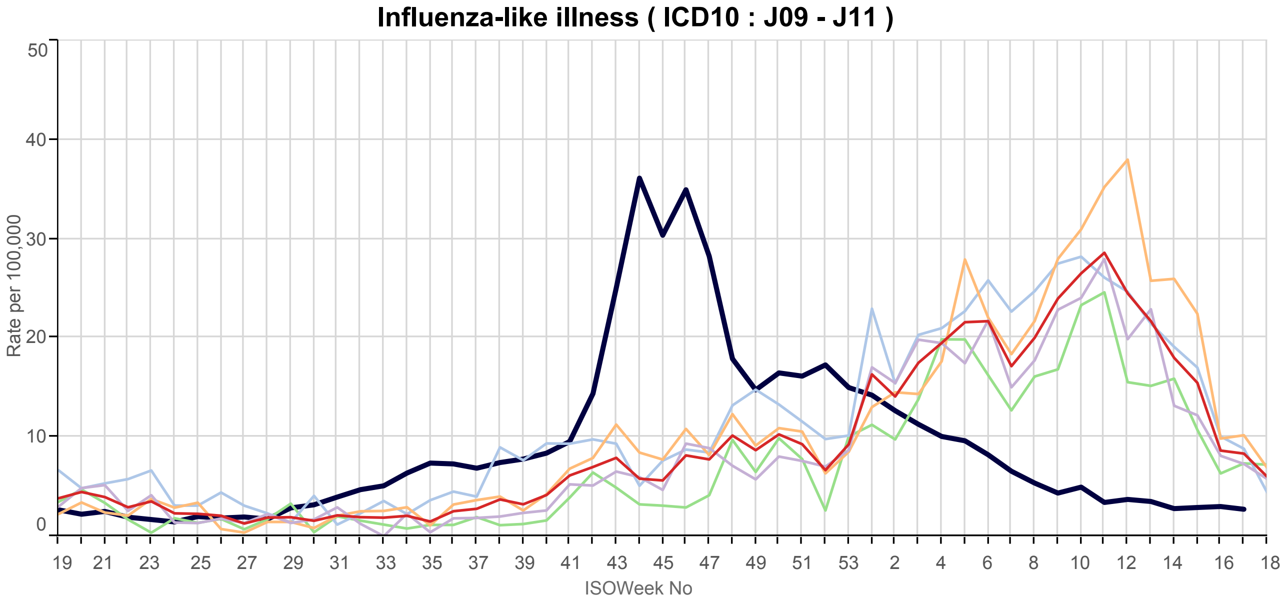

Laryngitis / Tracheitis (ICD10 : J04)

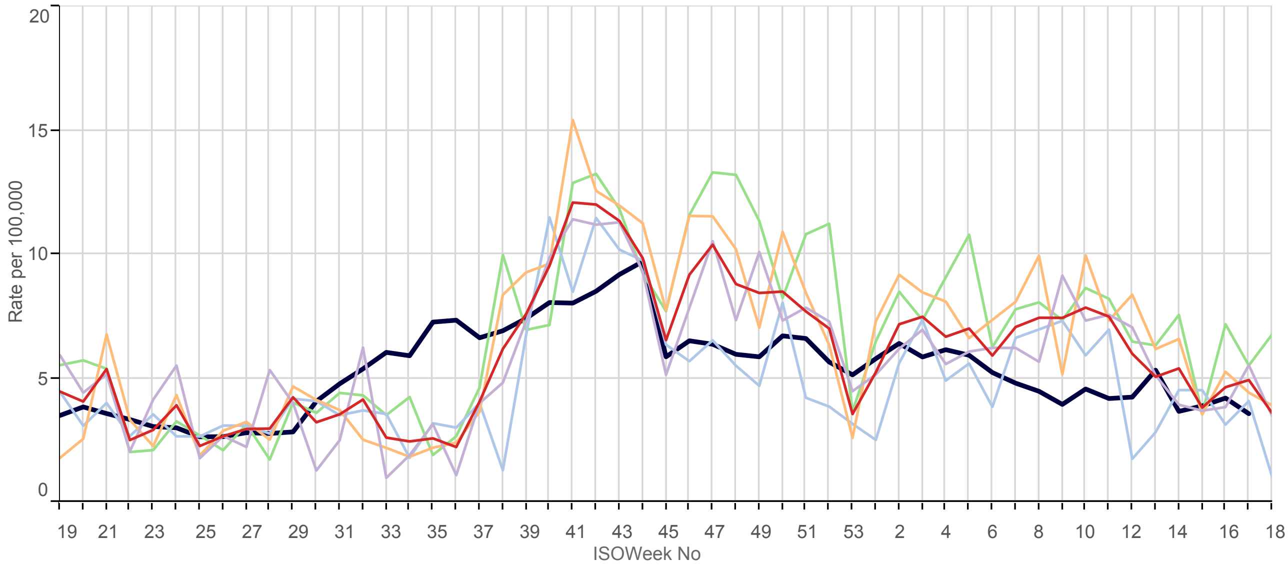

Pleurisy (ICD10 : R091)

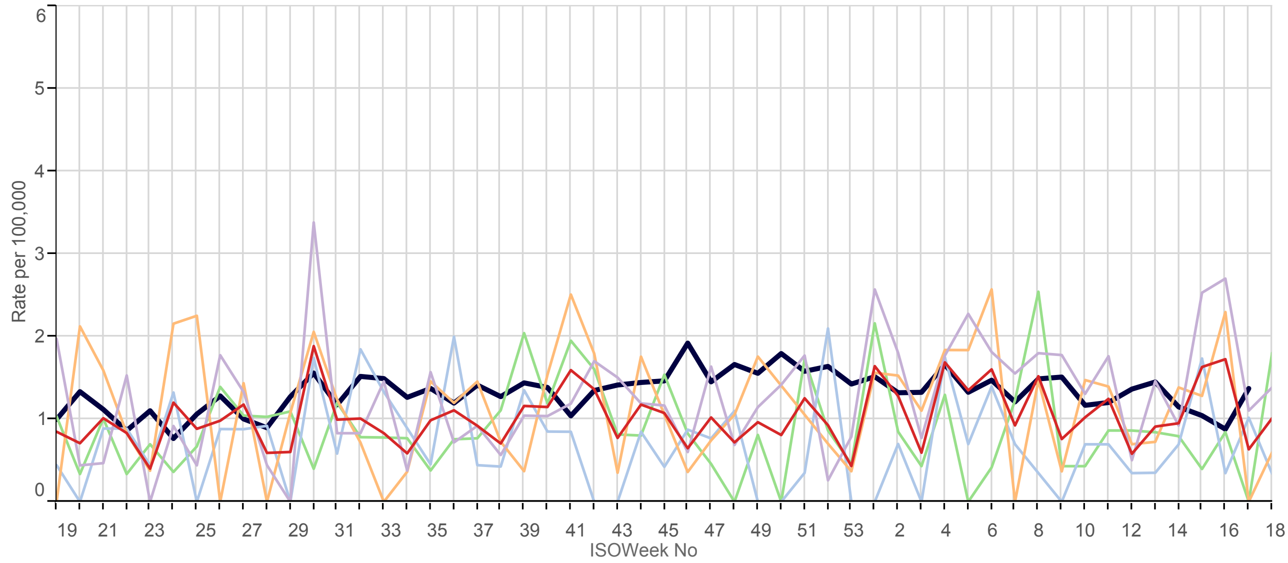

Pneumonia / Pneumonitis (ICD10 : J12 - J18)

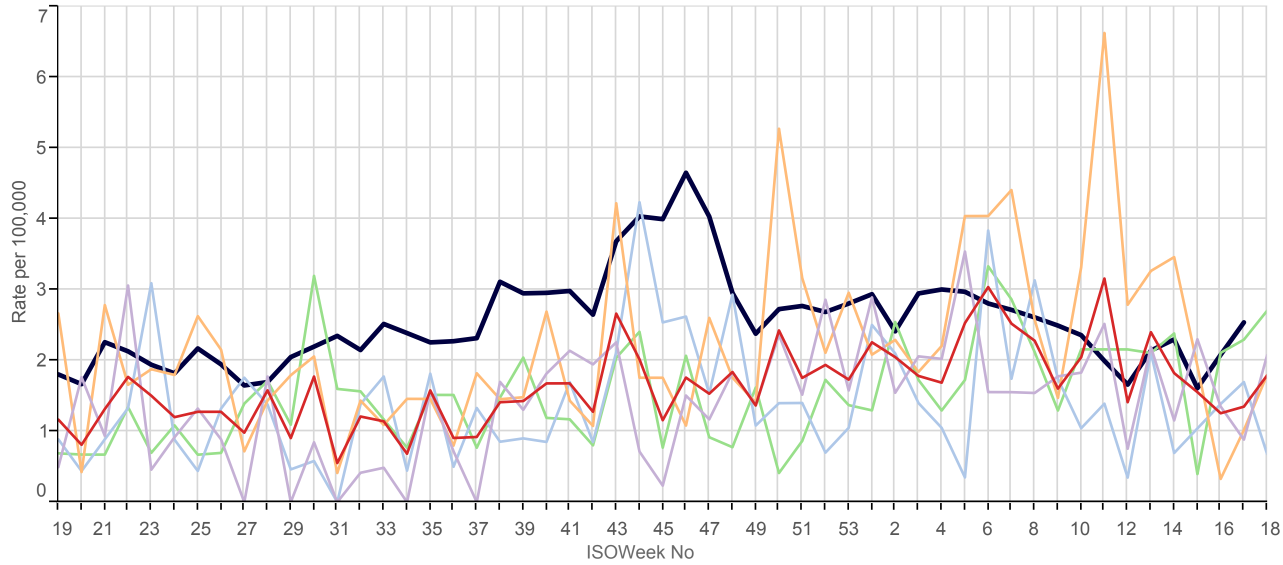

National North South London Midlands And East 5yr Avg

Respiratory disorders (ICD10 : J00-J99)

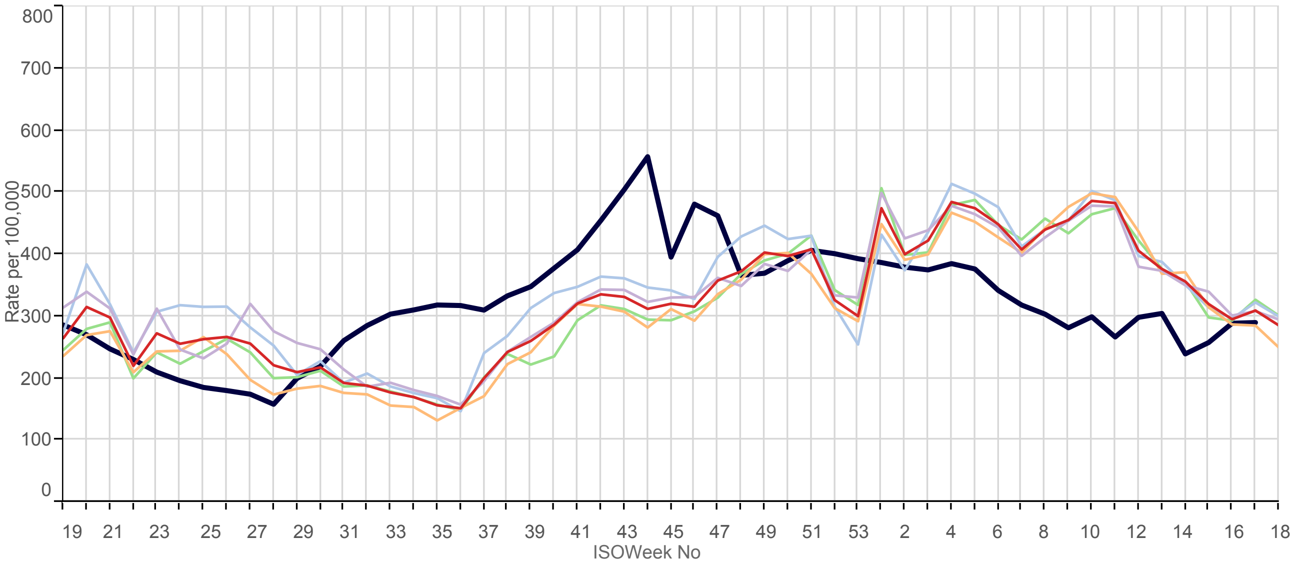

Acute Sinusitis (ICD10 : J01)

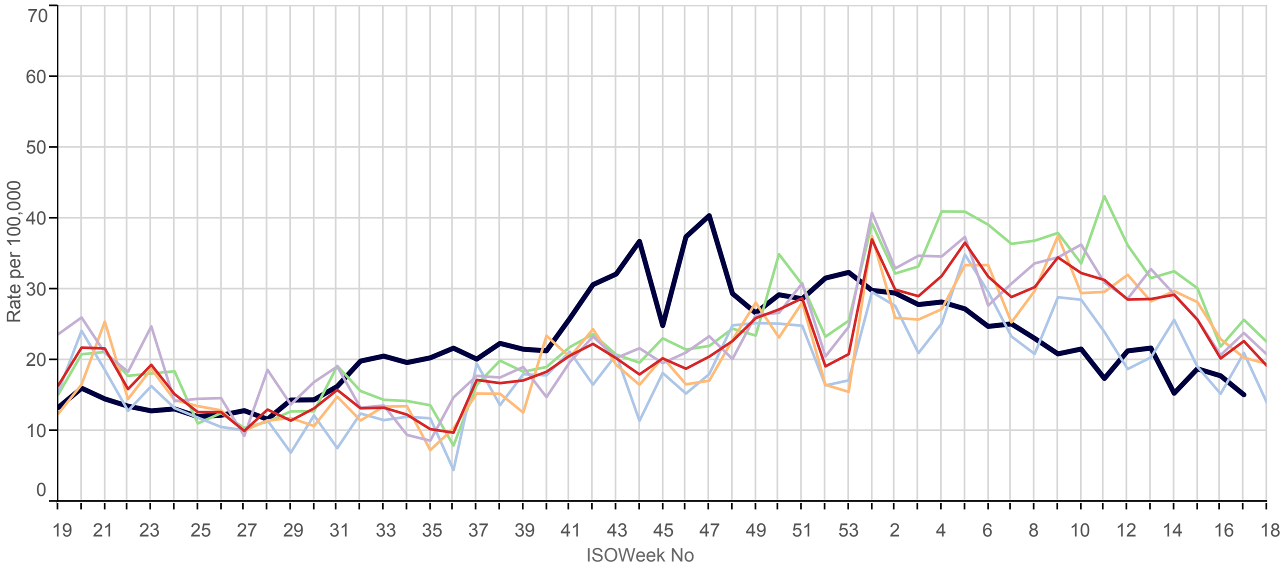

Strep throat / peritonsillar abscess (ICD10 : A38; J020; J36)

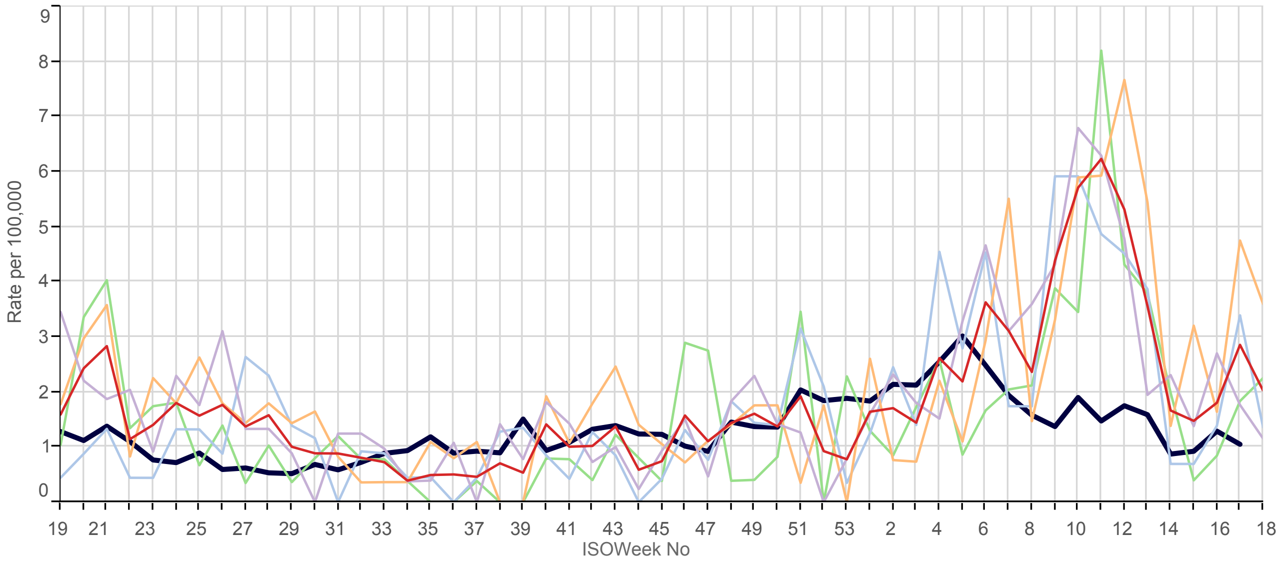

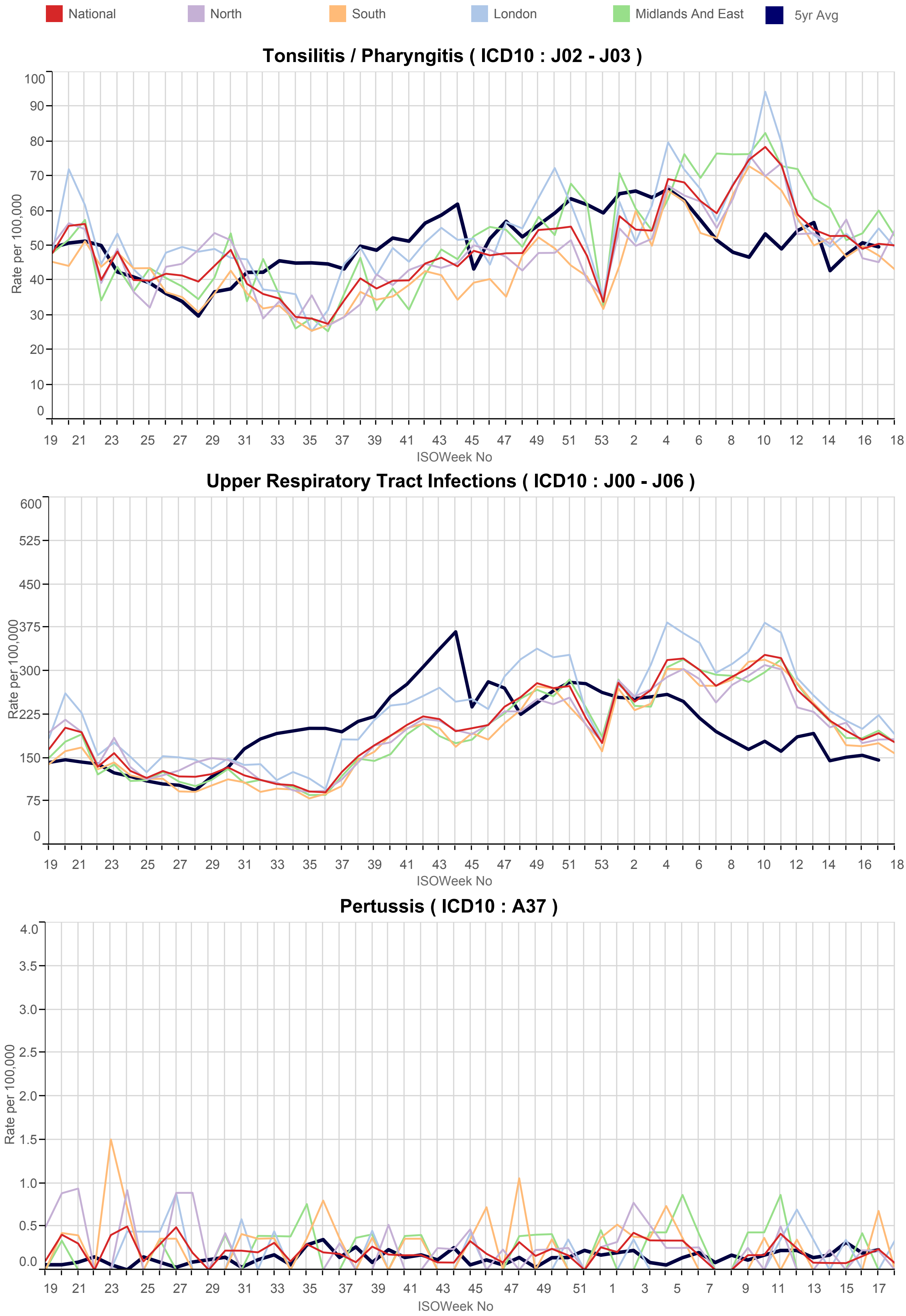
National
North
South
London
Midlands And East
5yr Avg
Tonsilitis / Pharyngitis (ICD10 : J02 - J03)
Rate per 100,000
ISOWeek No
Upper Respiratory Tract Infections (ICD10 : J00 - J06)
Rate per 100,000
ISOWeek No
Pertussis (ICD10 : A37)
Rate per 100,000
ISOWeek No

National North South London Midlands And East 5yr Avg

Infectious Mononucleosis (ICD10 : B27)

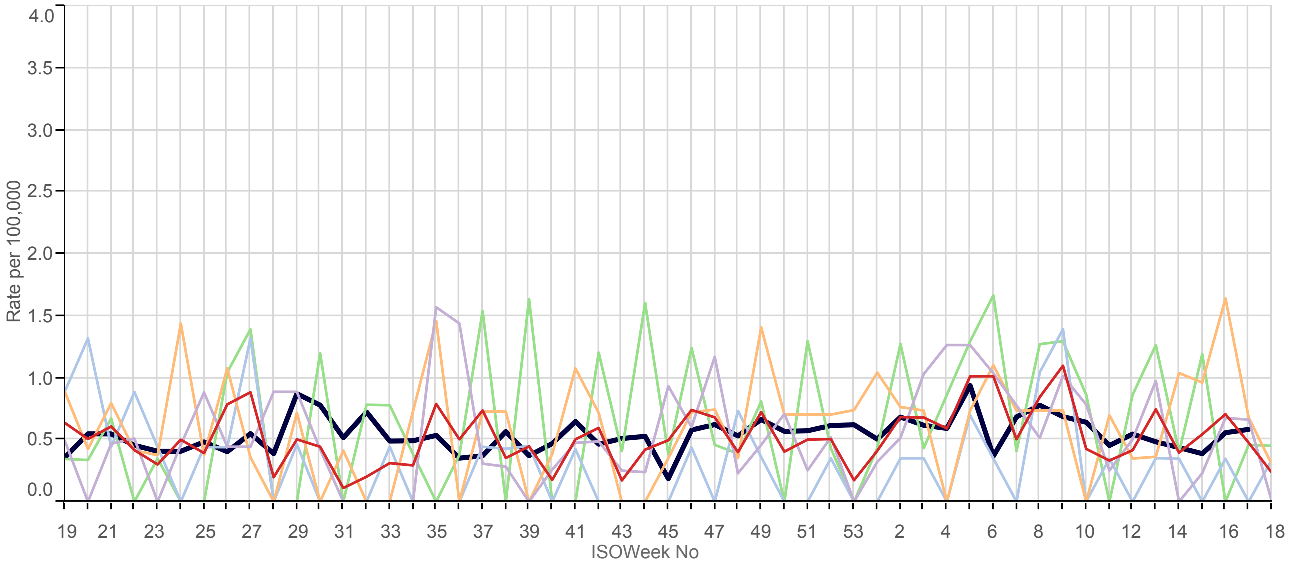

Lower Respiratory Tract Infections (ICD10 : J20-J22)

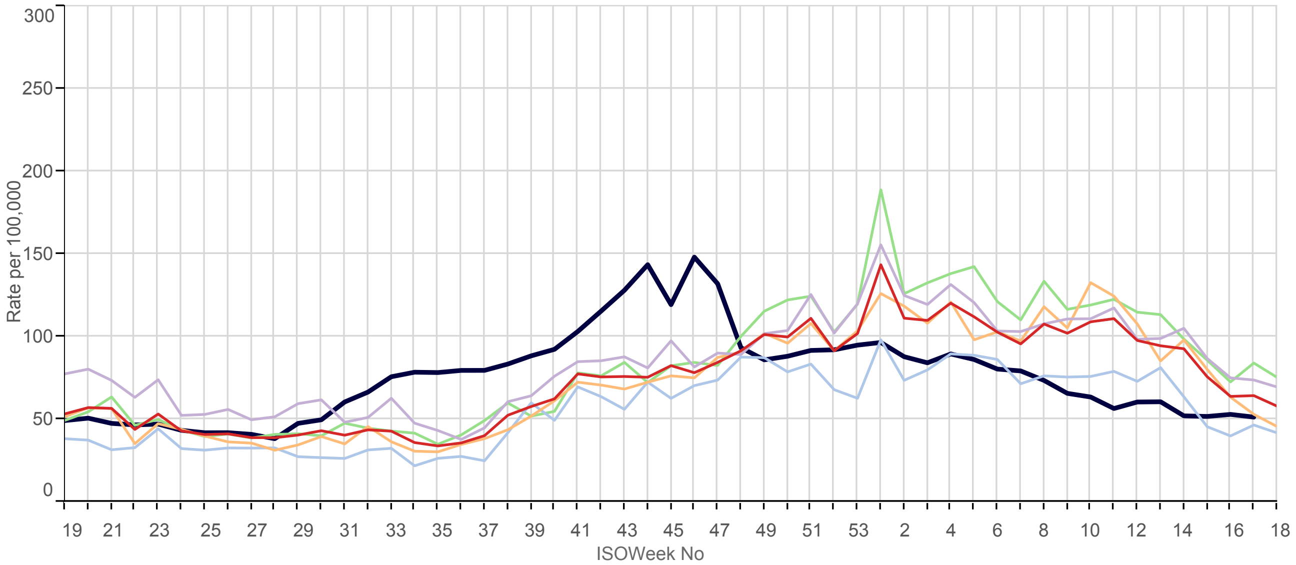

Acute Otitis Media (ICD10 : H650 - H651; H660; H669)

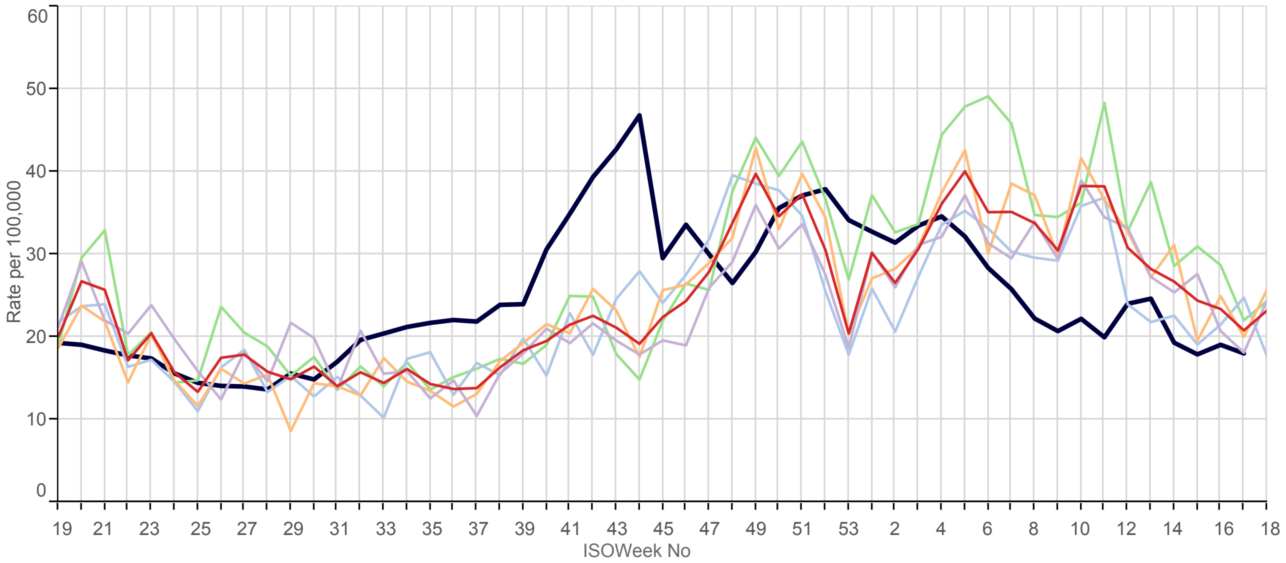

4. Vaccine Sensitive Disorders

National · North · South · London · Midlands And East · 5yr Avg

Measles (ICD10 : B05)

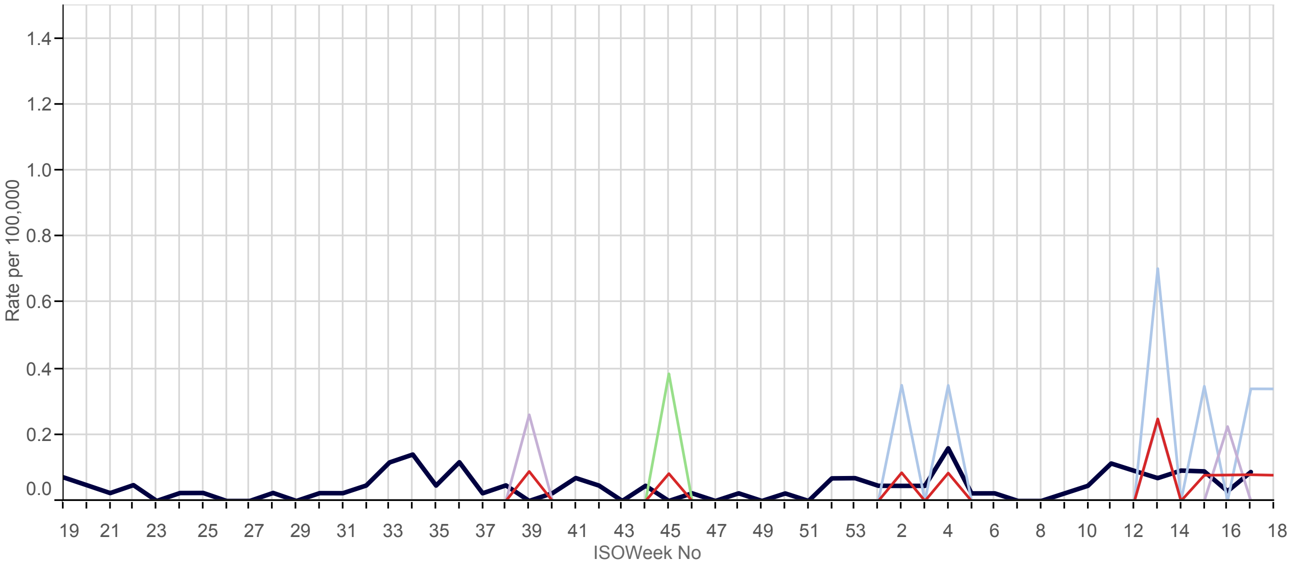

Mumps (ICD10 : B26)

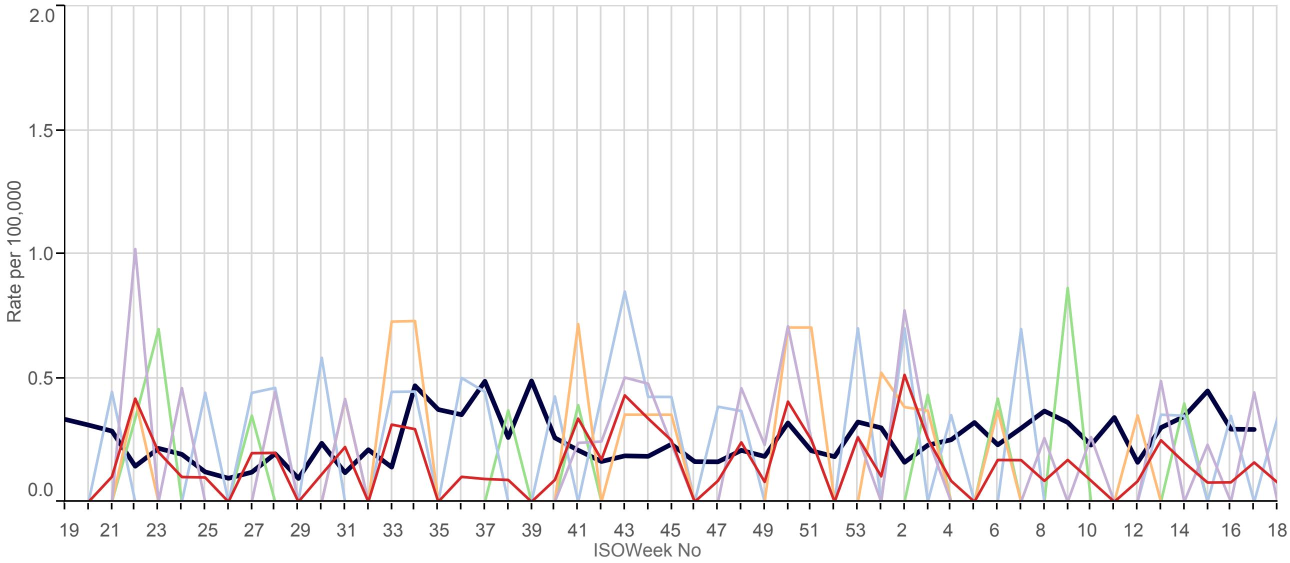

Rubella (ICD10 : B06)

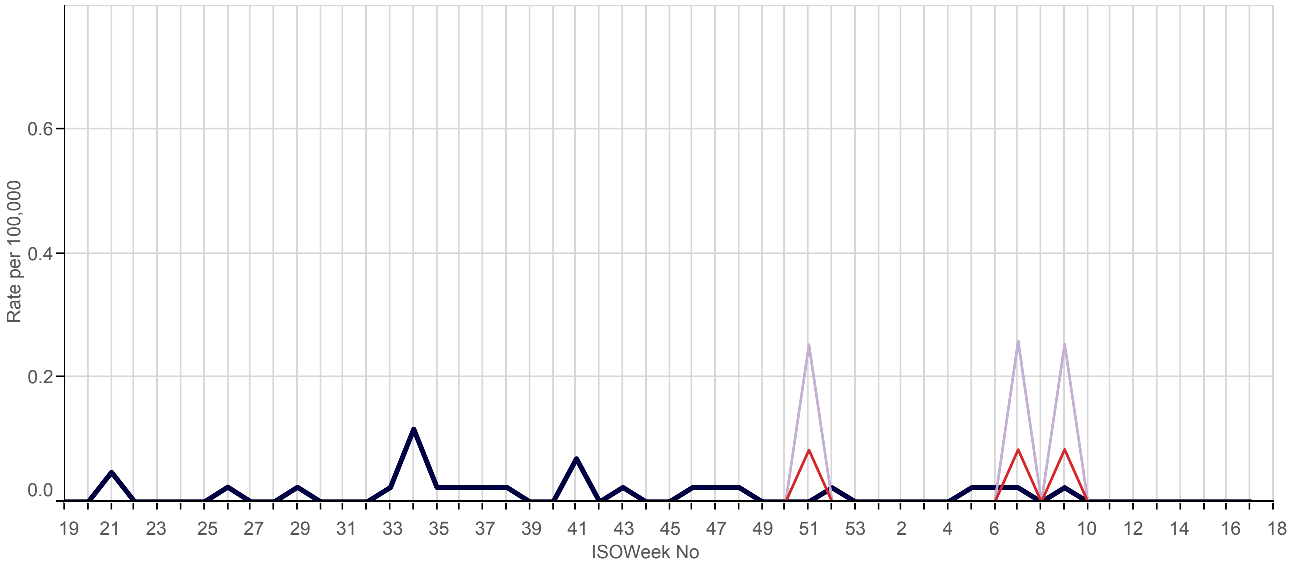

5. Skin Contagions

National North South London Midlands And East 5yr Avg

Bullous Dermatoses (ICD10 : L10 - L14)

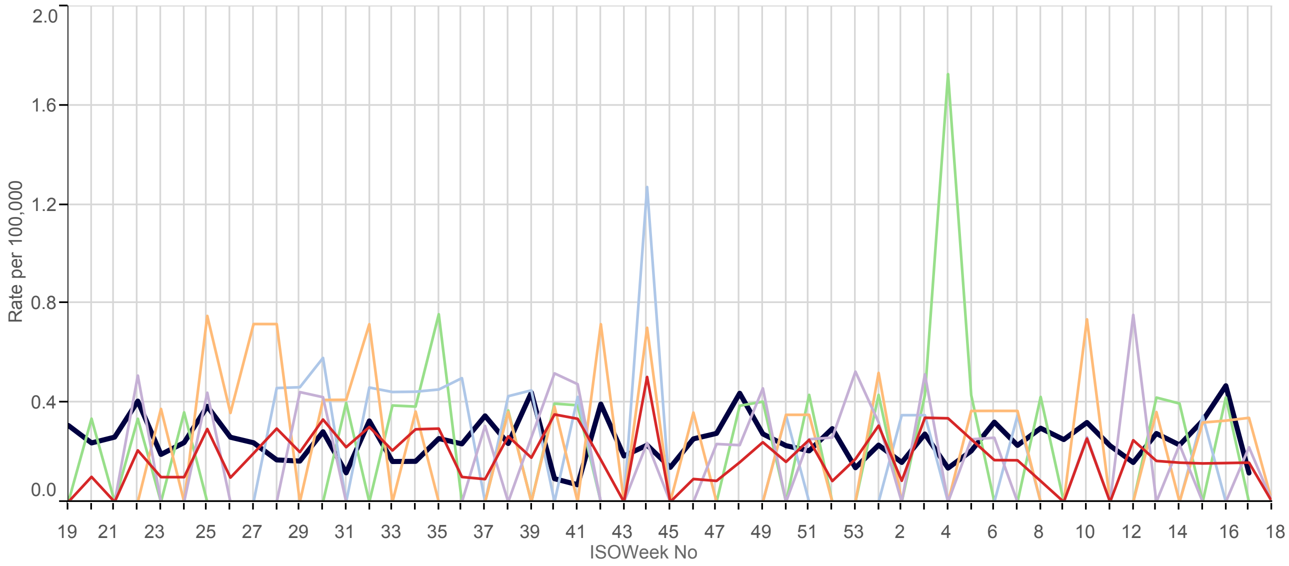

Chickenpox (ICD10 : B01)

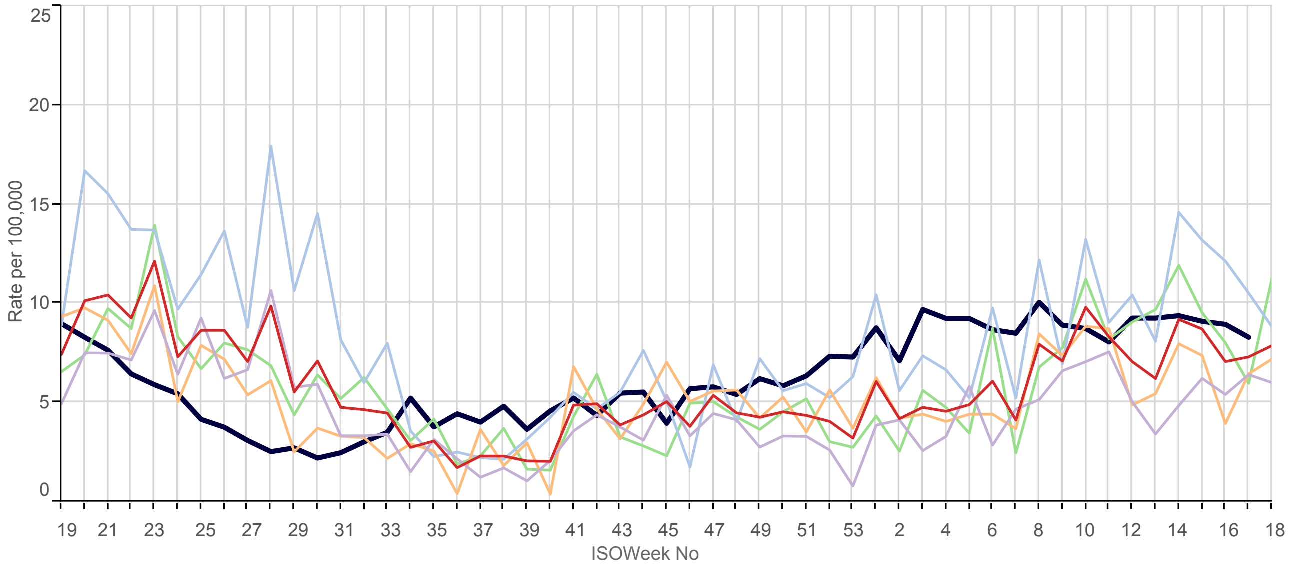

Herpes Simplex (ICD10 : B00)

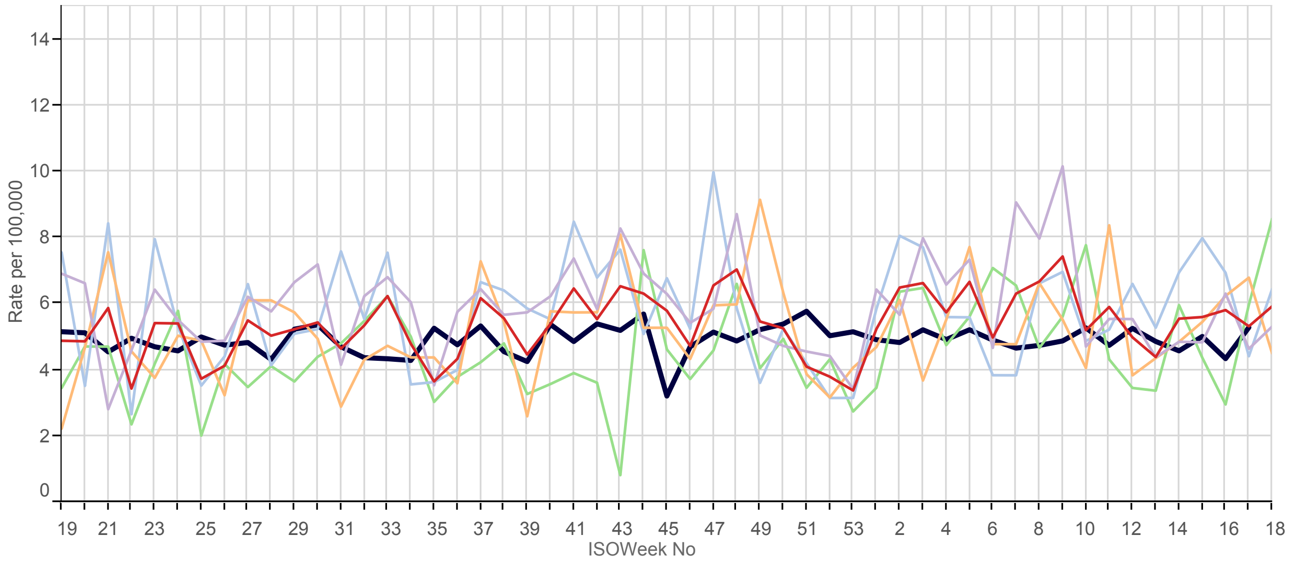

National North South London Midlands And East 5yr Avg

Herpes Zoster (ICD10 : B02)

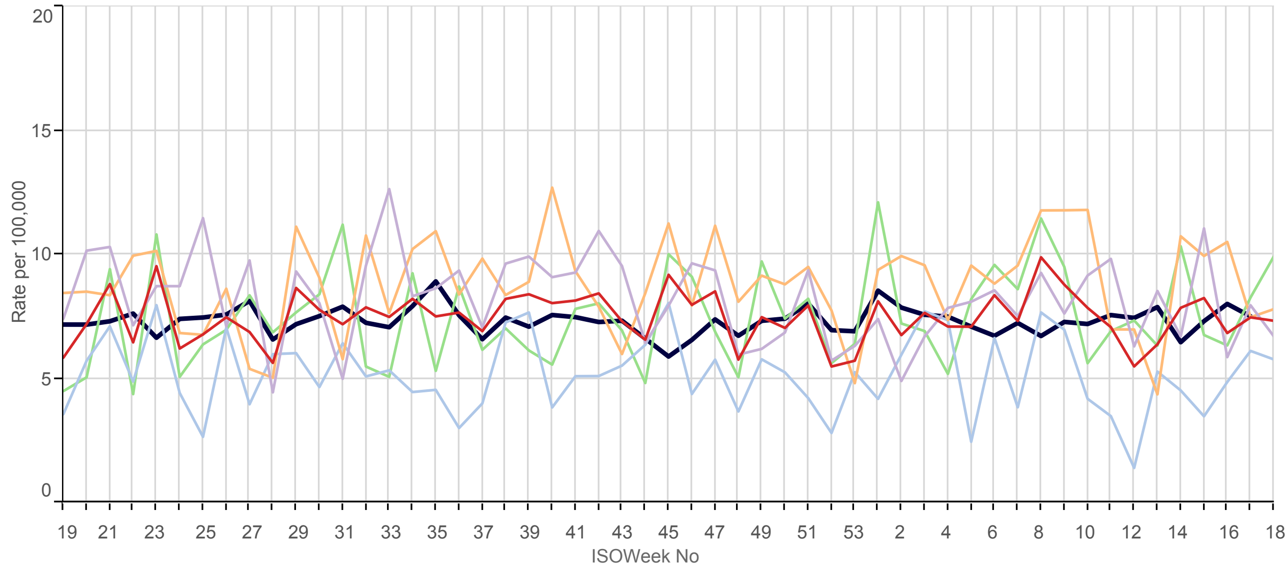

Skin / subcutaneous infections (ICD10 : L00 - L08)

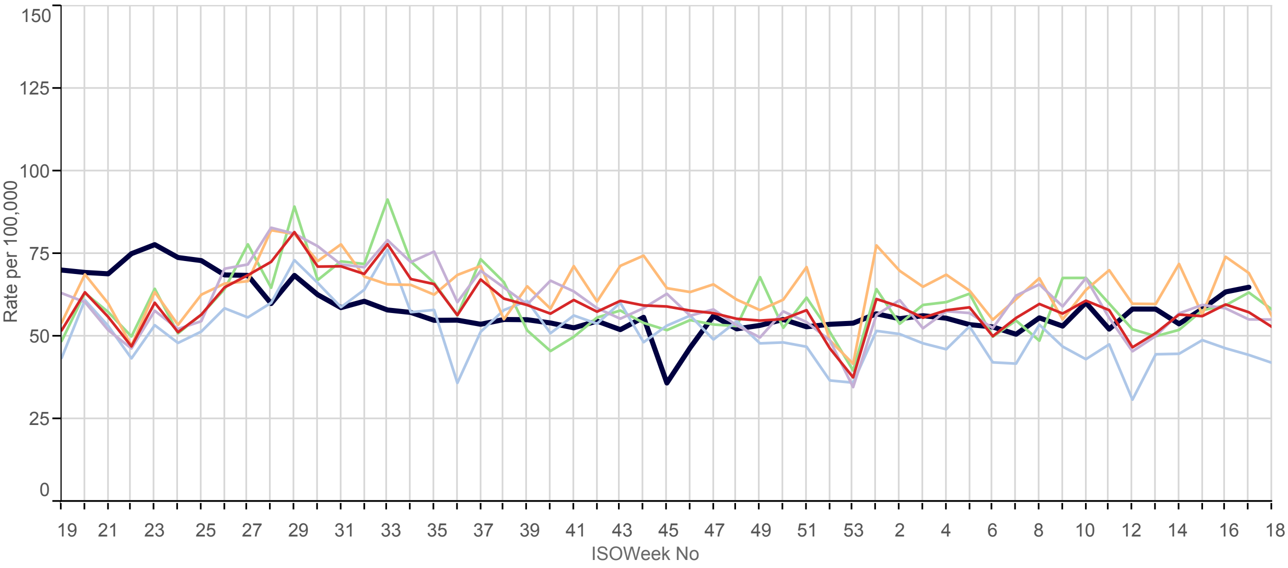

Scabies (ICD10 : B86)

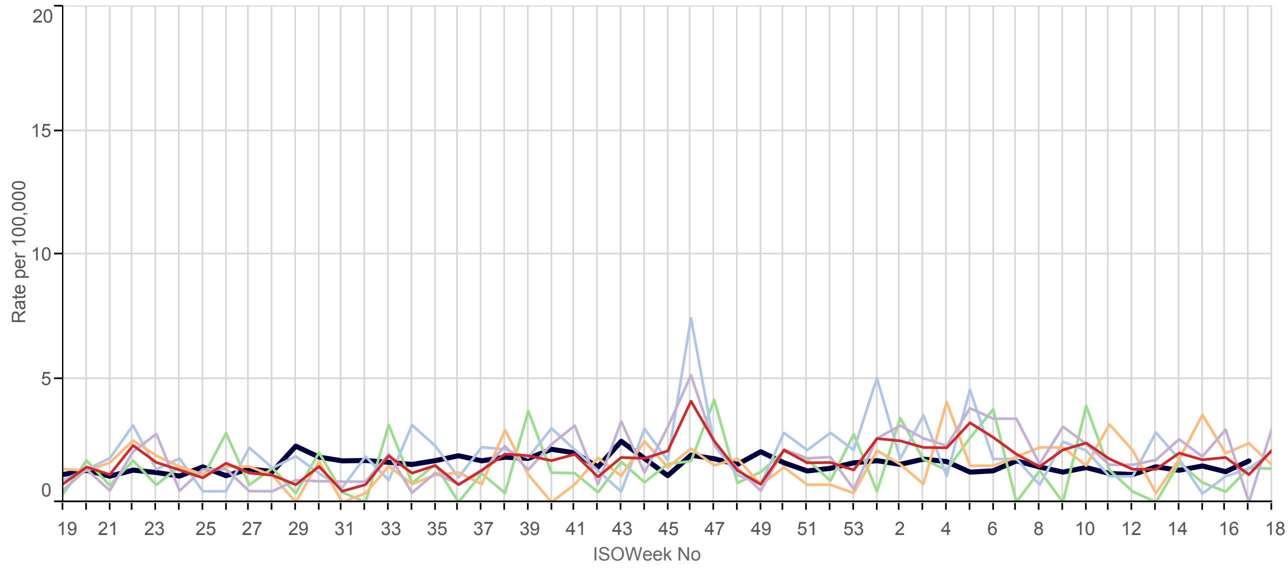

National North South London Midlands And East 5yr Avg

Skin symptoms (ICD10 : R20 - R23)

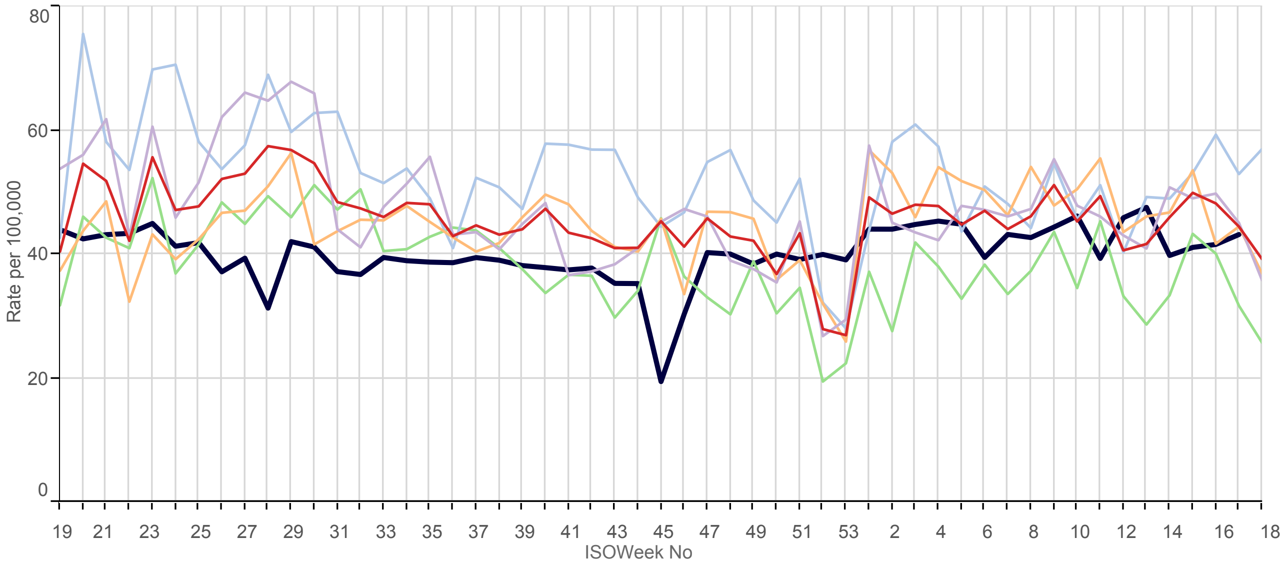

Impetigo (ICD10 - L01)

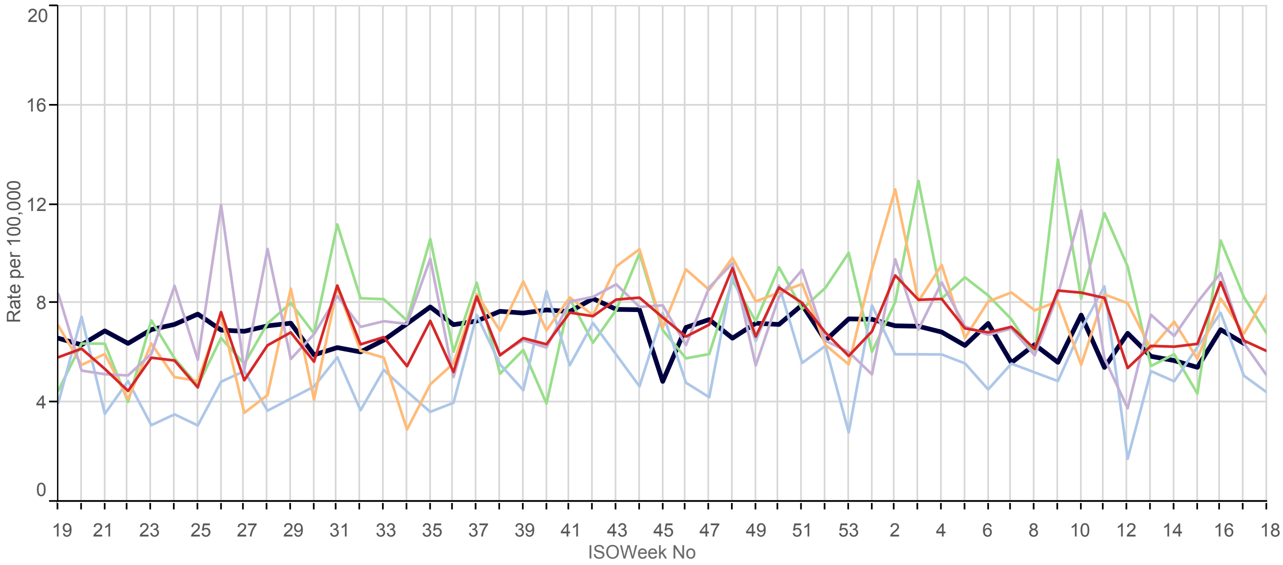

6. Disorders Affecting the Nervous System

National | North | South | London | Midlands And East | 5yr Avg

Peripheral Neuropathy (ICD10 : G50 - G64; G70 - G72)

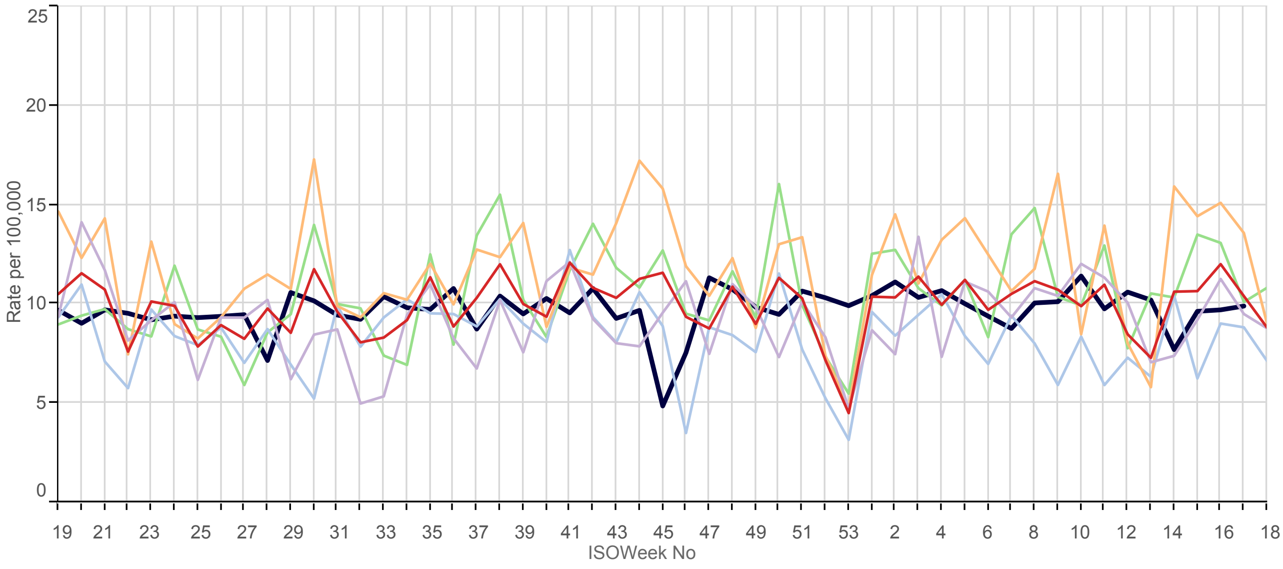

Meningitis and Encephalitis (ICD10 : A170 - A171; A 390; A83 - A85; A87; G00 - G05)

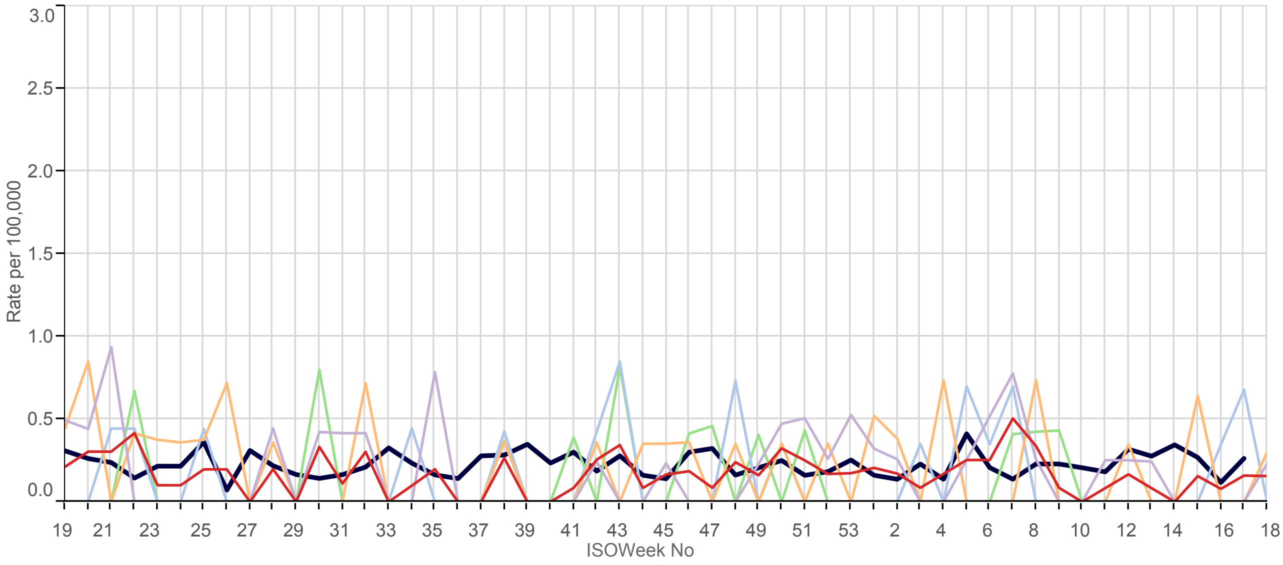

Musculoskeletal symptoms (ICD10 : R25 - R29)

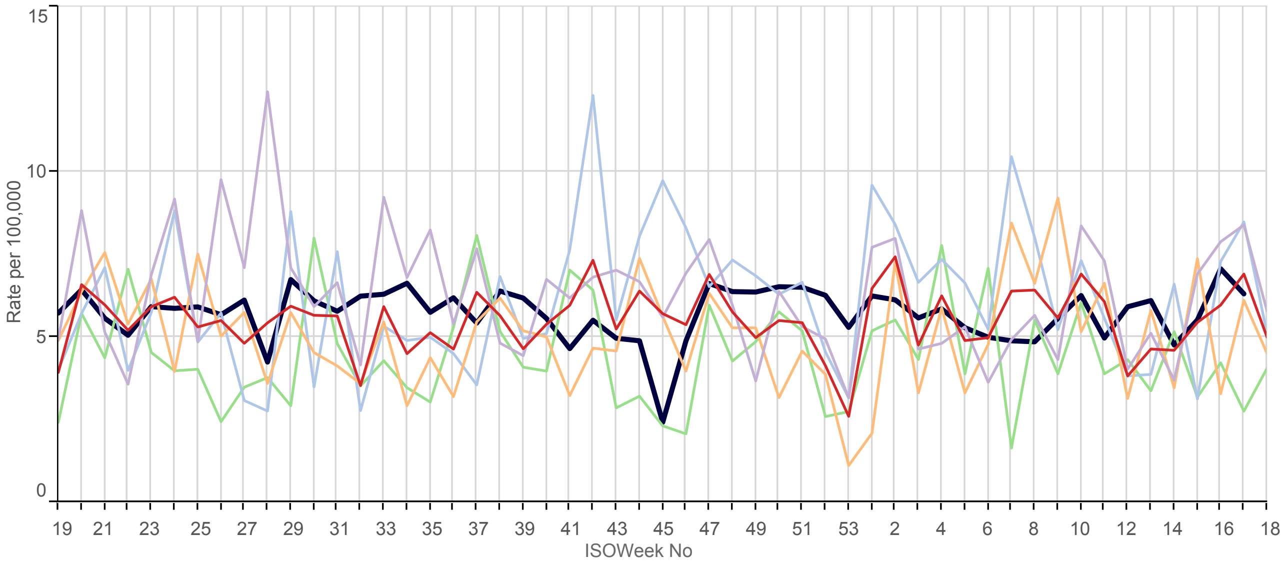

7. Genitourinary System Disorders

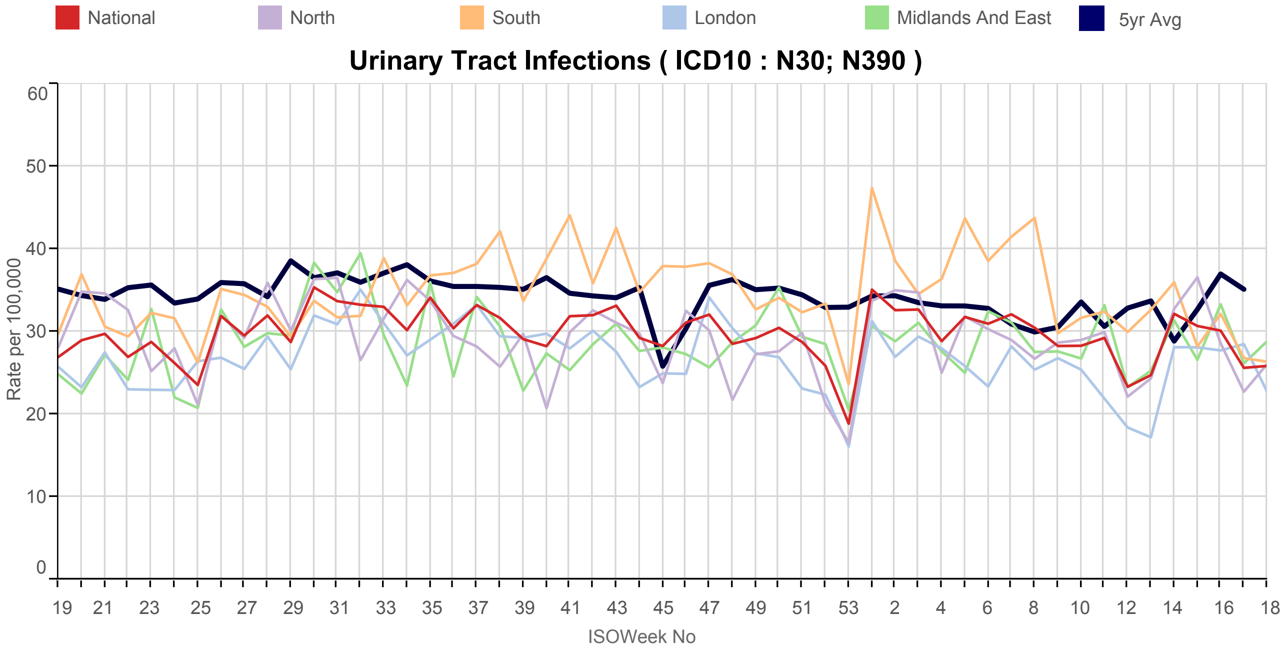

Incidence of Scarlatina

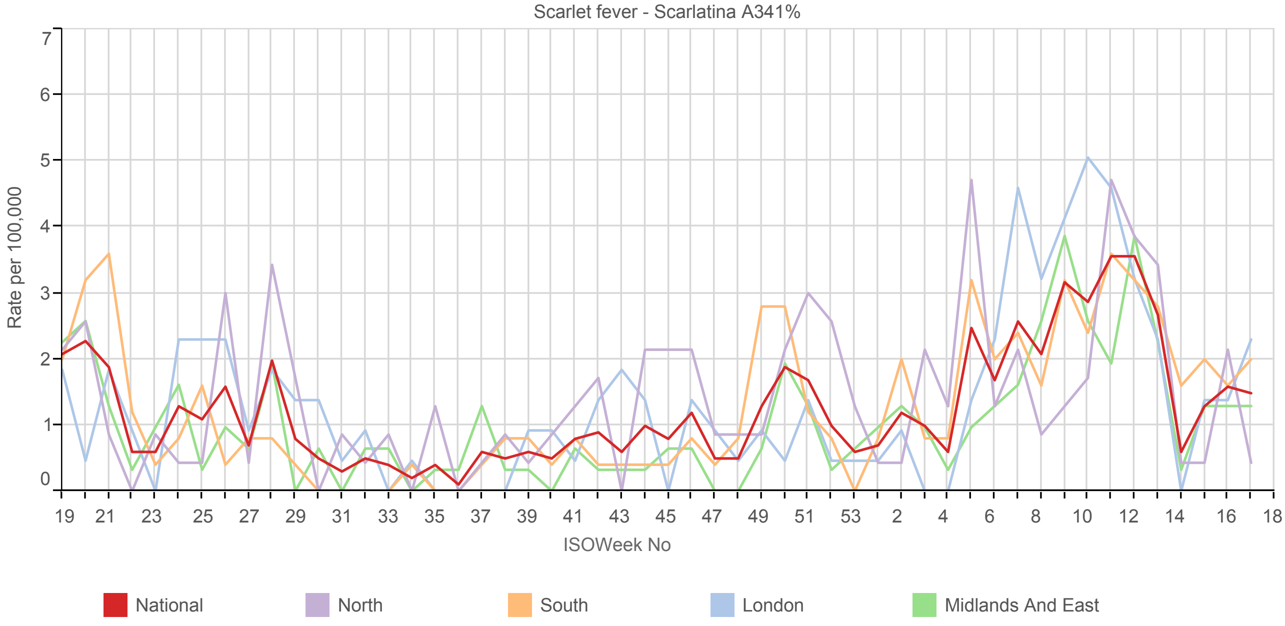

Weekly disease incidence by age group

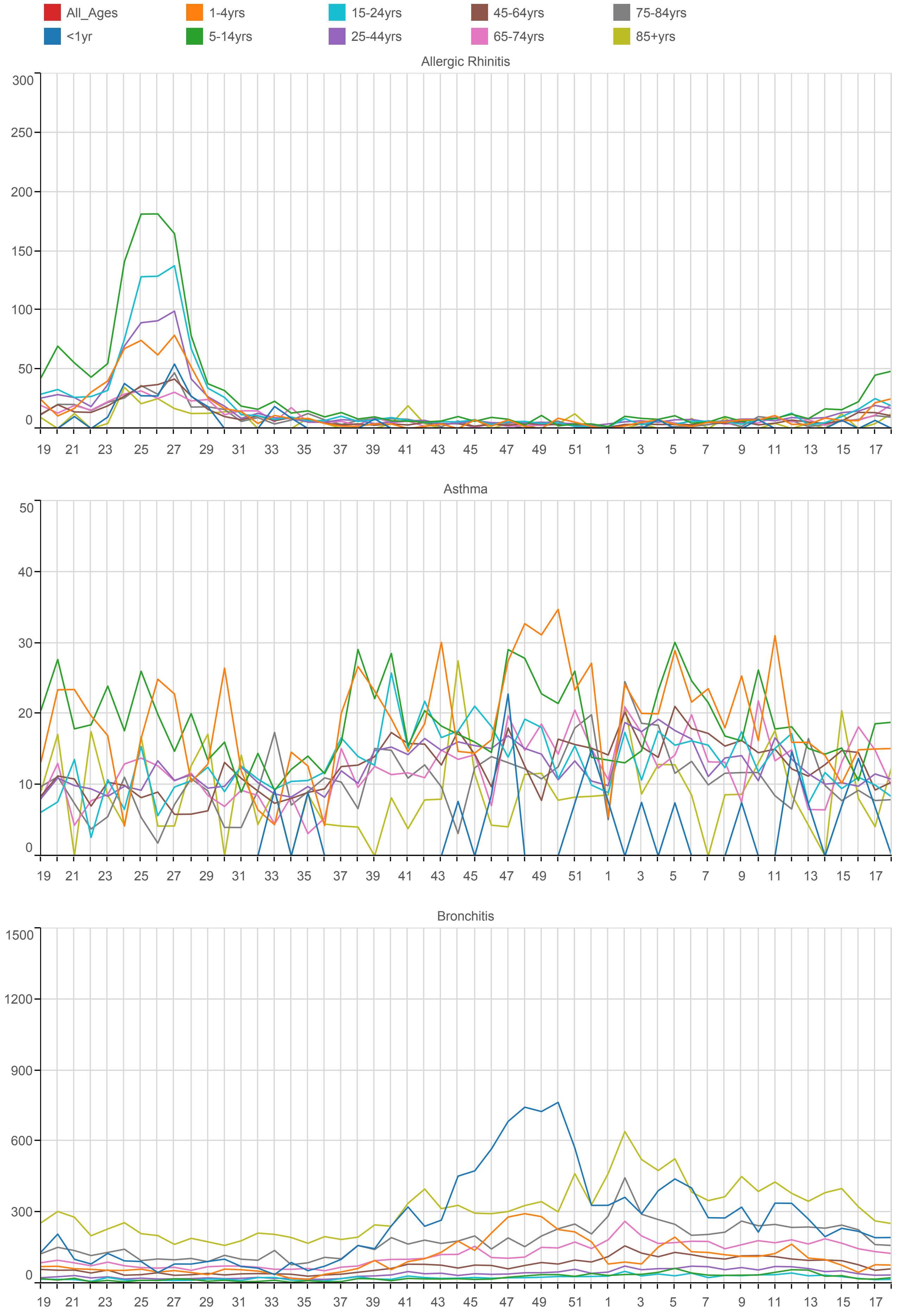

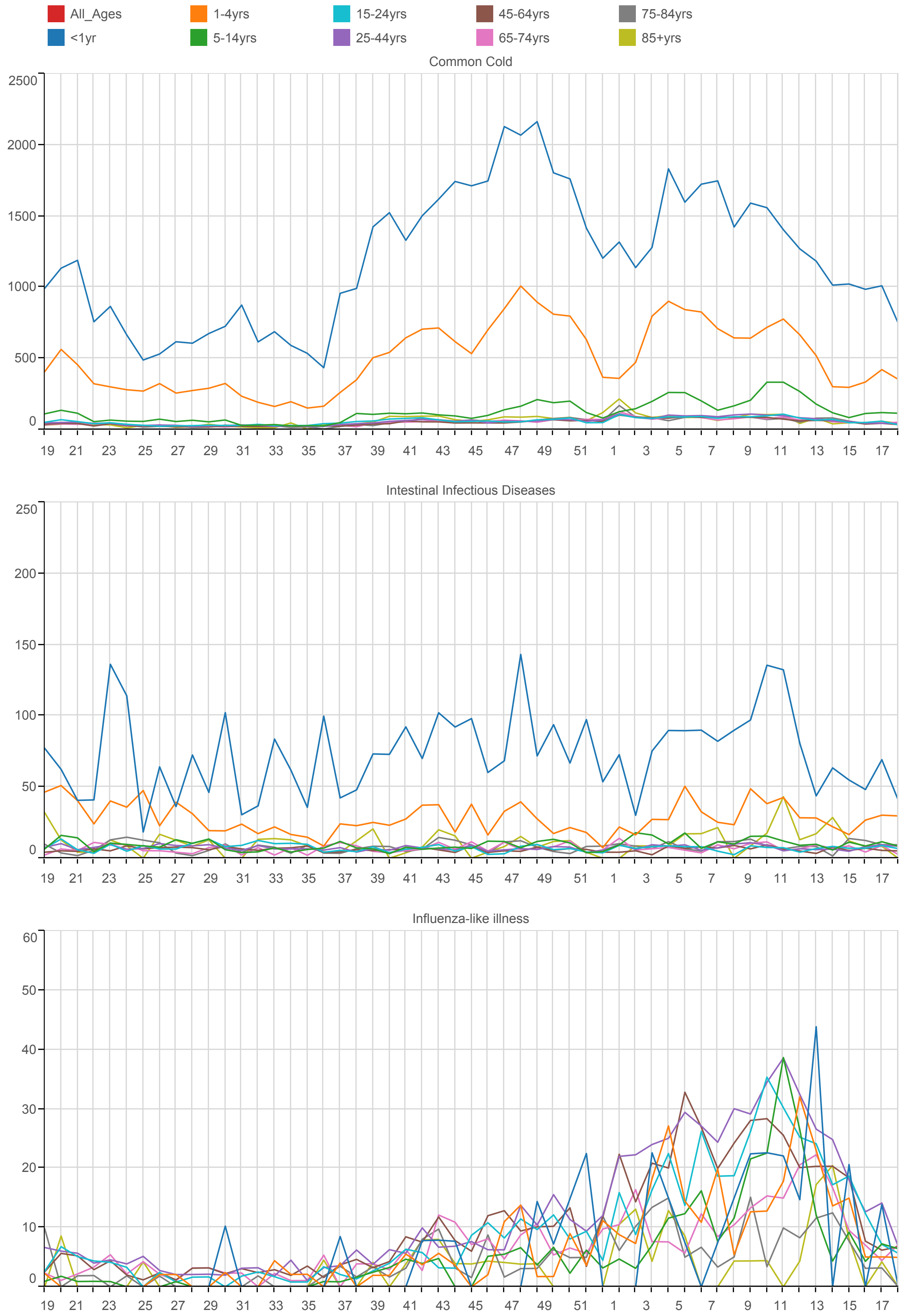
All_Ages
<1yr
1-4yrs
5-14yrs
15-24yrs
25-44yrs
45-64yrs
65-74yrs
75-84yrs
85+yrs
Common Cold
0
500
1000
1500
2000
2500
19 21 23 25 27 29 31 33 35 37 39 41 43 45 47 49 51 1 3 5 7 9 11 13 15 17
Intestinal Infectious Diseases
0
50
100
150
200
250
19 21 23 25 27 29 31 33 35 37 39 41 43 45 47 49 51 1 3 5 7 9 11 13 15 17
Influenza-like illness
0
10
20
30
40
50
60
19 21 23 25 27 29 31 33 35 37 39 41 43 45 47 49 51 1 3 5 7 9 11 13 15 17

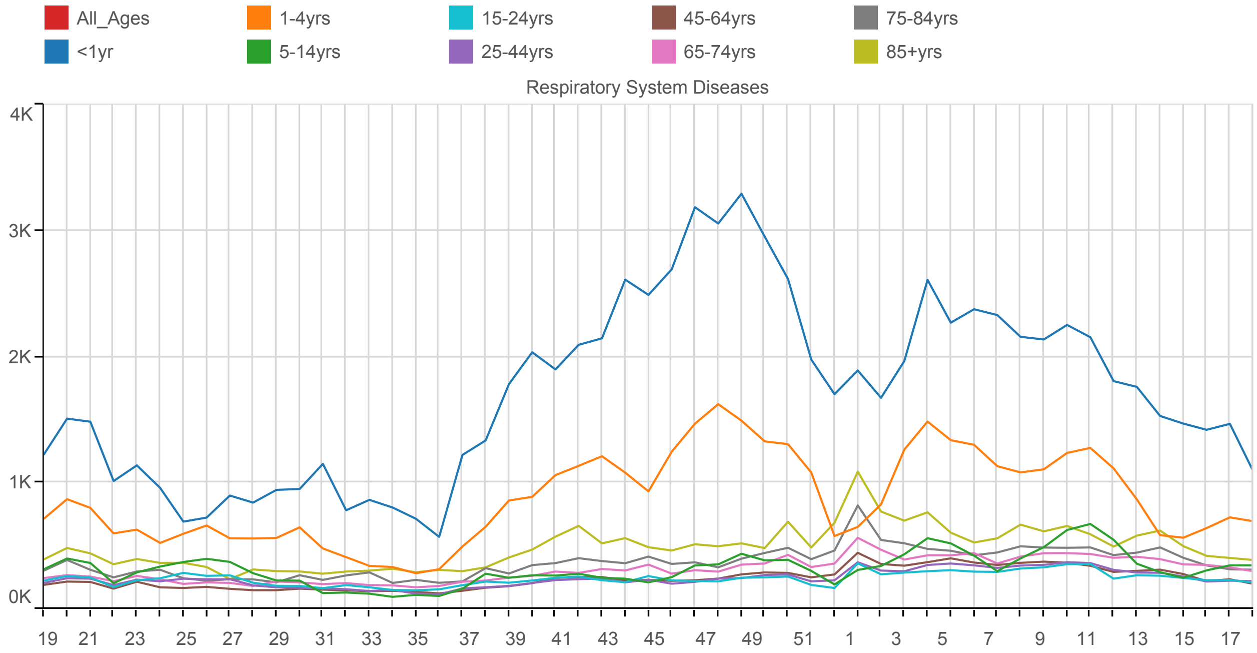

All_Ages
1-4yrs
15-24yrs
45-64yrs
75-84yrs
<1yr
5-14yrs
25-44yrs
65-74yrs
85+yrs
Respiratory System Diseases
4K
3K
2K
1K
0K
19 21 23 25 27 29 31 33 35 37 39 41 43 45 47 49 51 1 3 5 7 9 11 13 15 17

14. Appendix B: Demographic distribution by condition

Hayfever/Allergic Rhinitis (ICD10: J30)

Age-sex profile

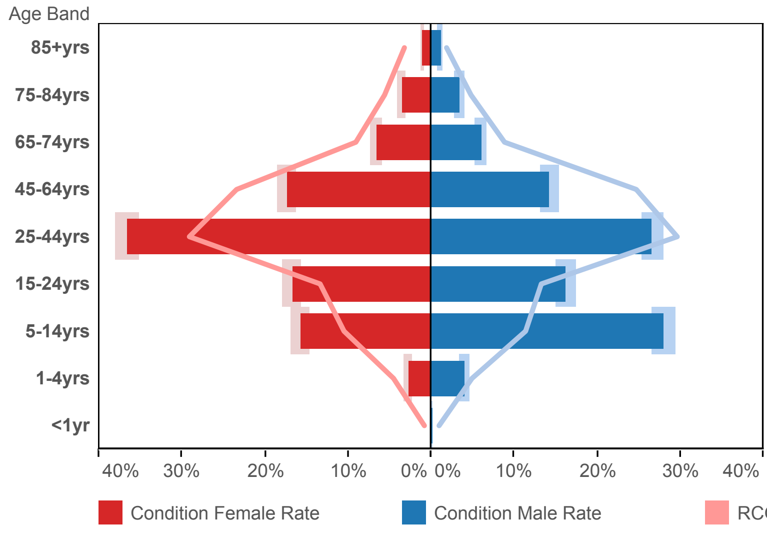

Age Band	Condition Female Rate	Condition Male Rate	Condition Female Population	Condition Male Population
**<1yr**	0.02	0.08	1	3
**1-4yrs**	2.70	4.10	119	158
**5-14yrs**	15.71	28.02	693	1,080
**15-24yrs**	16.69	16.21	736	625
**25-44yrs**	36.55	26.69	1,612	1,029
**45-64yrs**	17.27	14.29	762	551
**65-74yrs**	6.57	5.99	290	231
**75-84yrs**	3.49	3.48	154	134
**85+yrs**	1.00	1.14	44	44

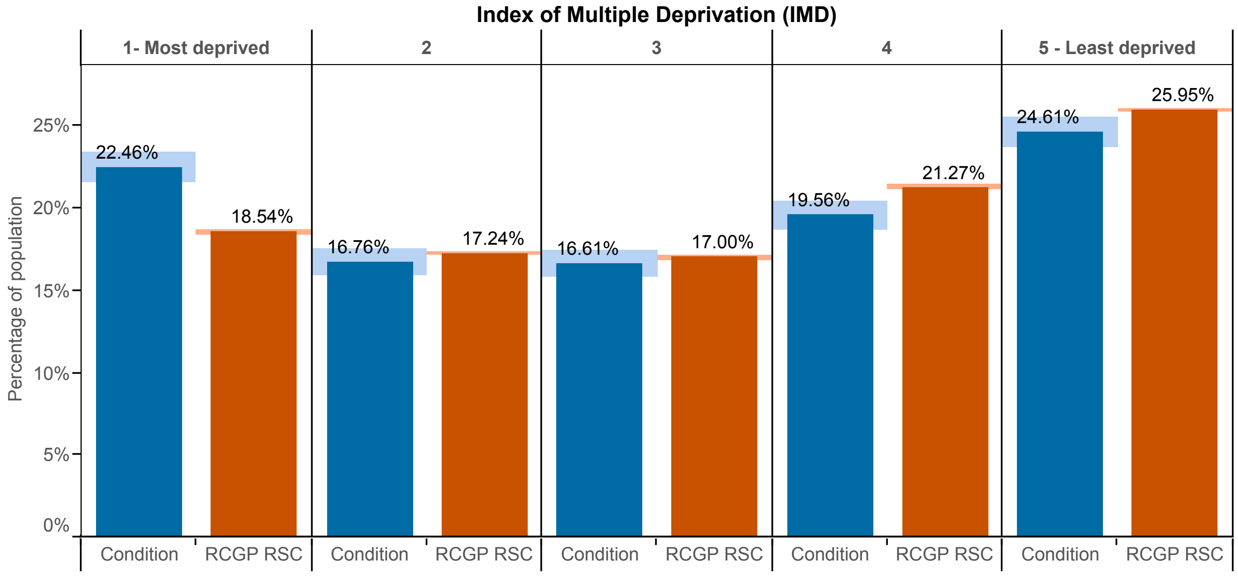

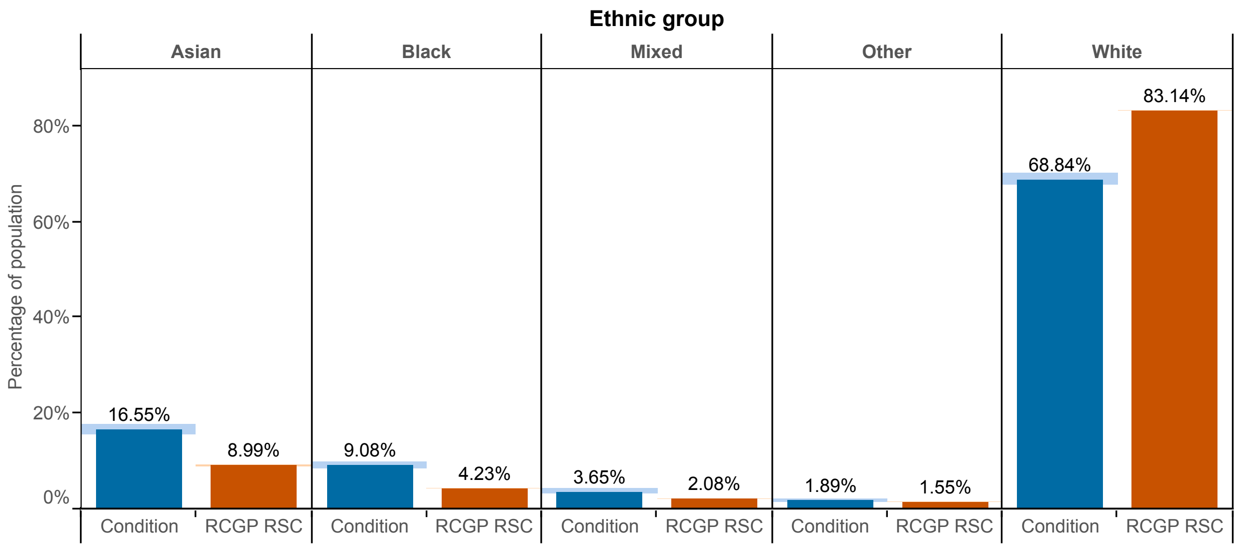

Asthma (ICD10 : J45 - J46)

Age-sex profile

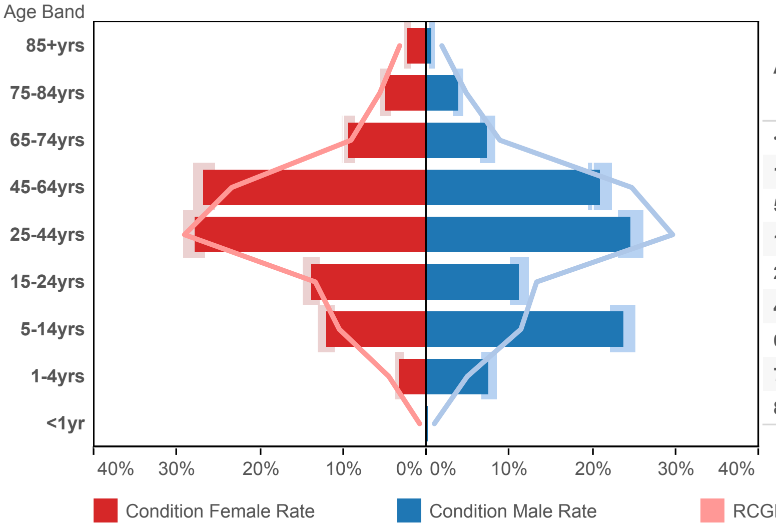

Age Band	Condition Female Rate	Condition Male Rate	Condition Female Population	Condition Male Population
<1yr	0.00	0.03	0	1
1-4yrs	3.15	7.61	139	229
5-14yrs	11.98	23.75	528	715
15-24yrs	13.84	11.16	610	336
25-44yrs	27.85	24.64	1,227	742
45-64yrs	26.76	20.96	1,179	631
65-74yrs	9.31	7.34	410	221
75-84yrs	4.88	3.85	215	116
85+yrs	2.22	0.66	98	20

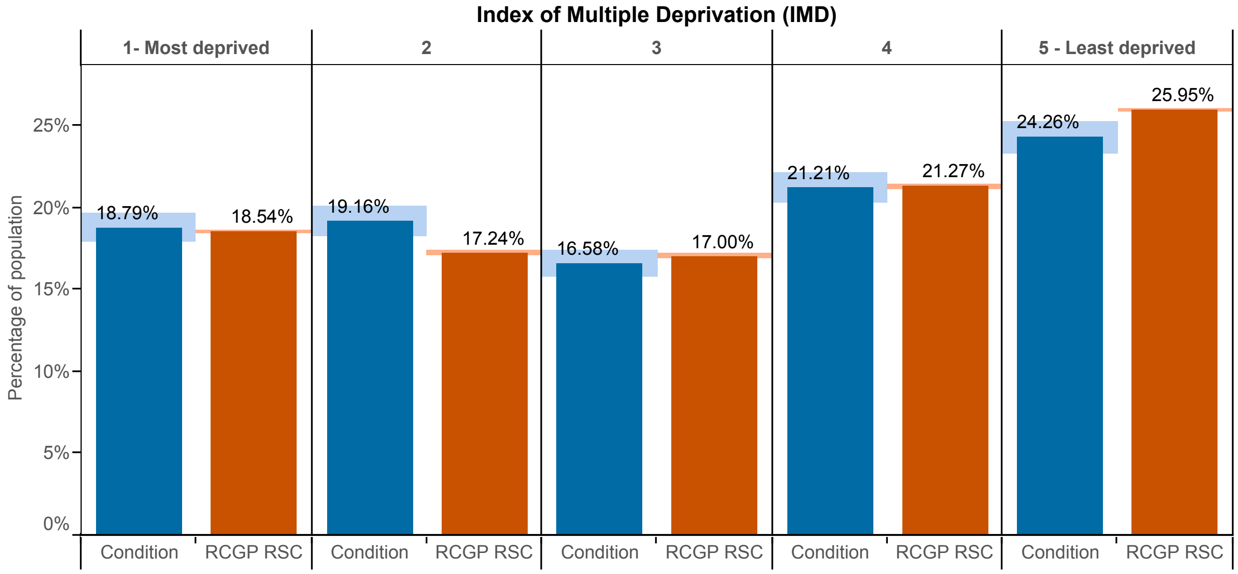

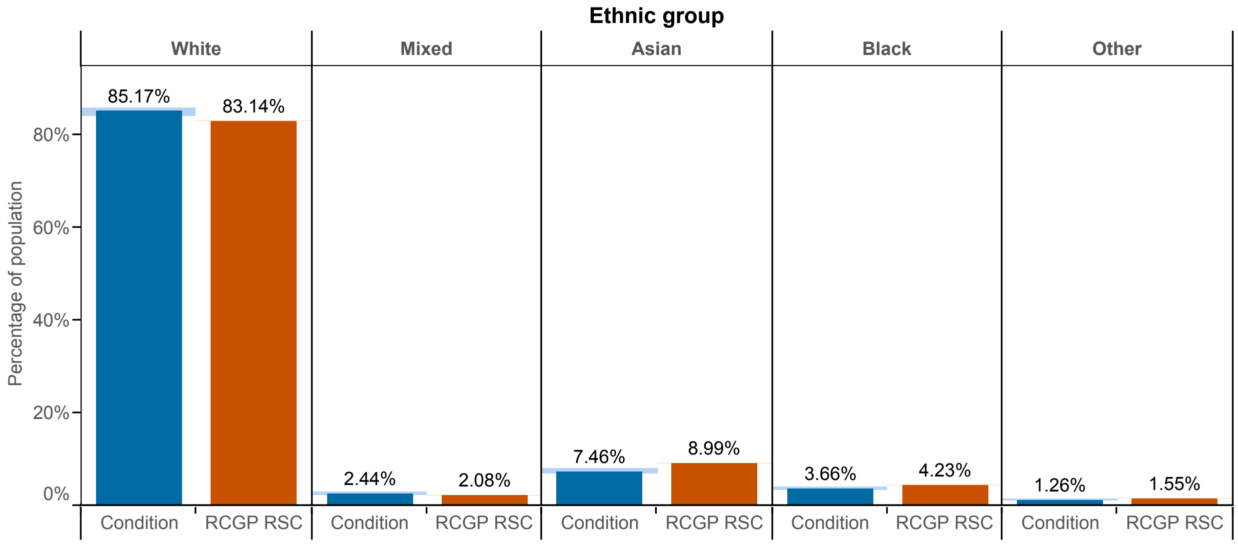

Bronchitis (ICD10: J20-J21,J40)

Age-sex profile

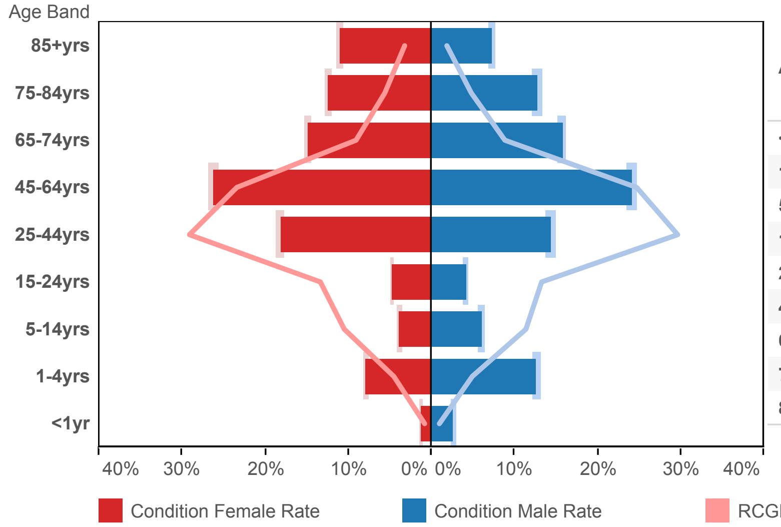

Age Band	Condition Female Rate	Condition Male Rate	Condition Female Population	Condition Male Population
<1yr	1.26	2.72	273	450
1-4yrs	7.82	12.67	1,694	2,100
5-14yrs	3.80	6.11	823	1,012
15-24yrs	4.66	4.17	1,008	691
25-44yrs	18.13	14.39	3,926	2,384
45-64yrs	26.22	24.13	5,676	3,998
65-74yrs	14.82	15.78	3,208	2,614
75-84yrs	12.37	12.79	2,679	2,119
85+yrs	10.91	7.25	2,363	1,201

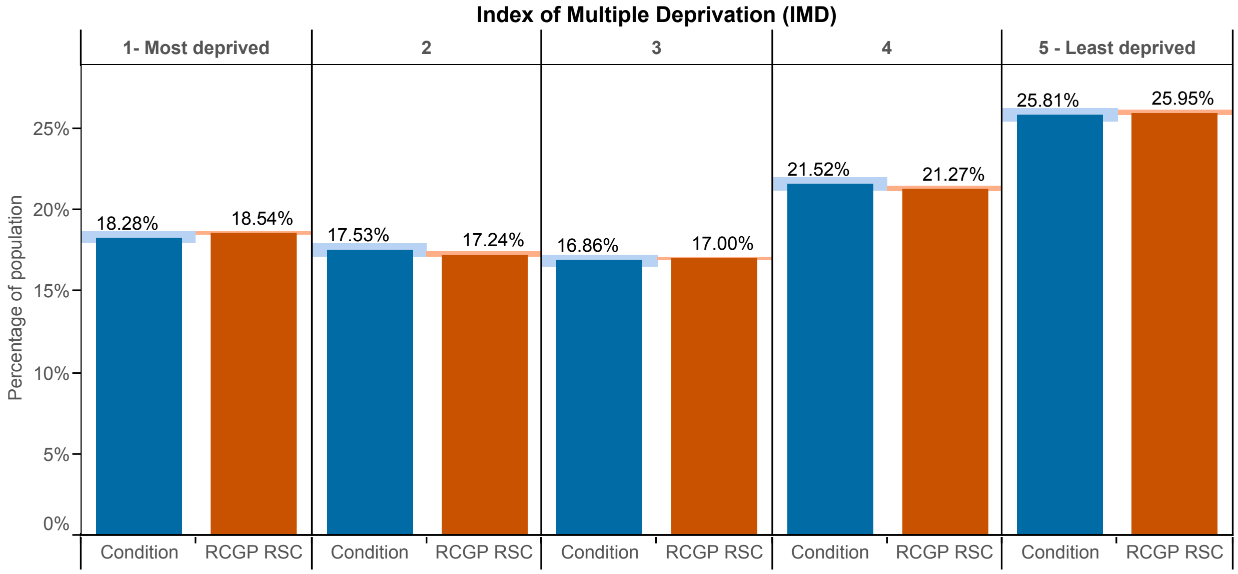

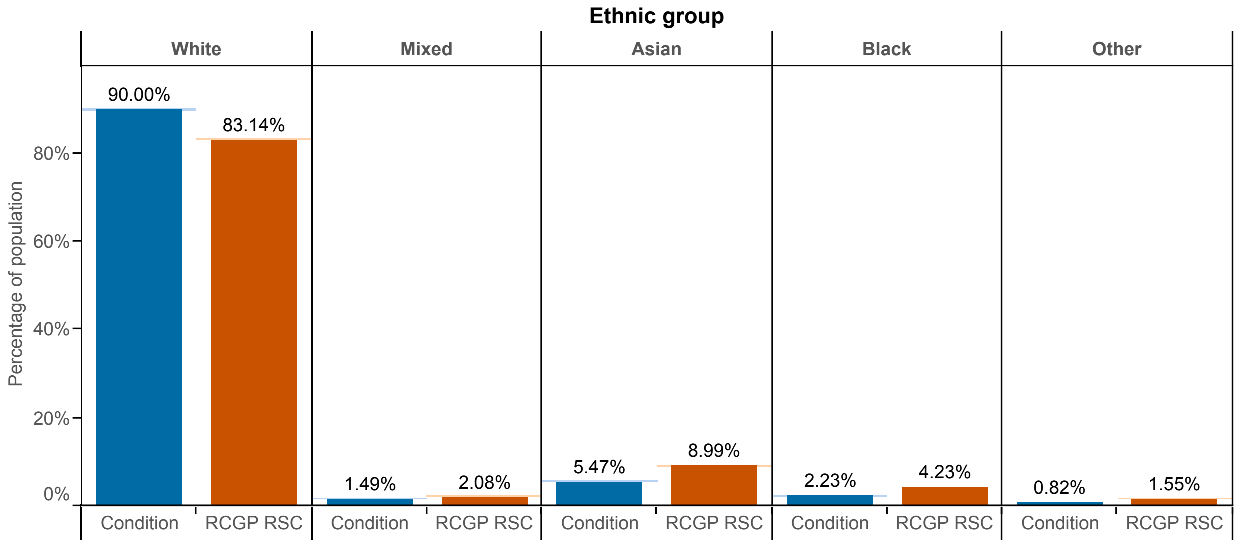

Bullous Dermatoses (ICD10 : L10 - L14)

Age-sex profile

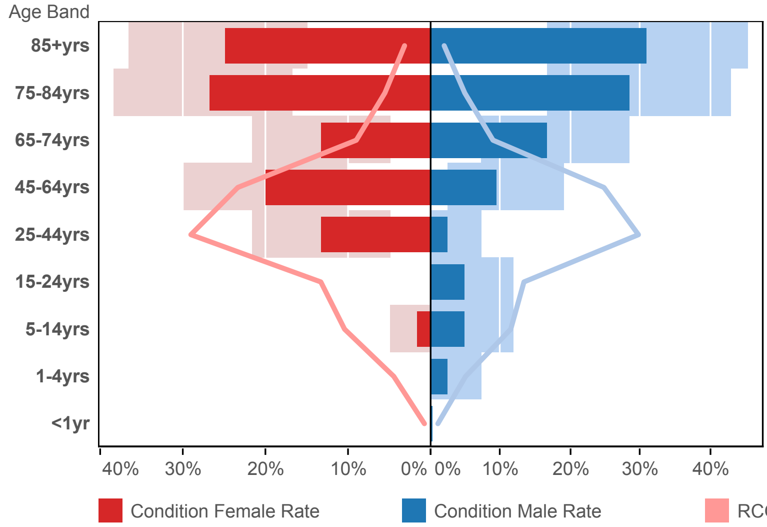

Age Band	Condition Female Rate	Condition Male Rate	Condition Female Population	Condition Male Population
<1yr	0.00	0.00	0.00	0.00
1-4yrs	0.00	2.38	0.00	1.00
5-14yrs	1.67	4.76	1.00	2.00
15-24yrs	0.00	4.76	0.00	2.00
25-44yrs	13.33	2.38	8.00	1.00
45-64yrs	20.00	9.52	12.00	4.00
65-74yrs	13.33	16.67	8.00	7.00
75-84yrs	26.67	28.57	16.00	12.00
85+yrs	25.00	30.95	15.00	13.00

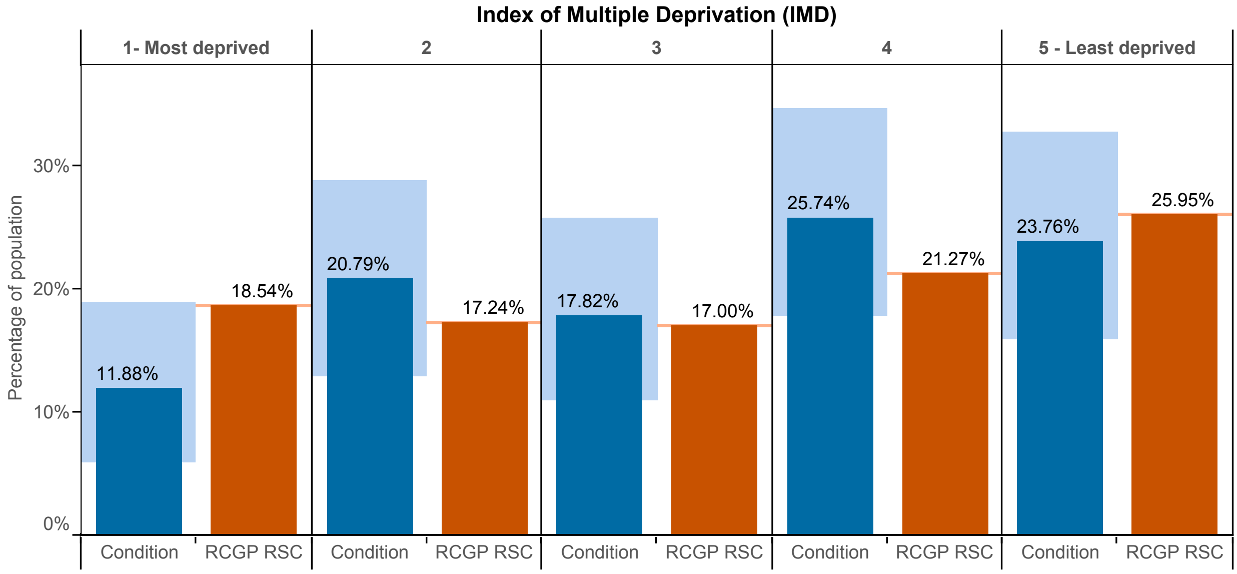

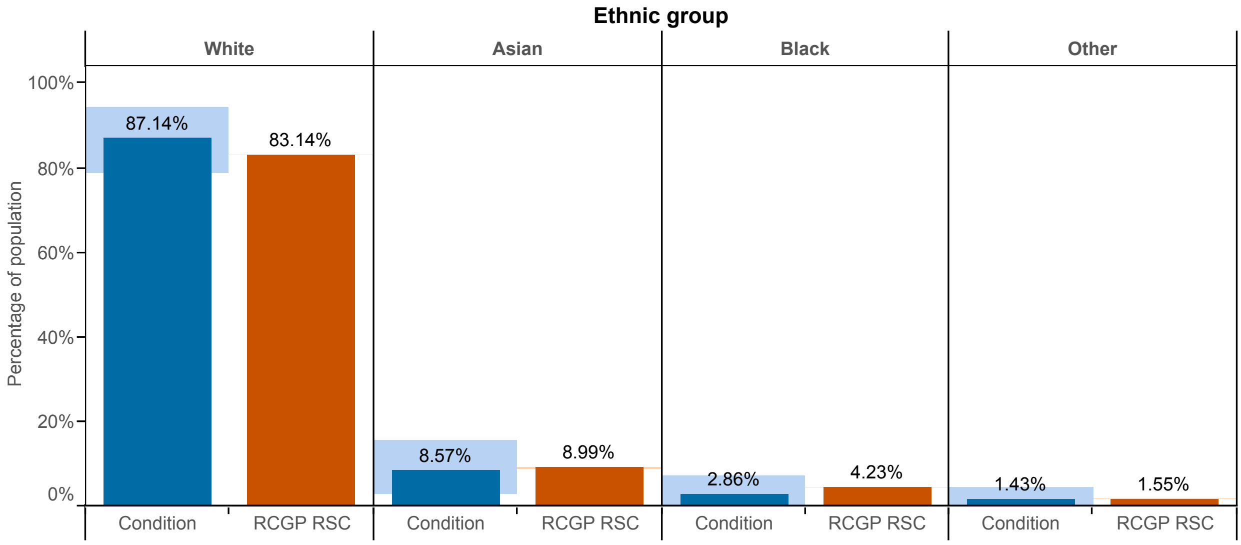

Chickenpox (ICD10 : B01)

Age-sex profile

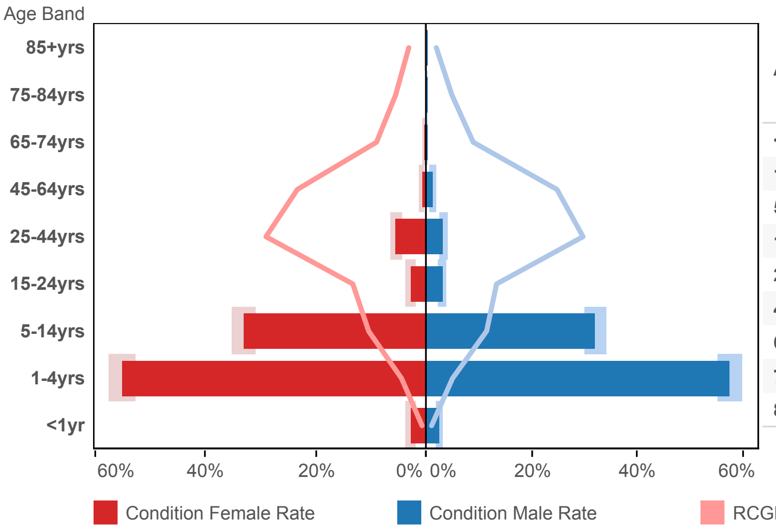

Age Band	Condition Female Rate	Condition Male Rate	Condition Female Population	Condition Male Population
<1yr	2.73	2.50	47.0	43.0
1-4yrs	55.08	57.63	948.0	993.0
5-14yrs	33.06	32.10	569.0	553.0
15-24yrs	2.73	3.08	47.0	53.0
25-44yrs	5.40	3.25	93.0	56.0
45-64yrs	0.70	1.28	12.0	22.0
65-74yrs	0.23	0.12	4.0	2.0
75-84yrs	0.00	0.06	0.0	1.0
85+yrs	0.06	0.00	1.0	0.0

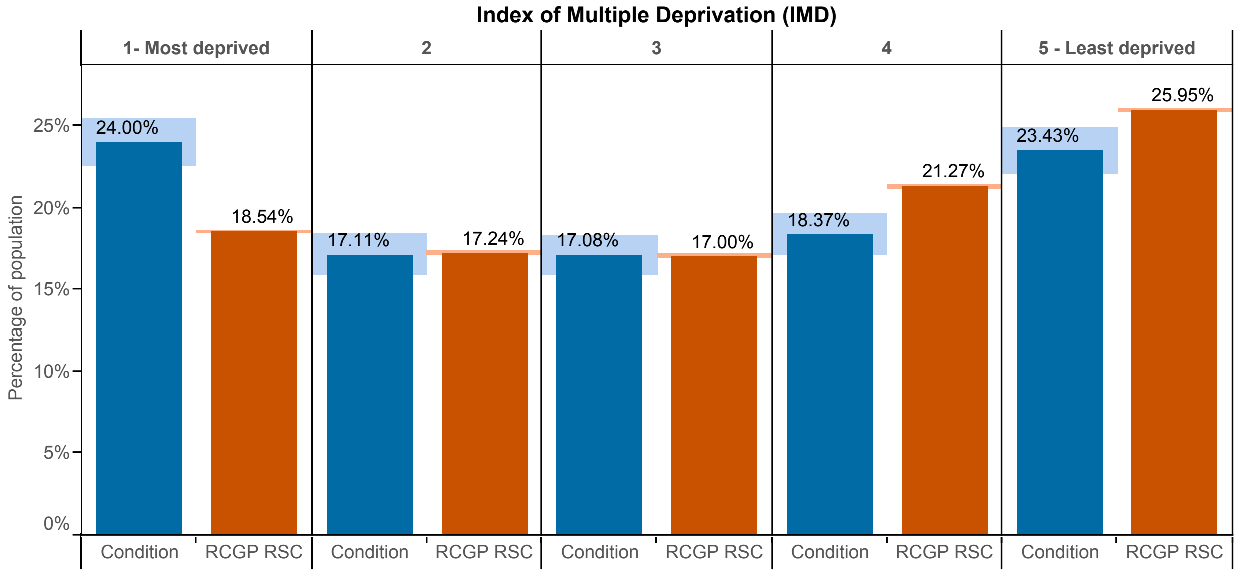

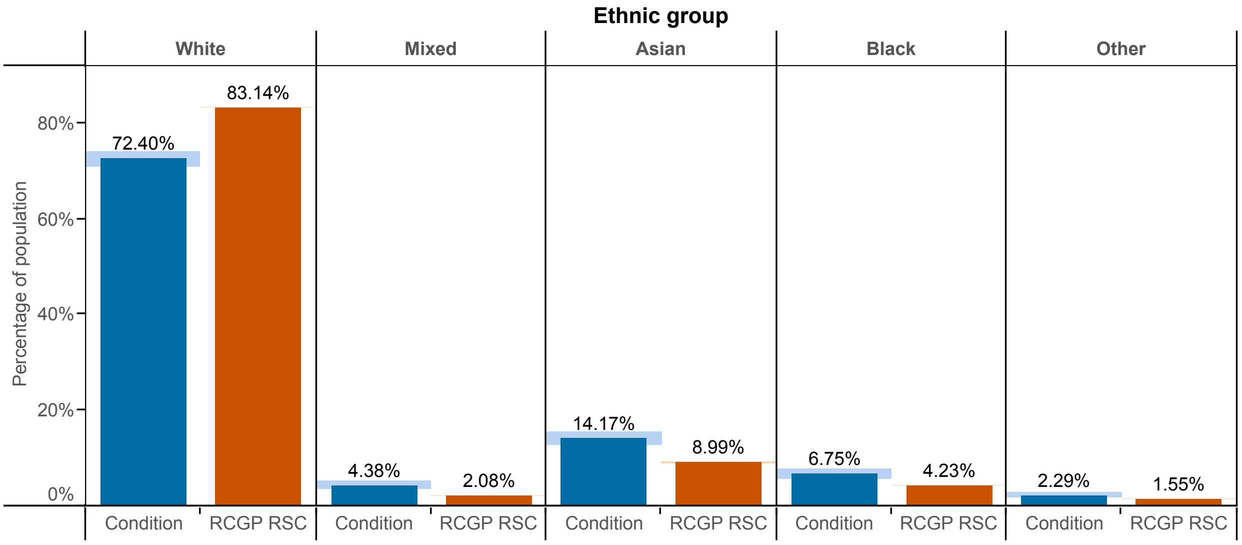

Common Cold (ICD10 : R05 - R07; R09)

Age-sex profile

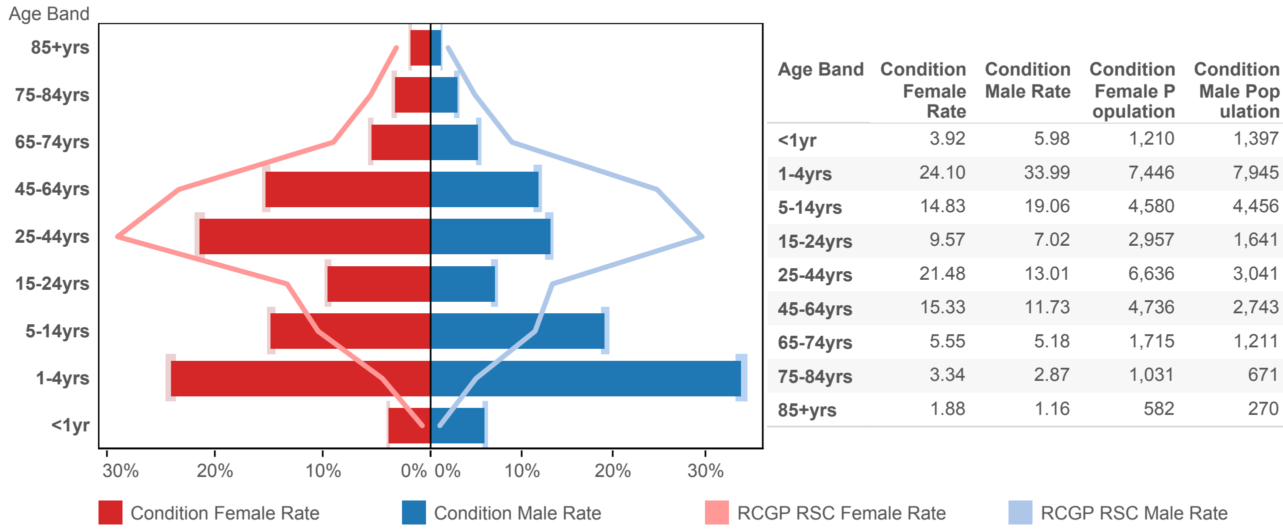

Age Band	Condition Female Rate	Condition Male Rate	Condition Female Population	Condition Male Population
<1yr	3.92	5.98	1,210	1,397
1-4yrs	24.10	33.99	7,446	7,945
5-14yrs	14.83	19.06	4,580	4,456
15-24yrs	9.57	7.02	2,957	1,641
25-44yrs	21.48	13.01	6,636	3,041
45-64yrs	15.33	11.73	4,736	2,743
65-74yrs	5.55	5.18	1,715	1,211
75-84yrs	3.34	2.87	1,031	671
85+yrs	1.88	1.16	582	270

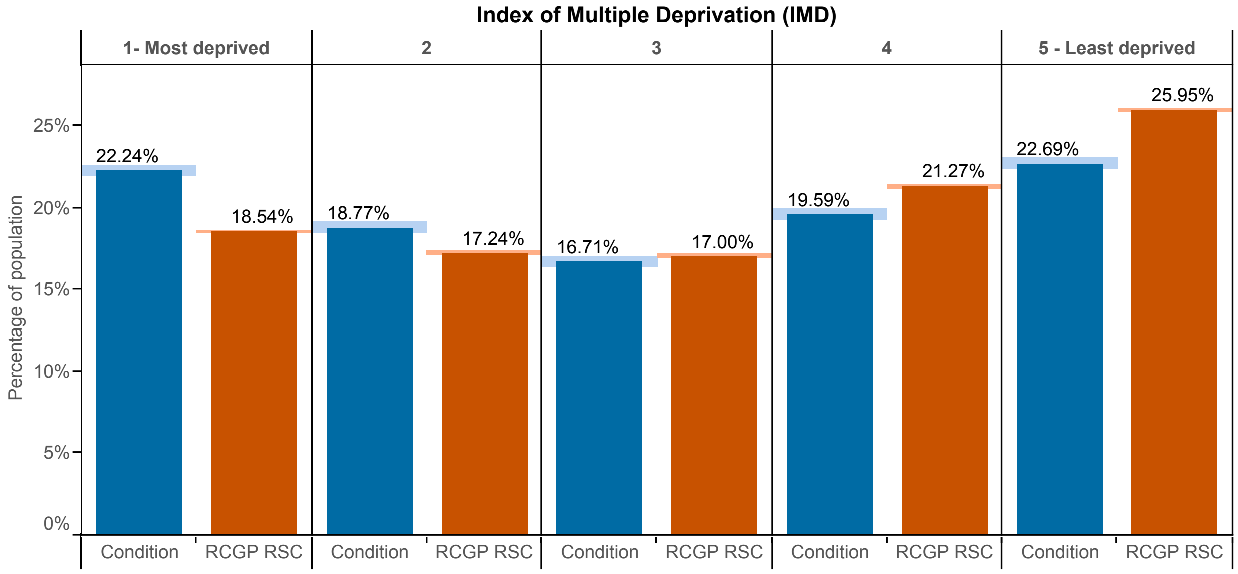

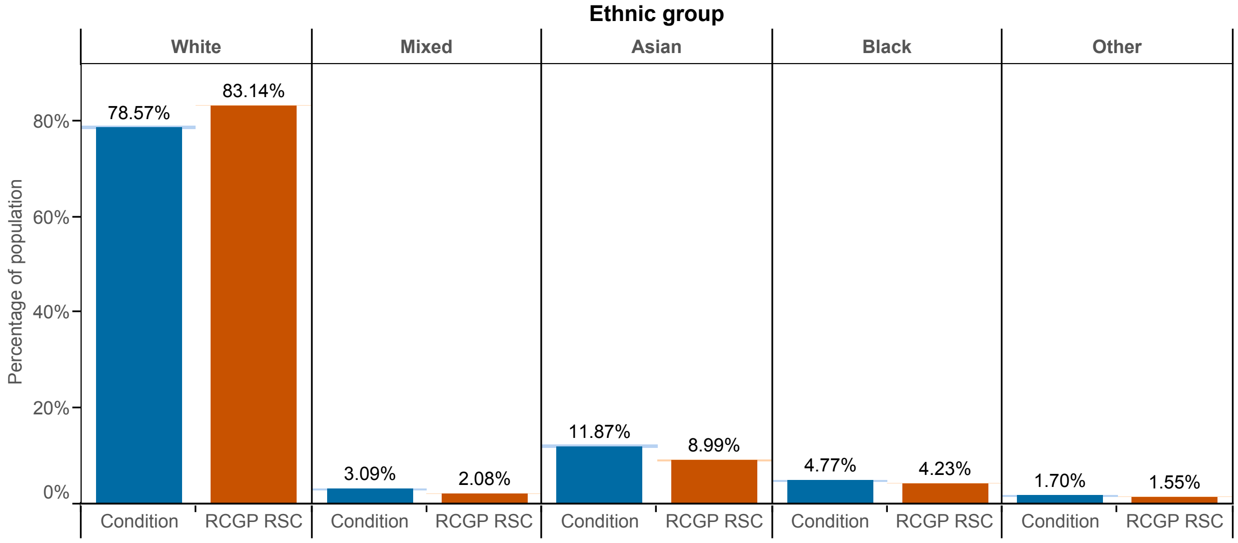

Conjunctivitis (ICD10 : H10 - H13)

Age-sex profile

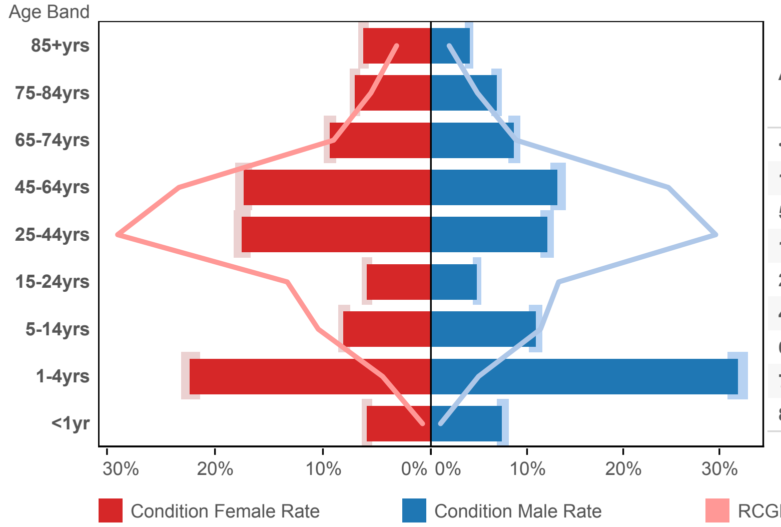

Age Band	Condition Female Rate	Condition Male Rate	Condition Female Population	Condition Male Population
<1yr	5.98	7.44	573	571
1-4yrs	22.36	31.95	2,141	2,451
5-14yrs	8.13	10.92	778	838
15-24yrs	5.92	4.82	567	370
25-44yrs	17.56	12.14	1,681	931
45-64yrs	17.37	13.27	1,663	1,018
65-74yrs	9.37	8.64	897	663
75-84yrs	7.02	6.80	672	522
85+yrs	6.30	4.01	603	308

Index of Multiple Deprivation (IMD)

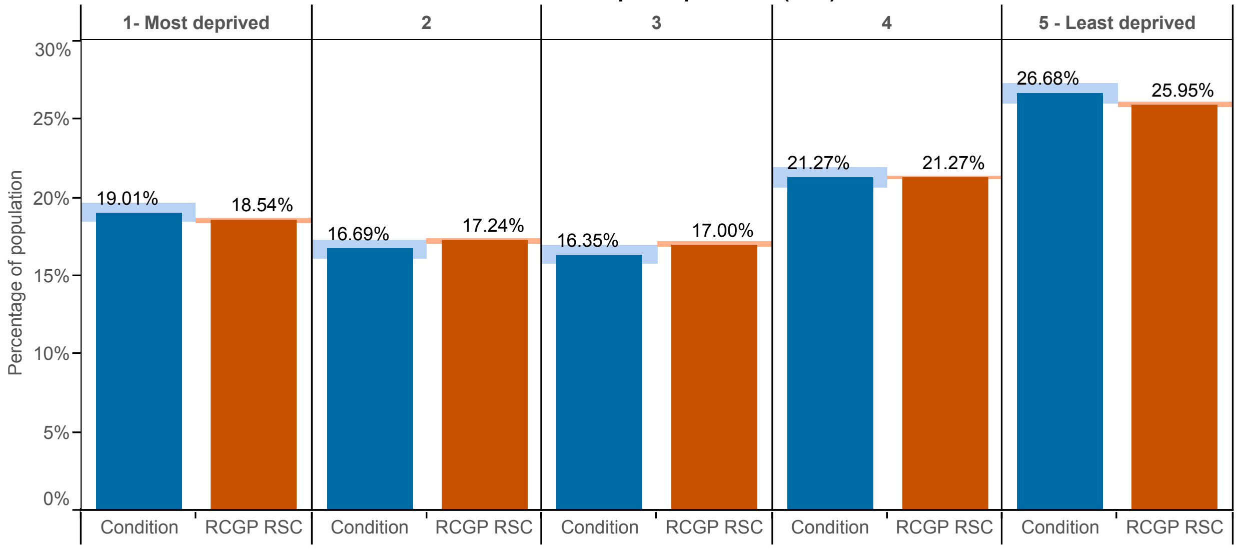

Ethnic group

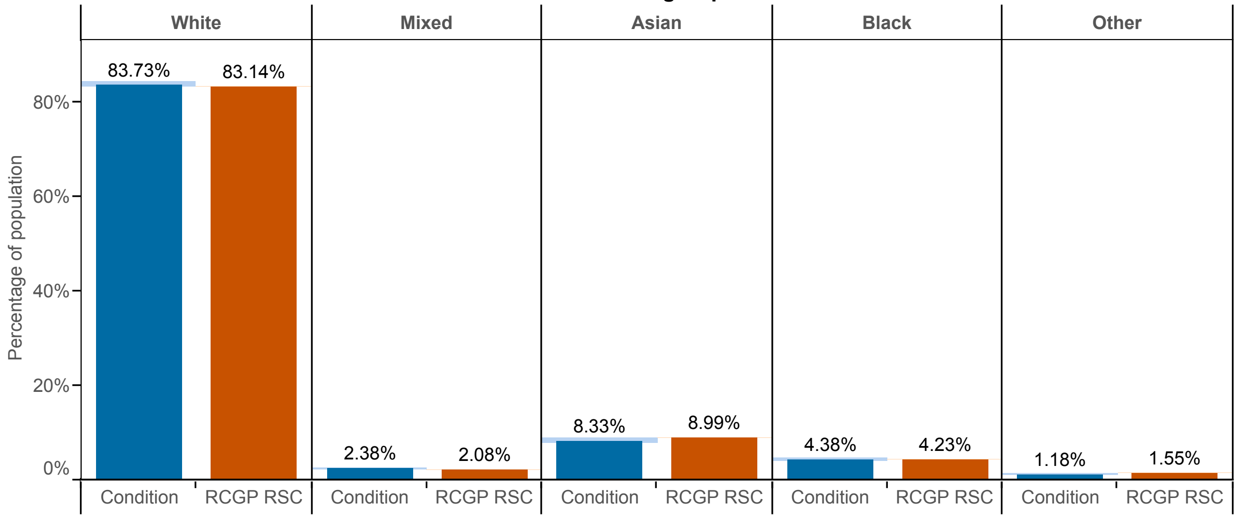

Herpes Simplex (ICD10 : B00)

Age-sex profile

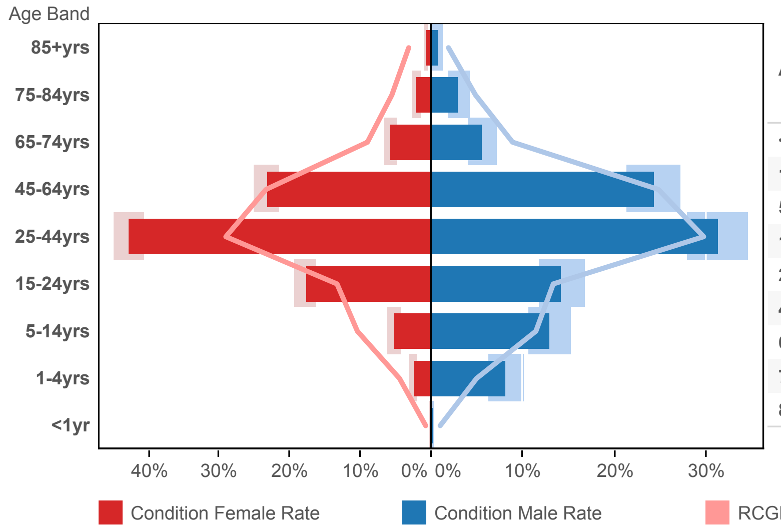

Age Band	Condition Female Rate	Condition Male Rate	Condition Female Population	Condition Male Population
<1yr	0.05	0.12	1.0	1.0
1-4yrs	2.51	8.08	54.0	65.0
5-14yrs	5.20	12.81	112.0	103.0
15-24yrs	17.79	14.18	383.0	114.0
25-44yrs	42.73	31.22	920.0	251.0
45-64yrs	23.18	24.25	499.0	195.0
65-74yrs	5.76	5.60	124.0	45.0
75-84yrs	2.09	2.99	45.0	24.0
85+yrs	0.70	0.75	15.0	6.0

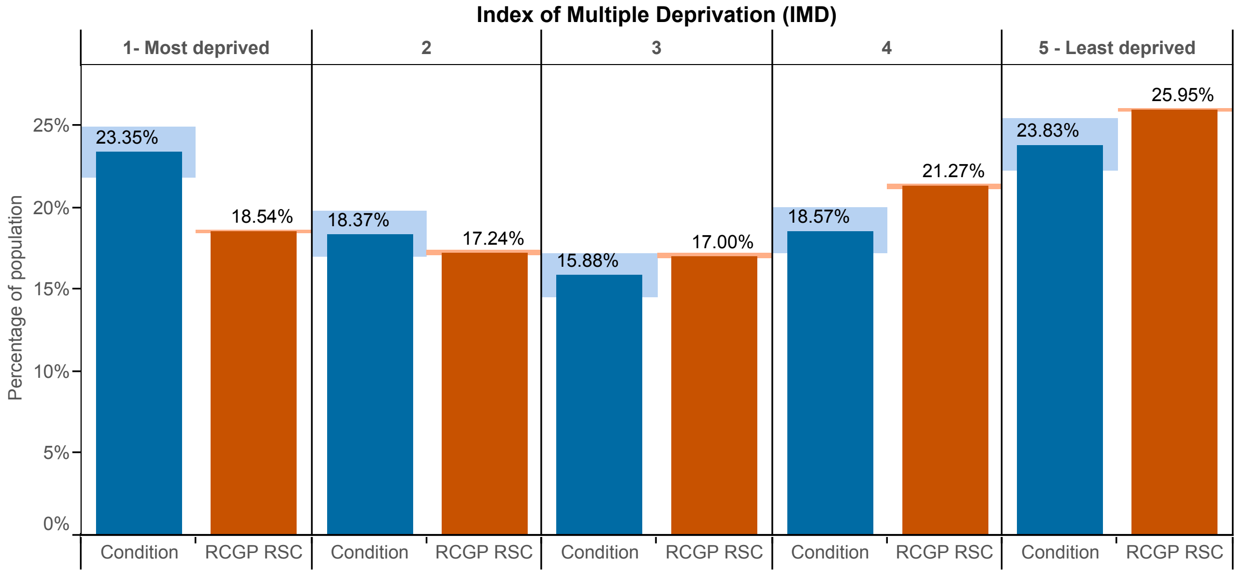

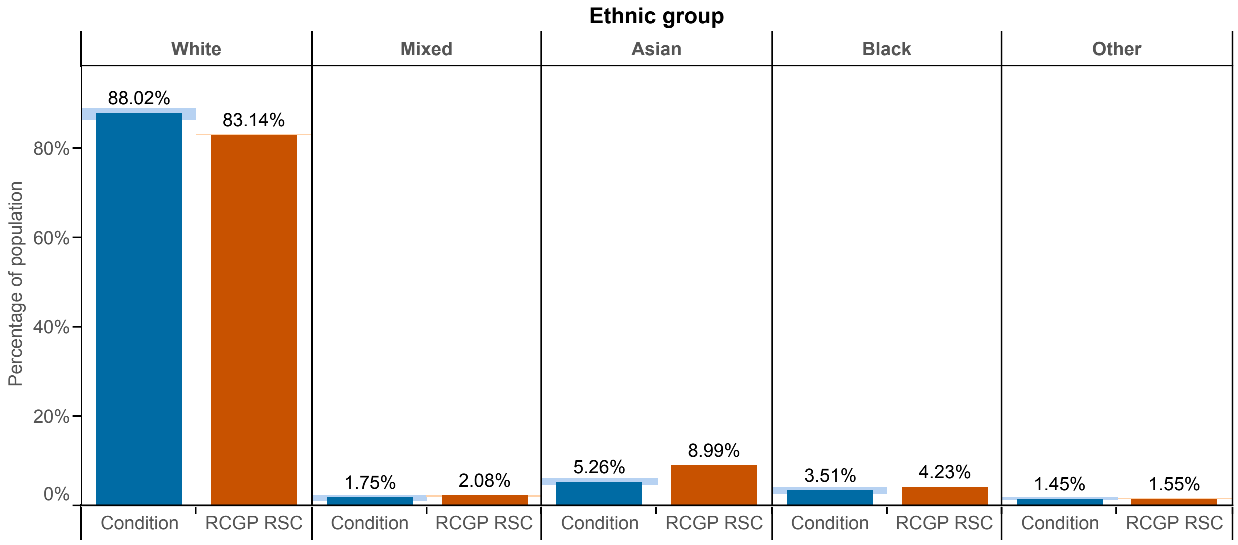

Herpes Zoster (ICD10 : B02)

Age-sex profile

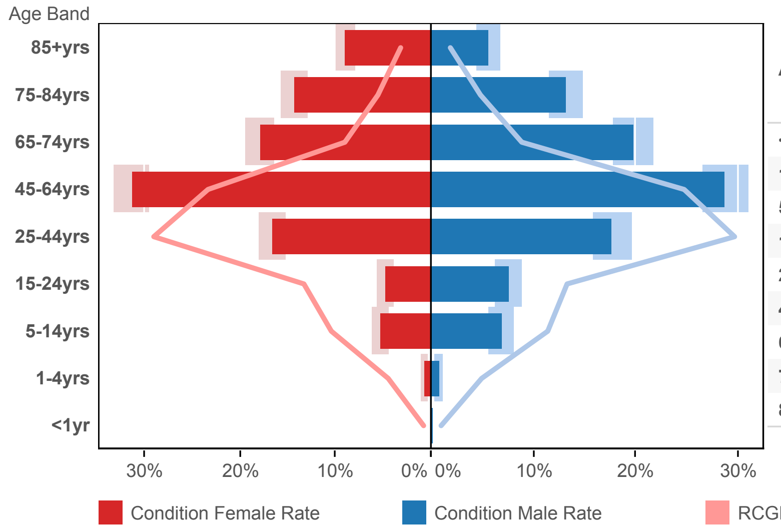

Age Band	Condition Female Rate	Condition Male Rate	Condition Female Population	Condition Male Population
<1yr	0.00	0.00	0.0	0.0
1-4yrs	0.68	0.76	17.0	12.0
5-14yrs	5.34	6.87	133.0	108.0
15-24yrs	4.82	7.51	120.0	118.0
25-44yrs	16.62	17.63	414.0	277.0
45-64yrs	31.35	28.71	781.0	451.0
65-74yrs	17.86	19.80	445.0	311.0
75-84yrs	14.29	13.18	356.0	207.0
85+yrs	9.03	5.54	225.0	87.0

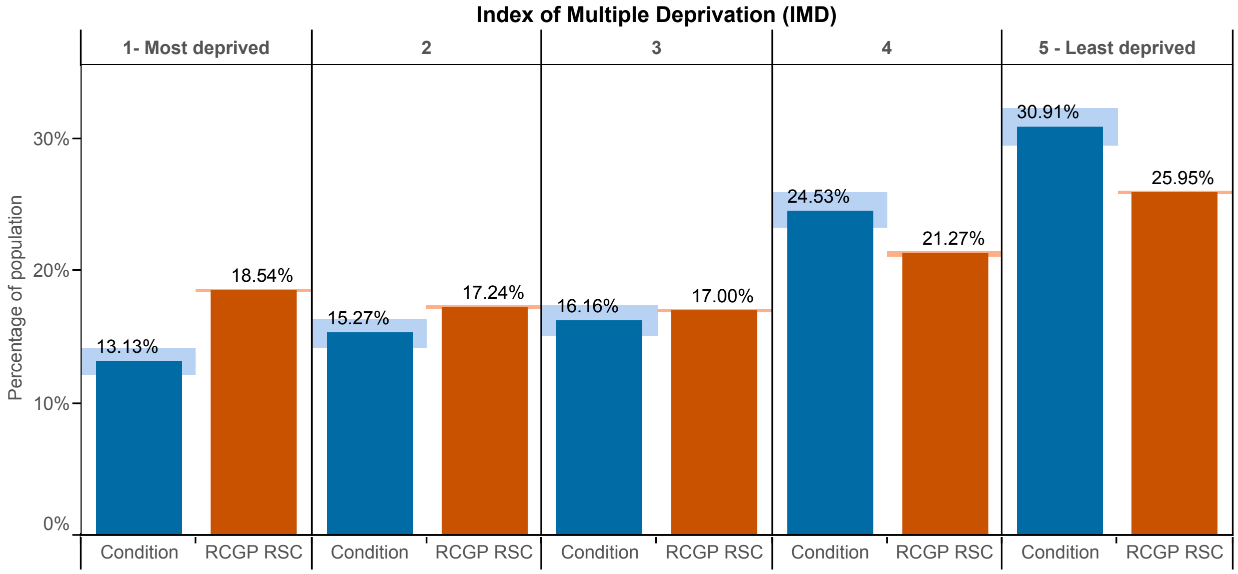

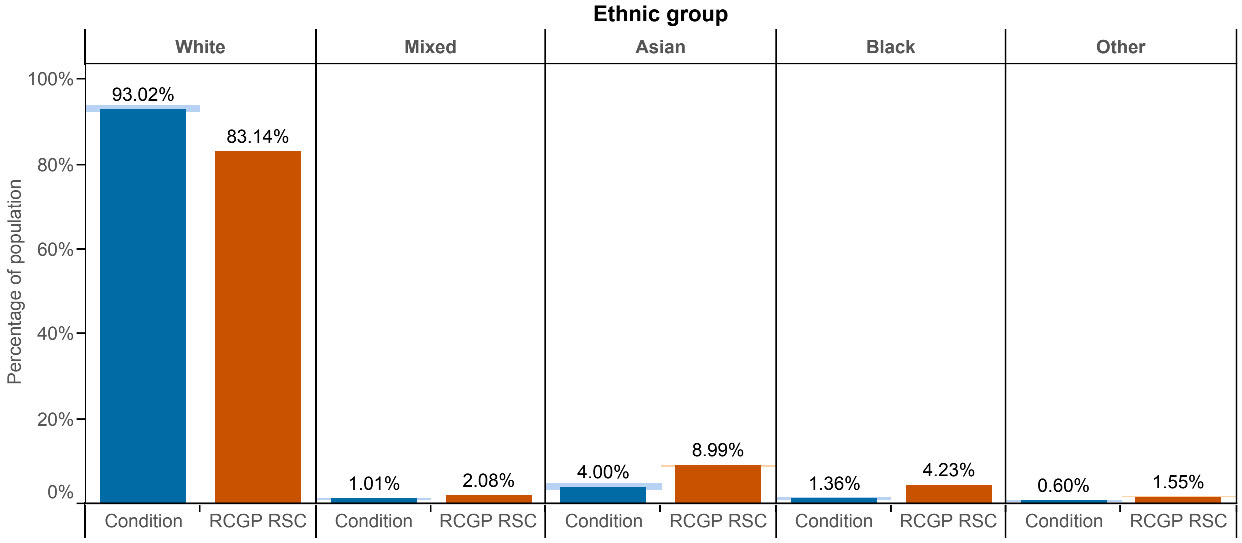

Impetigo (ICD10 - L01)

Age-sex profile

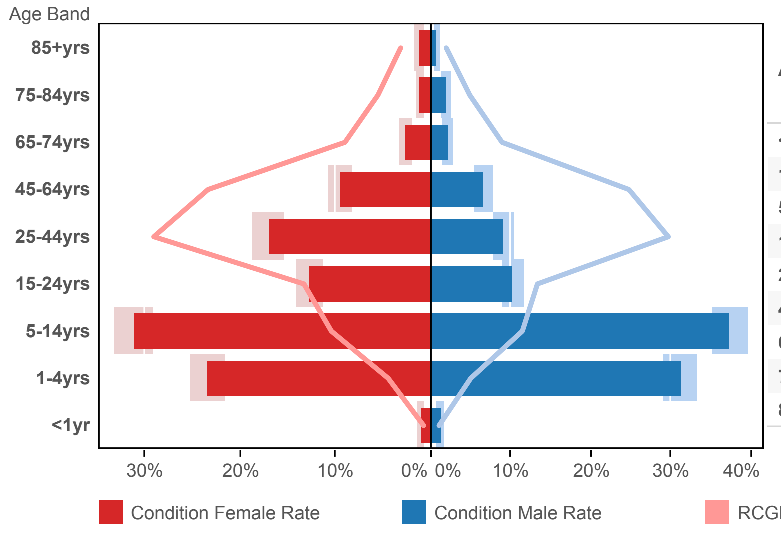

Age Band	Condition Female Rate	Condition Male Rate	Condition Female Population	Condition Male Population
<1yr	1.03	1.19	21.0	22.0
1-4yrs	23.46	31.16	480.0	574.0
5-14yrs	31.18	37.35	638.0	688.0
15-24yrs	12.71	10.15	260.0	187.0
25-44yrs	17.06	9.07	349.0	167.0
45-64yrs	9.53	6.57	195.0	121.0
65-74yrs	2.64	2.01	54.0	37.0
75-84yrs	1.17	1.85	24.0	34.0
85+yrs	1.22	0.65	25.0	12.0

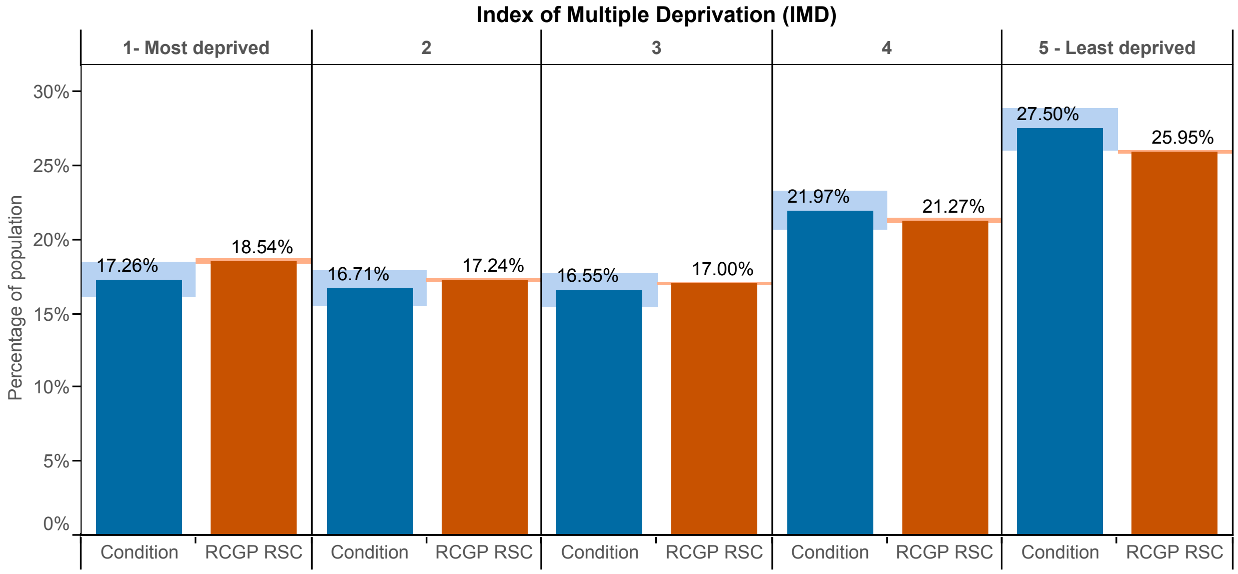

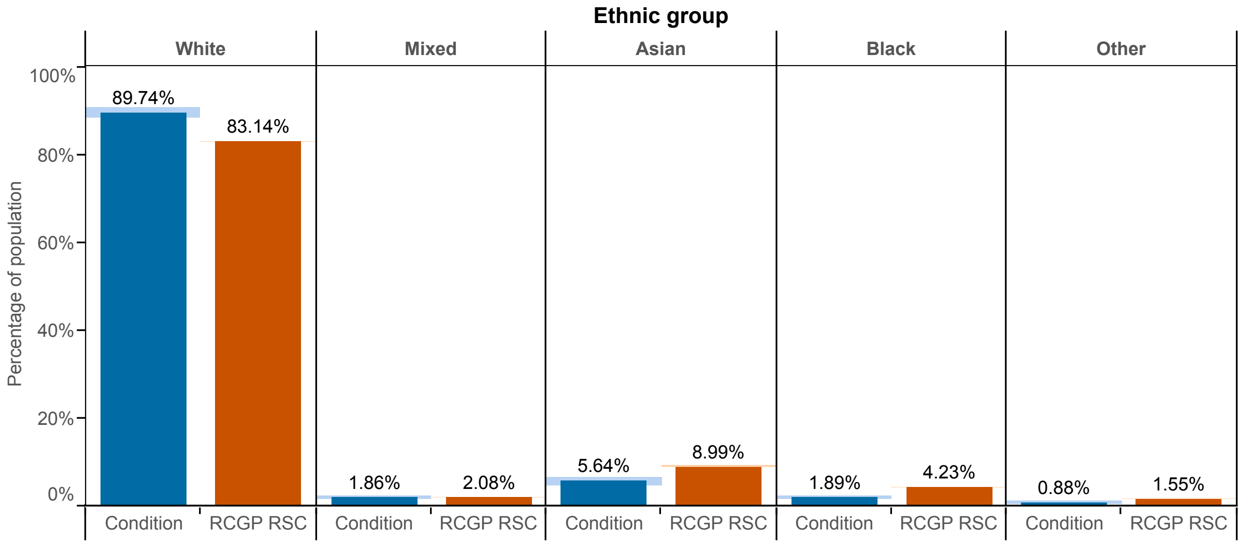

Infectious Mononucleosis (ICD10 : B27)

Age-sex profile

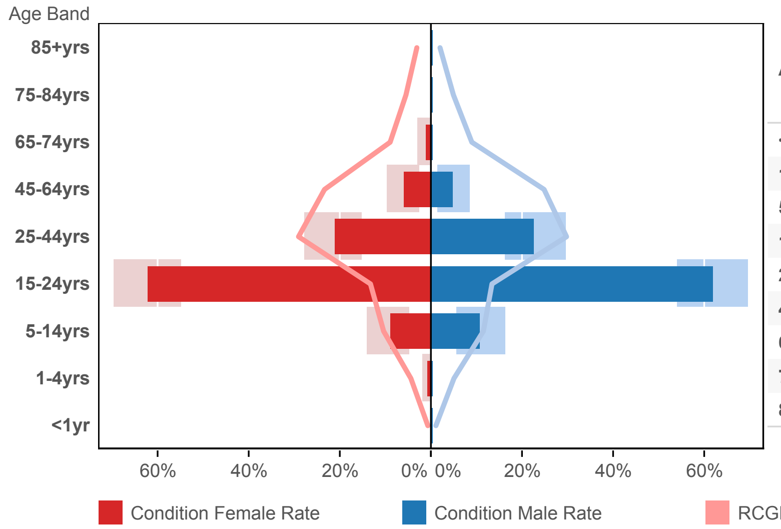

Age Band	Condition Female Rate	Condition Male Rate	Condition Female Population	Condition Male Population
<1yr	0.00	0.00	0.0	0.0
1-4yrs	0.60	0.00	1.0	0.0
5-14yrs	9.04	10.64	15.0	15.0
15-24yrs	62.05	61.70	103.0	87.0
25-44yrs	21.08	22.70	35.0	32.0
45-64yrs	6.02	4.96	10.0	7.0
65-74yrs	1.20	0.00	2.0	0.0
75-84yrs	0.00	0.00	0.0	0.0
85+yrs	0.00	0.00	0.0	0.0

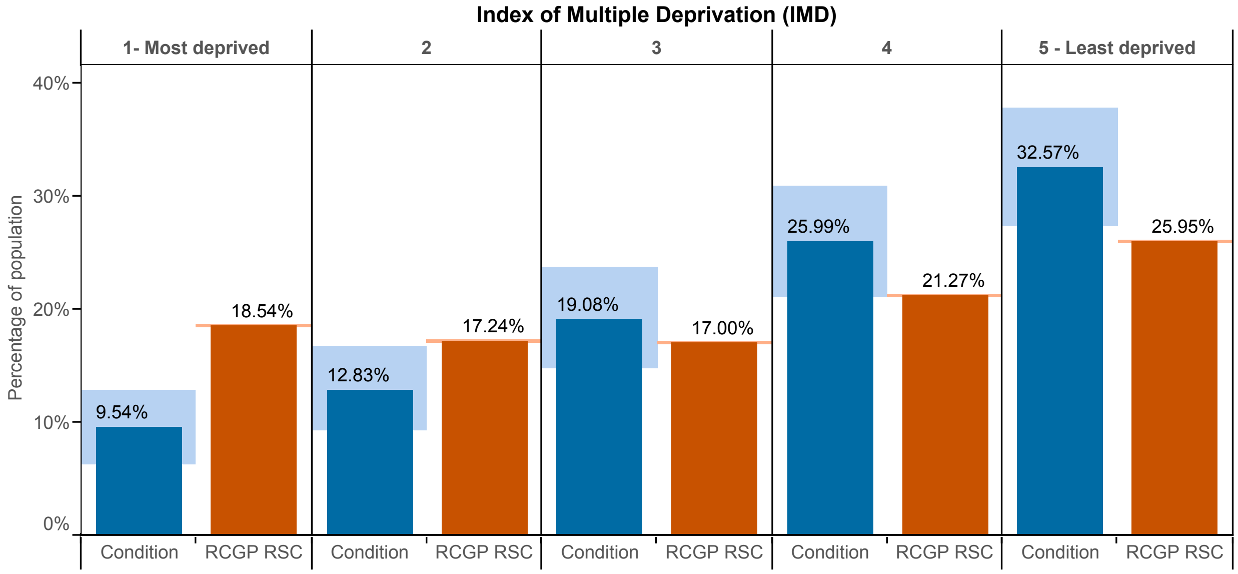

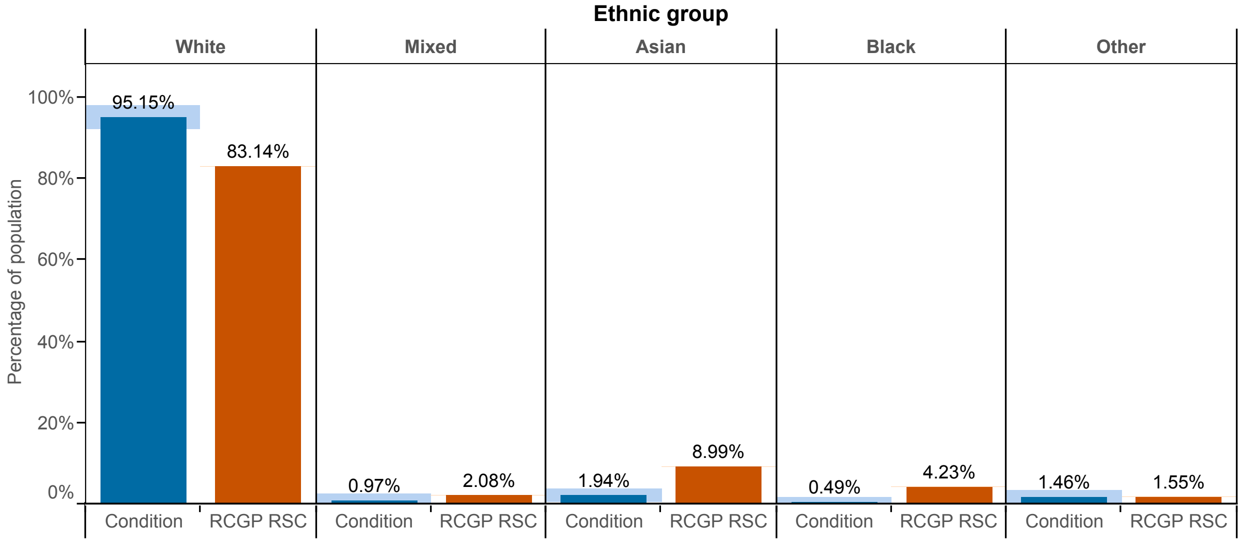

Influenza-like illness (ICD10 : J09 - J11)

Age-sex profile

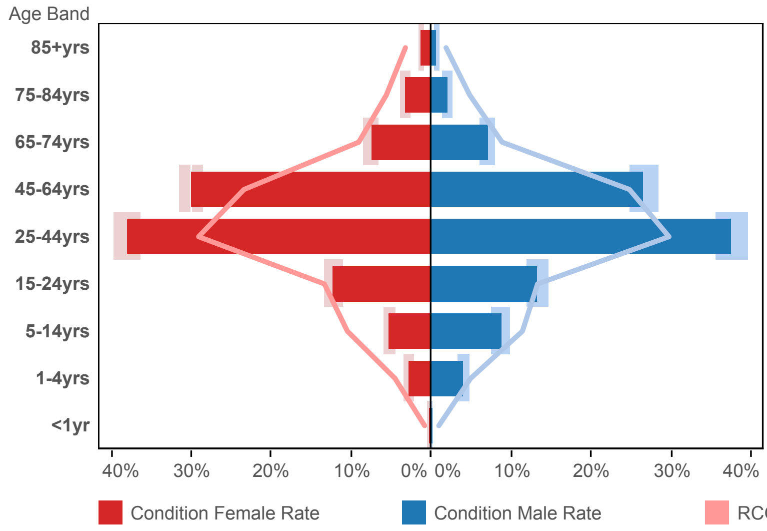

Age Band	Condition Female Rate	Condition Male Rate	Condition Female Population	Condition Male Population
<1yr	0.19	0.12	6	3
1-4yrs	2.75	4.06	89	98
5-14yrs	5.21	8.74	169	211
15-24yrs	12.15	13.33	394	322
25-44yrs	37.94	37.47	1,230	905
45-64yrs	29.98	26.50	972	640
65-74yrs	7.43	7.04	241	170
75-84yrs	3.18	2.11	103	51
85+yrs	1.17	0.62	38	15

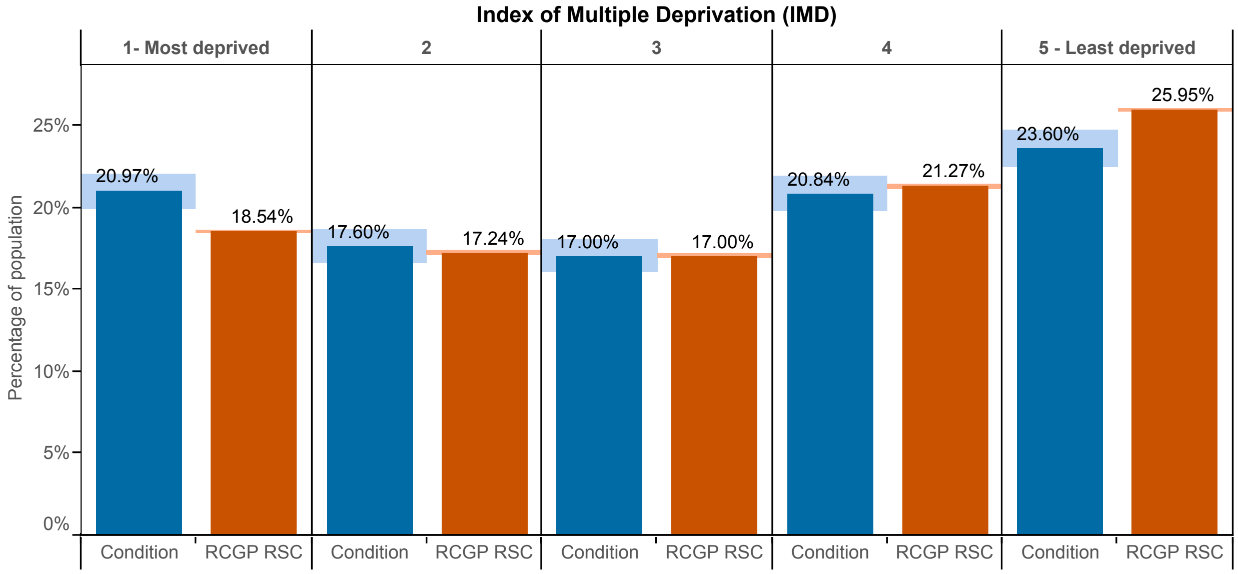

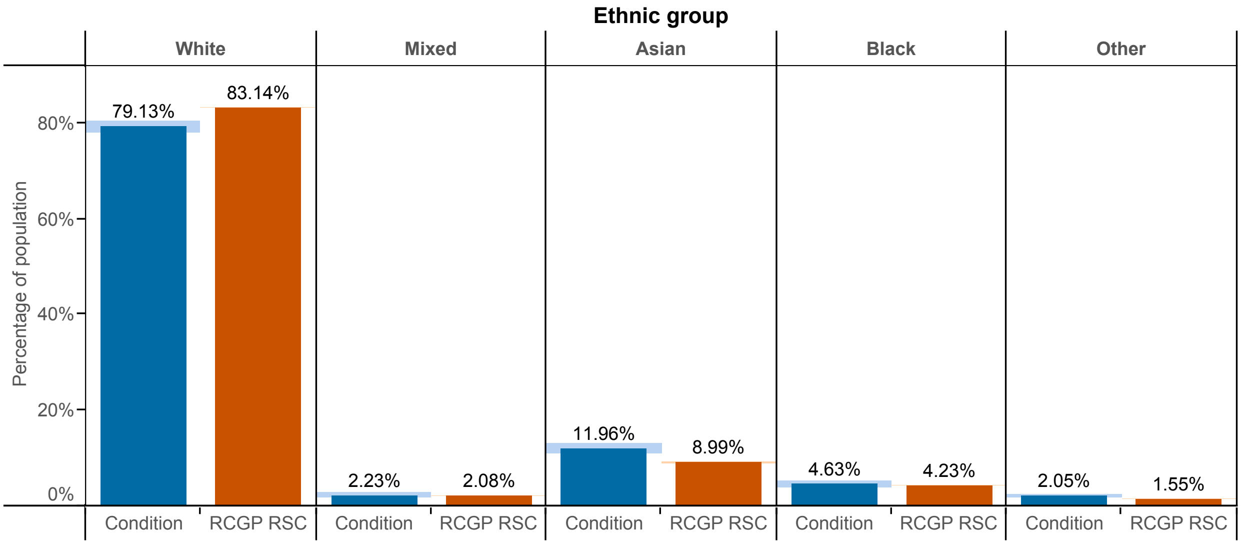

Intestinal Infections (ICD10 : A00-A09)

Age-sex profile

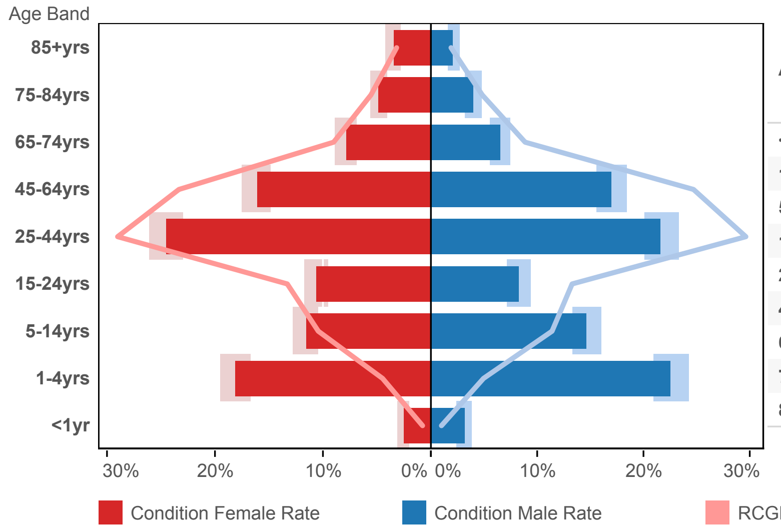

Age Band	Condition Female Rate	Condition Male Rate	Condition Female P opulation	Condition Male Pop ulation
<1yr	2.57	3.14	74.0	78.0
1-4yrs	18.21	22.64	525.0	563.0
5-14yrs	11.62	14.68	335.0	365.0
15-24yrs	10.61	8.24	306.0	205.0
25-44yrs	24.59	21.67	709.0	539.0
45-64yrs	16.13	17.01	465.0	423.0
65-74yrs	7.87	6.51	227.0	162.0
75-84yrs	4.89	3.98	141.0	99.0
85+yrs	3.50	2.13	101.0	53.0

Index of Multiple Deprivation (IMD)

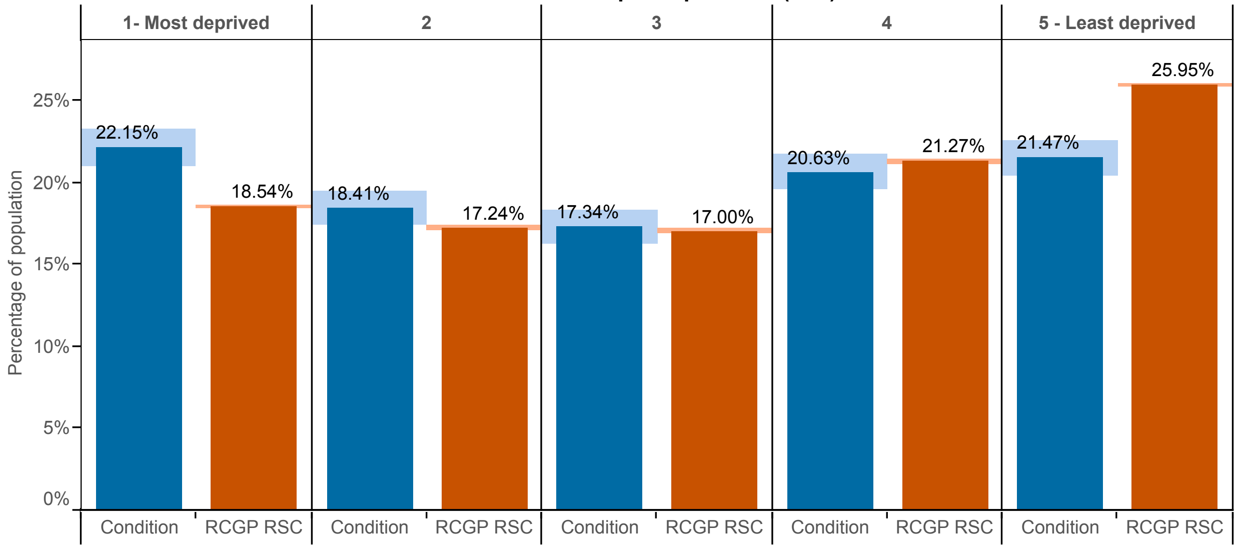

Ethnic group

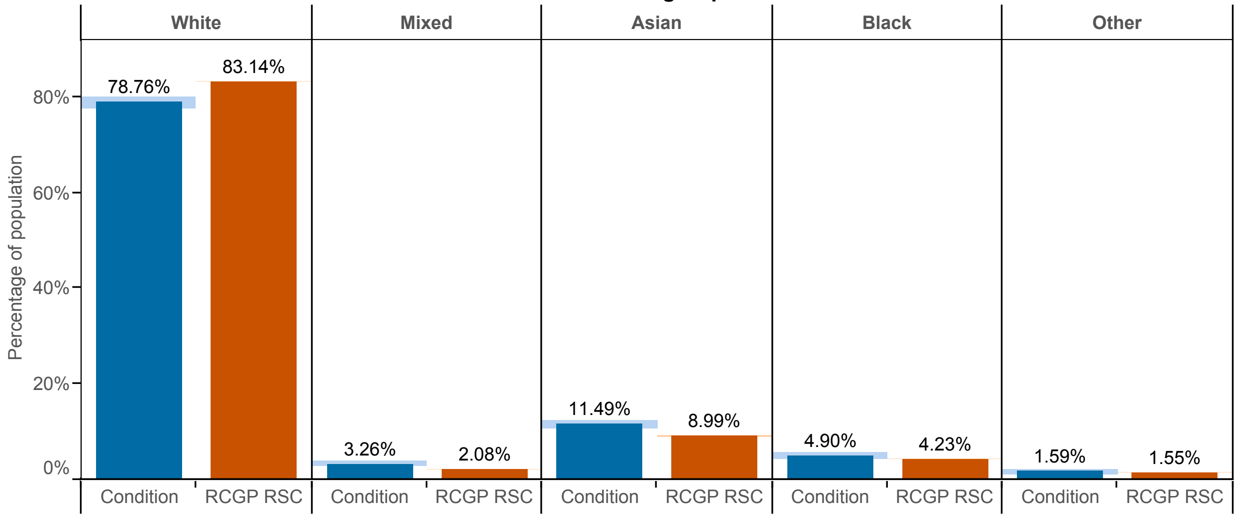

Laryngitis / Tracheitis (ICD10 : J04)

Age-sex profile

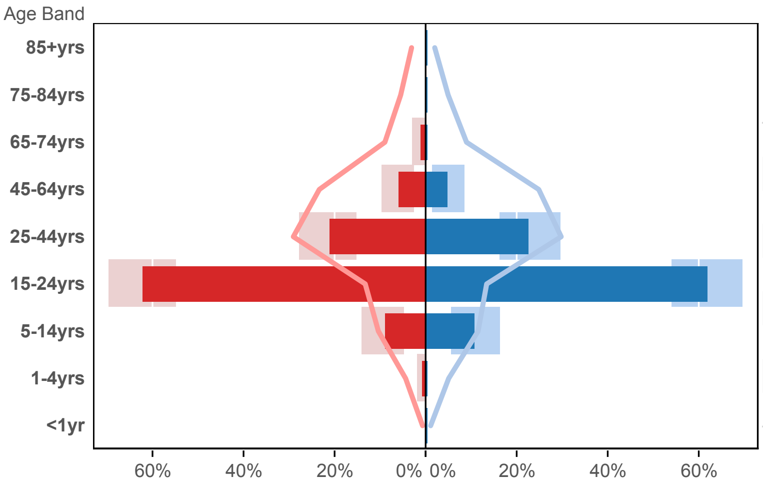

Age Band	Condition Female Rate	Condition Male Rate	Condition Female Population	Condition Male Population
<1yr	0.00	0.00	0.0	0.0
1-4yrs	0.60	0.00	1.0	0.0
5-14yrs	9.04	10.64	15.0	15.0
15-24yrs	62.05	61.70	103.0	87.0
25-44yrs	21.08	22.70	35.0	32.0
45-64yrs	6.02	4.96	10.0	7.0
65-74yrs	1.20	0.00	2.0	0.0
75-84yrs	0.00	0.00	0.0	0.0
85+yrs	0.00	0.00	0.0	0.0

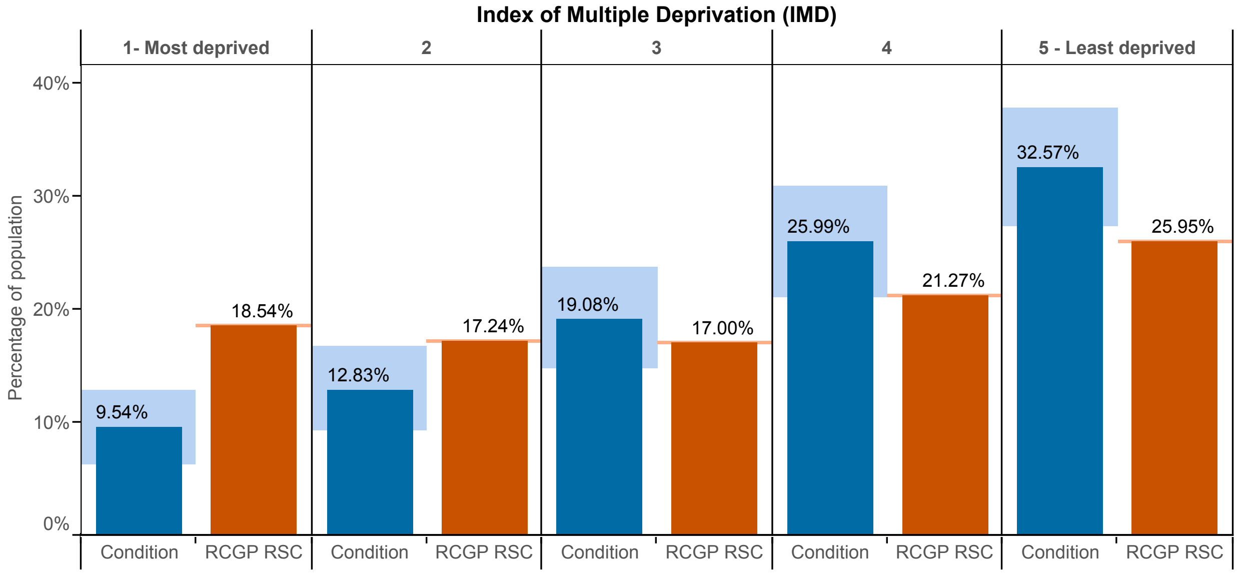

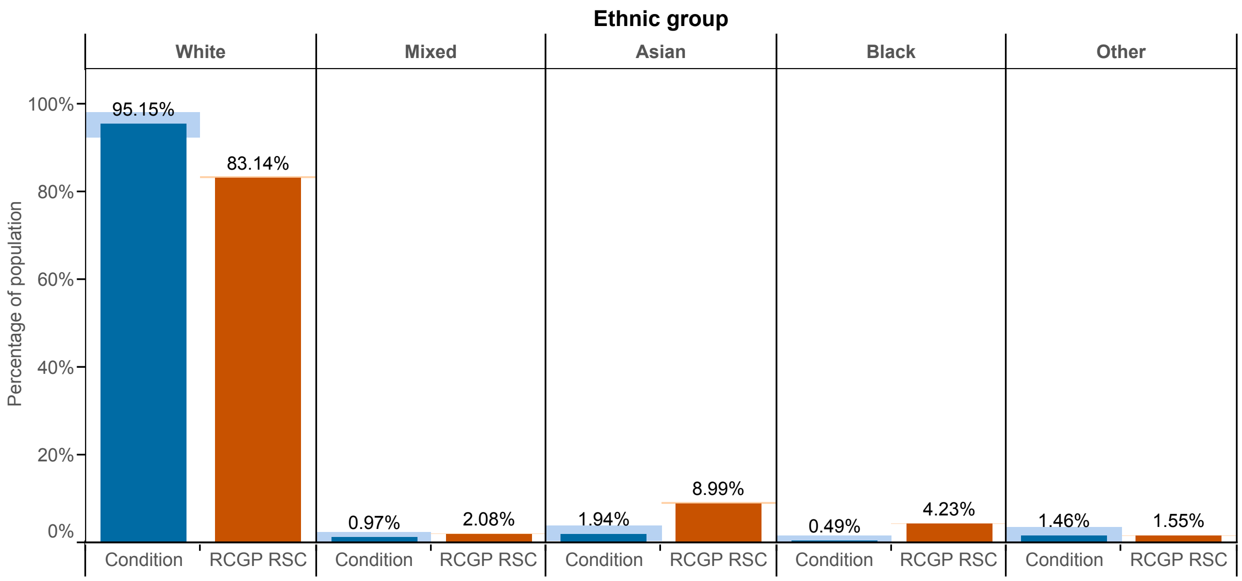

Lower Respiratory Tract Infections (ICD10 : J20-J22)

Age-sex profile

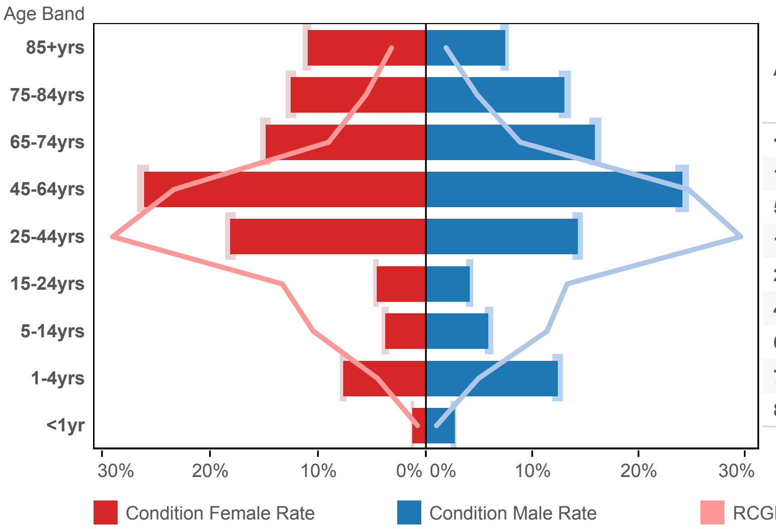

Age Band	Condition Female Rate	Condition Male Rate	Condition Female Population	Condition Male Population
<1yr	1.23	2.63	273	451
1-4yrs	7.67	12.37	1,707	2,123
5-14yrs	3.77	5.95	838	1,021
15-24yrs	4.58	4.14	1,019	711
25-44yrs	18.15	14.31	4,038	2,456
45-64yrs	26.22	24.14	5,832	4,144
65-74yrs	14.88	15.92	3,309	2,733
75-84yrs	12.48	13.09	2,777	2,247
85+yrs	11.02	7.46	2,452	1,281

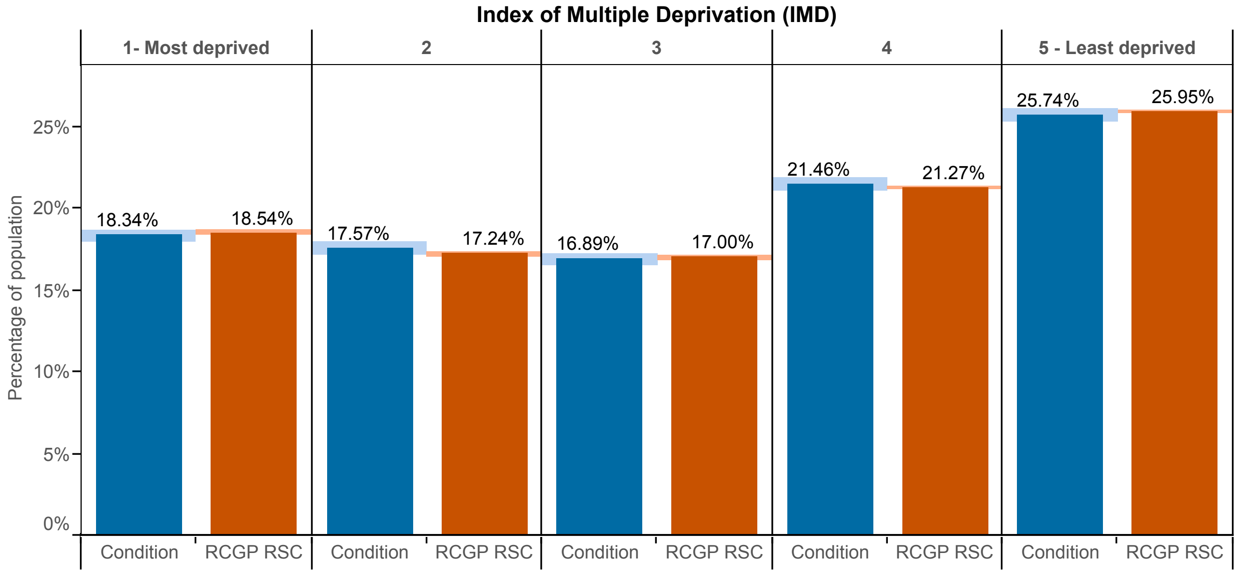

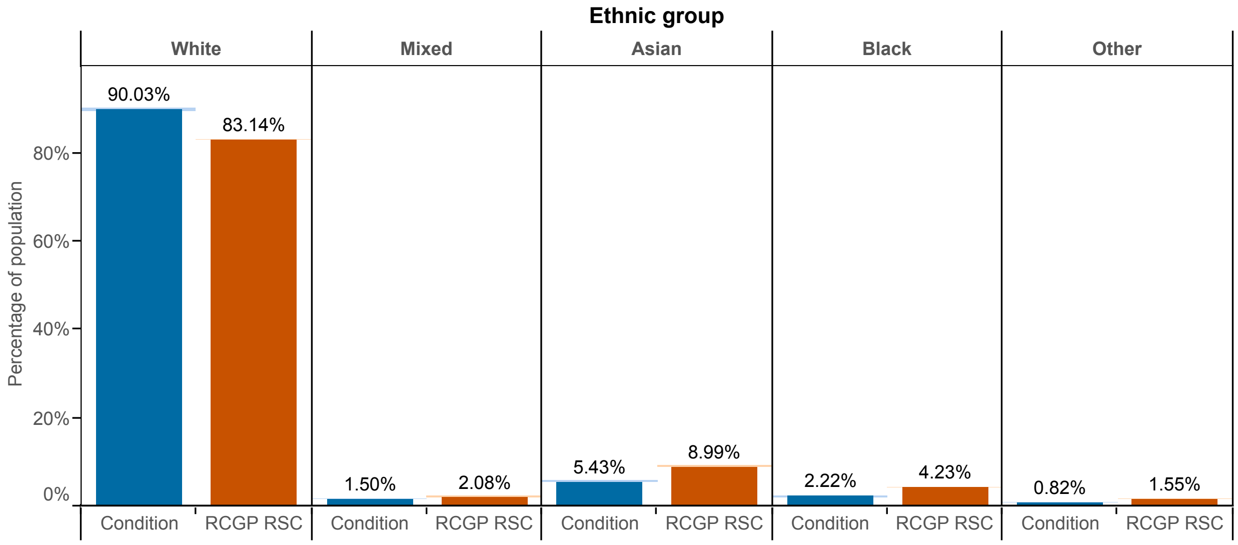

Measles (ICD10 : B05)

Age-sex profile

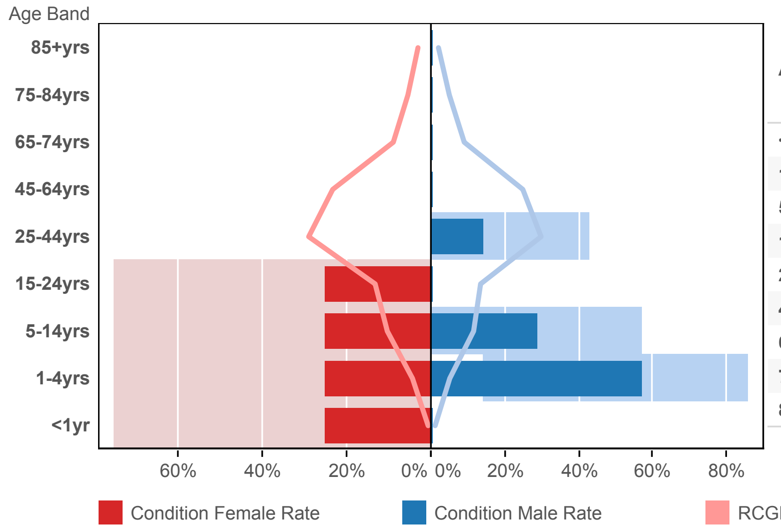

Age Band	Condition Female Rate	Condition Male Rate	Condition Female P opulation	Condition Male Pop ulation
<1yr	25.00	0.00	1.00	0.00
1-4yrs	25.00	57.14	1.00	4.00
5-14yrs	25.00	28.57	1.00	2.00
15-24yrs	25.00	0.00	1.00	0.00
25-44yrs	0.00	14.29	0.00	1.00
45-64yrs	0.00	0.00	0.00	0.00
65-74yrs	0.00	0.00	0.00	0.00
75-84yrs	0.00	0.00	0.00	0.00
85+yrs	0.00	0.00	0.00	0.00

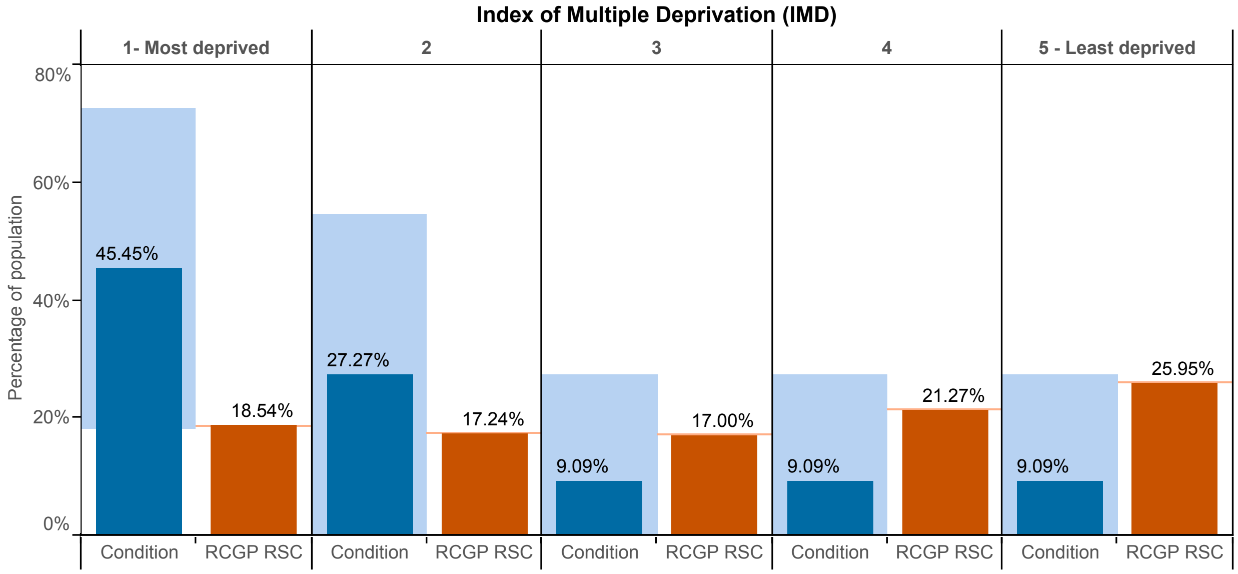

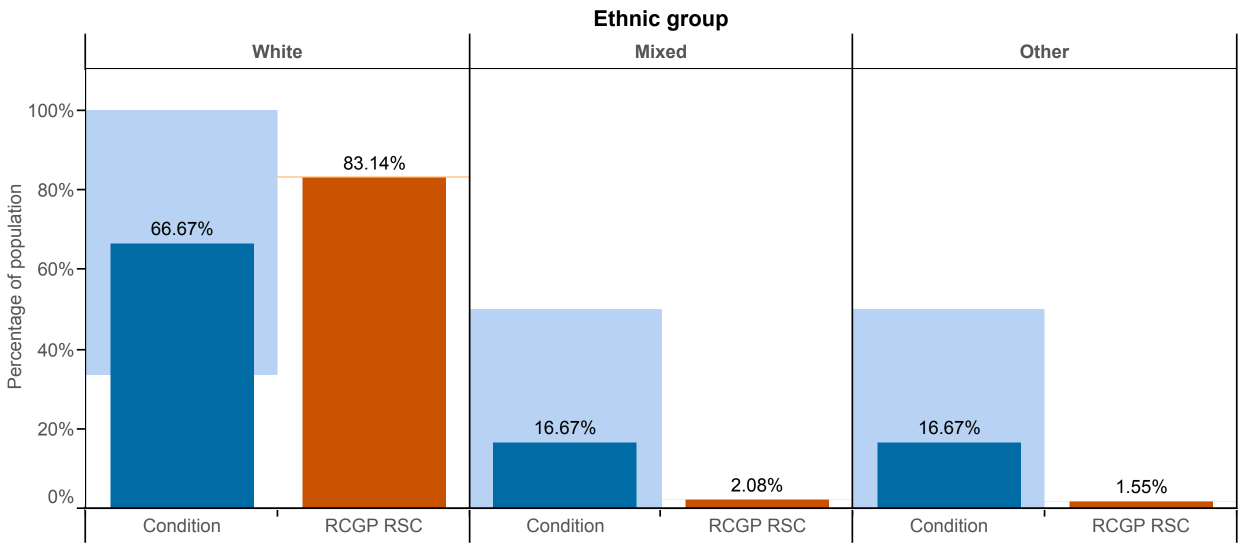

Meningitis and Encephalitis (ICD10 : A170 - A171; A 390; A83 - A85; A87; G00 - G05)

Age-sex profile

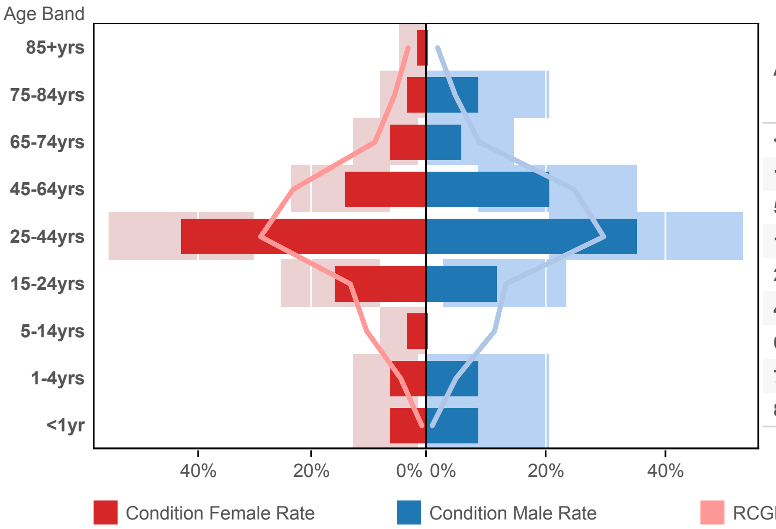

Age Band	Condition Female Rate	Condition Male Rate	Condition Female Population	Condition Male Population
<1yr	6.35	8.82	4.00	3.00
1-4yrs	6.35	8.82	4.00	3.00
5-14yrs	3.17	0.00	2.00	0.00
15-24yrs	15.87	11.76	10.00	4.00
25-44yrs	42.86	35.29	27.00	12.00
45-64yrs	14.29	20.59	9.00	7.00
65-74yrs	6.35	5.88	4.00	2.00
75-84yrs	3.17	8.82	2.00	3.00
85+yrs	1.59	0.00	1.00	0.00

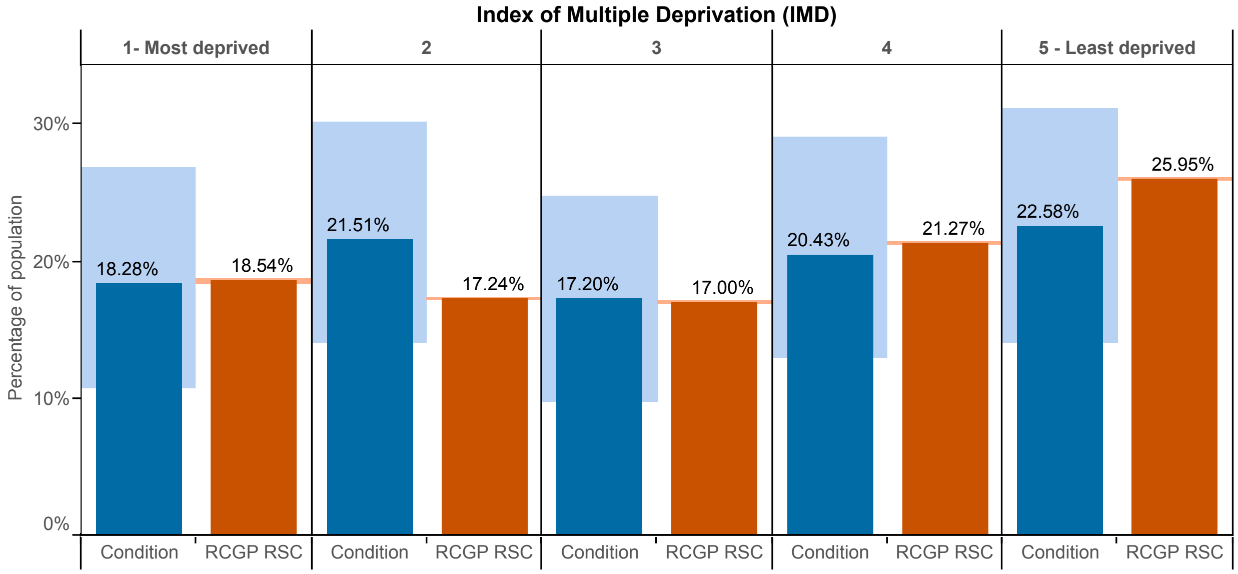

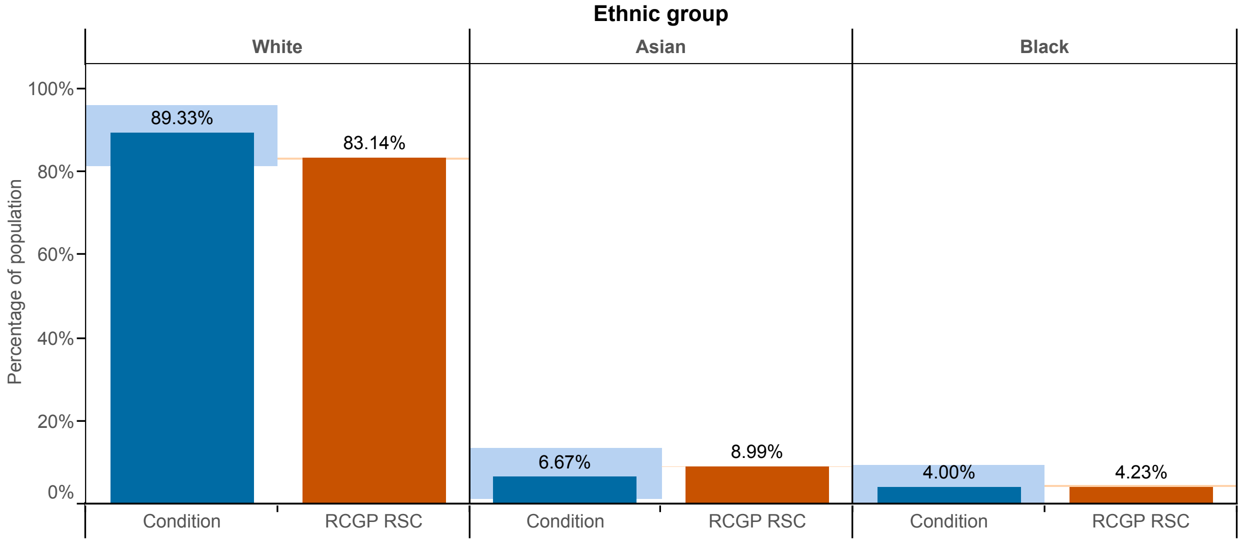

Mumps (ICD10 : B26)

Age-sex profile

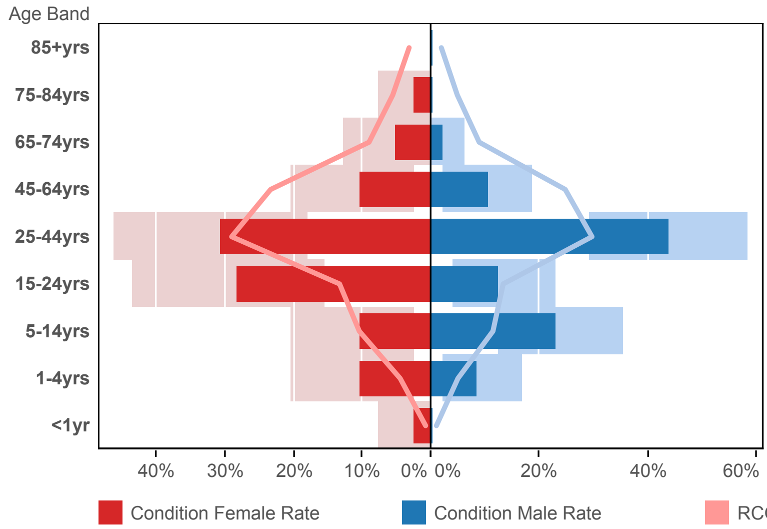

Age Band	Condition Female Rate	Condition Male Rate	Condition Female Population	Condition Male Population
<1yr	2.56	0.00	1.00	0.00
1-4yrs	10.26	8.33	4.00	4.00
5-14yrs	10.26	22.92	4.00	11.00
15-24yrs	28.21	12.50	11.00	6.00
25-44yrs	30.77	43.75	12.00	21.00
45-64yrs	10.26	10.42	4.00	5.00
65-74yrs	5.13	2.08	2.00	1.00
75-84yrs	2.56	0.00	1.00	0.00
85+yrs	0.00	0.00	0.00	0.00

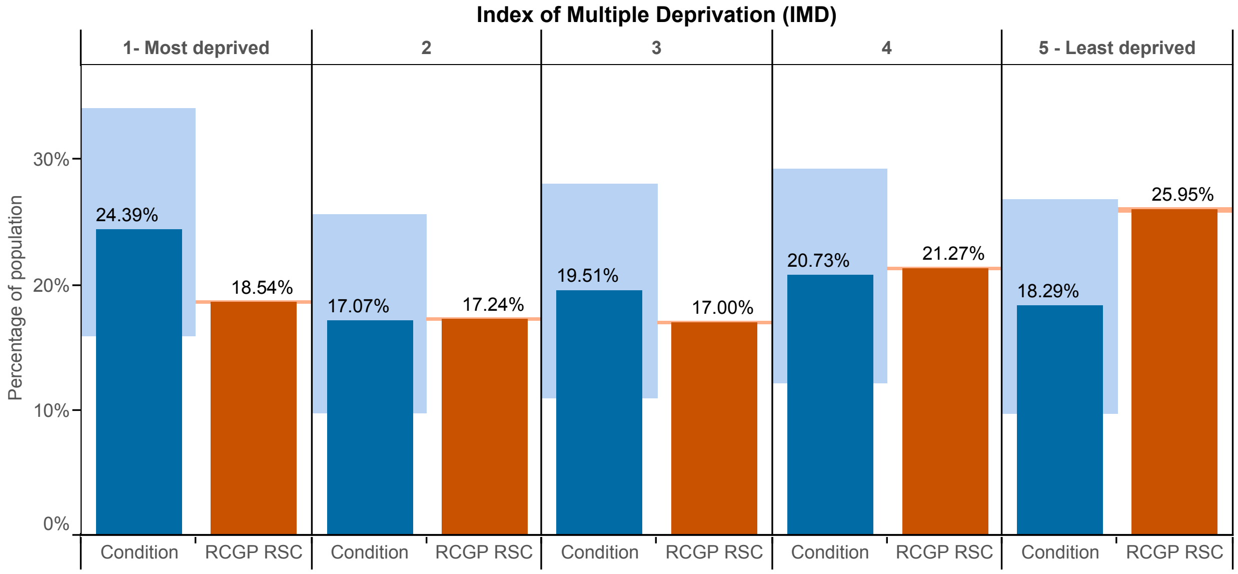

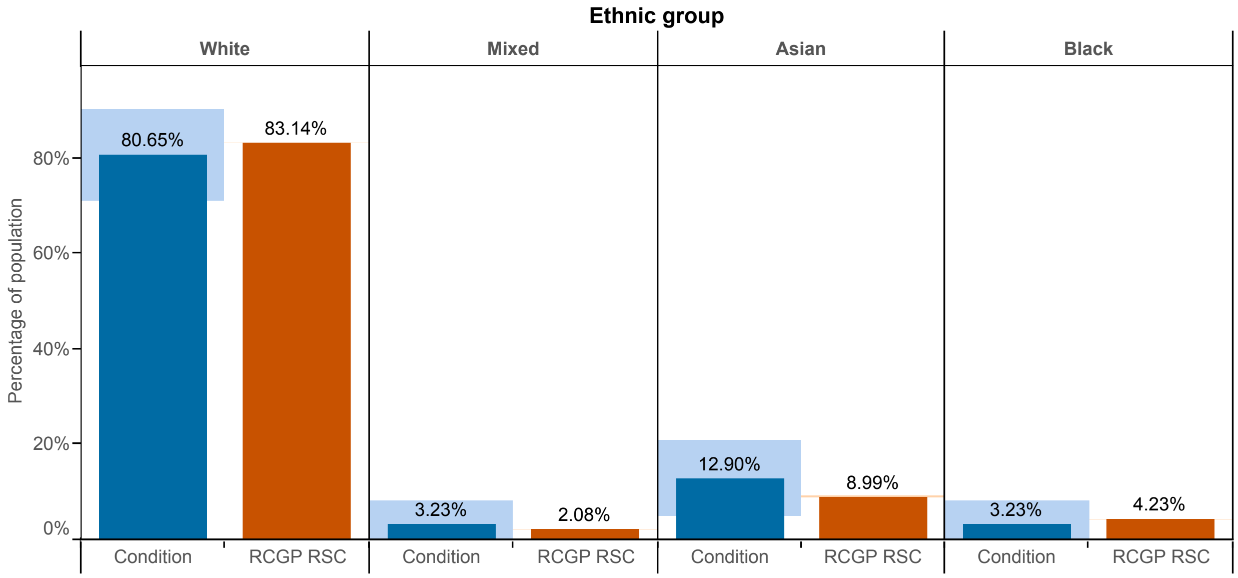

Non-infective Enteritis / Colitis (ICD10 : K50-K52)

Age-sex profile

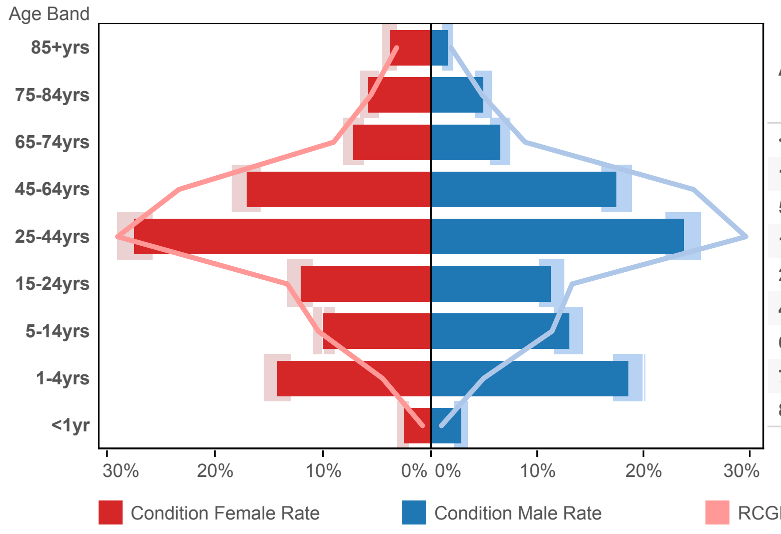

Age Band	Condition Female Rate	Condition Male Rate	Condition Female Population	Condition Male Population
<1yr	2.51	2.86	74.0	70.0
1-4yrs	14.19	18.62	419.0	455.0
5-14yrs	9.96	12.97	294.0	317.0
15-24yrs	12.06	11.33	356.0	277.0
25-44yrs	27.50	23.77	812.0	581.0
45-64yrs	17.14	17.47	506.0	427.0
65-74yrs	7.15	6.46	211.0	158.0
75-84yrs	5.72	4.91	169.0	120.0
85+yrs	3.79	1.60	112.0	39.0

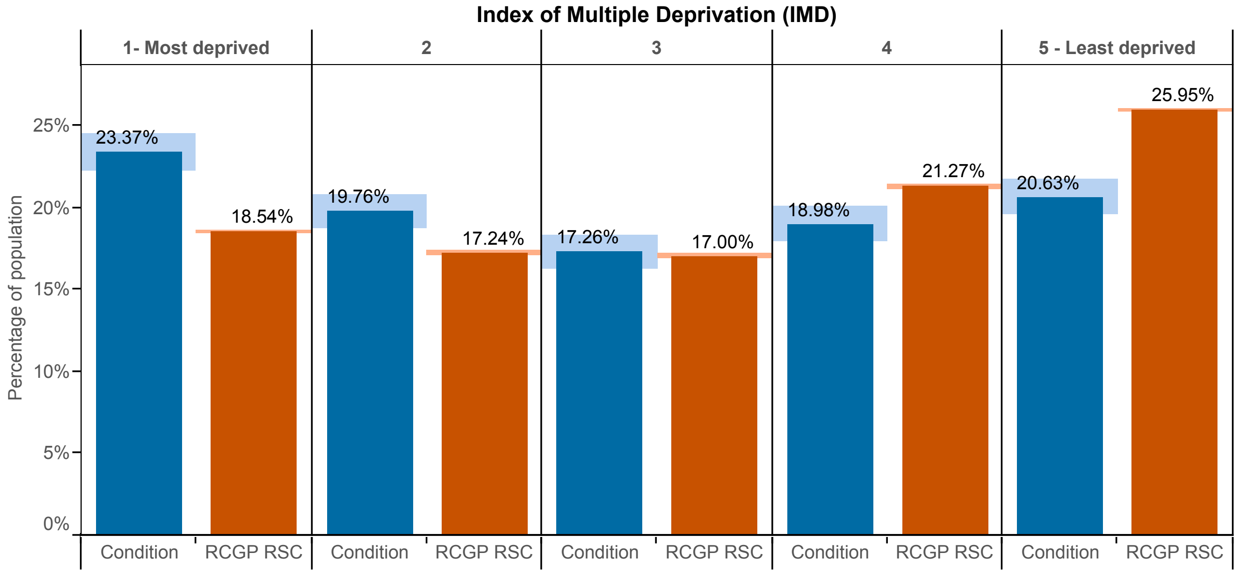

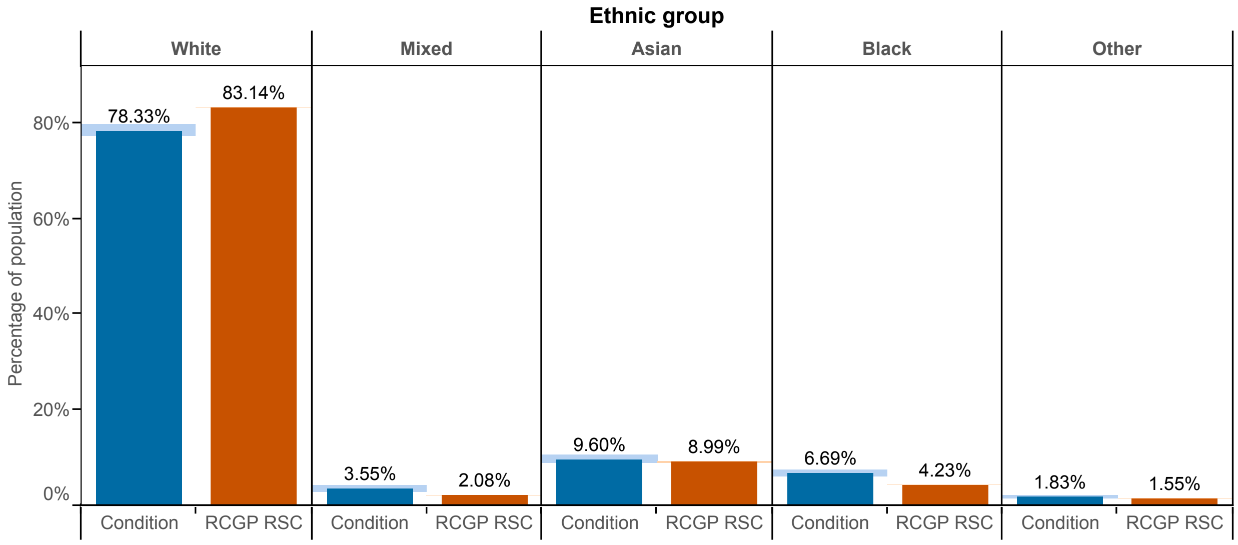

Acute Otitis Media (ICD10 : H650 - H651; H660; H669)

Age-sex profile

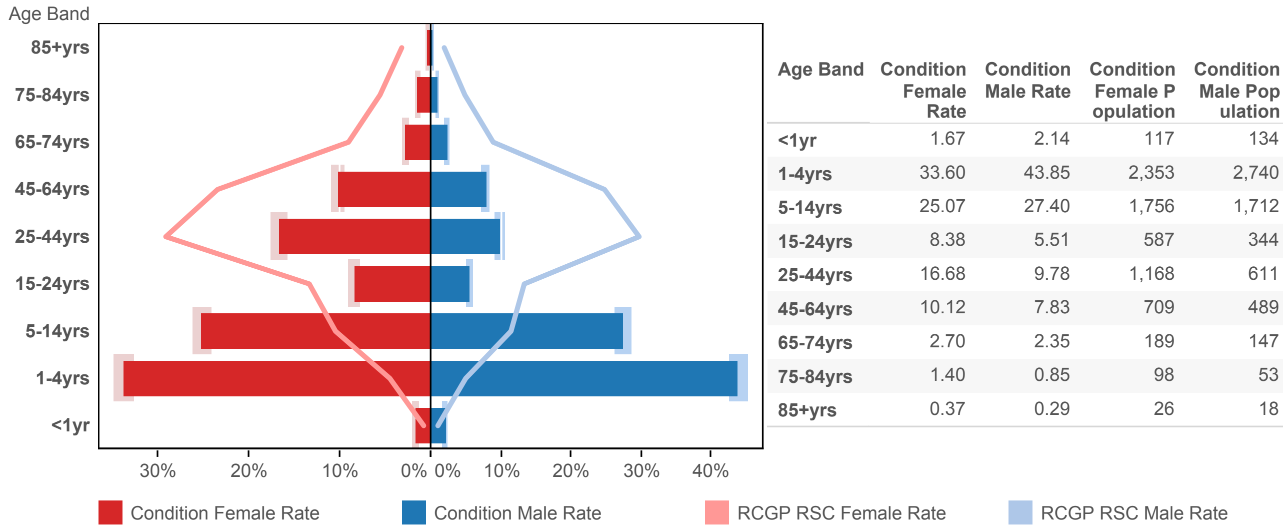

Age Band	Condition Female Rate	Condition Male Rate	Condition Female Population	Condition Male Population
<1yr	1.67	2.14	117	134
1-4yrs	33.60	43.85	2,353	2,740
5-14yrs	25.07	27.40	1,756	1,712
15-24yrs	8.38	5.51	587	344
25-44yrs	16.68	9.78	1,168	611
45-64yrs	10.12	7.83	709	489
65-74yrs	2.70	2.35	189	147
75-84yrs	1.40	0.85	98	53
85+yrs	0.37	0.29	26	18

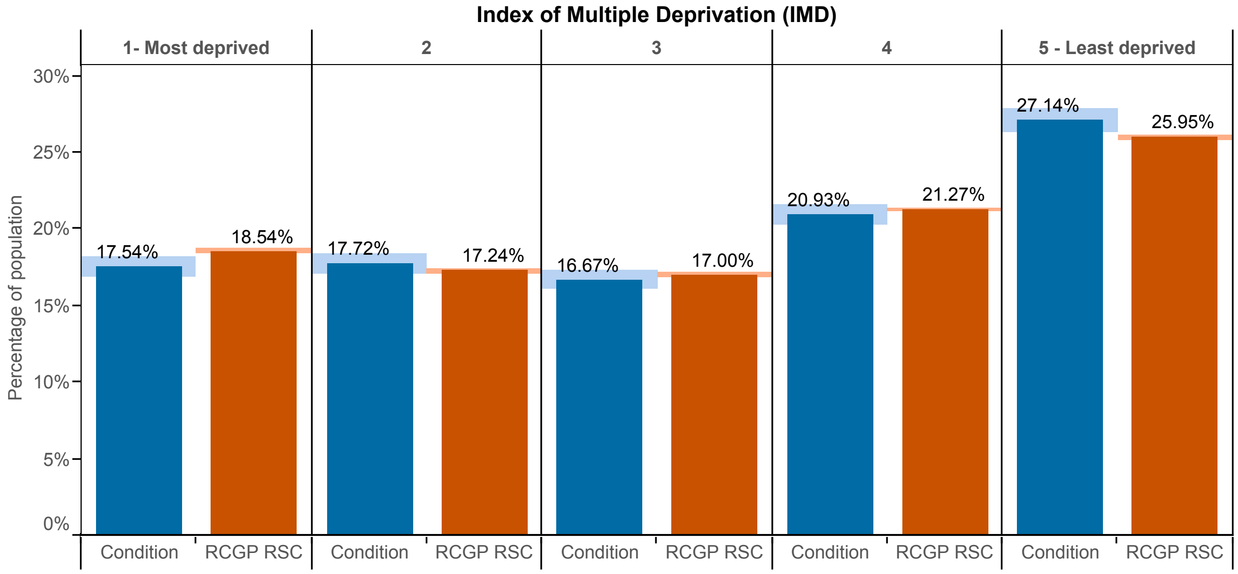

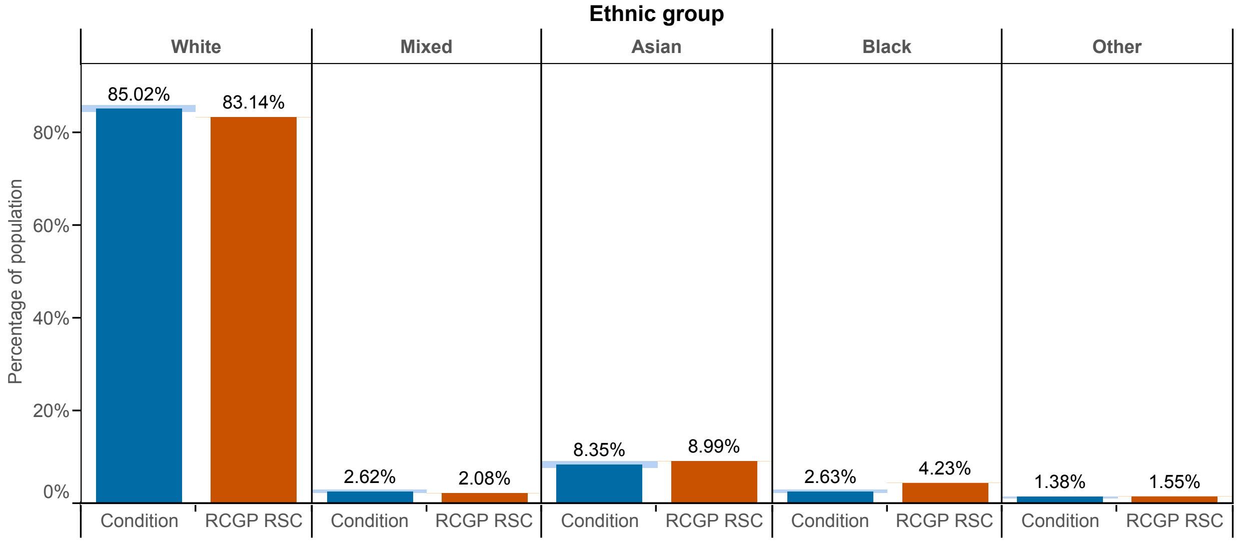

Peripheral Neuropathy (ICD10 : G50 - G64; G70 - G72)

Age-sex profile

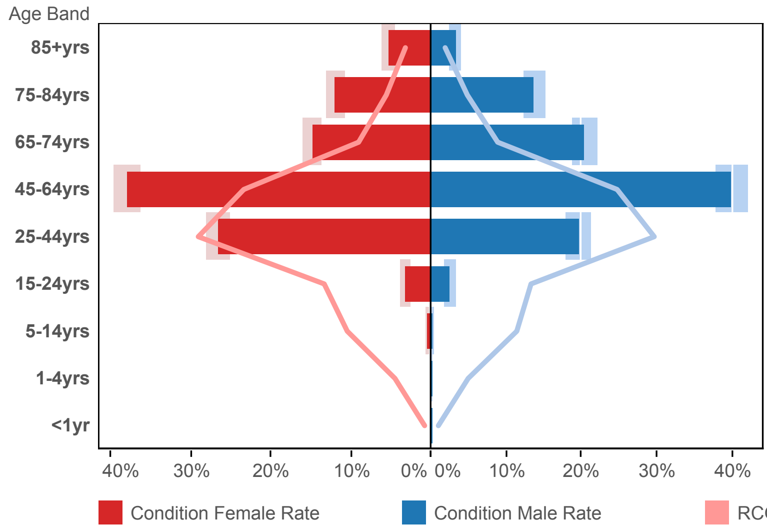

Age Band	Condition Female Rate	Condition Male Rate	Condition Female Population	Condition Male Population
<1yr	0.00	0.00	0	0
1-4yrs	0.00	0.14	0	3
5-14yrs	0.41	0.24	13	5
15-24yrs	3.12	2.56	99	54
25-44yrs	26.57	19.64	843	414
45-64yrs	37.85	39.94	1,201	842
65-74yrs	14.84	20.49	471	432
75-84yrs	11.94	13.71	379	289
85+yrs	5.26	3.27	167	69

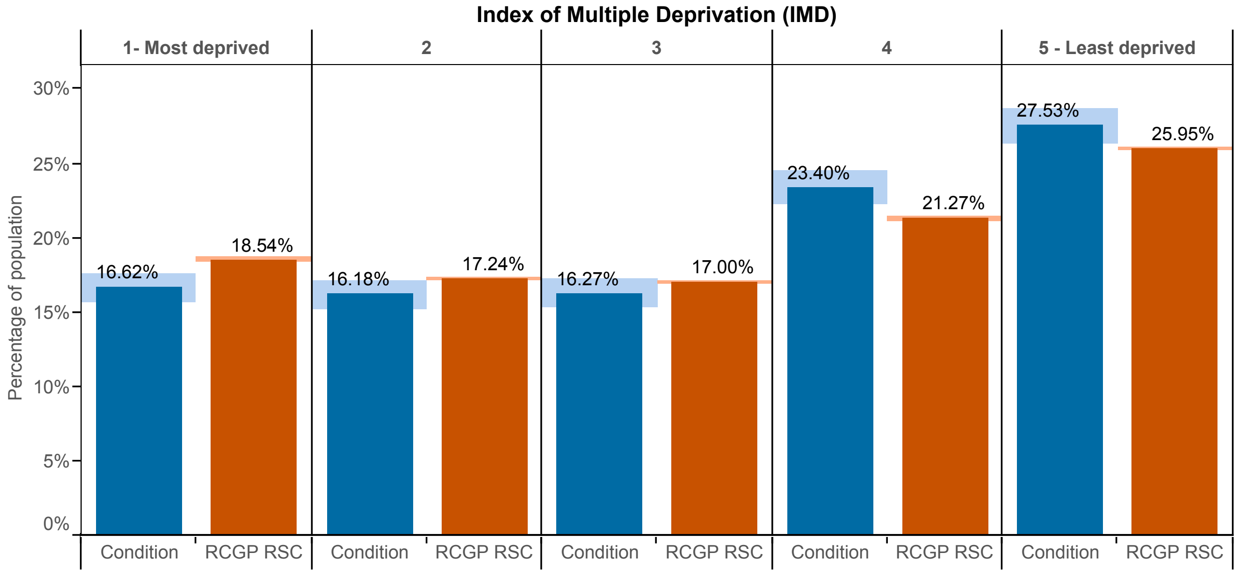

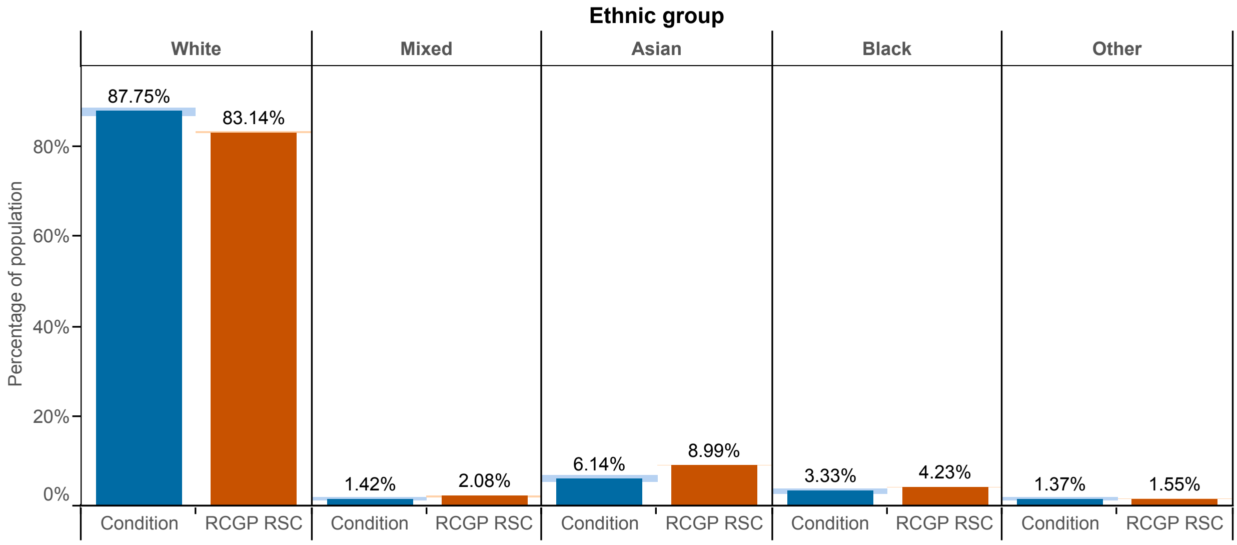

Pleurisy (ICD10 : R091)

Age-sex profile

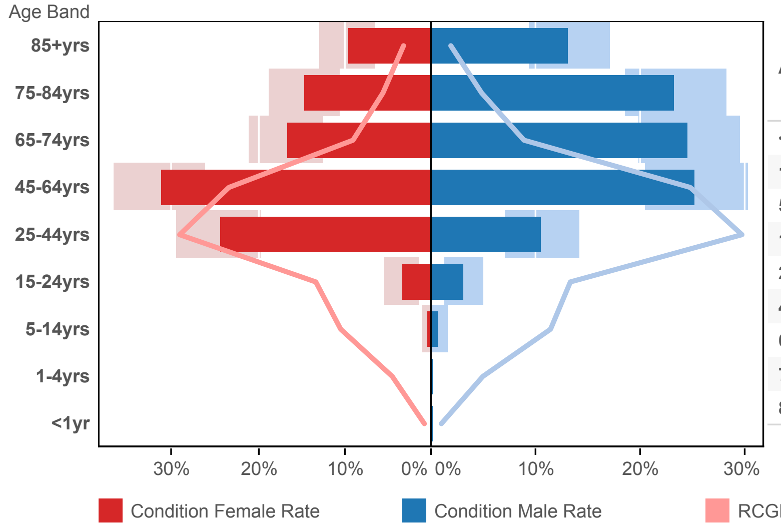

Age Band	Condition Female Rate	Condition Male Rate	Condition Female Population	Condition Male Population
<1yr	0.00	0.00	0.00	0.00
1-4yrs	0.00	0.00	0.00	0.00
5-14yrs	0.34	0.67	1.00	2.00
15-24yrs	3.39	3.02	10.00	9.00
25-44yrs	24.41	10.40	72.00	31.00
45-64yrs	31.19	25.17	92.00	75.00
65-74yrs	16.61	24.50	49.00	73.00
75-84yrs	14.58	23.15	43.00	69.00
85+yrs	9.49	13.09	28.00	39.00

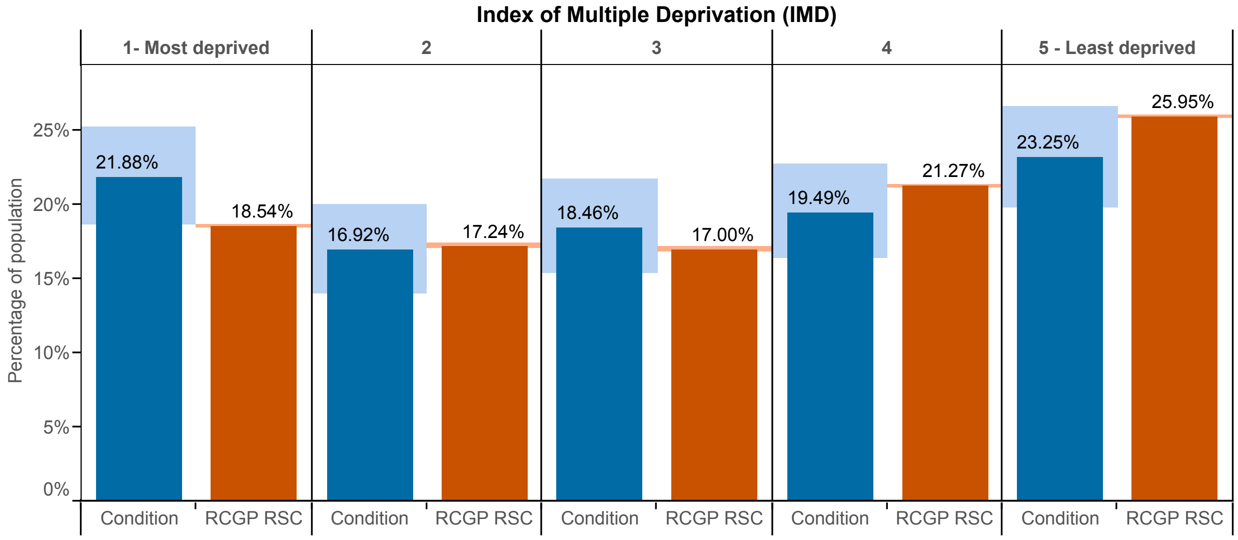

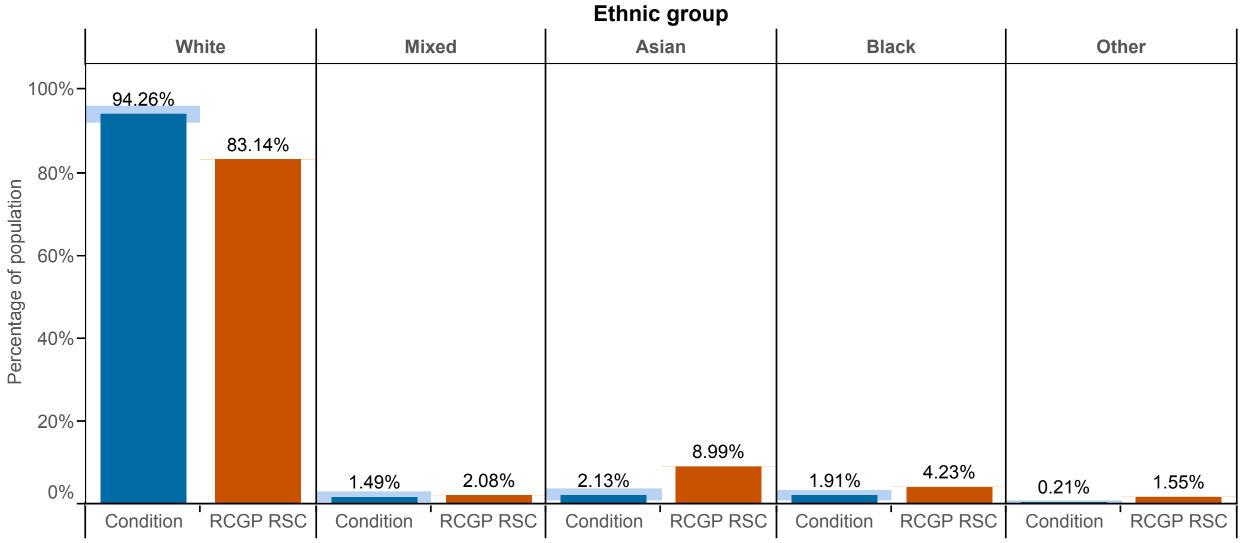

Pneumonia / Pneumonitis (ICD10 : J12 - J18)

Age-sex profile

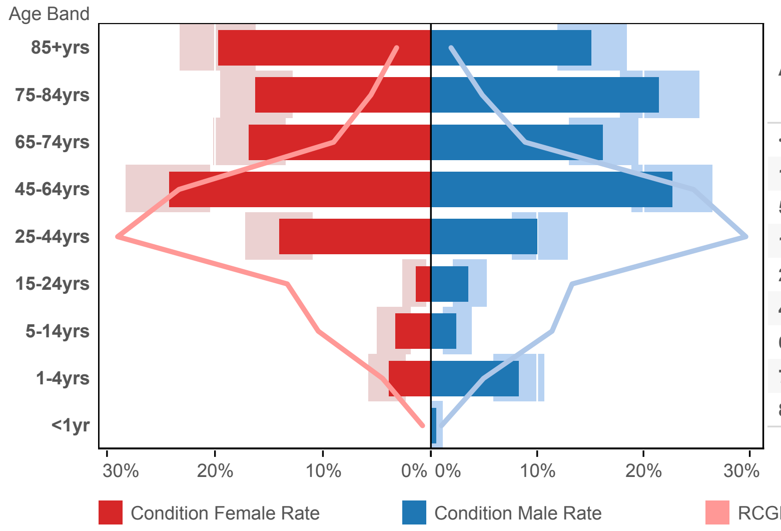

Age Band	Condition Female Rate	Condition Male Rate	Condition Female Population	Condition Male Population
<1yr	0.00	0.42	0.0	2.0
1-4yrs	3.95	8.19	19.0	39.0
5-14yrs	3.33	2.31	16.0	11.0
15-24yrs	1.46	3.57	7.0	17.0
25-44yrs	14.14	10.08	68.0	48.0
45-64yrs	24.32	22.69	117.0	108.0
65-74yrs	16.84	16.18	81.0	77.0
75-84yrs	16.22	21.43	78.0	102.0
85+yrs	19.75	15.13	95.0	72.0

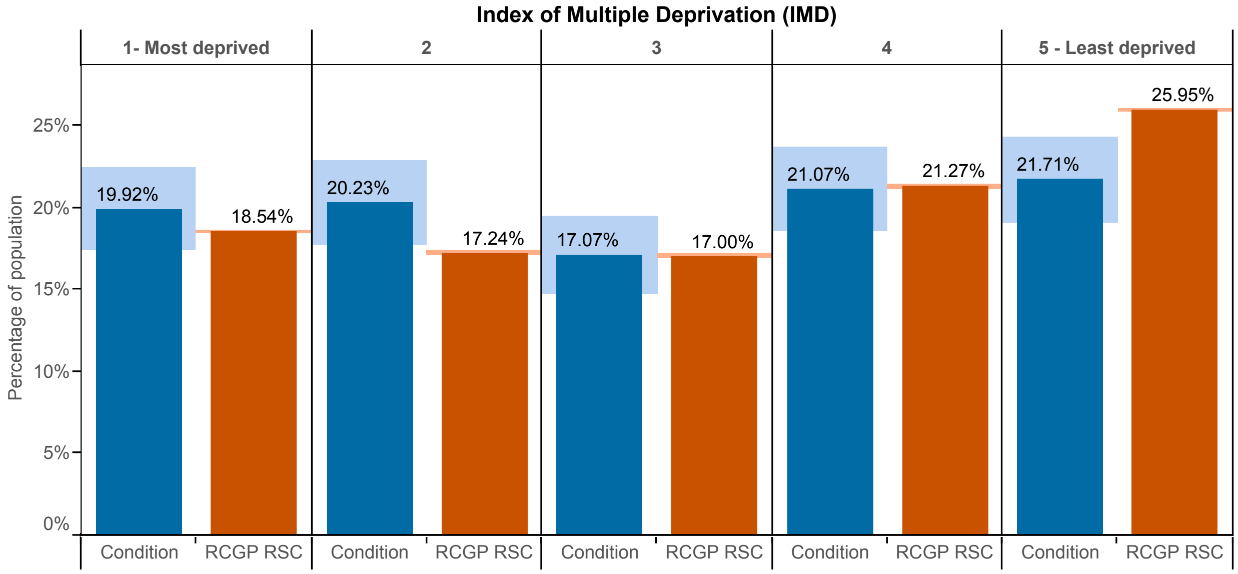

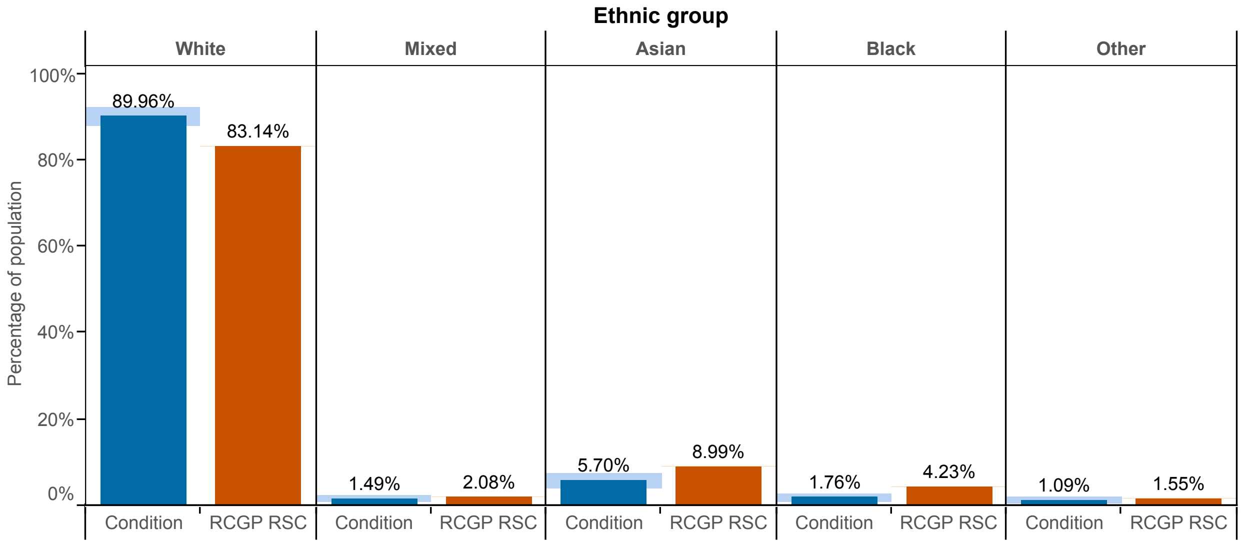

Respiratory disorders (ICD10 : J00-J99)

Age-sex profile

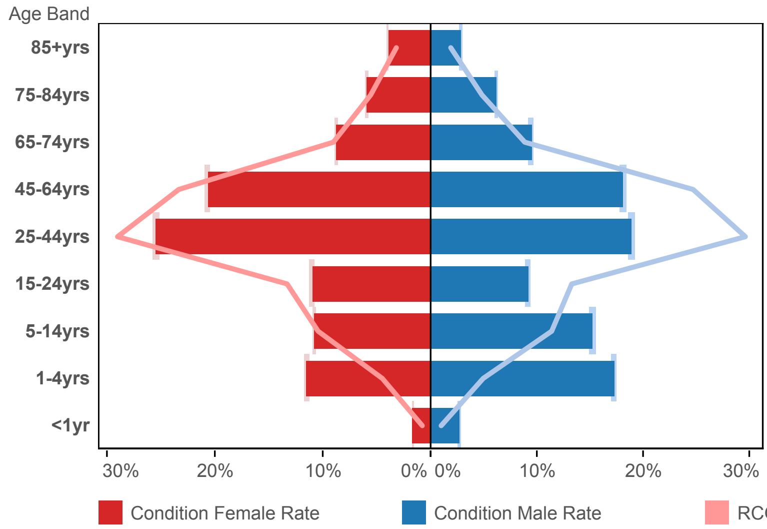

Age Band	Condition Female Rate	Condition Male Rate	Condition Female Population	Condition Male Population
<1yr	1.65	2.71	1,458	1,751
1-4yrs	11.53	17.27	10,164	11,139
5-14yrs	10.81	15.28	9,522	9,858
15-24yrs	11.01	9.19	9,699	5,926
25-44yrs	25.55	18.86	22,516	12,167
45-64yrs	20.75	18.07	18,282	11,654
65-74yrs	8.80	9.51	7,758	6,135
75-84yrs	5.93	6.23	5,226	4,018
85+yrs	3.97	2.88	3,498	1,856

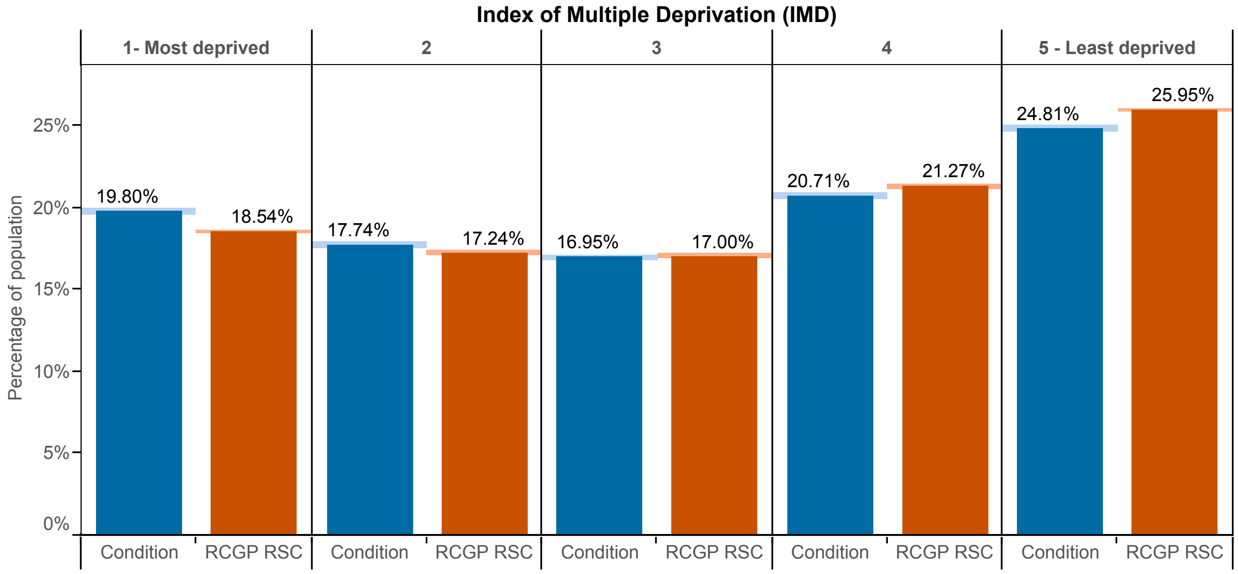

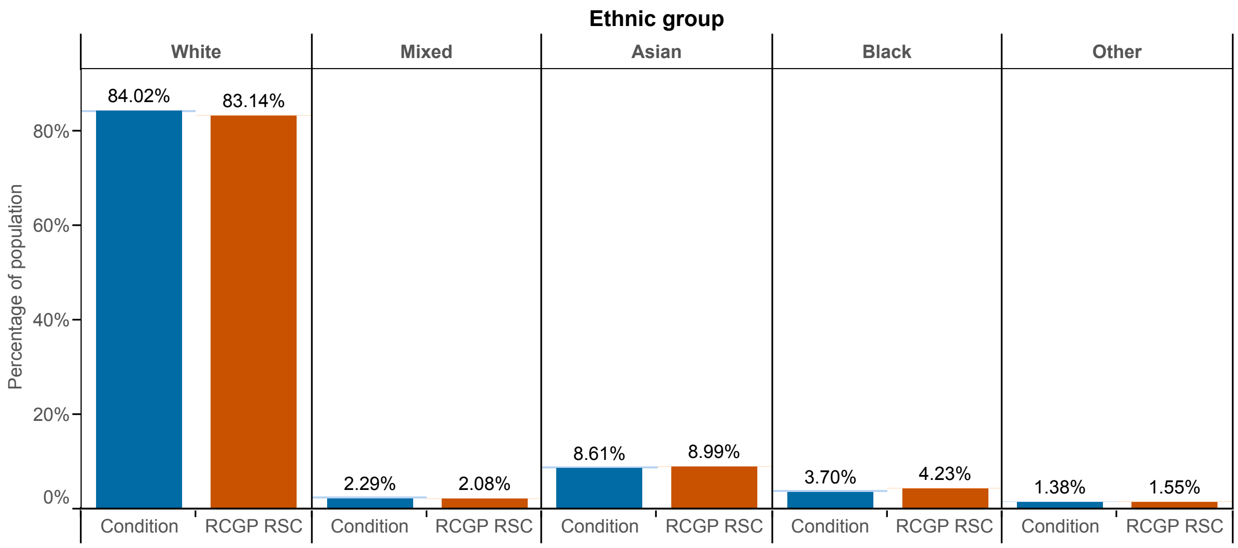

Rubella (ICD10 : B06)

Age-sex profile

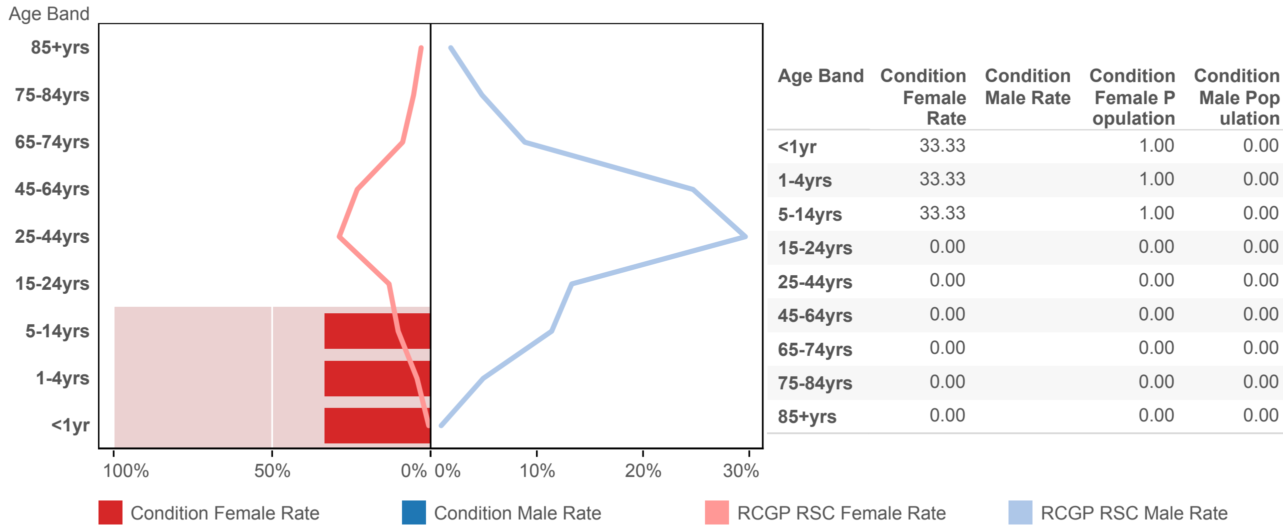

Age Band	Condition Female Rate	Condition Male Rate	Condition Female P opulation	Condition Male Pop ulation
<1yr	33.33		1.00	0.00
1-4yrs	33.33		1.00	0.00
5-14yrs	33.33		1.00	0.00
15-24yrs	0.00		0.00	0.00
25-44yrs	0.00		0.00	0.00
45-64yrs	0.00		0.00	0.00
65-74yrs	0.00		0.00	0.00
75-84yrs	0.00		0.00	0.00
85+yrs	0.00		0.00	0.00

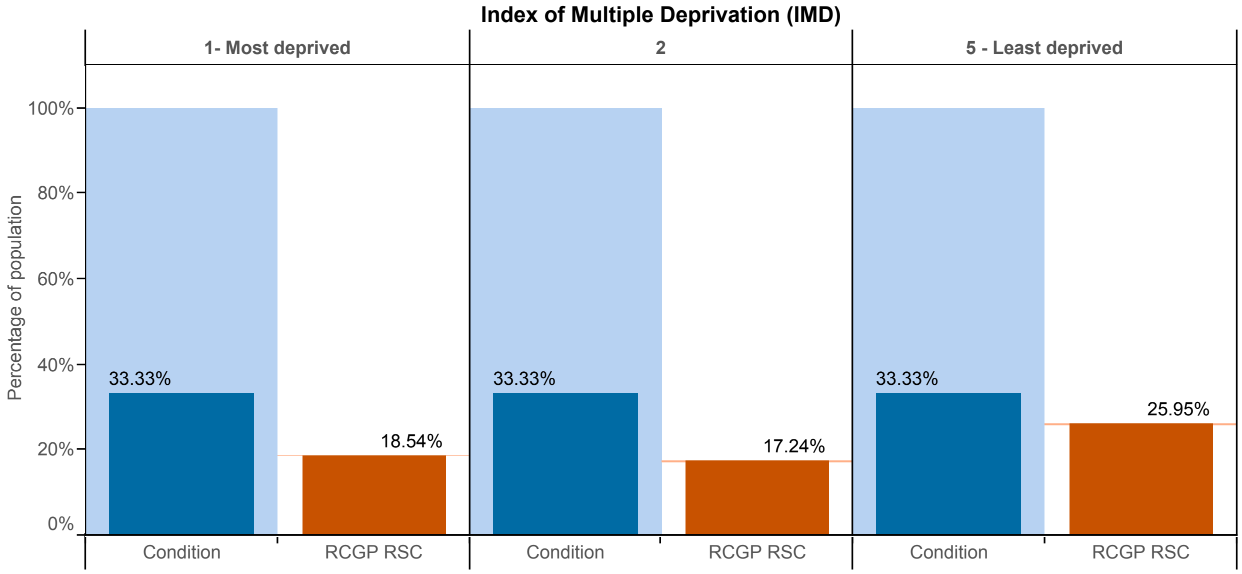

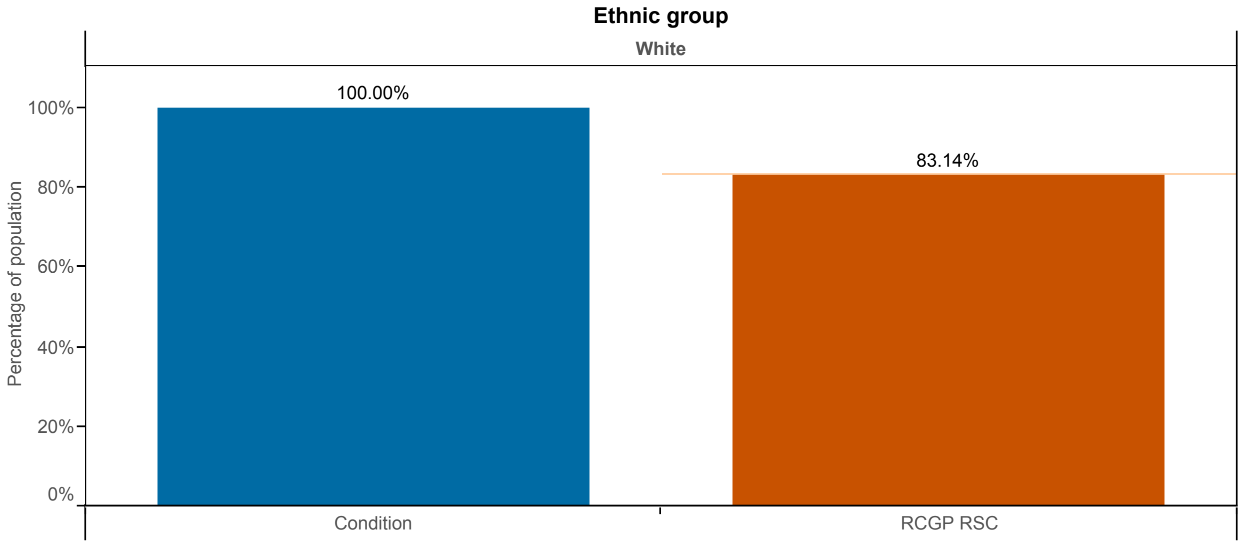

Scabies (ICD10 : B86)

Age-sex profile

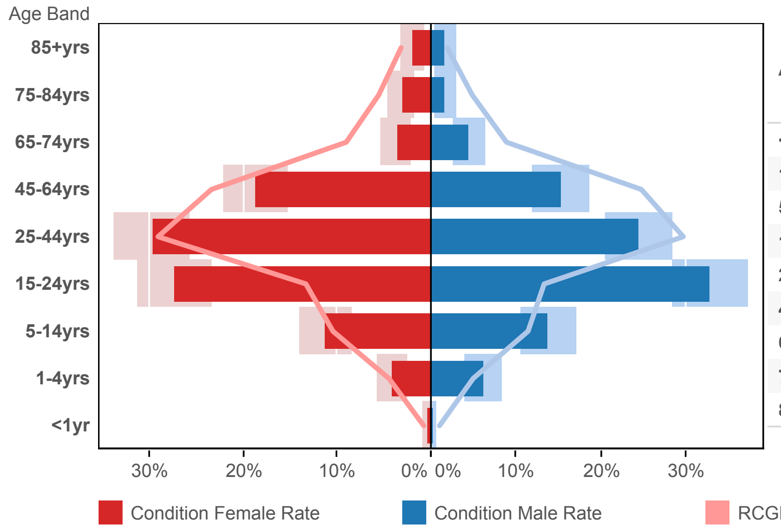

Age Band	Condition Female Rate	Condition Male Rate	Condition Female Population	Condition Male Population
<1yr	0.39	0.23	2.0	1.0
1-4yrs	4.07	6.09	21.0	27.0
5-14yrs	11.24	13.77	58.0	61.0
15-24yrs	27.33	32.73	141.0	145.0
25-44yrs	29.65	24.38	153.0	108.0
45-64yrs	18.60	15.35	96.0	68.0
65-74yrs	3.68	4.29	19.0	19.0
75-84yrs	3.10	1.58	16.0	7.0
85+yrs	1.94	1.58	10.0	7.0

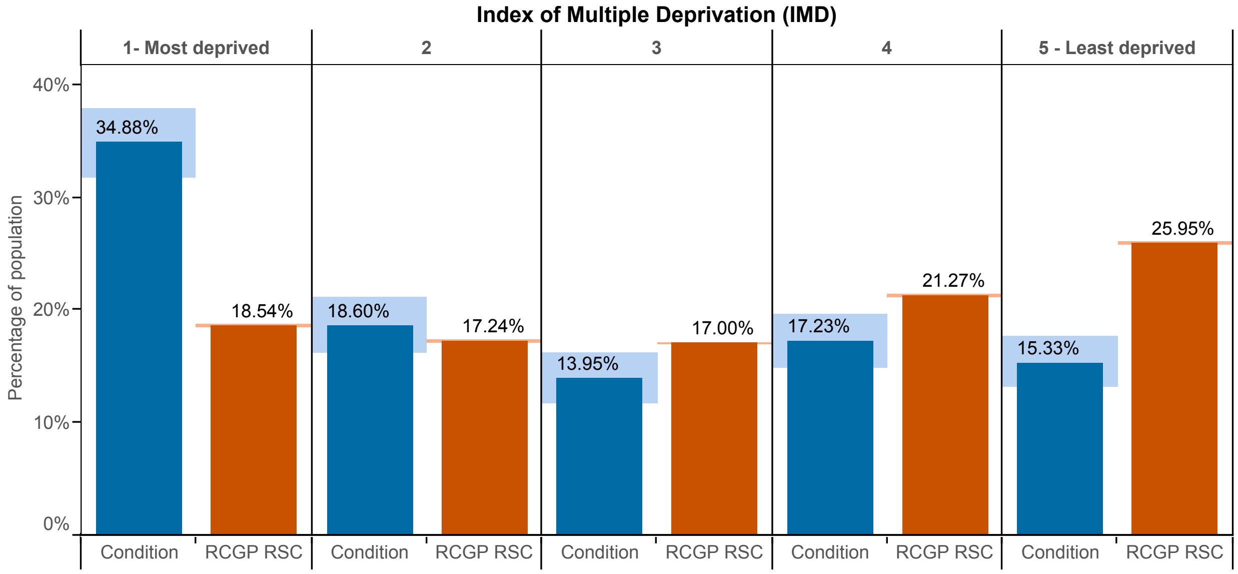

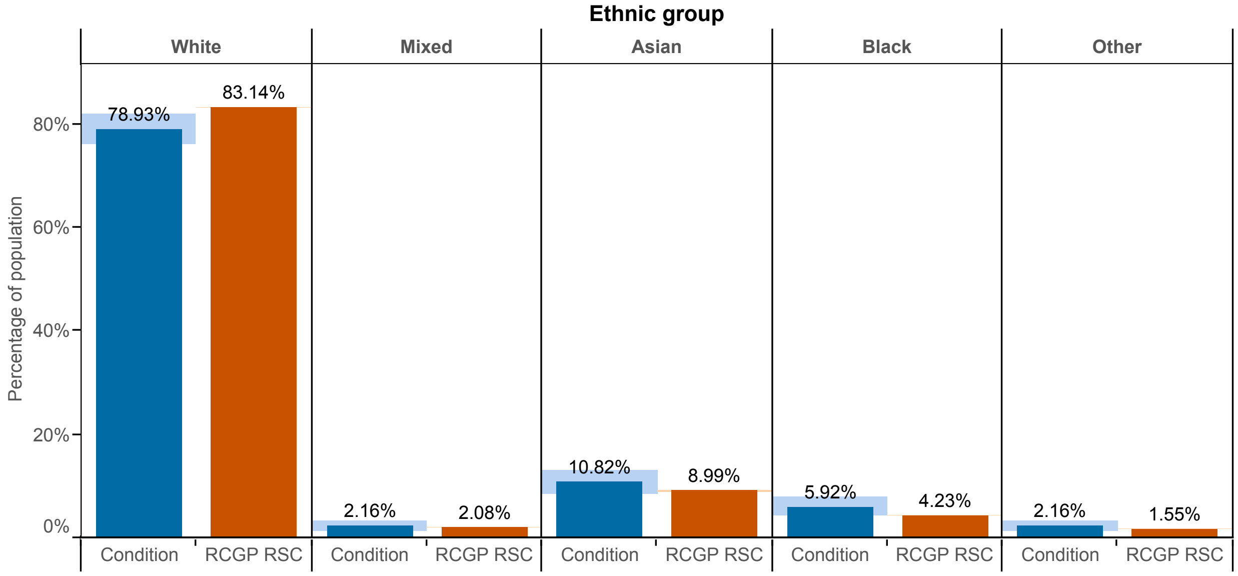

Acute Sinusitis (ICD10 : J01)

Age-sex profile

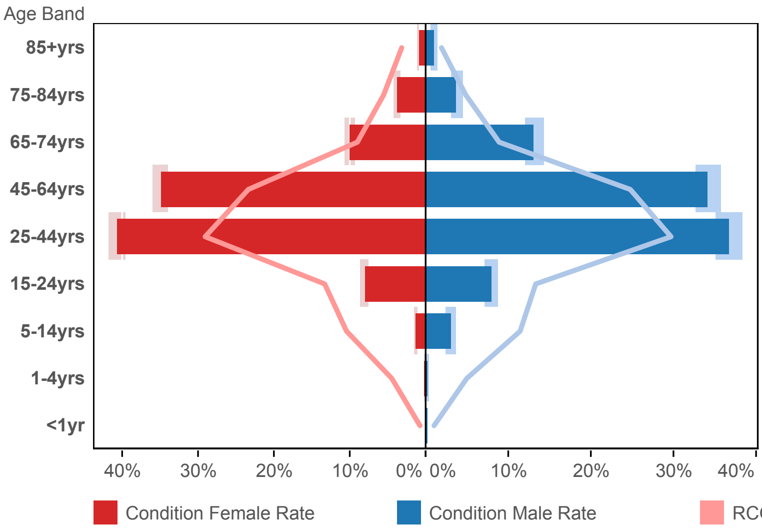

Age Band	Condition Female Rate	Condition Male Rate	Condition Female Population	Condition Male Population
<1yr	0.00	0.03	0	1
1-4yrs	0.20	0.24	17	8
5-14yrs	1.40	3.08	117	104
15-24yrs	8.06	7.95	674	268
25-44yrs	40.62	36.65	3,395	1,236
45-64yrs	34.90	34.16	2,917	1,152
65-74yrs	10.03	13.14	838	443
75-84yrs	3.80	3.77	318	127
85+yrs	0.98	0.98	82	33

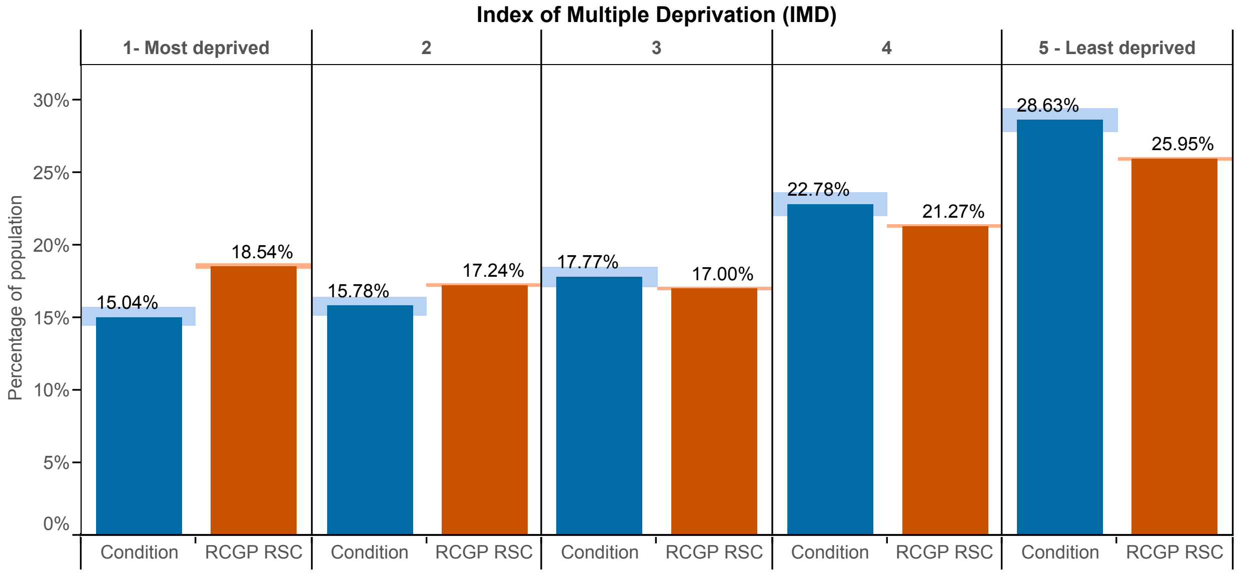

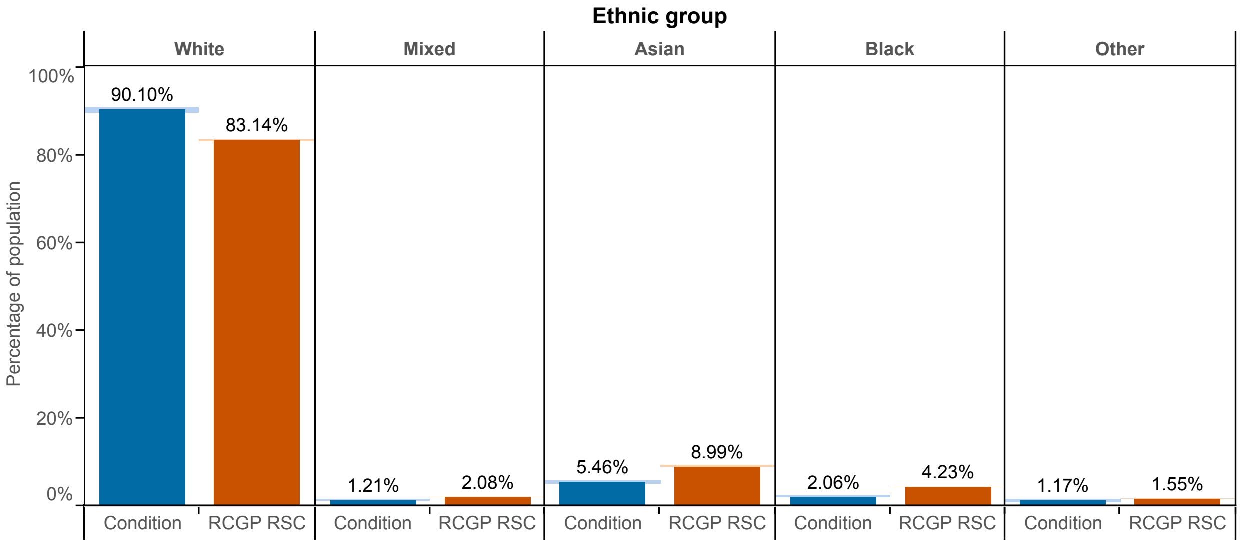

Skin / subcutaneous infections (ICD10 : L00 - L08)

Age-sex profile

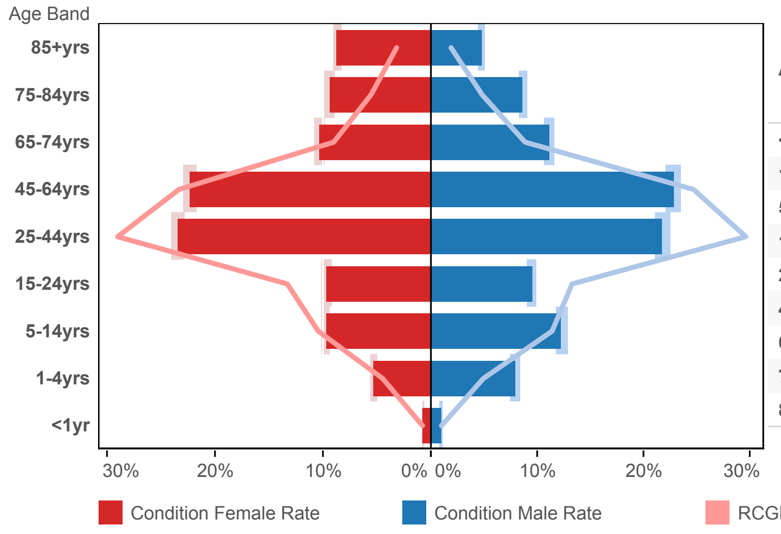

Age Band	Condition Female Rate	Condition Male Rate	Condition Female Population	Condition Male Population
<1yr	0.72	0.98	124	138
1-4yrs	5.36	8.00	920	1,121
5-14yrs	9.77	12.31	1,676	1,726
15-24yrs	9.66	9.56	1,657	1,340
25-44yrs	23.49	21.81	4,029	3,057
45-64yrs	22.46	22.82	3,852	3,199
65-74yrs	10.37	11.11	1,779	1,557
75-84yrs	9.42	8.65	1,616	1,213
85+yrs	8.73	4.74	1,497	665

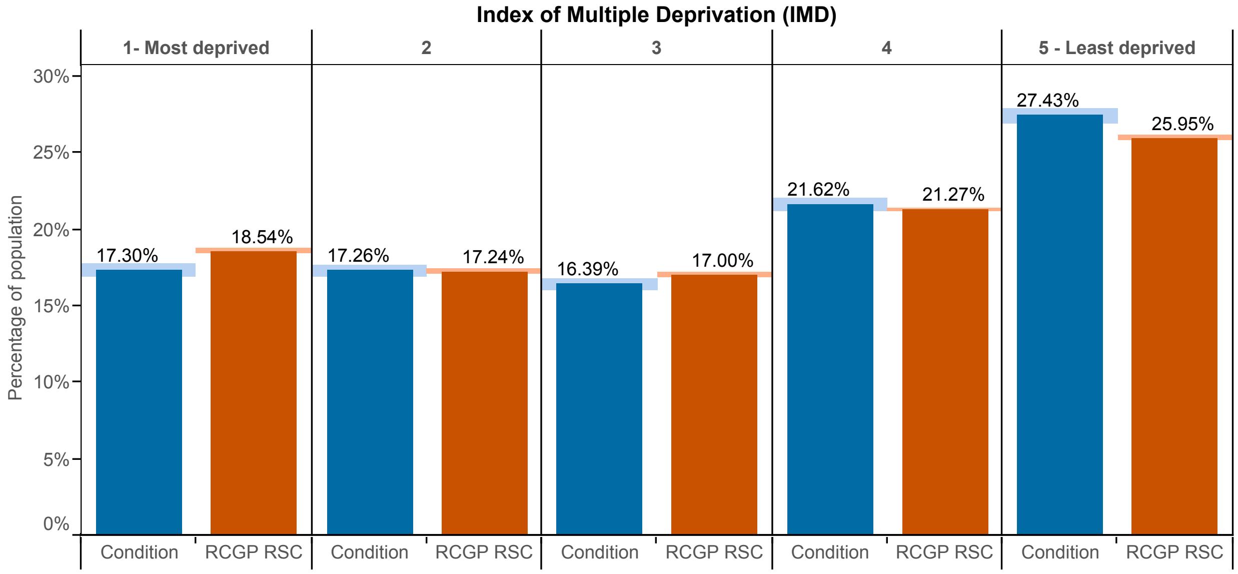

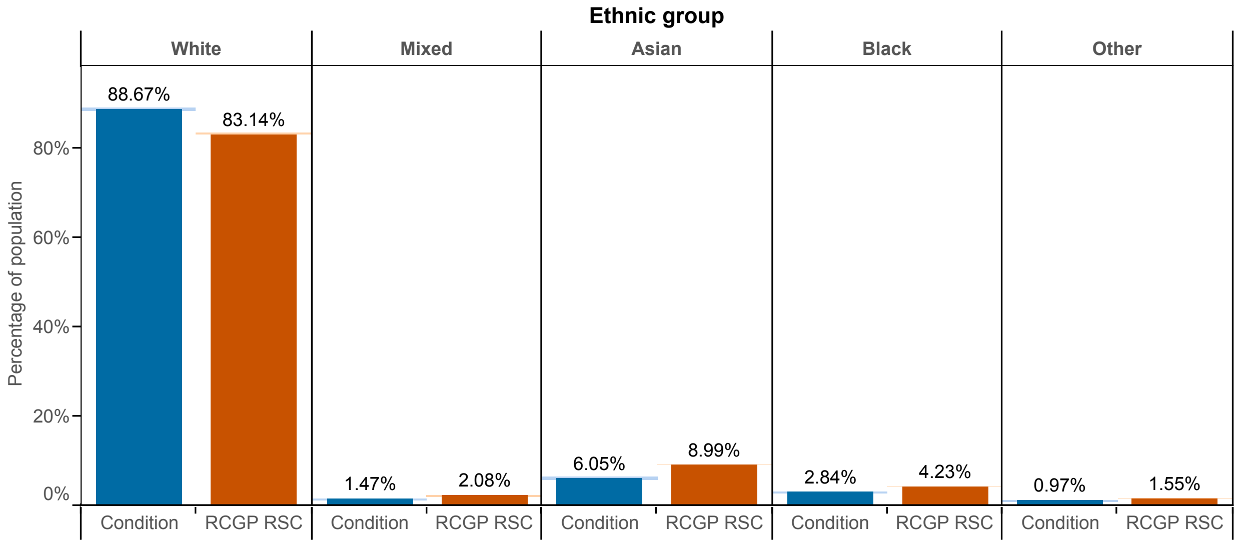

Strep throat / peritonsillar abscess (ICD10 : A38; J020; J36)

Age-sex profile

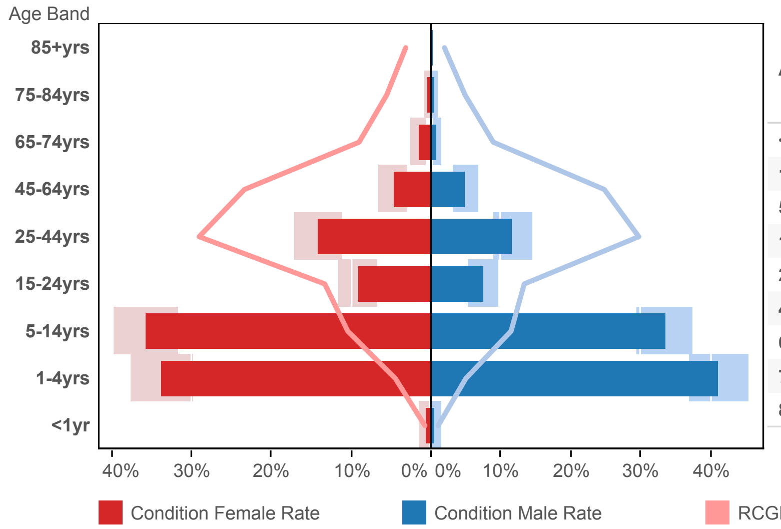

Age Band	Condition Female Rate	Condition Male Rate	Condition Female Population	Condition Male Population
<1yr	0.72	0.57	4.0	3.0
1-4yrs	33.75	41.06	188.0	216.0
5-14yrs	35.73	33.46	199.0	176.0
15-24yrs	9.16	7.41	51.0	39.0
25-44yrs	14.18	11.60	79.0	61.0
45-64yrs	4.67	4.75	26.0	25.0
65-74yrs	1.44	0.76	8.0	4.0
75-84yrs	0.36	0.38	2.0	2.0
85+yrs	0.00	0.00	0.0	0.0

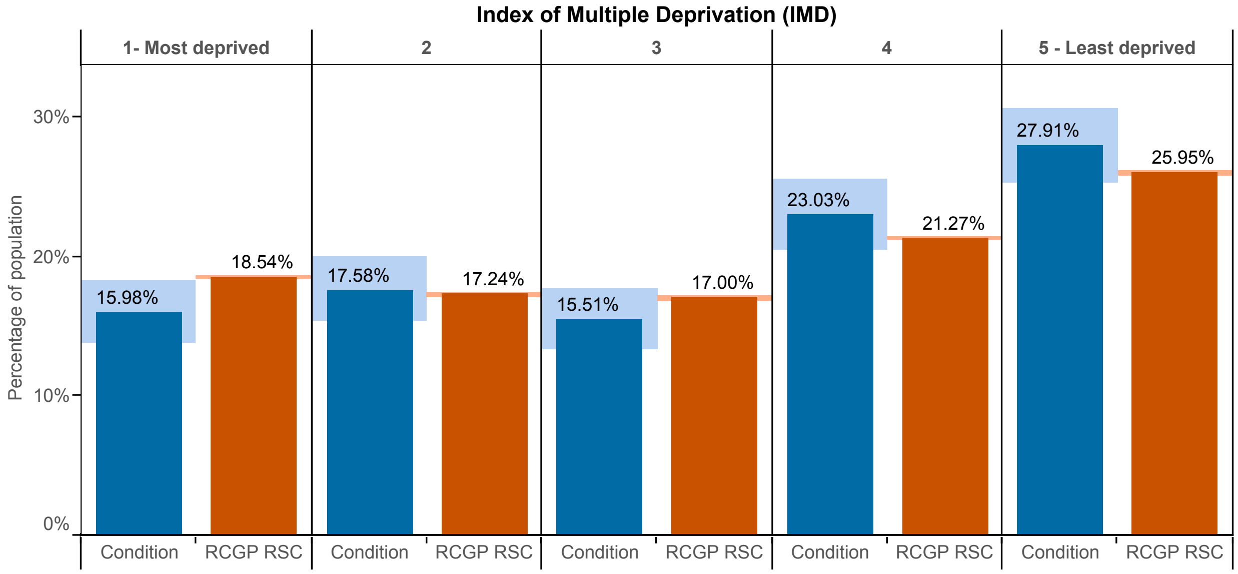

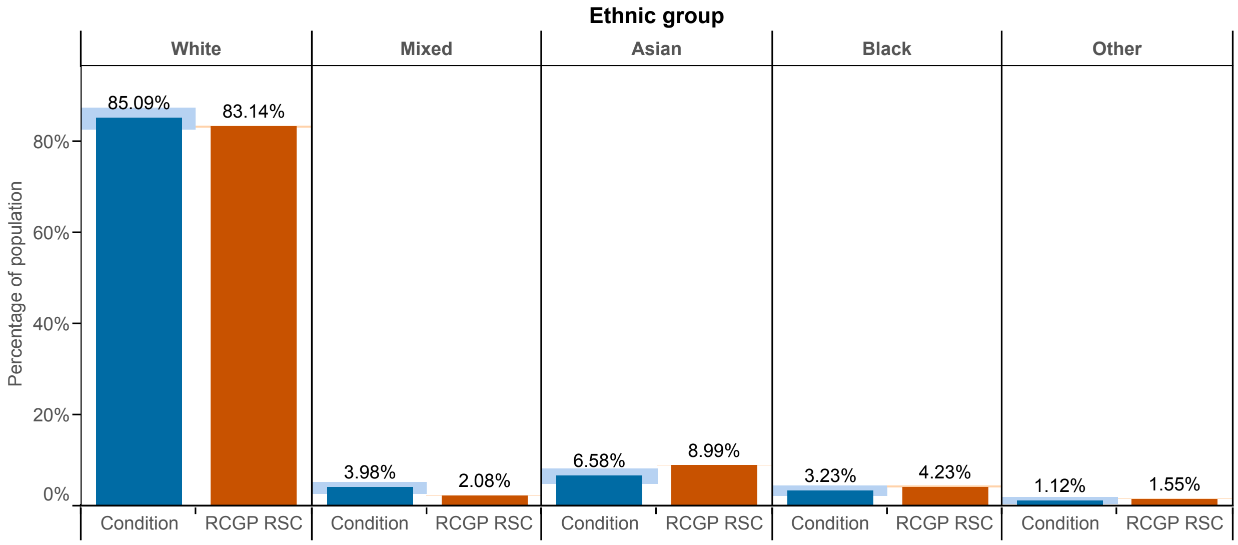

Musculoskeletal symptoms (ICD10 : R25 - R29)

Age-sex profile

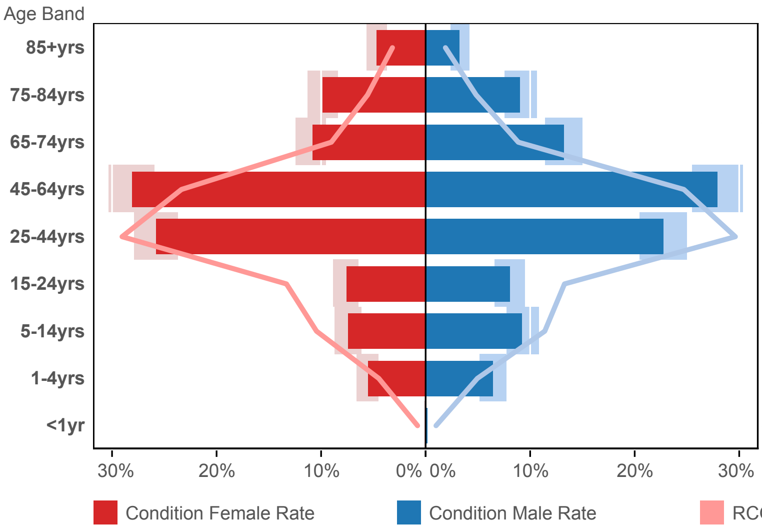

Age Band	Condition Female Rate	Condition Male Rate	Condition Female Population	Condition Male Population
<1yr	0.06	0.00	1.0	0.0
1-4yrs	5.56	6.43	97.0	88.0
5-14yrs	7.39	9.28	129.0	127.0
15-24yrs	7.62	8.04	133.0	110.0
25-44yrs	25.83	22.79	451.0	312.0
45-64yrs	28.18	27.90	492.0	382.0
65-74yrs	10.88	13.22	190.0	181.0
75-84yrs	9.85	9.06	172.0	124.0
85+yrs	4.64	3.29	81.0	45.0

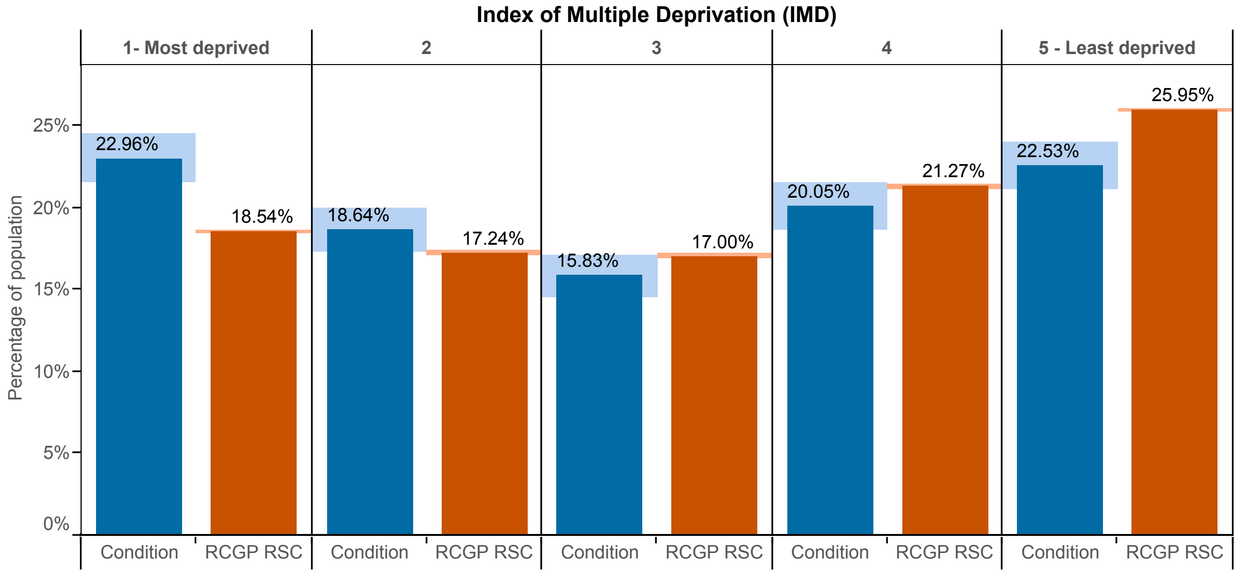

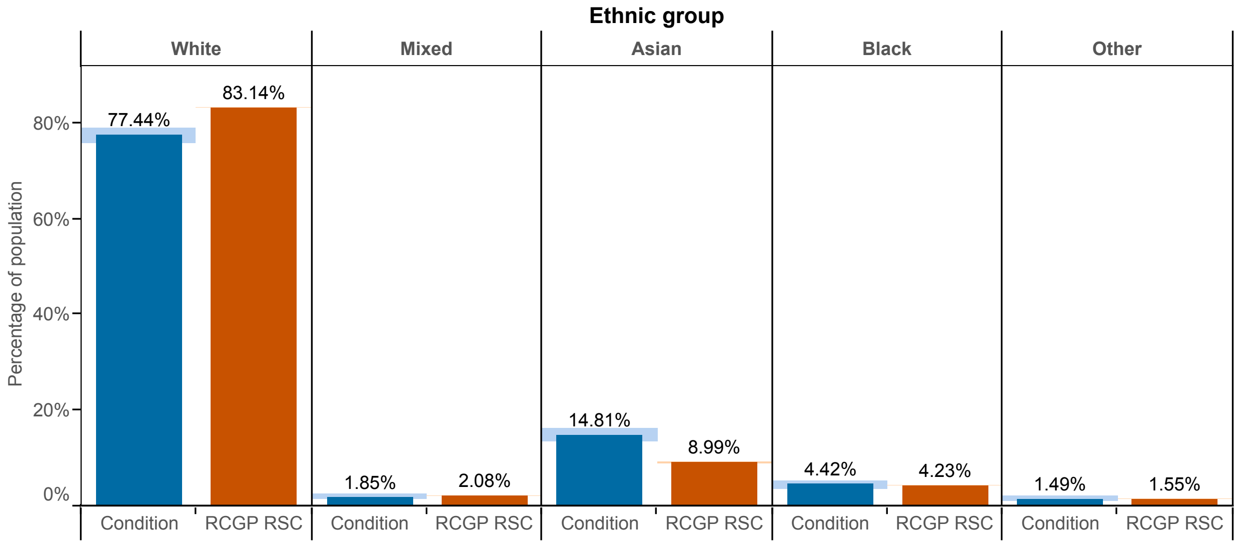

Respiratory / chest symptoms (ICD10 : R05 - R07; R09)

Age-sex profile

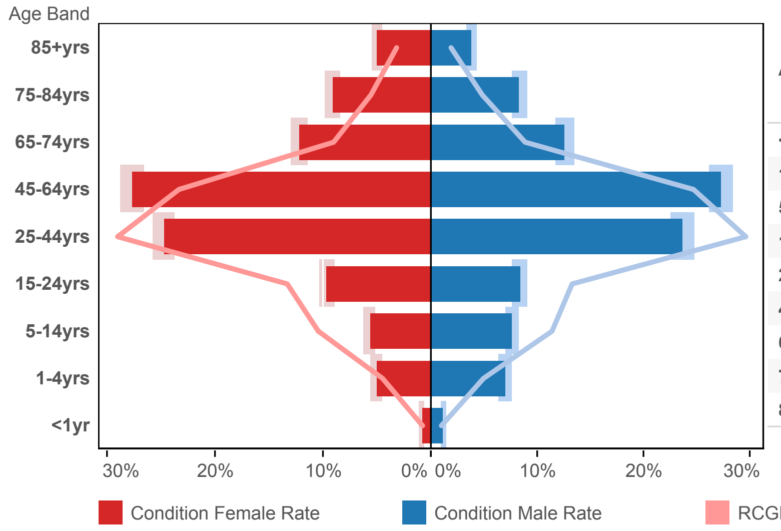

Age Band	Condition Female Rate	Condition Male Rate	Condition Female Population	Condition Male Population
<1yr	0.81	1.16	57	70
1-4yrs	5.06	7.05	355	426
5-14yrs	5.66	7.61	397	460
15-24yrs	9.63	8.38	676	506
25-44yrs	24.79	23.70	1,740	1,432
45-64yrs	27.71	27.35	1,945	1,652
65-74yrs	12.20	12.60	856	761
75-84yrs	9.13	8.34	641	504
85+yrs	5.01	3.81	352	230

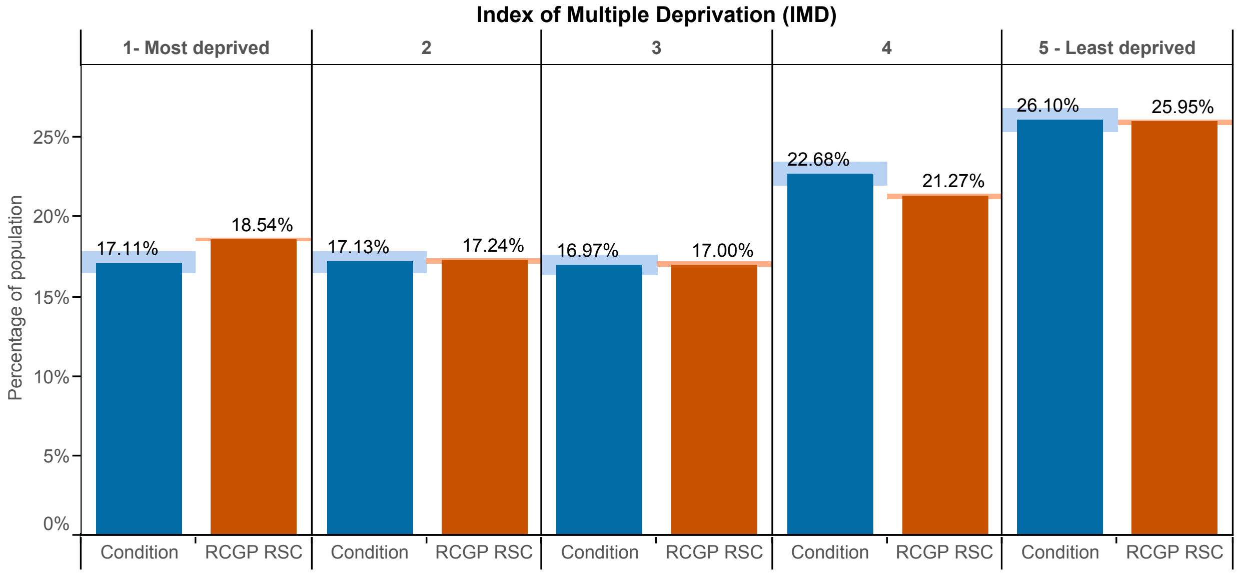

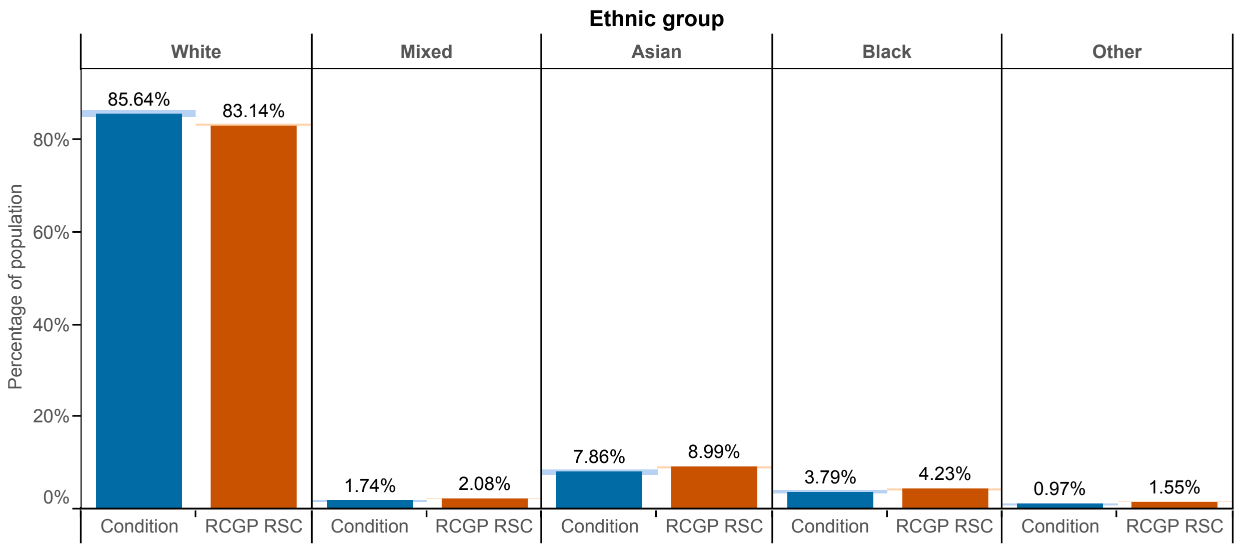

Skin symptoms (ICD10 : R20 - R23)

Age-sex profile

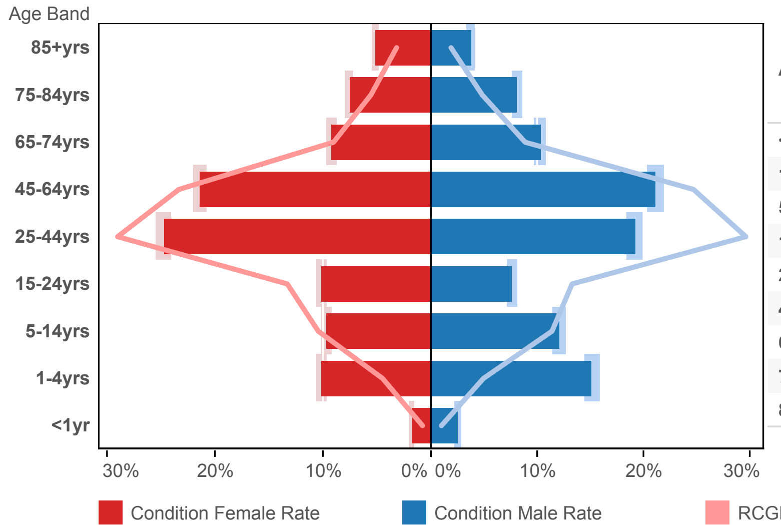

Age Band	Condition Female Rate	Condition Male Rate	Condition Female Population	Condition Male Population
<1yr	1.79	2.60	262	269
1-4yrs	10.18	15.13	1,488	1,568
5-14yrs	9.71	12.10	1,419	1,254
15-24yrs	10.14	7.64	1,482	792
25-44yrs	24.79	19.21	3,622	1,991
45-64yrs	21.43	21.17	3,132	2,194
65-74yrs	9.22	10.27	1,348	1,064
75-84yrs	7.57	8.13	1,106	842
85+yrs	5.16	3.75	754	389

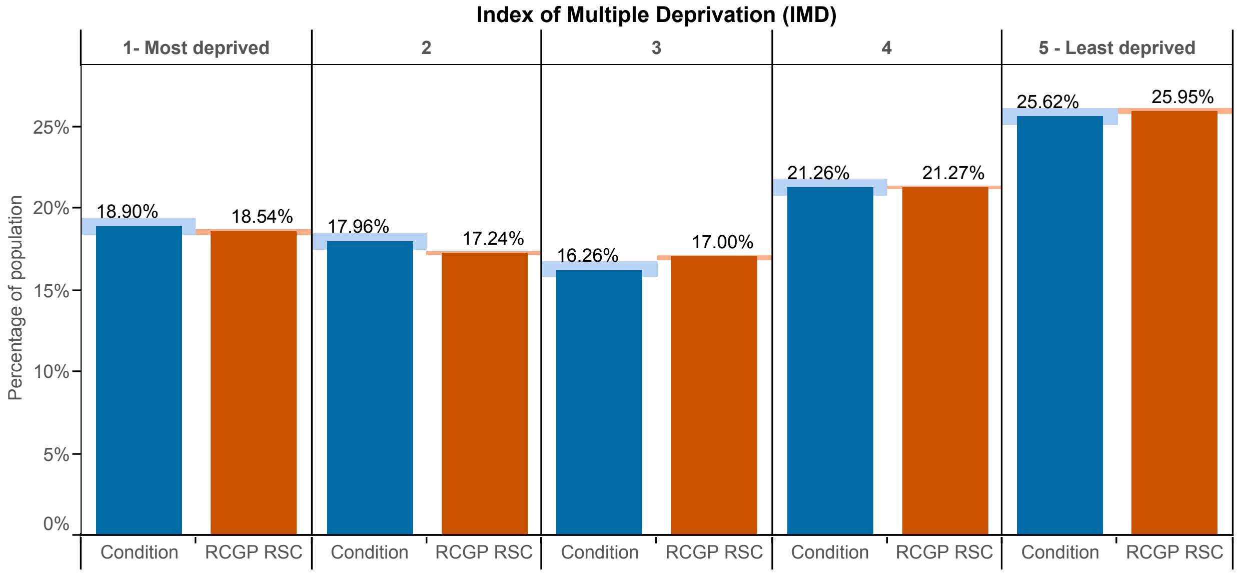

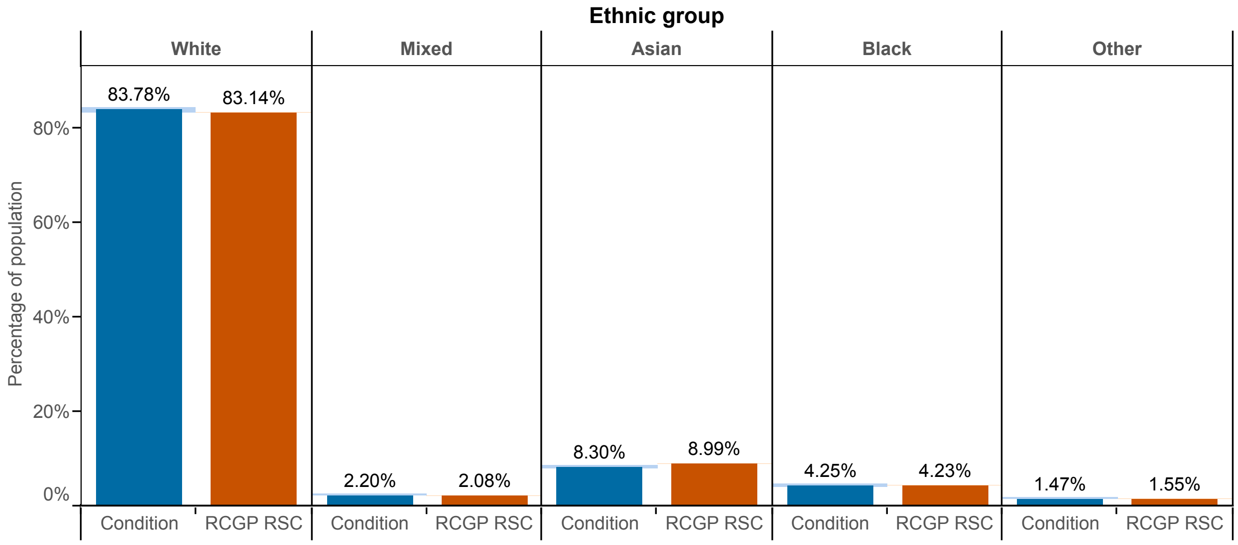

Tonsilitis / Pharyngitis (ICD10 : J02 - J03)

Age-sex profile

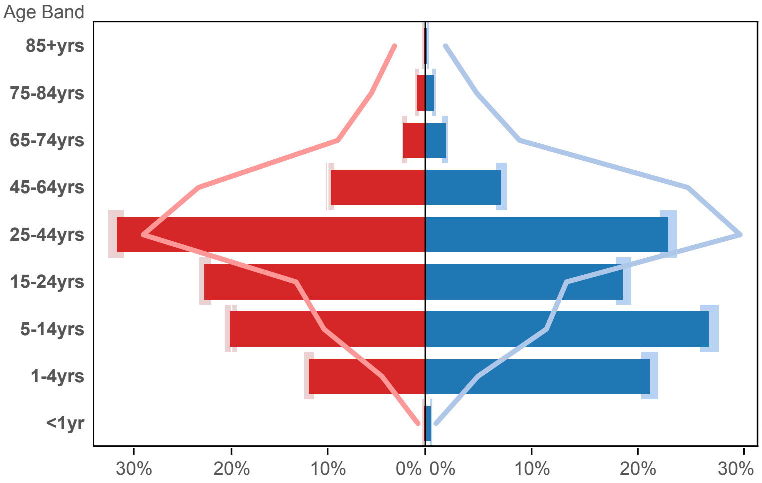

Age Band	Condition Female Rate	Condition Male Rate	Condition Female Population	Condition Male Population
<1yr	0.26	0.54	42	56
1-4yrs	12.03	21.13	1,943	2,201
5-14yrs	20.08	26.75	3,245	2,786
15-24yrs	22.68	18.67	3,664	1,945
25-44yrs	31.87	22.93	5,149	2,388
45-64yrs	9.76	7.13	1,577	743
65-74yrs	2.20	1.86	356	194
75-84yrs	0.89	0.77	143	80
85+yrs	0.24	0.22	39	23

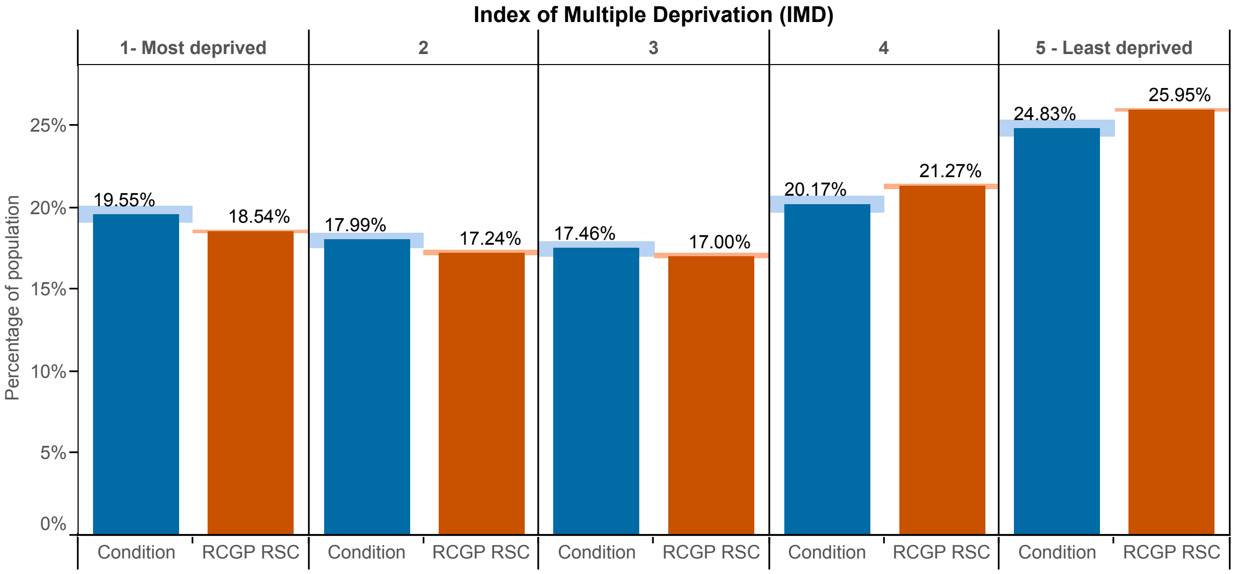

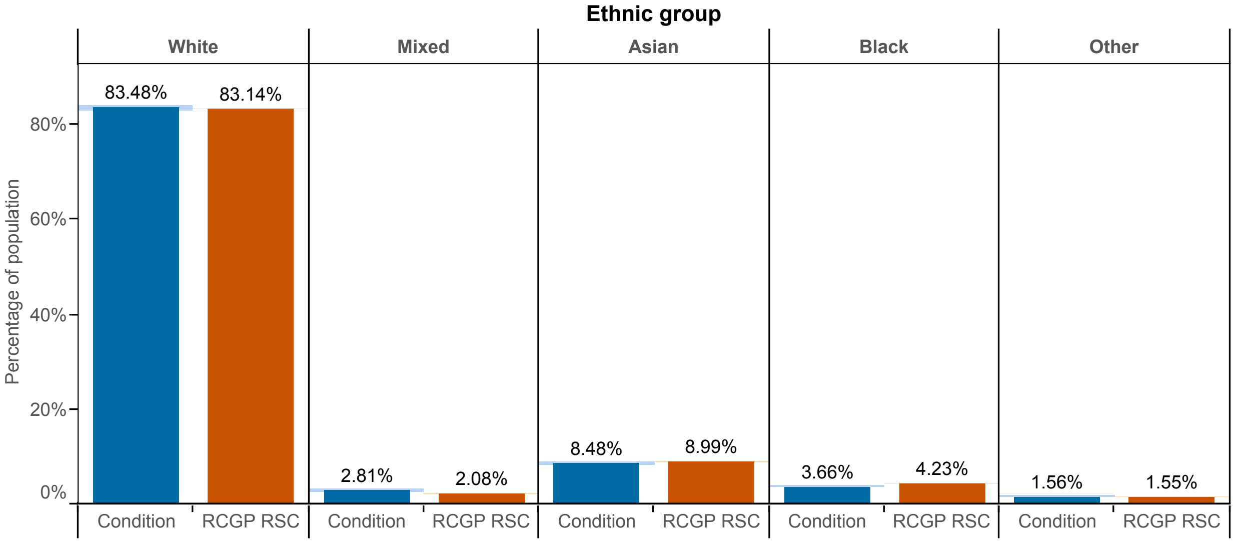

Upper Respiratory Tract Infections (ICD10 : J00 - J06)

Age-sex profile

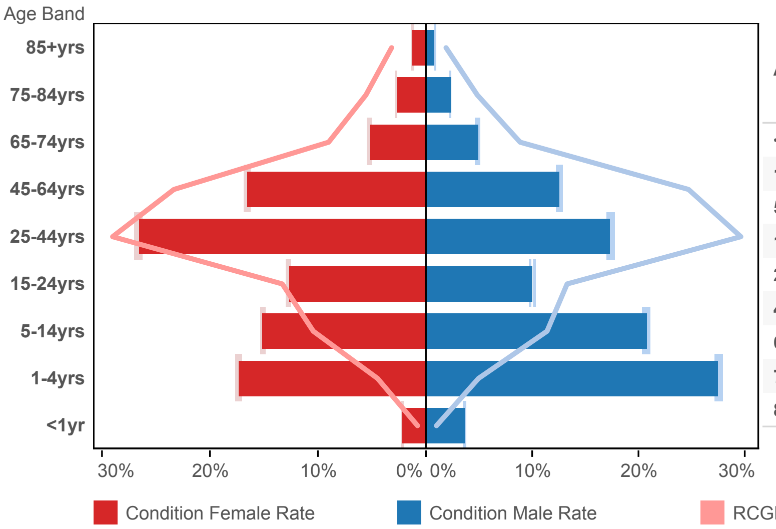

Age Band	Condition Female Rate	Condition Male Rate	Condition Female Population	Condition Male Population
<1yr	2.21	3.71	1,306	1,505
1-4yrs	17.43	27.56	10,305	11,184
5-14yrs	15.13	20.79	8,950	8,436
15-24yrs	12.72	10.03	7,523	4,071
25-44yrs	26.70	17.36	15,789	7,045
45-64yrs	16.61	12.50	9,825	5,074
65-74yrs	5.22	4.88	3,089	1,979
75-84yrs	2.72	2.31	1,606	939
85+yrs	1.26	0.85	745	346

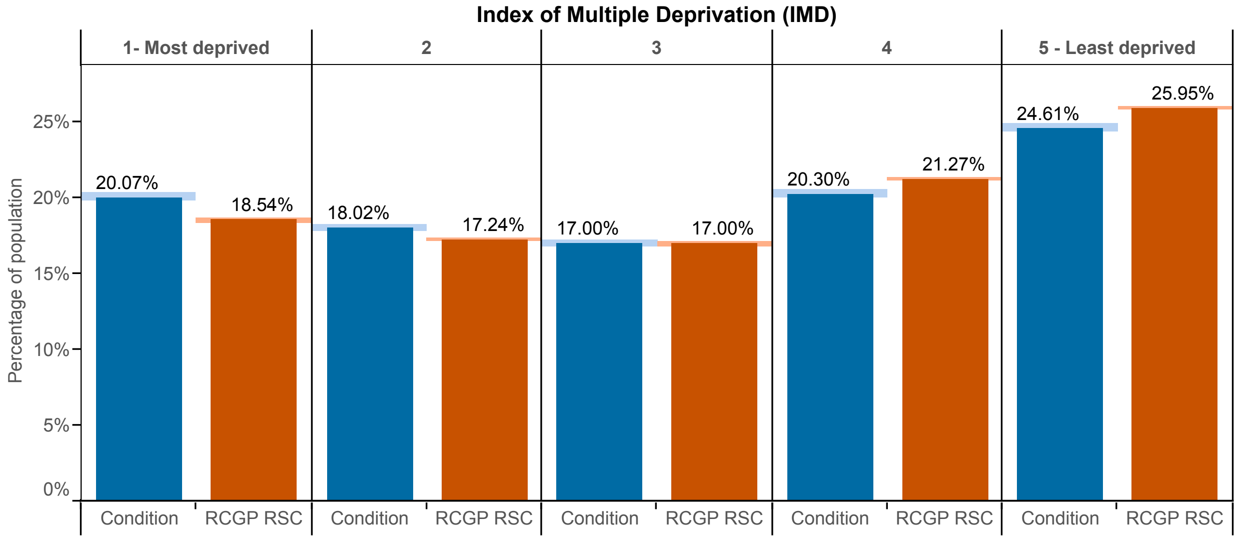

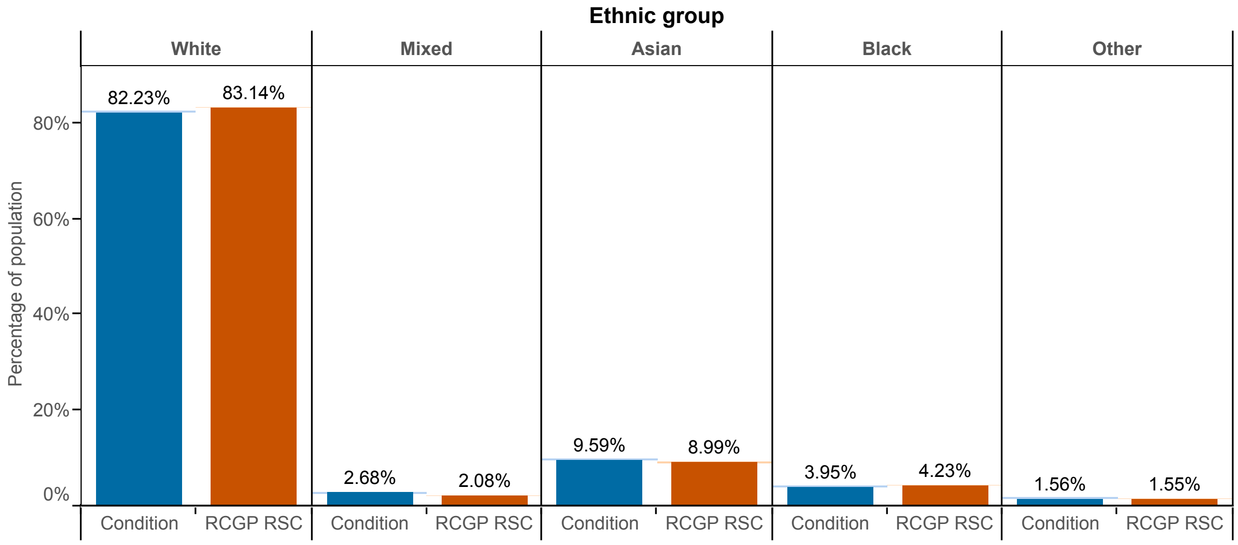

Urinary Tract Infections (ICD10 : N30; N390)

Age-sex profile

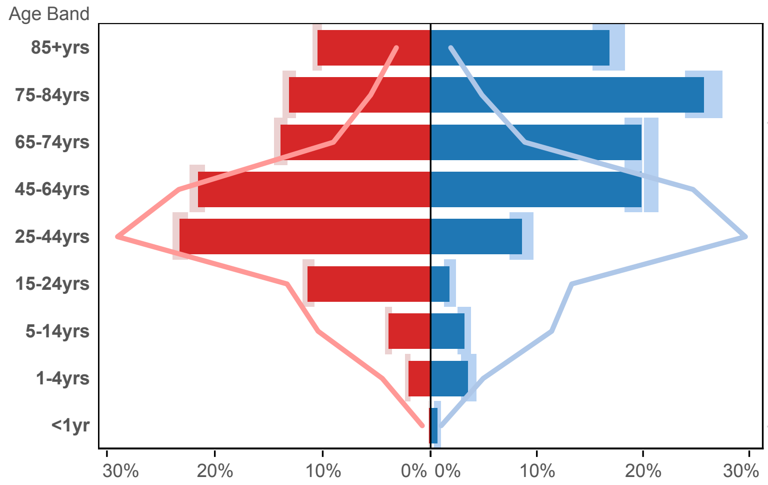

Age Band	Condition Female Rate	Condition Male Rate	Condition Female Population	Condition Male Population
<1yr	0.14	0.69	18	17
1-4yrs	2.06	3.54	262	87
5-14yrs	3.89	3.17	494	78
15-24yrs	11.42	1.79	1,451	44
25-44yrs	23.26	8.58	2,956	211
45-64yrs	21.68	19.85	2,755	488
65-74yrs	13.92	19.80	1,769	487
75-84yrs	13.16	25.78	1,672	634
85+yrs	10.47	16.80	1,331	413

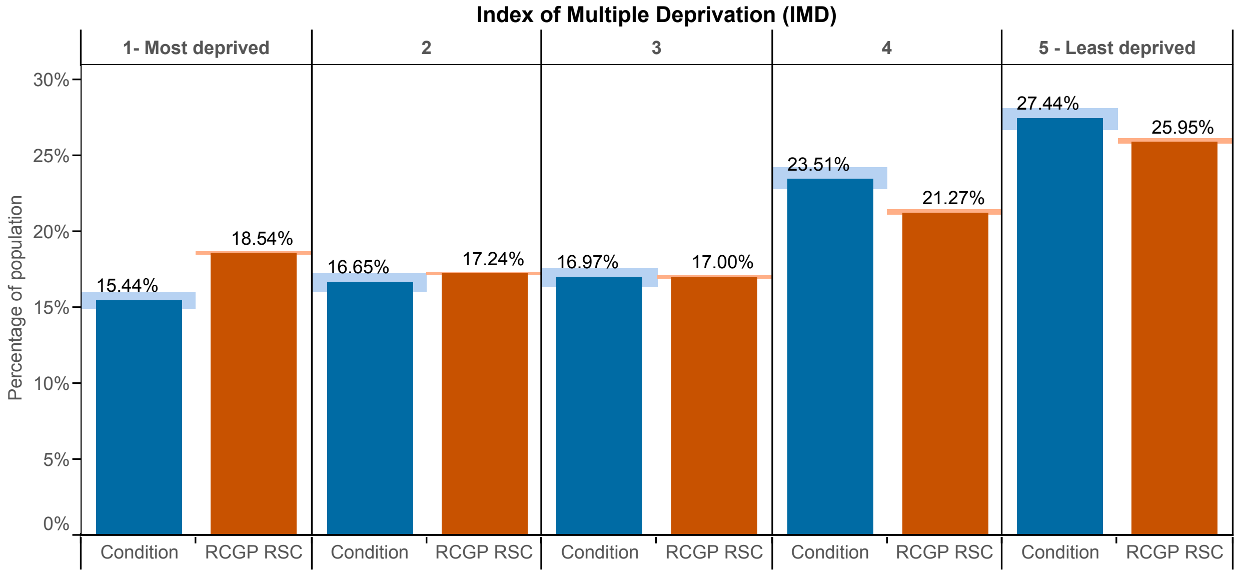

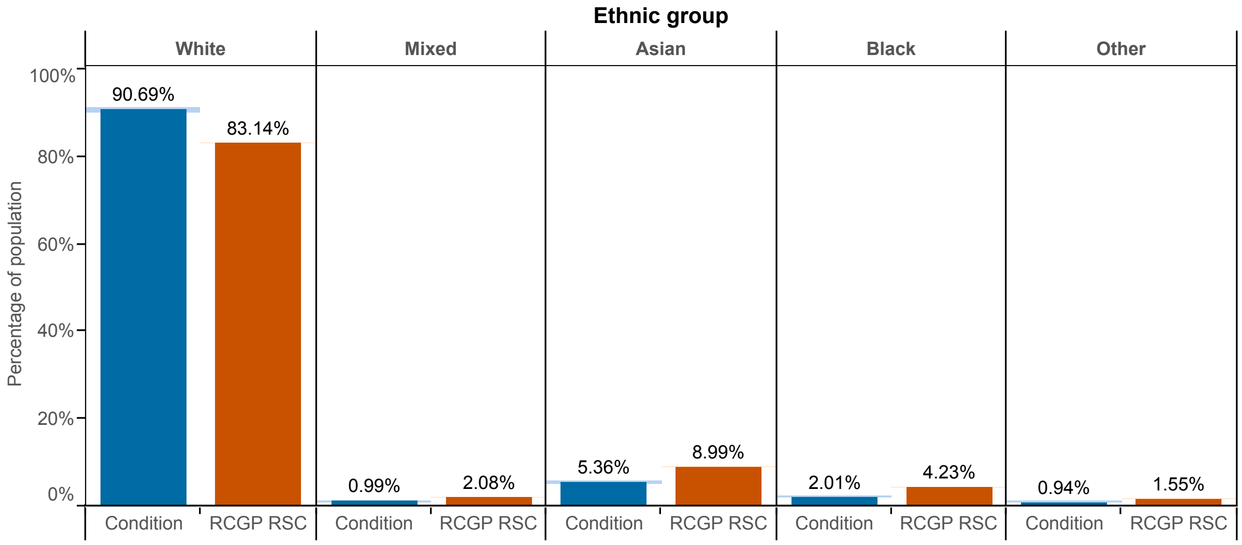

Viral Hepatitis (ICD10 : B15-B19)

Age-sex profile

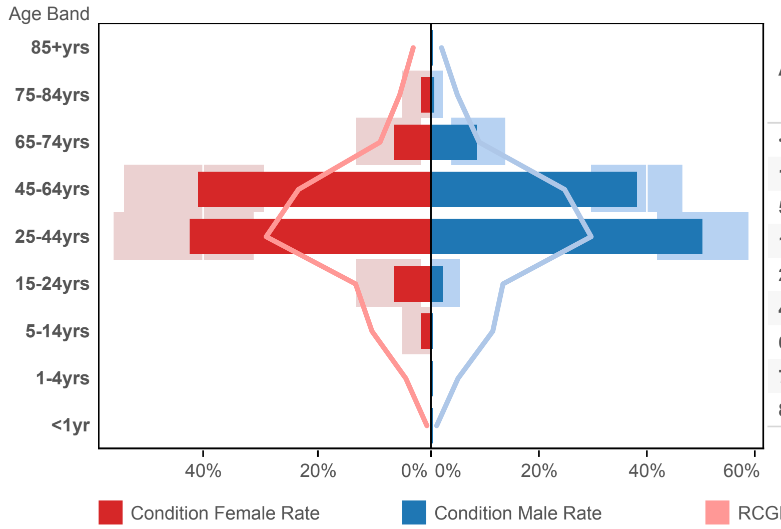

Age Band	Condition Female Rate	Condition Male Rate	Condition Female Population	Condition Male Population
<1yr	0.00	0.00	0.00	0.00
1-4yrs	0.00	0.00	0.00	0.00
5-14yrs	1.64	0.00	1.00	0.00
15-24yrs	6.56	2.29	4.00	3.00
25-44yrs	42.62	50.38	26.00	66.00
45-64yrs	40.98	38.17	25.00	50.00
65-74yrs	6.56	8.40	4.00	11.00
75-84yrs	1.64	0.76	1.00	1.00
85+yrs	0.00	0.00	0.00	0.00

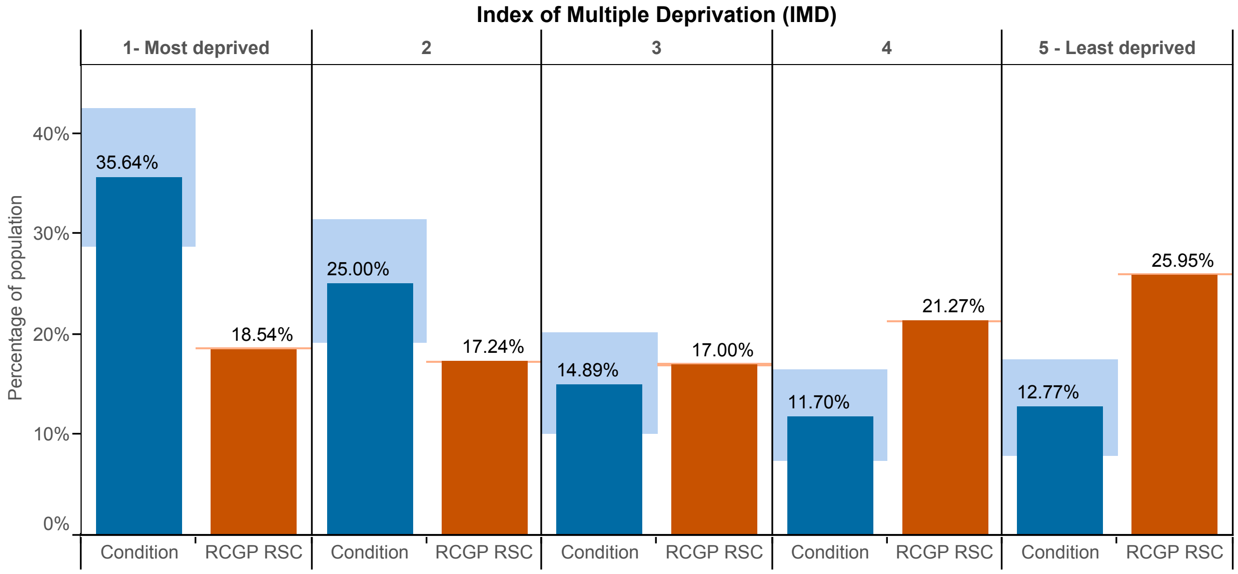

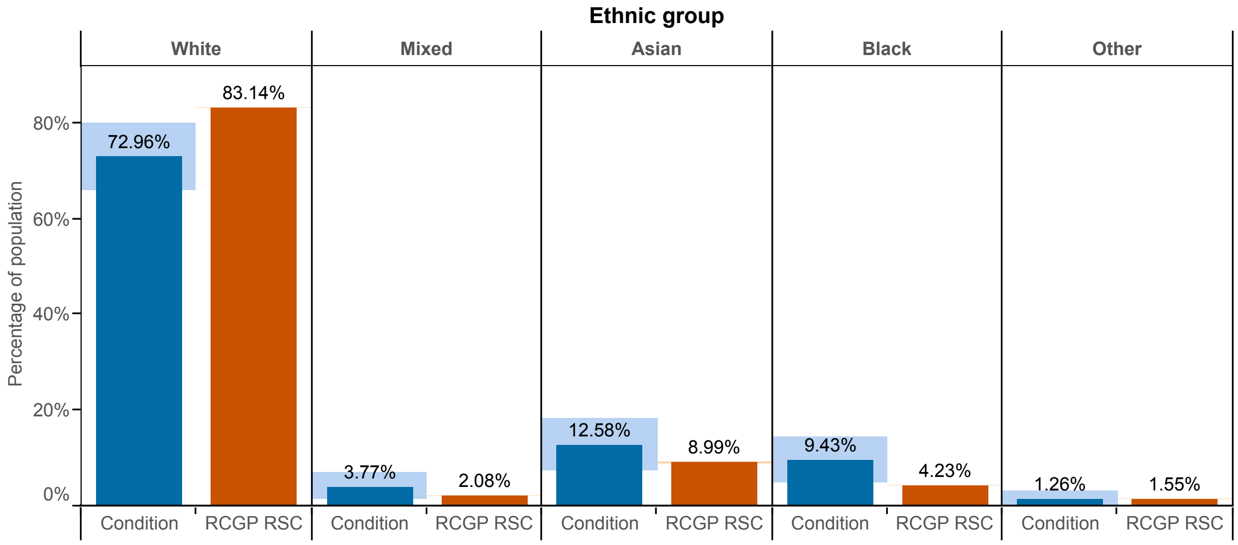

Pertussis (ICD10 : A37)

Age-sex profile

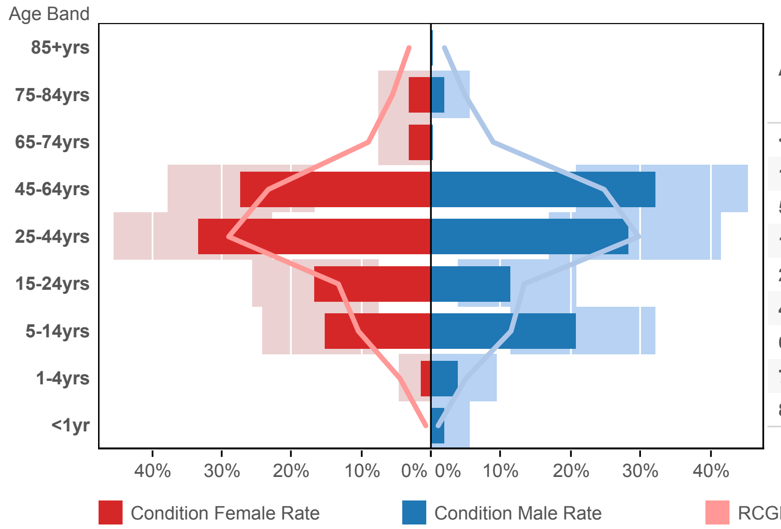

Age Band	Condition Female Rate	Condition Male Rate	Condition Female Population	Condition Male Population
<1yr	0.00	1.89	0.00	1.00
1-4yrs	1.52	3.77	1.00	2.00
5-14yrs	15.15	20.75	10.00	11.00
15-24yrs	16.67	11.32	11.00	6.00
25-44yrs	33.33	28.30	22.00	15.00
45-64yrs	27.27	32.08	18.00	17.00
65-74yrs	3.03	0.00	2.00	0.00
75-84yrs	3.03	1.89	2.00	1.00
85+yrs	0.00	0.00	0.00	0.00

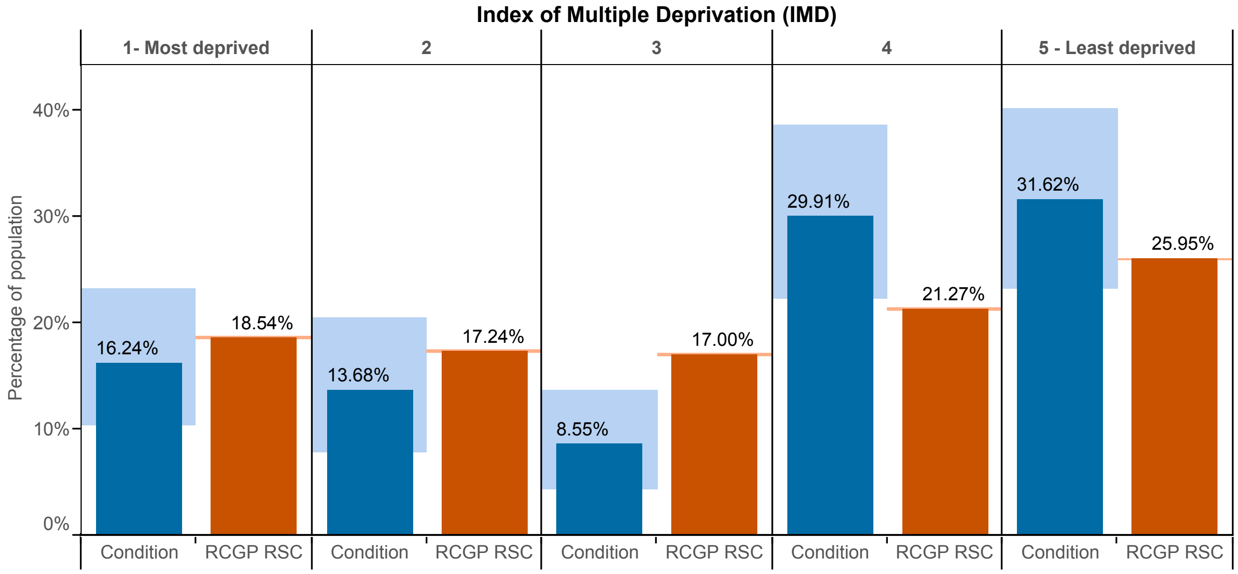

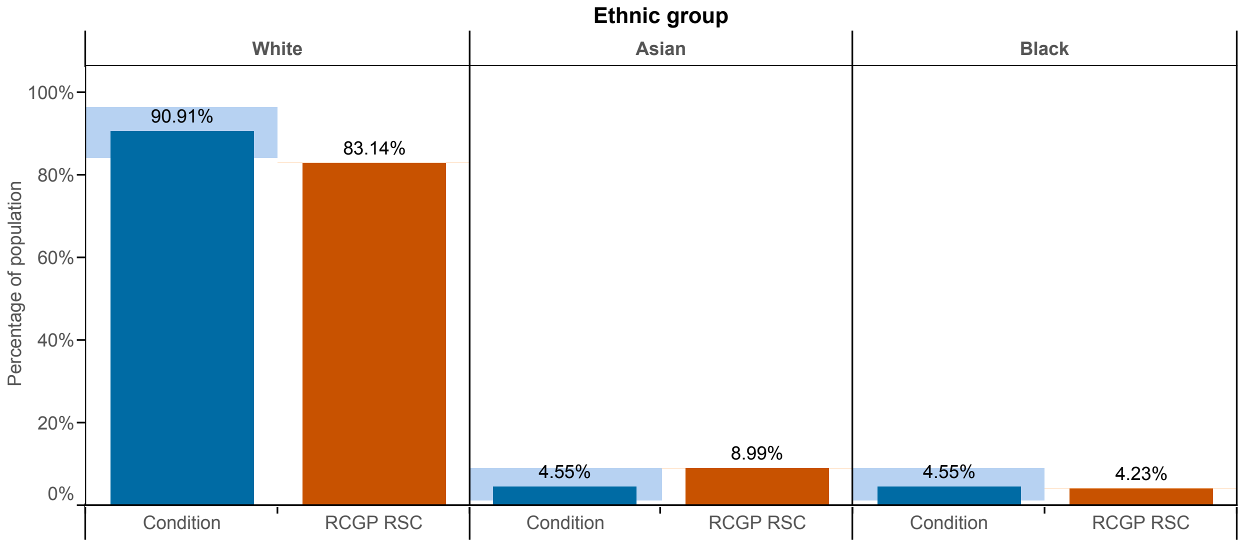

15. Appendix C: Data tables

These data tables show the incidence of monitored diseases by age, gender and region, by 4-week period, quarter and year. We are also including disparities for each individual condition.

Mean weekly incidence rate per 100,000 Persons.

Acute Bronchitis (ICD10: J20-J21,J40)

		All ages			Male									Female								
		Male & Female	Male	Female	<1yr	1-4yrs	5-14yrs	15-24yrs	25-44yrs	45-64yrs	65-74yrs	75-84yrs	85+yrs	<1yr	1-4yrs	5-14yrs	15-24yrs	25-44yrs	45-64yrs	65-74yrs	75-84yrs	85+yrs
**4 weekly**	1	71.88	68.50	75.26	0.00	101.94	18.40	11.35	23.68	40.65	84.94	132.18	203.36	0.00	79.73	13.70	20.56	33.86	61.96	86.69	117.71	263.16
	2	57.61	55.93	59.30	0.00	74.19	18.78	8.75	15.04	27.33	63.25	110.39	185.64	0.00	52.07	12.40	16.71	25.41	45.29	68.50	104.22	209.08
	3	61.95	53.78	70.12	0.00	84.72	16.54	10.36	15.18	34.83	64.28	113.18	144.93	156.25	43.15	12.52	18.37	27.88	39.96	62.13	96.62	174.22
	4	55.44	60.51	50.37	79.03	55.23	10.03	16.53	21.52	32.06	59.20	99.16	171.86	0.00	33.44	9.12	22.20	20.72	40.84	63.01	93.45	170.52
	5	66.72	65.78	67.66	31.50	95.81	19.78	11.69	16.53	34.83	69.08	120.02	192.80	32.94	65.94	14.19	25.46	29.79	51.79	70.04	128.89	189.88
	6	106.79	101.53	112.05	90.71	156.39	26.23	16.68	30.23	53.53	94.21	170.15	275.68	94.69	133.08	19.51	27.02	51.56	92.09	108.39	181.00	301.13
	7	149.42	145.22	153.61	310.86	333.42	35.33	12.44	26.05	55.66	106.25	160.34	266.67	314.79	282.65	25.03	30.22	48.07	76.91	132.04	183.47	289.33
	8	246.01	269.86	222.17	1134.34	443.58	48.37	21.55	34.46	63.18	131.64	199.25	352.35	667.15	385.78	40.70	33.32	61.14	100.87	154.67	207.06	348.86
	9	203.87	217.22	190.53	673.58	174.48	44.45	35.15	44.43	92.34	181.86	288.13	420.54	314.58	169.35	40.41	34.11	66.56	136.31	197.73	300.20	455.48
	10	180.22	185.50	174.94	440.33	251.59	60.47	30.01	49.20	92.68	153.95	228.72	362.53	236.02	197.73	48.93	39.60	86.48	134.80	184.29	233.51	413.10
	11	165.23	164.11	166.36	329.84	189.03	39.68	30.35	44.12	90.98	154.86	223.26	374.85	235.77	175.67	42.95	37.43	82.07	125.06	170.90	242.61	384.79
	12	155.95	158.12	153.78	313.89	177.86	51.41	25.70	46.33	75.99	150.63	212.87	368.41	149.18	146.27	46.70	43.76	73.87	110.18	189.73	239.59	384.72
	13	107.04	106.25	107.84	232.09	120.82	20.26	14.50	27.50	49.39	115.44	127.79	248.43	164.70	99.59	23.60	17.82	46.31	67.38	127.91	187.75	235.50
**Quarter**	1	64.44	60.10	68.77	0.00	88.10	17.95	10.25	18.40	34.76	71.91	119.63	179.93	48.08	59.97	12.94	18.70	29.42	50.06	73.54	107.07	219.15
	2	79.46	78.53	80.39	68.94	116.06	19.86	14.39	23.02	41.51	75.56	131.70	215.74	42.05	89.95	15.34	25.38	34.52	61.70	85.90	139.39	229.30
	3	205.16	216.02	194.31	712.51	314.47	48.36	25.39	37.08	74.85	145.10	227.35	359.05	439.26	273.71	39.01	33.55	63.91	113.49	165.07	234.88	385.87
	4	146.19	147.66	144.73	313.28	171.14	38.37	24.37	41.44	74.99	142.85	189.36	333.16	184.45	145.04	38.43	34.11	70.47	104.82	166.59	223.19	335.43
**Year**	15/16	124.24	126.00	122.48	274.43	172.42	31.27	18.71	30.20	56.88	109.50	167.43	273.12	178.57	142.22	26.66	28.05	49.98	82.94	123.60	177.02	293.25
**Episodes**	F/N	43119.00	18637.00	24482.00	517.00	2419.00	1075.00	721.00	2541.00	4400.00	3020.00	2482.00	1462.00	309.00	1939.00	882.00	1066.00	4223.00	6350.00	3686.00	3173.00	2854.00

Mean weekly incidence rate per 100,000 Persons.

Acute Bronchitis (ICD10: J20-J21,J40)

			All ages			Male									Female								
			Male & Female	Male	Female	<1yr	1-4yrs	5-14yrs	15-24yrs	25-44yrs	45-64yrs	65-74yrs	75-84yrs	85+yrs	<1yr	1-4yrs	5-14yrs	15-24yrs	25-44yrs	45-64yrs	65-74yrs	75-84yrs	85+yrs
**North**	Quarter	1	86.54	72.71	100.37	0.00	105.64	15.87	12.13	27.26	43.78	89.94	144.40	215.38	192.31	75.58	11.61	25.42	46.55	68.49	97.22	152.38	233.72
		2	91.00	92.39	89.61	122.19	116.27	15.71	18.82	28.31	50.64	86.51	160.02	233.04	23.46	102.45	15.29	25.22	43.42	75.14	97.97	152.55	270.96
		3	215.85	222.71	209.00	723.95	298.57	44.88	25.29	39.65	87.32	155.24	244.81	384.64	464.74	271.27	36.11	33.87	70.10	139.09	177.15	257.46	431.22
		4	158.85	163.51	154.20	420.83	174.62	38.43	24.63	47.77	78.63	144.30	200.36	342.01	237.72	125.30	38.81	33.06	80.36	114.94	172.72	231.82	353.06
	Year	15/16	138.45	138.31	138.59	318.71	173.79	28.91	20.30	35.97	65.35	119.47	187.64	294.68	229.71	143.30	25.71	29.46	60.49	99.71	136.95	199.18	322.82
	Episodes	F/N	15,394.00	6,558.00	8,836.00	193.00	707.00	297.00	280.00	880.00	1,619.00	1,145.00	945.00	492.00	107.00	562.00	256.00	409.00	1,425.00	2,443.00	1,408.00	1,224.00	1,002.00
**South**	Quarter	1	53.52	53.80	53.24	0.00	86.60	19.24	6.89	15.57	30.17	63.00	105.27	157.49	0.00	50.30	12.72	15.89	24.85	41.98	55.13	82.49	195.75
		2	73.78	71.70	75.86	35.94	116.31	18.89	11.32	20.85	32.29	69.71	101.21	238.78	35.71	95.92	15.09	21.05	31.17	44.97	80.53	122.79	235.48
		3	218.00	232.83	203.17	957.09	317.74	50.62	25.00	39.36	71.80	120.21	208.70	304.91	589.45	324.59	37.70	35.35	61.09	94.08	140.61	183.00	362.66
		4	146.23	143.18	149.28	299.05	201.33	40.20	20.36	42.38	66.75	128.17	180.28	310.10	247.85	175.03	37.91	34.63	70.29	97.86	163.94	192.54	323.47
	Year	15/16	123.32	125.71	120.93	322.57	180.89	32.39	15.98	29.78	50.56	95.90	149.46	253.90	218.81	161.72	26.08	26.88	47.29	70.25	111.07	146.10	280.17
	Episodes	F/N	9,937.00	4,348.00	5,589.00	124.00	578.00	270.00	153.00	542.00	966.00	724.00	602.00	389.00	85.00	487.00	205.00	241.00	847.00	1,343.00	887.00	712.00	782.00
**London**	Quarter	1	58.52	54.84	62.21	0.00	85.49	20.11	6.51	11.16	29.66	69.27	90.79	180.58	0.00	64.34	16.76	14.36	17.08	42.16	71.58	103.66	229.92
		2	79.73	81.03	78.43	117.63	114.78	23.74	8.98	18.80	42.45	78.19	142.61	182.04	68.54	89.53	16.59	24.42	23.42	66.65	82.73	162.76	171.25
		3	163.16	159.89	166.42	396.11	264.19	52.12	24.68	25.17	64.74	143.88	244.72	223.43	308.25	213.76	41.97	29.00	46.24	101.75	172.02	237.06	347.74
		4	125.30	119.85	130.76	218.18	114.07	32.55	19.53	28.48	65.39	146.55	179.72	274.18	127.54	131.53	35.28	28.03	51.59	90.17	150.38	236.64	325.64
	Year	15/16	107.03	104.20	109.86	183.65	144.06	32.14	15.01	21.05	50.84	110.17	164.75	216.17	126.11	124.92	27.79	24.03	34.90	75.47	119.77	186.00	269.71
	Episodes	F/N	7,428.00	3,174.00	4,254.00	105.00	568.00	261.00	114.00	578.00	726.00	365.00	308.00	149.00	70.00	492.00	223.00	195.00	1,025.00	1,021.00	457.00	453.00	318.00
**Midlands And East**	Quarter	1	59.16	59.06	59.27	0.00	74.68	16.57	15.46	19.63	35.42	65.42	138.06	166.27	0.00	49.63	10.66	19.13	29.19	47.61	70.21	89.76	217.22
		2	73.34	69.01	77.67	0.00	116.87	21.11	18.45	24.11	40.65	67.84	122.97	209.10	40.48	71.89	14.39	30.84	40.07	60.03	82.37	119.44	239.52
		3	223.64	248.64	198.63	772.88	377.39	45.81	26.57	44.16	75.54	161.09	211.16	523.21	394.60	285.21	40.25	35.99	78.24	119.06	170.51	261.98	401.85
		4	154.39	164.11	144.67	315.04	194.53	42.31	32.98	47.12	89.20	152.38	197.10	406.35	124.69	148.30	41.72	40.72	79.65	116.30	179.31	231.77	339.54
	Year	15/16	128.14	135.75	120.52	272.79	190.94	31.65	23.55	34.01	60.75	112.45	167.88	327.74	139.66	138.94	27.04	31.84	57.22	86.33	126.61	176.79	300.29
	Episodes	F/N	10,360.00	4,557.00	5,803.00	95.00	566.00	247.00	174.00	541.00	1,089.00	786.00	627.00	432.00	47.00	398.00	198.00	221.00	926.00	1,543.00	934.00	784.00	752.00

Mean weekly incidence rate per 100,000 Persons.

Acute Laryngitis/Tracheitis (ICD10: J04)

		All ages			Male									Female								
		Male & Female	Male	Female	<1yr	1-4yrs	5-14yrs	15-24yrs	25-44yrs	45-64yrs	65-74yrs	75-84yrs	85+yrs	<1yr	1-4yrs	5-14yrs	15-24yrs	25-44yrs	45-64yrs	65-74yrs	75-84yrs	85+yrs
**4 weekly**	1	3.82	3.63	4.02	0.00	15.07	4.66	1.68	1.58	1.28	1.23	3.29	3.90	0.00	11.25	3.17	5.73	5.89	5.84	2.85	0.56	0.85
	2	3.46	3.57	3.36	0.00	23.35	2.24	0.73	0.45	0.70	1.48	0.95	2.21	0.00	6.12	3.70	2.47	5.19	3.56	3.90	0.00	5.33
	3	4.79	4.01	5.57	0.00	27.16	3.86	0.68	0.36	0.92	3.12	0.00	0.00	0.00	31.44	2.43	2.31	5.27	3.42	3.15	2.14	0.00
	4	4.20	4.40	4.00	0.00	31.41	1.75	1.07	0.22	2.11	0.47	2.59	0.00	0.00	19.48	1.76	2.31	1.97	3.09	4.62	2.76	0.00
	5	9.10	9.62	8.57	0.00	75.46	6.88	0.22	0.38	1.03	1.83	0.82	0.00	0.00	55.22	3.84	4.19	2.67	1.83	3.56	0.49	5.33
	6	21.42	21.12	21.72	15.21	148.86	19.24	0.85	1.39	1.56	1.68	1.33	0.00	31.85	116.43	12.68	5.97	6.97	8.82	4.60	1.93	6.22
	7	17.28	20.64	13.93	53.52	109.01	13.94	0.77	1.10	2.19	1.71	3.50	0.00	0.00	88.26	9.62	4.38	7.15	7.90	5.35	1.46	1.23
	8	16.62	15.20	18.04	19.48	91.19	12.45	1.06	1.03	1.89	2.79	2.22	4.72	39.59	75.06	10.59	4.82	6.26	6.94	8.56	5.56	5.00
	9	8.84	7.40	10.27	11.53	42.23	4.46	3.17	1.10	0.78	2.43	0.94	0.00	16.70	32.73	5.35	4.59	8.73	6.67	6.55	8.11	2.98
	10	11.98	10.33	13.63	16.78	55.04	8.27	2.78	1.63	0.79	0.00	3.09	4.63	50.22	28.21	4.73	6.99	9.67	9.72	5.47	5.02	2.69
	11	11.90	15.49	8.31	60.63	51.64	10.68	4.01	2.55	1.49	5.28	1.25	1.93	4.12	23.04	4.59	7.38	10.23	11.01	4.72	7.23	2.44
	12	12.18	12.47	11.90	59.86	37.05	6.76	0.69	1.41	1.90	1.27	1.42	1.87	29.54	27.67	6.10	8.03	8.37	5.32	4.89	5.62	11.56
	13	8.03	8.87	7.18	30.99	34.97	3.01	0.97	1.56	1.16	1.92	0.54	4.75	22.07	14.94	3.02	4.41	5.67	5.46	2.94	2.42	3.67
**Quarter**	1	4.01	3.73	4.29	0.00	21.34	3.67	1.08	0.86	0.99	1.89	1.56	2.18	0.00	15.88	3.11	3.67	5.49	4.40	3.26	0.87	1.97
	2	12.20	12.80	11.61	11.29	87.91	10.07	0.79	0.65	1.64	1.37	1.46	0.00	9.80	65.07	6.38	4.31	4.23	4.77	4.55	1.82	3.56
	3	13.74	13.24	14.24	24.57	74.13	9.94	1.82	1.39	1.43	1.99	2.42	1.45	28.07	57.68	7.38	4.73	7.80	7.58	6.44	5.28	3.21
	4	10.64	11.70	9.58	43.28	42.92	6.40	2.02	1.64	1.40	2.42	1.45	3.76	20.29	23.46	5.01	6.77	8.17	7.62	4.44	5.01	5.47
**Year**	15/16	10.16	10.39	9.92	20.23	56.32	7.50	1.44	1.14	1.37	1.93	1.72	1.89	14.65	40.20	5.46	4.91	6.45	6.12	4.67	3.28	3.59
**Episodes**	F/N	3570.00	1418.00	2152.00	40.00	789.00	253.00	55.00	95.00	104.00	45.00	28.00	9.00	29.00	524.00	180.00	182.00	543.00	464.00	135.00	62.00	33.00

Mean weekly incidence rate per 100,000 Persons.

Acute Laryngitis/Tracheitis (ICD10: J04)

			All ages			Male								Female							
			Male & Female	Male	Female	<1yr	1-4yrs	5-14yrs	15-24yrs	25-44yrs	45-64yrs	65-74yrs	75-84yrs	85+yrs	<1yr	1-4yrs	5-14yrs	15-24yrs	25-44yrs	45-64yrs	65-74yrs	75-84yrs	85+yrs
**North**	Quarter	1	4.15	4.38	3.93	0.00	26.30	3.16	1.20	0.93	0.77	1.33	2.29	3.40	0.00	8.81	6.41	2.47	7.00	4.58	3.44	0.93	1.76
		2	11.90	13.81	10.00	10.37	97.01	8.18	1.32	1.15	1.10	1.27	3.87	0.00	0.00	60.07	6.86	1.64	6.02	4.17	4.42	2.00	4.81
		3	13.68	13.76	13.60	24.35	83.95	7.97	1.59	0.56	1.63	1.57	2.25	0.00	37.89	45.21	5.18	4.10	8.23	9.45	6.28	6.02	0.00
		4	11.18	11.22	11.14	39.43	45.74	4.70	1.16	0.91	0.83	1.10	2.72	4.35	33.87	20.13	6.72	6.69	7.96	7.42	6.65	6.19	4.61
	Year	15/16	10.25	10.80	9.69	18.93	62.92	5.98	1.31	0.89	1.08	1.31	2.78	1.98	18.24	33.30	6.30	3.78	7.31	6.42	5.22	3.83	2.83
	Episodes	F/N	1,102.00	428.00	674.00	13.00	260.00	61.00	18.00	21.00	27.00	12.00	13.00	3.00	12.00	132.00	61.00	56.00	169.00	157.00	53.00	25.00	9.00
**South**	Quarter	1	4.12	3.59	4.66	0.00	22.09	2.23	2.18	0.00	1.57	0.56	1.04	2.60	0.00	19.40	2.29	3.93	5.74	5.06	1.60	2.57	1.33
		2	16.58	17.17	15.99	34.78	105.71	10.18	0.00	0.66	1.25	0.00	1.97	0.00	39.20	73.81	6.27	6.27	3.92	6.25	4.54	0.82	2.78
		3	17.55	17.63	17.46	38.12	95.08	10.87	3.13	3.00	1.87	1.10	2.94	2.54	44.55	72.81	10.36	3.78	7.11	6.67	4.08	4.89	2.90
		4	11.84	13.16	10.52	55.73	44.37	11.11	1.85	1.74	1.70	1.01	0.95	0.00	18.73	33.70	8.32	8.35	7.54	7.68	5.20	3.78	1.36
	Year	15/16	12.51	12.89	12.12	32.60	66.39	8.65	1.79	1.36	1.60	0.67	1.71	1.26	25.49	49.62	6.84	5.64	6.10	6.44	3.88	3.03	2.08
	Episodes	F/N	930.00	384.00	546.00	13.00	212.00	72.00	17.00	25.00	31.00	5.00	7.00	2.00	9.00	149.00	54.00	50.00	109.00	124.00	31.00	14.00	6.00
**London**	Quarter	1	3.37	3.29	3.45	0.00	18.34	4.96	0.00	1.03	0.64	4.59	0.00	0.00	0.00	10.00	0.00	3.75	4.98	4.85	3.97	0.00	3.46
		2	8.40	9.02	7.79	0.00	65.35	10.81	1.32	0.33	2.00	1.36	0.00	0.00	0.00	41.57	5.89	3.56	4.26	3.33	6.03	1.77	3.66
		3	6.92	6.74	7.09	8.36	37.14	6.88	1.98	0.86	0.85	2.31	2.32	0.00	6.00	24.76	1.99	4.68	5.59	6.89	9.18	1.55	3.22
		4	6.63	6.86	6.41	13.05	25.55	4.19	1.82	1.63	0.48	4.27	0.00	10.71	12.91	9.58	1.30	5.62	5.98	7.39	2.78	5.84	6.25
	Year	15/16	6.33	6.48	6.19	5.50	36.39	6.66	1.29	0.98	0.98	3.16	0.57	2.83	4.88	21.25	2.28	4.43	5.21	5.65	5.44	2.36	4.19
	Episodes	F/N	647.00	256.00	391.00	4.00	138.00	51.00	10.00	27.00	13.00	10.00	1.00	2.00	4.00	77.00	17.00	36.00	149.00	77.00	20.00	6.00	5.00
**Midlands And East**	Quarter	1	4.40	3.67	5.14	0.00	18.61	4.32	0.93	1.46	0.99	1.06	2.89	2.72	0.00	25.32	3.74	4.54	4.23	3.10	4.05	0.00	1.31
		2	11.93	11.19	12.66	0.00	83.58	11.11	0.55	0.46	2.22	2.83	0.00	0.00	0.00	84.82	6.50	5.75	2.72	5.32	3.21	2.67	2.97
		3	16.81	14.81	18.81	27.44	80.34	14.05	0.57	1.13	1.34	2.97	2.18	3.27	23.83	87.94	12.00	6.34	10.27	7.31	6.22	8.66	6.71
		4	12.92	15.57	10.27	64.90	56.03	5.60	3.26	2.27	2.60	3.30	2.14	0.00	15.66	30.43	3.70	6.43	11.21	7.97	3.15	4.25	9.65
	Year	15/16	11.54	11.39	11.69	23.87	59.57	8.71	1.36	1.35	1.80	2.56	1.81	1.47	9.98	56.62	6.43	5.78	7.19	5.96	4.14	3.90	5.25
	Episodes	F/N	891.00	350.00	541.00	10.00	179.00	69.00	10.00	22.00	33.00	18.00	7.00	2.00	4.00	166.00	48.00	40.00	116.00	106.00	31.00	17.00	13.00

Mean weekly incidence rate per 100,000 Persons.

Acute Otitis Media (ICD10: H650-H651,H660,H669)

		All ages			Male									Female								
		Male & Female	Male	Female	<1yr	1-4yrs	5-14yrs	15-24yrs	25-44yrs	45-64yrs	65-74yrs	75-84yrs	85+yrs	<1yr	1-4yrs	5-14yrs	15-24yrs	25-44yrs	45-64yrs	65-74yrs	75-84yrs	85+yrs
**4 weekly**	1	31.04	31.48	30.61	0.00	191.98	60.53	6.08	6.66	6.35	7.04	4.66	0.00	0.00	157.75	63.90	12.21	13.64	8.94	6.38	10.92	1.75
	2	23.25	23.13	23.36	0.00	134.76	39.63	6.52	6.58	5.13	3.72	4.39	7.44	0.00	129.41	47.09	10.67	6.85	7.38	5.36	1.38	2.11
	3	21.79	22.28	21.30	0.00	134.17	32.59	7.91	6.33	6.32	4.74	3.65	4.85	0.00	118.82	32.25	10.08	10.55	6.77	4.54	3.49	5.17
	4	18.61	18.36	18.86	0.00	102.64	30.37	7.10	7.51	6.19	5.33	1.67	4.42	0.00	78.82	35.34	17.78	12.02	10.02	8.46	4.87	2.42
	5	21.83	22.88	20.77	0.00	125.79	37.83	9.21	6.73	5.37	10.95	3.71	6.34	16.67	91.17	40.65	11.70	9.93	7.75	2.16	4.34	2.57
	6	35.63	37.68	33.59	40.35	226.36	40.97	9.85	8.18	5.25	2.39	3.99	1.77	22.02	179.17	43.83	19.94	12.03	7.93	9.58	6.86	0.94
	7	43.86	42.16	45.57	43.47	262.12	38.76	6.83	7.06	6.98	6.26	5.16	2.82	67.34	246.86	45.80	16.79	13.23	9.01	5.20	4.96	0.89
	8	65.12	70.80	59.43	103.12	408.36	81.24	12.68	8.32	8.51	8.64	4.05	2.30	32.64	324.40	100.27	20.24	20.77	11.61	7.37	8.76	8.82
	9	48.42	48.03	48.81	102.06	225.38	61.88	14.51	8.59	8.79	7.55	1.57	1.90	89.84	219.10	58.17	24.44	18.03	13.37	6.97	4.78	4.62
	10	67.49	71.29	63.69	127.61	378.14	93.93	10.04	9.20	9.56	6.34	1.43	5.40	101.66	324.88	80.27	19.39	17.82	13.09	8.56	6.04	1.47
	11	71.64	71.29	71.99	170.91	356.18	74.64	9.70	9.58	8.21	5.74	2.41	4.24	180.05	323.21	81.53	20.25	19.20	12.40	5.31	4.46	1.48
	12	63.55	61.78	65.32	141.78	309.66	70.24	10.21	6.71	6.49	5.60	5.33	0.00	166.87	276.13	75.27	20.47	17.82	12.55	8.46	8.51	1.84
	13	48.00	49.61	46.40	164.11	206.33	42.68	12.29	6.81	6.08	3.65	1.59	2.90	125.00	186.69	48.90	13.39	16.69	9.84	7.64	7.33	2.10
**Quarter**	1	25.80	26.08	25.51	0.00	156.58	45.50	6.78	6.54	5.97	5.31	4.27	3.78	0.00	137.05	48.99	11.08	10.60	7.79	5.50	5.70	2.91
	2	26.30	27.02	25.58	12.42	160.46	35.71	8.57	7.11	5.72	6.21	3.15	3.86	19.51	122.51	38.26	15.72	11.50	8.48	6.83	5.28	2.10
	3	55.18	56.44	53.92	85.92	313.55	65.90	11.70	8.60	8.06	7.33	3.05	3.82	67.00	277.38	73.13	21.48	17.31	11.47	7.03	6.37	4.13
	4	62.67	63.61	61.73	163.94	302.98	69.23	10.44	7.91	7.61	5.26	3.08	2.04	148.84	271.66	70.86	18.13	18.26	12.21	7.04	6.58	1.97
**Year**	15/16	42.87	43.67	42.06	67.43	234.71	54.37	9.39	7.54	6.85	6.02	3.38	3.35	60.54	203.46	58.06	16.63	14.49	10.03	6.61	5.99	2.76
**Episodes**	F/N	14656.00	7005.00	7651.00	150.00	3280.00	1853.00	350.00	634.00	512.00	155.00	53.00	18.00	130.00	2726.00	1891.00	619.00	1231.00	730.00	195.00	102.00	27.00

Mean weekly incidence rate per 100,000 Persons.

Acute Otitis Media (ICD10: H650-H651,H660,H669)

			All ages			Male									Female								
			Male & Female	Male	Female	<1yr	1-4yrs	5-14yrs	15-24yrs	25-44yrs	45-64yrs	65-74yrs	75-84yrs	85+yrs	<1yr	1-4yrs	5-14yrs	15-24yrs	25-44yrs	45-64yrs	65-74yrs	75-84yrs	85+yrs
**North**	Quarter	1	29.67	27.70	31.64	0.00	167.94	47.57	5.02	8.20	7.50	4.30	2.29	6.51	0.00	178.83	56.93	9.60	11.46	8.33	11.22	6.62	1.78
		2	26.53	26.21	26.85	12.80	157.07	32.80	7.90	6.73	6.23	5.79	4.41	2.17	12.67	130.27	42.33	16.16	14.35	8.26	6.26	7.44	3.85
		3	51.86	49.76	53.96	60.06	279.26	67.26	11.53	7.81	7.52	8.56	3.51	2.34	90.55	263.18	71.01	17.79	18.83	12.69	7.88	3.68	0.00
		4	62.45	64.61	60.29	158.03	317.52	69.75	8.32	10.85	7.18	4.82	2.71	2.28	139.19	273.38	64.15	18.88	19.95	11.11	8.47	4.28	3.19
	Year	15/16	43.00	42.50	43.51	59.62	232.09	54.64	8.20	8.45	7.11	5.85	3.22	3.30	62.08	212.58	58.71	15.67	16.22	10.12	8.46	5.48	2.22
	Episodes	F/N	4,412.00	2,070.00	2,342.00	45.00	929.00	536.00	114.00	202.00	167.00	55.00	17.00	5.00	41.00	796.00	547.00	220.00	379.00	239.00	81.00	32.00	7.00
**South**	Quarter	1	23.93	25.85	22.02	0.00	157.69	48.22	6.00	5.56	6.22	2.88	3.27	2.78	0.00	114.25	43.47	13.24	9.66	5.78	3.20	2.68	5.87
		2	27.70	31.36	24.04	23.10	183.85	39.16	7.94	6.76	6.49	1.65	2.96	10.32	0.00	124.53	44.35	18.64	10.06	6.34	7.05	4.04	1.38
		3	61.95	61.84	62.06	92.57	363.12	66.90	9.74	9.78	8.16	5.26	0.98	0.00	110.07	311.89	72.84	24.45	17.93	8.81	5.75	2.37	4.42
		4	68.02	71.97	64.08	214.03	319.32	77.98	13.77	5.70	5.47	4.37	4.54	2.57	146.34	300.41	71.93	16.01	20.56	10.54	5.11	4.47	1.31
	Year	15/16	45.83	48.21	43.45	84.91	257.19	58.44	9.45	6.92	6.56	3.56	2.97	3.89	65.66	214.42	58.41	18.05	14.67	7.92	5.28	3.41	3.21
	Episodes	F/N	3,514.00	1,737.00	1,777.00	41.00	823.00	485.00	91.00	127.00	126.00	26.00	12.00	6.00	31.00	646.00	457.00	160.00	264.00	151.00	42.00	17.00	9.00
**London**	Quarter	1	21.54	22.88	20.20	0.00	123.85	45.84	8.04	5.27	5.28	7.13	4.64	5.84	0.00	102.94	40.39	9.87	7.63	10.54	3.48	6.97	0.00
		2	24.93	24.05	25.81	13.76	141.40	31.63	8.37	6.54	6.45	8.28	0.00	0.00	47.79	106.42	35.71	11.76	10.50	11.43	6.93	1.77	0.00
		3	42.81	43.53	42.10	58.16	238.78	61.44	6.37	7.12	8.05	9.68	2.13	0.00	13.05	231.73	66.72	11.40	14.26	12.36	6.05	12.81	10.49
		4	45.66	45.44	45.89	108.92	218.24	51.74	6.29	6.02	9.43	6.43	1.91	0.00	109.76	187.25	58.51	13.34	12.78	14.27	8.33	8.77	0.00
	Year	15/16	33.96	34.19	33.73	46.41	181.28	47.74	7.25	6.23	7.34	7.85	2.17	1.43	43.91	157.65	50.48	11.62	11.32	12.19	6.24	7.60	2.57
	Episodes	F/N	3,153.00	1,485.00	1,668.00	33.00	720.00	383.00	51.00	164.00	104.00	25.00	4.00	1.00	29.00	615.00	398.00	93.00	325.00	162.00	24.00	19.00	3.00
**Midlands And East**	Quarter	1	28.05	27.90	28.19	0.00	176.85	40.39	8.06	7.12	4.87	6.92	6.86	0.00	0.00	152.18	55.17	11.61	13.65	6.51	4.09	6.53	4.01
		2	26.04	26.48	25.60	0.00	159.51	39.25	10.09	8.40	3.71	9.14	5.25	2.93	17.56	128.81	30.65	16.32	11.08	7.88	7.09	7.88	3.16
		3	64.10	70.63	57.58	132.90	373.05	67.99	19.16	9.67	8.51	5.82	5.58	12.95	54.34	302.71	81.96	32.29	18.21	12.01	8.44	6.63	1.63
		4	74.54	72.42	76.66	174.79	356.83	77.44	13.36	9.06	8.35	5.44	3.14	3.32	200.08	325.62	88.86	24.31	19.75	12.92	6.27	8.78	3.37
	Year	15/16	48.68	49.79	47.57	78.77	268.27	56.67	12.68	8.57	6.40	6.80	5.17	4.77	70.49	229.18	64.63	21.19	15.75	9.89	6.47	7.48	3.05
	Episodes	F/N	3,577.00	1,713.00	1,864.00	31.00	808.00	449.00	94.00	141.00	115.00	49.00	20.00	6.00	29.00	669.00	489.00	146.00	263.00	178.00	48.00	34.00	8.00

Mean weekly incidence rate per 100,000 Persons.

Sinusitis (ICD10: J01)

		All ages			Male								Female							
		Male & Female	Male	Female	<1yr	1-4yrs	5-14yrs	15-24yrs	25-44yrs	45-64yrs	65-74yrs	75-84yrs	85+yrs	<1yr	1-4yrs	5-14yrs	15-24yrs	25-44yrs	45-64yrs	65-74yrs	75-84yrs	85+yrs
**4 weekly**	1	12.28	7.02	17.55	0.00	0.70	2.68	6.11	14.60	13.82	15.37	2.87	7.02	0.00	3.29	2.67	18.32	41.05	36.74	21.18	26.15	8.52
	2	7.68	5.95	9.40	0.00	0.00	1.22	6.36	12.16	11.22	8.23	6.92	7.44	0.00	0.00	1.19	7.34	26.91	23.61	11.51	11.96	2.11
	3	8.52	4.88	12.16	0.00	0.00	0.39	3.38	9.02	10.06	11.12	7.94	2.05	0.00	2.38	1.84	10.69	31.16	23.55	18.78	15.88	5.16
	4	8.60	5.68	11.52	0.00	0.00	0.40	2.95	9.22	8.96	10.83	11.99	6.75	0.00	0.00	0.83	8.02	22.94	25.57	21.38	14.18	10.72
	5	10.31	6.23	14.39	0.00	0.00	3.42	5.97	9.32	8.64	11.77	10.68	6.26	0.00	0.99	2.18	18.10	32.47	28.70	28.56	13.83	4.68
	6	13.72	8.64	18.81	0.00	0.00	2.76	8.74	13.18	14.90	17.82	10.89	9.43	0.00	0.80	5.45	16.26	43.28	42.42	26.25	20.16	14.70
	7	12.56	8.10	17.03	0.00	0.00	1.22	8.02	16.39	16.39	13.00	9.04	8.86	0.00	1.13	2.05	18.24	39.61	34.22	25.84	20.27	11.88
	8	15.97	9.78	22.16	0.00	0.00	4.57	8.91	20.12	18.37	22.13	9.77	4.13	0.00	1.54	6.15	17.66	60.12	51.89	39.29	18.84	3.94
	9	17.20	11.37	23.04	0.00	1.57	2.24	9.16	19.59	22.30	18.59	10.71	18.16	0.00	1.52	3.10	32.88	57.32	49.85	34.34	17.80	10.55
	10	20.85	11.58	30.13	0.00	1.51	5.93	9.51	21.42	23.36	22.20	10.77	9.49	0.00	0.76	9.20	29.82	68.24	71.58	50.94	25.04	15.58
	11	19.80	12.09	27.50	0.00	0.77	5.41	9.23	20.99	23.08	29.59	12.89	6.88	0.00	0.00	7.08	23.80	64.13	71.63	43.58	25.73	11.54
	12	19.73	11.91	27.55	0.00	1.38	7.76	11.93	22.06	24.51	23.74	10.80	5.02	0.00	2.23	6.18	27.97	55.36	61.11	50.20	29.83	15.06
	13	14.55	8.05	21.06	3.26	0.00	2.76	6.99	16.68	15.68	19.27	7.78	0.00	0.00	0.00	2.06	20.35	44.71	45.27	38.28	21.37	17.49
**Quarter**	1	9.71	6.03	13.38	0.00	0.27	1.53	5.35	12.13	11.87	11.86	5.68	5.62	0.00	2.00	1.96	12.59	33.65	28.64	17.46	18.62	5.51
	2	10.84	6.79	14.90	0.00	0.00	2.02	6.06	10.85	11.38	13.11	10.77	6.90	0.00	0.90	2.87	13.56	33.01	32.32	25.43	16.43	9.53
	3	16.41	10.43	22.38	0.00	0.71	3.28	8.82	19.59	19.56	19.58	11.19	11.10	0.00	1.18	4.00	25.51	55.08	48.94	35.94	20.26	10.54
	4	18.50	10.70	26.29	0.93	0.83	5.49	9.44	20.00	21.61	23.63	9.70	4.70	0.00	0.64	6.27	24.61	57.68	62.34	45.48	25.09	14.54
**Year**	15/16	13.95	8.53	19.37	0.25	0.46	3.13	7.46	15.73	16.21	17.17	9.34	7.04	0.00	1.17	3.82	19.17	45.10	43.42	31.35	20.20	10.12
**Episodes**	F/N	13006.00	3636.00	9370.00	1.00	8.00	109.00	283.00	1322.00	1248.00	494.00	137.00	34.00	0.00	17.00	124.00	726.00	3795.00	3314.00	937.00	362.00	95.00

Mean weekly incidence rate per 100,000 Persons.

Acute Sinusitis (ICD10: J01)

			All ages			Male								Female							
			Male & Female	Male	Female	<1yr	1-4yrs	5-14yrs	15-24yrs	25-44yrs	45-64yrs	65-74yrs	75-84yrs	85+yrs	<1yr	1-4yrs	5-14yrs	15-24yrs	25-44yrs	45-64yrs	65-74yrs	75-84yrs	85+yrs
**North**	Quarter	1	11.19	6.60	15.79	0.00	0.00	1.91	4.48	13.90	14.39	13.71	4.39	6.58	0.00	0.00	1.31	17.01	43.10	35.32	15.83	24.07	5.46
		2	11.02	7.30	14.75	0.00	0.00	1.20	6.71	10.60	11.16	17.87	12.70	5.43	0.00	0.99	1.14	9.87	35.36	34.24	24.39	15.39	11.35
		3	17.51	10.16	24.86	0.00	2.86	3.75	9.13	17.56	22.04	21.70	14.44	0.00	0.00	0.00	3.42	28.43	56.83	55.26	44.95	23.13	11.76
		4	18.78	10.50	27.06	0.00	3.32	5.12	6.99	22.24	22.65	24.00	8.07	2.14	0.00	2.54	4.24	22.83	64.28	61.08	47.17	28.45	12.97
	Year	15/16	14.71	8.68	20.74	0.00	1.58	3.04	6.83	16.19	17.66	19.41	9.87	3.51	0.00	0.91	2.56	19.60	50.16	46.75	33.35	22.87	10.43
	Episodes	F/N	4,341.00	1,206.00	3,135.00	0.00	7.00	31.00	95.00	396.00	438.00	185.00	49.00	5.00	0.00	4.00	26.00	276.00	1,164.00	1,145.00	350.00	138.00	32.00
**South**	Quarter	1	9.36	5.59	13.12	0.00	0.00	0.48	6.82	12.36	10.31	7.85	7.34	5.17	0.00	1.61	4.51	11.85	30.15	29.90	16.39	15.07	8.63
		2	10.48	7.35	13.62	0.00	0.00	1.96	6.98	9.74	11.75	15.66	9.92	10.14	0.00	0.00	4.16	14.06	34.41	30.72	19.65	13.94	5.64
		3	15.35	10.67	20.03	0.00	0.00	1.44	9.98	17.89	20.60	20.32	10.04	15.75	0.00	0.00	4.26	27.91	53.60	40.75	26.26	21.71	5.79
		4	17.96	11.29	24.62	0.00	0.00	8.37	8.87	20.23	21.17	27.63	10.13	5.21	0.00	0.00	7.18	24.58	56.75	63.72	40.28	21.00	8.08
	Year	15/16	13.37	8.77	17.98	0.00	0.00	3.16	8.18	15.15	16.06	18.05	9.37	9.00	0.00	0.39	5.07	19.69	43.97	41.69	25.92	17.99	7.05
	Episodes	F/N	2,993.00	876.00	2,117.00	0.00	0.00	27.00	78.00	276.00	306.00	137.00	38.00	14.00	0.00	1.00	39.00	174.00	786.00	801.00	208.00	88.00	20.00
**London**	Quarter	1	8.01	5.78	10.24	0.00	0.00	1.16	4.05	10.06	11.65	12.38	6.91	5.79	0.00	5.06	0.60	7.16	26.76	22.68	13.24	16.63	0.00
		2	10.57	6.50	14.64	0.00	0.00	2.86	5.75	9.72	10.91	9.15	14.02	6.12	0.00	1.22	2.96	13.32	26.66	28.07	27.25	25.00	7.27
		3	14.84	9.09	20.59	0.00	0.00	4.20	8.16	17.64	14.67	11.85	6.74	18.55	0.00	4.71	3.75	17.87	40.81	47.51	34.66	18.44	17.55
		4	15.97	8.79	23.15	3.72	0.00	3.34	9.96	14.31	17.68	17.12	7.64	5.30	0.00	0.00	5.17	18.57	43.21	51.17	48.24	23.39	18.60
	Year	15/16	12.41	7.56	17.27	0.98	0.00	2.90	7.04	12.96	13.80	12.71	8.81	8.87	0.00	2.69	3.16	14.31	34.53	37.62	31.18	20.91	11.00
	Episodes	F/N	2,532.00	687.00	1,845.00	1.00	0.00	24.00	52.00	351.00	195.00	42.00	16.00	6.00	0.00	10.00	26.00	119.00	994.00	514.00	119.00	50.00	13.00
**Midlands And East**	Quarter	1	10.28	6.17	14.39	0.00	1.08	2.56	6.04	12.22	11.11	13.51	4.07	4.93	0.00	1.32	1.41	14.36	34.61	26.67	24.40	18.72	7.96
		2	11.29	6.00	16.58	0.00	0.00	2.07	4.80	13.36	11.69	9.74	6.43	5.92	0.00	1.39	3.23	17.00	35.63	36.24	30.43	11.38	13.88
		3	17.92	11.79	24.05	0.00	0.00	3.75	8.01	25.28	20.94	24.45	13.55	10.10	0.00	0.00	4.57	27.81	69.09	52.23	37.90	17.78	7.08
		4	21.29	12.24	30.34	0.00	0.00	5.13	11.95	23.24	24.92	25.78	12.94	6.18	0.00	0.00	8.50	32.46	66.49	73.38	46.22	27.51	18.49
	Year	15/16	15.31	9.11	21.51	0.00	0.27	3.41	7.78	18.61	17.31	18.51	9.32	6.77	0.00	0.67	4.51	23.09	51.74	47.62	34.96	19.01	11.98
	Episodes	F/N	3,140.00	867.00	2,273.00	0.00	1.00	27.00	58.00	299.00	309.00	130.00	34.00	9.00	0.00	2.00	33.00	157.00	851.00	854.00	260.00	86.00	30.00

Mean weekly incidence rate per 100,000 Persons.

Acute Tonsillitis/Pharyngitis (ICD10: J02-J03)

		All ages			Male									Female								
		Male & Female	Male	Female	<1yr	1-4yrs	5-14yrs	15-24yrs	25-44yrs	45-64yrs	65-74yrs	75-84yrs	85+yrs	<1yr	1-4yrs	5-14yrs	15-24yrs	25-44yrs	45-64yrs	65-74yrs	75-84yrs	85+yrs
**4 weekly**	1	48.27	41.78	54.76	0.00	158.13	95.46	54.16	32.31	11.67	8.16	5.91	10.26	0.00	134.04	119.29	110.55	73.92	23.04	18.07	10.20	3.72
	2	39.14	33.26	45.03	0.00	130.31	90.91	38.10	24.34	9.04	3.09	3.55	0.00	0.00	105.92	95.89	104.91	55.40	22.04	12.95	5.62	2.52
	3	41.66	36.62	46.70	0.00	146.94	74.26	49.20	30.05	11.36	10.18	5.32	2.28	0.00	123.06	105.32	86.99	64.28	20.64	11.65	5.83	2.53
	4	29.66	25.95	33.37	0.00	92.95	45.37	43.73	26.66	8.02	7.79	4.29	4.75	0.00	76.89	38.06	91.16	51.81	16.33	8.98	12.54	4.56
	5	35.26	30.93	39.59	17.12	104.40	58.56	49.75	25.31	8.23	7.11	3.71	4.19	0.00	99.79	73.01	95.58	47.53	13.91	9.11	8.10	9.32
	6	46.13	40.59	51.66	0.00	183.52	80.26	45.77	25.08	9.93	7.33	6.81	6.64	11.73	158.27	99.29	98.51	55.77	15.81	12.81	8.03	4.76
	7	51.78	44.70	58.86	8.00	202.06	88.30	54.26	24.82	8.01	9.28	7.60	0.00	12.16	201.48	95.43	115.38	57.24	20.01	17.30	6.77	3.93
	8	59.40	54.82	63.99	41.03	215.75	110.25	64.00	31.83	9.80	8.27	6.35	6.11	15.61	182.63	126.67	123.58	66.15	21.88	15.90	15.59	7.90
	9	49.27	43.53	55.02	11.27	172.19	77.18	61.30	31.21	10.95	12.30	12.69	2.66	24.40	121.98	114.91	106.42	71.01	23.48	15.32	11.26	6.40
	10	73.60	67.27	79.92	73.26	268.07	137.20	58.59	33.82	10.25	7.20	3.72	13.32	37.24	256.19	159.05	129.95	81.99	31.83	14.09	5.32	3.65
	11	79.02	71.69	86.35	74.03	284.26	136.66	80.71	42.14	13.86	9.19	4.36	0.00	57.05	253.85	169.88	144.64	94.43	32.02	14.60	9.28	1.41
	12	67.62	56.10	79.14	51.57	209.57	114.13	62.56	35.18	14.94	7.14	4.57	5.22	47.45	250.53	161.23	117.99	81.97	25.70	13.60	11.29	2.48
	13	56.14	48.92	63.37	54.62	169.48	91.97	65.47	30.85	10.47	7.65	7.70	2.09	64.38	156.54	115.19	116.06	66.68	27.17	14.85	7.60	1.83
**Quarter**	1	43.43	37.57	49.28	0.00	146.13	87.54	47.69	29.16	10.77	7.22	5.00	4.65	0.00	122.01	107.79	101.57	65.25	21.99	14.52	7.45	2.98
	2	37.70	33.03	42.36	5.27	132.50	62.14	46.08	25.60	8.80	7.29	4.79	4.79	3.61	115.88	70.26	96.40	52.57	15.96	10.28	9.88	6.41
	3	56.54	50.88	62.20	26.74	205.06	101.91	60.92	30.07	9.76	9.96	8.14	5.37	20.93	177.92	121.94	117.56	66.93	22.45	15.97	10.01	6.06
	4	69.03	60.51	77.56	64.82	230.05	116.20	68.11	35.99	12.61	7.76	5.64	3.41	54.36	229.51	150.87	126.83	81.78	29.52	14.64	8.89	1.63
**Year**	15/16	52.00	45.78	58.22	24.97	179.41	92.41	55.93	30.31	10.52	8.05	5.89	4.53	20.38	162.62	113.44	110.89	66.92	22.61	13.87	9.05	4.22
**Episodes**	F/N	29321.00	11420.00	17901.00	59.00	2518.00	3147.00	2100.00	2528.00	764.00	197.00	84.00	23.00	45.00	2222.00	3709.00	4127.00	5593.00	1646.00	369.00	150.00	40.00

Mean weekly incidence rate per 100,000 Persons.

Acute Tonsillitis/Pharyngitis (ICD10: J02-J03)

			All ages			Female									Male								
			Male & Female	Male	Female	<1yr	1-4yrs	5-14yrs	15-24yrs	25-44yrs	45-64yrs	65-74yrs	75-84yrs	85+yrs	<1yr	1-4yrs	5-14yrs	15-24yrs	25-44yrs	45-64yrs	65-74yrs	75-84yrs	85+yrs
**North**	Quarter	1	45.12	36.96	53.27	0.00	110.26	117.59	129.35	73.87	24.46	11.96	8.42	3.54	0.00	136.31	78.87	59.57	34.71	11.14	6.12	5.89	0.00
		2	37.61	28.84	46.38	0.00	140.28	69.78	116.52	54.67	16.05	10.44	2.71	6.97	0.00	115.50	54.75	44.20	20.84	7.66	4.16	7.87	4.62
		3	53.70	45.93	61.46	7.82	194.03	120.06	110.04	67.48	17.01	16.35	10.81	9.57	23.84	179.28	98.34	54.77	28.23	9.55	4.92	7.79	6.64
		4	66.54	60.49	72.59	24.03	219.28	158.16	121.83	79.97	28.52	11.11	7.17	3.26	72.40	225.27	121.47	71.29	33.60	8.84	6.09	5.44	0.00
	Year	15/16	51.04	43.38	58.69	8.26	166.97	117.18	119.48	69.21	21.64	12.44	7.28	5.79	24.97	165.24	88.98	57.72	29.43	9.29	5.34	6.72	2.76
	Episodes	F/N	8,836.00	3,288.00	5,548.00	6.00	635.00	1,112.00	1,558.00	1,547.00	507.00	122.00	43.00	18.00	18.00	652.00	877.00	755.00	683.00	216.00	50.00	32.00	5.00
**South**	Quarter	1	41.76	37.52	46.01	0.00	115.06	96.30	103.47	63.89	17.16	7.56	6.10	4.53	0.00	149.42	83.13	51.92	27.38	10.47	6.12	6.60	2.60
		2	33.36	29.89	36.83	0.00	80.89	69.67	95.18	54.56	11.29	7.00	11.39	1.49	0.00	120.66	55.72	46.36	24.38	7.58	5.89	5.84	2.58
		3	52.41	46.84	57.99	45.89	128.59	112.61	128.36	64.22	20.10	13.86	6.88	1.40	35.21	203.00	72.40	55.11	31.41	6.63	7.95	7.11	2.73
		4	62.91	54.17	71.66	42.57	212.17	132.00	126.67	89.40	25.15	10.18	6.80	0.00	55.49	203.45	96.36	67.26	37.92	10.69	8.59	5.38	2.39
	Year	15/16	47.90	42.33	53.47	22.50	135.65	103.20	113.67	68.42	18.55	9.66	7.77	1.82	23.29	169.78	77.27	55.39	30.42	8.88	7.17	6.22	2.57
	Episodes	F/N	6,466.00	2,530.00	3,936.00	10.00	407.00	809.00	1,016.00	1,221.00	354.00	76.00	38.00	5.00	11.00	538.00	643.00	532.00	554.00	169.00	54.00	25.00	4.00
**London**	Quarter	1	45.01	39.18	50.84	0.00	150.91	122.84	67.96	59.82	25.52	25.20	5.34	0.00	0.00	168.70	91.52	35.24	27.63	13.03	8.42	2.27	5.78
		2	41.30	38.71	43.89	14.43	123.78	71.63	73.78	47.36	19.06	13.95	20.14	10.89	21.07	142.58	73.43	49.97	27.96	11.60	13.34	2.31	6.12
		3	58.38	55.14	61.63	7.14	205.78	122.19	82.33	60.93	29.32	22.42	14.81	9.74	36.22	223.88	112.42	52.40	25.80	9.74	17.26	12.99	5.51
		4	65.13	59.27	70.99	63.34	203.11	153.08	92.28	60.86	32.83	23.20	10.24	0.00	59.96	225.81	119.22	56.58	31.55	16.94	8.54	9.58	5.29
	Year	15/16	52.70	48.29	57.11	22.02	171.50	118.11	79.34	57.31	26.80	21.23	12.59	5.06	29.89	190.91	99.53	48.70	28.30	12.91	11.82	6.84	5.67
	Episodes	F/N	7,235.00	2,909.00	4,326.00	16.00	662.00	921.00	639.00	1,612.00	360.00	80.00	30.00	6.00	20.00	749.00	798.00	354.00	753.00	180.00	38.00	13.00	4.00
**Midlands And East**	Quarter	1	41.83	36.64	47.01	0.00	111.80	94.44	105.48	63.44	20.84	13.37	9.92	3.85	0.00	130.07	96.63	44.05	26.94	8.42	8.22	5.24	10.21
		2	38.51	34.68	42.34	0.00	118.58	69.99	100.11	53.67	17.44	9.73	5.30	6.27	0.00	151.28	64.68	43.79	29.22	8.37	5.79	3.15	5.85
		3	61.66	55.62	67.71	22.89	183.30	132.89	149.52	75.08	23.37	11.24	7.56	3.55	11.69	214.06	124.46	81.37	34.83	13.13	9.71	4.68	6.60
		4	81.55	68.11	94.99	87.49	283.46	160.24	166.53	96.88	31.57	14.09	11.34	3.27	71.44	265.69	127.77	77.32	40.88	13.96	7.83	2.17	5.96
	Year	15/16	56.37	49.13	63.62	28.73	176.34	115.25	131.09	72.73	23.46	12.14	8.58	4.21	21.74	191.70	103.84	61.93	33.12	11.03	7.89	3.78	7.13
	Episodes	F/N	6,784.00	2,693.00	4,091.00	13.00	518.00	867.00	914.00	1,213.00	425.00	91.00	39.00	11.00	10.00	579.00	829.00	459.00	538.00	199.00	55.00	14.00	10.00

Mean weekly incidence rate per 100,000 Persons.

Asthma (ICD10: J45-J46)

		All ages			Male									Female								
		Male & Female	Male	Female	<1yr	1-4yrs	5-14yrs	15-24yrs	25-44yrs	45-64yrs	65-74yrs	75-84yrs	85+yrs	<1yr	1-4yrs	5-14yrs	15-24yrs	25-44yrs	45-64yrs	65-74yrs	75-84yrs	85+yrs
**4 weekly**	1	9.52	7.97	11.06	0.00	11.57	26.25	5.98	7.23	6.61	3.92	6.38	3.82	0.00	12.85	20.68	10.56	10.71	12.80	12.30	8.73	10.88
	2	9.68	8.97	10.39	0.00	14.32	23.82	7.47	9.76	6.68	8.95	5.82	3.95	0.00	11.55	16.82	11.92	11.30	10.62	13.64	9.77	7.90
	3	9.95	8.85	11.04	0.00	10.00	15.58	8.36	10.51	7.08	9.24	9.70	9.21	0.00	11.83	15.83	14.38	11.40	12.02	12.57	4.14	17.17
	4	7.93	6.59	9.26	0.00	11.93	12.82	8.93	6.08	6.10	5.46	8.03	0.00	0.00	4.57	10.58	13.67	12.23	11.11	7.54	14.34	9.33
	5	10.85	10.60	11.10	0.00	21.64	24.90	13.42	8.48	8.02	9.25	4.01	5.70	0.00	5.27	15.85	15.06	13.90	16.96	17.13	12.22	3.52
	6	12.65	10.61	14.69	0.00	20.36	25.74	10.69	11.50	10.29	8.20	8.73	0.00	0.00	9.73	18.87	22.93	19.85	22.50	10.97	17.72	9.67
	7	13.18	11.28	15.07	0.00	16.44	22.47	11.25	11.55	10.37	12.31	12.30	4.85	0.00	12.48	17.26	22.20	19.20	21.28	16.08	12.56	14.60
	8	14.89	12.01	17.78	0.00	23.68	28.91	9.18	9.51	9.07	11.88	12.17	3.70	0.00	23.35	23.27	22.25	18.35	17.94	21.57	15.49	17.79
	9	12.98	11.63	14.34	0.00	19.66	17.86	10.11	10.54	11.73	10.52	15.19	9.08	0.00	13.08	12.38	12.56	16.70	22.06	18.93	22.46	10.86
	10	14.38	13.48	15.28	0.00	25.26	30.09	10.35	11.47	13.21	11.76	10.47	8.67	0.00	10.94	22.11	21.07	19.89	22.93	17.95	13.51	9.09
	11	13.04	10.66	15.43	0.00	16.70	24.65	10.67	7.84	10.09	9.69	9.77	6.51	0.00	13.96	17.34	17.33	19.62	19.61	22.00	11.82	17.19
	12	9.87	6.57	13.17	0.00	11.80	16.66	6.55	8.23	6.20	7.28	2.37	0.00	0.00	9.10	15.08	16.22	15.42	18.71	14.40	14.88	14.68
	13	10.44	8.90	11.98	3.17	23.23	18.30	7.73	6.01	8.87	7.44	5.34	0.00	0.00	7.96	14.01	13.78	14.45	13.68	17.76	13.68	12.51
**Quarter**	1	9.70	8.55	10.85	0.00	11.94	22.22	7.17	9.02	6.78	7.10	7.23	5.52	0.00	12.14	18.00	12.16	11.11	11.89	12.80	7.64	11.90
	2	10.67	9.25	12.09	0.00	17.66	20.41	11.04	9.06	8.41	7.92	6.39	2.38	0.00	6.60	15.90	17.28	15.43	17.86	12.15	14.63	8.96
	3	14.02	12.24	15.81	0.00	21.66	25.53	9.38	10.86	11.00	10.78	14.14	6.78	0.00	16.42	17.61	20.34	18.60	19.97	18.94	17.26	13.17
	4	11.41	9.28	13.55	0.91	17.97	20.89	9.28	7.56	8.82	9.43	6.18	2.50	0.00	10.17	16.52	15.66	16.87	18.24	18.09	12.87	13.52
**Year**	15/16	11.45	9.82	13.08	0.24	17.32	22.24	9.22	9.09	8.75	8.82	8.44	4.26	0.00	11.31	17.00	16.35	15.53	17.01	15.54	13.09	11.92
**Episodes**	F/N	7883.00	3177.00	4706.00	1.00	247.00	763.00	349.00	772.00	661.00	236.00	127.00	21.00	0.00	152.00	549.00	638.00	1313.00	1267.00	443.00	235.00	109.00

Mean weekly incidence rate per 100,000 Persons.

Asthma (ICD10: J45 - J46)

			All ages			Male								Female							
			Male & Female	Male	Female	<1yr	1-4yrs	5-14yrs	15-24yrs	25-44yrs	45-64yrs	65-74yrs	75-84yrs	85+yrs	<1yr	1-4yrs	5-14yrs	15-24yrs	25-44yrs	45-64yrs	65-74yrs	75-84yrs	85+yrs
**North**	Quarter	1	8.23	7.02	9.44	0.00	11.60	18.34	7.07	8.42	5.39	3.16	5.85	3.35	0.00	3.29	16.75	14.15	11.72	10.20	8.57	7.63	12.66
		2	11.74	10.76	12.72	0.00	21.93	22.34	12.09	11.41	6.32	9.40	6.31	7.01	0.00	7.92	13.62	22.70	16.13	18.36	12.33	16.07	7.34
		3	14.43	12.30	16.56	0.00	26.60	28.63	8.73	10.37	11.76	9.32	10.95	4.30	0.00	21.00	17.16	22.10	23.40	18.72	19.12	17.39	10.16
		4	12.54	11.37	13.70	3.62	22.87	26.29	8.66	9.30	8.99	10.17	10.18	2.28	0.00	13.56	16.36	17.77	19.18	19.00	15.08	15.72	6.61
	Year	15/16	11.75	10.38	13.12	0.96	20.79	23.94	9.13	9.86	8.13	8.05	8.36	4.20	0.00	11.48	15.98	19.15	17.64	16.62	13.80	14.23	9.14
	Episodes	F/N	2,561.00	1,015.00	1,546.00	1.00	85.00	237.00	123.00	239.00	202.00	80.00	41.00	7.00	0.00	46.00	149.00	268.00	418.00	406.00	143.00	89.00	27.00
**South**	Quarter	1	9.56	8.48	10.64	0.00	12.91	21.99	5.29	9.26	7.20	7.27	7.41	4.96	0.00	23.03	13.51	6.55	11.67	12.81	15.31	4.14	8.71
		2	10.08	8.37	11.78	0.00	18.22	17.63	8.84	7.05	8.62	7.41	5.06	2.50	0.00	5.52	14.48	15.15	18.71	18.15	8.69	13.96	11.37
		3	14.68	11.88	17.49	0.00	17.70	24.48	13.19	12.93	11.32	12.92	11.80	2.54	0.00	18.72	18.24	23.10	19.12	22.29	22.48	21.17	12.27
		4	12.18	10.22	14.14	0.00	22.03	20.81	8.75	5.90	9.76	9.66	7.35	7.71	0.00	11.97	19.42	18.63	15.54	15.46	20.48	11.96	13.76
	Year	15/16	11.63	9.75	13.52	0.00	17.80	21.22	9.01	8.73	9.23	9.32	7.90	4.49	0.00	14.76	16.47	15.91	16.25	17.15	16.81	12.79	11.57
	Episodes	F/N	1,917.00	758.00	1,159.00	0.00	57.00	175.00	87.00	154.00	176.00	70.00	32.00	7.00	0.00	43.00	129.00	145.00	286.00	327.00	134.00	63.00	32.00
**London**	Quarter	1	10.74	9.41	12.07	0.00	11.64	25.13	8.63	9.82	6.17	9.84	4.63	8.81	0.00	11.63	21.61	11.46	10.01	13.27	15.92	10.49	14.25
		2	10.12	7.98	12.26	0.00	13.32	18.56	8.98	8.14	10.28	7.96	4.61	0.00	0.00	4.69	18.65	13.83	10.90	20.12	15.14	16.14	10.86
		3	13.43	13.27	13.59	0.00	19.52	24.73	6.39	10.50	11.29	15.54	14.64	16.80	0.00	8.20	17.72	14.36	13.94	18.28	19.93	16.64	13.27
		4	10.96	8.12	13.79	0.00	13.53	21.64	12.20	6.91	7.26	9.63	1.91	0.00	0.00	9.55	15.45	12.13	12.65	18.08	23.18	17.54	15.57
	Year	15/16	11.31	9.67	12.95	0.00	14.49	22.50	9.11	8.80	8.72	10.72	6.36	6.28	0.00	8.54	18.30	12.93	11.89	17.45	18.63	15.25	13.53
	Episodes	F/N	1,666.00	703.00	963.00	0.00	56.00	177.00	66.00	232.00	121.00	35.00	12.00	4.00	0.00	32.00	138.00	102.00	338.00	231.00	69.00	37.00	16.00
**Midlands And East**	Quarter	1	10.27	9.30	11.24	0.00	11.58	23.43	7.69	8.57	8.36	8.14	11.02	4.94	0.00	10.59	20.12	16.46	11.02	11.28	11.39	8.29	11.98
		2	10.75	9.89	11.60	0.00	17.18	23.09	14.27	9.63	8.41	6.90	9.58	0.00	0.00	8.27	16.88	17.46	15.98	14.80	12.42	12.35	6.28
		3	13.56	11.51	15.60	0.00	22.84	24.29	9.22	9.65	9.63	5.34	19.18	3.47	0.00	17.76	17.31	21.79	17.91	20.59	14.20	13.83	16.97
		4	9.99	7.41	12.57	0.00	13.44	14.80	7.50	8.12	9.27	8.26	5.29	0.00	0.00	5.62	14.85	14.13	20.10	20.43	13.61	6.26	18.12
	Year	15/16	11.12	9.49	12.75	0.00	16.21	21.28	9.63	8.98	8.92	7.18	11.15	2.06	0.00	10.47	17.24	17.40	16.32	16.84	12.92	10.11	13.43
	Episodes	F/N	1,739.00	701.00	1,038.00	0.00	49.00	174.00	73.00	147.00	162.00	51.00	42.00	3.00	0.00	31.00	133.00	123.00	271.00	303.00	97.00	46.00	34.00

Mean weekly incidence rate per 100,000 Persons.

Bullous Dermatoses (ICD10: L10-L14)

		All ages			Male								Female							
		Male & Female	Male	Female	<1yr	1-4yrs	5-14yrs	15-24yrs	25-44yrs	45-64yrs	65-74yrs	75-84yrs	85+yrs	<1yr	1-4yrs	5-14yrs	15-24yrs	25-44yrs	45-64yrs	65-74yrs	75-84yrs	85+yrs
**4 weekly**	1	0.18	0.14	0.22	0.00	1.27	0.00	0.00	0.00	0.00	0.00	0.00	0.00	0.00	0.00	0.00	0.00	0.00	0.00	0.34	0.00	1.64
	2	0.41	0.42	0.40	0.00	0.00	0.00	0.00	0.00	0.00	0.00	1.75	1.98	0.00	0.00	0.00	0.00	0.00	0.00	0.00	1.33	2.28
	3	0.52	0.61	0.43	0.00	0.00	0.46	0.00	0.00	0.17	0.00	0.00	4.89	0.00	0.00	0.00	0.00	0.16	0.39	1.37	0.74	1.21
	4	0.68	0.88	0.49	0.00	0.00	0.00	0.00	0.00	0.00	2.17	0.79	4.94	0.00	0.00	0.00	0.00	0.18	0.29	0.00	2.83	1.10
	5	0.31	0.26	0.35	0.00	0.00	0.00	0.00	0.00	0.25	0.00	0.00	2.09	0.00	0.00	0.00	0.00	0.19	0.32	0.98	0.49	1.19
	6	0.40	0.12	0.67	0.00	0.00	0.00	0.33	0.00	0.18	0.61	0.00	0.00	0.00	0.00	0.00	0.00	0.00	0.31	0.00	0.46	5.29
	7	0.32	0.06	0.58	0.00	0.00	0.00	0.51	0.00	0.00	0.00	0.00	0.00	0.00	0.00	0.00	0.00	0.00	0.27	0.40	2.56	2.01
	8	0.47	0.63	0.31	0.00	0.00	0.00	0.00	0.00	0.00	0.00	1.32	4.37	0.00	0.00	0.29	0.00	0.00	0.34	0.44	0.78	0.99
	9	0.39	0.68	0.09	0.00	0.00	0.00	0.00	0.00	0.00	0.00	1.79	4.36	0.00	0.00	0.00	0.00	0.11	0.19	0.00	0.52	0.00
	10	0.71	1.00	0.42	0.00	0.00	0.00	0.00	0.00	0.19	0.49	1.77	6.50	0.00	0.00	0.00	0.00	0.22	0.25	0.00	2.05	1.23
	11	0.14	0.07	0.20	0.00	0.00	0.00	0.00	0.00	0.19	0.44	0.00	0.00	0.00	0.00	0.00	0.00	0.11	0.17	0.31	0.00	1.25
	12	0.36	0.50	0.22	0.00	0.00	0.28	0.00	0.00	0.00	0.00	1.47	2.71	0.00	0.00	0.00	0.00	0.00	0.00	0.42	0.50	1.03
	13	0.19	0.35	0.03	0.00	0.00	0.00	0.00	0.16	0.00	0.39	0.92	1.70	0.00	0.00	0.00	0.00	0.27	0.00	0.00	0.00	0.00
**Quarter**	1	0.36	0.37	0.34	0.00	0.49	0.14	0.00	0.00	0.05	0.00	0.54	2.11	0.00	0.00	0.00	0.00	0.05	0.12	0.55	0.64	1.70
	2	0.51	0.41	0.61	0.00	0.00	0.00	0.26	0.00	0.13	0.86	0.24	2.16	0.00	0.00	0.00	0.00	0.11	0.36	0.42	1.61	2.95
	3	0.46	0.68	0.23	0.00	0.00	0.00	0.00	0.00	0.06	0.15	1.25	4.69	0.00	0.00	0.09	0.00	0.03	0.16	0.13	1.37	0.30
	4	0.24	0.29	0.18	0.00	0.00	0.08	0.00	0.05	0.05	0.24	0.92	1.26	0.00	0.00	0.00	0.00	0.17	0.12	0.21	0.14	1.00
**Year**	15/16	0.39	0.43	0.34	0.00	0.12	0.06	0.06	0.01	0.07	0.31	0.74	2.53	0.00	0.00	0.02	0.00	0.09	0.19	0.33	0.93	1.48
**Episodes**	F/N	106.00	43.00	63.00	0.00	1.00	2.00	2.00	1.00	5.00	7.00	12.00	13.00	0.00	0.00	1.00	0.00	8.00	13.00	8.00	17.00	16.00

Mean weekly incidence rate per 100,000 Persons.

Bullous Dermatoses (ICD10: L10 - L14)

			All ages			Male								Female							
			Male & Female	Male	Female	<1yr	1-4yrs	5-14yrs	15-24yrs	25-44yrs	45-64yrs	65-74yrs	75-84yrs	85+yrs	<1yr	1-4yrs	5-14yrs	15-24yrs	25-44yrs	45-64yrs	65-74yrs	75-84yrs	85+yrs
**North**	Quarter	1	0.20	0.34	0.05	0.00	1.95	0.00	0.00	0.00	0.00	0.00	1.13	0.00	0.00	0.00	0.00	0.00	0.00	0.48	0.00	0.00	0.00
		2	0.32	0.37	0.27	0.00	0.00	0.00	0.00	0.00	0.00	0.75	0.00	2.57	0.00	0.00	0.00	0.00	0.00	0.15	0.00	1.17	1.10
		3	0.80	1.28	0.32	0.00	0.00	0.00	0.00	0.00	0.00	0.00	1.46	10.05	0.00	0.00	0.35	0.00	0.00	0.14	0.00	1.20	1.22
		4	0.30	0.33	0.27	0.00	0.00	0.32	0.00	0.00	0.00	0.00	0.70	1.94	0.00	0.00	0.00	0.00	0.00	0.29	0.35	0.57	1.18
	Year	15/16	0.40	0.57	0.23	0.00	0.48	0.08	0.00	0.00	0.00	0.18	0.82	3.61	0.00	0.00	0.09	0.00	0.00	0.26	0.09	0.73	0.88
	Episodes	F/N	30.00	14.00	16.00	0.00	1.00	1.00	0.00	0.00	0.00	2.00	4.00	6.00	0.00	0.00	1.00	0.00	0.00	6.00	1.00	5.00	3.00
**South**	Quarter	1	0.51	0.47	0.54	0.00	0.00	0.56	0.00	0.00	0.21	0.00	1.03	2.44	0.00	0.00	0.00	0.00	0.00	0.00	1.01	2.55	1.33
		2	0.47	0.15	0.79	0.00	0.00	0.00	0.41	0.00	0.00	0.00	0.97	0.00	0.00	0.00	0.00	0.00	0.23	0.00	0.49	0.82	5.61
		3	0.32	0.55	0.09	0.00	0.00	0.00	0.00	0.00	0.00	0.00	2.38	2.54	0.00	0.00	0.00	0.00	0.00	0.00	0.00	0.81	0.00
		4	0.32	0.23	0.41	0.00	0.00	0.00	0.00	0.19	0.00	0.95	0.95	0.00	0.00	0.00	0.00	0.00	0.19	0.20	0.48	0.00	2.83
	Year	15/16	0.40	0.35	0.46	0.00	0.00	0.14	0.10	0.05	0.05	0.25	1.33	1.22	0.00	0.00	0.00	0.00	0.10	0.05	0.49	1.03	2.45
	Episodes	F/N	32.00	13.00	19.00	0.00	0.00	1.00	1.00	1.00	1.00	2.00	5.00	2.00	0.00	0.00	0.00	0.00	2.00	1.00	4.00	5.00	7.00
**London**	Quarter	1	0.41	0.67	0.15	0.00	0.00	0.00	0.00	0.00	0.00	0.00	0.00	6.01	0.00	0.00	0.00	0.00	0.20	0.00	1.19	0.00	0.00
		2	0.97	1.08	0.85	0.00	0.00	0.00	0.62	0.00	0.31	2.67	0.00	6.08	0.00	0.00	0.00	0.00	0.00	1.07	1.20	1.78	3.63
		3	0.11	0.00	0.22	0.00	0.00	0.00	0.00	0.00	0.00	0.00	0.00	0.00	0.00	0.00	0.00	0.00	0.13	0.27	0.00	1.56	0.00
		4	0.01	0.00	0.03	0.00	0.00	0.00	0.00	0.00	0.00	0.00	0.00	0.00	0.00	0.00	0.00	0.00	0.24	0.00	0.00	0.00	0.00
	Year	15/16	0.37	0.43	0.31	0.00	0.00	0.00	0.15	0.00	0.08	0.66	0.00	2.97	0.00	0.00	0.00	0.00	0.14	0.33	0.59	0.82	0.89
	Episodes	F/N	19.00	6.00	13.00	0.00	0.00	0.00	1.00	0.00	1.00	2.00	0.00	2.00	0.00	0.00	0.00	0.00	4.00	4.00	2.00	2.00	1.00
**Midlands And East**	Quarter	1	0.30	0.00	0.61	0.00	0.00	0.00	0.00	0.00	0.00	0.00	0.00	0.00	0.00	0.00	0.00	0.00	0.00	0.00	0.00	0.00	5.48
		2	0.27	0.02	0.51	0.00	0.00	0.00	0.00	0.00	0.22	0.00	0.00	0.00	0.00	0.00	0.00	0.00	0.23	0.22	0.00	2.66	1.47
		3	0.60	0.91	0.30	0.00	0.00	0.00	0.00	0.00	0.24	0.61	1.15	6.15	0.00	0.00	0.00	0.00	0.00	0.24	0.54	1.92	0.00
		4	0.31	0.59	0.03	0.00	0.00	0.00	0.00	0.00	0.22	0.00	2.03	3.09	0.00	0.00	0.00	0.00	0.25	0.00	0.00	0.00	0.00
	Year	15/16	0.37	0.39	0.36	0.00	0.00	0.00	0.00	0.00	0.17	0.15	0.82	2.33	0.00	0.00	0.00	0.00	0.12	0.11	0.13	1.12	1.70
	Episodes	F/N	25.00	10.00	15.00	0.00	0.00	0.00	0.00	0.00	3.00	1.00	3.00	3.00	0.00	0.00	0.00	0.00	2.00	2.00	1.00	5.00	5.00

Mean weekly incidence rate per 100,000 Persons.

Chickenpox (ICD10: B01)

		All ages			Male									Female								
		Male & Female	Male	Female	<1yr	1-4yrs	5-14yrs	15-24yrs	25-44yrs	45-64yrs	65-74yrs	75-84yrs	85+yrs	<1yr	1-4yrs	5-14yrs	15-24yrs	25-44yrs	45-64yrs	65-74yrs	75-84yrs	85+yrs
**4 weekly**	1	17.05	18.50	15.61	0.00	128.18	35.45	1.79	0.62	0.43	0.00	0.00	0.00	0.00	98.77	37.37	3.14	1.16	0.00	0.00	0.00	0.00
	2	13.92	12.73	15.11	0.00	83.74	27.80	1.49	0.83	0.72	0.00	0.00	0.00	0.00	106.41	27.81	0.38	1.00	0.00	0.41	0.00	0.00
	3	18.36	25.12	11.60	125.00	68.72	27.67	2.55	0.93	0.71	0.49	0.00	0.00	0.00	74.55	25.02	2.46	1.54	0.38	0.46	0.00	0.00
	4	6.76	6.95	6.57	0.00	52.43	8.45	0.49	0.53	0.64	0.00	0.00	0.00	0.00	46.89	9.19	0.84	0.79	0.45	0.97	0.00	0.00
	5	3.77	4.07	3.48	0.00	30.48	4.44	0.76	0.93	0.00	0.00	0.00	0.00	0.00	23.24	6.61	0.85	0.60	0.00	0.00	0.00	0.00
	6	9.15	10.79	7.51	28.69	52.57	13.27	1.57	0.81	0.25	0.00	0.00	0.00	11.02	45.29	9.68	0.36	1.06	0.17	0.00	0.00	0.00
	7	10.12	8.89	11.35	7.79	55.78	14.36	1.28	0.55	0.25	0.00	0.00	0.00	22.04	65.55	11.71	1.82	0.80	0.28	0.00	0.00	0.00
	8	9.78	9.25	10.30	17.29	52.01	13.30	0.33	0.11	0.21	0.00	0.00	0.00	12.80	64.67	13.99	0.00	1.27	0.00	0.00	0.00	0.00
	9	9.49	9.03	9.95	21.38	44.97	11.61	1.50	0.92	0.57	0.32	0.00	0.00	16.25	57.28	13.90	1.39	0.69	0.00	0.00	0.00	0.00
	10	12.81	14.05	11.56	36.43	73.10	13.31	1.60	0.87	0.21	0.00	0.90	0.00	35.51	52.08	13.88	1.77	0.69	0.13	0.00	0.00	0.00
	11	16.95	16.32	17.58	24.44	104.52	14.71	2.25	0.59	0.34	0.00	0.00	0.00	40.21	96.20	19.52	0.99	1.09	0.22	0.00	0.00	0.00
	12	19.25	17.92	20.58	31.41	109.32	17.79	1.47	1.03	0.21	0.00	0.00	0.00	59.06	101.95	20.80	0.55	0.80	0.48	0.43	0.00	1.19
	13	20.33	19.83	20.83	65.05	90.77	19.10	2.92	0.43	0.21	0.00	0.00	0.00	66.86	94.25	21.63	2.38	2.11	0.22	0.00	0.00	0.00
**Quarter**	1	16.49	18.76	14.22	38.46	96.21	30.70	1.93	0.78	0.61	0.15	0.00	0.00	0.00	93.67	30.63	2.08	1.23	0.12	0.27	0.00	0.00
	2	6.68	7.22	6.14	8.83	45.08	8.94	1.02	0.80	0.35	0.00	0.00	0.00	3.39	40.95	8.93	0.74	0.75	0.19	0.30	0.00	0.00
	3	10.38	10.00	10.77	21.00	54.26	12.93	0.80	0.56	0.30	0.10	0.00	0.00	21.24	60.74	12.72	1.21	0.94	0.09	0.00	0.00	0.00
	4	17.98	17.47	18.50	38.73	98.03	16.93	2.35	0.67	0.22	0.00	0.26	0.00	52.48	90.55	20.15	1.32	1.26	0.30	0.12	0.00	0.34
**Year**	15/16	12.98	13.44	12.52	26.98	73.86	17.37	1.54	0.70	0.37	0.06	0.07	0.00	19.91	71.84	18.15	1.34	1.05	0.18	0.17	0.00	0.09
**Episodes**	F/N	3469.00	1741.00	1728.00	43.00	1004.00	558.00	53.00	57.00	23.00	2.00	1.00	0.00	47.00	954.00	570.00	47.00	93.00	12.00	4.00	0.00	1.00

Mean weekly incidence rate per 100,000 Persons.

Chickenpox (ICD10: B01)

			All ages			Male									Female								
			Male & Female	Male	Female	<1yr	1-4yrs	5-14yrs	15-24yrs	25-44yrs	45-64yrs	65-74yrs	75-84yrs	85+yrs	<1yr	1-4yrs	5-14yrs	15-24yrs	25-44yrs	45-64yrs	65-74yrs	75-84yrs	85+yrs
**North**	Quarter	1	13.49	13.48	13.50	0.00	93.31	25.54	1.09	0.86	0.50	0.00	0.00	0.00	0.00	93.02	26.69	1.23	0.57	0.00	0.00	0.00	0.00
		2	5.19	5.79	4.59	0.00	43.38	6.71	1.38	0.14	0.46	0.00	0.00	0.00	0.00	31.14	9.12	0.00	1.01	0.00	0.00	0.00	0.00
		3	9.81	9.60	10.02	25.11	52.29	7.99	0.27	0.15	0.20	0.40	0.00	0.00	22.83	58.26	7.61	0.77	0.60	0.14	0.00	0.00	0.00
		4	15.25	15.06	15.44	50.00	73.03	10.68	1.40	0.27	0.15	0.00	0.00	0.00	25.47	94.55	16.37	1.58	0.67	0.29	0.00	0.00	0.00
	Year	15/16	11.01	11.06	10.97	19.37	65.64	12.69	1.04	0.35	0.32	0.10	0.00	0.00	12.33	69.72	14.97	0.91	0.71	0.11	0.00	0.00	0.00
	Episodes	F/N	807.00	389.00	418.00	14.00	238.00	110.00	13.00	7.00	6.00	1.00	0.00	0.00	9.00	249.00	128.00	12.00	17.00	3.00	0.00	0.00	0.00
**South**	Quarter	1	13.46	13.39	13.54	0.00	96.53	22.44	0.47	0.23	0.22	0.60	0.00	0.00	0.00	97.84	21.76	0.46	1.22	0.00	0.57	0.00	0.00
		2	6.31	6.65	5.96	0.00	53.67	5.31	0.42	0.45	0.00	0.00	0.00	0.00	0.00	43.62	7.64	1.76	0.23	0.42	0.00	0.00	0.00
		3	11.22	11.05	11.39	19.99	65.88	11.73	0.83	1.00	0.00	0.00	0.00	0.00	14.41	70.06	15.14	0.90	1.81	0.20	0.00	0.00	0.00
		4	15.08	16.19	13.97	24.04	101.52	17.06	2.28	0.82	0.00	0.00	0.00	0.00	30.97	77.66	13.28	0.81	1.46	0.19	0.00	0.00	1.36
	Year	15/16	11.58	11.90	11.27	11.25	79.82	14.19	1.03	0.63	0.05	0.15	0.00	0.00	11.71	72.40	14.43	0.98	1.18	0.20	0.14	0.00	0.36
	Episodes	F/N	765.00	396.00	369.00	6.00	251.00	116.00	10.00	11.00	1.00	1.00	0.00	0.00	6.00	214.00	112.00	9.00	22.00	4.00	1.00	0.00	1.00
**London**	Quarter	1	17.10	18.40	15.80	0.00	102.31	56.03	4.73	1.44	1.09	0.00	0.00	0.00	0.00	86.50	49.73	3.15	2.32	0.47	0.00	0.00	0.00
		2	7.92	8.23	7.61	14.85	40.66	15.65	1.25	0.67	0.95	0.00	0.00	0.00	13.57	42.42	9.00	1.19	0.81	0.34	1.20	0.00	0.00
		3	10.32	9.12	11.52	13.67	45.44	20.63	0.99	0.55	0.78	0.00	0.00	0.00	25.64	55.52	19.57	1.86	1.10	0.00	0.00	0.00	0.00
		4	19.89	18.79	20.99	43.83	93.85	26.22	3.62	0.87	0.73	0.00	0.00	0.00	71.21	84.50	30.04	1.19	1.43	0.51	0.00	0.00	0.00
	Year	15/16	13.92	13.73	14.11	18.57	71.00	29.57	2.66	0.88	0.88	0.00	0.00	0.00	28.43	67.56	27.14	1.84	1.42	0.33	0.29	0.00	0.00
	Episodes	F/N	1,100.00	563.00	537.00	14.00	270.00	225.00	19.00	23.00	12.00	0.00	0.00	0.00	20.00	255.00	204.00	14.00	39.00	4.00	1.00	0.00	0.00
**Midlands And East**	Quarter	1	21.91	29.78	14.05	153.85	92.69	18.80	1.43	0.61	0.62	0.00	0.00	0.00	0.00	97.31	24.33	3.48	0.79	0.00	0.51	0.00	0.00
		2	7.32	8.24	6.40	20.46	42.61	8.09	1.04	1.93	0.00	0.00	0.00	0.00	0.00	46.63	9.97	0.00	0.96	0.00	0.00	0.00	0.00
		3	10.18	10.22	10.15	25.22	53.43	11.38	1.12	0.56	0.24	0.00	0.00	0.00	22.10	59.11	8.55	1.30	0.24	0.00	0.00	0.00	0.00
		4	21.72	19.82	23.62	37.05	123.71	13.77	2.11	0.73	0.00	0.00	1.03	0.00	82.27	105.50	20.93	1.69	1.46	0.22	0.49	0.00	0.00
	Year	15/16	15.40	17.07	13.74	58.73	78.97	13.02	1.44	0.96	0.21	0.00	0.27	0.00	27.15	77.67	16.04	1.62	0.88	0.06	0.25	0.00	0.00
	Episodes	F/N	797.00	393.00	404.00	9.00	245.00	107.00	11.00	16.00	4.00	0.00	1.00	0.00	12.00	236.00	126.00	12.00	15.00	1.00	2.00	0.00	0.00

Mean weekly incidence rate per 100,000 Persons.

Common Cold (ICD10: J00,J06))

		All ages			Male									Female								
		Male & Female	Male	Female	<1yr	1-4yrs	5-14yrs	15-24yrs	25-44yrs	45-64yrs	65-74yrs	75-84yrs	85+yrs	<1yr	1-4yrs	5-14yrs	15-24yrs	25-44yrs	45-64yrs	65-74yrs	75-84yrs	85+yrs
**4 weekly**	1	108.45	106.36	110.54	0.00	635.44	111.05	42.66	31.71	26.09	34.09	36.11	40.10	0.00	552.36	116.98	77.01	65.84	54.84	57.53	39.38	30.90
	2	136.08	116.69	155.47	390.63	427.90	76.78	26.02	23.55	21.68	27.11	24.71	31.88	781.25	358.19	72.33	40.44	37.83	28.56	33.26	26.90	20.46
	3	121.81	130.02	113.60	562.65	435.54	69.65	16.48	17.83	15.40	16.89	13.89	21.83	320.26	421.49	75.35	42.95	37.69	34.03	26.77	37.53	26.37
	4	94.98	106.71	83.25	479.10	322.63	36.48	20.68	17.09	16.64	25.14	24.22	18.42	243.24	287.35	34.76	42.66	37.07	29.80	33.20	24.51	16.65
	5	169.21	174.50	163.93	800.21	509.35	77.99	46.43	23.58	25.22	29.72	21.28	36.71	614.67	502.14	92.96	67.46	52.67	39.58	37.59	28.33	39.95
	6	267.42	268.35	266.50	1027.93	937.35	134.46	45.92	36.88	35.44	56.44	56.90	83.81	821.93	908.73	154.01	109.24	90.16	76.78	59.94	74.86	102.84
	7	294.05	283.54	304.56	1130.14	1025.36	122.02	36.15	38.29	34.49	46.61	54.75	64.09	1114.92	1054.52	141.24	81.66	74.29	60.42	74.43	63.74	75.84
	8	421.68	427.65	415.72	2078.71	1210.65	232.06	48.32	35.61	41.92	53.59	61.82	86.16	1717.91	1253.56	233.43	101.69	93.25	86.72	85.60	80.82	88.52
	9	279.62	270.05	289.19	1190.96	706.89	135.22	55.05	51.85	57.68	76.25	87.80	68.81	1153.05	660.47	148.06	91.61	109.34	104.53	91.47	103.47	140.74
	10	366.68	368.77	364.60	1565.61	1075.08	276.42	70.91	53.42	67.33	74.30	67.94	67.92	1325.90	1015.77	293.87	122.52	129.38	109.94	96.17	96.20	91.63
	11	337.53	335.91	339.14	1363.49	979.34	241.45	74.36	59.25	65.93	68.96	65.11	105.31	1248.51	904.39	244.56	127.35	143.48	110.03	89.87	96.04	88.06
	12	294.43	298.39	290.46	1310.40	772.84	249.95	70.78	55.57	48.22	66.60	68.59	42.58	1095.45	724.87	260.20	109.05	108.39	83.84	80.62	75.75	76.00
	13	223.36	226.51	220.21	1148.21	537.36	114.92	36.57	28.44	32.71	39.97	55.47	44.96	997.79	512.27	124.18	66.32	63.62	56.02	48.64	62.40	50.67
**Quarter**	1	121.06	116.82	125.31	293.31	510.07	87.76	29.48	24.92	21.44	26.65	25.77	31.94	338.93	452.35	90.43	55.28	48.56	40.35	40.60	34.97	26.30
	2	183.92	188.15	179.69	775.01	618.88	86.66	37.61	26.62	26.33	38.23	37.07	46.92	598.74	597.09	96.03	73.22	61.03	49.39	44.39	43.24	54.05
	3	341.85	339.73	343.97	1536.30	992.43	182.80	50.62	43.24	48.78	59.68	66.93	76.82	1342.65	993.99	196.75	97.88	98.70	89.51	87.73	86.16	102.38
	4	297.21	298.51	295.90	1309.43	811.45	212.92	62.40	49.40	51.85	62.67	64.53	61.89	1149.67	758.01	221.63	103.85	109.30	87.50	76.60	81.59	75.00
**Year**	15/16	237.16	236.98	237.34	984.76	734.69	143.86	45.36	36.30	37.38	47.11	48.88	54.53	863.01	701.45	152.54	82.96	79.96	67.08	62.60	61.87	64.63
**Episodes**	F/N	61996.00	27146.00	34850.00	1760.00	10477.00	4967.00	1699.00	3165.00	2823.00	1267.00	704.00	284.00	1495.00	9696.00	5068.00	3125.00	6985.00	4969.00	1800.00	1087.00	625.00

Mean weekly incidence rate per 100,000 Persons.

Common Cold (ICD10: J00; J06)

			All ages			Male								Female							
			Male & Female	Male	Female	<1yr	1-4yrs	5-14yrs	15-24yrs	25-44yrs	45-64yrs	65-74yrs	75-84yrs	85+yrs	<1yr	1-4yrs	5-14yrs	15-24yrs	25-44yrs	45-64yrs	65-74yrs	75-84yrs	85+yrs
**North**	Quarter	1	119.74	141.19	98.30	389.91	625.28	87.32	32.97	26.93	22.80	28.45	30.03	26.99	0.00	501.46	107.26	59.13	62.46	44.17	41.24	44.18	24.81
		2	171.99	168.30	175.68	654.93	574.50	79.72	32.71	27.90	27.18	38.05	33.64	46.09	514.52	624.49	104.47	72.44	64.68	50.69	46.51	47.53	55.80
		3	342.81	355.32	330.30	1,670.97	999.41	171.76	46.99	47.29	47.24	65.54	74.06	74.63	1,237.38	982.16	192.84	88.81	106.45	91.43	84.91	95.59	93.12
		4	314.76	315.73	313.79	1,506.20	803.15	187.37	52.84	49.95	50.81	65.56	63.50	62.22	1,332.19	765.27	200.88	86.25	100.94	82.68	84.82	79.44	91.65
	Year	15/16	238.79	246.47	231.11	1,064.01	751.57	132.59	41.59	38.24	37.27	49.70	50.56	52.67	781.61	719.23	152.29	76.84	83.96	67.53	64.75	66.93	66.82
	Episodes	F/N	18,897.00	8,191.00	10,706.00	584.00	2,968.00	1,349.00	576.00	953.00	927.00	486.00	258.00	90.00	465.00	2,742.00	1,482.00	1,082.00	1,977.00	1,666.00	661.00	417.00	214.00
**South**	Quarter	1	84.29	78.08	90.51	76.16	443.56	56.49	20.42	25.45	18.99	17.33	23.36	20.96	160.26	381.97	63.74	45.13	40.35	32.59	32.73	27.24	30.56
		2	191.07	199.41	182.73	969.92	564.94	67.15	33.64	28.05	24.49	28.09	32.54	45.92	722.42	555.36	74.96	65.82	59.19	43.75	45.38	33.95	43.73
		3	349.61	341.03	358.18	1,632.13	979.12	147.12	50.08	41.13	40.73	48.54	53.87	76.58	1,533.74	959.49	165.65	115.56	96.98	82.02	92.09	78.37	99.72
		4	293.97	303.57	284.38	1,378.82	815.91	189.61	59.75	52.67	54.73	60.72	66.76	53.15	1,058.21	754.25	202.32	104.74	114.63	87.74	77.43	74.77	85.30
	Year	15/16	230.95	231.90	229.99	1,021.14	703.05	116.50	41.33	37.12	35.11	39.08	44.56	49.23	872.23	664.49	128.10	83.22	78.48	62.02	62.20	53.98	65.21
	Episodes	F/N	13,437.00	5,875.00	7,562.00	381.00	2,234.00	972.00	396.00	670.00	673.00	295.00	178.00	76.00	311.00	1,986.00	1,009.00	743.00	1,393.00	1,185.00	491.00	264.00	180.00
**London**	Quarter	1	138.16	158.54	117.77	480.77	599.66	139.46	40.31	29.03	25.50	34.49	28.16	49.44	76.92	555.62	128.04	78.42	52.38	55.93	58.37	40.41	13.88
		2	222.78	222.62	222.93	804.08	790.02	140.43	52.73	28.62	33.15	52.45	53.31	48.78	710.50	769.10	138.49	84.95	70.83	60.63	49.21	60.68	61.99
		3	377.76	368.36	387.15	1,560.17	1,117.92	277.41	59.68	43.26	61.08	65.89	72.41	57.44	1,397.76	1,186.15	280.65	96.18	98.06	110.38	99.48	106.55	109.17
		4	331.12	327.51	334.73	1,279.25	974.43	294.95	70.27	51.31	57.86	77.00	72.78	69.75	1,213.02	898.78	314.21	117.20	110.30	103.15	85.35	99.27	71.33
	Year	15/16	268.65	270.36	266.95	1,035.75	872.47	214.61	56.02	38.31	44.65	57.83	56.97	56.60	856.41	853.29	217.21	94.62	83.41	82.91	73.33	77.15	64.23
	Episodes	F/N	18,557.00	8,177.00	10,380.00	541.00	3,429.00	1,772.00	414.00	1,041.00	642.00	192.00	107.00	39.00	489.00	3,304.00	1,743.00	770.00	2,414.00	1,120.00	278.00	186.00	76.00
**Midlands And East**	Quarter	1	142.05	89.47	194.64	226.42	371.79	67.78	24.24	18.28	18.49	26.33	21.51	30.39	1,118.52	370.35	62.68	38.43	39.05	28.71	30.04	28.04	35.92
		2	149.83	162.25	137.41	671.11	546.07	59.32	31.37	21.89	20.51	34.32	28.81	46.87	447.52	439.43	66.22	69.69	49.40	42.48	36.48	30.81	54.67
		3	297.24	294.22	300.26	1,281.92	873.28	134.89	45.72	41.29	46.05	58.77	67.39	98.63	1,201.72	848.18	147.85	90.97	93.30	74.22	74.45	64.14	107.52
		4	248.97	247.21	250.72	1,073.46	652.31	179.76	66.76	43.68	44.01	47.42	55.09	62.44	995.26	613.74	169.12	107.22	111.32	76.41	58.81	72.87	51.72
	Year	15/16	210.27	199.21	221.32	818.13	611.64	111.75	42.49	31.52	32.49	41.81	43.42	59.64	941.79	568.79	112.56	77.15	73.99	55.85	50.11	49.42	62.25
	Episodes	F/N	11,105.00	4,903.00	6,202.00	254.00	1,846.00	874.00	313.00	501.00	581.00	294.00	161.00	79.00	230.00	1,664.00	834.00	530.00	1,201.00	998.00	370.00	220.00	155.00

Mean weekly incidence rate per 100,000 Persons.

Disorders of Conjunctiva (ICD10: H10-H13))

		All ages			Male									Female								
		Male & Female	Male	Female	<1yr	1-4yrs	5-14yrs	15-24yrs	25-44yrs	45-64yrs	65-74yrs	75-84yrs	85+yrs	<1yr	1-4yrs	5-14yrs	15-24yrs	25-44yrs	45-64yrs	65-74yrs	75-84yrs	85+yrs
**4 weekly**	1	48.88	45.53	52.22	0.00	207.06	33.54	8.26	12.46	16.06	23.95	38.78	69.65	0.00	212.81	37.44	13.07	25.98	23.79	36.21	51.00	69.71
	2	62.27	83.86	40.68	403.65	176.92	38.02	11.33	12.99	13.20	18.21	33.17	47.26	0.00	136.98	33.53	17.71	23.56	19.62	23.64	37.47	73.60
	3	119.61	129.42	109.81	802.79	125.20	30.58	10.14	14.86	16.22	27.91	45.34	91.70	630.35	117.97	22.88	15.08	20.01	26.17	34.60	43.96	77.30
	4	84.75	94.24	75.27	605.97	83.34	22.91	8.91	10.40	15.68	19.05	30.04	51.88	399.70	61.26	13.64	11.65	15.93	25.77	37.15	33.32	79.00
	5	81.64	88.25	75.03	530.21	83.14	17.75	7.21	8.86	15.42	24.76	30.40	76.53	413.63	66.28	15.55	13.41	13.51	20.87	32.06	43.25	56.76
	6	74.74	74.59	74.89	354.37	120.61	24.21	10.38	10.02	11.28	28.68	36.27	75.45	358.15	105.73	22.66	17.62	18.05	19.34	35.93	35.80	60.70
	7	83.32	79.75	86.90	373.27	148.53	23.00	9.51	9.20	13.80	29.31	43.56	67.53	455.26	120.21	20.67	13.21	16.94	20.19	31.07	31.14	73.44
	8	89.63	93.14	86.12	384.50	230.61	27.99	10.93	10.73	13.62	23.75	42.99	93.17	354.26	193.99	23.47	15.00	17.16	24.29	31.68	41.58	73.64
	9	95.92	91.18	100.65	419.79	182.32	19.38	12.93	9.80	14.20	28.87	48.37	84.98	479.82	198.49	23.13	8.28	16.65	29.82	33.69	53.04	62.97
	10	129.41	124.08	134.74	545.10	372.33	27.65	14.40	14.39	14.82	30.06	42.98	54.95	610.98	346.32	27.61	18.34	23.56	23.73	28.94	51.42	81.74
	11	129.91	132.40	127.42	618.24	354.19	31.70	11.61	13.16	15.02	32.32	47.41	67.97	600.37	274.25	35.80	18.14	26.40	27.21	32.06	42.59	89.94
	12	110.99	105.22	116.76	510.30	254.28	22.83	10.83	9.70	15.13	22.77	39.01	62.13	548.85	253.15	23.77	18.15	25.98	25.57	35.84	43.34	76.16
	13	94.68	87.01	102.36	425.57	172.97	26.76	8.99	13.48	17.57	29.34	34.10	54.29	493.68	159.99	26.08	21.90	22.79	25.85	41.69	40.54	88.72
**Quarter**	1	74.76	83.13	66.39	371.21	172.60	34.00	9.79	13.36	15.23	23.40	39.07	69.55	193.95	160.30	31.76	15.12	23.40	23.24	31.85	44.67	73.24
	2	80.91	85.15	76.66	489.54	99.77	21.02	8.39	9.65	14.04	24.38	32.84	66.71	406.16	77.39	17.65	14.00	16.04	21.75	34.53	36.65	65.81
	3	95.18	94.37	95.98	422.07	214.52	24.90	12.01	10.52	14.08	27.60	44.63	79.06	434.29	198.22	23.40	13.71	17.67	24.53	31.54	45.14	75.29
	4	115.07	110.40	119.73	515.60	280.25	27.13	11.26	12.62	15.76	28.57	40.95	61.46	566.64	252.56	28.27	18.75	24.97	26.36	35.83	42.92	81.28
**Year**	15/16	91.92	93.59	90.26	450.85	193.45	26.77	10.38	11.56	14.79	26.04	39.40	69.05	403.40	173.63	25.33	15.46	20.60	24.01	33.48	42.36	74.05
**Episodes**	F/N	18577.00	8318.00	10259.00	631.00	2712.00	893.00	381.00	970.00	1090.00	713.00	569.00	359.00	640.00	2352.00	808.00	589.00	1740.00	1740.00	951.00	733.00	706.00

Mean weekly incidence rate per 100,000 Persons.

Disorders of Conjunctiva (ICD10: H10-H13)

			All ages			Male									Female								
			Male & Female	Male	Female	<1yr	1-4yrs	5-14yrs	15-24yrs	25-44yrs	45-64yrs	65-74yrs	75-84yrs	85+yrs	<1yr	1-4yrs	5-14yrs	15-24yrs	25-44yrs	45-64yrs	65-74yrs	75-84yrs	85+yrs
**North**	Quarter	1	73.69	87.86	59.53	431.10	171.52	26.48	11.15	13.93	15.58	23.68	41.34	56.00	113.12	184.00	30.09	16.69	26.07	22.10	30.05	44.35	69.25
		2	81.44	93.26	69.63	567.31	93.57	15.88	9.22	10.53	15.80	24.73	32.65	69.65	341.15	86.33	15.72	19.58	16.17	21.87	31.89	28.05	65.90
		3	102.72	103.06	102.39	491.48	227.14	19.31	9.96	9.43	11.76	26.34	44.34	87.77	494.53	207.00	20.60	17.83	18.39	22.09	30.67	35.71	74.68
		4	124.15	118.38	129.92	597.16	287.04	27.84	9.37	12.23	13.69	26.63	36.64	54.83	617.78	306.53	26.49	18.89	24.42	22.06	28.59	49.28	75.27
	Year	15/16	96.04	100.98	91.11	523.18	196.56	22.48	9.92	11.54	14.20	25.37	38.70	66.83	395.91	198.05	23.29	18.26	21.32	22.03	30.27	39.53	71.35
	Episodes	F/N	5,595.00	2,463.00	3,132.00	225.00	771.00	214.00	127.00	263.00	326.00	238.00	190.00	109.00	211.00	732.00	209.00	246.00	467.00	515.00	298.00	235.00	219.00
**South**	Quarter	1	77.58	57.34	97.82	178.89	174.52	22.11	8.20	14.50	13.77	25.41	21.95	56.73	517.08	140.60	27.78	15.08	23.31	20.75	26.59	38.91	70.27
		2	91.73	81.93	101.53	470.30	105.28	20.52	7.19	9.24	11.96	21.98	32.40	58.55	648.85	68.75	14.36	7.30	17.41	16.43	34.04	46.60	60.07
		3	101.15	103.66	98.65	524.60	220.43	22.73	14.65	9.56	15.19	26.24	42.78	56.72	480.74	193.95	22.04	14.22	16.89	23.44	32.34	38.00	66.24
		4	121.59	108.20	134.97	519.66	277.88	22.50	9.81	14.33	14.97	32.31	31.36	51.01	698.09	264.70	27.66	15.66	23.11	27.93	43.04	30.42	84.16
	Year	15/16	98.46	88.17	108.75	425.18	196.10	21.97	9.96	11.95	13.99	26.60	32.11	55.66	588.30	168.84	23.05	13.12	20.24	22.25	34.18	38.33	70.45
	Episodes	F/N	4,367.00	1,947.00	2,420.00	140.00	624.00	183.00	96.00	218.00	268.00	202.00	130.00	86.00	169.00	506.00	179.00	118.00	361.00	427.00	273.00	187.00	200.00
**London**	Quarter	1	68.37	76.55	60.19	240.38	182.16	55.41	12.00	13.79	17.08	17.03	48.76	102.30	76.92	168.74	41.10	16.80	21.78	28.04	41.88	50.19	96.26
		2	76.36	82.25	70.48	500.33	77.06	28.07	10.29	10.63	13.92	25.01	26.07	48.85	347.37	70.54	22.96	13.96	15.48	26.09	38.00	40.13	59.76
		3	86.98	84.00	89.96	369.49	198.89	33.85	11.42	11.42	12.96	29.21	48.39	40.41	371.12	181.03	28.00	9.74	16.23	27.53	32.22	65.98	77.83
		4	90.11	89.18	91.05	343.66	236.84	37.44	13.99	11.39	17.66	29.94	36.41	75.25	375.30	208.45	29.18	16.97	25.15	28.51	34.31	39.46	62.16
	Year	15/16	80.64	83.11	78.17	363.09	174.93	38.67	11.96	11.80	15.45	25.38	39.84	66.86	294.24	158.16	30.29	14.42	19.76	27.56	36.56	48.76	73.78
	Episodes	F/N	4,303.00	1,950.00	2,353.00	150.00	691.00	299.00	86.00	310.00	213.00	83.00	73.00	45.00	148.00	614.00	229.00	113.00	556.00	358.00	134.00	116.00	85.00
**Midlands And East**	Quarter	1	79.41	110.78	48.03	634.47	162.20	32.02	7.80	11.21	14.48	27.49	44.23	63.15	68.68	147.85	28.06	11.89	22.42	22.07	28.86	45.24	57.19
		2	74.08	83.15	65.02	420.21	123.16	19.60	6.86	8.18	14.48	25.80	40.25	89.81	287.27	83.95	17.56	15.17	15.10	22.61	34.19	31.80	77.50
		3	89.84	86.78	92.90	302.71	211.61	23.70	11.98	11.68	16.41	28.62	43.01	131.32	390.74	210.88	22.95	13.06	19.16	25.04	30.92	40.89	82.42
		4	124.41	125.84	122.97	601.92	319.25	20.74	11.88	12.55	16.71	25.39	59.40	64.76	575.41	230.55	29.77	23.48	27.20	26.92	37.37	52.52	103.54
	Year	15/16	92.55	102.10	83.00	491.94	206.23	23.95	9.67	10.94	15.54	26.80	46.96	86.84	335.15	169.48	24.68	16.04	21.09	24.21	32.92	42.80	80.60
	Episodes	F/N	4,312.00	1,958.00	2,354.00	116.00	626.00	197.00	72.00	179.00	283.00	190.00	176.00	119.00	112.00	500.00	191.00	112.00	356.00	440.00	246.00	195.00	202.00

Mean weekly incidence rate per 100,000 Persons.

Disorders of The Peripheral Nervous System (ICD10: G50-G64,G70-G72)

		All ages			Male									Female								
		Male & Female	Male	Female	<1yr	1-4yrs	5-14yrs	15-24yrs	25-44yrs	45-64yrs	65-74yrs	75-84yrs	85+yrs	<1yr	1-4yrs	5-14yrs	15-24yrs	25-44yrs	45-64yrs	65-74yrs	75-84yrs	85+yrs
**4 weekly**	1	9.86	8.66	11.07	0.00	0.00	0.00	0.34	5.71	12.47	16.21	24.74	18.44	0.00	0.00	0.37	2.53	10.87	17.89	19.34	20.18	28.42
	2	9.07	9.28	8.86	0.00	0.00	0.00	0.81	5.83	9.74	14.91	19.88	32.32	0.00	0.00	0.95	0.41	9.66	14.47	17.86	25.29	11.12
	3	8.75	7.33	10.17	0.00	0.00	0.50	2.55	5.62	11.60	13.66	27.82	4.27	0.00	0.00	0.49	2.73	8.99	20.43	16.98	23.44	18.44
	4	9.17	8.08	10.26	0.00	0.00	0.41	2.68	5.12	11.30	13.51	22.91	16.76	0.00	0.00	1.27	3.94	9.21	16.10	18.16	26.55	17.16
	5	9.82	8.16	11.48	0.00	0.00	0.00	1.03	4.44	12.89	20.13	21.35	13.61	0.00	0.00	0.39	4.09	11.53	19.18	21.95	20.90	25.26
	6	9.46	7.43	11.49	0.00	0.80	0.00	1.04	6.70	13.84	18.47	21.95	4.05	0.00	0.00	0.41	2.81	13.05	18.41	20.72	26.78	21.19
	7	9.53	8.96	10.11	0.00	0.00	0.46	2.54	7.02	10.96	20.84	24.75	14.03	0.00	0.00	0.41	4.00	11.61	16.58	19.99	24.82	13.60
	8	10.23	9.35	11.10	0.00	0.00	0.29	0.76	5.30	12.38	22.62	24.67	18.14	0.00	0.00	1.27	4.88	11.31	18.77	16.43	31.84	15.45
	9	7.82	7.82	7.81	0.00	0.00	0.00	1.67	5.49	10.88	18.98	19.97	13.38	0.00	0.00	0.47	2.80	8.26	14.11	13.40	16.39	14.89
	10	9.33	8.20	10.47	0.00	0.00	0.00	2.58	7.03	12.70	20.15	19.61	11.72	0.00	0.00	0.00	1.90	13.07	21.95	16.39	20.56	20.36
	11	9.95	8.44	11.47	0.00	1.89	0.40	0.64	3.55	13.48	17.91	23.45	14.66	0.00	0.00	0.00	3.26	13.61	20.47	22.68	23.78	19.38
	12	9.17	6.74	11.60	0.00	0.00	0.00	1.28	3.60	14.16	13.71	13.57	14.36	0.00	0.00	0.00	3.57	9.89	18.89	21.46	22.89	27.69
	13	9.62	6.70	12.55	0.00	0.00	0.00	1.68	6.32	13.44	20.05	17.06	1.72	0.00	0.00	0.00	2.61	12.66	20.56	18.51	32.69	25.90
**Quarter**	1	9.28	8.44	10.11	0.00	0.00	0.15	1.16	5.72	11.36	15.03	24.19	18.35	0.00	0.00	0.58	1.94	9.92	17.62	18.16	22.75	20.03
	2	9.59	8.09	11.09	0.00	0.25	0.27	1.94	5.38	12.85	17.94	21.76	12.47	0.00	0.00	0.76	3.78	11.34	17.97	20.22	24.33	21.40
	3	9.16	8.48	9.85	0.00	0.00	0.09	1.30	5.98	11.34	19.69	22.98	14.91	0.00	0.00	0.54	3.55	10.59	17.52	16.62	25.03	14.78
	4	9.47	7.43	11.51	0.00	0.54	0.11	1.54	5.04	13.54	18.31	18.25	9.56	0.00	0.00	0.00	2.87	12.27	19.92	19.99	24.79	23.80
**Year**	15/16	9.38	8.10	10.66	0.00	0.20	0.16	1.49	5.52	12.30	17.75	21.73	13.74	0.00	0.00	0.46	3.03	11.05	18.29	18.77	24.24	20.07
**Episodes**	F/N	5876.00	2304.00	3572.00	0.00	3.00	5.00	54.00	452.00	915.00	477.00	325.00	73.00	0.00	0.00	14.00	108.00	939.00	1357.00	529.00	430.00	195.00

Mean weekly incidence rate per 100,000 Persons.

Disorders of The Peripheral Nervous System (ICD10: G50-G64,G70-G72)

			All ages			Male								Female							
			Male & Female	Male	Female	<1yr	1-4yrs	5-14yrs	15-24yrs	25-44yrs	45-64yrs	65-74yrs	75-84yrs	85+yrs	<1yr	1-4yrs	5-14yrs	15-24yrs	25-44yrs	45-64yrs	65-74yrs	75-84yrs	85+yrs
**North**	Quarter	1	8.06	7.65	8.48	0.00	0.00	0.62	1.07	6.07	10.97	13.87	22.92	13.33	0.00	0.00	0.66	1.77	8.19	16.86	12.84	23.52	12.46
		2	7.39	6.34	8.44	0.00	0.98	0.00	0.78	5.08	10.40	17.44	17.40	4.98	0.00	0.00	0.48	3.60	8.63	15.44	13.07	21.21	13.55
		3	8.33	8.14	8.51	0.00	0.00	0.35	0.83	6.36	9.43	13.12	19.74	23.44	0.00	0.00	0.00	1.20	10.27	17.99	18.83	20.33	8.01
		4	8.98	7.86	10.09	0.00	0.00	0.00	1.87	5.25	12.78	19.03	18.83	13.01	0.00	0.00	0.00	2.69	12.65	18.28	14.79	22.04	20.39
	Year	15/16	8.20	7.50	8.90	0.00	0.24	0.24	1.15	5.68	10.93	15.92	19.70	13.68	0.00	0.00	0.28	2.32	9.98	17.16	14.88	21.78	13.73
	Episodes	F/N	1,677.00	677.00	1,000.00	0.00	1.00	2.00	15.00	133.00	261.00	148.00	95.00	22.00	0.00	0.00	2.00	31.00	232.00	411.00	150.00	132.00	42.00
**South**	Quarter	1	10.76	9.98	11.54	0.00	0.00	0.00	0.94	7.29	13.75	18.93	24.76	24.10	0.00	0.00	1.68	0.95	10.76	20.44	16.01	28.38	25.63
		2	10.93	10.25	11.61	0.00	0.00	0.00	3.32	7.60	13.66	18.17	31.68	17.82	0.00	0.00	2.03	2.25	13.93	18.79	19.29	26.02	22.15
		3	11.05	10.26	11.85	0.00	0.00	0.00	1.67	7.37	11.84	20.18	29.37	21.87	0.00	0.00	0.50	4.51	12.03	18.35	19.17	28.06	24.01
		4	10.44	8.94	11.95	0.00	0.00	0.46	1.14	5.96	14.55	22.76	22.80	12.75	0.00	0.00	0.00	4.09	14.37	23.13	21.71	25.43	18.84
	Year	15/16	10.79	9.84	11.74	0.00	0.00	0.12	1.75	7.04	13.47	20.06	27.07	19.01	0.00	0.00	1.03	2.97	12.80	20.23	19.10	26.94	22.59
	Episodes	F/N	1,696.00	696.00	1,000.00	0.00	0.00	1.00	17.00	128.00	259.00	152.00	110.00	29.00	0.00	0.00	8.00	26.00	230.00	387.00	152.00	133.00	64.00
**London**	Quarter	1	9.29	8.25	10.33	0.00	0.00	0.00	0.67	4.03	10.72	11.76	23.71	23.32	0.00	0.00	0.00	2.53	9.93	18.72	25.12	19.54	17.10
		2	10.21	8.73	11.69	0.00	0.00	0.57	2.56	3.78	15.46	21.29	16.61	18.35	0.00	0.00	0.00	5.84	12.03	18.33	24.63	25.42	18.96
		3	8.06	7.22	8.90	0.00	0.00	0.00	1.51	4.22	11.82	23.54	12.89	11.02	0.00	0.00	0.48	4.83	10.24	16.73	10.67	30.65	6.46
		4	8.70	5.15	12.24	0.00	0.86	0.00	0.92	3.13	11.88	18.17	11.43	0.00	0.00	0.00	0.00	2.40	9.67	18.05	24.12	27.77	28.15
	Year	15/16	9.06	7.30	10.82	0.00	0.23	0.14	1.41	3.78	12.46	18.68	16.07	12.92	0.00	0.00	0.12	3.87	10.46	17.96	21.19	25.88	17.87
	Episodes	F/N	1,101.00	382.00	719.00	0.00	1.00	1.00	10.00	99.00	171.00	62.00	29.00	9.00	0.00	0.00	1.00	30.00	292.00	235.00	77.00	63.00	21.00
**Midlands And East**	Quarter	1	9.00	7.89	10.10	0.00	0.00	0.00	1.97	5.47	10.00	15.55	25.38	12.64	0.00	0.00	0.00	2.51	10.80	14.47	18.66	19.57	24.92
		2	9.83	7.05	12.62	0.00	0.00	0.50	1.09	5.07	11.88	14.85	21.36	8.72	0.00	0.00	0.53	3.43	10.77	19.31	23.88	24.68	30.94
		3	9.21	8.29	10.13	0.00	0.00	0.00	1.19	5.97	12.29	21.93	29.94	3.33	0.00	0.00	1.16	3.64	9.81	17.00	17.82	21.08	20.65
		4	9.77	7.77	11.77	0.00	1.30	0.00	2.22	5.82	14.95	13.26	19.94	12.47	0.00	0.00	0.00	2.30	12.37	20.22	19.33	23.93	27.81
	Year	15/16	9.46	7.75	11.17	0.00	0.34	0.12	1.63	5.59	12.33	16.34	24.08	9.35	0.00	0.00	0.41	2.96	10.97	17.79	19.91	22.35	26.11
	Episodes	F/N	1,402.00	549.00	853.00	0.00	1.00	1.00	12.00	92.00	224.00	115.00	91.00	13.00	0.00	0.00	3.00	21.00	185.00	324.00	150.00	102.00	68.00

Mean weekly incidence rate per 100,000 Persons.

Hayfever/Allergic Rhinitis (ICD10: J30))

		All ages			Male									Female								
		Male & Female	Male	Female	<1yr	1-4yrs	5-14yrs	15-24yrs	25-44yrs	45-64yrs	65-74yrs	75-84yrs	85+yrs	<1yr	1-4yrs	5-14yrs	15-24yrs	25-44yrs	45-64yrs	65-74yrs	75-84yrs	85+yrs
**4 weekly**	1	21.31	20.06	22.56	0.00	18.89	62.42	33.03	17.86	11.43	15.37	14.32	7.27	0.00	16.97	40.68	33.14	32.51	21.93	27.05	25.02	5.74
	2	64.11	68.12	60.10	0.00	62.17	204.89	116.00	71.04	34.91	33.87	45.43	44.80	0.00	47.53	126.76	126.84	105.64	45.97	32.78	29.29	26.09
	3	21.78	21.25	22.32	0.00	23.01	47.07	31.13	18.63	13.61	19.02	22.43	16.37	0.00	22.73	34.81	42.80	32.04	21.22	16.55	14.07	16.63
	4	8.53	8.40	8.66	0.00	7.97	21.42	7.38	5.49	7.65	7.23	11.69	6.75	0.00	5.40	12.83	11.29	11.85	9.29	16.57	10.69	0.00
	5	5.24	5.90	4.58	0.00	2.71	12.25	7.77	5.32	4.39	4.85	11.80	4.01	0.00	2.02	8.59	7.95	6.50	4.09	6.53	5.54	0.00
	6	5.08	5.89	4.28	0.00	2.12	5.08	5.94	6.21	4.00	9.85	10.79	8.99	0.00	2.05	6.28	7.66	5.52	4.96	3.21	4.67	4.18
	7	4.03	3.50	4.55	0.00	1.95	7.99	5.64	3.81	3.50	2.85	4.15	1.65	0.00	3.49	8.28	7.11	6.84	3.08	4.25	3.11	4.77
	8	3.70	4.02	3.37	0.00	3.46	5.24	2.17	4.12	4.43	5.29	3.10	8.40	0.00	3.52	5.77	5.31	4.77	3.16	2.91	2.79	2.13
	9	3.27	4.53	2.02	9.03	3.40	5.80	5.69	4.60	2.49	5.62	1.44	2.66	0.00	0.00	5.33	3.83	4.28	2.40	1.54	0.78	0.00
	10	5.00	5.64	4.35	16.53	3.72	8.68	5.73	4.98	3.82	3.99	0.62	2.70	0.00	3.11	5.79	5.99	7.67	5.07	5.71	4.82	1.00
	11	5.82	5.67	5.97	0.00	5.53	7.28	6.54	6.20	4.07	7.26	9.49	4.63	0.00	4.96	9.73	8.25	7.94	5.91	6.98	6.00	3.93
	12	6.37	5.83	6.91	0.00	7.97	14.44	6.87	7.91	4.43	4.50	4.17	2.21	0.00	1.54	11.99	10.41	11.47	8.67	7.60	5.39	5.11
	13	18.45	17.43	19.47	0.00	18.07	58.28	18.69	14.01	11.39	11.10	16.03	9.31	3.38	14.06	31.69	36.42	26.09	19.24	20.14	19.59	4.65
**Quarter**	1	34.63	35.22	34.04	0.00	33.47	101.53	57.97	34.46	19.33	22.19	26.39	21.62	0.00	28.15	65.36	64.94	54.87	29.11	25.58	22.97	15.35
	2	6.12	6.48	5.75	0.00	3.94	12.53	6.85	5.42	5.33	7.06	11.13	6.08	0.00	2.91	9.35	9.01	7.84	5.94	8.30	6.79	1.63
	3	3.81	4.27	3.35	5.14	3.31	6.95	4.80	4.49	3.29	4.49	2.09	3.91	0.00	2.75	6.28	5.06	5.52	3.18	3.51	2.08	1.78
	4	9.49	9.10	9.88	2.53	9.53	23.76	9.87	8.70	6.33	7.15	8.66	5.39	0.97	6.22	15.85	16.71	14.15	10.35	10.59	9.86	4.20
**Year**	15/16	13.44	13.68	13.19	1.93	12.50	35.96	19.68	13.18	8.53	10.16	12.00	9.17	0.26	9.93	24.05	23.79	20.47	12.11	11.97	10.41	5.71
**Episodes**	F/N	8663.00	4043.00	4620.00	3.00	163.00	1143.00	652.00	1084.00	571.00	237.00	144.00	46.00	1.00	124.00	732.00	771.00	1697.00	787.00	304.00	158.00	46.00

Mean weekly incidence rate per 100,000 Persons.

Hayfever/Allergic Rhinitis (ICD10: J30)

			All ages			Male									Female								
			Male & Female	Male	Female	<1yr	1-4yrs	5-14yrs	15-24yrs	25-44yrs	45-64yrs	65-74yrs	75-84yrs	85+yrs	<1yr	1-4yrs	5-14yrs	15-24yrs	25-44yrs	45-64yrs	65-74yrs	75-84yrs	85+yrs
**North**	Quarter	1	30.84	30.88	30.79	0.00	44.93	102.84	46.39	27.25	12.70	12.28	18.20	13.32	0.00	33.66	66.01	60.74	52.05	19.47	17.35	19.09	8.76
		2	4.67	5.14	4.19	0.00	5.51	10.30	4.40	5.63	3.06	6.12	6.11	5.16	0.00	1.44	7.16	5.98	7.15	4.16	6.28	3.31	2.26
		3	2.49	2.50	2.48	0.00	1.74	3.67	3.36	3.12	1.08	3.29	2.14	4.11	0.00	1.92	4.14	2.99	4.43	1.29	3.64	1.65	2.29
		4	5.67	5.43	5.91	0.00	4.64	13.87	5.64	5.87	5.06	3.70	4.13	5.92	0.00	3.16	11.92	7.37	10.60	4.78	7.72	6.49	1.14
	Year	15/16	10.82	10.88	10.75	0.00	14.03	32.32	14.77	10.38	5.47	6.30	7.58	7.10	0.00	9.92	22.11	19.05	18.41	7.37	8.73	7.61	3.57
	Episodes	F/N	1,767.00	815.00	952.00	0.00	39.00	239.00	136.00	192.00	114.00	52.00	32.00	11.00	0.00	26.00	163.00	166.00	322.00	147.00	78.00	40.00	10.00
**South**	Quarter	1	28.18	29.49	26.87	0.00	28.13	87.54	52.64	29.04	13.33	17.96	13.65	23.16	0.00	25.48	50.79	52.70	49.62	17.99	19.50	18.68	7.10
		2	5.67	6.22	5.12	0.00	5.18	14.08	8.41	4.71	4.23	4.23	4.93	10.23	0.00	1.37	7.82	10.44	7.74	4.43	5.05	6.49	2.75
		3	4.35	5.72	2.99	20.55	3.71	5.30	5.64	3.82	3.03	3.90	2.96	2.54	0.00	2.72	4.58	5.36	6.08	2.91	3.02	0.79	1.40
		4	8.92	9.12	8.72	0.00	7.55	23.74	12.91	8.17	3.03	9.59	9.88	7.25	0.00	6.64	13.60	18.78	12.94	8.53	7.23	6.64	4.13
	Year	15/16	11.73	12.57	10.88	5.04	11.08	32.50	19.77	11.37	5.85	8.93	7.89	10.73	0.00	9.01	19.09	21.77	18.98	8.46	8.67	8.12	3.85
	Episodes	F/N	1,926.00	933.00	993.00	2.00	35.00	271.00	187.00	208.00	113.00	68.00	32.00	17.00	0.00	27.00	149.00	196.00	338.00	163.00	69.00	40.00	11.00
**London**	Quarter	1	48.14	48.11	48.18	0.00	34.91	129.26	69.84	45.27	35.66	35.15	47.73	35.14	0.00	27.14	88.02	83.96	67.25	56.06	39.07	38.73	33.35
		2	9.31	9.96	8.67	0.00	1.26	17.86	8.37	7.23	9.45	13.36	26.03	6.08	0.00	4.90	13.99	11.22	9.70	8.20	15.30	14.68	0.00
		3	5.59	5.83	5.35	0.00	4.77	14.51	6.71	6.48	4.68	9.66	0.00	5.71	0.00	2.02	10.63	7.58	8.20	6.17	5.15	4.92	3.42
		4	14.88	14.00	15.75	0.00	17.81	40.03	14.34	11.60	13.06	8.55	15.32	5.30	3.87	9.47	24.78	22.28	18.07	21.07	18.49	17.53	6.21
	Year	15/16	19.39	19.37	19.41	0.00	14.75	50.22	24.62	17.53	15.66	16.53	22.14	12.91	1.02	10.86	34.18	31.09	25.66	22.84	19.48	18.94	10.66
	Episodes	F/N	2,960.00	1,342.00	1,618.00	0.00	55.00	376.00	164.00	439.00	208.00	52.00	39.00	9.00	1.00	38.00	245.00	230.00	685.00	290.00	72.00	45.00	12.00
**Midlands And East**	Quarter	1	31.35	32.39	30.30	0.00	25.92	86.50	63.02	36.29	15.62	23.36	25.97	14.86	0.00	26.29	56.61	62.37	50.55	22.92	26.42	15.37	12.19
		2	4.82	4.60	5.03	0.00	3.80	7.88	6.22	4.11	4.60	4.51	7.45	2.84	0.00	3.93	8.42	8.42	6.78	6.98	6.59	2.68	1.50
		3	2.81	3.04	2.58	0.00	3.00	4.31	3.48	4.54	4.38	1.12	3.27	3.27	0.00	4.34	5.78	4.29	3.36	2.34	2.21	0.95	0.00
		4	8.49	7.85	9.12	10.13	8.12	17.38	6.59	9.14	4.17	6.74	5.31	3.08	0.00	5.60	13.10	18.38	14.98	7.04	8.91	8.80	5.31
	Year	15/16	11.80	11.89	11.71	2.68	10.17	28.80	19.58	13.44	7.13	8.89	10.40	5.96	0.00	9.96	20.83	23.27	18.84	9.77	10.99	6.99	4.76
	Episodes	F/N	2,010.00	953.00	1,057.00	1.00	34.00	257.00	165.00	245.00	136.00	65.00	41.00	9.00	0.00	33.00	175.00	179.00	352.00	187.00	85.00	33.00	13.00

Mean weekly incidence rate per 100,000 Persons.

Herpes Simplex (ICD10: B00)

		All ages			Male									Female								
		Male & Female	Male	Female	<1yr	1-4yrs	5-14yrs	15-24yrs	25-44yrs	45-64yrs	65-74yrs	75-84yrs	85+yrs	<1yr	1-4yrs	5-14yrs	15-24yrs	25-44yrs	45-64yrs	65-74yrs	75-84yrs	85+yrs
**4 weekly**	1	3.63	2.11	5.15	0.00	2.91	3.29	1.80	3.34	3.22	3.19	1.27	0.00	0.00	7.73	3.58	11.08	8.69	7.95	3.95	2.18	1.17
	2	3.47	1.78	5.17	0.00	1.01	3.49	2.74	1.87	3.56	2.42	0.95	0.00	0.00	7.25	3.36	10.21	11.13	5.59	4.05	4.92	0.00
	3	3.86	1.92	5.79	0.00	1.04	2.52	3.68	5.14	2.15	0.99	1.80	0.00	0.00	8.43	0.93	13.70	8.41	6.08	6.83	5.55	2.18
	4	3.22	1.76	4.67	0.00	1.05	1.84	2.97	3.35	3.53	2.24	0.88	0.00	0.00	3.23	2.83	8.40	11.58	6.90	4.81	3.03	1.28
	5	3.61	2.38	4.85	0.00	3.68	3.27	3.98	3.64	2.41	1.28	3.14	0.00	0.00	3.97	2.36	10.96	11.73	6.43	2.61	3.22	2.36
	6	4.00	2.80	5.21	0.00	9.28	3.36	1.95	3.19	4.11	2.53	0.78	0.00	0.00	1.94	6.82	11.97	11.90	8.10	2.55	2.36	1.22
	7	4.41	3.28	5.55	0.00	9.30	4.30	4.39	2.22	2.66	1.12	1.77	3.75	0.00	5.09	4.02	10.30	13.06	8.53	6.22	2.74	0.00
	8	4.06	3.28	4.85	0.00	11.69	5.12	3.49	3.14	2.70	0.32	0.61	2.48	0.00	3.70	4.86	9.68	12.85	5.57	5.64	0.45	0.86
	9	3.16	1.40	4.92	0.00	1.50	2.06	1.85	2.11	1.42	1.11	0.63	1.94	0.00	4.98	2.51	13.29	12.46	5.22	3.79	2.01	0.00
	10	4.30	2.80	5.81	0.00	5.23	3.55	4.78	3.56	3.01	1.69	3.34	0.00	0.00	2.32	2.82	14.52	15.67	5.30	4.49	2.23	4.91
	11	4.74	3.40	6.08	6.88	3.33	3.60	3.88	3.86	3.33	0.97	4.79	0.00	0.00	3.89	3.85	14.92	12.42	10.52	3.12	2.29	3.72
	12	3.82	2.78	4.86	0.00	3.94	2.42	3.20	4.23	2.92	1.67	1.75	4.91	3.70	3.37	3.60	7.58	11.11	7.65	3.79	1.77	1.19
	13	3.94	1.97	5.92	0.00	4.68	3.37	3.58	2.33	2.36	1.37	0.00	0.00	0.00	0.77	5.13	8.76	13.97	9.06	8.55	4.27	2.77
**Quarter**	1	3.65	1.95	5.35	0.00	1.75	3.11	2.67	3.44	2.99	2.27	1.33	0.00	0.00	7.80	2.69	11.62	9.35	6.65	4.87	4.06	1.12
	2	3.70	2.40	4.99	0.00	5.32	2.95	3.36	3.30	3.25	1.95	1.48	0.00	0.00	3.05	3.93	10.77	11.97	7.23	3.61	2.87	1.49
	3	3.84	2.60	5.08	0.00	6.69	3.49	3.43	2.53	2.37	0.98	1.40	2.52	0.00	4.24	4.00	11.36	13.30	6.31	4.66	1.59	0.27
	4	4.22	2.74	5.71	1.96	4.18	3.41	3.43	3.63	2.91	1.36	2.38	1.40	1.06	2.74	3.71	10.99	12.76	8.36	5.33	2.82	3.59
**Year**	15/16	3.86	2.43	5.29	0.52	4.48	3.24	3.22	3.23	2.88	1.64	1.66	0.99	0.28	4.42	3.59	11.18	11.86	7.16	4.63	2.83	1.66
**Episodes**	F/N	3208.00	859.00	2349.00	1.00	68.00	112.00	123.00	264.00	213.00	47.00	25.00	6.00	1.00	58.00	115.00	428.00	1015.00	541.00	129.00	47.00	15.00

Mean weekly incidence rate per 100,000 Persons.

Herpes Simplex (ICD10: B00)

			All ages			Male									Female								
			Male & Female	Male	Female	<1yr	1-4yrs	5-14yrs	15-24yrs	25-44yrs	45-64yrs	65-74yrs	75-84yrs	85+yrs	<1yr	1-4yrs	5-14yrs	15-24yrs	25-44yrs	45-64yrs	65-74yrs	75-84yrs	85+yrs
**North**	Quarter	1	4.53	2.25	6.81	0.00	1.95	4.54	2.20	3.48	2.73	3.11	2.25	0.00	0.00	13.75	4.64	17.26	10.84	6.65	4.53	1.85	1.81
		2	4.73	3.38	6.09	0.00	11.24	3.72	5.48	3.60	3.27	1.55	1.53	0.00	0.00	4.12	3.09	16.39	14.00	6.52	4.78	4.42	1.52
		3	4.75	3.89	5.61	0.00	9.91	5.78	3.95	2.59	2.77	0.75	2.27	7.01	0.00	8.03	2.96	11.05	13.78	6.85	4.86	1.91	1.06
		4	4.32	2.88	5.76	0.00	4.97	5.37	3.55	2.60	3.19	3.51	2.71	0.00	0.00	4.57	3.43	14.56	13.20	8.79	3.93	1.07	2.33
	Year	15/16	4.58	3.10	6.07	0.00	6.98	4.86	3.79	3.06	3.00	2.26	2.20	1.72	0.00	7.56	3.53	14.81	12.96	7.23	4.51	2.29	1.69
	Episodes	F/N	1,079.00	301.00	778.00	0.00	30.00	48.00	49.00	67.00	72.00	21.00	11.00	3.00	0.00	25.00	32.00	188.00	297.00	175.00	43.00	13.00	5.00
**South**	Quarter	1	3.73	1.96	5.49	0.00	1.28	2.06	3.57	3.59	2.19	2.84	2.13	0.00	0.00	9.09	2.79	10.10	8.61	6.95	4.31	4.92	2.68
		2	3.52	2.00	5.04	0.00	3.93	1.49	2.91	3.17	2.91	2.66	0.96	0.00	0.00	4.11	3.60	8.42	12.70	9.42	2.49	1.70	2.88
		3	3.61	1.96	5.26	0.00	6.23	3.03	3.74	1.32	2.56	0.76	0.00	0.00	0.00	1.40	4.25	11.52	16.50	5.28	6.76	1.66	0.00
		4	4.52	3.64	5.39	7.86	6.52	1.69	3.30	4.14	2.49	1.41	2.85	2.52	0.00	2.43	3.60	13.49	13.36	7.44	4.04	1.43	2.76
	Year	15/16	3.86	2.42	5.30	2.08	4.53	2.06	3.38	3.08	2.54	1.91	1.51	0.67	0.00	4.22	3.56	10.93	12.80	7.28	4.39	2.41	2.09
	Episodes	F/N	754.00	192.00	562.00	1.00	15.00	17.00	33.00	56.00	49.00	14.00	6.00	1.00	0.00	12.00	28.00	99.00	229.00	141.00	35.00	12.00	6.00
**London**	Quarter	1	3.70	2.41	4.99	0.00	3.76	3.18	3.42	4.09	4.62	2.60	0.00	0.00	0.00	2.52	2.44	10.56	9.26	5.38	8.63	6.13	0.00
		2	3.60	2.71	4.50	0.00	4.75	4.59	3.96	2.58	4.84	1.30	2.33	0.00	0.00	0.00	5.91	10.41	11.81	6.42	2.34	3.59	0.00
		3	3.65	2.51	4.78	0.00	6.49	3.51	3.66	2.81	2.75	1.15	2.18	0.00	0.00	6.03	5.47	9.83	11.86	5.56	4.31	0.00	0.00
		4	4.23	1.83	6.63	0.00	2.53	2.51	3.62	3.26	2.66	0.00	1.91	0.00	4.23	2.62	3.43	7.74	11.55	9.92	6.50	4.40	9.29
	Year	15/16	3.80	2.35	5.25	0.00	4.35	3.43	3.67	3.19	3.70	1.24	1.61	0.00	1.12	2.79	4.29	9.60	11.13	6.88	5.47	3.55	2.45
	Episodes	F/N	765.00	208.00	557.00	0.00	17.00	26.00	26.00	82.00	50.00	4.00	3.00	0.00	1.00	11.00	33.00	75.00	313.00	93.00	20.00	8.00	3.00
**Midlands And East**	Quarter	1	2.64	1.19	4.10	0.00	0.00	2.66	1.49	2.59	2.43	0.54	0.95	0.00	0.00	5.83	0.90	8.56	8.70	7.61	2.00	3.33	0.00
		2	2.93	1.52	4.34	0.00	1.36	2.01	1.08	3.83	1.99	2.30	1.08	0.00	0.00	3.96	3.12	7.86	9.35	6.57	4.83	1.77	1.57
		3	3.35	2.04	4.66	0.00	4.13	1.64	2.35	3.39	1.39	1.26	1.15	3.05	0.00	1.49	3.33	13.04	11.04	7.56	2.72	2.78	0.00
		4	3.83	2.61	5.04	0.00	2.70	4.09	3.25	4.53	3.29	0.52	2.06	3.09	0.00	1.34	4.40	8.16	12.91	7.28	6.87	4.37	0.00
	Year	15/16	3.20	1.85	4.54	0.00	2.06	2.63	2.07	3.60	2.29	1.14	1.33	1.57	0.00	3.12	2.97	9.38	10.54	7.25	4.16	3.09	0.39
	Episodes	F/N	610.00	158.00	452.00	0.00	6.00	21.00	15.00	59.00	42.00	8.00	5.00	2.00	0.00	10.00	22.00	66.00	176.00	132.00	31.00	14.00	1.00

Mean weekly incidence rate per 100,000 Persons.

Herpes Zoster (ICD10: B02)

		All ages			Male									Female								
		Male & Female	Male	Female	<1yr	1-4yrs	5-14yrs	15-24yrs	25-44yrs	45-64yrs	65-74yrs	75-84yrs	85+yrs	<1yr	1-4yrs	5-14yrs	15-24yrs	25-44yrs	45-64yrs	65-74yrs	75-84yrs	85+yrs
**4 weekly**	1	9.19	6.79	11.59	0.00	0.94	5.20	1.43	2.83	5.67	13.62	10.59	20.83	0.00	1.01	3.43	3.60	4.20	12.11	21.88	24.72	33.33
	2	8.42	6.78	10.06	0.00	1.00	3.09	1.66	2.73	4.23	7.74	22.92	17.65	0.00	2.38	3.17	5.19	6.07	7.67	19.00	21.21	25.81
	3	8.65	8.69	8.61	0.00	1.04	3.13	2.50	5.75	5.88	10.61	26.00	23.27	0.00	0.90	3.34	1.78	3.47	10.52	16.02	19.87	21.57
	4	9.18	7.72	10.64	0.00	1.04	5.36	5.97	4.76	5.47	11.82	16.32	18.77	0.00	3.32	1.81	3.78	3.77	11.43	18.15	25.05	28.43
	5	8.42	7.65	9.19	0.00	1.80	1.87	3.46	3.37	7.80	12.07	23.57	14.94	0.00	1.03	5.39	2.41	5.28	10.36	15.03	29.60	13.63
	6	8.23	5.97	10.49	0.00	0.00	2.45	6.32	2.63	5.78	9.90	15.78	10.86	0.00	1.20	3.83	4.99	5.66	12.98	18.85	25.43	21.48
	7	8.34	6.09	10.59	0.00	0.00	4.19	3.07	3.87	7.33	12.78	13.41	10.11	0.00	1.13	5.44	2.32	5.74	14.54	12.47	31.72	21.97
	8	8.66	7.49	9.83	0.00	1.19	1.68	1.96	3.06	6.42	17.19	14.14	21.78	0.00	0.00	3.92	1.99	4.69	11.13	15.54	23.92	27.33
	9	6.92	5.65	8.20	0.00	3.16	1.78	1.96	4.14	3.67	14.45	15.76	5.95	0.00	0.00	3.61	1.47	4.19	13.40	17.81	17.37	15.92
	10	9.03	6.06	12.00	0.00	1.78	3.17	2.46	3.24	7.09	11.72	13.84	11.27	0.00	1.99	5.99	4.95	6.17	10.12	19.22	18.74	40.78
	11	11.09	10.94	11.24	0.00	0.00	2.36	4.53	4.35	7.89	16.13	23.36	39.82	0.00	2.27	5.68	4.40	6.29	11.70	14.31	23.16	33.35
	12	7.46	5.75	9.17	0.00	0.00	2.88	1.67	1.93	7.37	13.78	15.35	8.77	0.00	1.54	3.18	2.42	5.34	9.08	19.02	16.12	25.81
	13	9.10	7.73	10.46	0.00	0.00	3.45	3.57	2.55	7.73	11.78	28.71	11.78	0.00	0.70	5.72	3.92	7.07	9.11	14.90	16.32	36.42
**Quarter**	1	8.78	7.37	10.20	0.00	0.99	3.91	1.83	3.70	5.29	10.89	19.12	20.60	0.00	1.40	3.32	3.53	4.55	10.25	19.19	22.15	27.40
	2	8.43	6.92	9.95	0.00	0.87	3.27	4.85	3.66	6.42	10.90	17.95	14.34	0.00	1.71	3.67	3.59	4.77	11.90	16.97	26.40	20.51
	3	8.33	6.62	10.03	0.00	1.34	2.62	2.39	3.50	5.79	15.34	15.47	13.11	0.00	0.73	4.97	2.13	5.10	12.49	16.22	23.09	25.57
	4	9.13	7.73	10.54	0.00	0.51	2.86	3.28	3.03	7.67	13.24	20.47	18.53	0.00	1.51	4.70	4.02	6.32	9.89	16.40	19.02	32.96
**Year**	15/16	8.68	7.17	10.19	0.00	0.92	3.16	3.09	3.46	6.32	12.60	18.29	16.68	0.00	1.34	4.18	3.33	5.21	11.11	17.18	22.60	26.73
**Episodes**	F/N	4421.00	1699.00	2722.00	0.00	12.00	110.00	122.00	285.00	485.00	339.00	250.00	96.00	0.00	17.00	137.00	122.00	434.00	841.00	491.00	418.00	262.00

Mean weekly incidence rate per 100,000 Persons.

Herpes Zoster (ICD10: B02)

			All ages			Male									Female								
			Male & Female	Male	Female	<1yr	1-4yrs	5-14yrs	15-24yrs	25-44yrs	45-64yrs	65-74yrs	75-84yrs	85+yrs	<1yr	1-4yrs	5-14yrs	15-24yrs	25-44yrs	45-64yrs	65-74yrs	75-84yrs	85+yrs
**North**	Quarter	1	9.05	6.82	11.29	0.00	0.00	2.56	0.55	4.00	5.66	13.80	8.03	26.75	0.00	1.80	5.79	3.60	4.69	10.95	24.81	24.73	25.24
		2	9.94	8.05	11.83	0.00	1.07	7.05	7.04	3.64	6.96	12.52	15.61	18.55	0.00	1.44	6.13	3.18	6.89	12.86	17.93	34.96	23.12
		3	7.88	6.00	9.75	0.00	1.21	4.13	4.39	2.53	4.75	11.59	9.72	15.71	0.00	0.00	5.72	1.70	6.62	13.36	12.95	22.93	24.51
		4	10.06	7.94	12.17	0.00	0.00	4.26	2.99	2.31	8.38	11.92	20.13	21.43	0.00	2.56	5.13	4.02	6.65	11.02	13.41	22.07	44.71
	Year	15/16	9.25	7.22	11.28	0.00	0.56	4.49	3.73	3.10	6.48	12.45	13.50	20.63	0.00	1.47	5.68	3.14	6.22	12.03	17.20	26.09	29.69
	Episodes	F/N	1,468.00	538.00	930.00	0.00	2.00	43.00	52.00	68.00	159.00	114.00	68.00	32.00	0.00	5.00	52.00	39.00	142.00	288.00	160.00	153.00	91.00
**South**	Quarter	1	10.59	10.07	11.11	0.00	2.73	4.65	1.68	3.49	4.21	8.50	22.45	42.88	0.00	1.55	3.78	4.76	3.88	10.43	16.99	28.07	30.50
		2	9.44	8.16	10.72	0.00	1.28	4.44	5.38	4.14	8.63	16.24	18.05	15.24	0.00	2.85	2.60	3.61	5.86	13.05	20.35	26.02	22.14
		3	9.85	7.84	11.87	0.00	1.76	3.48	1.88	3.55	8.13	20.88	17.99	12.88	0.00	1.39	6.55	1.78	5.95	10.74	17.81	25.30	37.27
		4	10.67	9.49	11.84	0.00	1.18	2.63	3.45	3.34	8.14	13.43	20.14	33.12	0.00	1.26	4.71	4.75	8.98	11.57	19.79	24.11	31.40
	Year	15/16	10.15	8.90	11.39	0.00	1.73	3.78	3.11	3.62	7.29	14.74	19.67	26.16	0.00	1.75	4.42	3.74	6.22	11.45	18.75	25.84	30.35
	Episodes	F/N	1,270.00	504.00	766.00	0.00	5.00	31.00	30.00	65.00	141.00	112.00	80.00	40.00	0.00	5.00	35.00	34.00	112.00	219.00	149.00	127.00	85.00
**London**	Quarter	1	7.73	6.48	8.98	0.00	1.23	3.71	2.61	4.71	6.41	12.98	26.66	0.00	0.00	0.00	1.41	0.59	2.50	8.08	21.26	15.92	31.10
		2	6.96	5.78	8.15	0.00	1.14	0.61	3.80	3.74	5.47	7.96	23.29	6.00	0.00	0.00	1.81	4.65	2.84	10.88	13.24	21.72	18.22
		3	7.78	6.29	9.27	0.00	0.92	1.35	3.27	3.58	3.77	16.62	21.57	5.50	0.00	0.00	3.57	4.41	3.15	11.49	18.29	22.72	19.85
		4	7.64	7.37	7.91	0.00	0.86	2.08	1.78	3.14	4.83	16.06	26.87	10.69	0.00	0.87	2.56	3.23	3.94	6.87	13.91	8.75	31.10
	Year	15/16	7.53	6.49	8.57	0.00	1.03	1.94	2.85	3.78	5.11	13.46	24.64	5.64	0.00	0.23	2.34	3.22	3.12	9.28	16.62	17.12	25.18
	Episodes	F/N	687.00	298.00	389.00	0.00	4.00	15.00	20.00	98.00	69.00	44.00	44.00	4.00	0.00	1.00	18.00	26.00	89.00	122.00	62.00	41.00	30.00
**Midlands And East**	Quarter	1	7.77	6.12	9.41	0.00	0.00	4.74	2.48	2.58	4.89	8.26	19.36	12.76	0.00	2.23	2.31	5.16	7.13	11.55	13.71	19.89	22.75
		2	7.38	5.69	9.08	0.00	0.00	0.99	3.16	3.13	4.62	6.88	14.83	17.57	0.00	2.53	4.16	2.91	3.48	10.80	16.37	22.91	18.57
		3	7.79	6.34	9.24	0.00	1.46	1.52	0.00	4.34	6.53	12.25	12.62	18.35	0.00	1.51	4.03	0.64	4.68	14.35	15.84	21.43	20.65
		4	8.17	6.13	10.21	0.00	0.00	2.49	4.88	3.33	9.34	11.56	14.73	8.88	0.00	1.34	6.41	4.08	5.73	10.09	18.51	21.15	24.62
	Year	15/16	7.79	6.07	9.50	0.00	0.36	2.44	2.67	3.35	6.40	9.77	15.37	14.28	0.00	1.89	4.27	3.21	5.26	11.67	16.15	21.34	21.70
	Episodes	F/N	996.00	359.00	637.00	0.00	1.00	21.00	20.00	54.00	116.00	69.00	58.00	20.00	0.00	6.00	32.00	23.00	91.00	212.00	120.00	97.00	56.00

Mean weekly incidence rate per 100,000 Persons.

Impetigo (ICD10 : L01)

		All ages			Male									Female								
		Male & Female	Male	Female	<1yr	1-4yrs	5-14yrs	15-24yrs	25-44yrs	45-64yrs	65-74yrs	75-84yrs	85+yrs	<1yr	1-4yrs	5-14yrs	15-24yrs	25-44yrs	45-64yrs	65-74yrs	75-84yrs	85+yrs
4 weekly	1	6.96	6.88	7.03	0.00	33.94	17.04	4.69	1.70	1.86	0.76	0.00	1.95	0.00	25.69	18.24	7.50	3.40	1.59	1.85	3.35	1.68
	2	6.67	6.40	6.93	0.00	23.60	16.99	6.77	3.77	0.33	1.01	2.52	2.65	0.00	21.63	21.58	4.49	4.39	3.22	0.00	1.31	5.78
	3	13.48	8.46	18.51	0.00	33.27	27.21	6.68	2.94	1.30	1.36	3.39	0.00	97.66	31.77	19.41	5.85	4.37	2.55	2.63	0.00	2.33
	4	7.76	8.50	7.02	0.00	34.88	20.59	6.91	2.79	1.84	0.46	4.24	4.77	0.00	26.89	19.44	4.92	5.94	1.73	2.11	2.10	0.00
	5	9.62	10.64	8.60	27.90	34.24	16.76	5.91	1.67	2.26	2.14	4.85	0.00	0.00	33.68	20.57	10.65	5.10	2.24	1.59	0.00	3.57
	6	9.92	9.72	10.12	0.00	54.58	23.44	4.69	1.45	1.43	1.33	0.56	0.00	0.00	48.78	23.53	7.87	5.46	2.40	0.73	1.44	0.88
	7	12.26	12.57	11.95	9.26	72.37	21.37	4.69	1.83	1.53	1.36	0.71	0.00	12.94	56.57	18.29	6.55	3.86	1.80	1.49	0.84	5.24
	8	13.29	13.08	13.50	9.22	67.85	24.96	3.68	2.91	1.61	0.91	1.64	4.91	24.44	55.59	25.73	3.90	4.31	3.84	1.54	1.32	0.86
	9	11.01	11.85	10.16	14.64	49.47	24.68	5.21	2.08	1.48	2.43	1.58	5.05	12.06	36.61	21.92	7.14	4.43	2.97	2.72	1.19	2.45
	10	11.68	10.81	12.56	13.58	51.31	20.85	4.73	2.71	1.77	1.43	0.94	0.00	16.71	42.11	26.16	9.07	4.13	3.61	2.43	2.66	6.14
	11	12.87	14.12	11.62	48.99	41.06	22.21	5.92	1.52	2.16	0.79	2.18	2.26	17.98	45.92	18.62	10.29	6.22	3.31	1.51	0.78	0.00
	12	10.58	11.26	9.90	18.65	45.62	21.27	5.42	1.80	1.25	1.35	1.40	4.61	15.75	32.53	22.55	4.72	3.24	2.84	1.93	2.21	3.30
	13	10.24	10.07	10.40	13.48	34.46	24.20	4.30	2.72	2.92	2.76	5.82	0.00	16.75	39.57	20.68	6.25	3.97	2.09	1.76	1.45	1.09
Quarter	1	8.88	7.22	10.53	0.00	30.55	20.15	5.94	2.71	1.22	1.02	1.82	1.57	30.05	26.31	19.63	6.07	4.00	2.39	1.52	1.69	3.14
	2	9.29	9.78	8.80	8.59	43.49	20.37	5.89	2.14	1.86	1.21	2.97	1.47	0.00	38.09	21.07	8.21	5.38	2.09	1.57	1.09	1.71
	3	12.22	12.28	12.16	11.64	61.49	22.90	4.25	2.12	1.69	1.88	1.50	3.07	17.66	48.87	23.08	5.82	4.51	3.27	1.80	1.85	2.59
	4	11.23	11.69	10.77	25.71	41.32	22.76	5.29	2.18	1.91	1.40	2.69	1.97	16.93	38.75	21.14	7.36	4.15	2.69	1.96	1.27	2.72
Year	15/16	10.42	10.27	10.57	11.75	44.16	21.57	5.34	2.29	1.67	1.38	2.25	2.02	16.17	38.02	21.23	6.87	4.50	2.61	1.72	1.47	2.55
Episodes	F/N	4109.00	1954.00	2155.00	23.00	605.00	734.00	197.00	183.00	128.00	38.00	34.00	12.00	21.00	505.00	686.00	268.00	368.00	202.00	55.00	25.00	25.00

Mean weekly incidence rate per 100,000 Persons.

Impetigo (ICD10: L01)

			All ages			Male									Female								
			Male & Female	Male	Female	<1yr	1-4yrs	5-14yrs	15-24yrs	25-44yrs	45-64yrs	65-74yrs	75-84yrs	85+yrs	<1yr	1-4yrs	5-14yrs	15-24yrs	25-44yrs	45-64yrs	65-74yrs	75-84yrs	85+yrs
**North**	Quarter	1	8.37	9.17	7.57	0.00	35.37	27.88	5.59	4.55	1.24	2.44	2.25	3.26	0.00	19.50	28.56	7.83	5.52	2.68	1.21	1.04	1.77
		2	10.14	8.92	11.35	0.00	40.40	24.90	5.01	2.51	1.15	1.03	2.34	2.94	0.00	56.30	22.36	11.83	6.61	2.46	1.55	0.00	1.08
		3	12.65	11.85	13.45	7.47	60.24	20.75	4.40	1.88	1.44	0.47	1.66	8.38	14.85	56.90	24.23	7.61	6.18	3.40	1.67	1.27	4.92
		4	10.80	11.45	10.16	22.99	34.29	28.31	5.62	2.24	2.43	1.03	4.01	2.14	19.76	26.20	23.59	8.69	4.63	3.30	1.99	0.00	3.25
	Year	15/16	10.50	10.37	10.62	7.91	42.42	25.51	5.16	2.79	1.58	1.24	2.59	4.14	8.86	39.47	24.66	8.98	5.71	2.97	1.61	0.57	2.76
	Episodes	F/N	1,330.00	610.00	720.00	6.00	165.00	244.00	65.00	59.00	41.00	10.00	13.00	7.00	6.00	149.00	222.00	119.00	125.00	71.00	16.00	3.00	9.00
**South**	Quarter	1	7.32	7.29	7.35	0.00	30.65	17.10	7.27	2.07	0.95	0.52	4.04	3.01	0.00	27.01	13.71	10.03	4.62	3.11	3.28	1.62	2.74
		2	11.48	13.09	9.86	34.34	47.49	21.88	4.97	1.56	1.47	2.14	4.00	0.00	0.00	47.04	19.48	9.06	5.79	2.08	3.10	0.82	1.38
		3	14.20	15.91	12.49	21.90	80.73	27.50	5.41	2.30	0.44	1.06	0.00	3.88	9.76	57.43	23.38	5.82	6.12	3.68	3.74	2.51	0.00
		4	12.14	13.29	10.98	31.84	47.70	25.78	4.21	2.76	1.10	1.86	1.81	2.59	10.94	44.61	23.53	8.50	3.46	2.80	2.28	1.47	1.24
	Year	15/16	11.30	12.42	10.19	22.20	51.57	23.12	5.44	2.19	0.99	1.41	2.45	2.37	5.28	44.03	20.09	8.35	4.97	2.91	3.08	1.60	1.34
	Episodes	F/N	1,046.00	498.00	548.00	7.00	164.00	193.00	51.00	40.00	19.00	11.00	10.00	3.00	3.00	131.00	159.00	74.00	89.00	56.00	24.00	8.00	4.00
**London**	Quarter	1	11.70	4.60	18.80	0.00	20.73	15.65	1.94	1.96	1.08	0.00	0.00	0.00	120.19	26.64	14.59	2.40	2.34	1.34	0.00	1.73	0.00
		2	6.50	6.54	6.47	0.00	33.30	18.07	1.98	1.85	1.32	0.00	2.30	0.00	0.00	23.97	19.88	4.06	2.53	1.65	0.00	1.77	4.39
		3	9.56	10.18	8.94	17.20	41.97	21.74	2.61	1.56	2.24	2.29	2.02	0.00	17.11	33.93	15.55	4.15	2.36	2.04	0.00	1.62	3.69
		4	8.78	8.23	9.33	20.76	27.33	14.59	3.64	1.12	1.70	1.07	3.85	0.00	26.98	28.70	15.46	3.62	3.46	1.78	0.93	0.00	3.05
	Year	15/16	9.13	7.40	10.86	9.70	30.77	17.46	2.56	1.61	1.59	0.85	2.08	0.00	40.81	28.32	16.35	3.56	2.69	1.71	0.25	1.26	2.79
	Episodes	F/N	727.00	352.00	375.00	7.00	119.00	136.00	19.00	42.00	22.00	3.00	4.00	0.00	9.00	107.00	124.00	29.00	76.00	23.00	1.00	3.00	3.00
**Midlands And East**	Quarter	1	8.12	7.82	8.41	0.00	35.47	19.98	8.96	2.28	1.58	1.13	0.99	0.00	0.00	32.09	21.66	4.00	3.54	2.41	1.59	2.37	8.06
		2	9.04	10.56	7.53	0.00	52.78	16.64	11.61	2.63	3.53	1.66	3.23	2.93	0.00	25.07	22.58	7.89	6.60	2.18	1.64	1.77	0.00
		3	12.47	11.18	13.75	0.00	63.02	21.62	4.58	2.74	2.64	3.72	2.31	0.00	28.90	47.21	29.14	5.68	3.36	3.96	1.77	1.99	1.77
		4	13.21	13.80	12.63	27.25	55.98	22.36	7.71	2.60	2.42	1.65	1.08	3.14	10.03	55.48	21.98	8.64	5.03	2.87	2.64	3.62	3.34
	Year	15/16	10.76	10.89	10.62	7.20	51.89	20.19	8.20	2.56	2.54	2.03	1.88	1.55	9.74	40.26	23.80	6.59	4.64	2.86	1.92	2.46	3.30
	Episodes	F/N	1,006.00	494.00	512.00	3.00	157.00	161.00	62.00	42.00	46.00	14.00	7.00	2.00	3.00	118.00	181.00	46.00	78.00	52.00	14.00	11.00	9.00

Mean weekly incidence rate per 100,000 Persons.

Infections of Skin & Subcutaneous Tissue (ICD10: L00-L08)

		All ages			Male									Female								
		Male & Female	Male	Female	<1yr	1-4yrs	5-14yrs	15-24yrs	25-44yrs	45-64yrs	65-74yrs	75-84yrs	85+yrs	<1yr	1-4yrs	5-14yrs	15-24yrs	25-44yrs	45-64yrs	65-74yrs	75-84yrs	85+yrs
**4 weekly**	1	64.70	59.14	70.26	0.00	71.99	49.86	35.68	39.92	43.22	63.99	96.67	130.92	0.00	63.92	52.63	40.21	51.77	54.29	70.76	110.04	188.76
	2	145.56	62.30	228.83	0.00	79.40	52.71	31.82	44.69	46.07	56.91	83.33	165.78	1388.89	52.69	54.06	53.61	61.55	67.05	64.56	119.92	197.13
	3	110.49	97.53	123.45	196.32	83.06	82.62	47.94	52.33	65.05	87.24	111.16	152.06	331.44	85.86	60.04	60.68	67.89	82.38	79.52	118.47	224.78
	4	98.56	93.21	103.91	183.83	82.24	50.33	52.45	49.10	55.90	81.30	117.41	166.33	156.69	71.38	53.02	58.09	67.91	76.67	102.15	124.36	224.93
	5	91.29	91.97	90.62	267.62	59.92	48.03	50.39	40.54	49.70	65.69	121.64	124.15	161.04	69.62	64.76	58.54	59.54	60.18	68.60	107.50	165.78
	6	73.16	71.73	74.59	73.33	89.52	50.53	35.58	41.27	47.15	66.44	109.70	132.00	27.01	82.77	67.38	45.39	54.55	58.20	66.94	108.92	160.18
	7	81.13	77.24	85.01	78.01	116.28	55.41	41.21	42.59	51.21	68.38	95.74	146.36	111.80	81.19	49.82	47.27	47.10	54.25	70.96	104.77	197.94
	8	76.86	73.85	79.88	84.14	108.89	54.47	38.49	41.13	41.90	61.69	94.91	139.02	66.89	93.64	57.47	48.62	45.32	54.84	59.22	105.37	187.53
	9	74.28	70.06	78.50	96.74	105.50	56.05	34.91	35.25	40.29	45.84	73.71	142.28	89.71	80.57	51.12	40.17	46.52	44.74	58.56	99.16	195.95
	10	76.85	70.02	83.68	98.82	96.08	48.98	39.58	41.72	44.70	57.91	81.91	120.47	79.13	82.33	62.52	52.99	50.63	47.95	71.15	98.14	208.32
	11	86.77	90.01	83.53	189.36	83.37	56.78	41.87	39.68	45.56	61.24	91.15	201.04	115.24	77.05	56.23	54.51	50.77	55.28	68.37	90.40	183.90
	12	73.49	71.52	75.45	80.66	83.49	51.13	36.31	30.94	49.62	59.56	93.87	158.11	63.67	68.63	53.64	37.23	48.45	48.76	63.88	98.46	196.37
	13	79.40	74.32	84.47	79.38	77.03	57.04	38.05	34.15	46.57	67.72	100.17	168.79	119.79	70.03	56.09	46.90	50.82	55.56	74.45	112.66	173.94
**Quarter**	1	103.67	71.92	135.42	60.41	77.67	60.82	38.26	45.21	50.81	68.96	97.02	148.15	529.33	67.21	55.35	50.63	59.74	66.86	71.55	115.67	202.42
	2	87.25	85.30	89.19	170.67	81.47	49.56	45.21	43.26	51.58	72.07	115.64	138.26	112.50	74.81	60.87	53.06	59.72	63.60	81.04	113.41	183.76
	3	76.97	72.88	81.06	86.02	104.45	54.59	38.76	40.34	43.82	55.65	85.72	146.52	80.21	86.96	55.00	46.35	47.03	51.45	62.40	104.03	196.12
	4	79.45	77.10	81.81	113.62	85.17	54.35	38.84	35.61	46.45	63.05	93.09	163.71	104.20	71.85	55.85	47.61	49.91	52.15	68.03	98.01	188.65
**Year**	15/16	86.70	76.81	96.59	107.79	87.15	54.82	40.24	41.00	48.13	64.90	97.78	149.44	204.63	75.14	56.75	49.38	54.02	58.39	70.70	107.60	192.66
**Episodes**	F/N	34834.00	15614.00	19220.00	144.00	1198.00	1845.00	1483.00	3403.00	3607.00	1757.00	1396.00	781.00	128.00	995.00	1821.00	1817.00	4455.00	4301.00	2011.00	1888.00	1804.00

Mean weekly incidence rate per 100,000 Persons.

Infections of Skin & Subcutaneous Tissue (ICD10: L00-L08)

			All ages			Male									Female								
			Male & Female	Male	Female	<1yr	1-4yrs	5-14yrs	15-24yrs	25-44yrs	45-64yrs	65-74yrs	75-84yrs	85+yrs	<1yr	1-4yrs	5-14yrs	15-24yrs	25-44yrs	45-64yrs	65-74yrs	75-84yrs	85+yrs
**North**	Quarter	1	170.15	64.38	275.91	0.00	87.89	68.03	39.66	48.20	50.15	68.64	86.95	129.95	1,796.81	63.99	68.88	58.37	65.59	58.05	63.72	115.67	192.11
		2	87.79	87.26	88.31	193.35	81.46	50.24	36.68	49.04	51.80	77.32	106.40	139.10	88.19	94.61	64.04	62.69	65.39	65.06	82.07	103.21	169.52
		3	76.40	72.53	80.27	111.74	95.71	55.39	37.18	44.82	41.91	50.07	90.67	125.25	104.44	93.36	53.19	41.64	47.89	50.57	51.89	83.82	195.58
		4	79.39	77.16	81.62	91.56	86.88	61.81	38.10	37.94	47.35	64.40	91.25	175.10	120.11	63.25	56.44	46.13	54.34	52.81	63.79	99.08	178.67
	Year	15/16	102.98	75.37	130.59	99.02	87.96	58.92	37.91	44.86	47.80	65.09	93.77	142.97	519.70	78.51	60.56	52.09	58.23	56.55	65.34	100.42	183.87
	Episodes	F/N	10,746.00	4,866.00	5,880.00	43.00	336.00	558.00	490.00	1,027.00	1,125.00	597.00	458.00	232.00	43.00	285.00	545.00	653.00	1,257.00	1,322.00	633.00	588.00	554.00
**South**	Quarter	1	82.07	77.22	86.93	89.45	78.81	56.95	40.51	49.13	55.42	60.34	102.07	162.26	80.13	68.66	52.07	48.68	60.65	69.64	82.23	109.09	211.23
		2	84.65	85.28	84.01	163.47	90.99	57.11	46.30	40.67	56.05	68.84	109.68	134.42	49.09	93.83	55.33	51.94	60.84	68.42	77.19	125.95	173.52
		3	81.11	78.95	83.27	56.98	137.69	61.36	38.71	44.10	45.03	66.01	83.88	176.78	47.94	96.89	59.44	47.79	62.73	54.04	62.07	117.91	200.59
		4	86.74	86.00	87.47	136.96	90.18	64.13	39.40	39.04	53.89	64.68	103.76	181.95	91.18	74.00	61.04	60.23	53.52	57.98	69.21	107.06	213.02
	Year	15/16	83.70	81.94	85.46	112.19	99.24	59.97	41.20	43.16	52.62	64.96	99.92	164.20	67.54	83.17	57.05	52.31	59.32	62.44	72.61	114.85	199.84
	Episodes	F/N	9,318.00	4,180.00	5,138.00	32.00	313.00	498.00	392.00	782.00	1,012.00	495.00	405.00	251.00	21.00	247.00	447.00	471.00	1,054.00	1,201.00	576.00	562.00	559.00
**London**	Quarter	1	87.10	69.81	104.39	65.75	63.11	56.57	31.06	41.31	52.41	68.00	107.03	143.06	240.38	61.72	45.80	42.39	50.12	74.15	67.78	122.33	234.87
		2	93.81	87.41	100.21	175.42	61.92	48.74	45.17	36.17	48.39	78.01	144.90	147.99	203.11	51.78	66.51	41.00	52.30	63.41	85.70	122.84	215.28
		3	75.75	69.19	82.30	68.75	82.88	50.62	37.66	33.78	43.42	56.77	96.81	152.04	97.92	66.45	45.72	45.47	36.13	51.19	72.27	108.86	216.70
		4	71.87	67.50	76.24	101.81	67.09	36.25	32.11	29.94	41.41	60.96	92.00	145.95	92.16	64.45	48.56	37.19	38.32	48.60	76.94	87.68	192.26
	Year	15/16	81.94	73.37	90.51	102.91	68.72	47.82	36.42	35.20	46.31	65.84	109.84	147.24	157.14	61.16	51.59	41.43	44.10	59.13	75.70	109.99	214.35
	Episodes	F/N	6,730.00	2,987.00	3,743.00	40.00	265.00	366.00	257.00	915.00	631.00	211.00	201.00	101.00	42.00	230.00	387.00	325.00	1,211.00	759.00	282.00	260.00	247.00
**Midlands And East**	Quarter	1	75.36	76.28	74.44	86.43	80.88	61.72	41.83	42.18	45.27	78.87	92.04	157.33	0.00	74.48	54.65	53.08	62.60	65.59	72.46	115.60	171.46
		2	82.75	81.25	84.24	150.43	91.52	42.16	52.68	47.18	50.08	64.11	101.56	131.54	109.59	59.01	57.58	56.59	60.34	57.51	79.20	101.65	176.71
		3	74.62	70.83	78.41	106.61	101.52	51.01	41.49	38.67	44.92	49.74	71.51	132.03	70.52	91.12	61.65	50.50	41.39	49.99	63.36	105.53	171.59
		4	79.81	77.74	81.89	124.12	96.51	55.21	45.74	35.54	43.13	62.17	85.35	151.83	113.34	85.69	57.36	46.91	53.45	49.21	62.19	98.23	170.64
	Year	15/16	78.17	76.55	79.78	117.03	92.68	52.58	45.44	40.79	45.80	63.69	87.57	143.35	74.12	77.73	57.80	51.68	54.43	55.45	69.17	105.12	172.56
	Episodes	F/N	8,040.00	3,581.00	4,459.00	29.00	284.00	423.00	344.00	679.00	839.00	454.00	332.00	197.00	22.00	233.00	442.00	368.00	933.00	1,019.00	520.00	478.00	444.00

Mean weekly incidence rate per 100,000 Persons.

Infectious Intestinal Disease (ICD10: A00-A09))

		All ages			Male									Female								
		Male & Female	Male	Female	<1yr	1-4yrs	5-14yrs	15-24yrs	25-44yrs	45-64yrs	65-74yrs	75-84yrs	85+yrs	<1yr	1-4yrs	5-14yrs	15-24yrs	25-44yrs	45-64yrs	65-74yrs	75-84yrs	85+yrs
**4 weekly**	1	12.79	12.24	13.35	0.00	50.84	12.13	8.98	8.41	4.40	6.56	5.38	13.42	0.00	53.07	13.88	7.83	8.23	5.60	7.86	8.99	14.70
	2	11.73	12.24	11.21	0.00	41.81	11.25	6.17	6.77	8.24	5.13	10.52	20.27	0.00	43.97	13.52	6.31	8.78	7.93	7.71	9.10	3.61
	3	13.41	16.13	10.68	77.16	26.84	10.71	6.14	6.77	5.15	3.84	4.44	4.12	0.00	39.94	10.35	12.67	8.90	9.79	6.51	4.38	3.64
	4	9.53	10.40	8.65	0.00	35.04	5.32	5.31	7.30	6.13	5.96	3.22	25.31	0.00	22.60	7.44	12.51	7.64	7.77	6.19	7.22	6.52
	5	10.93	10.93	10.92	15.13	37.70	10.68	4.25	5.41	5.19	8.68	7.27	4.06	20.63	26.34	7.88	7.99	6.45	3.69	8.51	4.57	12.23
	6	13.87	14.15	13.59	29.62	46.60	8.45	9.03	6.14	6.73	4.90	4.05	11.83	20.94	43.63	8.40	6.87	8.20	5.14	7.57	9.73	11.86
	7	18.20	21.35	15.05	107.57	37.09	13.46	2.50	4.81	4.78	7.24	7.06	7.63	34.37	43.21	9.02	7.15	8.38	6.31	9.20	7.76	10.10
	8	15.08	14.18	15.97	30.89	38.67	11.73	6.63	6.67	7.27	5.66	7.26	12.84	33.24	48.61	12.46	9.14	7.32	6.39	10.23	7.82	8.56
	9	11.09	8.79	13.38	6.46	28.61	10.13	4.85	5.28	5.02	8.09	8.75	1.94	44.74	24.55	9.00	6.82	7.46	5.26	7.42	9.12	6.07
	10	18.64	18.12	19.16	54.96	52.99	12.63	7.46	7.00	5.39	7.04	6.04	9.57	64.75	45.47	12.37	8.71	9.43	8.14	5.34	8.03	10.21
	11	20.42	20.43	20.41	78.44	50.06	14.96	4.51	7.87	5.29	7.98	6.98	7.81	53.82	54.77	12.64	7.76	10.06	8.85	11.64	12.35	11.80
	12	22.27	22.65	21.90	106.04	43.60	10.91	4.98	4.58	4.96	4.40	5.38	18.96	81.93	43.31	10.03	7.47	7.69	5.55	8.08	7.89	25.19
	13	16.75	14.15	19.34	44.11	33.37	10.08	5.18	6.39	6.53	4.14	13.76	3.81	78.86	30.43	12.27	10.01	8.19	6.26	6.85	11.98	9.18
**Quarter**	1	12.65	13.43	11.87	23.74	40.68	11.42	7.24	7.40	5.81	5.28	6.67	12.66	0.00	46.23	12.68	8.85	8.61	7.60	7.40	7.61	7.89
	2	12.07	12.99	11.14	26.12	39.48	7.96	5.86	6.13	5.81	6.34	5.05	14.16	12.79	32.30	7.82	9.17	7.41	5.56	7.74	7.41	10.11
	3	14.69	14.26	15.11	39.62	36.59	13.00	5.32	6.12	5.71	7.38	7.66	6.93	40.52	38.32	10.05	7.61	8.24	6.20	8.68	8.48	7.92
	4	19.95	19.03	20.87	74.16	44.72	11.49	5.25	6.18	5.67	5.57	8.13	10.06	74.29	43.84	12.40	8.54	8.56	7.19	7.90	9.95	15.18
**Year**	15/16	14.94	15.00	14.87	41.54	40.45	10.98	5.90	6.45	5.75	6.13	6.90	10.94	32.70	40.24	10.77	8.54	8.21	6.65	7.93	8.39	10.37
**Episodes**	F/N	5532.00	2562.00	2970.00	79.00	575.00	375.00	214.00	554.00	435.00	172.00	102.00	56.00	76.00	545.00	344.00	310.00	727.00	484.00	234.00	147.00	103.00

Mean weekly incidence rate per 100,000 Persons.

Infectious Intestinal Disease (ICD10: A00-A09)

			All ages			Male									Female								
			Male & Female	Male	Female	<1yr	1-4yrs	5-14yrs	15-24yrs	25-44yrs	45-64yrs	65-74yrs	75-84yrs	85+yrs	<1yr	1-4yrs	5-14yrs	15-24yrs	25-44yrs	45-64yrs	65-74yrs	75-84yrs	85+yrs
**North**	Quarter	1	16.14	19.83	12.45	94.97	34.35	10.58	6.12	4.63	5.15	4.19	11.60	6.90	0.00	48.42	12.22	7.78	7.24	7.18	11.65	6.46	11.12
		2	12.80	10.63	14.96	0.00	47.27	11.64	3.55	4.30	6.94	7.10	6.43	8.46	51.16	26.19	3.41	8.33	6.55	6.55	9.93	9.18	13.32
		3	18.27	16.81	19.73	53.76	45.00	14.13	5.24	3.88	6.37	6.07	10.45	6.41	71.00	47.59	8.93	6.92	7.94	5.78	9.65	8.02	11.69
		4	26.50	26.23	26.78	116.70	53.32	13.34	5.09	7.94	5.91	9.20	9.23	15.32	114.17	51.09	11.86	7.62	8.60	7.27	10.92	10.61	18.88
	Year	15/16	18.58	18.52	18.64	67.31	45.14	12.44	5.00	5.24	6.09	6.69	9.42	9.39	60.12	43.47	9.16	7.67	7.60	6.71	10.55	8.61	13.85
	Episodes	F/N	1,700.00	796.00	904.00	34.00	175.00	122.00	64.00	126.00	148.00	65.00	47.00	15.00	36.00	159.00	84.00	96.00	174.00	156.00	103.00	53.00	43.00
**South**	Quarter	1	11.32	10.79	11.84	0.00	39.78	11.48	10.18	8.52	5.97	3.90	4.34	12.91	0.00	42.32	11.53	10.55	9.60	6.68	6.83	8.67	10.41
		2	13.48	17.63	9.33	68.57	37.23	4.90	7.49	8.14	6.94	4.34	5.96	15.08	0.00	32.81	5.71	10.48	8.05	7.13	7.55	4.03	8.24
		3	12.75	11.91	13.59	29.54	33.44	6.95	3.99	9.07	4.07	6.58	10.84	2.74	36.18	35.14	8.34	8.22	8.34	5.52	7.98	6.85	5.73
		4	17.43	17.45	17.42	64.87	44.46	7.13	6.06	5.97	4.73	5.46	5.66	12.70	53.79	39.44	7.94	10.80	7.53	8.80	8.90	8.51	11.06
	Year	15/16	13.81	14.50	13.13	41.20	38.84	7.61	6.91	7.89	5.41	5.08	6.68	10.89	23.08	37.47	8.37	10.03	8.36	7.07	7.84	7.04	8.90
	Episodes	F/N	1,274.00	592.00	682.00	15.00	122.00	62.00	65.00	142.00	104.00	38.00	27.00	17.00	11.00	111.00	65.00	89.00	149.00	136.00	62.00	34.00	25.00
**London**	Quarter	1	13.91	13.00	14.82	0.00	40.62	17.14	10.38	9.79	5.19	3.90	6.80	23.19	0.00	61.75	20.23	10.51	9.91	10.88	7.98	8.75	3.39
		2	13.00	13.91	12.09	18.63	48.60	9.26	6.98	7.55	5.80	5.34	4.66	18.39	0.00	43.35	15.84	8.78	8.19	4.38	8.11	9.27	10.89
		3	15.48	13.75	17.20	19.35	35.09	22.06	8.20	6.18	8.23	10.98	8.18	5.51	43.38	37.10	17.31	7.87	10.89	8.48	11.06	11.23	7.51
		4	21.70	21.25	22.15	74.39	57.05	20.46	7.68	7.50	7.50	3.20	13.43	0.00	63.73	53.97	20.59	10.42	12.28	9.16	6.49	10.25	12.44
	Year	15/16	16.13	15.59	16.67	28.97	45.56	17.29	8.29	7.75	6.70	5.81	8.37	11.55	27.47	49.14	18.53	9.41	10.36	8.24	8.37	9.88	8.63
	Episodes	F/N	1,616.00	731.00	885.00	20.00	175.00	138.00	59.00	202.00	94.00	19.00	16.00	8.00	20.00	182.00	141.00	75.00	293.00	108.00	32.00	24.00	10.00
**Midlands And East**	Quarter	1	9.25	10.12	8.37	0.00	47.96	6.48	2.30	6.65	6.94	9.15	3.95	7.65	0.00	32.42	6.73	6.56	7.68	5.66	3.12	6.56	6.62
		2	8.99	9.79	8.19	17.29	24.82	6.05	5.40	4.55	3.55	8.57	3.16	14.70	0.00	26.83	6.35	9.07	6.84	4.17	5.36	7.15	7.96
		3	12.25	14.55	9.94	55.84	32.82	8.85	3.87	5.37	4.16	5.88	1.15	13.06	11.53	33.45	5.62	7.45	5.80	5.00	6.05	7.83	6.74
		4	14.16	11.18	17.14	40.67	24.04	5.03	2.17	3.30	4.56	4.43	4.21	12.23	65.46	30.85	9.23	5.32	5.82	3.54	5.29	10.44	18.35
	Year	15/16	11.22	11.41	11.03	28.68	32.25	6.57	3.41	4.94	4.80	6.96	3.14	11.91	20.12	30.88	7.02	7.07	6.52	4.57	4.96	8.04	10.08
	Episodes	F/N	942.00	443.00	499.00	10.00	103.00	53.00	26.00	84.00	89.00	50.00	12.00	16.00	9.00	93.00	54.00	50.00	111.00	84.00	37.00	36.00	25.00

Mean weekly incidence rate per 100,000 Persons.

Infectious Mononucleosis (ICD10: B27)

		All ages			Male									Female								
		Male & Female	Male	Female	<1yr	1-4yrs	5-14yrs	15-24yrs	25-44yrs	45-64yrs	65-74yrs	75-84yrs	85+yrs	<1yr	1-4yrs	5-14yrs	15-24yrs	25-44yrs	45-64yrs	65-74yrs	75-84yrs	85+yrs
**4 weekly**	1	0.36	0.25	0.46	0.00	0.00	0.75	1.01	0.34	0.15	0.00	0.00	0.00	0.00	0.00	0.76	2.16	0.62	0.22	0.42	0.00	0.00
	2	0.48	0.35	0.62	0.00	0.00	0.83	1.79	0.50	0.00	0.00	0.00	0.00	0.00	0.00	0.78	3.34	0.70	0.34	0.40	0.00	0.00
	3	0.20	0.29	0.12	0.00	0.00	0.39	1.61	0.56	0.00	0.00	0.00	0.00	0.00	0.00	0.00	0.49	0.23	0.38	0.00	0.00	0.00
	4	0.25	0.25	0.25	0.00	0.00	0.00	1.49	0.38	0.35	0.00	0.00	0.00	0.00	0.00	0.00	1.30	0.78	0.18	0.00	0.00	0.00
	5	0.39	0.34	0.44	0.00	0.00	0.42	1.58	1.09	0.00	0.00	0.00	0.00	0.00	0.00	0.42	3.22	0.19	0.14	0.00	0.00	0.00
	6	0.27	0.26	0.27	0.00	0.00	0.46	1.52	0.39	0.00	0.00	0.00	0.00	0.00	0.00	0.00	2.00	0.24	0.18	0.00	0.00	0.00
	7	0.45	0.55	0.34	0.00	0.00	0.44	4.40	0.14	0.00	0.00	0.00	0.00	0.00	0.00	0.00	2.62	0.44	0.00	0.00	0.00	0.00
	8	0.45	0.37	0.54	0.00	0.00	0.29	2.48	0.18	0.35	0.00	0.00	0.00	0.00	0.00	1.21	3.67	0.00	0.00	0.00	0.00	0.00
	9	0.35	0.28	0.42	0.00	0.00	0.00	2.35	0.00	0.17	0.00	0.00	0.00	0.00	0.00	0.47	2.44	0.84	0.00	0.00	0.00	0.00
	10	0.65	0.54	0.77	0.00	0.00	0.88	3.06	0.88	0.00	0.00	0.00	0.00	0.00	0.76	0.00	5.88	0.13	0.17	0.00	0.00	0.00
	11	0.58	0.57	0.59	0.00	0.00	0.82	3.75	0.34	0.20	0.00	0.00	0.00	0.00	0.00	1.69	3.33	0.31	0.00	0.00	0.00	0.00
	12	0.34	0.36	0.32	0.00	0.00	0.68	2.20	0.40	0.00	0.00	0.00	0.00	0.00	0.00	0.00	1.96	0.73	0.19	0.00	0.00	0.00
	13	0.39	0.22	0.57	0.00	0.00	0.00	1.53	0.27	0.15	0.00	0.00	0.00	0.00	0.00	0.94	3.73	0.43	0.00	0.00	0.00	0.00
**Quarter**	1	0.35	0.29	0.41	0.00	0.00	0.66	1.44	0.46	0.06	0.00	0.00	0.00	0.00	0.00	0.53	2.01	0.52	0.31	0.28	0.00	0.00
	2	0.31	0.29	0.32	0.00	0.00	0.27	1.69	0.57	0.11	0.00	0.00	0.00	0.00	0.00	0.13	2.15	0.47	0.15	0.00	0.00	0.00
	3	0.44	0.39	0.48	0.00	0.00	0.36	2.83	0.13	0.16	0.00	0.00	0.00	0.00	0.24	0.51	3.27	0.33	0.00	0.00	0.00	0.00
	4	0.49	0.44	0.54	0.00	0.00	0.55	2.77	0.50	0.10	0.00	0.00	0.00	0.00	0.00	0.75	3.58	0.42	0.10	0.00	0.00	0.00
**Year**	15/16	0.40	0.35	0.44	0.00	0.00	0.46	2.19	0.42	0.11	0.00	0.00	0.00	0.00	0.06	0.49	2.77	0.44	0.14	0.07	0.00	0.00
**Episodes**	F/N	310.00	143.00	167.00	0.00	0.00	15.00	87.00	33.00	8.00	0.00	0.00	0.00	0.00	1.00	15.00	103.00	36.00	10.00	2.00	0.00	0.00

Mean weekly incidence rate per 100,000 Persons.

Infectious Mononucleosis (ICD10: B27)

			All ages			Male								Female							
			Male & Female	Male	Female	<1yr	1-4yrs	5-14yrs	15-24yrs	25-44yrs	45-64yrs	65-74yrs	75-84yrs	85+yrs	<1yr	1-4yrs	5-14yrs	15-24yrs	25-44yrs	45-64yrs	65-74yrs	75-84yrs	85+yrs
**North**	Quarter	1	0.36	0.42	0.29	0.00	0.00	0.72	2.77	0.27	0.00	0.00	0.00	0.00	0.00	0.00	0.00	1.18	0.58	0.24	0.64	0.00	0.00
		2	0.29	0.22	0.37	0.00	0.00	0.00	1.94	0.00	0.00	0.00	0.00	0.00	0.00	0.00	0.00	2.25	0.94	0.17	0.00	0.00	0.00
		3	0.43	0.43	0.43	0.00	0.00	0.35	3.34	0.15	0.00	0.00	0.00	0.00	0.00	0.00	0.00	3.44	0.46	0.00	0.00	0.00	0.00
		4	0.44	0.45	0.43	0.00	0.00	0.34	3.34	0.40	0.00	0.00	0.00	0.00	0.00	0.00	0.37	3.35	0.15	0.00	0.00	0.00	0.00
	Year	15/16	0.38	0.38	0.38	0.00	0.00	0.35	2.86	0.21	0.00	0.00	0.00	0.00	0.00	0.00	0.10	2.57	0.53	0.10	0.16	0.00	0.00
	Episodes	F/N	97.00	45.00	52.00	0.00	0.00	3.00	37.00	5.00	0.00	0.00	0.00	0.00	0.00	0.00	1.00	37.00	11.00	2.00	1.00	0.00	0.00
**South**	Quarter	1	0.43	0.28	0.58	0.00	0.00	0.48	1.41	0.44	0.23	0.00	0.00	0.00	0.00	0.00	1.08	2.98	0.24	0.41	0.50	0.00	0.00
		2	0.33	0.33	0.33	0.00	0.00	0.00	2.08	0.67	0.21	0.00	0.00	0.00	0.00	0.00	0.52	2.25	0.23	0.00	0.00	0.00	0.00
		3	0.55	0.51	0.59	0.00	0.00	0.00	3.92	0.22	0.41	0.00	0.00	0.00	0.00	0.00	1.00	3.82	0.47	0.00	0.00	0.00	0.00
		4	0.55	0.56	0.54	0.00	0.00	0.43	3.83	0.59	0.17	0.00	0.00	0.00	0.00	0.00	0.49	3.57	0.57	0.20	0.00	0.00	0.00
	Year	15/16	0.47	0.42	0.51	0.00	0.00	0.23	2.83	0.48	0.25	0.00	0.00	0.00	0.00	0.00	0.76	3.16	0.38	0.15	0.12	0.00	0.00
	Episodes	F/N	88.00	43.00	45.00	0.00	0.00	2.00	27.00	9.00	5.00	0.00	0.00	0.00	0.00	0.00	6.00	28.00	7.00	3.00	1.00	0.00	0.00
**London**	Quarter	1	0.29	0.23	0.36	0.00	0.00	0.59	0.64	0.87	0.00	0.00	0.00	0.00	0.00	0.00	0.60	1.78	0.48	0.34	0.00	0.00	0.00
		2	0.14	0.08	0.20	0.00	0.00	0.56	0.00	0.17	0.00	0.00	0.00	0.00	0.00	0.00	0.00	1.78	0.00	0.00	0.00	0.00	0.00
		3	0.18	0.08	0.28	0.00	0.00	0.00	0.52	0.17	0.00	0.00	0.00	0.00	0.00	0.94	0.49	0.92	0.13	0.00	0.00	0.00	0.00
		4	0.25	0.28	0.23	0.00	0.00	0.42	1.81	0.25	0.00	0.00	0.00	0.00	0.00	0.00	0.00	1.63	0.48	0.00	0.00	0.00	0.00
	Year	15/16	0.22	0.17	0.27	0.00	0.00	0.39	0.76	0.36	0.00	0.00	0.00	0.00	0.00	0.23	0.27	1.53	0.28	0.08	0.00	0.00	0.00
	Episodes	F/N	42.00	18.00	24.00	0.00	0.00	3.00	6.00	9.00	0.00	0.00	0.00	0.00	0.00	1.00	2.00	12.00	8.00	1.00	0.00	0.00	0.00
**Midlands And East**	Quarter	1	0.31	0.22	0.39	0.00	0.00	0.86	0.92	0.24	0.00	0.00	0.00	0.00	0.00	0.00	0.45	2.08	0.79	0.23	0.00	0.00	0.00
		2	0.47	0.54	0.39	0.00	0.00	0.52	2.72	1.44	0.22	0.00	0.00	0.00	0.00	0.00	0.00	2.32	0.71	0.45	0.00	0.00	0.00
		3	0.59	0.54	0.64	0.00	0.00	1.09	3.51	0.00	0.22	0.00	0.00	0.00	0.00	0.00	0.57	4.90	0.27	0.00	0.00	0.00	0.00
		4	0.71	0.46	0.96	0.00	0.00	1.01	2.12	0.77	0.22	0.00	0.00	0.00	0.00	0.00	2.14	5.77	0.49	0.22	0.00	0.00	0.00
	Year	15/16	0.52	0.44	0.60	0.00	0.00	0.87	2.31	0.62	0.17	0.00	0.00	0.00	0.00	0.00	0.82	3.80	0.56	0.22	0.00	0.00	0.00
	Episodes	F/N	83.00	37.00	46.00	0.00	0.00	7.00	17.00	10.00	3.00	0.00	0.00	0.00	0.00	0.00	6.00	26.00	10.00	4.00	0.00	0.00	0.00

Mean weekly incidence rate per 100,000 Persons.

Influenza-like illness (ICD10 : J09 - J11)

		All ages			Male								Female							
		Male & Female	Male	Female	<1yr	1-4yrs	5-14yrs	15-24yrs	25-44yrs	45-64yrs	65-74yrs	75-84yrs	85+yrs	<1yr	1-4yrs	5-14yrs	15-24yrs	25-44yrs	45-64yrs	65-74yrs	75-84yrs	85+yrs
**4 weekly**	1	2.71	2.58	2.84	0.00	0.00	1.55	3.44	5.24	4.09	4.13	2.95	1.85	0.00	0.00	1.23	6.50	3.99	5.41	2.05	5.29	1.13
	2	1.28	0.79	1.77	0.00	0.00	0.48	0.34	3.46	0.92	1.88	0.00	0.00	0.00	0.00	0.73	2.05	3.15	2.88	3.09	2.86	1.14
	3	1.18	0.68	1.69	0.00	0.00	0.00	0.42	1.46	1.89	2.31	0.00	0.00	0.00	2.62	0.00	2.04	2.48	4.12	3.94	0.00	0.00
	4	1.23	1.14	1.32	0.00	4.09	0.00	0.68	2.09	2.09	0.47	0.79	0.00	0.00	0.00	0.00	2.18	2.73	3.25	3.71	0.00	0.00
	5	2.36	1.70	3.02	0.00	1.04	1.19	2.07	3.50	1.84	3.47	2.21	0.00	0.00	2.85	0.98	3.15	4.67	5.10	5.45	2.76	2.18
	6	5.29	4.39	6.20	0.00	2.88	4.94	3.01	6.26	6.61	6.87	6.49	2.43	0.00	5.26	4.33	7.29	8.26	9.65	6.57	6.72	7.74
	7	5.05	4.99	5.11	0.00	3.44	2.55	7.47	5.43	7.76	5.96	3.47	8.85	0.00	3.68	2.32	7.61	7.81	11.09	7.20	5.36	0.89
	8	7.62	6.11	9.12	0.00	7.95	6.65	9.22	9.18	9.08	8.24	2.61	2.05	10.89	4.20	5.40	11.60	15.33	13.96	8.81	8.66	3.27
	9	9.00	7.52	10.47	0.00	5.87	4.21	10.64	12.55	9.27	6.40	6.35	12.39	5.25	8.48	4.65	6.88	18.47	16.68	14.47	9.37	10.01
	10	15.51	13.26	17.75	7.48	18.98	14.05	16.71	20.44	21.39	5.94	11.69	2.70	21.75	13.55	8.09	20.56	32.19	29.65	15.01	12.30	6.68
	11	14.61	12.46	16.76	0.00	15.01	14.62	21.02	26.24	20.07	12.69	2.50	0.00	0.00	13.15	15.21	26.38	33.74	30.88	14.00	9.03	8.46
	12	17.84	15.25	20.42	4.09	19.13	21.84	18.67	27.87	17.44	15.98	5.36	6.84	4.03	23.74	18.16	27.80	34.96	26.68	19.69	15.73	13.01
	13	7.15	7.13	7.18	6.40	8.37	6.75	11.53	11.19	8.04	6.04	4.08	1.76	0.00	5.25	5.08	10.00	15.73	12.43	8.13	5.84	2.13
**Quarter**	1	1.80	1.44	2.16	0.00	0.00	0.74	1.56	3.53	2.44	2.88	1.14	0.71	0.00	0.81	0.70	3.76	3.27	4.23	2.95	2.91	0.78
	2	3.03	2.47	3.59	0.00	3.06	1.89	1.77	4.20	3.93	3.73	2.92	0.75	0.00	2.82	1.63	4.19	5.24	6.03	5.83	3.26	3.33
	3	8.54	7.55	9.53	0.00	9.54	5.75	10.81	10.72	10.31	6.39	6.46	8.00	4.97	7.12	4.60	10.63	16.87	15.94	10.58	9.55	5.53
	4	13.72	11.82	15.63	5.13	13.08	14.85	17.18	21.79	16.37	11.20	4.30	2.46	7.37	13.67	12.56	21.52	29.16	25.14	14.19	9.76	7.32
**Year**	15/16	6.91	5.93	7.88	1.36	6.55	5.98	8.01	10.28	8.42	6.14	3.72	2.97	3.16	6.25	5.02	10.24	13.93	13.07	8.50	6.43	4.30
**Episodes**	F/N	5737.00	2444.00	3293.00	3.00	100.00	212.00	326.00	913.00	650.00	174.00	51.00	15.00	6.00	90.00	172.00	399.00	1251.00	989.00	243.00	104.00	39.00

Mean weekly incidence rate per 100,000 Persons.

Influenza-like illness (ICD10: J09 - J11)

			All ages			Male									Female								
			Male & Female	Male	Female	<1yr	1-4yrs	5-14yrs	15-24yrs	25-44yrs	45-64yrs	65-74yrs	75-84yrs	85+yrs	<1yr	1-4yrs	5-14yrs	15-24yrs	25-44yrs	45-64yrs	65-74yrs	75-84yrs	85+yrs
**North**	Quarter	1	1.81	1.63	1.99	0.00	0.00	0.64	2.92	3.47	2.06	5.56	0.00	0.00	0.00	1.61	1.30	2.39	2.61	4.01	2.17	2.05	1.73
		2	2.31	1.57	3.05	0.00	0.94	1.48	0.71	2.52	4.12	2.00	2.40	0.00	0.00	3.50	0.37	2.85	2.90	4.06	5.62	3.62	4.52
		3	7.82	7.34	8.29	0.00	13.33	3.42	9.78	11.22	10.20	6.67	7.09	4.38	0.00	7.98	3.79	9.25	18.19	15.62	10.12	6.13	3.55
		4	12.67	12.18	13.16	4.67	15.82	15.86	17.65	19.53	14.65	11.43	5.90	4.15	4.60	7.72	14.31	16.54	24.31	23.99	9.38	7.39	10.19
	Year	15/16	6.28	5.81	6.74	1.23	7.68	5.55	7.95	9.38	7.89	6.51	3.89	2.17	1.22	5.25	5.12	7.92	12.23	12.15	6.87	4.85	5.10
	Episodes	F/N	1,701.00	756.00	945.00	1.00	34.00	61.00	119.00	247.00	206.00	63.00	21.00	4.00	1.00	21.00	53.00	124.00	314.00	310.00	74.00	31.00	17.00
**South**	Quarter	1	1.49	1.19	1.79	0.00	0.00	0.00	0.90	1.94	2.24	2.81	0.00	2.84	0.00	0.00	0.60	5.39	2.76	3.10	2.05	0.83	1.41
		2	3.65	3.01	4.29	0.00	5.05	0.47	3.39	5.36	3.31	3.63	5.89	0.00	0.00	5.47	2.04	5.88	7.79	6.51	5.43	4.00	1.49
		3	8.18	7.27	9.08	0.00	5.11	6.00	13.04	10.43	10.64	6.38	2.99	10.87	0.00	4.09	3.03	12.66	16.02	17.21	13.26	12.59	2.89
		4	16.48	14.59	18.38	8.54	14.44	13.22	23.27	26.60	22.64	16.40	3.66	2.52	0.00	18.12	7.82	28.29	38.89	31.06	19.34	12.15	9.74
	Year	15/16	7.62	6.67	8.57	2.26	6.31	5.08	10.40	11.38	9.95	7.48	3.15	4.03	0.00	7.13	3.45	13.34	16.79	14.79	10.19	7.48	3.99
	Episodes	F/N	1,539.00	648.00	891.00	1.00	21.00	43.00	102.00	211.00	194.00	57.00	13.00	6.00	0.00	22.00	28.00	121.00	305.00	286.00	82.00	36.00	11.00
**London**	Quarter	1	2.80	2.27	3.32	0.00	0.00	2.33	1.94	5.58	3.47	2.60	4.54	0.00	0.00	1.61	0.00	4.18	4.65	5.52	5.11	8.78	0.00
		2	4.41	3.17	5.65	0.00	2.36	5.11	1.91	6.18	5.41	5.27	2.31	0.00	0.00	2.32	4.11	6.31	7.91	9.75	9.54	3.63	7.29
		3	11.30	10.26	12.34	0.00	11.17	7.03	18.10	11.94	13.49	4.91	12.31	13.42	6.46	10.51	9.30	11.90	18.91	16.54	9.28	17.62	10.55
		4	14.42	11.17	17.67	0.00	12.77	17.98	17.63	20.21	15.76	8.57	7.65	0.00	5.33	19.18	15.10	23.16	27.51	25.72	17.64	16.09	9.33
	Year	15/16	8.35	6.80	9.90	0.00	6.69	8.30	10.04	11.15	9.65	5.40	6.72	3.29	2.99	8.61	7.28	11.61	14.99	14.60	10.53	11.61	6.84
	Episodes	F/N	1,593.00	666.00	927.00	0.00	29.00	71.00	79.00	313.00	141.00	18.00	13.00	2.00	2.00	36.00	61.00	100.00	448.00	203.00	40.00	29.00	8.00
**Midlands And East**	Quarter	1	1.11	0.68	1.53	0.00	0.00	0.00	0.46	3.12	1.98	0.54	0.00	0.00	0.00	0.00	0.90	3.07	3.05	4.31	2.48	0.00	0.00
		2	1.75	2.13	1.38	0.00	3.89	0.48	1.08	2.75	2.88	4.01	1.07	2.99	0.00	0.00	0.00	1.72	2.35	3.78	2.75	1.78	0.00
		3	6.87	5.34	8.41	0.00	8.56	6.55	2.33	9.29	6.92	7.59	3.47	3.32	13.40	5.91	2.26	8.70	14.34	14.40	9.68	1.86	5.11
		4	11.32	9.32	13.31	7.31	9.30	12.33	10.17	20.81	12.43	8.39	0.00	3.16	19.53	9.64	13.03	18.07	25.92	19.77	10.42	3.40	0.00
	Year	15/16	5.38	4.46	6.29	1.93	5.51	4.98	3.64	9.21	6.17	5.19	1.11	2.38	8.45	4.00	4.22	8.08	11.69	10.74	6.41	1.79	1.25
	Episodes	F/N	904.00	374.00	530.00	1.00	16.00	37.00	26.00	142.00	109.00	36.00	4.00	3.00	3.00	11.00	30.00	54.00	184.00	190.00	47.00	8.00	3.00

Mean weekly incidence rate per 100,000 Persons.

Lower Respiratory Tract Infections (ICD10: J20-J22)

		All ages			Male								Female							
		Male & Female	Male	Female	<1yr	1-4yrs	5-14yrs	15-24yrs	25-44yrs	45-64yrs	65-74yrs	75-84yrs	85+yrs	<1yr	1-4yrs	5-14yrs	15-24yrs	25-44yrs	45-64yrs	65-74yrs	75-84yrs	85+yrs
**4 weekly**	1	76.03	74.13	77.93	0.00	102.75	18.40	11.77	24.15	42.61	89.40	139.35	238.78	0.00	80.74	14.42	20.56	35.18	63.58	89.38	123.52	273.96
	2	61.15	59.51	62.79	0.00	75.28	18.78	10.30	16.17	28.73	66.81	124.52	195.04	0.00	53.24	12.40	17.55	26.54	46.70	75.40	112.81	220.43
	3	66.00	59.20	72.80	0.00	84.72	16.54	10.91	16.13	36.60	70.56	124.44	172.95	156.25	44.23	12.52	18.37	29.36	41.01	67.11	100.98	185.33
	4	58.92	64.83	53.02	79.03	55.23	10.03	16.87	22.34	33.61	66.11	114.34	185.92	0.00	33.44	9.12	23.16	22.13	42.00	68.43	97.77	181.09
	5	70.17	70.58	69.76	31.50	97.65	19.78	12.46	17.09	37.54	73.67	136.04	209.44	32.94	66.85	14.60	25.71	29.94	54.69	76.90	132.86	193.36
	6	111.46	107.28	115.65	90.71	159.90	27.03	17.77	30.97	56.47	98.06	185.20	299.43	94.69	133.89	19.95	27.22	53.00	95.02	114.95	191.25	310.83
	7	155.10	152.60	157.60	310.86	336.88	35.74	12.64	26.47	57.88	113.77	174.78	304.34	314.79	282.65	25.50	30.22	49.99	80.39	134.54	197.18	303.15
	8	250.08	274.74	225.43	1134.34	449.40	49.48	21.88	35.15	64.69	137.22	213.41	367.10	667.15	388.08	42.55	33.32	62.84	104.61	158.47	212.26	359.54
	9	208.58	221.53	195.63	673.58	180.07	44.82	35.55	44.95	95.60	187.52	299.61	432.05	314.58	172.87	41.17	34.32	68.32	140.25	203.99	309.55	475.61
	10	186.01	192.26	179.75	440.33	254.58	60.47	30.41	50.88	95.37	161.99	247.31	389.00	241.15	199.73	49.95	40.91	89.12	140.80	188.98	238.35	428.80
	11	170.78	171.37	170.18	329.84	196.14	40.79	31.25	45.26	95.76	159.99	237.52	405.78	235.77	179.40	42.95	38.53	84.09	129.03	175.40	249.78	396.72
	12	161.84	164.72	158.96	319.84	179.79	52.61	26.10	48.46	78.52	157.12	222.35	397.73	149.18	148.08	47.08	44.49	76.80	112.70	194.92	248.58	408.81
	13	111.73	111.70	111.76	232.09	120.82	20.63	15.71	28.43	52.05	124.38	135.97	275.19	164.70	99.59	24.33	18.35	48.27	70.00	130.32	198.35	251.93
**Quarter**	1	68.37	65.04	71.69	0.00	88.75	17.95	11.05	19.22	36.49	76.65	130.20	205.06	48.08	61.05	13.22	18.96	30.73	51.44	78.23	113.29	230.22
	2	83.55	83.76	83.34	68.94	117.99	20.11	15.14	23.76	43.92	81.26	147.19	235.51	42.05	90.47	15.75	25.82	35.65	64.16	92.09	145.76	238.27
	3	209.71	221.14	198.28	712.51	319.37	48.94	25.61	37.86	77.04	151.32	239.86	377.76	439.26	275.88	40.03	33.79	65.67	117.48	168.89	244.09	399.39
	4	151.88	154.48	149.29	314.98	174.02	39.14	25.21	42.78	78.39	149.69	202.46	363.67	185.91	146.84	38.84	35.00	72.92	108.33	171.06	231.12	353.56
**Year**	15/16	128.82	131.55	126.09	274.88	175.01	31.68	19.36	31.13	59.33	115.39	180.35	296.79	178.96	143.62	27.18	28.52	51.65	85.79	128.39	184.46	306.27
**Episodes**	F/N	44746.00	19448.00	25298.00	518.00	2456.00	1089.00	746.00	2624.00	4589.00	3177.00	2671.00	1578.00	310.00	1957.00	900.00	1085.00	4371.00	6570.00	3822.00	3303.00	2980.00

Mean weekly incidence rate per 100,000 Persons.

Lower Respiratory Tract Infections (ICD10: J20-J22)

			All ages			Male									Female								
			Male & Female	Male	Female	<1yr	1-4yrs	5-14yrs	15-24yrs	25-44yrs	45-64yrs	65-74yrs	75-84yrs	85+yrs	<1yr	1-4yrs	5-14yrs	15-24yrs	25-44yrs	45-64yrs	65-74yrs	75-84yrs	85+yrs
**North**	Quarter	1	90.03	76.88	103.18	0.00	105.64	15.87	12.13	27.55	44.51	94.83	152.45	238.96	192.31	75.58	12.27	26.01	47.78	70.00	100.17	158.10	246.41
		2	94.57	97.10	92.04	122.19	119.07	15.71	20.31	29.28	52.18	92.23	174.18	248.77	23.46	104.56	15.29	25.77	44.65	77.51	99.41	157.85	279.88
		3	220.10	227.11	213.08	723.95	304.09	44.88	25.29	40.38	91.06	160.77	259.36	394.24	464.74	271.27	37.26	34.37	73.31	143.07	182.81	264.40	446.47
		4	164.38	168.92	159.84	420.83	177.26	38.78	25.55	49.77	82.73	149.21	209.07	367.10	243.58	125.30	39.17	33.97	83.44	119.16	177.15	238.81	377.99
	Year	15/16	142.69	143.00	142.37	318.71	176.53	29.00	20.91	36.99	67.90	124.73	198.96	313.30	231.26	143.82	26.25	30.10	62.69	102.75	140.59	205.43	338.45
	Episodes	F/N	15,915.00	6,809.00	9,106.00	193.00	719.00	298.00	289.00	906.00	1,686.00	1,195.00	1,001.00	522.00	108.00	564.00	261.00	418.00	1,478.00	2,520.00	1,445.00	1,262.00	1,050.00
**South**	Quarter	1	58.18	60.07	56.28	0.00	87.95	19.24	8.16	16.83	33.05	67.51	122.55	185.36	0.00	53.30	12.72	16.34	26.02	43.28	60.89	91.16	202.83
		2	78.02	77.54	78.50	35.94	118.96	19.37	11.74	21.50	33.37	75.06	122.93	259.00	35.71	95.92	15.60	21.05	32.31	46.85	89.54	126.99	242.51
		3	223.40	238.17	208.64	957.09	318.98	51.12	25.41	40.14	73.28	125.18	221.11	331.20	589.45	327.81	38.73	35.35	62.88	100.55	146.69	197.29	378.98
		4	152.42	150.66	154.18	305.85	204.87	42.41	20.72	44.24	68.30	135.37	206.27	327.89	247.85	178.73	37.91	36.31	73.94	102.55	168.51	200.76	341.05
	Year	15/16	128.46	131.97	124.96	324.36	183.11	33.21	16.59	30.94	52.31	101.43	168.93	276.84	218.81	164.22	26.46	27.43	49.26	73.86	117.39	154.93	292.28
	Episodes	F/N	10,416.00	4,579.00	5,837.00	125.00	585.00	277.00	159.00	563.00	999.00	766.00	681.00	424.00	85.00	494.00	208.00	246.00	883.00	1,413.00	938.00	755.00	815.00
**London**	Quarter	1	62.73	59.89	65.58	0.00	86.74	20.11	8.47	12.06	31.58	73.82	99.52	206.71	0.00	64.34	16.76	14.36	18.05	43.66	78.53	110.75	243.74
		2	84.26	86.91	81.61	117.63	117.05	23.74	8.98	19.67	45.71	84.80	156.95	207.70	68.54	89.53	17.18	25.04	25.33	70.07	92.16	168.13	178.55
		3	168.01	166.46	169.55	396.11	271.24	53.94	25.17	26.74	66.59	151.13	260.06	247.14	308.25	214.70	43.87	29.44	47.70	104.38	173.24	246.32	358.07
		4	131.61	127.87	135.35	218.18	114.07	32.55	20.45	29.24	68.53	154.05	191.16	322.60	127.54	135.04	36.55	28.42	52.91	92.46	154.09	246.86	344.29
	Year	15/16	112.03	110.61	113.44	183.65	146.65	32.59	15.85	22.07	53.39	116.67	177.19	247.48	126.11	126.07	28.74	24.40	36.32	77.92	125.07	194.03	282.35
	Episodes	F/N	7,716.00	3,321.00	4,395.00	105.00	578.00	265.00	120.00	605.00	762.00	386.00	330.00	170.00	70.00	497.00	231.00	198.00	1,065.00	1,053.00	476.00	472.00	333.00
**Midlands And East**	Quarter	1	62.52	63.33	61.72	0.00	74.68	16.57	15.46	20.46	36.82	70.43	146.28	189.23	0.00	50.95	11.11	19.13	31.07	48.83	73.30	93.15	227.89
		2	77.33	73.47	81.19	0.00	116.87	21.61	19.51	24.59	44.44	72.94	134.73	226.56	40.48	71.89	14.92	31.41	40.32	62.21	87.25	130.08	252.14
		3	227.33	252.82	201.83	772.88	383.16	45.81	26.57	44.16	77.22	168.22	218.92	538.45	394.60	289.73	40.25	35.99	78.78	121.92	172.83	268.37	414.03
		4	159.13	170.48	147.78	315.04	199.87	42.81	34.11	47.89	94.00	160.15	203.34	437.11	124.69	148.30	41.72	41.28	81.39	119.17	184.49	238.04	350.90
	Year	15/16	132.10	140.60	123.59	272.79	193.76	31.91	24.10	34.53	63.70	118.73	176.34	349.52	139.66	140.37	27.28	32.13	58.33	88.62	130.51	183.46	311.99
	Episodes	F/N	10,699.00	4,739.00	5,960.00	95.00	574.00	249.00	178.00	550.00	1,142.00	830.00	659.00	462.00	47.00	402.00	200.00	223.00	945.00	1,584.00	963.00	814.00	782.00

Mean weekly incidence rate per 100,000 Persons.

Measles (ICD10: B05)

		All ages			Male									Female								
		Male & Female	Male	Female	<1yr	1-4yrs	5-14yrs	15-24yrs	25-44yrs	45-64yrs	65-74yrs	75-84yrs	85+yrs	<1yr	1-4yrs	5-14yrs	15-24yrs	25-44yrs	45-64yrs	65-74yrs	75-84yrs	85+yrs
**4 weekly**	1	0.00	0.00	0.00	0.00	0.00	0.00	0.00	0.00	0.00	0.00	0.00	0.00	0.00	0.00	0.00	0.00	0.00	0.00	0.00	0.00	0.00
	2	0.00	0.00	0.00	0.00	0.00	0.00	0.00	0.00	0.00	0.00	0.00	0.00	0.00	0.00	0.00	0.00	0.00	0.00	0.00	0.00	0.00
	3	0.00	0.00	0.00	0.00	0.00	0.00	0.00	0.00	0.00	0.00	0.00	0.00	0.00	0.00	0.00	0.00	0.00	0.00	0.00	0.00	0.00
	4	0.00	0.00	0.00	0.00	0.00	0.00	0.00	0.00	0.00	0.00	0.00	0.00	0.00	0.00	0.00	0.00	0.00	0.00	0.00	0.00	0.00
	5	0.02	0.04	0.00	0.00	0.00	0.32	0.00	0.00	0.00	0.00	0.00	0.00	0.00	0.00	0.00	0.00	0.00	0.00	0.00	0.00	0.00
	6	0.00	0.00	0.00	0.00	0.00	0.00	0.00	0.00	0.00	0.00	0.00	0.00	0.00	0.00	0.00	0.00	0.00	0.00	0.00	0.00	0.00
	7	0.02	0.04	0.00	0.00	0.00	0.40	0.00	0.00	0.00	0.00	0.00	0.00	0.00	0.00	0.00	0.00	0.00	0.00	0.00	0.00	0.00
	8	0.00	0.00	0.00	0.00	0.00	0.00	0.00	0.00	0.00	0.00	0.00	0.00	0.00	0.00	0.00	0.00	0.00	0.00	0.00	0.00	0.00
	9	0.04	0.08	0.00	0.00	0.75	0.00	0.00	0.00	0.00	0.00	0.00	0.00	0.00	0.00	0.00	0.00	0.00	0.00	0.00	0.00	0.00
	10	0.04	0.08	0.00	0.00	0.75	0.00	0.00	0.00	0.00	0.00	0.00	0.00	0.00	0.00	0.00	0.00	0.00	0.00	0.00	0.00	0.00
	11	0.00	0.00	0.00	0.00	0.00	0.00	0.00	0.00	0.00	0.00	0.00	0.00	0.00	0.00	0.00	0.00	0.00	0.00	0.00	0.00	0.00
	12	0.26	0.01	0.51	0.00	0.00	0.00	0.00	0.11	0.00	0.00	0.00	0.00	3.84	0.76	0.00	0.00	0.00	0.00	0.00	0.00	0.00
	13	0.12	0.15	0.08	0.00	1.38	0.00	0.00	0.00	0.00	0.00	0.00	0.00	0.00	0.00	0.37	0.35	0.00	0.00	0.00	0.00	0.00
**Quarter**	1	0.00	0.00	0.00	0.00	0.00	0.00	0.00	0.00	0.00	0.00	0.00	0.00	0.00	0.00	0.00	0.00	0.00	0.00	0.00	0.00	0.00
	2	0.01	0.01	0.00	0.00	0.00	0.10	0.00	0.00	0.00	0.00	0.00	0.00	0.00	0.00	0.00	0.00	0.00	0.00	0.00	0.00	0.00
	3	0.03	0.07	0.00	0.00	0.46	0.12	0.00	0.00	0.00	0.00	0.00	0.00	0.00	0.00	0.00	0.00	0.00	0.00	0.00	0.00	0.00
	4	0.11	0.05	0.17	0.00	0.40	0.00	0.00	0.03	0.00	0.00	0.00	0.00	1.10	0.22	0.10	0.10	0.00	0.00	0.00	0.00	0.00
**Year**	15/16	0.04	0.03	0.04	0.00	0.22	0.05	0.00	0.01	0.00	0.00	0.00	0.00	0.29	0.06	0.03	0.03	0.00	0.00	0.00	0.00	0.00
**Episodes**	F/N	11.00	7.00	4.00	0.00	4.00	2.00	0.00	1.00	0.00	0.00	0.00	0.00	1.00	1.00	1.00	1.00	0.00	0.00	0.00	0.00	0.00

Mean weekly incidence rate per 100,000 Persons.

Measles (ICD10: B05)

			All ages			Male									Female								
			Male & Female	Male	Female	<1yr	1-4yrs	5-14yrs	15-24yrs	25-44yrs	45-64yrs	65-74yrs	75-84yrs	85+yrs	<1yr	1-4yrs	5-14yrs	15-24yrs	25-44yrs	45-64yrs	65-74yrs	75-84yrs	85+yrs
**North**	Quarter	1	0.00	0.00	0.00	0.00	0.00	0.00	0.00	0.00	0.00	0.00	0.00	0.00	0.00	0.00	0.00	0.00	0.00	0.00	0.00	0.00	0.00
		2	0.02	0.04	0.00	0.00	0.00	0.39	0.00	0.00	0.00	0.00	0.00	0.00	0.00	0.00	0.00	0.00	0.00	0.00	0.00	0.00	0.00
		3	0.00	0.00	0.00	0.00	0.00	0.00	0.00	0.00	0.00	0.00	0.00	0.00	0.00	0.00	0.00	0.00	0.00	0.00	0.00	0.00	0.00
		4	0.09	0.08	0.10	0.00	0.76	0.00	0.00	0.00	0.00	0.00	0.00	0.00	0.00	0.86	0.00	0.00	0.00	0.00	0.00	0.00	0.00
	Year	15/16	0.03	0.03	0.03	0.00	0.20	0.10	0.00	0.00	0.00	0.00	0.00	0.00	0.00	0.23	0.00	0.00	0.00	0.00	0.00	0.00	0.00
	Episodes	F/N	3.00	2.00	1.00	0.00	1.00	1.00	0.00	0.00	0.00	0.00	0.00	0.00	0.00	1.00	0.00	0.00	0.00	0.00	0.00	0.00	0.00
**South**	Quarter	1	0.00	0.00	0.00	0.00	0.00	0.00	0.00	0.00	0.00	0.00	0.00	0.00	0.00	0.00	0.00	0.00	0.00	0.00	0.00	0.00	0.00
		2	0.00	0.00	0.00	0.00	0.00	0.00	0.00	0.00	0.00	0.00	0.00	0.00	0.00	0.00	0.00	0.00	0.00	0.00	0.00	0.00	0.00
		3	0.00	0.00	0.00	0.00	0.00	0.00	0.00	0.00	0.00	0.00	0.00	0.00	0.00	0.00	0.00	0.00	0.00	0.00	0.00	0.00	0.00
		4	0.00	0.00	0.00	0.00	0.00	0.00	0.00	0.00	0.00	0.00	0.00	0.00	0.00	0.00	0.00	0.00	0.00	0.00	0.00	0.00	0.00
	Year	15/16	0.00	0.00	0.00	0.00	0.00	0.00	0.00	0.00	0.00	0.00	0.00	0.00	0.00	0.00	0.00	0.00	0.00	0.00	0.00	0.00	0.00
	Episodes	F/N	0.00	0.00	0.00	0.00	0.00	0.00	0.00	0.00	0.00	0.00	0.00	0.00	0.00	0.00	0.00	0.00	0.00	0.00	0.00	0.00	0.00
**London**	Quarter	1	0.00	0.00	0.00	0.00	0.00	0.00	0.00	0.00	0.00	0.00	0.00	0.00	0.00	0.00	0.00	0.00	0.00	0.00	0.00	0.00	0.00
		2	0.00	0.00	0.00	0.00	0.00	0.00	0.00	0.00	0.00	0.00	0.00	0.00	0.00	0.00	0.00	0.00	0.00	0.00	0.00	0.00	0.00
		3	0.10	0.21	0.00	0.00	1.84	0.00	0.00	0.00	0.00	0.00	0.00	0.00	0.00	0.00	0.00	0.00	0.00	0.00	0.00	0.00	0.00
		4	0.34	0.11	0.58	0.00	0.82	0.00	0.00	0.13	0.00	0.00	0.00	0.00	4.39	0.00	0.42	0.40	0.00	0.00	0.00	0.00	0.00
	Year	15/16	0.12	0.08	0.15	0.00	0.67	0.00	0.00	0.03	0.00	0.00	0.00	0.00	1.16	0.00	0.11	0.11	0.00	0.00	0.00	0.00	0.00
	Episodes	F/N	7.00	4.00	3.00	0.00	3.00	0.00	0.00	1.00	0.00	0.00	0.00	0.00	1.00	0.00	1.00	1.00	0.00	0.00	0.00	0.00	0.00
**Midlands And East**	Quarter	1	0.00	0.00	0.00	0.00	0.00	0.00	0.00	0.00	0.00	0.00	0.00	0.00	0.00	0.00	0.00	0.00	0.00	0.00	0.00	0.00	0.00
		2	0.00	0.00	0.00	0.00	0.00	0.00	0.00	0.00	0.00	0.00	0.00	0.00	0.00	0.00	0.00	0.00	0.00	0.00	0.00	0.00	0.00
		3	0.03	0.06	0.00	0.00	0.00	0.50	0.00	0.00	0.00	0.00	0.00	0.00	0.00	0.00	0.00	0.00	0.00	0.00	0.00	0.00	0.00
		4	0.00	0.00	0.00	0.00	0.00	0.00	0.00	0.00	0.00	0.00	0.00	0.00	0.00	0.00	0.00	0.00	0.00	0.00	0.00	0.00	0.00
	Year	15/16	0.01	0.01	0.00	0.00	0.00	0.12	0.00	0.00	0.00	0.00	0.00	0.00	0.00	0.00	0.00	0.00	0.00	0.00	0.00	0.00	0.00
	Episodes	F/N	1.00	1.00	0.00	0.00	0.00	1.00	0.00	0.00	0.00	0.00	0.00	0.00	0.00	0.00	0.00	0.00	0.00	0.00	0.00	0.00	0.00

Mean weekly incidence rate per 100,000 Persons.

Meningitis and Encephalitis (ICD10: A170 - A171; A 390; A83 - A85; A87; G00 - G05)

		All ages			Male									Female								
		Male & Female	Male	Female	<1yr	1-4yrs	5-14yrs	15-24yrs	25-44yrs	45-64yrs	65-74yrs	75-84yrs	85+yrs	<1yr	1-4yrs	5-14yrs	15-24yrs	25-44yrs	45-64yrs	65-74yrs	75-84yrs	85+yrs
**4 weekly**	1	0.37	0.31	0.43	0.00	0.82	0.00	0.00	0.15	0.37	0.00	1.44	0.00	0.00	2.64	0.00	0.00	0.57	0.00	0.00	0.64	0.00
	2	0.06	0.02	0.09	0.00	0.00	0.00	0.00	0.19	0.00	0.00	0.00	0.00	0.00	0.00	0.00	0.36	0.31	0.17	0.00	0.00	0.00
	3	0.12	0.05	0.20	0.00	0.00	0.00	0.42	0.00	0.00	0.00	0.00	0.00	0.00	0.00	0.00	1.42	0.00	0.35	0.00	0.00	0.00
	4	0.07	0.06	0.08	0.00	0.00	0.00	0.00	0.20	0.36	0.00	0.00	0.00	0.00	0.00	0.00	0.36	0.32	0.00	0.00	0.00	0.00
	5	0.06	0.09	0.03	0.00	0.00	0.00	0.00	0.00	0.00	0.00	0.81	0.00	0.00	0.00	0.00	0.00	0.31	0.00	0.00	0.00	0.00
	6	0.18	0.14	0.23	0.00	0.00	0.00	0.00	0.00	0.19	1.06	0.00	0.00	0.00	0.00	0.00	0.00	0.57	0.17	0.00	0.00	1.30
	7	1.87	0.87	2.87	7.79	0.00	0.00	0.00	0.00	0.00	0.00	0.00	0.00	24.94	0.00	0.00	0.49	0.41	0.00	0.00	0.00	0.00
	8	0.51	0.83	0.19	7.21	0.00	0.00	0.00	0.23	0.00	0.00	0.00	0.00	0.00	0.00	0.40	0.00	0.62	0.71	0.00	0.00	0.00
	9	0.10	0.07	0.13	0.00	0.00	0.00	0.35	0.24	0.00	0.00	0.00	0.00	0.00	0.00	0.00	0.24	0.58	0.00	0.32	0.00	0.00
	10	0.18	0.14	0.23	0.00	0.00	0.00	0.21	0.11	0.00	0.94	0.00	0.00	0.00	0.76	0.00	0.37	0.42	0.00	0.00	0.51	0.00
	11	0.27	0.24	0.31	0.00	1.94	0.00	0.00	0.00	0.17	0.00	0.00	0.00	0.00	0.00	0.38	0.49	0.35	0.17	1.43	0.00	0.00
	12	0.26	0.49	0.04	4.09	0.00	0.00	0.21	0.12	0.00	0.00	0.00	0.00	0.00	0.00	0.00	0.36	0.00	0.00	0.00	0.00	0.00
	13	0.41	0.06	0.77	0.00	0.00	0.00	0.00	0.37	0.15	0.00	0.00	0.00	6.79	0.00	0.00	0.00	0.15	0.00	0.00	0.00	0.00
**Quarter**	1	0.20	0.14	0.25	0.00	0.31	0.00	0.13	0.12	0.14	0.00	0.55	0.00	0.00	1.02	0.00	0.55	0.32	0.16	0.00	0.25	0.00
	2	0.31	0.09	0.54	0.00	0.00	0.00	0.00	0.06	0.17	0.33	0.25	0.00	3.93	0.00	0.00	0.11	0.37	0.05	0.00	0.00	0.40
	3	0.55	0.54	0.56	4.62	0.00	0.00	0.11	0.18	0.00	0.00	0.00	0.00	3.74	0.00	0.12	0.34	0.55	0.22	0.10	0.00	0.00
	4	0.31	0.26	0.37	1.17	0.55	0.00	0.12	0.14	0.09	0.27	0.00	0.00	1.94	0.22	0.11	0.24	0.21	0.05	0.41	0.15	0.00
**Year**	15/16	0.34	0.26	0.43	1.44	0.22	0.00	0.09	0.12	0.10	0.15	0.20	0.00	2.39	0.31	0.06	0.31	0.36	0.12	0.13	0.10	0.10
**Episodes**	F/N	98.00	34.00	64.00	3.00	3.00	0.00	4.00	12.00	7.00	2.00	3.00	0.00	4.00	4.00	2.00	10.00	28.00	9.00	4.00	2.00	1.00

Mean weekly incidence rate per 100,000 Persons.

Meningitis and Encephalitis (ICD10: A170 - A171; A 390; A83 - A85; A87; G00 - G05)

			All ages			Male								Female							
			Male & Female	Male	Female	<1yr	1-4yrs	5-14yrs	15-24yrs	25-44yrs	45-64yrs	65-74yrs	75-84yrs	85+yrs	<1yr	1-4yrs	5-14yrs	15-24yrs	25-44yrs	45-64yrs	65-74yrs	75-84yrs	85+yrs
**North**	Quarter	1	0.28	0.06	0.51	0.00	0.00	0.00	0.52	0.00	0.00	0.00	0.00	0.00	0.00	1.86	0.00	1.15	0.60	0.00	0.00	0.98	0.00
		2	0.05	0.08	0.02	0.00	0.00	0.00	0.00	0.25	0.45	0.00	0.00	0.00	0.00	0.00	0.00	0.00	0.15	0.00	0.00	0.00	0.00
		3	0.64	1.13	0.14	9.59	0.00	0.00	0.00	0.58	0.00	0.00	0.00	0.00	0.00	0.00	0.00	0.29	0.45	0.15	0.39	0.00	0.00
		4	0.65	0.69	0.61	4.67	0.89	0.00	0.48	0.13	0.00	0.00	0.00	0.00	3.89	0.00	0.00	0.00	0.30	0.00	0.71	0.58	0.00
	Year	15/16	0.41	0.49	0.32	3.59	0.23	0.00	0.25	0.24	0.11	0.00	0.00	0.00	1.03	0.46	0.00	0.35	0.37	0.04	0.28	0.39	0.00
	Episodes	F/N	33.00	14.00	19.00	2.00	1.00	0.00	3.00	6.00	2.00	0.00	0.00	0.00	1.00	1.00	0.00	3.00	8.00	1.00	3.00	2.00	0.00
**South**	Quarter	1	0.24	0.32	0.15	0.00	0.00	0.00	0.00	0.46	0.24	0.00	2.21	0.00	0.00	0.00	0.00	0.45	0.50	0.41	0.00	0.00	0.00
		2	0.98	0.11	1.85	0.00	0.00	0.00	0.00	0.00	0.00	0.00	1.00	0.00	15.73	0.00	0.00	0.45	0.23	0.21	0.00	0.00	0.00
		3	0.96	0.05	1.87	0.00	0.00	0.00	0.43	0.00	0.00	0.00	0.00	0.00	14.97	0.00	0.00	0.45	1.22	0.20	0.00	0.00	0.00
		4	0.07	0.06	0.09	0.00	0.00	0.00	0.00	0.18	0.37	0.00	0.00	0.00	0.00	0.00	0.00	0.41	0.17	0.20	0.00	0.00	0.00
	Year	15/16	0.55	0.13	0.97	0.00	0.00	0.00	0.11	0.16	0.16	0.00	0.79	0.00	7.53	0.00	0.00	0.44	0.52	0.26	0.00	0.00	0.00
	Episodes	F/N	30.00	10.00	20.00	0.00	0.00	0.00	1.00	3.00	3.00	0.00	3.00	0.00	2.00	0.00	0.00	4.00	9.00	5.00	0.00	0.00	0.00
**London**	Quarter	1	0.10	0.18	0.02	0.00	1.25	0.00	0.00	0.00	0.32	0.00	0.00	0.00	0.00	0.00	0.00	0.00	0.16	0.00	0.00	0.00	0.00
		2	0.11	0.14	0.07	0.00	0.00	0.00	0.00	0.00	0.00	1.30	0.00	0.00	0.00	0.00	0.00	0.00	0.63	0.00	0.00	0.00	0.00
		3	0.54	1.00	0.08	8.87	0.00	0.00	0.00	0.14	0.00	0.00	0.00	0.00	0.00	0.00	0.49	0.00	0.00	0.27	0.00	0.00	0.00
		4	0.42	0.15	0.69	0.00	0.00	0.00	0.00	0.25	0.00	1.07	0.00	0.00	3.87	0.87	0.43	0.00	0.12	0.00	0.93	0.00	0.00
	Year	15/16	0.29	0.36	0.22	2.18	0.31	0.00	0.00	0.10	0.08	0.60	0.00	0.00	1.02	0.23	0.23	0.00	0.22	0.07	0.25	0.00	0.00
	Episodes	F/N	20.00	8.00	12.00	1.00	1.00	0.00	0.00	3.00	1.00	2.00	0.00	0.00	1.00	1.00	2.00	0.00	6.00	1.00	1.00	0.00	0.00
**Midlands And East**	Quarter	1	0.17	0.00	0.34	0.00	0.00	0.00	0.00	0.00	0.00	0.00	0.00	0.00	0.00	2.20	0.00	0.60	0.00	0.23	0.00	0.00	0.00
		2	0.13	0.03	0.23	0.00	0.00	0.00	0.00	0.00	0.23	0.00	0.00	0.00	0.00	0.00	0.00	0.00	0.46	0.00	0.00	0.00	1.60
		3	0.08	0.00	0.15	0.00	0.00	0.00	0.00	0.00	0.00	0.00	0.00	0.00	0.00	0.00	0.00	0.61	0.53	0.24	0.00	0.00	0.00
		4	0.12	0.15	0.09	0.00	1.33	0.00	0.00	0.00	0.00	0.00	0.00	0.00	0.00	0.00	0.00	0.56	0.25	0.00	0.00	0.00	0.00
	Year	15/16	0.12	0.05	0.20	0.00	0.35	0.00	0.00	0.00	0.06	0.00	0.00	0.00	0.00	0.54	0.00	0.44	0.31	0.11	0.00	0.00	0.39
	Episodes	F/N	15.00	2.00	13.00	0.00	1.00	0.00	0.00	0.00	1.00	0.00	0.00	0.00	0.00	2.00	0.00	3.00	5.00	2.00	0.00	0.00	1.00

Mean weekly incidence rate per 100,000 Persons.

Mumps (ICD10: B26)

		All ages			Male									Female								
		Male & Female	Male	Female	<1yr	1-4yrs	5-14yrs	15-24yrs	25-44yrs	45-64yrs	65-74yrs	75-84yrs	85+yrs	<1yr	1-4yrs	5-14yrs	15-24yrs	25-44yrs	45-64yrs	65-74yrs	75-84yrs	85+yrs
**4 weekly**	1	0.09	0.12	0.07	0.00	0.00	0.28	0.30	0.46	0.00	0.00	0.00	0.00	0.00	0.00	0.00	0.44	0.17	0.00	0.00	0.00	0.00
	2	0.06	0.11	0.01	0.00	0.00	0.35	0.46	0.14	0.00	0.00	0.00	0.00	0.00	0.00	0.00	0.00	0.13	0.00	0.00	0.00	0.00
	3	0.07	0.06	0.07	0.00	0.00	0.00	0.00	0.56	0.00	0.00	0.00	0.00	0.00	0.00	0.00	0.45	0.21	0.00	0.00	0.00	0.00
	4	0.10	0.02	0.18	0.00	0.00	0.00	0.00	0.14	0.00	0.00	0.00	0.00	0.00	0.00	0.42	0.00	0.50	0.00	0.00	0.67	0.00
	5	0.04	0.09	0.00	0.00	0.00	0.48	0.00	0.16	0.17	0.00	0.00	0.00	0.00	0.00	0.00	0.00	0.00	0.00	0.00	0.00	0.00
	6	0.16	0.13	0.19	0.00	0.00	0.39	0.00	0.44	0.30	0.00	0.00	0.00	0.00	0.00	0.00	1.47	0.12	0.12	0.00	0.00	0.00
	7	0.17	0.12	0.23	0.00	0.00	0.75	0.00	0.29	0.00	0.00	0.00	0.00	0.00	1.56	0.00	0.35	0.13	0.00	0.00	0.00	0.00
	8	0.14	0.19	0.10	0.00	0.00	0.56	0.53	0.63	0.00	0.00	0.00	0.00	0.00	0.00	0.69	0.00	0.18	0.00	0.00	0.00	0.00
	9	0.18	0.21	0.15	0.00	0.75	0.68	0.21	0.24	0.00	0.00	0.00	0.00	0.00	0.00	0.44	0.56	0.11	0.22	0.00	0.00	0.00
	10	0.13	0.19	0.08	0.00	1.19	0.37	0.00	0.00	0.13	0.00	0.00	0.00	0.00	0.00	0.00	0.37	0.19	0.19	0.00	0.00	0.00
	11	0.10	0.07	0.12	0.00	0.00	0.45	0.21	0.00	0.00	0.00	0.00	0.00	0.00	0.00	0.00	0.72	0.11	0.22	0.00	0.00	0.00
	12	0.14	0.12	0.16	0.00	0.75	0.00	0.00	0.21	0.12	0.00	0.00	0.00	0.00	0.77	0.00	0.00	0.00	0.00	0.68	0.00	0.00
	13	0.29	0.14	0.44	0.00	0.65	0.37	0.00	0.00	0.00	0.29	0.00	0.00	3.32	0.68	0.00	0.00	0.00	0.00	0.00	0.00	0.00
**Quarter**	1	0.07	0.10	0.05	0.00	0.00	0.22	0.25	0.39	0.00	0.00	0.00	0.00	0.00	0.00	0.00	0.31	0.17	0.00	0.00	0.00	0.00
	2	0.11	0.08	0.13	0.00	0.00	0.36	0.00	0.26	0.14	0.00	0.00	0.00	0.00	0.00	0.13	0.56	0.23	0.04	0.00	0.20	0.00
	3	0.17	0.20	0.14	0.00	0.60	0.64	0.23	0.32	0.04	0.00	0.00	0.00	0.00	0.48	0.35	0.29	0.09	0.07	0.00	0.00	0.00
	4	0.16	0.10	0.22	0.00	0.40	0.23	0.06	0.06	0.03	0.08	0.00	0.00	0.95	0.41	0.00	0.21	0.08	0.12	0.20	0.00	0.00
**Year**	15/16	0.13	0.12	0.14	0.00	0.25	0.36	0.13	0.26	0.05	0.02	0.00	0.00	0.25	0.23	0.12	0.34	0.14	0.06	0.05	0.05	0.00
**Episodes**	F/N	90.00	51.00	39.00	0.00	4.00	13.00	6.00	22.00	5.00	1.00	0.00	0.00	1.00	4.00	4.00	11.00	12.00	4.00	2.00	1.00	0.00

Mean weekly incidence rate per 100,000 Persons.

Mumps (ICD10: B26)

			All ages			Male									Female								
			Male & Female	Male	Female	<1yr	1-4yrs	5-14yrs	15-24yrs	25-44yrs	45-64yrs	65-74yrs	75-84yrs	85+yrs	<1yr	1-4yrs	5-14yrs	15-24yrs	25-44yrs	45-64yrs	65-74yrs	75-84yrs	85+yrs
**North**	Quarter	1	0.28	0.06	0.51	0.00	0.00	0.00	0.52	0.00	0.00	0.00	0.00	0.00	0.00	1.86	0.00	1.15	0.60	0.00	0.00	0.98	0.00
		2	0.05	0.08	0.02	0.00	0.00	0.00	0.00	0.25	0.45	0.00	0.00	0.00	0.00	0.00	0.00	0.00	0.15	0.00	0.00	0.00	0.00
		3	0.64	1.13	0.14	9.59	0.00	0.00	0.00	0.58	0.00	0.00	0.00	0.00	0.00	0.00	0.00	0.29	0.45	0.15	0.39	0.00	0.00
		4	0.65	0.69	0.61	4.67	0.89	0.00	0.48	0.13	0.00	0.00	0.00	0.00	3.89	0.00	0.00	0.00	0.30	0.00	0.71	0.58	0.00
	Year	15/16	0.41	0.49	0.32	3.59	0.23	0.00	0.25	0.24	0.11	0.00	0.00	0.00	1.03	0.46	0.00	0.35	0.37	0.04	0.28	0.39	0.00
	Episodes	F/N	33.00	14.00	19.00	2.00	1.00	0.00	3.00	6.00	2.00	0.00	0.00	0.00	1.00	1.00	0.00	3.00	8.00	1.00	3.00	2.00	0.00
**South**	Quarter	1	0.24	0.32	0.15	0.00	0.00	0.00	0.00	0.46	0.24	0.00	2.21	0.00	0.00	0.00	0.00	0.45	0.50	0.41	0.00	0.00	0.00
		2	0.98	0.11	1.85	0.00	0.00	0.00	0.00	0.00	0.00	0.00	1.00	0.00	15.73	0.00	0.00	0.45	0.23	0.21	0.00	0.00	0.00
		3	0.96	0.05	1.87	0.00	0.00	0.00	0.43	0.00	0.00	0.00	0.00	0.00	14.97	0.00	0.00	0.45	1.22	0.20	0.00	0.00	0.00
		4	0.07	0.06	0.09	0.00	0.00	0.00	0.00	0.18	0.37	0.00	0.00	0.00	0.00	0.00	0.00	0.41	0.17	0.20	0.00	0.00	0.00
	Year	15/16	0.55	0.13	0.97	0.00	0.00	0.00	0.11	0.16	0.16	0.00	0.79	0.00	7.53	0.00	0.00	0.44	0.52	0.26	0.00	0.00	0.00
	Episodes	F/N	30.00	10.00	20.00	0.00	0.00	0.00	1.00	3.00	3.00	0.00	3.00	0.00	2.00	0.00	0.00	4.00	9.00	5.00	0.00	0.00	0.00
**London**	Quarter	1	0.10	0.18	0.02	0.00	1.25	0.00	0.00	0.00	0.32	0.00	0.00	0.00	0.00	0.00	0.00	0.00	0.16	0.00	0.00	0.00	0.00
		2	0.11	0.14	0.07	0.00	0.00	0.00	0.00	0.00	0.00	1.30	0.00	0.00	0.00	0.00	0.00	0.00	0.63	0.00	0.00	0.00	0.00
		3	0.54	1.00	0.08	8.87	0.00	0.00	0.00	0.14	0.00	0.00	0.00	0.00	0.00	0.00	0.49	0.00	0.00	0.27	0.00	0.00	0.00
		4	0.42	0.15	0.69	0.00	0.00	0.00	0.00	0.25	0.00	1.07	0.00	0.00	3.87	0.87	0.43	0.00	0.12	0.00	0.93	0.00	0.00
	Year	15/16	0.29	0.36	0.22	2.18	0.31	0.00	0.00	0.10	0.08	0.60	0.00	0.00	1.02	0.23	0.23	0.00	0.22	0.07	0.25	0.00	0.00
	Episodes	F/N	20.00	8.00	12.00	1.00	1.00	0.00	0.00	3.00	1.00	2.00	0.00	0.00	1.00	1.00	2.00	0.00	6.00	1.00	1.00	0.00	0.00
**Midlands And East**	Quarter	1	0.17	0.00	0.34	0.00	0.00	0.00	0.00	0.00	0.00	0.00	0.00	0.00	0.00	2.20	0.00	0.60	0.00	0.23	0.00	0.00	0.00
		2	0.13	0.03	0.23	0.00	0.00	0.00	0.00	0.00	0.23	0.00	0.00	0.00	0.00	0.00	0.00	0.00	0.46	0.00	0.00	0.00	1.60
		3	0.08	0.00	0.15	0.00	0.00	0.00	0.00	0.00	0.00	0.00	0.00	0.00	0.00	0.00	0.00	0.61	0.53	0.24	0.00	0.00	0.00
		4	0.12	0.15	0.09	0.00	1.33	0.00	0.00	0.00	0.00	0.00	0.00	0.00	0.00	0.00	0.00	0.56	0.25	0.00	0.00	0.00	0.00
	Year	15/16	0.12	0.05	0.20	0.00	0.35	0.00	0.00	0.00	0.06	0.00	0.00	0.00	0.00	0.54	0.00	0.44	0.31	0.11	0.00	0.00	0.39
	Episodes	F/N	15.00	2.00	13.00	0.00	1.00	0.00	0.00	0.00	1.00	0.00	0.00	0.00	0.00	2.00	0.00	3.00	5.00	2.00	0.00	0.00	1.00

Mean weekly incidence rate per 100,000 Persons.

Non-infective Enteritis and Colitis (ICD10: K50-K52)

		All ages			Male									Female								
		Male & Female	Male	Female	<1yr	1-4yrs	5-14yrs	15-24yrs	25-44yrs	45-64yrs	65-74yrs	75-84yrs	85+yrs	<1yr	1-4yrs	5-14yrs	15-24yrs	25-44yrs	45-64yrs	65-74yrs	75-84yrs	85+yrs
**4 weekly**	1	11.84	10.62	13.07	0.00	43.73	9.90	10.61	7.80	6.51	4.62	12.37	0.00	0.00	43.42	14.61	12.13	9.86	6.29	9.69	10.40	11.20
	2	11.99	11.23	12.75	0.00	34.27	13.86	12.88	8.92	5.54	7.48	11.49	6.60	0.00	40.30	10.27	9.85	9.88	5.47	9.84	8.25	20.89
	3	16.21	15.61	16.81	60.68	30.13	8.90	8.88	10.82	5.27	5.10	4.16	6.51	62.50	20.55	6.53	10.06	13.48	7.74	9.81	10.79	9.78
	4	12.26	12.96	11.55	42.52	21.67	4.67	8.97	6.66	5.05	7.01	6.68	13.41	25.83	22.59	3.75	8.01	9.47	7.58	5.45	7.67	13.64
	5	11.42	11.11	11.74	30.68	30.05	7.89	8.14	6.72	6.71	5.28	4.53	0.00	16.67	27.94	9.18	9.01	9.58	6.36	9.44	6.99	10.47
	6	16.02	13.74	18.30	36.36	34.48	10.99	7.29	7.65	5.93	3.14	8.75	9.08	61.80	32.90	9.04	7.51	11.22	7.79	7.23	10.46	16.71
	7	17.84	15.09	20.59	58.80	34.93	10.23	6.32	5.86	4.93	7.28	3.09	4.33	79.17	34.91	8.38	9.51	8.05	9.54	6.52	10.90	18.38
	8	20.80	21.19	20.41	85.87	37.41	11.67	7.15	7.01	8.79	9.55	7.54	15.75	79.10	43.16	8.54	9.62	10.74	5.83	7.10	8.19	11.42
	9	12.68	13.34	12.02	36.25	28.36	7.02	4.41	5.53	5.57	6.15	7.33	19.41	40.48	12.13	9.45	9.35	7.23	7.63	7.75	4.91	9.26
	10	15.32	15.63	15.01	58.30	30.37	11.09	7.11	5.64	5.76	3.11	14.65	4.60	34.79	32.25	9.74	11.66	8.69	7.40	8.25	12.24	10.03
	11	17.04	16.73	17.35	76.82	28.67	12.15	7.56	5.48	5.44	4.68	5.83	3.92	61.93	29.14	10.15	9.95	8.45	8.11	7.25	9.54	11.63
	12	15.25	13.97	16.54	49.84	30.19	8.99	6.17	5.80	5.47	8.89	6.69	3.68	55.71	31.79	11.27	8.89	8.86	6.88	5.39	9.20	10.86
	13	16.84	15.68	18.00	35.82	37.55	12.44	8.64	7.26	5.86	6.15	13.99	13.37	61.80	28.29	11.10	12.24	8.82	5.79	6.86	14.44	12.69
**Quarter**	1	13.23	12.34	14.12	18.67	36.63	10.81	10.78	9.07	5.83	5.65	9.58	4.03	19.23	35.42	10.79	10.79	10.98	6.48	9.77	9.86	13.75
	2	13.03	12.14	13.91	33.71	29.01	7.61	8.03	6.82	5.79	5.28	6.14	6.92	35.30	27.88	7.00	8.18	10.04	7.19	7.01	8.73	13.83
	3	17.06	16.73	17.39	60.01	33.96	10.21	6.18	6.08	6.29	6.98	8.67	12.15	64.54	29.00	9.31	9.46	8.68	7.35	7.81	8.08	12.26
	4	16.49	15.89	17.09	59.04	31.16	11.20	7.31	6.20	5.79	6.12	8.84	7.31	55.09	31.49	10.74	10.97	8.65	7.33	6.59	11.40	11.52
**Year**	15/16	14.98	14.30	15.65	43.16	32.66	9.98	8.06	7.03	5.92	6.01	8.32	7.60	43.76	30.96	9.48	9.87	9.57	7.09	7.77	9.55	12.81
**Episodes**	F/N	5516.00	2508.00	3008.00	71.00	467.00	326.00	284.00	596.00	439.00	162.00	122.00	41.00	75.00	422.00	300.00	366.00	823.00	520.00	218.00	170.00	114.00

Mean weekly incidence rate per 100,000 Persons.

Non-infective Enteritis and Colitis (ICD10: K50-K52)

			All ages			Male								Female							
			Male & Female	Male	Female	<1yr	1-4yrs	5-14yrs	15-24yrs	25-44yrs	45-64yrs	65-74yrs	75-84yrs	85+yrs	<1yr	1-4yrs	5-14yrs	15-24yrs	25-44yrs	45-64yrs	65-74yrs	75-84yrs	85+yrs
**North**	Quarter	1	9.86	8.97	10.74	0.00	25.18	7.43	12.70	9.69	5.48	7.97	9.01	3.26	0.00	30.28	10.31	8.97	10.15	6.14	7.42	5.59	17.82
		2	11.27	12.18	10.37	28.63	23.90	5.65	4.93	7.16	7.31	7.19	9.58	15.27	12.35	23.07	4.33	6.07	7.36	9.13	8.99	6.69	15.33
		3	18.01	17.03	18.99	79.29	33.48	5.32	5.63	5.18	5.93	4.52	13.94	0.00	87.49	24.94	9.78	10.36	6.54	7.01	5.78	6.72	12.33
		4	12.17	12.51	11.83	41.12	27.06	5.54	7.49	6.02	4.29	4.87	5.51	10.73	40.76	11.80	5.97	9.80	7.17	6.13	5.71	11.12	7.98
	Year	15/16	12.82	12.67	12.96	37.33	27.40	5.98	7.68	6.99	5.73	6.11	9.44	7.38	35.26	22.32	7.57	8.82	7.79	7.08	6.95	7.60	13.26
	Episodes	F/N	1,450.00	675.00	775.00	21.00	107.00	56.00	90.00	153.00	134.00	56.00	46.00	12.00	20.00	79.00	67.00	120.00	171.00	164.00	68.00	48.00	38.00
**South**	Quarter	1	11.63	11.17	12.09	0.00	47.30	9.39	6.30	6.33	7.06	7.22	9.30	7.65	0.00	30.45	7.33	12.41	10.62	6.16	14.33	14.24	13.27
		2	14.63	14.14	15.11	52.33	29.88	3.37	4.68	7.77	6.97	5.86	3.98	12.41	67.50	16.50	1.58	8.64	9.74	9.49	6.69	8.99	6.89
		3	14.98	13.94	16.03	53.69	21.71	8.68	5.35	4.41	4.80	5.56	6.86	14.38	55.30	28.39	8.43	6.73	8.79	5.66	4.50	12.22	14.20
		4	15.12	15.11	15.13	58.38	28.50	9.08	8.83	6.27	5.70	4.35	9.97	4.91	39.70	30.80	8.19	10.89	9.23	7.60	6.02	11.32	12.41
	Year	15/16	14.11	13.62	14.60	41.43	31.78	7.66	6.34	6.20	6.12	5.72	7.57	9.74	40.61	26.62	6.41	9.69	9.59	7.23	7.85	11.68	11.71
	Episodes	F/N	1,250.00	558.00	692.00	13.00	100.00	64.00	62.00	113.00	117.00	43.00	31.00	15.00	14.00	79.00	50.00	87.00	170.00	140.00	62.00	57.00	33.00
**London**	Quarter	1	22.20	20.80	23.61	74.68	45.83	19.09	15.56	12.05	6.02	2.60	11.32	0.00	76.92	44.99	21.36	14.88	12.14	7.03	6.30	11.38	17.46
		2	17.22	13.22	21.22	34.84	34.64	16.91	13.63	5.13	5.15	4.05	4.61	0.00	61.35	37.37	16.81	11.21	13.78	6.17	7.46	11.39	25.45
		3	17.59	17.42	17.76	23.53	57.72	20.25	10.28	8.02	7.57	10.77	6.09	12.57	43.18	39.35	16.23	12.27	9.92	8.73	14.81	5.02	10.33
		4	24.88	23.45	26.31	86.05	50.36	18.78	9.00	8.37	6.78	7.48	13.47	10.72	90.50	58.25	20.61	13.33	11.23	10.18	8.34	8.77	15.57
	Year	15/16	20.56	18.81	22.30	55.36	47.20	18.76	12.06	8.39	6.39	6.25	8.96	5.91	68.42	45.24	18.79	12.93	11.76	8.07	9.21	9.13	17.17
	Episodes	F/N	1,745.00	789.00	956.00	24.00	184.00	146.00	84.00	220.00	89.00	21.00	17.00	4.00	30.00	173.00	144.00	102.00	325.00	107.00	34.00	21.00	20.00
**Midlands And East**	Quarter	1	9.23	8.42	10.04	0.00	28.21	7.34	8.56	8.22	4.76	4.80	8.67	5.22	0.00	35.96	4.15	6.91	10.99	6.61	11.04	8.23	6.44
		2	8.98	9.04	8.93	19.04	27.61	4.52	8.87	7.21	3.71	4.01	6.39	0.00	0.00	34.58	5.28	6.80	9.29	3.97	4.92	7.87	7.63
		3	17.64	18.51	16.77	83.52	22.91	6.60	3.47	6.70	6.86	7.05	7.80	21.66	72.17	23.33	2.80	8.48	9.46	8.00	6.17	8.37	12.16
		4	13.78	12.48	15.08	50.63	18.71	11.41	3.92	4.16	6.39	7.79	6.40	2.88	49.38	25.11	8.17	9.88	6.96	5.41	6.29	14.38	10.12
	Year	15/16	12.43	12.12	12.75	38.53	24.25	7.54	6.16	6.53	5.45	5.95	7.30	7.35	30.75	29.66	5.16	8.05	9.13	5.99	7.09	9.80	9.11
	Episodes	F/N	1,071.00	486.00	585.00	13.00	76.00	60.00	48.00	110.00	99.00	42.00	28.00	10.00	11.00	91.00	39.00	57.00	157.00	109.00	54.00	44.00	23.00

Mean weekly incidence rate per 100,000 Persons.

Pleurisy (ICD10: R091)

		All ages			Male									Female								
		Male & Female	Male	Female	<1yr	1-4yrs	5-14yrs	15-24yrs	25-44yrs	45-64yrs	65-74yrs	75-84yrs	85+yrs	<1yr	1-4yrs	5-14yrs	15-24yrs	25-44yrs	45-64yrs	65-74yrs	75-84yrs	85+yrs
**4 weekly**	1	1.30	1.71	0.89	0.00	0.00	0.00	0.00	0.46	0.68	1.22	2.15	10.85	0.00	0.00	0.00	0.00	0.65	0.85	1.49	2.85	2.15
	2	1.62	1.99	1.26	0.00	0.00	0.00	1.21	0.54	0.55	2.20	6.65	6.72	0.00	0.00	0.00	0.48	0.31	0.68	2.16	6.60	1.09
	3	1.84	2.97	0.72	0.00	0.00	0.00	0.00	0.20	0.72	4.86	8.67	12.27	0.00	0.00	0.00	0.00	0.77	1.04	1.83	1.41	1.39
	4	1.27	1.68	0.86	0.00	0.00	0.00	0.00	0.57	0.40	5.11	4.32	4.76	0.00	0.00	0.00	0.50	0.76	0.52	1.42	0.68	3.84
	5	1.40	1.80	1.00	0.00	0.00	0.00	0.37	0.37	1.36	2.07	6.21	5.82	0.00	0.00	0.00	0.25	0.15	1.52	2.74	2.01	2.34
	6	1.53	1.91	1.15	0.00	0.00	0.80	0.00	0.32	0.79	1.48	7.09	6.70	0.00	0.00	0.00	0.00	1.08	1.41	2.88	4.07	0.96
	7	1.29	1.81	0.77	0.00	0.00	0.00	0.00	0.14	1.04	2.55	6.24	6.35	0.00	0.00	0.00	0.00	1.26	1.37	0.00	3.18	1.12
	8	1.14	1.59	0.69	0.00	0.00	0.00	0.33	0.12	0.90	2.64	2.32	7.99	0.00	0.00	0.29	0.00	0.88	1.16	2.09	0.64	1.13
	9	1.38	1.41	1.34	0.00	0.00	0.00	0.40	0.27	1.26	3.41	4.94	2.43	0.00	0.00	0.00	0.21	0.66	1.44	2.19	0.51	7.10
	10	1.49	1.82	1.15	0.00	0.00	0.00	0.40	0.46	0.92	3.52	4.78	6.33	0.00	0.00	0.00	0.36	1.41	2.32	1.75	1.47	3.04
	11	1.23	1.42	1.04	0.00	0.00	0.00	0.00	0.12	1.49	1.35	2.91	6.90	0.00	0.00	0.00	0.41	0.97	2.17	1.84	1.28	2.68
	12	1.79	2.67	0.91	0.00	0.00	0.00	0.00	0.35	1.19	2.34	4.08	16.06	0.00	0.00	0.00	0.36	1.36	0.56	1.11	1.29	3.54
	13	1.93	2.23	1.64	0.00	0.00	0.00	0.51	0.64	1.07	4.15	5.60	8.09	0.00	0.00	0.00	0.53	0.67	1.08	0.86	5.00	6.61
**Quarter**	1	1.57	2.18	0.95	0.00	0.00	0.00	0.37	0.41	0.66	2.64	5.54	10.02	0.00	0.00	0.00	0.15	0.58	0.86	1.80	3.56	1.59
	2	1.44	1.91	0.98	0.00	0.00	0.25	0.11	0.39	0.91	3.31	5.66	6.57	0.00	0.00	0.00	0.23	0.70	1.10	2.16	2.08	2.54
	3	1.19	1.42	0.96	0.00	0.00	0.00	0.22	0.23	0.93	2.88	3.92	4.59	0.00	0.00	0.09	0.17	0.96	1.55	1.54	1.78	2.53
	4	1.68	2.15	1.22	0.00	0.00	0.00	0.26	0.38	1.27	2.42	4.96	10.04	0.00	0.00	0.00	0.37	1.08	1.41	1.38	2.16	4.53
**Year**	15/16	1.47	1.92	1.03	0.00	0.00	0.06	0.24	0.35	0.95	2.81	5.02	7.85	0.00	0.00	0.02	0.23	0.84	1.23	1.71	2.39	2.83
**Episodes**	F/N	611.00	308.00	303.00	0.00	0.00	2.00	9.00	31.00	76.00	75.00	72.00	43.00	0.00	0.00	1.00	10.00	74.00	96.00	50.00	44.00	28.00

Mean weekly incidence rate per 100,000 Persons.

Pleurisy (ICD10: R091)

			All ages			Male								Female							
			Male & Female	Male	Female	<1yr	1-4yrs	5-14yrs	15-24yrs	25-44yrs	45-64yrs	65-74yrs	75-84yrs	85+yrs	<1yr	1-4yrs	5-14yrs	15-24yrs	25-44yrs	45-64yrs	65-74yrs	75-84yrs	85+yrs
**North**	Quarter	1	1.78	2.45	1.11	0.00	0.00	0.00	0.00	0.29	0.49	2.45	5.66	13.17	0.00	0.00	0.00	0.59	0.91	1.03	0.00	3.76	3.68
		2	1.59	1.76	1.41	0.00	0.00	0.00	0.45	0.14	1.17	3.02	3.70	7.38	0.00	0.00	0.00	0.31	0.84	1.06	1.04	3.93	5.51
		3	1.41	1.83	1.00	0.00	0.00	0.00	0.00	0.00	2.06	3.11	4.27	7.01	0.00	0.00	0.35	0.25	1.83	1.92	1.87	1.18	1.60
		4	2.08	2.31	1.84	0.00	0.00	0.00	0.22	1.05	2.04	2.37	4.64	10.47	0.00	0.00	0.00	0.68	2.24	2.20	2.07	1.66	7.73
	Year	15/16	1.72	2.09	1.35	0.00	0.00	0.00	0.17	0.38	1.45	2.73	4.57	9.52	0.00	0.00	0.09	0.46	1.47	1.56	1.26	2.61	4.69
	Episodes	F/N	236.00	112.00	124.00	0.00	0.00	0.00	2.00	10.00	38.00	26.00	21.00	15.00	0.00	0.00	1.00	6.00	35.00	39.00	14.00	15.00	14.00
**South**	Quarter	1	1.83	2.86	0.81	0.00	0.00	0.00	0.85	0.76	1.09	3.39	5.99	13.65	0.00	0.00	0.00	0.00	0.45	0.43	1.06	5.34	0.00
		2	1.74	2.75	0.72	0.00	0.00	0.48	0.00	0.22	0.22	3.77	9.85	10.22	0.00	0.00	0.00	0.00	0.92	0.85	2.50	0.82	1.38
		3	1.36	1.21	1.52	0.00	0.00	0.00	0.40	0.33	0.44	2.08	2.37	5.28	0.00	0.00	0.00	0.00	0.68	2.30	3.09	4.08	3.52
		4	1.66	2.32	1.00	0.00	0.00	0.00	0.36	0.36	0.00	2.40	10.26	7.54	0.00	0.00	0.00	0.41	1.86	1.35	1.37	1.40	2.64
	Year	15/16	1.65	2.29	1.01	0.00	0.00	0.12	0.40	0.42	0.43	2.90	7.18	9.14	0.00	0.00	0.00	0.11	0.99	1.23	1.99	2.88	1.90
	Episodes	F/N	163.00	85.00	78.00	0.00	0.00	1.00	4.00	7.00	8.00	22.00	29.00	14.00	0.00	0.00	0.00	1.00	18.00	24.00	16.00	14.00	5.00
**London**	Quarter	1	1.41	1.74	1.07	0.00	0.00	0.00	0.64	0.17	0.64	1.94	6.46	5.84	0.00	0.00	0.00	0.00	0.32	1.16	4.62	3.52	0.00
		2	0.85	1.12	0.58	0.00	0.00	0.00	0.00	0.70	1.35	5.31	2.68	0.00	0.00	0.00	0.00	0.62	0.81	1.40	2.37	0.00	0.00
		3	0.82	1.08	0.56	0.00	0.00	0.00	0.49	0.58	0.52	3.43	4.67	0.00	0.00	0.00	0.00	0.44	0.79	0.58	0.00	0.00	3.23
		4	1.96	2.72	1.20	0.00	0.00	0.00	0.46	0.13	1.70	2.15	3.83	16.21	0.00	0.00	0.00	0.39	0.24	1.02	0.00	2.93	6.20
	Year	15/16	1.27	1.68	0.86	0.00	0.00	0.00	0.40	0.39	1.06	3.19	4.40	5.72	0.00	0.00	0.00	0.36	0.53	1.04	1.71	1.64	2.43
	Episodes	F/N	93.00	49.00	44.00	0.00	0.00	0.00	3.00	10.00	15.00	10.00	7.00	4.00	0.00	0.00	0.00	3.00	15.00	13.00	6.00	4.00	3.00
**Midlands And East**	Quarter	1	1.24	1.67	0.81	0.00	0.00	0.00	0.00	0.41	0.39	2.77	4.04	7.41	0.00	0.00	0.00	0.00	0.64	0.82	1.52	1.64	2.67
		2	1.61	2.01	1.21	0.00	0.00	0.51	0.00	0.48	0.90	1.14	6.41	8.68	0.00	0.00	0.00	0.00	0.24	1.09	2.75	3.55	3.27
		3	1.16	1.56	0.76	0.00	0.00	0.00	0.00	0.00	0.71	2.92	4.35	6.09	0.00	0.00	0.00	0.00	0.55	1.41	1.19	1.88	1.78
		4	1.03	1.24	0.82	0.00	0.00	0.00	0.00	0.00	1.33	2.77	1.11	5.94	0.00	0.00	0.00	0.00	0.00	1.09	2.09	2.66	1.55
	Year	15/16	1.26	1.61	0.90	0.00	0.00	0.12	0.00	0.22	0.84	2.41	3.92	7.01	0.00	0.00	0.00	0.00	0.35	1.10	1.89	2.44	2.30
	Episodes	F/N	119.00	62.00	57.00	0.00	0.00	1.00	0.00	4.00	15.00	17.00	15.00	10.00	0.00	0.00	0.00	0.00	6.00	20.00	14.00	11.00	6.00

Mean weekly incidence rate per 100,000 Persons.

Pneumonia and Pneumonitis (ICD10: J12 - J18)

		All ages			Male								Female							
		Male & Female	Male	Female	<1yr	1-4yrs	5-14yrs	15-24yrs	25-44yrs	45-64yrs	65-74yrs	75-84yrs	85+yrs	<1yr	1-4yrs	5-14yrs	15-24yrs	25-44yrs	45-64yrs	65-74yrs	75-84yrs	85+yrs
**4 weekly**	1	3.10	4.31	1.89	0.00	0.81	0.00	0.42	0.00	1.28	3.24	5.03	28.03	0.00	1.01	0.72	0.00	0.67	0.77	2.26	2.95	8.66
	2	1.87	1.82	1.92	0.00	1.09	0.00	0.34	0.59	0.85	1.36	6.69	5.41	0.00	1.17	0.00	0.36	0.65	0.53	4.81	1.99	7.75
	3	2.38	2.60	2.15	0.00	1.05	0.00	0.55	0.37	1.22	1.90	2.58	15.76	0.00	1.07	0.00	0.00	0.72	0.00	2.70	3.61	11.24
	4	2.24	2.41	2.06	0.00	0.00	0.00	0.34	0.25	0.97	1.33	9.84	8.96	0.00	0.00	0.00	0.46	0.66	0.64	4.01	3.65	9.16
	5	2.12	3.04	1.20	0.00	1.84	0.00	0.41	0.18	1.35	2.53	10.62	10.40	0.00	0.91	0.42	0.00	0.00	1.38	3.71	2.00	2.36
	6	3.21	3.82	2.61	0.00	2.80	0.00	1.09	0.43	2.15	1.43	7.20	19.26	0.00	0.80	0.43	0.20	0.49	1.35	4.25	6.12	9.87
	7	4.26	5.60	2.93	0.00	3.46	0.41	0.20	0.18	0.63	4.68	9.53	31.31	0.00	0.00	0.47	0.00	0.76	1.94	2.06	8.40	12.70
	8	2.90	3.04	2.76	0.00	5.81	1.11	0.00	0.45	0.61	2.45	10.17	6.76	0.00	4.43	1.17	0.00	0.83	2.77	1.43	4.56	9.69
	9	3.26	2.97	3.54	0.00	5.59	0.37	0.00	0.25	2.26	2.66	6.54	9.08	0.00	3.18	0.76	0.00	1.09	2.32	3.68	8.85	12.03
	10	3.89	5.13	2.65	0.00	4.03	0.00	0.21	1.35	1.26	3.55	11.01	24.76	0.00	1.23	1.49	0.94	1.19	3.67	2.95	2.18	10.23
	11	4.12	5.90	2.34	4.05	7.12	1.11	0.90	0.91	3.28	3.78	10.46	21.46	0.00	3.73	0.00	0.49	0.70	1.80	2.35	5.19	6.82
	12	4.21	3.99	4.43	5.95	3.04	0.76	0.40	1.66	1.33	4.15	5.40	13.26	0.00	1.81	0.38	0.37	1.40	1.96	4.51	7.42	22.04
	13	2.56	3.16	1.95	0.00	0.68	0.37	0.70	0.29	1.60	4.79	3.12	16.95	0.00	0.00	0.73	0.00	1.13	1.54	1.12	4.32	8.68
**Quarter**	1	2.50	3.02	1.98	0.00	0.97	0.00	0.43	0.30	1.13	2.25	4.79	17.29	0.00	1.08	0.28	0.11	0.68	0.46	3.18	2.86	9.17
	2	2.75	3.38	2.11	0.00	1.71	0.00	0.63	0.32	1.37	1.86	9.36	15.19	0.00	0.53	0.41	0.20	0.43	1.31	3.94	4.29	7.89
	3	3.15	3.44	2.86	0.00	4.89	0.58	0.00	0.50	1.18	3.16	8.11	12.57	0.00	2.72	0.81	0.06	0.86	2.38	2.08	6.41	10.41
	4	3.89	4.82	2.95	2.86	3.69	0.64	0.63	0.93	2.04	4.29	7.75	20.57	0.00	1.58	0.54	0.46	1.16	2.10	3.00	5.12	12.59
**Year**	15/16	3.09	3.69	2.48	0.75	2.83	0.31	0.43	0.52	1.44	2.92	7.51	16.48	0.00	1.48	0.51	0.21	0.79	1.57	3.05	4.68	10.07
**Episodes**	F/N	985.00	488.00	497.00	2.00	40.00	11.00	17.00	48.00	108.00	77.00	109.00	76.00	0.00	19.00	17.00	8.00	70.00	121.00	84.00	81.00	97.00

Mean weekly incidence rate per 100,000 Persons.

Pneumonia and Pneumonitis (ICD10: J12-J18)

			All ages			Male								Female							
			Male & Female	Male	Female	<1yr	1-4yrs	5-14yrs	15-24yrs	25-44yrs	45-64yrs	65-74yrs	75-84yrs	85+yrs	<1yr	1-4yrs	5-14yrs	15-24yrs	25-44yrs	45-64yrs	65-74yrs	75-84yrs	85+yrs
**North**	Quarter	1	46.31	20.84	71.78	0.00	11.58	19.65	11.21	17.80	23.66	34.79	35.49	33.40	427.35	23.64	10.46	22.26	24.05	23.20	31.92	36.83	46.34
		2	26.20	24.01	28.38	0.00	29.65	12.01	13.19	16.62	22.84	30.18	37.71	53.91	49.97	25.28	8.67	19.02	20.84	25.53	25.65	38.26	42.24
		3	30.21	28.70	31.73	46.87	38.16	14.59	11.33	20.31	24.33	31.14	31.01	40.53	70.08	22.73	12.24	18.43	21.03	30.51	36.61	37.33	36.58
		4	31.92	30.88	32.97	66.45	16.69	14.61	15.50	18.75	24.98	39.18	37.88	43.88	65.36	18.62	14.24	16.61	26.98	31.16	40.66	41.54	41.54
	Year	15/16	33.63	26.20	41.06	29.05	23.88	15.20	12.86	18.38	23.97	33.92	35.57	42.95	151.53	22.49	11.46	19.03	23.30	27.67	33.84	38.55	41.67
	Episodes	F/N	4,319.00	1,994.00	2,325.00	22.00	96.00	142.00	173.00	434.00	571.00	313.00	172.00	71.00	26.00	81.00	106.00	243.00	519.00	663.00	333.00	230.00	124.00
**South**	Quarter	1	19.34	21.51	17.17	0.00	22.49	7.78	16.15	21.47	23.29	27.30	31.29	43.80	0.00	14.97	7.72	15.86	16.21	18.97	22.26	29.03	29.48
		2	18.83	18.82	18.85	0.00	19.22	8.84	13.82	14.33	17.80	29.16	30.39	35.79	0.00	19.05	7.28	13.89	18.41	22.66	22.21	36.85	29.29
		3	30.39	29.75	31.03	60.81	34.64	12.73	15.95	18.63	20.63	25.71	34.32	44.31	56.38	27.17	16.31	19.38	23.33	29.94	24.54	41.29	40.92
		4	26.87	27.55	26.19	39.27	29.39	14.12	12.56	18.21	27.76	28.10	38.35	40.14	34.28	15.54	16.05	27.86	21.66	29.97	29.97	32.75	27.60
	Year	15/16	23.91	24.46	23.36	25.29	26.49	10.93	14.58	18.16	22.47	27.58	33.68	40.99	22.89	19.11	11.92	19.41	19.94	25.47	24.84	34.94	31.74
	Episodes	F/N	3,130.00	1,493.00	1,637.00	11.00	84.00	91.00	139.00	328.00	431.00	209.00	137.00	63.00	9.00	57.00	92.00	175.00	355.00	491.00	198.00	171.00	89.00
**London**	Quarter	1	23.58	23.53	23.63	0.00	26.30	14.93	13.94	13.72	25.05	27.42	40.99	49.42	0.00	23.81	10.54	15.68	16.64	26.12	41.88	38.07	39.94
		2	28.21	30.29	26.13	42.62	41.61	14.41	12.84	18.35	24.11	34.98	34.81	48.86	39.65	35.45	16.92	15.69	19.37	28.75	37.44	19.95	22.00
		3	33.58	29.78	37.38	69.70	41.15	15.83	11.23	17.69	24.85	27.86	31.44	28.22	59.52	47.39	13.47	14.44	17.48	31.03	40.97	49.41	62.74
		4	33.48	30.95	36.02	59.36	30.69	17.46	16.69	14.65	26.38	22.46	47.73	43.10	38.76	40.09	15.49	17.78	21.50	33.58	50.13	54.13	52.74
	Year	15/16	29.78	28.68	30.89	43.23	34.86	15.69	13.73	16.08	25.12	28.07	38.91	42.41	34.56	36.75	14.13	15.94	18.80	29.94	42.75	40.65	44.51
	Episodes	F/N	2,989.00	1,353.00	1,636.00	25.00	134.00	125.00	100.00	426.00	350.00	91.00	73.00	29.00	17.00	142.00	109.00	127.00	532.00	398.00	160.00	99.00	52.00
**Midlands And East**	Quarter	1	33.14	24.96	41.33	0.00	43.80	14.59	15.18	17.38	19.42	29.87	34.01	50.37	132.63	38.72	10.34	23.17	24.42	25.62	31.63	41.15	44.29
		2	29.32	31.56	27.07	24.42	53.86	10.10	16.35	15.71	18.08	26.32	38.55	80.69	24.81	22.88	13.32	24.15	24.08	25.15	25.79	38.21	45.21
		3	36.90	41.16	32.63	82.29	47.83	19.06	10.97	25.04	27.26	27.99	42.50	87.53	29.33	43.97	16.74	23.60	22.89	32.79	33.13	40.56	50.70
		4	29.70	30.69	28.72	49.69	33.10	19.14	12.36	16.52	23.23	29.42	44.47	48.28	27.65	28.03	10.74	21.84	24.05	26.95	35.14	47.60	36.44
	Year	15/16	32.22	32.07	32.37	39.30	44.43	15.79	13.69	18.62	22.02	28.42	39.97	66.37	53.11	33.30	12.75	23.16	23.86	27.61	31.49	41.99	44.01
	Episodes	F/N	3,344.00	1,529.00	1,815.00	13.00	138.00	126.00	105.00	305.00	399.00	201.00	150.00	92.00	7.00	101.00	97.00	164.00	406.00	501.00	236.00	190.00	113.00

Mean weekly incidence rate per 100,000 Persons.

Respiratory System Diseases (ICD10: J00-J99)

		All ages			Male								Female							
		Male & Female	Male	Female	<1yr	1-4yrs	5-14yrs	15-24yrs	25-44yrs	45-64yrs	65-74yrs	75-84yrs	85+yrs	<1yr	1-4yrs	5-14yrs	15-24yrs	25-44yrs	45-64yrs	65-74yrs	75-84yrs	85+yrs
**4 weekly**	1	319.35	303.17	335.53	0.00	976.89	344.85	177.99	155.84	145.68	239.16	288.28	399.82	0.00	843.47	334.88	298.13	292.40	254.70	279.87	322.12	394.24
	2	348.24	324.70	371.77	390.63	755.55	437.37	218.37	176.82	135.18	196.33	272.11	339.94	781.25	589.18	346.29	327.94	293.29	209.52	229.10	249.36	320.03
	3	320.39	293.27	347.51	562.65	744.82	244.47	131.82	122.80	122.09	191.10	243.85	275.85	813.03	670.38	258.51	235.19	227.90	184.76	210.89	239.34	287.62
	4	247.86	259.85	235.87	622.05	539.42	135.86	118.19	104.02	112.61	166.40	237.87	302.27	269.07	432.80	114.19	209.94	180.16	167.53	211.88	260.23	277.06
	5	347.42	349.43	345.42	935.92	824.09	217.99	149.15	108.92	119.13	199.47	259.09	331.13	682.78	737.75	220.87	251.37	208.05	193.29	245.73	242.20	326.72
	6	529.68	509.77	549.59	1215.81	1449.18	310.14	155.25	149.07	170.04	268.49	376.24	493.71	1129.99	1336.69	333.53	309.71	302.40	312.87	297.96	388.21	534.93
	7	593.07	571.34	614.80	1546.41	1698.93	309.94	153.53	145.24	172.29	261.62	376.00	478.07	1482.54	1656.01	316.82	299.59	281.53	270.70	355.83	384.25	485.93
	8	819.33	837.05	801.62	3269.56	1993.14	460.29	176.27	164.98	189.32	321.49	387.58	570.83	2433.44	1927.17	459.31	333.80	350.29	347.03	411.63	425.91	525.97
	9	627.71	614.78	640.64	1919.72	1134.30	303.01	199.20	192.24	241.78	387.51	527.96	627.32	1579.13	1029.91	342.53	307.25	369.41	402.52	433.37	562.80	738.87
	10	737.02	728.10	745.95	2175.56	1715.95	556.57	226.30	218.35	271.47	366.63	432.14	589.92	1710.02	1540.66	559.54	396.87	460.50	465.32	466.75	492.78	621.13
	11	696.38	682.53	710.23	1892.41	1548.69	492.67	254.07	226.00	272.60	385.88	455.68	614.78	1586.40	1400.87	521.39	411.03	479.63	446.33	447.17	491.90	607.32
	12	625.53	607.45	643.60	1774.35	1239.24	493.59	216.02	224.21	228.73	353.54	414.58	522.83	1343.75	1190.61	532.75	374.30	414.63	382.26	461.97	485.81	606.30
	13	487.70	478.36	497.04	1529.42	918.50	329.35	178.37	156.82	172.10	290.12	303.21	427.35	1301.51	822.15	326.53	300.62	306.58	284.96	354.10	386.83	390.11
**Quarter**	1	328.56	306.75	350.37	293.31	837.38	342.43	176.21	152.13	135.19	211.19	269.63	343.25	490.55	711.97	314.89	287.94	272.83	219.28	243.02	274.26	338.60
	2	386.87	382.83	390.91	939.10	988.65	227.54	140.31	122.29	137.26	214.56	295.05	380.73	727.99	885.35	228.52	258.65	233.28	227.82	260.28	302.60	393.71
	3	701.39	697.75	705.02	2325.41	1625.09	397.96	186.25	176.28	213.44	332.20	443.10	580.02	1864.47	1542.27	407.60	328.84	354.12	361.57	410.96	472.51	602.85
	4	621.39	607.65	635.13	1791.87	1304.79	450.45	218.57	205.57	231.51	349.77	391.81	524.50	1455.18	1195.67	474.31	367.18	412.61	387.79	429.95	457.65	535.87
**Year**	15/16	511.66	500.80	522.53	1346.00	1191.16	356.40	181.06	164.85	180.33	278.30	350.69	458.40	1140.60	1085.93	358.55	311.72	319.99	300.79	337.83	378.28	469.04
**Episodes**	F/N	192176.00	81056.00	111120.00	2446.00	16874.00	12137.00	6760.00	14000.00	13701.00	7602.00	5126.00	2410.00	1947.00	14907.00	11753.00	11624.00	27246.00	22590.00	9815.00	6698.00	4540.00

Mean weekly incidence rate per 100,000 Persons.

Respiratory System Diseases (ICD10: J00-J99)

			All ages			Male									Female								
			Male & Female	Male	Female	<1yr	1-4yrs	5-14yrs	15-24yrs	25-44yrs	45-64yrs	65-74yrs	75-84yrs	85+yrs	<1yr	1-4yrs	5-14yrs	15-24yrs	25-44yrs	45-64yrs	65-74yrs	75-84yrs	85+yrs
**North**	Quarter	1	344.13	340.94	347.32	389.91	983.55	331.64	184.13	164.38	139.17	223.52	289.09	363.06	192.31	754.42	347.29	328.28	320.95	246.77	250.65	340.23	344.94
		2	383.35	368.01	398.70	843.17	942.86	202.82	134.54	124.81	141.26	229.61	322.28	370.70	612.53	950.36	228.65	278.07	251.85	246.34	267.87	319.82	432.83
		3	716.84	721.55	712.13	2,496.59	1,613.50	376.28	174.42	179.71	235.16	349.31	480.36	588.63	1,795.51	1,535.81	391.32	316.13	377.10	401.14	436.97	508.47	646.74
		4	650.09	638.71	661.47	2,064.27	1,309.71	425.76	206.29	214.53	234.24	354.26	400.75	538.61	1,684.95	1,170.27	459.52	333.27	418.98	394.24	438.68	473.50	579.81
	Year	15/16	525.99	519.59	532.39	1,460.10	1,214.24	335.85	175.44	171.68	188.34	290.40	373.64	466.63	1,082.90	1,103.99	358.63	314.30	343.67	323.49	350.24	411.70	502.57
	Episodes	F/N	61,262.00	25,418.00	35,844.00	825.00	4,808.00	3,285.00	2,309.00	4,120.00	4,641.00	2,781.00	1,874.00	775.00	613.00	4,214.00	3,402.00	4,188.00	7,890.00	7,878.00	3,582.00	2,513.00	1,564.00
**South**	Quarter	1	267.23	257.49	276.97	76.16	766.81	292.09	161.91	145.35	123.17	181.74	241.48	328.73	160.26	633.94	255.99	264.60	250.47	190.30	200.60	217.25	319.30
		2	382.67	382.37	382.98	1,086.97	944.96	201.23	134.37	116.36	118.88	193.03	264.11	381.41	846.03	819.93	206.75	245.53	238.34	195.25	229.00	270.08	395.87
		3	723.07	714.62	731.52	2,676.00	1,634.04	336.10	191.77	177.03	202.46	299.35	396.69	518.18	2,246.60	1,532.73	372.84	368.12	348.70	324.56	380.72	410.28	599.14
		4	617.70	611.63	623.77	1,876.42	1,313.27	419.15	225.43	212.06	228.01	343.76	401.92	484.66	1,422.43	1,217.21	434.09	383.70	429.25	383.02	409.35	404.28	530.63
	Year	15/16	499.93	493.80	506.07	1,437.33	1,167.57	314.16	179.26	163.63	169.26	256.16	327.48	429.31	1,173.62	1,054.09	319.62	316.77	318.81	275.35	306.89	326.96	462.54
	Episodes	F/N	43,949.00	18,723.00	25,226.00	545.00	3,713.00	2,615.00	1,722.00	2,971.00	3,241.00	1,936.00	1,321.00	659.00	427.00	3,155.00	2,510.00	2,834.00	5,689.00	5,277.00	2,447.00	1,598.00	1,289.00
**London**	Quarter	1	360.81	359.28	362.34	480.77	948.31	429.38	184.57	154.53	151.96	239.56	272.00	372.47	289.79	843.47	394.34	288.22	262.34	246.73	288.19	297.89	350.07
		2	435.80	434.25	437.35	1,089.79	1,141.78	303.18	157.36	124.68	159.16	238.64	327.00	366.64	880.25	1,035.94	283.58	245.41	221.33	253.71	304.07	364.29	347.58
		3	692.82	672.14	713.50	2,024.37	1,681.80	510.41	190.46	160.28	209.58	341.41	476.31	454.66	1,761.41	1,665.60	497.65	275.05	308.83	371.52	441.33	533.47	566.64
		4	630.06	603.73	656.38	1,644.94	1,391.53	540.14	209.56	181.76	229.06	349.75	392.10	494.73	1,460.48	1,294.28	570.88	335.03	350.81	385.74	461.04	515.59	533.57
	Year	15/16	531.76	518.98	544.54	1,316.29	1,292.75	447.56	185.94	155.81	188.23	293.42	367.33	423.50	1,104.82	1,211.42	439.14	286.85	287.05	315.77	375.31	429.47	451.05
	Episodes	F/N	45,984.00	19,494.00	26,490.00	696.00	5,068.00	3,612.00	1,349.00	4,175.00	2,656.00	966.00	681.00	291.00	603.00	4,688.00	3,454.00	2,305.00	8,202.00	4,248.00	1,418.00	1,041.00	531.00
**Midlands And East**	Quarter	1	342.07	269.27	414.86	226.42	650.84	316.61	174.22	144.25	126.46	199.96	275.96	308.75	1,319.83	616.04	261.93	270.65	257.56	193.33	232.65	241.66	340.09
		2	345.66	346.71	344.62	736.46	925.01	202.91	134.98	123.32	129.73	196.94	266.83	404.18	573.14	735.16	195.10	265.60	221.59	215.98	240.21	256.24	398.54
		3	672.81	682.68	662.94	2,104.68	1,571.01	369.05	188.34	188.11	206.56	338.72	419.05	758.63	1,654.37	1,434.96	368.59	356.08	381.85	349.07	384.80	437.83	598.87
		4	587.72	576.52	598.92	1,581.86	1,204.65	416.73	233.02	213.92	234.74	351.31	372.46	580.00	1,252.87	1,100.91	432.75	416.72	451.39	388.17	410.75	437.22	499.46
	Year	15/16	488.96	470.83	507.10	1,170.27	1,090.08	328.03	183.59	168.28	175.51	273.23	334.31	514.15	1,201.05	974.20	316.82	328.95	330.43	288.55	318.87	345.01	460.00
	Episodes	F/N	40,981.00	17,421.00	23,560.00	380.00	3,285.00	2,625.00	1,380.00	2,734.00	3,163.00	1,919.00	1,250.00	685.00	304.00	2,850.00	2,387.00	2,297.00	5,465.00	5,187.00	2,368.00	1,546.00	1,156.00

Mean weekly incidence rate per 100,000 Persons.

Rubella (ICD10: B06)

		All ages			Male									Female								
		Male & Female	Male	Female	<1yr	1-4yrs	5-14yrs	15-24yrs	25-44yrs	45-64yrs	65-74yrs	75-84yrs	85+yrs	<1yr	1-4yrs	5-14yrs	15-24yrs	25-44yrs	45-64yrs	65-74yrs	75-84yrs	85+yrs
**4 weekly**	1	0.00	0.00	0.00	0.00	0.00	0.00	0.00	0.00	0.00	0.00	0.00	0.00	0.00	0.00	0.00	0.00	0.00	0.00	0.00	0.00	0.00
	2	0.00	0.00	0.00	0.00	0.00	0.00	0.00	0.00	0.00	0.00	0.00	0.00	0.00	0.00	0.00	0.00	0.00	0.00	0.00	0.00	0.00
	3	0.00	0.00	0.00	0.00	0.00	0.00	0.00	0.00	0.00	0.00	0.00	0.00	0.00	0.00	0.00	0.00	0.00	0.00	0.00	0.00	0.00
	4	0.00	0.00	0.00	0.00	0.00	0.00	0.00	0.00	0.00	0.00	0.00	0.00	0.00	0.00	0.00	0.00	0.00	0.00	0.00	0.00	0.00
	5	0.00	0.00	0.00	0.00	0.00	0.00	0.00	0.00	0.00	0.00	0.00	0.00	0.00	0.00	0.00	0.00	0.00	0.00	0.00	0.00	0.00
	6	0.00	0.00	0.00	0.00	0.00	0.00	0.00	0.00	0.00	0.00	0.00	0.00	0.00	0.00	0.00	0.00	0.00	0.00	0.00	0.00	0.00
	7	0.00	0.00	0.00	0.00	0.00	0.00	0.00	0.00	0.00	0.00	0.00	0.00	0.00	0.00	0.00	0.00	0.00	0.00	0.00	0.00	0.00
	8	0.04	0.00	0.09	0.00	0.00	0.00	0.00	0.00	0.00	0.00	0.00	0.00	0.00	0.80	0.00	0.00	0.00	0.00	0.00	0.00	0.00
	9	0.00	0.00	0.00	0.00	0.00	0.00	0.00	0.00	0.00	0.00	0.00	0.00	0.00	0.00	0.00	0.00	0.00	0.00	0.00	0.00	0.00
	10	0.00	0.00	0.00	0.00	0.00	0.00	0.00	0.00	0.00	0.00	0.00	0.00	0.00	0.00	0.00	0.00	0.00	0.00	0.00	0.00	0.00
	11	0.30	0.00	0.59	0.00	0.00	0.00	0.00	0.00	0.00	0.00	0.00	0.00	5.00	0.00	0.32	0.00	0.00	0.00	0.00	0.00	0.00
	12	0.00	0.00	0.00	0.00	0.00	0.00	0.00	0.00	0.00	0.00	0.00	0.00	0.00	0.00	0.00	0.00	0.00	0.00	0.00	0.00	0.00
	13	0.00	0.00	0.00	0.00	0.00	0.00	0.00	0.00	0.00	0.00	0.00	0.00	0.00	0.00	0.00	0.00	0.00	0.00	0.00	0.00	0.00
**Quarter**	1	0.00	0.00	0.00	0.00	0.00	0.00	0.00	0.00	0.00	0.00	0.00	0.00	0.00	0.00	0.00	0.00	0.00	0.00	0.00	0.00	0.00
	2	0.00	0.00	0.00	0.00	0.00	0.00	0.00	0.00	0.00	0.00	0.00	0.00	0.00	0.00	0.00	0.00	0.00	0.00	0.00	0.00	0.00
	3	0.01	0.00	0.03	0.00	0.00	0.00	0.00	0.00	0.00	0.00	0.00	0.00	0.00	0.25	0.00	0.00	0.00	0.00	0.00	0.00	0.00
	4	0.08	0.00	0.17	0.00	0.00	0.00	0.00	0.00	0.00	0.00	0.00	0.00	1.43	0.00	0.09	0.00	0.00	0.00	0.00	0.00	0.00
**Year**	15/16	0.03	0.00	0.05	0.00	0.00	0.00	0.00	0.00	0.00	0.00	0.00	0.00	0.38	0.06	0.02	0.00	0.00	0.00	0.00	0.00	0.00
**Episodes**	F/N	3.00	0.00	3.00	0.00	0.00	0.00	0.00	0.00	0.00	0.00	0.00	0.00	1.00	1.00	1.00	0.00	0.00	0.00	0.00	0.00	0.00

Mean weekly incidence rate per 100,000 Persons.

Rubella (ICD10: B06)

			All ages			Male								Female							
			Male & Female	Male	Female	<1yr	1-4yrs	5-14yrs	15-24yrs	25-44yrs	45-64yrs	65-74yrs	75-84yrs	85+yrs	<1yr	1-4yrs	5-14yrs	15-24yrs	25-44yrs	45-64yrs	65-74yrs	75-84yrs	85+yrs
**North**	Quarter	1	0.00	0.00	0.00	0.00	0.00	0.00	0.00	0.00	0.00	0.00	0.00	0.00	0.00	0.00	0.00	0.00	0.00	0.00	0.00	0.00	0.00
		2	0.00	0.00	0.00	0.00	0.00	0.00	0.00	0.00	0.00	0.00	0.00	0.00	0.00	0.00	0.00	0.00	0.00	0.00	0.00	0.00	0.00
		3	0.05	0.00	0.11	0.00	0.00	0.00	0.00	0.00	0.00	0.00	0.00	0.00	0.00	0.99	0.00	0.00	0.00	0.00	0.00	0.00	0.00
		4	0.34	0.00	0.67	0.00	0.00	0.00	0.00	0.00	0.00	0.00	0.00	0.00	5.71	0.00	0.36	0.00	0.00	0.00	0.00	0.00	0.00
	Year	15/16	0.10	0.00	0.21	0.00	0.00	0.00	0.00	0.00	0.00	0.00	0.00	0.00	1.51	0.24	0.10	0.00	0.00	0.00	0.00	0.00	0.00
	Episodes	F/N	3.00	0.00	3.00	0.00	0.00	0.00	0.00	0.00	0.00	0.00	0.00	0.00	1.00	1.00	1.00	0.00	0.00	0.00	0.00	0.00	0.00
**South**	Quarter	1	0.00	0.00	0.00	0.00	0.00	0.00	0.00	0.00	0.00	0.00	0.00	0.00	0.00	0.00	0.00	0.00	0.00	0.00	0.00	0.00	0.00
		2	0.00	0.00	0.00	0.00	0.00	0.00	0.00	0.00	0.00	0.00	0.00	0.00	0.00	0.00	0.00	0.00	0.00	0.00	0.00	0.00	0.00
		3	0.00	0.00	0.00	0.00	0.00	0.00	0.00	0.00	0.00	0.00	0.00	0.00	0.00	0.00	0.00	0.00	0.00	0.00	0.00	0.00	0.00
		4	0.00	0.00	0.00	0.00	0.00	0.00	0.00	0.00	0.00	0.00	0.00	0.00	0.00	0.00	0.00	0.00	0.00	0.00	0.00	0.00	0.00
	Year	15/16	0.00	0.00	0.00	0.00	0.00	0.00	0.00	0.00	0.00	0.00	0.00	0.00	0.00	0.00	0.00	0.00	0.00	0.00	0.00	0.00	0.00
	Episodes	F/N	0.00	0.00	0.00	0.00	0.00	0.00	0.00	0.00	0.00	0.00	0.00	0.00	0.00	0.00	0.00	0.00	0.00	0.00	0.00	0.00	0.00
**London**	Quarter	1	0.00	0.00	0.00	0.00	0.00	0.00	0.00	0.00	0.00	0.00	0.00	0.00	0.00	0.00	0.00	0.00	0.00	0.00	0.00	0.00	0.00
		2	0.00	0.00	0.00	0.00	0.00	0.00	0.00	0.00	0.00	0.00	0.00	0.00	0.00	0.00	0.00	0.00	0.00	0.00	0.00	0.00	0.00
		3	0.00	0.00	0.00	0.00	0.00	0.00	0.00	0.00	0.00	0.00	0.00	0.00	0.00	0.00	0.00	0.00	0.00	0.00	0.00	0.00	0.00
		4	0.00	0.00	0.00	0.00	0.00	0.00	0.00	0.00	0.00	0.00	0.00	0.00	0.00	0.00	0.00	0.00	0.00	0.00	0.00	0.00	0.00
	Year	15/16	0.00	0.00	0.00	0.00	0.00	0.00	0.00	0.00	0.00	0.00	0.00	0.00	0.00	0.00	0.00	0.00	0.00	0.00	0.00	0.00	0.00
	Episodes	F/N	0.00	0.00	0.00	0.00	0.00	0.00	0.00	0.00	0.00	0.00	0.00	0.00	0.00	0.00	0.00	0.00	0.00	0.00	0.00	0.00	0.00
**Midlands And East**	Quarter	1	0.00	0.00	0.00	0.00	0.00	0.00	0.00	0.00	0.00	0.00	0.00	0.00	0.00	0.00	0.00	0.00	0.00	0.00	0.00	0.00	0.00
		2	0.00	0.00	0.00	0.00	0.00	0.00	0.00	0.00	0.00	0.00	0.00	0.00	0.00	0.00	0.00	0.00	0.00	0.00	0.00	0.00	0.00
		3	0.00	0.00	0.00	0.00	0.00	0.00	0.00	0.00	0.00	0.00	0.00	0.00	0.00	0.00	0.00	0.00	0.00	0.00	0.00	0.00	0.00
		4	0.00	0.00	0.00	0.00	0.00	0.00	0.00	0.00	0.00	0.00	0.00	0.00	0.00	0.00	0.00	0.00	0.00	0.00	0.00	0.00	0.00
	Year	15/16	0.00	0.00	0.00	0.00	0.00	0.00	0.00	0.00	0.00	0.00	0.00	0.00	0.00	0.00	0.00	0.00	0.00	0.00	0.00	0.00	0.00
	Episodes	F/N	0.00	0.00	0.00	0.00	0.00	0.00	0.00	0.00	0.00	0.00	0.00	0.00	0.00	0.00	0.00	0.00	0.00	0.00	0.00	0.00	0.00

Mean weekly incidence rate per 100,000 Persons.

Scabies (ICD10: B86)

		All ages			Male								Female							
		Male & Female	Male	Female	<1yr	1-4yrs	5-14yrs	15-24yrs	25-44yrs	45-64yrs	65-74yrs	75-84yrs	85+yrs	<1yr	1-4yrs	5-14yrs	15-24yrs	25-44yrs	45-64yrs	65-74yrs	75-84yrs	85+yrs
**4 weekly**	1	1.12	1.11	1.14	0.00	1.69	2.25	2.08	1.68	1.66	0.00	0.62	0.00	0.00	1.77	1.05	3.36	1.73	1.00	0.31	0.00	1.01
	2	1.27	1.27	1.27	0.00	0.00	0.83	4.13	0.46	0.83	0.43	0.00	4.73	0.00	0.00	1.41	3.99	1.12	1.07	1.32	1.43	1.08
	3	0.80	0.72	0.89	0.00	1.76	1.53	0.85	0.89	0.56	0.00	0.89	0.00	0.00	1.04	0.93	2.11	0.91	1.12	0.40	1.45	0.00
	4	1.13	1.12	1.14	0.00	1.04	0.87	2.33	1.65	0.70	0.44	0.00	3.01	0.00	0.00	0.49	3.72	1.78	0.91	0.45	0.72	2.21
	5	1.25	0.95	1.55	0.00	1.84	1.72	3.40	1.05	0.58	0.00	0.00	0.00	0.00	2.21	2.66	2.32	1.62	2.07	1.58	1.44	0.00
	6	1.54	1.62	1.46	0.00	2.93	0.46	3.02	1.65	0.36	0.31	1.87	3.94	0.00	0.80	2.36	2.88	1.78	1.44	0.28	2.38	1.22
	7	2.28	2.23	2.33	0.00	3.13	5.55	4.04	1.63	1.60	2.34	1.77	0.00	0.00	0.98	4.81	6.96	2.28	1.78	1.46	1.50	1.16
	8	1.28	1.44	1.12	0.00	1.14	1.97	3.95	1.16	0.72	0.42	1.73	1.87	0.00	1.23	1.95	4.16	1.38	0.61	0.27	0.46	0.00
	9	1.57	1.89	1.25	0.00	4.62	2.41	4.92	1.61	1.06	2.42	0.00	0.00	0.00	1.99	1.24	2.84	3.03	1.32	0.31	0.52	0.00
	10	2.51	2.46	2.56	4.96	2.94	2.50	6.49	1.75	1.82	1.69	0.00	0.00	5.21	3.55	3.50	4.45	2.64	2.04	0.45	0.00	1.22
	11	1.52	1.36	1.68	0.00	1.03	0.37	3.71	1.79	1.09	3.58	0.63	0.00	0.00	1.99	2.39	5.18	2.40	1.30	0.78	0.00	1.06
	12	1.66	1.57	1.74	0.00	2.48	2.08	3.45	0.74	0.62	0.00	0.00	4.74	3.88	1.85	1.58	3.76	1.85	1.96	0.81	0.00	0.00
	13	1.79	1.62	1.96	0.00	1.33	1.61	4.99	1.13	0.70	0.93	1.76	2.15	0.00	2.34	0.85	3.27	1.31	1.26	0.65	3.15	4.86
**Quarter**	1	1.07	1.04	1.10	0.00	1.19	1.59	2.33	1.06	1.07	0.13	0.51	1.46	0.00	1.00	1.12	3.17	1.29	1.06	0.65	0.89	0.72
	2	1.33	1.30	1.37	0.00	2.07	1.30	3.11	1.34	0.59	0.56	0.58	2.14	0.00	0.93	1.70	3.25	1.82	1.36	0.80	1.40	1.06
	3	1.88	1.98	1.78	1.53	2.69	3.24	4.45	1.61	1.14	1.55	1.08	0.57	1.60	1.53	2.82	4.30	2.19	1.50	0.54	0.76	0.73
	4	1.74	1.60	1.88	0.00	2.01	1.36	4.50	1.31	1.04	1.50	0.68	1.97	1.11	2.56	2.04	4.29	2.01	1.54	0.77	0.90	1.69
**Year**	15/16	1.51	1.48	1.54	0.37	1.99	1.86	3.61	1.33	0.96	0.95	0.71	1.54	0.69	1.53	1.92	3.76	1.83	1.37	0.69	0.98	1.06
**Episodes**	F/N	1002.00	461.00	541.00	1.00	28.00	62.00	150.00	116.00	69.00	20.00	8.00	7.00	2.00	21.00	62.00	150.00	159.00	98.00	21.00	17.00	11.00

Mean weekly incidence rate per 100,000 Persons.

Scabies (ICD10: B86)

			All ages			Male								Female							
			Male & Female	Male	Female	<1yr	1-4yrs	5-14yrs	15-24yrs	25-44yrs	45-64yrs	65-74yrs	75-84yrs	85+yrs	<1yr	1-4yrs	5-14yrs	15-24yrs	25-44yrs	45-64yrs	65-74yrs	75-84yrs	85+yrs
**North**	Quarter	1	0.70	0.79	0.61	0.00	0.00	0.69	3.90	1.74	0.82	0.00	0.00	0.00	0.00	0.00	1.26	2.40	0.88	0.97	0.00	0.00	0.00
		2	1.46	1.58	1.33	0.00	2.05	0.82	2.76	1.18	1.31	0.38	0.00	5.75	0.00	0.99	1.23	4.76	1.79	1.39	0.69	1.16	0.00
		3	2.13	1.87	2.39	0.00	1.91	2.76	6.41	2.04	0.61	0.75	0.00	2.30	6.42	0.00	3.60	4.62	2.15	1.35	2.17	1.20	0.00
		4	1.91	1.69	2.14	0.00	2.38	1.65	7.95	1.35	0.41	0.74	0.72	0.00	0.00	1.71	2.80	7.90	1.84	1.52	0.67	0.51	2.29
	Year	15/16	1.56	1.49	1.63	0.00	1.60	1.48	5.30	1.58	0.78	0.47	0.19	1.97	1.57	0.69	2.23	4.98	1.67	1.31	0.88	0.71	0.60
	Episodes	F/N	346.00	161.00	185.00	0.00	7.00	15.00	74.00	38.00	18.00	5.00	1.00	3.00	1.00	3.00	21.00	71.00	40.00	32.00	10.00	5.00	2.00
**South**	Quarter	1	1.13	1.06	1.20	0.00	1.51	2.19	3.22	1.00	1.11	0.52	0.00	0.00	0.00	1.61	1.60	1.84	1.47	0.89	0.50	0.00	2.89
		2	1.01	0.70	1.32	0.00	1.28	0.50	2.87	0.67	0.41	0.54	0.00	0.00	0.00	0.00	2.09	3.07	2.55	0.64	0.00	0.82	2.72
		3	1.27	1.28	1.25	0.00	1.76	1.93	5.24	1.10	0.41	1.09	0.00	0.00	0.00	0.00	0.51	4.40	1.47	1.15	0.00	0.81	2.92
		4	1.81	2.00	1.62	0.00	3.48	2.12	5.57	1.73	0.75	1.01	0.90	2.45	0.00	2.25	1.86	4.14	2.21	1.70	0.42	0.70	1.31
	Year	15/16	1.31	1.27	1.35	0.00	2.03	1.69	4.25	1.14	0.67	0.79	0.24	0.65	0.00	0.99	1.52	3.38	1.93	1.10	0.23	0.59	2.44
	Episodes	F/N	216.00	103.00	113.00	0.00	6.00	14.00	41.00	21.00	13.00	6.00	1.00	1.00	0.00	3.00	12.00	31.00	34.00	21.00	2.00	3.00	7.00
**London**	Quarter	1	1.37	1.31	1.44	0.00	0.00	2.57	1.29	0.86	1.28	0.00	0.00	5.82	0.00	1.28	1.20	4.99	1.17	1.37	1.14	1.76	0.00
		2	1.54	1.79	1.30	0.00	2.33	2.88	5.76	1.56	0.00	1.30	2.31	0.00	0.00	0.00	2.38	1.17	2.23	2.75	1.37	1.77	0.00
		3	2.59	3.38	1.79	6.11	4.13	5.01	2.62	1.91	2.59	3.78	4.31	0.00	0.00	3.08	4.88	3.83	2.22	2.10	0.00	0.00	0.00
		4	2.02	1.90	2.15	0.00	0.86	1.68	1.81	1.38	1.70	4.27	0.00	5.41	4.44	3.50	0.87	0.41	2.27	2.29	0.93	1.47	3.16
	Year	15/16	1.89	2.09	1.68	1.50	1.81	3.01	2.85	1.43	1.40	2.38	1.62	2.86	1.17	1.99	2.30	2.56	1.98	2.13	0.86	1.25	0.84
	Episodes	F/N	258.00	122.00	136.00	1.00	7.00	23.00	20.00	38.00	20.00	8.00	3.00	2.00	1.00	8.00	17.00	19.00	56.00	28.00	3.00	3.00	1.00
**Midlands And East**	Quarter	1	1.07	0.98	1.15	0.00	3.26	0.91	0.92	0.62	1.06	0.00	2.04	0.00	0.00	1.11	0.43	3.44	1.64	1.00	0.97	1.79	0.00
		2	1.32	1.12	1.52	0.00	2.63	1.00	1.07	1.93	0.65	0.00	0.00	2.81	0.00	2.72	1.09	3.99	0.70	0.67	1.13	1.83	1.50
		3	1.54	1.41	1.67	0.00	2.94	3.27	3.51	1.38	0.95	0.60	0.00	0.00	0.00	3.03	2.29	4.36	2.94	1.40	0.00	1.03	0.00
		4	1.21	0.80	1.61	0.00	1.32	0.00	2.65	0.79	1.30	0.00	1.11	0.00	0.00	2.81	2.65	4.70	1.73	0.67	1.05	0.93	0.00
	Year	15/16	1.28	1.07	1.49	0.00	2.51	1.27	2.05	1.17	1.00	0.15	0.79	0.69	0.00	2.42	1.64	4.14	1.75	0.93	0.79	1.39	0.37
	Episodes	F/N	182.00	75.00	107.00	0.00	8.00	10.00	15.00	19.00	18.00	1.00	3.00	1.00	0.00	7.00	12.00	29.00	29.00	17.00	6.00	6.00	1.00

Mean weekly incidence rate per 100,000 Persons.

Strep Sore Throat, Scarlatina and Peritonsillar Abscess (ICD10: A38,J020,J36)

		All ages			Male									Female								
		Male & Female	Male	Female	<1yr	1-4yrs	5-14yrs	15-24yrs	25-44yrs	45-64yrs	65-74yrs	75-84yrs	85+yrs	<1yr	1-4yrs	5-14yrs	15-24yrs	25-44yrs	45-64yrs	65-74yrs	75-84yrs	85+yrs
**4 weekly**	1	2.33	2.48	2.17	0.00	13.20	5.06	1.26	2.06	0.77	0.00	0.00	0.00	0.00	9.73	9.06	0.00	0.00	0.74	0.00	0.00	0.00
	2	2.14	1.76	2.52	0.00	11.77	1.50	1.26	0.96	0.38	0.00	0.00	0.00	0.00	11.28	7.01	2.80	0.98	0.64	0.00	0.00	0.00
	3	1.39	1.47	1.31	0.00	7.94	3.02	1.00	0.55	0.72	0.00	0.00	0.00	0.00	6.19	3.35	0.80	0.74	0.29	0.46	0.00	0.00
	4	0.55	0.35	0.76	0.00	1.11	0.00	0.95	0.90	0.22	0.00	0.00	0.00	0.00	3.14	0.85	0.37	1.15	0.46	0.00	0.84	0.00
	5	0.70	0.71	0.68	0.00	3.78	2.51	0.00	0.00	0.14	0.00	0.00	0.00	0.00	3.13	1.07	1.21	0.66	0.00	0.00	0.00	0.00
	6	1.37	1.62	1.12	0.00	7.68	4.89	0.42	0.56	0.29	0.72	0.00	0.00	0.00	4.00	1.78	1.78	1.03	0.59	0.30	0.64	0.00
	7	1.46	1.44	1.48	0.00	9.40	2.56	0.84	0.18	0.00	0.00	0.00	0.00	0.00	9.01	1.74	0.35	2.07	0.17	0.00	0.00	0.00
	8	2.00	2.03	1.98	0.00	11.33	4.94	0.72	0.45	0.41	0.42	0.00	0.00	0.00	9.78	4.64	1.65	1.28	0.46	0.00	0.00	0.00
	9	1.81	1.77	1.84	0.00	8.76	3.10	1.97	0.71	0.25	0.00	1.15	0.00	0.00	12.26	2.06	1.32	0.81	0.13	0.00	0.00	0.00
	10	4.00	4.36	3.64	7.26	20.64	8.32	1.43	0.72	0.00	0.00	0.83	0.00	0.00	22.81	5.61	2.34	0.76	0.00	1.24	0.00	0.00
	11	6.33	6.60	6.06	0.00	43.82	11.80	1.84	0.72	0.30	0.94	0.00	0.00	4.64	33.53	12.31	1.94	1.27	0.35	0.46	0.00	0.00
	12	7.00	6.77	7.22	8.18	36.71	13.55	0.93	1.24	0.34	0.00	0.00	0.00	6.46	34.16	20.38	1.46	1.61	0.68	0.27	0.00	0.00
	13	3.66	3.18	4.14	0.00	22.25	4.63	0.78	0.62	0.32	0.00	0.00	0.00	8.40	20.43	6.11	0.98	0.71	0.16	0.49	0.00	0.00
**Quarter**	1	1.98	1.95	2.01	0.00	11.14	3.34	1.18	1.26	0.63	0.00	0.00	0.00	0.00	9.11	6.67	1.11	0.53	0.57	0.14	0.00	0.00
	2	0.85	0.87	0.83	0.00	3.87	2.51	0.56	0.45	0.20	0.22	0.00	0.00	0.00	3.16	1.39	1.03	0.97	0.32	0.09	0.46	0.00
	3	2.07	2.10	2.05	0.00	12.26	3.97	1.16	0.56	0.20	0.13	0.61	0.00	0.00	12.54	2.76	1.41	1.21	0.23	0.29	0.00	0.00
	4	5.54	5.48	5.59	4.41	32.31	10.06	1.23	0.81	0.27	0.27	0.00	0.00	5.57	28.92	12.31	1.56	1.21	0.34	0.43	0.00	0.00
**Year**	15/16	2.67	2.65	2.68	1.16	15.22	5.07	1.04	0.77	0.33	0.16	0.15	0.00	1.47	13.73	5.91	1.28	0.99	0.37	0.24	0.11	0.00
**Episodes**	F/N	1092.00	532.00	560.00	3.00	218.00	180.00	39.00	61.00	25.00	4.00	2.00	0.00	4.00	188.00	199.00	51.00	81.00	27.00	8.00	2.00	0.00

Mean weekly incidence rate per 100,000 Persons.

Strep Sore Throat, Scarlatina and Peritonsillar Abscess (ICD10: A38,J020,J36)

			All ages			Male									Female								
			Male & Female	Male	Female	<1yr	1-4yrs	5-14yrs	15-24yrs	25-44yrs	45-64yrs	65-74yrs	75-84yrs	85+yrs	<1yr	1-4yrs	5-14yrs	15-24yrs	25-44yrs	45-64yrs	65-74yrs	75-84yrs	85+yrs
**North**	Quarter	1	2.53	3.01	2.05	0.00	20.22	2.53	1.73	1.84	0.77	0.00	0.00	0.00	0.00	9.43	7.05	0.59	0.29	0.49	0.57	0.00	0.00
		2	1.01	1.19	0.83	0.00	6.35	2.06	1.03	0.30	0.59	0.37	0.00	0.00	0.00	2.28	0.81	1.23	1.42	0.30	0.37	1.04	0.00
		3	2.03	2.19	1.88	0.00	13.00	4.84	1.24	0.32	0.31	0.00	0.00	0.00	0.00	9.18	5.63	1.02	0.77	0.30	0.00	0.00	0.00
		4	6.00	6.20	5.79	9.34	28.82	14.86	1.18	1.36	0.27	0.00	0.00	0.00	5.31	27.12	15.38	2.25	1.15	0.27	0.65	0.00	0.00
	Year	15/16	2.95	3.21	2.70	2.47	17.32	6.24	1.29	0.96	0.48	0.09	0.00	0.00	1.40	12.29	7.37	1.29	0.91	0.34	0.40	0.25	0.00
	Episodes	F/N	351.00	179.00	172.00	2.00	65.00	65.00	16.00	20.00	10.00	1.00	0.00	0.00	1.00	47.00	71.00	19.00	21.00	8.00	4.00	1.00	0.00
**South**	Quarter	1	2.19	2.13	2.25	0.00	10.69	3.84	0.85	2.44	1.37	0.00	0.00	0.00	0.00	8.88	7.22	2.77	0.91	0.49	0.00	0.00	0.00
		2	1.14	1.27	1.00	0.00	6.63	3.42	0.00	0.67	0.21	0.52	0.00	0.00	0.00	2.84	2.01	1.77	1.16	0.41	0.00	0.79	0.00
		3	2.10	1.89	2.31	0.00	11.35	1.61	0.40	0.68	0.00	0.51	2.44	0.00	0.00	15.78	1.71	2.24	0.67	0.41	0.00	0.00	0.00
		4	6.51	6.64	6.38	8.30	36.72	11.17	1.84	1.12	0.58	0.00	0.00	0.00	5.56	31.15	15.33	2.82	2.01	0.58	0.00	0.00	0.00
	Year	15/16	3.05	3.05	3.05	2.19	16.73	5.13	0.79	1.23	0.54	0.25	0.60	0.00	1.47	14.97	6.73	2.41	1.20	0.48	0.00	0.19	0.00
	Episodes	F/N	296.00	143.00	153.00	1.00	55.00	43.00	8.00	22.00	10.00	2.00	2.00	0.00	1.00	45.00	53.00	22.00	22.00	9.00	0.00	1.00	0.00
**London**	Quarter	1	1.28	0.82	1.75	0.00	3.77	2.62	0.67	0.34	0.00	0.00	0.00	0.00	0.00	7.88	6.08	0.59	0.48	0.69	0.00	0.00	0.00
		2	0.74	0.75	0.74	0.00	1.14	4.58	0.65	0.35	0.00	0.00	0.00	0.00	0.00	4.87	0.58	0.57	0.33	0.35	0.00	0.00	0.00
		3	2.21	2.22	2.19	0.00	13.13	5.60	0.60	0.41	0.27	0.00	0.00	0.00	0.00	16.02	1.98	0.48	1.26	0.00	0.00	0.00	0.00
		4	4.67	4.90	4.45	0.00	34.04	7.09	1.36	0.50	0.00	1.07	0.00	0.00	4.04	25.28	9.46	0.00	0.72	0.51	0.00	0.00	0.00
	Year	15/16	2.27	2.22	2.32	0.00	13.42	5.01	0.83	0.40	0.07	0.28	0.00	0.00	1.07	13.73	4.62	0.40	0.70	0.39	0.00	0.00	0.00
	Episodes	F/N	240.00	118.00	122.00	0.00	58.00	41.00	6.00	11.00	1.00	1.00	0.00	0.00	1.00	56.00	37.00	3.00	20.00	5.00	0.00	0.00	0.00
**Midlands And East**	Quarter	1	1.92	1.83	2.02	0.00	9.89	4.36	1.47	0.40	0.39	0.00	0.00	0.00	0.00	10.27	6.34	0.49	0.44	0.60	0.00	0.00	0.00
		2	0.50	0.26	0.73	0.00	1.35	0.00	0.55	0.47	0.00	0.00	0.00	0.00	0.00	2.65	2.16	0.57	0.98	0.22	0.00	0.00	0.00
		3	1.96	2.10	1.82	0.00	11.54	3.84	2.41	0.83	0.24	0.00	0.00	0.00	0.00	9.20	1.73	1.89	2.15	0.23	1.15	0.00	0.00
		4	4.98	4.20	5.76	0.00	29.65	7.11	0.54	0.25	0.24	0.00	0.00	0.00	7.38	32.12	9.07	1.18	0.98	0.00	1.08	0.00	0.00
	Year	15/16	2.39	2.14	2.64	0.00	13.42	3.89	1.23	0.48	0.22	0.00	0.00	0.00	1.95	13.91	4.90	1.03	1.13	0.26	0.57	0.00	0.00
	Episodes	F/N	205.00	92.00	113.00	0.00	40.00	31.00	9.00	8.00	4.00	0.00	0.00	0.00	1.00	40.00	38.00	7.00	18.00	5.00	4.00	0.00	0.00

Mean weekly incidence rate per 100,000 Persons.

Symptoms Involving Nervous and Musculoskeletal (ICD10: R25-R29)

		All ages			Male								Female							
		Male & Female	Male	Female	<1yr	1-4yrs	5-14yrs	15-24yrs	25-44yrs	45-64yrs	65-74yrs	75-84yrs	85+yrs	<1yr	1-4yrs	5-14yrs	15-24yrs	25-44yrs	45-64yrs	65-74yrs	75-84yrs	85+yrs
**4 weekly**	1	6.00	5.71	6.28	0.00	4.94	4.10	2.94	4.03	4.23	10.29	11.60	9.30	0.00	8.02	3.58	4.48	5.66	6.49	6.95	13.91	7.46
	2	5.41	5.21	5.60	0.00	5.71	3.17	4.79	4.44	6.25	5.28	7.81	9.48	0.00	8.44	2.40	4.48	5.12	8.08	5.73	11.13	5.04
	3	5.90	4.41	7.40	0.00	4.38	2.74	1.89	3.52	6.47	8.68	7.34	4.69	0.00	9.19	5.03	2.44	4.14	8.28	9.15	14.21	14.13
	4	5.21	5.14	5.28	0.00	7.64	2.51	3.81	3.82	4.76	5.82	7.85	10.08	0.00	4.86	3.58	6.20	4.43	3.73	7.62	13.65	3.47
	5	5.20	4.79	5.60	0.00	5.92	4.97	2.95	4.04	5.70	3.45	11.96	4.15	0.00	8.07	5.40	2.96	6.27	6.53	1.61	15.27	4.33
	6	7.45	6.98	7.92	0.00	7.38	5.24	3.35	3.53	7.25	6.29	8.53	21.22	0.00	9.14	3.88	2.31	5.16	7.77	11.05	5.05	26.93
	7	5.79	5.25	6.32	0.00	8.99	3.91	5.52	3.35	6.46	5.63	10.61	2.82	0.00	3.99	5.45	5.36	6.14	8.36	8.56	11.12	7.93
	8	5.57	4.57	6.57	0.00	6.36	4.54	2.65	4.29	5.20	7.47	7.03	3.56	7.29	6.44	3.01	4.38	4.98	7.82	7.32	8.27	9.58
	9	5.07	5.04	5.10	0.00	6.38	3.46	1.43	4.35	4.65	7.89	7.23	10.01	0.00	4.54	2.11	5.08	5.11	6.81	7.02	7.93	7.25
	10	5.84	6.21	5.46	0.00	8.52	3.51	2.99	3.96	3.20	9.07	12.94	11.71	0.00	7.44	4.02	3.73	5.88	7.93	9.91	2.46	7.79
	11	7.39	7.82	6.97	0.00	9.47	2.58	5.20	4.23	6.28	7.77	16.66	18.19	0.00	8.66	7.11	3.91	5.11	8.33	9.99	6.46	13.12
	12	4.55	3.87	5.23	0.00	3.36	3.66	2.47	3.65	5.59	3.89	8.39	3.80	0.00	9.13	4.30	0.71	4.93	6.62	3.95	12.26	5.15
	13	6.07	5.46	6.68	0.00	8.31	5.63	1.62	2.76	4.79	7.93	3.36	14.74	0.00	10.61	3.93	2.83	6.61	7.52	8.35	14.99	5.26
**Quarter**	1	5.79	5.16	6.42	0.00	5.00	3.40	3.18	4.00	5.54	8.25	9.12	7.94	0.00	8.51	3.66	3.85	5.03	7.53	7.25	13.14	8.77
	2	5.97	5.67	6.27	0.00	7.00	4.44	3.55	3.85	5.94	5.46	9.87	10.91	0.00	7.14	4.58	3.96	5.14	6.15	7.12	11.01	11.30
	3	5.46	4.94	5.98	0.00	7.46	3.78	3.17	4.04	4.97	7.53	8.50	5.04	2.24	4.74	3.53	4.63	5.61	7.70	8.08	8.21	9.09
	4	5.99	5.90	6.09	0.00	7.24	3.79	2.91	3.52	5.26	6.57	9.96	13.84	0.00	9.79	4.69	2.72	5.62	7.55	7.43	10.00	7.01
**Year**	15/16	5.81	5.43	6.19	0.00	6.69	3.85	3.20	3.85	5.43	6.94	9.37	9.52	0.55	7.59	4.13	3.77	5.36	7.24	7.47	10.58	9.00
**Episodes**	F/N	3263.00	1441.00	1822.00	0.00	94.00	130.00	120.00	324.00	406.00	191.00	128.00	48.00	1.00	102.00	135.00	135.00	465.00	520.00	201.00	181.00	82.00

Mean weekly incidence rate per 100,000 Persons.

Symptoms Involving Nervous and Musculoskeletal (ICD10: R25-R29)

			All ages			Male								Female							
			Male & Female	Male	Female	<1yr	1-4yrs	5-14yrs	15-24yrs	25-44yrs	45-64yrs	65-74yrs	75-84yrs	85+yrs	<1yr	1-4yrs	5-14yrs	15-24yrs	25-44yrs	45-64yrs	65-74yrs	75-84yrs	85+yrs
**North**	Quarter	1	6.72	6.31	7.12	0.00	8.45	4.29	2.81	6.05	8.39	7.41	9.36	10.00	0.00	5.12	2.53	5.91	5.94	10.01	7.43	9.32	17.84
		2	7.00	6.23	7.77	0.00	8.90	6.21	2.40	4.16	7.05	7.50	8.79	11.05	0.00	9.93	1.63	5.88	7.17	5.83	7.03	18.38	14.12
		3	5.78	6.23	5.33	0.00	7.01	2.83	2.88	5.42	5.99	9.40	8.76	13.78	0.00	2.00	4.75	2.67	5.92	6.40	8.01	12.68	5.53
		4	6.24	6.55	5.93	0.00	8.77	2.94	3.75	3.45	7.01	8.04	10.22	14.82	0.00	13.10	3.85	2.87	6.05	8.19	4.57	5.98	8.77
	Year	15/16	6.43	6.33	6.53	0.00	8.29	4.05	2.98	4.74	7.11	8.09	9.30	12.46	0.00	7.64	3.20	4.31	6.27	7.62	6.72	11.49	11.51
	Episodes	F/N	1,113.00	523.00	590.00	0.00	31.00	38.00	40.00	107.00	167.00	75.00	45.00	20.00	0.00	28.00	31.00	48.00	139.00	176.00	66.00	67.00	35.00
**South**	Quarter	1	4.96	4.68	5.25	0.00	2.86	4.31	4.44	5.31	4.94	6.95	5.46	7.87	0.00	10.07	2.76	3.81	5.92	7.79	3.71	7.57	5.57
		2	4.74	4.56	4.91	0.00	9.08	3.88	3.28	3.39	3.99	4.78	9.94	2.71	0.00	6.90	6.16	4.86	4.65	4.21	6.14	5.77	5.54
		3	4.14	3.73	4.54	0.00	6.35	3.41	2.09	2.86	3.24	5.45	7.29	2.92	0.00	5.34	3.06	2.71	6.47	5.64	6.58	6.59	4.51
		4	5.56	6.04	5.07	0.00	6.79	3.91	3.02	4.60	4.57	9.55	9.41	12.48	0.00	8.18	4.24	1.69	6.38	6.28	7.90	10.99	0.00
	Year	15/16	4.86	4.78	4.95	0.00	6.28	3.88	3.21	4.05	4.19	6.74	8.05	6.61	0.00	7.63	4.06	3.24	5.86	5.99	6.12	7.79	3.83
	Episodes	F/N	732.00	329.00	403.00	0.00	20.00	32.00	31.00	73.00	80.00	51.00	32.00	10.00	0.00	23.00	32.00	29.00	105.00	115.00	49.00	39.00	11.00
**London**	Quarter	1	6.62	6.29	6.95	0.00	6.29	3.49	2.61	2.73	5.79	10.44	13.79	11.47	0.00	8.58	3.70	3.72	5.59	7.55	8.63	21.29	3.45
		2	7.08	7.26	6.91	0.00	4.82	4.73	3.79	4.21	8.98	3.92	16.59	18.28	0.00	3.53	4.79	2.35	4.27	9.92	11.56	11.12	14.60
		3	7.78	5.76	9.79	0.00	11.00	6.79	4.26	4.00	6.13	8.54	11.13	0.00	8.98	8.59	3.44	8.87	6.90	12.47	11.44	9.76	17.68
		4	7.32	6.16	8.47	0.00	6.85	6.22	2.70	3.50	5.34	5.34	9.55	15.95	0.00	9.55	6.90	4.02	5.83	11.45	12.98	13.18	12.36
	Year	15/16	7.20	6.36	8.04	0.00	7.23	5.33	3.33	3.61	6.54	7.03	12.70	11.51	2.20	7.60	4.75	4.73	5.65	10.37	11.19	13.82	12.03
	Episodes	F/N	824.00	334.00	490.00	0.00	28.00	43.00	24.00	96.00	89.00	23.00	23.00	8.00	1.00	28.00	37.00	38.00	160.00	138.00	42.00	32.00	14.00
**Midlands And East**	Quarter	1	4.85	3.36	6.35	0.00	2.41	1.48	2.87	1.91	3.06	8.19	7.88	2.41	0.00	10.28	5.64	1.97	2.65	4.76	9.25	14.39	8.20
		2	5.06	4.64	5.47	0.00	5.23	2.96	4.73	3.66	3.74	5.64	4.19	11.59	0.00	8.19	5.75	2.75	4.46	4.62	3.73	8.79	10.96
		3	4.15	4.04	4.26	0.00	5.48	2.09	3.42	3.88	4.52	6.73	6.81	3.47	0.00	3.04	2.88	4.29	3.17	6.27	6.29	3.80	8.65
		4	4.86	4.84	4.88	0.00	6.54	2.08	2.16	2.53	4.14	3.33	10.65	12.11	0.00	8.33	3.78	2.31	4.22	4.28	4.26	9.83	6.92
	Year	15/16	4.73	4.23	5.24	0.00	4.94	2.15	3.27	2.98	3.87	5.92	7.44	7.49	0.00	7.48	4.50	2.82	3.64	4.97	5.85	9.21	8.65
	Episodes	F/N	594.00	255.00	339.00	0.00	15.00	17.00	25.00	48.00	70.00	42.00	28.00	10.00	0.00	23.00	35.00	20.00	61.00	91.00	44.00	43.00	22.00

Mean weekly incidence rate per 100,000 Persons.

Symptoms involving Respiratory & Chest (ICD10: R05-R07,R09)

		All ages			Male								Female							
		Male & Female	Male	Female	<1yr	1-4yrs	5-14yrs	15-24yrs	25-44yrs	45-64yrs	65-74yrs	75-84yrs	85+yrs	<1yr	1-4yrs	5-14yrs	15-24yrs	25-44yrs	45-64yrs	65-74yrs	75-84yrs	85+yrs
**4 weekly**	1	24.61	24.05	25.18	0.00	32.60	14.64	14.14	17.37	21.35	29.81	41.65	44.87	0.00	29.81	10.02	19.40	24.78	26.55	38.82	35.62	41.60
	2	41.84	23.57	60.11	0.00	17.38	17.90	15.95	17.09	24.94	34.82	32.36	51.74	347.22	19.98	9.94	18.89	19.25	19.96	25.87	37.45	42.46
	3	26.82	20.17	33.46	0.00	26.52	10.07	12.26	18.38	22.65	24.92	30.76	35.99	107.76	24.92	9.28	19.40	15.84	23.16	29.34	35.90	35.58
	4	23.46	20.22	26.70	0.00	20.22	11.73	12.81	14.05	17.08	28.47	35.57	42.09	63.78	15.15	8.56	19.46	18.50	25.12	26.34	29.43	33.97
	5	25.93	28.91	22.95	34.94	30.01	8.67	12.94	14.38	20.75	33.82	32.11	72.58	20.16	19.50	11.76	14.45	19.85	22.79	27.53	40.95	29.59
	6	25.86	26.95	24.77	0.00	48.33	14.29	15.18	18.88	22.64	30.23	37.30	55.69	0.00	36.22	12.73	17.97	24.97	29.76	26.55	29.62	45.14
	7	32.37	31.26	33.47	66.85	52.96	11.12	13.93	17.44	24.17	23.06	32.79	39.04	46.06	40.83	11.15	22.21	17.63	28.86	36.64	47.07	50.76
	8	33.26	32.15	34.37	41.36	49.09	16.12	13.45	20.59	23.22	29.81	34.24	61.45	73.47	51.11	15.09	19.42	20.78	30.44	31.28	37.37	30.38
	9	29.88	31.58	28.18	93.14	26.24	15.68	9.13	21.77	25.07	27.04	33.38	32.75	23.97	25.71	15.08	15.01	19.39	31.98	34.86	37.02	50.60
	10	36.54	35.29	37.78	55.74	36.03	19.69	16.47	22.37	27.76	34.72	43.46	61.40	82.35	24.06	16.76	25.22	25.94	33.11	37.28	47.21	48.11
	11	32.80	33.02	32.59	59.78	32.05	19.27	19.42	19.29	27.53	33.33	57.34	29.13	39.83	28.55	14.15	22.31	26.95	30.66	42.90	44.46	43.48
	12	28.36	27.50	29.23	44.33	33.54	12.57	9.87	15.75	22.42	27.00	31.00	50.97	32.47	28.63	17.72	19.02	22.62	28.51	42.70	43.31	28.10
	13	28.10	28.36	27.84	57.25	16.41	15.42	12.63	14.28	25.10	26.37	38.27	49.50	31.11	17.94	11.74	19.25	21.82	29.15	30.47	42.83	46.26
**Quarter**	1	30.59	22.71	38.48	0.00	26.04	14.24	14.12	17.59	22.85	29.85	35.44	44.25	139.99	25.28	9.77	19.24	20.33	23.48	31.92	36.27	40.01
	2	25.64	26.17	25.11	16.76	36.09	11.34	14.05	16.25	20.71	30.16	35.36	54.81	28.61	25.66	11.55	18.19	20.67	25.52	27.77	33.32	34.68
	3	32.77	32.35	33.19	64.92	40.45	15.55	12.37	20.42	24.27	28.18	34.82	50.15	53.83	35.32	14.69	18.96	21.18	31.07	33.81	42.15	47.74
	4	30.49	30.01	30.97	53.69	27.47	16.33	14.28	17.03	25.59	29.79	42.11	43.85	41.51	25.57	14.13	21.02	23.55	30.41	38.98	44.00	39.58
**Year**	15/16	29.89	27.85	31.92	34.22	32.42	14.40	13.72	17.81	23.40	29.50	37.03	48.18	65.52	27.91	12.56	19.39	21.47	27.67	33.23	39.03	40.49
**Episodes**	F/N	13782.00	6369.00	7413.00	71.00	452.00	484.00	517.00	1493.00	1751.00	814.00	532.00	255.00	59.00	381.00	404.00	709.00	1812.00	2053.00	927.00	690.00	378.00

Mean weekly incidence rate per 100,000 Persons.

Symptoms involving Respiratory and Chest (ICD10: R05 - R07; R09)

			All ages			Male									Female								
			Male & Female	Male	Female	<1yr	1-4yrs	5-14yrs	15-24yrs	25-44yrs	45-64yrs	65-74yrs	75-84yrs	85+yrs	<1yr	1-4yrs	5-14yrs	15-24yrs	25-44yrs	45-64yrs	65-74yrs	75-84yrs	85+yrs
**North**	Quarter	1	344.13	340.94	347.32	389.91	983.55	331.64	184.13	164.38	139.17	223.52	289.09	363.06	192.31	754.42	347.29	328.28	320.95	246.77	250.65	340.23	344.94
		2	383.35	368.01	398.70	843.17	942.86	202.82	134.54	124.81	141.26	229.61	322.28	370.70	612.53	950.36	228.65	278.07	251.85	246.34	267.87	319.82	432.83
		3	716.84	721.55	712.13	2,496.59	1,613.50	376.28	174.42	179.71	235.16	349.31	480.36	588.63	1,795.51	1,535.81	391.32	316.13	377.10	401.14	436.97	508.47	646.74
		4	650.09	638.71	661.47	2,064.27	1,309.71	425.76	206.29	214.53	234.24	354.26	400.75	538.61	1,684.95	1,170.27	459.52	333.27	418.98	394.24	438.68	473.50	579.81
	Year	15/16	525.99	519.59	532.39	1,460.10	1,214.24	335.85	175.44	171.68	188.34	290.40	373.64	466.63	1,082.90	1,103.99	358.63	314.30	343.67	323.49	350.24	411.70	502.57
	Episodes	F/N	61,262.00	25,418.00	35,844.00	825.00	4,808.00	3,285.00	2,309.00	4,120.00	4,641.00	2,781.00	1,874.00	775.00	613.00	4,214.00	3,402.00	4,188.00	7,890.00	7,878.00	3,582.00	2,513.00	1,564.00
**South**	Quarter	1	267.23	257.49	276.97	76.16	766.81	292.09	161.91	145.35	123.17	181.74	241.48	328.73	160.26	633.94	255.99	264.60	250.47	190.30	200.60	217.25	319.30
		2	382.67	382.37	382.98	1,086.97	944.96	201.23	134.37	116.36	118.88	193.03	264.11	381.41	846.03	819.93	206.75	245.53	238.34	195.25	229.00	270.08	395.87
		3	723.07	714.62	731.52	2,676.00	1,634.04	336.10	191.77	177.03	202.46	299.35	396.69	518.18	2,246.60	1,532.73	372.84	368.12	348.70	324.56	380.72	410.28	599.14
		4	617.70	611.63	623.77	1,876.42	1,313.27	419.15	225.43	212.06	228.01	343.76	401.92	484.66	1,422.43	1,217.21	434.09	383.70	429.25	383.02	409.35	404.28	530.63
	Year	15/16	499.93	493.80	506.07	1,437.33	1,167.57	314.16	179.26	163.63	169.26	256.16	327.48	429.31	1,173.62	1,054.09	319.62	316.77	318.81	275.35	306.89	326.96	462.54
	Episodes	F/N	43,949.00	18,723.00	25,226.00	545.00	3,713.00	2,615.00	1,722.00	2,971.00	3,241.00	1,936.00	1,321.00	659.00	427.00	3,155.00	2,510.00	2,834.00	5,689.00	5,277.00	2,447.00	1,598.00	1,289.00
**London**	Quarter	1	360.81	359.28	362.34	480.77	948.31	429.38	184.57	154.53	151.96	239.56	272.00	372.47	289.79	843.47	394.34	288.22	262.34	246.73	288.19	297.89	350.07
		2	435.80	434.25	437.35	1,089.79	1,141.78	303.18	157.36	124.68	159.16	238.64	327.00	366.64	880.25	1,035.94	283.58	245.41	221.33	253.71	304.07	364.29	347.58
		3	692.82	672.14	713.50	2,024.37	1,681.80	510.41	190.46	160.28	209.58	341.41	476.31	454.66	1,761.41	1,665.60	497.65	275.05	308.83	371.52	441.33	533.47	566.64
		4	630.06	603.73	656.38	1,644.94	1,391.53	540.14	209.56	181.76	229.06	349.75	392.10	494.73	1,460.48	1,294.28	570.88	335.03	350.81	385.74	461.04	515.59	533.57
	Year	15/16	531.76	518.98	544.54	1,316.29	1,292.75	447.56	185.94	155.81	188.23	293.42	367.33	423.50	1,104.82	1,211.42	439.14	286.85	287.05	315.77	375.31	429.47	451.05
	Episodes	F/N	45,984.00	19,494.00	26,490.00	696.00	5,068.00	3,612.00	1,349.00	4,175.00	2,656.00	966.00	681.00	291.00	603.00	4,688.00	3,454.00	2,305.00	8,202.00	4,248.00	1,418.00	1,041.00	531.00
**Midlands And East**	Quarter	1	342.07	269.27	414.86	226.42	650.84	316.61	174.22	144.25	126.46	199.96	275.96	308.75	1,319.83	616.04	261.93	270.65	257.56	193.33	232.65	241.66	340.09
		2	345.66	346.71	344.62	736.46	925.01	202.91	134.98	123.32	129.73	196.94	266.83	404.18	573.14	735.16	195.10	265.60	221.59	215.98	240.21	256.24	398.54
		3	672.81	682.68	662.94	2,104.68	1,571.01	369.05	188.34	188.11	206.56	338.72	419.05	758.63	1,654.37	1,434.96	368.59	356.08	381.85	349.07	384.80	437.83	598.87
		4	587.72	576.52	598.92	1,581.86	1,204.65	416.73	233.02	213.92	234.74	351.31	372.46	580.00	1,252.87	1,100.91	432.75	416.72	451.39	388.17	410.75	437.22	499.46
	Year	15/16	488.96	470.83	507.10	1,170.27	1,090.08	328.03	183.59	168.28	175.51	273.23	334.31	514.15	1,201.05	974.20	316.82	328.95	330.43	288.55	318.87	345.01	460.00
	Episodes	F/N	40,981.00	17,421.00	23,560.00	380.00	3,285.00	2,625.00	1,380.00	2,734.00	3,163.00	1,919.00	1,250.00	685.00	304.00	2,850.00	2,387.00	2,297.00	5,465.00	5,187.00	2,368.00	1,546.00	1,156.00

Mean weekly incidence rate per 100,000 Persons.

Symptoms involving Skin and Oth Integument Tiss (ICD10: R20-R23)

		All ages			Male								Female							
		Male & Female	Male	Female	<1yr	1-4yrs	5-14yrs	15-24yrs	25-44yrs	45-64yrs	65-74yrs	75-84yrs	85+yrs	<1yr	1-4yrs	5-14yrs	15-24yrs	25-44yrs	45-64yrs	65-74yrs	75-84yrs	85+yrs
**4 weekly**	1	58.14	55.03	61.24	0.00	150.42	50.46	17.61	28.79	34.72	48.06	68.86	96.38	0.00	143.52	53.37	44.06	48.38	52.49	52.59	64.79	91.96
	2	111.81	98.72	124.89	377.25	177.88	44.51	21.61	30.05	34.33	53.90	70.49	78.47	568.18	132.40	55.00	46.81	52.88	45.46	54.35	82.44	86.53
	3	88.80	90.02	87.59	283.27	158.26	46.03	29.92	31.01	39.14	57.39	81.71	83.41	172.06	135.61	53.41	47.37	57.02	53.37	70.82	94.17	104.46
	4	74.88	68.85	80.90	204.67	113.52	38.67	23.99	27.87	31.46	42.48	65.73	71.28	196.32	118.53	53.18	41.45	46.93	53.53	59.21	78.57	80.41
	5	67.81	63.96	71.67	149.87	129.51	33.69	20.12	24.07	28.95	39.53	82.75	67.13	142.30	124.10	46.64	37.75	45.85	44.84	47.36	69.06	87.11
	6	70.35	58.94	81.76	97.10	134.48	38.84	19.50	21.95	28.15	42.36	61.67	86.41	212.81	137.86	42.88	40.53	43.82	45.68	55.82	66.43	89.98
	7	70.45	68.26	72.64	169.76	147.34	35.07	22.11	21.23	32.87	35.81	59.16	90.97	191.42	111.33	43.38	42.20	45.69	44.31	46.78	59.41	69.24
	8	68.34	62.82	73.86	145.58	112.11	36.81	26.22	23.84	25.28	38.23	64.84	92.43	213.06	112.54	35.57	44.33	40.79	42.62	45.64	56.04	74.12
	9	63.95	60.83	67.06	211.08	74.87	32.70	19.28	20.80	25.76	40.15	51.78	71.07	165.21	104.58	36.93	34.57	36.71	36.92	46.58	57.74	84.34
	10	76.07	72.94	79.20	217.44	119.66	46.48	25.67	25.78	31.33	44.44	63.11	82.53	203.54	126.14	49.29	44.18	45.42	44.70	45.21	64.54	89.75
	11	79.58	70.07	89.09	205.93	90.94	41.49	21.04	25.51	32.28	45.98	72.39	95.05	269.43	123.94	44.00	43.63	41.54	47.54	45.15	77.76	108.79
	12	74.24	75.84	72.65	254.55	121.42	31.83	23.04	22.95	36.78	45.44	55.65	90.90	176.61	113.50	48.76	36.54	40.27	39.88	54.17	73.24	70.89
	13	75.69	74.14	77.24	248.33	114.08	42.10	22.53	22.04	35.30	49.14	61.81	71.94	214.10	92.63	45.02	37.23	41.29	47.05	54.54	61.99	101.36
**Quarter**	1	84.09	79.24	88.93	203.24	161.28	47.27	22.63	29.86	35.96	52.73	73.31	86.88	227.77	137.66	53.88	45.92	52.42	50.60	58.74	79.26	94.14
	2	70.21	63.21	77.21	149.89	126.86	36.87	21.20	24.44	29.16	40.65	68.68	71.17	178.87	125.90	47.72	39.79	45.20	48.33	51.88	70.37	86.84
	3	69.64	67.02	72.27	191.03	109.98	37.86	24.05	22.68	29.00	38.40	59.98	90.20	193.55	115.04	39.73	40.83	42.52	41.04	47.52	56.61	73.56
	4	76.24	72.40	80.08	227.52	110.92	38.52	21.83	23.68	34.05	47.62	63.07	84.36	221.27	109.17	46.13	40.11	40.92	44.75	50.69	72.02	95.62
**Year**	15/16	75.07	70.50	79.63	193.57	126.95	40.10	22.42	25.14	32.08	44.90	66.20	83.17	205.66	121.70	46.85	41.64	45.18	46.15	52.18	69.61	87.69
**Episodes**	F/N	26947.00	11219.00	15728.00	287.00	1736.00	1329.00	834.00	2127.00	2366.00	1163.00	942.00	435.00	277.00	1621.00	1500.00	1555.00	3873.00	3372.00	1469.00	1220.00	841.00

Mean weekly incidence rate per 100,000 Persons.

Symptoms involving Skin and Oth Integument Tiss (ICD10: R20-R23)

			All ages			Male									Female								
			Male & Female	Male	Female	<1yr	1-4yrs	5-14yrs	15-24yrs	25-44yrs	45-64yrs	65-74yrs	75-84yrs	85+yrs	<1yr	1-4yrs	5-14yrs	15-24yrs	25-44yrs	45-64yrs	65-74yrs	75-84yrs	85+yrs
**North**	Quarter	1	73.61	76.34	70.89	109.89	203.31	58.21	21.40	34.59	35.71	53.61	81.06	89.24	0.00	149.59	59.11	49.56	59.62	53.51	64.79	85.43	116.43
		2	68.59	63.53	73.65	149.01	128.26	36.43	19.62	22.34	28.55	40.28	69.68	77.61	150.20	135.30	46.49	44.01	44.58	48.39	41.86	65.56	86.47
		3	75.60	71.02	80.19	250.51	103.88	34.50	24.58	20.07	30.26	35.90	60.31	79.14	255.75	123.02	44.81	38.48	45.16	42.41	43.27	60.70	68.12
		4	77.39	73.78	80.99	210.76	114.82	39.64	22.20	23.84	34.01	48.81	71.69	98.30	217.82	109.59	45.38	45.03	44.62	47.13	53.68	76.92	88.75
	Year	15/16	73.87	71.22	76.52	180.62	137.14	42.15	21.95	25.18	32.17	44.73	70.70	86.30	157.11	129.00	48.88	44.29	48.42	47.85	50.95	72.24	89.92
	Episodes	F/N	8,307.00	3,440.00	4,867.00	85.00	488.00	383.00	288.00	555.00	761.00	405.00	338.00	137.00	83.00	451.00	440.00	569.00	1,045.00	1,105.00	490.00	421.00	263.00
**South**	Quarter	1	77.67	91.74	63.59	360.22	165.14	30.12	29.20	22.69	31.84	47.62	62.15	76.64	91.58	117.16	36.06	36.10	46.54	40.74	46.47	69.42	88.28
		2	65.97	61.05	70.89	132.37	132.77	33.16	19.45	20.61	28.49	34.60	74.30	73.73	98.17	122.18	44.64	47.09	42.24	46.23	54.57	80.57	102.33
		3	67.92	66.09	69.76	175.59	131.43	31.71	20.97	22.90	30.87	37.67	65.68	77.94	144.16	130.46	37.27	48.48	41.63	43.98	45.55	61.90	74.44
		4	83.24	79.25	87.23	276.95	123.14	33.29	21.72	22.98	36.09	52.94	62.54	83.56	240.64	110.61	49.69	48.04	39.61	49.44	53.84	67.81	125.41
	Year	15/16	73.88	74.62	73.14	237.05	137.84	32.09	22.81	22.31	31.91	43.39	66.10	78.07	145.46	119.92	42.06	44.99	42.45	45.18	50.18	69.88	98.14
	Episodes	F/N	6,490.00	2,711.00	3,779.00	65.00	437.00	265.00	216.00	401.00	610.00	327.00	269.00	121.00	52.00	353.00	331.00	403.00	752.00	867.00	400.00	344.00	277.00
**London**	Quarter	1	114.61	63.66	165.57	0.00	177.25	69.73	24.56	36.74	45.40	67.25	90.66	61.32	819.49	170.22	78.65	57.63	59.51	59.10	64.12	84.74	96.62
		2	90.68	83.62	97.74	276.50	138.45	48.15	24.24	33.65	38.06	55.66	77.21	60.71	326.64	148.30	63.62	36.81	49.17	57.42	56.43	74.22	67.09
		3	80.99	80.98	81.00	220.63	123.52	59.37	29.63	28.71	33.94	49.78	67.57	115.67	239.69	110.81	46.60	42.54	44.79	51.59	63.25	57.28	72.49
		4	86.82	82.37	91.27	262.36	114.57	52.87	25.70	24.16	39.48	52.40	72.71	97.06	261.61	131.11	55.40	39.18	47.74	47.04	57.46	92.05	89.82
	Year	15/16	93.15	77.75	108.56	191.24	138.00	57.44	26.02	30.69	39.22	56.20	76.96	83.94	409.02	139.94	60.96	43.95	50.25	53.66	60.26	77.36	81.66
	Episodes	F/N	6,978.00	2,963.00	4,015.00	104.00	513.00	447.00	187.00	798.00	535.00	180.00	141.00	58.00	107.00	518.00	453.00	344.00	1,394.00	700.00	222.00	184.00	93.00
**Midlands And East**	Quarter	1	70.45	85.23	55.68	342.84	99.42	31.01	15.35	25.43	30.88	42.43	59.38	120.31	0.00	113.69	41.70	40.41	44.01	49.03	59.59	77.46	75.22
		2	55.61	44.65	66.56	41.71	107.96	29.75	21.50	21.18	21.54	32.04	53.55	72.63	140.48	97.80	36.14	31.27	44.79	41.28	54.68	61.13	91.48
		3	54.05	49.99	58.10	117.37	81.08	25.84	21.05	19.04	20.91	30.26	46.35	88.03	134.60	95.88	30.22	33.81	38.51	26.15	37.99	46.58	79.20
		4	57.50	54.18	60.82	160.00	91.13	28.28	17.72	23.73	26.61	36.34	45.33	58.53	165.02	85.38	34.05	28.21	31.74	35.41	37.79	51.28	78.52
	Year	15/16	59.37	58.43	60.30	165.38	94.83	28.71	18.88	22.37	25.01	35.29	51.04	84.38	111.06	97.95	35.50	33.32	39.61	37.92	47.33	58.97	81.05
	Episodes	F/N	5,172.00	2,105.00	3,067.00	33.00	298.00	234.00	143.00	373.00	460.00	251.00	194.00	119.00	35.00	299.00	276.00	239.00	682.00	700.00	357.00	271.00	208.00

Mean weekly incidence rate per 100,000 Persons.

Upper Respiratory Tract Infections (ICD10: J00-J06)

		All ages			Male									Female								
		Male & Female	Male	Female	<1yr	1-4yrs	5-14yrs	15-24yrs	25-44yrs	45-64yrs	65-74yrs	75-84yrs	85+yrs	<1yr	1-4yrs	5-14yrs	15-24yrs	25-44yrs	45-64yrs	65-74yrs	75-84yrs	85+yrs
**4 weekly**	1	202.69	189.48	215.91	0.00	997.51	272.61	110.69	85.85	58.65	65.88	52.84	61.27	0.00	852.19	303.05	221.70	199.47	128.57	105.25	87.21	45.73
	2	208.97	181.95	236.00	390.63	712.39	209.64	77.73	66.43	47.61	43.61	40.51	48.97	781.25	596.57	219.67	165.47	131.93	84.71	66.05	45.86	32.53
	3	197.78	197.01	198.56	562.65	738.09	179.53	77.31	63.58	44.06	46.06	30.80	31.01	320.26	694.89	213.71	153.02	147.97	88.21	64.89	64.88	39.23
	4	155.25	160.25	150.26	479.10	542.41	114.37	75.53	60.26	41.93	49.55	44.77	34.35	243.24	457.07	110.75	161.56	125.37	84.46	76.64	58.87	34.35
	5	243.49	243.21	243.78	817.33	807.10	184.67	111.58	64.99	48.49	60.98	40.20	53.49	614.67	736.97	212.13	195.72	145.00	91.58	80.98	55.09	61.84
	6	381.79	373.51	390.08	1083.49	1473.63	275.30	110.61	84.59	66.74	85.66	79.92	101.65	887.53	1348.14	313.71	248.80	207.89	151.59	113.18	110.39	129.46
	7	416.81	396.30	437.33	1235.13	1576.35	261.48	106.03	87.21	67.84	76.86	80.04	75.76	1194.42	1572.26	292.70	235.51	190.85	131.11	128.12	97.20	93.76
	8	574.22	572.35	576.09	2229.55	1891.89	436.77	133.71	96.51	80.49	95.41	83.43	103.42	1805.75	1817.71	473.21	267.42	244.72	177.95	154.84	129.06	114.17
	9	400.91	378.99	422.83	1315.82	1138.75	280.08	142.78	111.68	100.28	116.28	113.71	91.53	1265.28	1030.27	326.84	258.89	262.63	196.59	154.25	145.43	165.29
	10	536.39	525.27	547.50	1778.38	1752.24	519.61	150.86	119.38	110.96	109.10	86.12	100.76	1500.91	1608.34	543.31	307.08	306.24	235.04	174.50	137.12	115.02
	11	515.26	501.33	529.19	1651.38	1651.19	464.84	174.91	134.40	112.16	118.75	86.01	118.36	1485.60	1480.29	505.21	322.49	328.85	236.05	156.53	142.74	104.93
	12	453.15	436.58	469.73	1553.31	1310.24	444.96	155.35	120.36	95.70	103.90	90.70	54.68	1324.58	1266.00	502.96	281.91	269.83	187.39	156.96	130.99	106.94
	13	347.97	339.62	356.33	1390.50	940.52	254.45	121.25	84.02	66.00	72.04	73.09	54.70	1204.43	863.00	291.77	218.61	196.57	143.35	112.35	101.13	75.75
**Quarter**	1	203.12	189.48	216.75	293.31	829.96	224.59	90.28	73.02	50.76	52.93	42.27	48.17	338.93	725.14	249.90	183.27	162.84	102.66	80.77	67.61	39.67
	2	269.03	266.14	271.93	803.98	986.52	195.73	98.97	70.50	53.75	66.08	57.25	62.47	626.53	889.26	213.18	202.35	162.02	110.69	91.49	76.21	75.64
	3	480.27	467.09	493.45	1669.59	1562.11	361.23	133.19	102.49	87.43	97.99	91.24	98.57	1450.13	1494.50	400.38	266.10	244.38	178.96	152.28	127.94	126.33
	4	454.29	441.26	467.33	1569.97	1371.79	407.55	150.89	114.62	94.79	101.51	84.40	75.81	1364.93	1266.53	451.00	278.72	273.47	198.21	147.46	127.01	98.60
**Year**	15/16	353.61	342.88	364.34	1093.38	1191.07	299.35	118.94	90.62	72.12	80.04	69.08	71.34	953.05	1097.11	330.93	233.48	211.86	148.58	118.56	100.21	85.32
**Episodes**	F/N	121728.00	50253.00	71475.00	1998.00	16864.00	10261.00	4464.00	7707.00	5435.00	2152.00	1004.00	368.00	1689.00	15022.00	10895.00	8736.00	18052.00	11077.00	3424.00	1760.00	820.00

Mean weekly incidence rate per 100,000 Persons.

Upper Respiratory Tract Infections (ICD10: J00-J06)

			All ages			Male								Female							
			Male & Female	Male	Female	<1yr	1-4yrs	5-14yrs	15-24yrs	25-44yrs	45-64yrs	65-74yrs	75-84yrs	85+yrs	<1yr	1-4yrs	5-14yrs	15-24yrs	25-44yrs	45-64yrs	65-74yrs	75-84yrs	85+yrs
**North**	Quarter	1	208.90	216.05	201.76	389.91	950.72	217.53	103.23	84.39	56.35	53.91	44.89	43.48	0.00	795.36	284.85	216.96	197.06	116.32	83.69	84.23	37.35
		2	257.02	243.04	271.01	678.09	933.27	175.88	92.84	66.60	53.20	66.66	62.49	58.31	527.19	934.06	223.77	216.14	174.61	113.41	92.02	75.07	82.78
		3	475.76	470.31	481.21	1,763.47	1,523.23	345.43	123.74	101.16	87.84	102.29	102.05	83.61	1,365.86	1,472.63	390.62	247.38	255.88	185.41	160.03	138.62	114.45
		4	468.14	456.05	480.24	1,738.84	1,378.89	384.64	139.91	117.16	90.04	101.57	82.43	70.98	1,513.95	1,260.70	431.42	255.57	271.49	190.82	157.52	124.97	115.69
	Year	15/16	354.64	348.43	360.85	1,153.83	1,199.97	282.83	115.40	92.79	72.20	81.49	73.14	64.23	864.24	1,118.43	334.53	234.42	225.64	152.23	123.96	106.08	88.10
	Episodes	F/N	37,343.00	15,073.00	22,270.00	650.00	4,760.00	2,829.00	1,554.00	2,246.00	1,770.00	787.00	369.00	108.00	520.00	4,252.00	3,206.00	3,178.00	5,208.00	3,709.00	1,264.00	653.00	280.00
**South**	Quarter	1	162.66	149.87	175.44	76.16	768.39	189.58	86.91	69.80	47.55	34.75	41.61	34.11	160.26	629.24	208.59	176.20	148.57	90.03	61.47	53.66	50.93
		2	277.97	283.64	272.30	1,027.79	962.41	173.70	94.92	69.37	51.15	51.28	53.22	68.96	761.63	829.18	198.90	198.19	162.14	97.90	83.62	64.14	55.02
		3	492.84	474.94	510.74	1,798.03	1,618.90	296.82	127.22	102.76	78.00	82.14	72.96	97.61	1,718.99	1,451.39	362.12	299.17	238.05	157.50	141.03	114.22	114.21
		4	451.03	450.55	451.52	1,699.28	1,361.10	380.36	149.99	118.26	93.57	101.33	87.76	63.32	1,260.36	1,281.01	418.05	278.75	287.03	193.86	137.72	110.81	96.06
	Year	15/16	348.10	341.84	354.37	1,160.67	1,181.16	262.38	115.42	90.58	68.06	68.02	64.34	65.95	980.69	1,052.11	299.20	238.84	210.42	135.94	106.56	86.18	79.37
	Episodes	F/N	27,143.00	11,315.00	15,828.00	445.00	3,758.00	2,185.00	1,107.00	1,645.00	1,302.00	513.00	258.00	102.00	359.00	3,151.00	2,349.00	2,130.00	3,751.00	2,602.00	845.00	421.00	220.00
**London**	Quarter	1	214.97	228.84	201.10	480.77	905.56	281.76	87.64	72.49	55.48	67.02	41.99	66.85	76.92	819.51	289.23	165.97	150.76	118.85	101.97	69.35	17.35
		2	304.93	298.76	311.10	838.92	1,122.92	256.90	117.51	73.17	64.13	84.58	69.65	61.03	752.21	1,030.25	253.48	187.37	159.30	122.52	103.38	107.59	83.81
		3	498.31	480.16	516.46	1,662.92	1,598.29	460.28	128.09	93.84	94.13	105.84	96.58	81.50	1,423.96	1,643.45	472.34	211.57	217.66	205.02	169.81	154.15	150.16
		4	461.35	444.99	477.71	1,464.91	1,424.41	470.51	142.64	103.94	102.15	113.36	91.92	91.04	1,394.31	1,286.49	526.22	244.17	231.45	207.03	166.04	147.52	96.19
	Year	15/16	371.61	364.73	378.50	1,118.54	1,265.85	369.31	119.42	86.20	79.41	93.09	75.35	75.41	920.95	1,196.65	387.97	203.06	190.58	164.18	135.88	120.18	87.05
	Episodes	F/N	31,884.00	13,408.00	18,476.00	599.00	4,975.00	3,011.00	874.00	2,320.00	1,130.00	306.00	141.00	52.00	536.00	4,630.00	3,079.00	1,646.00	5,458.00	2,219.00	515.00	290.00	103.00
**Midlands And East**	Quarter	1	225.94	163.15	288.72	226.42	695.16	209.50	83.32	65.41	43.68	56.05	40.58	48.25	1,118.52	656.45	216.94	173.94	154.99	85.42	75.95	63.21	53.05
		2	236.21	239.11	233.31	671.11	927.48	176.44	90.61	72.85	46.51	61.82	43.64	61.57	465.08	763.55	176.59	207.69	152.03	108.92	86.94	58.04	80.95
		3	454.17	442.96	465.37	1,453.95	1,508.02	342.39	153.70	112.21	89.73	101.71	93.38	131.55	1,291.73	1,410.52	376.45	306.29	265.95	167.92	138.25	104.77	126.48
		4	436.64	413.44	459.84	1,376.84	1,322.76	394.67	171.01	119.13	93.41	89.78	75.49	77.90	1,291.11	1,237.90	428.32	336.40	303.92	201.15	128.53	124.75	86.49
	Year	15/16	340.10	316.53	363.66	940.47	1,117.30	282.90	125.53	92.90	68.81	77.57	63.50	79.78	1,046.32	1,021.27	302.00	257.60	220.82	141.99	107.82	88.39	86.74
	Episodes	F/N	25,358.00	10,457.00	14,901.00	304.00	3,371.00	2,236.00	929.00	1,496.00	1,233.00	546.00	236.00	106.00	274.00	2,989.00	2,261.00	1,782.00	3,635.00	2,547.00	800.00	396.00	217.00

Mean weekly incidence rate per 100,000 Persons.

Urinary Tract Infection/Cystitis (ICD10: N30,N390)

		All ages			Male									Female								
		Male & Female	Male	Female	<1yr	1-4yrs	5-14yrs	15-24yrs	25-44yrs	45-64yrs	65-74yrs	75-84yrs	85+yrs	<1yr	1-4yrs	5-14yrs	15-24yrs	25-44yrs	45-64yrs	65-74yrs	75-84yrs	85+yrs
**4 weekly**	1	37.47	20.59	54.35	0.00	9.15	1.15	1.06	1.71	6.55	19.55	57.64	88.54	0.00	13.28	16.33	43.31	36.98	41.70	60.13	103.68	173.71
	2	37.93	22.49	53.38	0.00	6.15	2.81	2.64	1.80	6.39	18.08	50.45	114.08	0.00	11.62	13.44	33.23	34.86	41.50	76.90	94.54	174.32
	3	45.20	29.32	61.08	82.24	6.92	2.24	0.34	2.82	9.38	26.98	47.97	84.98	0.00	23.83	17.44	41.04	43.53	46.37	78.78	131.12	167.64
	4	43.19	19.86	66.51	0.00	6.28	2.21	0.76	3.15	6.88	23.12	46.24	90.12	0.00	21.75	16.31	41.87	41.22	46.47	91.45	142.88	196.63
	5	43.81	26.12	61.51	13.56	9.57	2.33	2.09	3.32	7.26	19.69	52.74	124.50	0.00	25.64	17.87	37.14	39.39	44.82	90.55	133.56	164.59
	6	43.65	21.94	65.36	21.91	7.36	2.67	1.01	3.83	8.08	22.23	42.61	87.77	25.31	29.97	17.73	50.29	39.37	46.11	75.68	137.33	166.41
	7	39.08	20.19	57.97	9.86	6.29	2.56	1.35	3.95	7.32	16.90	46.69	86.83	7.93	22.38	16.29	42.38	44.38	43.80	78.12	113.59	152.87
	8	40.42	19.70	61.13	0.00	4.96	2.38	1.49	2.16	7.68	20.76	53.13	84.75	16.06	26.80	17.30	47.03	37.80	43.13	83.83	112.20	166.03
	9	40.40	19.91	60.90	23.58	5.94	2.54	0.87	2.09	9.38	24.59	48.24	61.94	11.06	11.02	12.10	38.96	36.91	39.58	74.72	121.50	202.21
	10	45.00	26.11	63.89	13.01	9.06	3.34	1.83	3.35	8.05	29.66	64.12	102.58	18.09	24.06	24.37	47.75	40.55	41.17	68.59	114.77	195.70
	11	41.66	20.89	62.43	6.88	3.83	3.06	3.29	1.89	8.65	24.97	49.47	85.94	7.81	36.24	15.13	39.87	37.74	42.67	83.52	120.41	178.48
	12	40.28	21.35	59.21	13.49	10.51	2.66	0.41	1.86	7.93	20.73	51.57	82.99	25.30	27.66	17.83	41.05	33.16	41.15	67.77	105.05	173.95
	13	41.22	24.95	57.50	13.20	3.40	1.35	0.19	2.21	6.82	24.65	59.59	113.12	6.29	18.51	17.30	44.81	37.14	37.40	82.02	104.38	169.68
**Quarter**	1	39.99	23.86	56.12	25.30	7.54	2.00	1.32	2.08	7.37	21.38	52.45	95.31	0.00	16.02	15.78	39.51	38.34	43.07	71.03	109.31	172.03
	2	43.08	22.07	64.08	10.91	7.14	2.51	1.25	3.47	7.38	21.44	45.92	98.58	7.79	25.10	16.82	43.89	39.49	45.34	85.35	136.72	176.25
	3	40.97	21.80	60.15	14.29	6.91	2.54	1.08	2.54	8.53	22.51	54.43	83.33	12.49	20.68	16.75	42.97	40.58	41.46	78.11	114.67	173.60
	4	41.59	22.42	60.76	9.59	6.15	2.47	1.63	2.36	7.53	24.13	53.79	94.10	14.84	27.21	18.04	42.53	36.41	41.19	75.71	111.04	179.87
**Year**	15/16	41.41	22.53	60.29	14.92	6.92	2.38	1.33	2.61	7.70	22.40	51.69	92.85	8.89	22.35	16.87	42.23	38.66	42.74	77.52	117.81	175.52
**Episodes**	F/N	17602.00	2883.00	14719.00	18.00	96.00	82.00	48.00	221.00	573.00	596.00	764.00	485.00	18.00	294.00	552.00	1603.00	3272.00	3131.00	2178.00	2034.00	1637.00

Mean weekly incidence rate per 100,000 Persons.

Urinary Tract Infection/Cystitis (ICD10: N30,N390)

			All ages			Male								Female							
			Male & Female	Male	Female	<1yr	1-4yrs	5-14yrs	15-24yrs	25-44yrs	45-64yrs	65-74yrs	75-84yrs	85+yrs	<1yr	1-4yrs	5-14yrs	15-24yrs	25-44yrs	45-64yrs	65-74yrs	75-84yrs	85+yrs
**North**	Quarter	1	208.90	216.05	201.76	389.91	950.72	217.53	103.23	84.39	56.35	53.91	44.89	43.48	0.00	795.36	284.85	216.96	197.06	116.32	83.69	84.23	37.35
		2	257.02	243.04	271.01	678.09	933.27	175.88	92.84	66.60	53.20	66.66	62.49	58.31	527.19	934.06	223.77	216.14	174.61	113.41	92.02	75.07	82.78
		3	475.76	470.31	481.21	1,763.47	1,523.23	345.43	123.74	101.16	87.84	102.29	102.05	83.61	1,365.86	1,472.63	390.62	247.38	255.88	185.41	160.03	138.62	114.45
		4	468.14	456.05	480.24	1,738.84	1,378.89	384.64	139.91	117.16	90.04	101.57	82.43	70.98	1,513.95	1,260.70	431.42	255.57	271.49	190.82	157.52	124.97	115.69
	Year	15/16	354.64	348.43	360.85	1,153.83	1,199.97	282.83	115.40	92.79	72.20	81.49	73.14	64.23	864.24	1,118.43	334.53	234.42	225.64	152.23	123.96	106.08	88.10
	Episodes	F/N	37,343.00	15,073.00	22,270.00	650.00	4,760.00	2,829.00	1,554.00	2,246.00	1,770.00	787.00	369.00	108.00	520.00	4,252.00	3,206.00	3,178.00	5,208.00	3,709.00	1,264.00	653.00	280.00
**South**	Quarter	1	162.66	149.87	175.44	76.16	768.39	189.58	86.91	69.80	47.55	34.75	41.61	34.11	160.26	629.24	208.59	176.20	148.57	90.03	61.47	53.66	50.93
		2	277.97	283.64	272.30	1,027.79	962.41	173.70	94.92	69.37	51.15	51.28	53.22	68.96	761.63	829.18	198.90	198.19	162.14	97.90	83.62	64.14	55.02
		3	492.84	474.94	510.74	1,798.03	1,618.90	296.82	127.22	102.76	78.00	82.14	72.96	97.61	1,718.99	1,451.39	362.12	299.17	238.05	157.50	141.03	114.22	114.21
		4	451.03	450.55	451.52	1,699.28	1,361.10	380.36	149.99	118.26	93.57	101.33	87.76	63.32	1,260.36	1,281.01	418.05	278.75	287.03	193.86	137.72	110.81	96.06
	Year	15/16	348.10	341.84	354.37	1,160.67	1,181.16	262.38	115.42	90.58	68.06	68.02	64.34	65.95	980.69	1,052.11	299.20	238.84	210.42	135.94	106.56	86.18	79.37
	Episodes	F/N	27,143.00	11,315.00	15,828.00	445.00	3,758.00	2,185.00	1,107.00	1,645.00	1,302.00	513.00	258.00	102.00	359.00	3,151.00	2,349.00	2,130.00	3,751.00	2,602.00	845.00	421.00	220.00
**London**	Quarter	1	214.97	228.84	201.10	480.77	905.56	281.76	87.64	72.49	55.48	67.02	41.99	66.85	76.92	819.51	289.23	165.97	150.76	118.85	101.97	69.35	17.35
		2	304.93	298.76	311.10	838.92	1,122.92	256.90	117.51	73.17	64.13	84.58	69.65	61.03	752.21	1,030.25	253.48	187.37	159.30	122.52	103.38	107.59	83.81
		3	498.31	480.16	516.46	1,662.92	1,598.29	460.28	128.09	93.84	94.13	105.84	96.58	81.50	1,423.96	1,643.45	472.34	211.57	217.66	205.02	169.81	154.15	150.16
		4	461.35	444.99	477.71	1,464.91	1,424.41	470.51	142.64	103.94	102.15	113.36	91.92	91.04	1,394.31	1,286.49	526.22	244.17	231.45	207.03	166.04	147.52	96.19
	Year	15/16	371.61	364.73	378.50	1,118.54	1,265.85	369.31	119.42	86.20	79.41	93.09	75.35	75.41	920.95	1,196.65	387.97	203.06	190.58	164.18	135.88	120.18	87.05
	Episodes	F/N	31,884.00	13,408.00	18,476.00	599.00	4,975.00	3,011.00	874.00	2,320.00	1,130.00	306.00	141.00	52.00	536.00	4,630.00	3,079.00	1,646.00	5,458.00	2,219.00	515.00	290.00	103.00
**Midlands And East**	Quarter	1	225.94	163.15	288.72	226.42	695.16	209.50	83.32	65.41	43.68	56.05	40.58	48.25	1,118.52	656.45	216.94	173.94	154.99	85.42	75.95	63.21	53.05
		2	236.21	239.11	233.31	671.11	927.48	176.44	90.61	72.85	46.51	61.82	43.64	61.57	465.08	763.55	176.59	207.69	152.03	108.92	86.94	58.04	80.95
		3	454.17	442.96	465.37	1,453.95	1,508.02	342.39	153.70	112.21	89.73	101.71	93.38	131.55	1,291.73	1,410.52	376.45	306.29	265.95	167.92	138.25	104.77	126.48
		4	436.64	413.44	459.84	1,376.84	1,322.76	394.67	171.01	119.13	93.41	89.78	75.49	77.90	1,291.11	1,237.90	428.32	336.40	303.92	201.15	128.53	124.75	86.49
	Year	15/16	340.10	316.53	363.66	940.47	1,117.30	282.90	125.53	92.90	68.81	77.57	63.50	79.78	1,046.32	1,021.27	302.00	257.60	220.82	141.99	107.82	88.39	86.74
	Episodes	F/N	25,358.00	10,457.00	14,901.00	304.00	3,371.00	2,236.00	929.00	1,496.00	1,233.00	546.00	236.00	106.00	274.00	2,989.00	2,261.00	1,782.00	3,635.00	2,547.00	800.00	396.00	217.00

Mean weekly incidence rate per 100,000 Persons.

Viral Hepatitis (ICD10: B15-B19)

		All ages			Male									Female								
		Male & Female	Male	Female	<1yr	1-4yrs	5-14yrs	15-24yrs	25-44yrs	45-64yrs	65-74yrs	75-84yrs	85+yrs	<1yr	1-4yrs	5-14yrs	15-24yrs	25-44yrs	45-64yrs	65-74yrs	75-84yrs	85+yrs
**4 weekly**	1	0.16	0.13	0.18	0.00	0.00	0.00	0.00	0.45	0.74	0.00	0.00	0.00	0.00	0.00	0.00	0.00	0.17	0.00	1.49	0.00	0.00
	2	0.17	0.29	0.05	0.00	0.00	0.00	0.00	0.62	0.93	1.06	0.00	0.00	0.00	0.00	0.00	0.00	0.16	0.27	0.00	0.00	0.00
	3	0.22	0.39	0.04	0.00	0.00	0.00	0.00	1.57	1.96	0.00	0.00	0.00	0.00	0.00	0.00	0.00	0.00	0.38	0.00	0.00	0.00
	4	0.29	0.38	0.20	0.00	0.00	0.00	0.00	0.18	1.16	2.04	0.00	0.00	0.00	0.00	0.00	0.85	0.52	0.44	0.00	0.00	0.00
	5	0.15	0.24	0.06	0.00	0.00	0.00	0.00	0.83	0.29	1.08	0.00	0.00	0.00	0.00	0.00	0.00	0.13	0.36	0.00	0.00	0.00
	6	0.21	0.28	0.14	0.00	0.00	0.00	0.51	1.18	0.84	0.00	0.00	0.00	0.00	0.00	0.00	0.00	0.78	0.18	0.30	0.00	0.00
	7	0.19	0.34	0.05	0.00	0.00	0.00	0.46	0.73	0.78	1.08	0.00	0.00	0.00	0.00	0.00	0.00	0.00	0.44	0.00	0.00	0.00
	8	0.18	0.22	0.13	0.00	0.00	0.00	0.00	0.82	0.71	0.46	0.00	0.00	0.00	0.00	0.00	0.00	0.30	0.50	0.40	0.00	0.00
	9	0.20	0.17	0.24	0.00	0.00	0.00	0.00	0.11	0.99	0.42	0.00	0.00	0.00	0.00	0.48	0.00	0.82	0.35	0.00	0.51	0.00
	10	0.14	0.22	0.06	0.00	0.00	0.00	0.00	0.46	0.13	1.38	0.00	0.00	0.00	0.00	0.00	0.00	0.13	0.40	0.00	0.00	0.00
	11	0.22	0.31	0.14	0.00	0.00	0.00	0.40	1.04	0.39	0.94	0.00	0.00	0.00	0.00	0.00	0.37	0.48	0.43	0.00	0.00	0.00
	12	0.13	0.18	0.09	0.00	0.00	0.00	0.00	0.56	0.60	0.42	0.00	0.00	0.00	0.00	0.00	0.35	0.13	0.34	0.00	0.00	0.00
	13	0.17	0.22	0.11	0.00	0.00	0.00	0.00	1.20	0.26	0.00	0.53	0.00	0.00	0.00	0.00	0.00	0.31	0.67	0.00	0.00	0.00
**Quarter**	1	0.18	0.26	0.10	0.00	0.00	0.00	0.00	0.85	1.17	0.32	0.00	0.00	0.00	0.00	0.00	0.00	0.12	0.20	0.57	0.00	0.00
	2	0.20	0.29	0.12	0.00	0.00	0.00	0.16	0.75	0.71	0.96	0.00	0.00	0.00	0.00	0.00	0.26	0.44	0.30	0.09	0.00	0.00
	3	0.19	0.24	0.14	0.00	0.00	0.00	0.14	0.44	0.80	0.74	0.00	0.00	0.00	0.00	0.15	0.00	0.34	0.47	0.12	0.16	0.00
	4	0.18	0.25	0.11	0.00	0.00	0.00	0.11	0.93	0.36	0.65	0.15	0.00	0.00	0.00	0.00	0.21	0.30	0.46	0.00	0.00	0.00
**Year**	15/16	0.19	0.26	0.12	0.00	0.00	0.00	0.10	0.74	0.75	0.67	0.04	0.00	0.00	0.00	0.04	0.12	0.30	0.36	0.19	0.04	0.00
**Episodes**	F/N	194.00	133.00	61.00	0.00	0.00	0.00	3.00	66.00	51.00	12.00	1.00	0.00	0.00	0.00	1.00	4.00	26.00	25.00	4.00	1.00	0.00

Mean weekly incidence rate per 100,000 Persons.

Viral Hepatitis (ICD10: B15-B19)

			All ages			Male								Female							
			Male & Female	Male	Female	<1yr	1-4yrs	5-14yrs	15-24yrs	25-44yrs	45-64yrs	65-74yrs	75-84yrs	85+yrs	<1yr	1-4yrs	5-14yrs	15-24yrs	25-44yrs	45-64yrs	65-74yrs	75-84yrs	85+yrs
**North**	Quarter	1	0.03	0.06	0.00	0.00	0.00	0.00	0.00	0.29	0.26	0.00	0.00	0.00	0.00	0.00	0.00	0.00	0.00	0.00	0.00	0.00	0.00
		2	0.10	0.12	0.09	0.00	0.00	0.00	0.00	0.61	0.45	0.00	0.00	0.00	0.00	0.00	0.00	0.00	0.41	0.00	0.37	0.00	0.00
		3	0.13	0.14	0.12	0.00	0.00	0.00	0.00	0.55	0.67	0.00	0.00	0.00	0.00	0.00	0.00	0.00	0.14	0.30	0.00	0.62	0.00
		4	0.14	0.17	0.11	0.00	0.00	0.00	0.00	0.79	0.13	0.00	0.61	0.00	0.00	0.00	0.00	0.00	0.29	0.68	0.00	0.00	0.00
	Year	15/16	0.10	0.12	0.08	0.00	0.00	0.00	0.00	0.56	0.37	0.00	0.16	0.00	0.00	0.00	0.00	0.00	0.21	0.25	0.09	0.15	0.00
	Episodes	F/N	39.00	25.00	14.00	0.00	0.00	0.00	0.00	15.00	9.00	0.00	1.00	0.00	0.00	0.00	0.00	0.00	5.00	7.00	1.00	1.00	0.00
**South**	Quarter	1	0.15	0.28	0.03	0.00	0.00	0.00	0.00	0.97	1.52	0.00	0.00	0.00	0.00	0.00	0.00	0.00	0.27	0.00	0.00	0.00	0.00
		2	0.21	0.27	0.15	0.00	0.00	0.00	0.00	1.57	0.85	0.00	0.00	0.00	0.00	0.00	0.00	0.45	0.23	0.64	0.00	0.00	0.00
		3	0.20	0.22	0.18	0.00	0.00	0.00	0.00	0.22	0.71	1.06	0.00	0.00	0.00	0.00	0.00	0.00	0.69	0.41	0.49	0.00	0.00
		4	0.21	0.25	0.18	0.00	0.00	0.00	0.00	1.17	0.57	0.48	0.00	0.00	0.00	0.00	0.00	0.42	0.78	0.40	0.00	0.00	0.00
	Year	15/16	0.19	0.25	0.13	0.00	0.00	0.00	0.00	0.99	0.91	0.39	0.00	0.00	0.00	0.00	0.00	0.22	0.50	0.36	0.12	0.00	0.00
	Episodes	F/N	57.00	38.00	19.00	0.00	0.00	0.00	0.00	18.00	17.00	3.00	0.00	0.00	0.00	0.00	0.00	2.00	9.00	7.00	1.00	0.00	0.00
**London**	Quarter	1	0.45	0.56	0.34	0.00	0.00	0.00	0.00	1.47	2.30	1.30	0.00	0.00	0.00	0.00	0.00	0.00	0.00	0.81	2.29	0.00	0.00
		2	0.39	0.60	0.17	0.00	0.00	0.00	0.62	0.82	1.30	2.69	0.00	0.00	0.00	0.00	0.00	0.59	0.63	0.34	0.00	0.00	0.00
		3	0.26	0.32	0.19	0.00	0.00	0.00	0.00	0.47	1.09	1.33	0.00	0.00	0.00	0.00	0.00	0.00	0.54	1.16	0.00	0.00	0.00
		4	0.33	0.51	0.14	0.00	0.00	0.00	0.45	1.25	0.73	2.14	0.00	0.00	0.00	0.00	0.00	0.40	0.12	0.76	0.00	0.00	0.00
	Year	15/16	0.36	0.50	0.21	0.00	0.00	0.00	0.27	1.01	1.35	1.87	0.00	0.00	0.00	0.00	0.00	0.25	0.32	0.77	0.56	0.00	0.00
	Episodes	F/N	75.00	52.00	23.00	0.00	0.00	0.00	2.00	26.00	18.00	6.00	0.00	0.00	0.00	0.00	0.00	2.00	9.00	10.00	2.00	0.00	0.00
**Midlands And East**	Quarter	1	0.08	0.14	0.02	0.00	0.00	0.00	0.00	0.66	0.61	0.00	0.00	0.00	0.00	0.00	0.00	0.00	0.20	0.00	0.00	0.00	0.00
		2	0.12	0.15	0.08	0.00	0.00	0.00	0.00	0.00	0.22	1.16	0.00	0.00	0.00	0.00	0.00	0.00	0.49	0.23	0.00	0.00	0.00
		3	0.16	0.26	0.07	0.00	0.00	0.00	0.56	0.51	0.73	0.57	0.00	0.00	0.00	0.00	0.59	0.00	0.00	0.00	0.00	0.00	0.00
		4	0.03	0.06	0.00	0.00	0.00	0.00	0.00	0.52	0.00	0.00	0.00	0.00	0.00	0.00	0.00	0.00	0.00	0.00	0.00	0.00	0.00
	Year	15/16	0.10	0.15	0.04	0.00	0.00	0.00	0.14	0.42	0.38	0.42	0.00	0.00	0.00	0.00	0.14	0.00	0.17	0.06	0.00	0.00	0.00
	Episodes	F/N	23.00	18.00	5.00	0.00	0.00	0.00	1.00	7.00	7.00	3.00	0.00	0.00	0.00	0.00	1.00	0.00	3.00	1.00	0.00	0.00	0.00

Mean weekly incidence rate per 100,000 Persons.

Whooping Cough (ICD10: A37)

		All ages			Male									Female								
		Male & Female	Male	Female	<1yr	1-4yrs	5-14yrs	15-24yrs	25-44yrs	45-64yrs	65-74yrs	75-84yrs	85+yrs	<1yr	1-4yrs	5-14yrs	15-24yrs	25-44yrs	45-64yrs	65-74yrs	75-84yrs	85+yrs
**4 weekly**	1	0.14	0.21	0.08	0.00	0.00	0.35	0.33	0.73	0.45	0.00	0.00	0.00	0.00	0.00	0.41	0.00	0.31	0.00	0.00	0.00	0.00
	2	0.28	0.34	0.21	0.00	0.00	0.00	0.53	0.32	0.41	0.00	1.85	0.00	0.00	0.00	0.00	0.00	0.42	0.71	0.00	0.75	0.00
	3	0.12	0.08	0.17	0.00	0.00	0.48	0.00	0.23	0.00	0.00	0.00	0.00	0.00	0.00	0.52	0.41	0.16	0.00	0.45	0.00	0.00
	4	0.15	0.17	0.13	0.00	0.00	0.39	0.77	0.00	0.35	0.00	0.00	0.00	0.00	0.00	0.42	0.00	0.32	0.00	0.45	0.00	0.00
	5	0.15	0.12	0.17	0.00	0.00	0.84	0.26	0.00	0.00	0.00	0.00	0.00	0.00	0.00	0.00	0.91	0.00	0.63	0.00	0.00	0.00
	6	0.08	0.10	0.06	0.00	0.00	0.31	0.00	0.12	0.47	0.00	0.00	0.00	0.00	0.00	0.00	0.36	0.20	0.00	0.00	0.00	0.00
	7	0.08	0.05	0.12	0.00	0.00	0.00	0.00	0.29	0.11	0.00	0.00	0.00	0.00	0.00	0.47	0.19	0.12	0.34	0.00	0.00	0.00
	8	0.14	0.16	0.12	0.00	0.00	1.06	0.00	0.18	0.19	0.00	0.00	0.00	0.00	0.00	0.00	0.38	0.56	0.18	0.00	0.00	0.00
	9	0.11	0.08	0.14	0.00	0.00	0.00	0.25	0.00	0.51	0.00	0.00	0.00	0.00	0.00	0.33	0.20	0.56	0.13	0.00	0.00	0.00
	10	0.28	0.15	0.40	0.00	1.19	0.00	0.00	0.18	0.00	0.00	0.00	0.00	0.00	1.19	0.90	0.20	0.63	0.68	0.00	0.00	0.00
	11	0.11	0.21	0.00	0.00	1.18	0.45	0.00	0.12	0.17	0.00	0.00	0.00	0.00	0.00	0.00	0.00	0.00	0.00	0.00	0.00	0.00
	12	0.40	0.52	0.27	4.09	0.00	0.38	0.00	0.23	0.00	0.00	0.00	0.00	0.00	0.00	1.04	0.37	0.11	0.12	0.00	0.79	0.00
	13	0.07	0.07	0.07	0.00	0.00	0.37	0.00	0.11	0.16	0.00	0.00	0.00	0.00	0.00	0.00	0.18	0.32	0.11	0.00	0.00	0.00
**Quarter**	1	0.18	0.21	0.15	0.00	0.00	0.28	0.29	0.45	0.30	0.00	0.57	0.00	0.00	0.00	0.32	0.13	0.30	0.22	0.14	0.23	0.00
	2	0.12	0.12	0.11	0.00	0.00	0.47	0.31	0.04	0.29	0.00	0.00	0.00	0.00	0.00	0.13	0.39	0.16	0.19	0.14	0.00	0.00
	3	0.15	0.13	0.16	0.00	0.37	0.33	0.08	0.20	0.21	0.00	0.00	0.00	0.00	0.00	0.38	0.24	0.51	0.31	0.00	0.00	0.00
	4	0.20	0.23	0.17	1.17	0.34	0.34	0.00	0.13	0.10	0.00	0.00	0.00	0.00	0.34	0.43	0.21	0.18	0.15	0.00	0.22	0.00
**Year**	15/16	0.16	0.17	0.15	0.31	0.18	0.36	0.17	0.20	0.22	0.00	0.14	0.00	0.00	0.09	0.32	0.24	0.29	0.22	0.07	0.12	0.00
**Episodes**	F/N	120.00	54.00	66.00	1.00	2.00	12.00	6.00	15.00	17.00	0.00	1.00	0.00	0.00	1.00	10.00	11.00	22.00	18.00	2.00	2.00	0.00

Mean weekly incidence rate per 100,000 Persons.

Whooping Cough (ICD10: A37)

			All ages			Male									Female								
			Male & Female	Male	Female	<1yr	1-4yrs	5-14yrs	15-24yrs	25-44yrs	45-64yrs	65-74yrs	75-84yrs	85+yrs	<1yr	1-4yrs	5-14yrs	15-24yrs	25-44yrs	45-64yrs	65-74yrs	75-84yrs	85+yrs
**North**	Quarter	1	0.28	0.28	0.27	0.00	0.00	0.59	0.00	1.17	0.77	0.00	0.00	0.00	0.00	0.00	1.26	0.00	0.00	0.25	0.00	0.93	0.00
		2	0.06	0.13	0.00	0.00	0.00	0.38	0.32	0.15	0.29	0.00	0.00	0.00	0.00	0.00	0.00	0.00	0.00	0.00	0.00	0.00	0.00
		3	0.17	0.11	0.24	0.00	0.00	0.38	0.31	0.14	0.15	0.00	0.00	0.00	0.00	0.00	0.41	0.96	0.14	0.62	0.00	0.00	0.00
		4	0.33	0.53	0.13	4.67	0.00	0.00	0.00	0.14	0.00	0.00	0.00	0.00	0.00	0.00	0.33	0.44	0.00	0.41	0.00	0.00	0.00
	Year	15/16	0.21	0.27	0.16	1.23	0.00	0.33	0.15	0.39	0.30	0.00	0.00	0.00	0.00	0.00	0.50	0.35	0.03	0.32	0.00	0.23	0.00
	Episodes	F/N	39.00	19.00	20.00	1.00	0.00	3.00	2.00	7.00	6.00	0.00	0.00	0.00	0.00	0.00	4.00	6.00	1.00	8.00	0.00	1.00	0.00
**South**	Quarter	1	0.18	0.19	0.18	0.00	0.00	0.54	0.50	0.45	0.22	0.00	0.00	0.00	0.00	0.00	0.00	0.50	0.47	0.62	0.00	0.00	0.00
		2	0.18	0.21	0.15	0.00	0.00	1.04	0.42	0.00	0.42	0.00	0.00	0.00	0.00	0.00	0.51	0.44	0.00	0.42	0.00	0.00	0.00
		3	0.20	0.15	0.24	0.00	0.00	0.47	0.00	0.66	0.22	0.00	0.00	0.00	0.00	0.00	0.52	0.00	1.23	0.41	0.00	0.00	0.00
		4	0.08	0.14	0.02	0.00	0.00	0.86	0.00	0.00	0.38	0.00	0.00	0.00	0.00	0.00	0.00	0.00	0.00	0.20	0.00	0.00	0.00
	Year	15/16	0.16	0.17	0.15	0.00	0.00	0.73	0.23	0.27	0.31	0.00	0.00	0.00	0.00	0.00	0.25	0.23	0.42	0.41	0.00	0.00	0.00
	Episodes	F/N	38.00	19.00	19.00	0.00	0.00	6.00	2.00	5.00	6.00	0.00	0.00	0.00	0.00	0.00	2.00	2.00	7.00	8.00	0.00	0.00	0.00
**London**	Quarter	1	0.20	0.34	0.06	0.00	0.00	0.00	0.65	0.17	0.00	0.00	2.27	0.00	0.00	0.00	0.00	0.00	0.52	0.00	0.00	0.00	0.00
		2	0.03	0.00	0.06	0.00	0.00	0.00	0.00	0.00	0.00	0.00	0.00	0.00	0.00	0.00	0.00	0.00	0.16	0.35	0.00	0.00	0.00
		3	0.06	0.05	0.08	0.00	0.00	0.45	0.00	0.00	0.00	0.00	0.00	0.00	0.00	0.00	0.58	0.00	0.13	0.00	0.00	0.00	0.00
		4	0.09	0.01	0.17	0.00	0.00	0.00	0.00	0.13	0.00	0.00	0.00	0.00	0.00	0.00	0.86	0.42	0.24	0.00	0.00	0.00	0.00
	Year	15/16	0.10	0.10	0.09	0.00	0.00	0.11	0.16	0.08	0.00	0.00	0.56	0.00	0.00	0.00	0.37	0.11	0.26	0.09	0.00	0.00	0.00
	Episodes	F/N	17.00	5.00	12.00	0.00	0.00	1.00	1.00	2.00	0.00	0.00	1.00	0.00	0.00	0.00	3.00	1.00	7.00	1.00	0.00	0.00	0.00
**Midlands And East**	Quarter	1	0.05	0.02	0.08	0.00	0.00	0.00	0.00	0.00	0.19	0.00	0.00	0.00	0.00	0.00	0.00	0.00	0.20	0.00	0.55	0.00	0.00
		2	0.20	0.16	0.24	0.00	0.00	0.48	0.52	0.00	0.43	0.00	0.00	0.00	0.00	0.00	0.00	1.12	0.47	0.00	0.55	0.00	0.00
		3	0.15	0.22	0.08	0.00	1.46	0.00	0.00	0.00	0.48	0.00	0.00	0.00	0.00	0.00	0.00	0.00	0.52	0.22	0.00	0.00	0.00
		4	0.30	0.24	0.37	0.00	1.35	0.52	0.00	0.26	0.00	0.00	0.00	0.00	0.00	1.36	0.54	0.00	0.50	0.00	0.00	0.90	0.00
	Year	15/16	0.18	0.16	0.20	0.00	0.72	0.25	0.13	0.07	0.27	0.00	0.00	0.00	0.00	0.36	0.14	0.27	0.43	0.05	0.27	0.24	0.00
	Episodes	F/N	26.00	11.00	15.00	0.00	2.00	2.00	1.00	1.00	5.00	0.00	0.00	0.00	0.00	1.00	1.00	2.00	7.00	1.00	2.00	1.00	0.00
