# Supplementary material for: Incidence of Lower Respiratory Tract Infections and Atopic Conditions in Boys and Young Male Adults: Royal College of General Practitioners Research and Surveillance Centre Annual Report 2015-2016
Source: JMIR Public Health Surveill. 2018 Apr 30;4(2):e49. doi: 10.2196/publichealth.9307 (PMC5952117; doi:10.2196/publichealth.9307)
Supplement: Multimedia Appendix 2 [file publichealth_v4i2e49_app2.pdf]

Condition	Coding System	Read Code	Description
Acute Bronchitis	CTV3	A33y.	Whooping cough - other specified organism
Acute Bronchitis	CTV3	A33yz	Other whooping cough NOS
Acute Bronchitis	CTV3	A33z.	Whooping cough NOS
Acute Bronchitis	CTV3	Ayu39	[X]Whooping cough due to other Bordetella species
Acute Bronchitis	CTV3	Ayu3A	[X]Whooping cough, unspecified
Acute Bronchitis	CTV3	H06..	Acute bronchitis and bronchiolitis
Acute Bronchitis	CTV3	H060.	Acute bronchitis
Acute Bronchitis	CTV3	H0600	Acute fibrinous bronchitis
Acute Bronchitis	CTV3	H0601	Acute membranous bronchitis
Acute Bronchitis	CTV3	H0602	Acute pseudomembranous bronchitis
Acute Bronchitis	CTV3	H0603	Acute purulent bronchitis
Acute Bronchitis	CTV3	H0604	Acute croupous bronchitis
Acute Bronchitis	CTV3	H0605	Acute tracheobronchitis
Acute Bronchitis	CTV3	H0606	Acute pneumococcal bronchitis
Acute Bronchitis	CTV3	H0607	Acute streptococcal bronchitis
Acute Bronchitis	CTV3	H0608	Acute haemophilus influenzae bronchitis
Acute Bronchitis	CTV3	H0609	Acute neisseria catarrhalis bronchitis
Acute Bronchitis	CTV3	H060A	Acute bronchitis due to mycoplasma pneumoniae
Acute Bronchitis	CTV3	H060B	Acute bronchitis due to coxsackievirus
Acute Bronchitis	CTV3	H060C	Acute bronchitis due to parainfluenza virus
Acute Bronchitis	CTV3	H060D	Acute bronchitis due to respiratory syncytial virus
Acute Bronchitis	CTV3	H060E	Acute bronchitis due to rhinovirus
Acute Bronchitis	CTV3	H060F	Acute bronchitis due to echovirus
Acute Bronchitis	CTV3	H060v	Subacute bronchitis unspecified
Acute Bronchitis	CTV3	H060w	Acute viral bronchitis unspecified
Acute Bronchitis	CTV3	H060x	Acute bacterial bronchitis unspecified
Acute Bronchitis	CTV3	H060z	Acute bronchitis NOS
Acute Bronchitis	CTV3	H061.	Acute bronchiolitis
Acute Bronchitis	CTV3	H0610	Acute capillary bronchiolitis
Acute Bronchitis	CTV3	H0611	Acute obliterating bronchiolitis
Acute Bronchitis	CTV3	H0612	Acute bronchiolitis with bronchospasm
Acute Bronchitis	CTV3	H0613	Acute exudative bronchiolitis
Acute Bronchitis	CTV3	H0614	Obliterating fibrous bronchiolitis
Acute Bronchitis	CTV3	H0615	Acute bronchiolitis due to respiratory syncytial virus
Acute Bronchitis	CTV3	H0616	Acute bronchiolitis due to other specified organisms
Acute Bronchitis	CTV3	H0617	Acute bronchiolitis due to human metapneumovirus
Acute Bronchitis	CTV3	H061z	Acute bronchiolitis NOS
Acute Bronchitis	CTV3	H062.	Acute lower respiratory tract infection
Acute Bronchitis	CTV3	H06z.	Acute bronchitis or bronchiolitis NOS
Acute Bronchitis	CTV3	H06z0	Chest infection NOS
Acute Bronchitis	CTV3	H06z1	Lower resp tract infection
Acute Bronchitis	CTV3	H06z2	Recurrent chest infection
Acute Bronchitis	CTV3	H243.	Pneumonia with whooping cough
Acute Bronchitis	CTV3	H30..	Bronchitis unspecified

Acute Bronchitis	CTV3	H300.	Tracheobronchitis NOS
Acute Bronchitis	CTV3	H301.	Laryngotracheobronchitis
Acute Bronchitis	CTV3	H302.	Wheezy bronchitis
Acute Bronchitis	CTV3	H30z.	Bronchitis NOS
Acute Bronchitis	CTV3	Hyu10	[X]Acute bronchitis due to other specified organisms
Acute Bronchitis	CTV3	Hyu11	[X]Acute bronchiolitis due to other specified organisms
Acute Bronchitis	CTV3	X1006	Chest infection - unspecified bronchitis
Acute Bronchitis	CTV3	X1007	Acute bacterial bronchitis
Acute Bronchitis	CTV3	X1009	Acute mycoplasmal bronchitis
Acute Bronchitis	CTV3	X100A	Acute chlamydial bronchitis
Acute Bronchitis	CTV3	X100B	Acute viral bronchitis
Acute Bronchitis	CTV3	X100C	Acute viral bronchiolitis
Acute Bronchitis	CTV3	X100D	Acute bronchiolitis due to adenovirus
Acute Bronchitis	CTV3	Xa0lW	Acute laryngotracheobronchitis
Acute Bronchitis	CTV3	Xa0lZ	Asthmatic bronchitis
Acute Bronchitis	CTV3	XaDtB	Acute infective bronchitis
Acute Bronchitis	CTV3	XaDth	Acute infective tracheobronchitis
Acute Bronchitis	CTV3	XaYYt	Acute bronchiolitis due to human metapneumovirus
Acute Bronchitis	CTV3	XE0Qw	Whooping cough
Acute Bronchitis	CTV3	XE0Xr	Acute bronchitis
Acute Bronchitis	CTV3	XE0YL	Bronchitis unspecified
Acute Bronchitis	CTV3	XE0Yt	Acute: [bronchitis] or [chest infections] or [tracheobronchitis]
Acute Bronchitis	CTV3	XM1QT	Acute fibrinous laryngotracheobronchitis
Acute Bronchitis	CTV3	XM1QX	Acute wheezy bronchitis
Acute Bronchitis	READ2	H06..	Acute bronchitis and bronchiolitis
Acute Bronchitis	READ2	H060.	Acute bronchitis
Acute Bronchitis	READ2	H0600	Acute fibrinous bronchitis
Acute Bronchitis	READ2	H0601	Acute membranous bronchitis
Acute Bronchitis	READ2	H0602	Acute pseudomembranous bronchitis
Acute Bronchitis	READ2	H0603	Acute purulent bronchitis
Acute Bronchitis	READ2	H0604	Acute croupous bronchitis
Acute Bronchitis	READ2	H0605	Acute tracheobronchitis
Acute Bronchitis	READ2	H0606	Acute pneumococcal bronchitis
Acute Bronchitis	READ2	H0607	Acute streptococcal bronchitis
Acute Bronchitis	READ2	H0608	Acute haemophilus influenzae bronchitis
Acute Bronchitis	READ2	H0609	Acute neisseria catarrhalis bronchitis
Acute Bronchitis	READ2	H060A	Acute bronchitis due to mycoplasma pneumoniae
Acute Bronchitis	READ2	H060B	Acute bronchitis due to coxsackievirus
Acute Bronchitis	READ2	H060C	Acute bronchitis due to parainfluenza virus
Acute Bronchitis	READ2	H060D	Acute bronchitis due to respiratory syncytial virus
Acute Bronchitis	READ2	H060E	Acute bronchitis due to rhinovirus
Acute Bronchitis	READ2	H060F	Acute bronchitis due to echovirus
Acute Bronchitis	READ2	H060v	Subacute bronchitis unspecified
Acute Bronchitis	READ2	H060w	Acute viral bronchitis unspecified
Acute Bronchitis	READ2	H060x	Acute bacterial bronchitis unspecified

Acute Bronchitis	READ2	H060z	Acute bronchitis NOS
Acute Bronchitis	READ2	H061.	Acute bronchiolitis
Acute Bronchitis	READ2	H0610	Acute capillary bronchiolitis
Acute Bronchitis	READ2	H0611	Acute obliterating bronchiolitis
Acute Bronchitis	READ2	H0612	Acute bronchiolitis with bronchospasm
Acute Bronchitis	READ2	H0613	Acute exudative bronchiolitis
Acute Bronchitis	READ2	H0614	Obliterating fibrous bronchiolitis
Acute Bronchitis	READ2	H0615	Acute bronchiolitis due to respiratory syncytial virus
Acute Bronchitis	READ2	H0616	Acute bronchiolitis due to other specified organisms
Acute Bronchitis	READ2	H0617	Acute bronchiolitis due to human metapneumovirus
Acute Bronchitis	READ2	H061z	Acute bronchiolitis NOS
Acute Bronchitis	READ2	H062.	Acute lower respiratory tract infection
Acute Bronchitis	READ2	H06z.	Acute bronchitis or bronchiolitis NOS
Acute Bronchitis	READ2	H06z0	Chest infection NOS
Acute Bronchitis	READ2	H06z1	Lower resp tract infection
Acute Bronchitis	READ2	H06z2	Recurrent chest infection
Acute Bronchitis	READ2	H30..	Bronchitis unspecified
Acute Bronchitis	READ2	H300.	Tracheobronchitis NOS
Acute Bronchitis	READ2	H301.	Laryngotracheobronchitis
Acute Bronchitis	READ2	H302.	Wheezy bronchitis
Acute Bronchitis	READ2	H30z.	Bronchitis NOS
Acute Tonsilitis	CTV3	A3403	Streptococcal tonsillitis
Acute Tonsilitis	CTV3	A740.	Herpangina
Acute Tonsilitis	CTV3	AA12.	Vincent's pharyngitis
Acute Tonsilitis	CTV3	H02..	Acute pharyngitis
Acute Tonsilitis	CTV3	H020.	Acute gangrenous pharyngitis
Acute Tonsilitis	CTV3	H021.	Acute phlegmonous pharyngitis
Acute Tonsilitis	CTV3	H022.	Acute ulcerative pharyngitis
Acute Tonsilitis	CTV3	H023.	Acute bacterial pharyngitis
Acute Tonsilitis	CTV3	H0230	Acute pneumococcal pharyngitis
Acute Tonsilitis	CTV3	H0231	Acute staphylococcal pharyngitis
Acute Tonsilitis	CTV3	H023z	Acute bacterial pharyngitis NOS
Acute Tonsilitis	CTV3	H024.	Acute viral pharyngitis
Acute Tonsilitis	CTV3	H025.	Allergic pharyngitis
Acute Tonsilitis	CTV3	H02z.	Acute pharyngitis NOS
Acute Tonsilitis	CTV3	H03..	Acute tonsillitis
Acute Tonsilitis	CTV3	H030.	Acute erythematous tonsillitis
Acute Tonsilitis	CTV3	H031.	Acute follicular tonsillitis
Acute Tonsilitis	CTV3	H032.	Acute ulcerative tonsillitis
Acute Tonsilitis	CTV3	H033.	Acute catarrhal tonsillitis
Acute Tonsilitis	CTV3	H034.	Acute gangrenous tonsillitis
Acute Tonsilitis	CTV3	H035.	Acute bacterial tonsillitis
Acute Tonsilitis	CTV3	H0350	Acute pneumococcal tonsillitis
Acute Tonsilitis	CTV3	H0351	Acute staphylococcal tonsillitis
Acute Tonsilitis	CTV3	H035z	Acute bacterial tonsillitis NOS
Acute Tonsilitis	CTV3	H036.	Acute viral tonsillitis

Acute Tonsilitis	CTV3	H037.	Recurrent acute tonsillitis
Acute Tonsilitis	CTV3	H03z.	Acute tonsillitis NOS
Acute Tonsilitis	CTV3	X00mH	Acute herpes simplex pharyngitis
Acute Tonsilitis	CTV3	X00mI	Acute herpes zoster pharyngitis
Acute Tonsilitis	CTV3	X00mJ	Enteroviral lymphonodular pharyngitis
Acute Tonsilitis	CTV3	X00mL	Glandular fever pharyngitis
Acute Tonsilitis	CTV3	X00mq	Vincent's tonsillitis
Acute Tonsilitis	READ2	H02..	Acute pharyngitis
Acute Tonsilitis	READ2	H020.	Acute gangrenous pharyngitis
Acute Tonsilitis	READ2	H021.	Acute phlegmonous pharyngitis
Acute Tonsilitis	READ2	H022.	Acute ulcerative pharyngitis
Acute Tonsilitis	READ2	H023.	Acute bacterial pharyngitis
Acute Tonsilitis	READ2	H0230	Acute pneumococcal pharyngitis
Acute Tonsilitis	READ2	H0231	Acute staphylococcal pharyngitis
Acute Tonsilitis	READ2	H023z	Acute bacterial pharyngitis NOS
Acute Tonsilitis	READ2	H024.	Acute viral pharyngitis
Acute Tonsilitis	READ2	H025.	Allergic pharyngitis
Acute Tonsilitis	READ2	H02z.	Acute pharyngitis NOS
Acute Tonsilitis	READ2	H03..	Acute tonsillitis
Acute Tonsilitis	READ2	H030.	Acute erythematous tonsillitis
Acute Tonsilitis	READ2	H031.	Acute follicular tonsillitis
Acute Tonsilitis	READ2	H032.	Acute ulcerative tonsillitis
Acute Tonsilitis	READ2	H033.	Acute catarrhal tonsillitis
Acute Tonsilitis	READ2	H034.	Acute gangrenous tonsillitis
Acute Tonsilitis	READ2	H035.	Acute bacterial tonsillitis
Acute Tonsilitis	READ2	H0350	Acute pneumococcal tonsillitis
Acute Tonsilitis	READ2	H0351	Acute staphylococcal tonsillitis
Acute Tonsilitis	READ2	H035z	Acute bacterial tonsillitis NOS
Acute Tonsilitis	READ2	H036.	Acute viral tonsillitis
Acute Tonsilitis	READ2	H037.	Recurrent acute tonsillitis
Acute Tonsilitis	READ2	H03z.	Acute tonsillitis NOS
Allergic Rhinitis	CTV3	H17..	Allergic rhinitis
Allergic Rhinitis	CTV3	H170.	Allergic rhinitis due to pollens
Allergic Rhinitis	CTV3	H171.	Allergic rhinitis due to other allergens
Allergic Rhinitis	CTV3	H1710	Allergy to animal
Allergic Rhinitis	CTV3	H1711	Dog allergy
Allergic Rhinitis	CTV3	H172.	Allergic rhinitis due to unspecified allergen
Allergic Rhinitis	CTV3	H17z.	Allergic rhinitis NOS
Allergic Rhinitis	CTV3	Hyu20	[X]Other seasonal allergic rhinitis
Allergic Rhinitis	CTV3	Hyu21	[X]Other allergic rhinitis
Allergic Rhinitis	CTV3	X00l3	Cat allergy
Allergic Rhinitis	CTV3	X00l4	Dander (animal) allergy
Allergic Rhinitis	CTV3	X00l5	Feather allergy
Allergic Rhinitis	CTV3	X00l6	House dust allergy
Allergic Rhinitis	CTV3	X00l7	House dust mite allergy
Allergic Rhinitis	CTV3	X00l8	Hay fever - other allergen

Allergic Rhinitis	CTV3	X00l9	Hay fever - unspecified allergen
Allergic Rhinitis	CTV3	X00lA	Perennial allergic rhinitis
Allergic Rhinitis	CTV3	X00lB	Perennial allergic rhinitis with seasonal variation
Allergic Rhinitis	CTV3	X1020	Hay fever with asthma
Allergic Rhinitis	CTV3	Xa0lX	Seasonal allergic rhinitis
Allergic Rhinitis	CTV3	Xa7lM	Allergy to animal hair
Allergic Rhinitis	CTV3	XaIpW	Allergy to animal
Allergic Rhinitis	CTV3	XaOb5	Allergy to dog dander
Allergic Rhinitis	CTV3	XE0Y5	Allergic rhinitis
Allergic Rhinitis	CTV3	XE0Y6	Allergic rhinitis due to other allergens
Allergic Rhinitis	CTV3	XE0Y7	Allergic rhinitis due to unspecified allergen
Allergic Rhinitis	CTV3	XE0Z5	(Allergic rhinitis: [NOS] or [perennial] or [seasonal]) or (vasomotor rhinitis)
Allergic Rhinitis	CTV3	XE2QI	Allergic rhinitis due to pollens
Allergic Rhinitis	READ2	H17..	Allergic rhinitis
Allergic Rhinitis	READ2	H170.	Allergic rhinitis due to pollens
Allergic Rhinitis	READ2	H171.	Allergic rhinitis due to other allergens
Allergic Rhinitis	READ2	H1710	Allergy to animal
Allergic Rhinitis	READ2	H1711	Dog allergy
Allergic Rhinitis	READ2	H172.	Allergic rhinitis due to unspecified allergen
Allergic Rhinitis	READ2	H17z.	Allergic rhinitis NOS
Asthma	CTV3	173A.	Exercise induced asthma
Asthma	CTV3	H3120	Chronic asthmatic bronchitis
Asthma	CTV3	H33..	Asthma
Asthma	CTV3	H330.	Extrinsic (atopic) asthma
Asthma	CTV3	H3300	Extrinsic asthma without status asthmaticus
Asthma	CTV3	H3301	Extrinsic asthma with status asthmaticus
Asthma	CTV3	H330z	Extrinsic asthma NOS
Asthma	CTV3	H331.	Intrinsic asthma
Asthma	CTV3	H3310	Intrinsic asthma without status asthmaticus
Asthma	CTV3	H3311	Intrinsic asthma with status asthmaticus
Asthma	CTV3	H331z	Intrinsic asthma NOS
Asthma	CTV3	H332.	Mixed asthma
Asthma	CTV3	H333.	Acute exacerbation of asthma
Asthma	CTV3	H334.	Brittle asthma
Asthma	CTV3	H335.	Chronic asthma with fixed airflow obstruction
Asthma	CTV3	H33z.	Asthma unspecified
Asthma	CTV3	H33z0	Status asthmaticus NOS
Asthma	CTV3	H33z1	Asthma attack
Asthma	CTV3	H33z2	Late-onset asthma
Asthma	CTV3	H33zz	Asthma NOS
Asthma	CTV3	H3B..	Asthma-chronic obstructive pulmonary disease overlap syndrome
Asthma	CTV3	H44..	Pneumopathy due to inhalation of other dust
Asthma	CTV3	H440.	Byssinosis
Asthma	CTV3	H441.	Cannabinosis
Asthma	CTV3	H44z.	Pneumopathy due to inhalation of other dust NOS

Asthma	CTV3	H47y0	Detergent asthma
Asthma	CTV3	Ua1AX	Brittle asthma
Asthma	CTV3	X101k	Byssinosis grade 3
Asthma	CTV3	X101t	Childhood asthma
Asthma	CTV3	X101u	Late onset asthma
Asthma	CTV3	X101x	Allergic asthma
Asthma	CTV3	X101y	Extrinsic asthma with asthma attack
Asthma	CTV3	X101z	Allergic asthma NEC
Asthma	CTV3	X1020	Hay fever with asthma
Asthma	CTV3	X1021	Allergic non-atopic asthma
Asthma	CTV3	X1022	Intrinsic asthma with asthma attack
Asthma	CTV3	X1023	Drug-induced asthma
Asthma	CTV3	X1024	Aspirin-sensitive asthma with nasal polyps
Asthma	CTV3	X1025	Occupational asthma
Asthma	CTV3	X1026	Baker's asthma
Asthma	CTV3	X1027	Colophony asthma
Asthma	CTV3	X1028	Grain worker's asthma
Asthma	CTV3	X1029	Sulphite-induced asthma
Asthma	CTV3	X102B	Mill fever
Asthma	CTV3	X102D	Status asthmaticus
Asthma	CTV3	Xa0lZ	Asthmatic bronchitis
Asthma	CTV3	Xa1hD	Exacerbation of asthma
Asthma	CTV3	Xa9zf	Acute asthma
Asthma	CTV3	Xaa7B	Chronic asthma with fixed airflow obstruction
Asthma	CTV3	Xac33	Asthma-chronic obstructive pulmonary disease overlap syndrome
Asthma	CTV3	XaEKI	Flax-dressers' disease
Asthma	CTV3	XaJFG	Aspirin-induced asthma
Asthma	CTV3	XaKdk	Work aggravated asthma
Asthma	CTV3	XaLPE	Nocturnal asthma
Asthma	CTV3	XE0YQ	Allergic atopic asthma
Asthma	CTV3	XE0YR	Extrinsic asthma without status asthmaticus
Asthma	CTV3	XE0YS	Extrinsic asthma with status asthmaticus
Asthma	CTV3	XE0YT	Non-allergic asthma
Asthma	CTV3	XE0YU	Intrinsic asthma with status asthmaticus
Asthma	CTV3	XE0YV	Status asthmaticus NOS
Asthma	CTV3	XE0YW	Asthma attack
Asthma	CTV3	XE0YX	Asthma NOS
Asthma	CTV3	XE0ZP	Extrinsic asthma - atopy (& pollen)
Asthma	CTV3	XE0ZR	Asthma: [intrinsic] or [late onset]
Asthma	CTV3	XE0ZT	Asthma: [NOS] or [attack]
Asthma	CTV3	XM0s2	Asthma attack NOS
Asthma	READ2	H33..	Asthma
Asthma	READ2	H330.	Extrinsic (atopic) asthma
Asthma	READ2	H3300	Extrinsic asthma without status asthmaticus
Asthma	READ2	H3301	Extrinsic asthma with status asthmaticus
Asthma	READ2	H330z	Extrinsic asthma NOS

Asthma	READ2	H331.	Intrinsic asthma
Asthma	READ2	H3310	Intrinsic asthma without status asthmaticus
Asthma	READ2	H3311	Intrinsic asthma with status asthmaticus
Asthma	READ2	H331z	Intrinsic asthma NOS
Asthma	READ2	H332.	Mixed asthma
Asthma	READ2	H333.	Acute exacerbation of asthma
Asthma	READ2	H334.	Brittle asthma
Asthma	READ2	H335.	Chronic asthma with fixed airflow obstruction
Asthma	READ2	H33z.	Asthma unspecified
Asthma	READ2	H33z0	Status asthmaticus NOS
Asthma	READ2	H33z1	Asthma attack
Asthma	READ2	H33z2	Late-onset asthma
Asthma	READ2	H33zz	Asthma NOS
Asthma	READ2	H3B..	Asthma-chronic obstructive pulmonary disease overlap syndrome
Common Cold	CTV3	H00..	Acute nasopharyngitis
Common Cold	CTV3	H05..	Other acute upper respiratory infections
Common Cold	CTV3	H050.	Acute laryngopharyngitis
Common Cold	CTV3	H051.	Acute upper respiratory tract infection
Common Cold	CTV3	H052.	Pharyngotracheitis
Common Cold	CTV3	H053.	Tracheopharyngitis
Common Cold	CTV3	H054.	Recurrent upper respiratory tract infection
Common Cold	CTV3	H055.	Pharyngolaryngitis
Common Cold	CTV3	H05y.	Other upper respiratory infections of multiple sites
Common Cold	CTV3	H05z.	Upper respiratory infection NOS
Common Cold	CTV3	H0y..	Other specified acute respiratory infections
Common Cold	CTV3	H0z..	Acute respiratory infection NOS
Common Cold	CTV3	X1003	Viral upper respiratory tract infection NOS
Common Cold	CTV3	XE0Xl	Common cold
Common Cold	CTV3	XE0Xq	Upper respiratory infection NOS
Common Cold	CTV3	XE0Yn	(Acute nasopharyngitis) or (common cold) or (acute coryza) or (acute rhinitis) or (sniffles) or (snuffles)
Common Cold	READ2	H00..	Acute nasopharyngitis
Common Cold	READ2	H05..	Other acute upper respiratory infections
Common Cold	READ2	H050.	Acute laryngopharyngitis
Common Cold	READ2	H051.	Acute upper respiratory tract infection
Common Cold	READ2	H052.	Pharyngotracheitis
Common Cold	READ2	H053.	Tracheopharyngitis
Common Cold	READ2	H054.	Recurrent upper respiratory tract infection
Common Cold	READ2	H055.	Pharyngolaryngitis
Common Cold	READ2	H05y.	Other upper respiratory infections of multiple sites
Common Cold	READ2	H05z.	Upper respiratory infection NOS
Common Cold	READ2	H0y..	Other specified acute respiratory infections
Common Cold	READ2	H0z..	Acute respiratory infection NOS
Conjunctivitis	CTV3	A1734	Tuberculous keratoconjunctivitis
Conjunctivitis	CTV3	A213.	Oculoglandular tularaemia
Conjunctivitis	CTV3	A5321	Herpes zoster with keratoconjunctivitis

Conjunctivitis	CTV3	A55x0	Measles keratoconjunctivitis
Conjunctivitis	CTV3	A76..	Trachoma
Conjunctivitis	CTV3	A760.	Trachoma dubium - initial stage
Conjunctivitis	CTV3	A761.	Trachoma - active stage
Conjunctivitis	CTV3	A76z.	Unspecified trachoma
Conjunctivitis	CTV3	A77..	Other diseases of conjunctiva due to viruses and chlamydiae NEC
Conjunctivitis	CTV3	A770.	Inclusion conjunctivitis
Conjunctivitis	CTV3	A771.	Epidemic keratoconjunctivitis
Conjunctivitis	CTV3	A772.	Viral pharyngoconjunctivitis
Conjunctivitis	CTV3	A773.	Other adenoviral conjunctivitis
Conjunctivitis	CTV3	A774.	Epidemic haemorrhagic conjunctivitis
Conjunctivitis	CTV3	A776.	Keratoconjunctivitis due to adenovirus
Conjunctivitis	CTV3	A777.	Conjunctivitis due to adenovirus
Conjunctivitis	CTV3	A77y.	Other viral conjunctivitis
Conjunctivitis	CTV3	A77z.	Viral or chlamydial conjunctivitis NOS
Conjunctivitis	CTV3	A9840	Neonatal gonococcal conjunctivits
Conjunctivitis	CTV3	AD01.	Toxoplasma conjunctivitis
Conjunctivitis	CTV3	Ayu60	[X]Trachoma, unspecified
Conjunctivitis	CTV3	AyuD6	[X]Other viral conjunctivitis
Conjunctivitis	CTV3	AyuD7	[X]Viral conjunctivitis, unspecified
Conjunctivitis	CTV3	B503.	Malignant neoplasm of conjunctiva
Conjunctivitis	CTV3	B7E3.	Benign neoplasm of conjunctiva
Conjunctivitis	CTV3	C240.	Vitamin A deficiency with conjunctival xerosis
Conjunctivitis	CTV3	C2470	Vitamin A deficiency with xerophthalmia
Conjunctivitis	CTV3	F4A3.	Specific keratoconjunctivitis
Conjunctivitis	CTV3	F4A30	Phlyctenular keratoconjunctivitis
Conjunctivitis	CTV3	F4A31	Vernal conjunctivitis of limbus and cornea
Conjunctivitis	CTV3	F4A32	Keratoconjunctivitis sicca (excluding Sjogren's syndrome)
Conjunctivitis	CTV3	F4A34	Neurotrophic keratoconjunctivitis
Conjunctivitis	CTV3	F4A3z	Specific keratoconjunctivitis NOS
Conjunctivitis	CTV3	F4A4.	Other keratoconjunctivitis
Conjunctivitis	CTV3	F4A40	Unspecified keratoconjunctivitis
Conjunctivitis	CTV3	F4A41	Keratitis or keratoconjunctivitis in other exanthemata
Conjunctivitis	CTV3	F4A4z	Other keratoconjunctivitis NOS
Conjunctivitis	CTV3	F4C..	Disorders of conjunctiva
Conjunctivitis	CTV3	F4C0.	Acute conjunctivitis
Conjunctivitis	CTV3	F4C00	Unspecified acute conjunctivitis
Conjunctivitis	CTV3	F4C01	Serous conjunctivitis
Conjunctivitis	CTV3	F4C02	Acute follicular conjunctivitis
Conjunctivitis	CTV3	F4C03	Acute mucopurulent conjunctivitis
Conjunctivitis	CTV3	F4C04	Catarrhal conjunctivitis
Conjunctivitis	CTV3	F4C05	Pseudomembranous conjunctivitis
Conjunctivitis	CTV3	F4C06	Acute atopic conjunctivitis
Conjunctivitis	CTV3	F4C0z	Acute conjunctivitis NOS
Conjunctivitis	CTV3	F4C1.	Chronic conjunctivitis
Conjunctivitis	CTV3	F4C10	Unspecified chronic conjunctivitis

Conjunctivitis	CTV3	F4C11	Simple chronic conjunctivitis
Conjunctivitis	CTV3	F4C12	Chronic follicular conjunctivitis
Conjunctivitis	CTV3	F4C13	Vernal conjunctivitis
Conjunctivitis	CTV3	F4C14	Other chronic allergic conjunctivitis
Conjunctivitis	CTV3	F4C15	Parasitic conjunctivitis
Conjunctivitis	CTV3	F4C1z	Chronic conjunctivitis NOS
Conjunctivitis	CTV3	F4C2.	Blepharoconjunctivitis
Conjunctivitis	CTV3	F4C20	Unspecified blepharoconjunctivitis
Conjunctivitis	CTV3	F4C21	Angular blepharoconjunctivitis
Conjunctivitis	CTV3	F4C22	Contact blepharoconjunctivitis
Conjunctivitis	CTV3	F4C2z	Blepharoconjunctivitis NOS
Conjunctivitis	CTV3	F4C3.	Other and unspecified conjunctivitis
Conjunctivitis	CTV3	F4C30	Unspecified conjunctivitis
Conjunctivitis	CTV3	F4C31	Rosacea conjunctivitis
Conjunctivitis	CTV3	F4C32	Conjunctivitis with mucocutaneous disorder
Conjunctivitis	CTV3	F4C33	Bacterial conjunctivitis
Conjunctivitis	CTV3	F4C3z	Other conjunctivitis NOS
Conjunctivitis	CTV3	F4C4.	Pterygium
Conjunctivitis	CTV3	F4C40	Unspecified pterygium
Conjunctivitis	CTV3	F4C41	Peripheral pterygium, stationary
Conjunctivitis	CTV3	F4C42	Peripheral pterygium, progressive
Conjunctivitis	CTV3	F4C43	Central pterygium
Conjunctivitis	CTV3	F4C44	Double pterygium
Conjunctivitis	CTV3	F4C45	Recurrent pterygium
Conjunctivitis	CTV3	F4C4z	Pterygium NOS
Conjunctivitis	CTV3	F4C5.	Conjunctival degenerations and deposits
Conjunctivitis	CTV3	F4C50	Unspecified conjunctival degeneration
Conjunctivitis	CTV3	F4C51	Pinguecula
Conjunctivitis	CTV3	F4C52	Pseudopterygium
Conjunctivitis	CTV3	F4C53	Conjunctival xerosis
Conjunctivitis	CTV3	F4C54	Conjunctival concretions
Conjunctivitis	CTV3	F4C55	Conjunctival pigmentations
Conjunctivitis	CTV3	F4C56	Conjunctival deposits
Conjunctivitis	CTV3	F4C57	Conjunctival amyloidosis
Conjunctivitis	CTV3	F4C5z	Conjunctival degeneration or deposit NOS
Conjunctivitis	CTV3	F4C6.	Conjunctival scars
Conjunctivitis	CTV3	F4C60	Granuloma of conjunctiva
Conjunctivitis	CTV3	F4C61	Local conjunctival adhesions or strands
Conjunctivitis	CTV3	F4C62	Symblepharon
Conjunctivitis	CTV3	F4C63	Conjunctival scarring
Conjunctivitis	CTV3	F4C6z	Conjunctival scars NOS
Conjunctivitis	CTV3	F4C7.	Conjunctival vascular disorders and cysts
Conjunctivitis	CTV3	F4C70	Hyperaemia of conjunctiva
Conjunctivitis	CTV3	F4C71	Subconjunctival haemorrhage
Conjunctivitis	CTV3	F4C72	Conjunctival haemorrhage NOS
Conjunctivitis	CTV3	F4C73	Subconjunctival oedema

Conjunctivitis	CTV3	F4C74	Conjunctival oedema NOS
Conjunctivitis	CTV3	F4C75	Aneurysm of conjunctiva
Conjunctivitis	CTV3	F4C76	Subconjunctival vascular abnormality NOS
Conjunctivitis	CTV3	F4C77	Conjunctival vascular abnormality NOS
Conjunctivitis	CTV3	F4C78	Conjunctival cysts
Conjunctivitis	CTV3	F4C7z	Conjunctival vascular disorders or cysts NOS
Conjunctivitis	CTV3	F4Cy.	Other conjunctival disorders
Conjunctivitis	CTV3	F4Cy0	Filarial infection of conjunctiva
Conjunctivitis	CTV3	F4Cy1	Ocular pemphigoid
Conjunctivitis	CTV3	F4Cz.	Conjunctival disorder NOS
Conjunctivitis	CTV3	F4E45	Ankyloblepharon
Conjunctivitis	CTV3	FyuC0	[X]Other acute conjunctivitis
Conjunctivitis	CTV3	FyuC1	[X]Other conjunctivitis
Conjunctivitis	CTV3	FyuC2	[X]Other conjunctival vascular disorders and cysts
Conjunctivitis	CTV3	FyuC3	[X]Other specified disorders of conjunctiva
Conjunctivitis	CTV3	FyuC4	[X]Conjunctivitis in infectious and parasitic diseases classified elsewhere
Conjunctivitis	CTV3	FyuC5	[X]Conjunctivitis in other diseases classified elsewhere
Conjunctivitis	CTV3	FyuC7	[X]Other disorders of conjunctiva in diseases classified elsewhere
Conjunctivitis	CTV3	M1461	Ocular pemphigoid
Conjunctivitis	CTV3	P3622	Fused eyelids
Conjunctivitis	CTV3	SD82.	Superficial injury of conjunctiva
Conjunctivitis	CTV3	SG01.	Foreign body in conjunctival sac
Conjunctivitis	CTV3	X00a0	Conjunctival wound
Conjunctivitis	CTV3	X00a1	Conjunctival foreign body
Conjunctivitis	CTV3	X00a3	Radiational injury to conjunctiva
Conjunctivitis	CTV3	X00a5	Conjunctival telangiectasis
Conjunctivitis	CTV3	X00a6	Conjunctival lymphangiectasis
Conjunctivitis	CTV3	X00a7	Neoplasm of conjunctiva
Conjunctivitis	CTV3	X00ah	Keratoconjunctivitis
Conjunctivitis	CTV3	X00ai	Dendriform epithelial keratoconjunctivitis
Conjunctivitis	CTV3	X00aj	Superior limbic keratoconjunctivitis
Conjunctivitis	CTV3	X00ak	Diffuse stromal keratoconjunctivitis
Conjunctivitis	CTV3	X00al	Keratoconjunctivitis nodosa
Conjunctivitis	CTV3	X00Zh	Conjunctivitis
Conjunctivitis	CTV3	X00Zi	Atopic conjunctivitis
Conjunctivitis	CTV3	X00Zj	Seasonal allergic conjunctivitis
Conjunctivitis	CTV3	X00Zk	Perennial allergic conjunctivitis
Conjunctivitis	CTV3	X00Zl	Giant papillary conjunctivitis
Conjunctivitis	CTV3	X00Zm	Bacterial conjunctivitis
Conjunctivitis	CTV3	X00Zn	Gonococcal conjunctivitis
Conjunctivitis	CTV3	X00Zo	Viral conjunctivitis
Conjunctivitis	CTV3	X00Zp	Newcastle conjunctivitis
Conjunctivitis	CTV3	X00Zq	Chlamydial conjunctivitis
Conjunctivitis	CTV3	X00Zr	Fungal conjunctivitis
Conjunctivitis	CTV3	X00Zu	Conjunctival degeneration

Conjunctivitis	CTV3	X00Zv	Conjunctival amyloidosis
Conjunctivitis	CTV3	X00Zw	Argyrosis of conjunctiva
Conjunctivitis	CTV3	X00Zx	Injury of conjunctiva
Conjunctivitis	CTV3	X00Zy	Conjunctivitis artefacta
Conjunctivitis	CTV3	X75cX	Discharge from eye
Conjunctivitis	CTV3	X75fa	Symblepharon to lid
Conjunctivitis	CTV3	X75fb	Symblepharon to globe
Conjunctivitis	CTV3	X75ga	Bulbar conjunctival degeneration
Conjunctivitis	CTV3	X78bL	Bowen's disease of conjunctiva
Conjunctivitis	CTV3	X78bM	Squamous cell carcinoma of conjunctiva
Conjunctivitis	CTV3	X78bN	Malignant melanoma of conjunctiva
Conjunctivitis	CTV3	X78bP	Papilloma of conjunctiva
Conjunctivitis	CTV3	X78bQ	Naevus of conjunctiva
Conjunctivitis	CTV3	X78je	Kaposi's sarcoma of conjunctiva
Conjunctivitis	CTV3	X78VF	Oculocutaneous melanocytic naevus
Conjunctivitis	CTV3	Xa01u	Acute adenoviral follicular conjunctivitis
Conjunctivitis	CTV3	Xa0Aw	Neonatal viral conjunctivitis
Conjunctivitis	CTV3	Xa0lQ	Xerophthalmia
Conjunctivitis	CTV3	Xa7Hs	Infective conjunctivitis
Conjunctivitis	CTV3	Xa8A5	Oedema of conjunctiva
Conjunctivitis	CTV3	Xa9BV	Keratoconjunctivitis sicca
Conjunctivitis	CTV3	Xa9BY	Conjunctival deposits due to drugs
Conjunctivitis	CTV3	Xa9BZ	Conjunctival vascular abnormality
Conjunctivitis	CTV3	XaF7u	Contact lens related giant papillary conjunctivitis
Conjunctivitis	CTV3	XaFae	Conjunctival lipodermoid
Conjunctivitis	CTV3	XaFaf	Limbal dermoid of conjunctiva
Conjunctivitis	CTV3	XaFTZ	Ocular cicatricial pemphigoid
Conjunctivitis	CTV3	XaNDk	Primary acquired melanosis of conjunctiva with atypia
Conjunctivitis	CTV3	XaNDl	Primary acquired melanosis of conjunctiva without atypia
Conjunctivitis	CTV3	XE0RO	Trachoma - active stage
Conjunctivitis	CTV3	XE0RP	Inclusion conjunctivitis
Conjunctivitis	CTV3	XE0RQ	Other viral conjunctivitis
Conjunctivitis	CTV3	XE0RR	Viral or chlamydial conjunctivitis NOS
Conjunctivitis	CTV3	XE16a	Pseudomembranous conjunctivitis
Conjunctivitis	CTV3	XE16b	Other chronic allergic conjunctivitis
Conjunctivitis	CTV3	XE16c	Conjunctival xerosis
Conjunctivitis	CTV3	XE16d	Conjunctival pigmentation
Conjunctivitis	CTV3	XE16e	Local conjunctival adhesions
Conjunctivitis	CTV3	XE16f	Conjunctival oedema NOS
Conjunctivitis	CTV3	XE16P	Punctate epithelial keratoconjunctivitis
Conjunctivitis	CTV3	XE16X	Acute conjunctivitis
Conjunctivitis	CTV3	XE16Y	Unspecified acute conjunctivitis
Conjunctivitis	CTV3	XE16Z	Acute mucopurulent conjunctivitis
Conjunctivitis	CTV3	XE19F	(Acute conjunctivitis) or (Newcastle's disease) or (viral pharyngoconjunctivitis) or (pink eye) or (sticky eye)
Conjunctivitis	CTV3	XE19H	(Disorders of conjunctiva NOS) or (chemosis) or (pinguecula) or (symblepharon)

Conjunctivitis	CTV3	XM1Q8	Eye infection
Conjunctivitis	READ2	F4C..	Disorders of conjunctiva
Conjunctivitis	READ2	F4C0.	Acute conjunctivitis
Conjunctivitis	READ2	F4C00	Unspecified acute conjunctivitis
Conjunctivitis	READ2	F4C01	Serous conjunctivitis
Conjunctivitis	READ2	F4C02	Acute follicular conjunctivitis
Conjunctivitis	READ2	F4C03	Acute mucopurulent conjunctivitis
Conjunctivitis	READ2	F4C04	Catarrhal conjunctivitis
Conjunctivitis	READ2	F4C05	Pseudomembranous conjunctivitis
Conjunctivitis	READ2	F4C06	Acute atopic conjunctivitis
Conjunctivitis	READ2	F4C0z	Acute conjunctivitis NOS
Conjunctivitis	READ2	F4C1.	Chronic conjunctivitis
Conjunctivitis	READ2	F4C10	Unspecified chronic conjunctivitis
Conjunctivitis	READ2	F4C11	Simple chronic conjunctivitis
Conjunctivitis	READ2	F4C12	Chronic follicular conjunctivitis
Conjunctivitis	READ2	F4C13	Vernal conjunctivitis
Conjunctivitis	READ2	F4C14	Other chronic allergic conjunctivitis
Conjunctivitis	READ2	F4C15	Parasitic conjunctivitis
Conjunctivitis	READ2	F4C1z	Chronic conjunctivitis NOS
Conjunctivitis	READ2	F4C2.	Blepharoconjunctivitis
Conjunctivitis	READ2	F4C20	Unspecified blepharoconjunctivitis
Conjunctivitis	READ2	F4C21	Angular blepharoconjunctivitis
Conjunctivitis	READ2	F4C22	Contact blepharoconjunctivitis
Conjunctivitis	READ2	F4C2z	Blepharoconjunctivitis NOS
Conjunctivitis	READ2	F4C3.	Other and unspecified conjunctivitis
Conjunctivitis	READ2	F4C30	Unspecified conjunctivitis
Conjunctivitis	READ2	F4C31	Rosacea conjunctivitis
Conjunctivitis	READ2	F4C32	Conjunctivitis with mucocutaneous disorder
Conjunctivitis	READ2	F4C33	Bacterial conjunctivitis
Conjunctivitis	READ2	F4C3z	Other conjunctivitis NOS
Conjunctivitis	READ2	F4C4.	Pterygium
Conjunctivitis	READ2	F4C40	Unspecified pterygium
Conjunctivitis	READ2	F4C41	Peripheral pterygium, stationary
Conjunctivitis	READ2	F4C42	Peripheral pterygium, progressive
Conjunctivitis	READ2	F4C43	Central pterygium
Conjunctivitis	READ2	F4C44	Double pterygium
Conjunctivitis	READ2	F4C45	Recurrent pterygium
Conjunctivitis	READ2	F4C4z	Pterygium NOS
Conjunctivitis	READ2	F4C5.	Conjunctival degenerations and deposits
Conjunctivitis	READ2	F4C50	Unspecified conjunctival degeneration
Conjunctivitis	READ2	F4C51	Pinguecula
Conjunctivitis	READ2	F4C52	Pseudopterygium
Conjunctivitis	READ2	F4C53	Conjunctival xerosis
Conjunctivitis	READ2	F4C54	Conjunctival concretions
Conjunctivitis	READ2	F4C55	Conjunctival pigmentations

Conjunctivitis	READ2	F4C56	Conjunctival deposits
Conjunctivitis	READ2	F4C57	Conjunctival amyloidosis
Conjunctivitis	READ2	F4C5z	Conjunctival degeneration or deposit NOS
Conjunctivitis	READ2	F4C6.	Conjunctival scars
Conjunctivitis	READ2	F4C60	Granuloma of conjunctiva
Conjunctivitis	READ2	F4C61	Local conjunctival adhesions or strands
Conjunctivitis	READ2	F4C62	Symblepharon
Conjunctivitis	READ2	F4C63	Conjunctival scarring
Conjunctivitis	READ2	F4C6z	Conjunctival scars NOS
Conjunctivitis	READ2	F4C7.	Conjunctival vascular disorders and cysts
Conjunctivitis	READ2	F4C70	Hyperaemia of conjunctiva
Conjunctivitis	READ2	F4C71	Subconjunctival haemorrhage
Conjunctivitis	READ2	F4C72	Conjunctival haemorrhage NOS
Conjunctivitis	READ2	F4C73	Subconjunctival oedema
Conjunctivitis	READ2	F4C74	Conjunctival oedema NOS
Conjunctivitis	READ2	F4C75	Aneurysm of conjunctiva
Conjunctivitis	READ2	F4C76	Subconjunctival vascular abnormality NOS
Conjunctivitis	READ2	F4C77	Conjunctival vascular abnormality NOS
Conjunctivitis	READ2	F4C78	Conjunctival cysts
Conjunctivitis	READ2	F4C7z	Conjunctival vascular disorders or cysts NOS
Conjunctivitis	READ2	F4Cy.	Other conjunctival disorders
Conjunctivitis	READ2	F4Cy0	Filarial infection of conjunctiva
Conjunctivitis	READ2	F4Cy1	Ocular pemphigoid
Conjunctivitis	READ2	F4Cz.	Conjunctival disorder NOS
Intestinal Infectious Disease	CTV3	A0...	Intestinal infectious diseases
Intestinal Infectious Disease	CTV3	A00..	Cholera
Intestinal Infectious Disease	CTV3	A000.	Cholera - Vibrio cholerae
Intestinal Infectious Disease	CTV3	A001.	Cholera - Vibrio cholerae El Tor
Intestinal Infectious Disease	CTV3	A00z.	Cholera NOS
Intestinal Infectious Disease	CTV3	A01..	Typhoid and paratyphoid fevers
Intestinal Infectious Disease	CTV3	A010.	Typhoid fever
Intestinal Infectious Disease	CTV3	A011.	Paratyphoid fever A
Intestinal Infectious Disease	CTV3	A012.	Paratyphoid fever B
Intestinal Infectious Disease	CTV3	A013.	Paratyphoid fever C
Intestinal Infectious Disease	CTV3	A01z.	Paratyphoid fever NOS
Intestinal Infectious Disease	CTV3	A02..	Other salmonella infections
Intestinal Infectious Disease	CTV3	A020.	Salmonella gastroenteritis
Intestinal Infectious Disease	CTV3	A021.	Salmonella septicaemia
Intestinal Infectious Disease	CTV3	A022.	Localised salmonella infection
Intestinal Infectious Disease	CTV3	A0220	Local salmonella infection unspecified
Intestinal Infectious Disease	CTV3	A0221	Salmonella meningitis
Intestinal Infectious Disease	CTV3	A0222	Salmonella pneumonia
Intestinal Infectious Disease	CTV3	A0223	Salmonella arthritis
Intestinal Infectious Disease	CTV3	A0224	Salmonella osteomyelitis
Intestinal Infectious Disease	CTV3	A022z	Other local salmonella infection
Intestinal Infectious Disease	CTV3	A023.	Salmonella sepsis

Intestinal Infectious Disease	CTV3	A02y.	Other specified salmonella infection
Intestinal Infectious Disease	CTV3	A02z.	Salmonella infection NOS
Intestinal Infectious Disease	CTV3	A03..	Shigellosis
Intestinal Infectious Disease	CTV3	A030.	Shigella dysenteriae (group A)
Intestinal Infectious Disease	CTV3	A031.	Shigella flexneri (group B)
Intestinal Infectious Disease	CTV3	A032.	Shigella boydii (group C)
Intestinal Infectious Disease	CTV3	A033.	Shigella sonnei (group D)
Intestinal Infectious Disease	CTV3	A03y.	Other specified shigella infection
Intestinal Infectious Disease	CTV3	A03z.	Shigellosis NOS
Intestinal Infectious Disease	CTV3	A04..	Other bacterial food poisoning
Intestinal Infectious Disease	CTV3	A040.	Staphylococcal food poisoning
Intestinal Infectious Disease	CTV3	A041.	Botulism
Intestinal Infectious Disease	CTV3	A042.	Clostridium perfringens food poisoning
Intestinal Infectious Disease	CTV3	A043.	Other clostridia causing food poisoning
Intestinal Infectious Disease	CTV3	A044.	Vibrio parahaemolyticus food poisoning
Intestinal Infectious Disease	CTV3	A04y.	Other specified bacterial food poisoning
Intestinal Infectious Disease	CTV3	A04y0	Foodborne Bacillus cereus intoxication
Intestinal Infectious Disease	CTV3	A04z.	Food poisoning NOS
Intestinal Infectious Disease	CTV3	A05..	Amoebiasis
Intestinal Infectious Disease	CTV3	A050.	Acute amoebic dysentery
Intestinal Infectious Disease	CTV3	A051.	Chronic intestinal amoebiasis
Intestinal Infectious Disease	CTV3	A052.	Amoebic nondysenteric colitis
Intestinal Infectious Disease	CTV3	A053.	Amoebic liver abscess
Intestinal Infectious Disease	CTV3	A054.	Amoebic lung abscess
Intestinal Infectious Disease	CTV3	A055.	Amoebic brain abscess
Intestinal Infectious Disease	CTV3	A056.	Amoebic skin ulceration
Intestinal Infectious Disease	CTV3	A05y.	Amoebic infection of other sites
Intestinal Infectious Disease	CTV3	A05y0	Amoebic appendicitis
Intestinal Infectious Disease	CTV3	A05y1	Amoebic balanitis
Intestinal Infectious Disease	CTV3	A05y2	Amoeboma
Intestinal Infectious Disease	CTV3	A05yz	Amoebic infection of other sites NOS
Intestinal Infectious Disease	CTV3	A05z.	Amoebiasis NOS
Intestinal Infectious Disease	CTV3	A06..	Other protozoal intestinal diseases
Intestinal Infectious Disease	CTV3	A060.	Balantidiasis
Intestinal Infectious Disease	CTV3	A061.	Giardiasis - Lambliasis
Intestinal Infectious Disease	CTV3	A062.	Coccidiosis
Intestinal Infectious Disease	CTV3	A063.	Intestinal trichomoniasis
Intestinal Infectious Disease	CTV3	A064.	Cryptosporidiosis
Intestinal Infectious Disease	CTV3	A06y.	Other specified protozoal intestinal diseases
Intestinal Infectious Disease	CTV3	A06z.	Protozoal intestinal diseases NOS
Intestinal Infectious Disease	CTV3	A07..	Intestinal infection due to other organisms
Intestinal Infectious Disease	CTV3	A070.	Escherichia coli gastrointestinal tract infection
Intestinal Infectious Disease	CTV3	A0700	Enteropathogenic Escherichia coli infection
Intestinal Infectious Disease	CTV3	A0701	Enterotoxigenic Escherichia coli infection
Intestinal Infectious Disease	CTV3	A0702	Enteroinvasive Escherichia coli infection
Intestinal Infectious Disease	CTV3	A0703	Enterohaemorrhagic Escherichia coli infection

Intestinal Infectious Disease	CTV3	A071.	Arizona paracolon gastrointestinal tract infection
Intestinal Infectious Disease	CTV3	A072.	Aerobacter aerogenes gastrointestinal tract infection
Intestinal Infectious Disease	CTV3	A073.	Proteus gastrointestinal tract infection
Intestinal Infectious Disease	CTV3	A0730	Proteus mirabilis gastrointestinal tract infection
Intestinal Infectious Disease	CTV3	A0731	Proteus morganii gastrointestinal tract infection
Intestinal Infectious Disease	CTV3	A073z	Proteus gastrointestinal tract infection NOS
Intestinal Infectious Disease	CTV3	A074.	Other specified gastrointestinal tract bacterial infection
Intestinal Infectious Disease	CTV3	A0740	Staphylococcal gastrointestinal tract infection
Intestinal Infectious Disease	CTV3	A0741	Pseudomonas gastrointestinal tract infection
Intestinal Infectious Disease	CTV3	A0743	Campylobacter gastrointestinal tract infection
Intestinal Infectious Disease	CTV3	A0744	Enteritis due to Yersinia enterocolitica
Intestinal Infectious Disease	CTV3	A0745	Helicobacter pylori gastrointestinal tract infection
Intestinal Infectious Disease	CTV3	A074y	Other specified other gastrointestinal infection
Intestinal Infectious Disease	CTV3	A074z	Other specified gastrointestinal tract infections NOS
Intestinal Infectious Disease	CTV3	A075.	Unspecified bacterial enteritis
Intestinal Infectious Disease	CTV3	A076.	Enteritis due to specified virus
Intestinal Infectious Disease	CTV3	A0760	Enteritis due to adenovirus
Intestinal Infectious Disease	CTV3	A0761	Enteritis due to enterovirus
Intestinal Infectious Disease	CTV3	A0762	Enteritis due to rotavirus
Intestinal Infectious Disease	CTV3	A0763	Enteritis due to norovirus
Intestinal Infectious Disease	CTV3	A076z	Enteritis due to specified virus NOS
Intestinal Infectious Disease	CTV3	A07y.	Gastrointestinal tract infection due to specified organism NEC
Intestinal Infectious Disease	CTV3	A07y0	Viral gastroenteritis
Intestinal Infectious Disease	CTV3	A07y1	Infantile viral gastroenteritis
Intestinal Infectious Disease	CTV3	A07z.	Gastrointestinal tract infection due to specified organism NOS
Intestinal Infectious Disease	CTV3	A08..	Ill-defined intestinal tract infections
Intestinal Infectious Disease	CTV3	A080.	Infectious colitis, enteritis and gastroenteritis
Intestinal Infectious Disease	CTV3	A0801	Infectious colitis
Intestinal Infectious Disease	CTV3	A0802	Infectious enteritis
Intestinal Infectious Disease	CTV3	A0803	Infectious gastroenteritis
Intestinal Infectious Disease	CTV3	A0804	Catarrhal dysentery
Intestinal Infectious Disease	CTV3	A0805	Haemorrhagic dysentery
Intestinal Infectious Disease	CTV3	A080z	Infectious colitis, enteritis and gastroenteritis NOS
Intestinal Infectious Disease	CTV3	A081.	Colitis, enteritis and gastroenteritis presumed infectious
Intestinal Infectious Disease	CTV3	A0810	Colitis - presumed infectious origin
Intestinal Infectious Disease	CTV3	A0811	Enteritis - presumed infectious origin
Intestinal Infectious Disease	CTV3	A0812	Gastroenteritis - presumed infectious origin
Intestinal Infectious Disease	CTV3	A081z	Colitis, enteritis and gastroenteritis presumed infectious origin NOS
Intestinal Infectious Disease	CTV3	A082.	Infectious diarrhoea
Intestinal Infectious Disease	CTV3	A0820	Dysenteric diarrhoea
Intestinal Infectious Disease	CTV3	A0821	Epidemic diarrhoea
Intestinal Infectious Disease	CTV3	A082z	Infectious diarrhoea NOS
Intestinal Infectious Disease	CTV3	A083.	Diarrhoea of presumed infectious origin
Intestinal Infectious Disease	CTV3	A08z.	Ill defined gastrointestinal tract infections NOS
Intestinal Infectious Disease	CTV3	A0y..	Other specified infectious diseases of intestinal tract
Intestinal Infectious Disease	CTV3	A0z..	Intestinal tract infectious disease NOS

Intestinal Infectious Disease	CTV3	Ayu0H	[X]Diarrhoea and gastroenteritis of presumed infectious origin
Intestinal Infectious Disease	CTV3	H24y6	Pneumonia with typhoid fever
Intestinal Infectious Disease	CTV3	X303g	Colitis,enteritis ? infectious
Intestinal Infectious Disease	CTV3	X303l	Amoebic colitis
Intestinal Infectious Disease	CTV3	X303n	Fulminant amoebic colitis
Intestinal Infectious Disease	CTV3	X303q	Cytomegaloviral colitis
Intestinal Infectious Disease	CTV3	X30Bc	Prototheca diarrhoea
Intestinal Infectious Disease	CTV3	X30Bd	Food poisoning
Intestinal Infectious Disease	CTV3	X30BP	Vomiting - infective
Intestinal Infectious Disease	CTV3	X30BR	Adenoviral gastroenteritis
Intestinal Infectious Disease	CTV3	X30BS	Rotavirus gastroenteritis
Intestinal Infectious Disease	CTV3	X30BT	Norwalk virus gastroenteritis
Intestinal Infectious Disease	CTV3	X30BU	Small round structured virus gastroenteritis
Intestinal Infectious Disease	CTV3	X30BX	Dysentery
Intestinal Infectious Disease	CTV3	X70Ft	Infantile gastroenteritis
Intestinal Infectious Disease	CTV3	X70Fv	Bacterial food poisoning
Intestinal Infectious Disease	CTV3	X70Fw	Bacillus cereus food poisoning
Intestinal Infectious Disease	CTV3	X70Ge	Asymptomatic amoebic infection
Intestinal Infectious Disease	CTV3	X70Gf	Symptomatic non-invasive amoebic infection
Intestinal Infectious Disease	CTV3	X70Gg	Amoebic toxic megacolon
Intestinal Infectious Disease	CTV3	X70Gi	Amoebic perianal ulceration
Intestinal Infectious Disease	CTV3	X70GJ	Cholera - Vibrio cholerae O1 Classical biotype
Intestinal Infectious Disease	CTV3	X70GK	Cholera - non-O1 group vibrio
Intestinal Infectious Disease	CTV3	X70GL	Cholera - O139 group Vibrio cholerae
Intestinal Infectious Disease	CTV3	X70GN	Enterotoxigenic Escherichia coli gastrointestinal tract infection
Intestinal Infectious Disease	CTV3	X70GO	Travellers' diarrhoea
Intestinal Infectious Disease	CTV3	X70Gp	Cryptosporidiosis
Intestinal Infectious Disease	CTV3	X70GP	Enteropathogenic Escherichia coli gastrointestinal tract infection
Intestinal Infectious Disease	CTV3	X70GQ	Verotoxigenic Escherichia coli gastrointestinal tract infection
Intestinal Infectious Disease	CTV3	X70GR	Enteroinvasive Escherichia coli gastrointestinal tract infection
Intestinal Infectious Disease	CTV3	X70GS	Enteroadherent Escherichia coli gastrointestinal tract infection
Intestinal Infectious Disease	CTV3	X70GZ	Yersinia enterocolitis
Intestinal Infectious Disease	CTV3	X73Mv	Vibrio cholerae
Intestinal Infectious Disease	CTV3	Xa01t	Recurrent salmonella septicaemia
Intestinal Infectious Disease	CTV3	Xa2Vc	Astrovirus gastroenteritis
Intestinal Infectious Disease	CTV3	Xa2Vd	Calicivirus gastroenteritis
Intestinal Infectious Disease	CTV3	Xa3dy	Specific gastrointestinal infectious disease
Intestinal Infectious Disease	CTV3	Xa8ES	Acute infective gastroenteritis
Intestinal Infectious Disease	CTV3	Xa8I7	Salmonella food poisoning
Intestinal Infectious Disease	CTV3	Xa97W	Cholera - O1 group Vibrio cholerae
Intestinal Infectious Disease	CTV3	Xa97X	Staphylococcus aureus food poisoning
Intestinal Infectious Disease	CTV3	XaBE5	Helicobacter pylori gastrointestinal tract infection
Intestinal Infectious Disease	CTV3	XaBLu	Infantile viral gastroenteritis
Intestinal Infectious Disease	CTV3	XaDbT	Fungal gastroenteritis
Intestinal Infectious Disease	CTV3	XaREr	Enteritis due to norovirus
Intestinal Infectious Disease	CTV3	XaYaf	Sepsis due to Salmonella

Intestinal Infectious Disease	CTV3	XE0Qc	Cholera
Intestinal Infectious Disease	CTV3	XE0Qd	Typhoid fever
Intestinal Infectious Disease	CTV3	XE0Qe	Salmonella gastroenteritis
Intestinal Infectious Disease	CTV3	XE0Qf	Shigella dysenteriae (group A)
Intestinal Infectious Disease	CTV3	XE0Qg	Shigella sonnei (group D)
Intestinal Infectious Disease	CTV3	XE0Qi	Intestinal coccidiosis
Intestinal Infectious Disease	CTV3	XE0Qj	Staphylococcal gastrointestinal tract infection
Intestinal Infectious Disease	CTV3	XE0Qk	Pseudomonas gastrointestinal tract infection
Intestinal Infectious Disease	CTV3	XE0Ql	Campylobacter gastrointestinal tract infection
Intestinal Infectious Disease	CTV3	XE0Qm	Enteritis due to specified virus
Intestinal Infectious Disease	CTV3	XE0Qn	Ill-defined intestinal tract infections
Intestinal Infectious Disease	CTV3	XE0Qo	Colitis, enteritis and gastroenteritis presumed infectious
Intestinal Infectious Disease	CTV3	XE0Qp	Epidemic diarrhoea
Intestinal Infectious Disease	CTV3	XE0Qq	Diarrhoea of presumed infectious origin
Intestinal Infectious Disease	CTV3	XE0Sm	(Intestinal infectious diseases) or (unspecified enteritis)
Intestinal Infectious Disease	CTV3	XE0Ss	(Bacillary dysentery) or (shigellosis)
Intestinal Infectious Disease	CTV3	XE0Su	Giardiasis (& [colitis])
Intestinal Infectious Disease	CTV3	XE0Sw	(OS GIT infections) or (botulism) or (Campylobacter enteritis)
Intestinal Infectious Disease	CTV3	XE0Sy	Viral: [ill-defined GIT infections (& diarrhoea)] or [gastroenteritis]
Intestinal Infectious Disease	CTV3	XE0T0	(Gastroenteritis (& [viral NOS])) or (vomiting: [viral] or [& diarrhoea infection]) or (gastric flu)
Intestinal Infectious Disease	CTV3	XE0T2	Infectious: [intestinal disease NOS] or [diarrhoea NOS]
Intestinal Infectious Disease	CTV3	XM0pJ	Bacterial gastroenteritis
Intestinal Infectious Disease	CTV3	XM0pK	Bacillary dysentery
Intestinal Infectious Disease	READ2	A0...	Intestinal infectious diseases
Intestinal Infectious Disease	READ2	A00..	Cholera
Intestinal Infectious Disease	READ2	A000.	Cholera - Vibrio cholerae
Intestinal Infectious Disease	READ2	A001.	Cholera - Vibrio cholerae El Tor
Intestinal Infectious Disease	READ2	A00z.	Cholera NOS
Intestinal Infectious Disease	READ2	A01..	Typhoid and paratyphoid fevers
Intestinal Infectious Disease	READ2	A010.	Typhoid fever
Intestinal Infectious Disease	READ2	A011.	Paratyphoid fever A
Intestinal Infectious Disease	READ2	A012.	Paratyphoid fever B
Intestinal Infectious Disease	READ2	A013.	Paratyphoid fever C
Intestinal Infectious Disease	READ2	A01z.	Paratyphoid fever NOS
Intestinal Infectious Disease	READ2	A02..	Other salmonella infections
Intestinal Infectious Disease	READ2	A020.	Salmonella gastroenteritis
Intestinal Infectious Disease	READ2	A021.	Salmonella septicaemia
Intestinal Infectious Disease	READ2	A022.	Localised salmonella infection
Intestinal Infectious Disease	READ2	A0220	Local salmonella infection unspecified
Intestinal Infectious Disease	READ2	A0221	Salmonella meningitis
Intestinal Infectious Disease	READ2	A0222	Salmonella pneumonia
Intestinal Infectious Disease	READ2	A0223	Salmonella arthritis
Intestinal Infectious Disease	READ2	A0224	Salmonella osteomyelitis
Intestinal Infectious Disease	READ2	A022z	Other local salmonella infection
Intestinal Infectious Disease	READ2	A023.	Salmonella sepsis

Intestinal Infectious Disease	READ2	A02y.	Other specified salmonella infection
Intestinal Infectious Disease	READ2	A02z.	Salmonella infection NOS
Intestinal Infectious Disease	READ2	A03..	Shigellosis
Intestinal Infectious Disease	READ2	A030.	Shigella dysenteriae (group A)
Intestinal Infectious Disease	READ2	A031.	Shigella flexneri (group B)
Intestinal Infectious Disease	READ2	A032.	Shigella boydii (group C)
Intestinal Infectious Disease	READ2	A033.	Shigella sonnei (group D)
Intestinal Infectious Disease	READ2	A03y.	Other specified shigella infection
Intestinal Infectious Disease	READ2	A03z.	Shigellosis NOS
Intestinal Infectious Disease	READ2	A04..	Other bacterial food poisoning
Intestinal Infectious Disease	READ2	A040.	Staphylococcal food poisoning
Intestinal Infectious Disease	READ2	A041.	Botulism
Intestinal Infectious Disease	READ2	A042.	Clostridium perfringens food poisoning
Intestinal Infectious Disease	READ2	A043.	Other clostridia causing food poisoning
Intestinal Infectious Disease	READ2	A044.	Vibrio parahaemolyticus food poisoning
Intestinal Infectious Disease	READ2	A04y.	Other specified bacterial food poisoning
Intestinal Infectious Disease	READ2	A04y0	Foodborne Bacillus cereus intoxication
Intestinal Infectious Disease	READ2	A04z.	Food poisoning NOS
Intestinal Infectious Disease	READ2	A05..	Amoebiasis
Intestinal Infectious Disease	READ2	A050.	Acute amoebic dysentery
Intestinal Infectious Disease	READ2	A051.	Chronic intestinal amoebiasis
Intestinal Infectious Disease	READ2	A052.	Amoebic nondysenteric colitis
Intestinal Infectious Disease	READ2	A053.	Amoebic liver abscess
Intestinal Infectious Disease	READ2	A054.	Amoebic lung abscess
Intestinal Infectious Disease	READ2	A055.	Amoebic brain abscess
Intestinal Infectious Disease	READ2	A056.	Amoebic skin ulceration
Intestinal Infectious Disease	READ2	A05y.	Amoebic infection of other sites
Intestinal Infectious Disease	READ2	A05y0	Amoebic appendicitis
Intestinal Infectious Disease	READ2	A05y1	Amoebic balanitis
Intestinal Infectious Disease	READ2	A05y2	Amoeboma
Intestinal Infectious Disease	READ2	A05yz	Amoebic infection of other sites NOS
Intestinal Infectious Disease	READ2	A05z.	Amoebiasis NOS
Intestinal Infectious Disease	READ2	A06..	Other protozoal intestinal diseases
Intestinal Infectious Disease	READ2	A060.	Balantidiasis
Intestinal Infectious Disease	READ2	A061.	Giardiasis - Lambliasis
Intestinal Infectious Disease	READ2	A062.	Coccidiosis
Intestinal Infectious Disease	READ2	A063.	Intestinal trichomoniasis
Intestinal Infectious Disease	READ2	A064.	Cryptosporidiosis
Intestinal Infectious Disease	READ2	A06y.	Other specified protozoal intestinal diseases
Intestinal Infectious Disease	READ2	A06z.	Protozoal intestinal diseases NOS
Intestinal Infectious Disease	READ2	A07..	Intestinal infection due to other organisms
Intestinal Infectious Disease	READ2	A070.	Escherichia coli gastrointestinal tract infection
Intestinal Infectious Disease	READ2	A0700	Enteropathogenic Escherichia coli infection
Intestinal Infectious Disease	READ2	A0701	Enterotoxigenic Escherichia coli infection
Intestinal Infectious Disease	READ2	A0702	Enteroinvasive Escherichia coli infection
Intestinal Infectious Disease	READ2	A0703	Enterohaemorrhagic Escherichia coli infection

Intestinal Infectious Disease	READ2	A071.	Arizona paracolon gastrointestinal tract infection
Intestinal Infectious Disease	READ2	A072.	Aerobacter aerogenes gastrointestinal tract infection
Intestinal Infectious Disease	READ2	A073.	Proteus gastrointestinal tract infection
Intestinal Infectious Disease	READ2	A0730	Proteus mirabilis gastrointestinal tract infection
Intestinal Infectious Disease	READ2	A0731	Proteus morganii gastrointestinal tract infection
Intestinal Infectious Disease	READ2	A073z	Proteus gastrointestinal tract infection NOS
Intestinal Infectious Disease	READ2	A074.	Other specified gastrointestinal tract bacterial infection
Intestinal Infectious Disease	READ2	A0740	Staphylococcal gastrointestinal tract infection
Intestinal Infectious Disease	READ2	A0741	Pseudomonas gastrointestinal tract infection
Intestinal Infectious Disease	READ2	A0743	Campylobacter gastrointestinal tract infection
Intestinal Infectious Disease	READ2	A0744	Enteritis due to Yersinia enterocolitica
Intestinal Infectious Disease	READ2	A0745	Helicobacter pylori gastrointestinal tract infection
Intestinal Infectious Disease	READ2	A074y	Other specified other gastrointestinal infection
Intestinal Infectious Disease	READ2	A074z	Other specified gastrointestinal tract infections NOS
Intestinal Infectious Disease	READ2	A075.	Unspecified bacterial enteritis
Intestinal Infectious Disease	READ2	A076.	Enteritis due to specified virus
Intestinal Infectious Disease	READ2	A0760	Enteritis due to adenovirus
Intestinal Infectious Disease	READ2	A0761	Enteritis due to enterovirus
Intestinal Infectious Disease	READ2	A0762	Enteritis due to rotavirus
Intestinal Infectious Disease	READ2	A0763	Enteritis due to norovirus
Intestinal Infectious Disease	READ2	A076z	Enteritis due to specified virus NOS
Intestinal Infectious Disease	READ2	A07y.	Gastrointestinal tract infection due to specified organism NEC
Intestinal Infectious Disease	READ2	A07y0	Viral gastroenteritis
Intestinal Infectious Disease	READ2	A07y1	Infantile viral gastroenteritis
Intestinal Infectious Disease	READ2	A07z.	Gastrointestinal tract infection due to specified organism NOS
Intestinal Infectious Disease	READ2	A08..	Ill-defined intestinal tract infections
Intestinal Infectious Disease	READ2	A080.	Infectious colitis, enteritis and gastroenteritis
Intestinal Infectious Disease	READ2	A0801	Infectious colitis
Intestinal Infectious Disease	READ2	A0802	Infectious enteritis
Intestinal Infectious Disease	READ2	A0803	Infectious gastroenteritis
Intestinal Infectious Disease	READ2	A0804	Catarrhal dysentery
Intestinal Infectious Disease	READ2	A0805	Haemorrhagic dysentery
Intestinal Infectious Disease	READ2	A080z	Infectious colitis, enteritis and gastroenteritis NOS
Intestinal Infectious Disease	READ2	A081.	Colitis, enteritis and gastroenteritis presumed infectious
Intestinal Infectious Disease	READ2	A0810	Colitis - presumed infectious origin
Intestinal Infectious Disease	READ2	A0811	Enteritis - presumed infectious origin
Intestinal Infectious Disease	READ2	A0812	Gastroenteritis - presumed infectious origin
Intestinal Infectious Disease	READ2	A081z	Colitis, enteritis and gastroenteritis presumed infectious origin NOS
Intestinal Infectious Disease	READ2	A082.	Infectious diarrhoea
Intestinal Infectious Disease	READ2	A0820	Dysenteric diarrhoea
Intestinal Infectious Disease	READ2	A0821	Epidemic diarrhoea
Intestinal Infectious Disease	READ2	A082z	Infectious diarrhoea NOS
Intestinal Infectious Disease	READ2	A083.	Diarrhoea of presumed infectious origin
Intestinal Infectious Disease	READ2	A08z.	Ill defined gastrointestinal tract infections NOS
Intestinal Infectious Disease	READ2	A0y..	Other specified infectious diseases of intestinal tract
Intestinal Infectious Disease	READ2	A0z..	Intestinal tract infectious disease NOS

Urinary Tract Infection	CTV3	A32y3	Diphtheritic cystitis
Urinary Tract Infection	CTV3	A9811	Acute gonococcal cystitis
Urinary Tract Infection	CTV3	A9831	Chronic gonococcal cystitis
Urinary Tract Infection	CTV3	K15..	Cystitis
Urinary Tract Infection	CTV3	K150.	Acute cystitis
Urinary Tract Infection	CTV3	K151.	Chronic interstitial cystitis
Urinary Tract Infection	CTV3	K1510	Hunner's ulcer
Urinary Tract Infection	CTV3	K1511	Panmural fibrosis of bladder
Urinary Tract Infection	CTV3	K1512	Submucous cystitis
Urinary Tract Infection	CTV3	K151z	Chronic interstitial cystitis NOS
Urinary Tract Infection	CTV3	K152.	Other chronic cystitis
Urinary Tract Infection	CTV3	K1520	Subacute cystitis
Urinary Tract Infection	CTV3	K152y	Chronic cystitis unspecified
Urinary Tract Infection	CTV3	K152z	Other chronic cystitis NOS
Urinary Tract Infection	CTV3	K153.	Trigonitis
Urinary Tract Infection	CTV3	K1530	Acute trigonitis
Urinary Tract Infection	CTV3	K1531	Chronic trigonitis
Urinary Tract Infection	CTV3	K1532	Urethrotrigonitis
Urinary Tract Infection	CTV3	K153z	Trigonitis NOS
Urinary Tract Infection	CTV3	K154.	Cystitis in diseases EC
Urinary Tract Infection	CTV3	K1540	Cystitis in actinomycosis
Urinary Tract Infection	CTV3	K1541	Cystitis in amoebiasis
Urinary Tract Infection	CTV3	K1542	Cystitis in bilharziasis
Urinary Tract Infection	CTV3	K1543	Cystitis in echinococcus infestation
Urinary Tract Infection	CTV3	K1544	Cystitis in diphtheria
Urinary Tract Infection	CTV3	K1545	Cystitis in gonorrhoea
Urinary Tract Infection	CTV3	K1546	Cystitis in moniliasis
Urinary Tract Infection	CTV3	K1547	Cystitis in trichomoniasis
Urinary Tract Infection	CTV3	K1548	Cystitis in tuberculosis
Urinary Tract Infection	CTV3	K154z	Cystitis in diseases EC NOS
Urinary Tract Infection	CTV3	K155.	Recurrent cystitis
Urinary Tract Infection	CTV3	K15y.	Other specified cystitis
Urinary Tract Infection	CTV3	K15y0	Cystitis cystica
Urinary Tract Infection	CTV3	K15y1	Irradiation cystitis
Urinary Tract Infection	CTV3	K15y2	Abscess of bladder
Urinary Tract Infection	CTV3	K15y3	Malakoplakia of bladder
Urinary Tract Infection	CTV3	K15yz	Other cystitis NOS
Urinary Tract Infection	CTV3	K15z.	Cystitis NOS
Urinary Tract Infection	CTV3	K1632	Bladder diverticulitis
Urinary Tract Infection	CTV3	K190.	Urinary tract infection, site not specified
Urinary Tract Infection	CTV3	K1900	Bacteriuria, site not specified
Urinary Tract Infection	CTV3	K1901	Pyuria, site not specified
Urinary Tract Infection	CTV3	K1902	Post operative urinary tract infection
Urinary Tract Infection	CTV3	K1903	Recurrent urinary tract infection
Urinary Tract Infection	CTV3	K1904	Chronic urinary tract infection
Urinary Tract Infection	CTV3	K1905	Urinary tract infection

Urinary Tract Infection	CTV3	K1906	Urosepsis
Urinary Tract Infection	CTV3	K190X	Persistent proteinuria, unspecified
Urinary Tract Infection	CTV3	K190z	Urinary tract infection, site not specified NOS
Urinary Tract Infection	CTV3	Kyu50	[X]Other chronic cystitis
Urinary Tract Infection	CTV3	Kyu51	[X]Other cystitis
Urinary Tract Infection	CTV3	X30Na	Recurrent cystitis - culture-negative
Urinary Tract Infection	CTV3	X30Nb	Chronic cystitis - culture negative
Urinary Tract Infection	CTV3	X30Nc	Chronic nonspecific cystitis
Urinary Tract Infection	CTV3	X30Nd	Acute radiation cystitis
Urinary Tract Infection	CTV3	X30Ne	Chronic radiation cystitis
Urinary Tract Infection	CTV3	X30Nf	Chemical cystitis
Urinary Tract Infection	CTV3	X30Ng	Leukoplakia of bladder
Urinary Tract Infection	CTV3	X30Nh	Malakoplakia of bladder
Urinary Tract Infection	CTV3	X30NV	Infective cystitis
Urinary Tract Infection	CTV3	X30NX	Chronic infective cystitis
Urinary Tract Infection	CTV3	X30NY	Non-infective cystitis
Urinary Tract Infection	CTV3	X30NZ	Acute cystitis - culture-negative
Urinary Tract Infection	CTV3	X30Pa	Recurrent infective cystitis
Urinary Tract Infection	CTV3	X30PZ	Acute culture positive cystitis
Urinary Tract Infection	CTV3	Xa0t3	Amyloid of bladder
Urinary Tract Infection	CTV3	Xa8EJ	Acute cystitis
Urinary Tract Infection	CTV3	Xa8EK	Chronic cystitis
Urinary Tract Infection	CTV3	XaA05	Bullous cystitis
Urinary Tract Infection	CTV3	XaB66	Acute infective cystitis
Urinary Tract Infection	CTV3	XaY6b	Follicular cystitis
Urinary Tract Infection	CTV3	XE0dq	Trigonitis
Urinary Tract Infection	CTV3	XE0fX	Acute cystitis (& [recurrent])
Urinary Tract Infection	CTV3	XE0fZ	(Cystitis NOS) or (trigonitis)
Urinary Tract Infection	CTV3	XM0sZ	Acute recurrent cystitis
Urinary Tract Infection	CTV3	XM1Ov	Infected diverticulum of bladder
Urinary Tract Infection	CTV3	XM1Ox	Cystitis of pregnancy
Urinary Tract Infection	CTV3	XM1Us	Sterile pyuria
Urinary Tract Infection	READ2	K15..	Cystitis
Urinary Tract Infection	READ2	K150.	Acute cystitis
Urinary Tract Infection	READ2	K151.	Chronic interstitial cystitis
Urinary Tract Infection	READ2	K1510	Hunner's ulcer
Urinary Tract Infection	READ2	K1511	Panmural fibrosis of bladder
Urinary Tract Infection	READ2	K1512	Submucous cystitis
Urinary Tract Infection	READ2	K151z	Chronic interstitial cystitis NOS
Urinary Tract Infection	READ2	K152.	Other chronic cystitis
Urinary Tract Infection	READ2	K1520	Subacute cystitis
Urinary Tract Infection	READ2	K152y	Chronic cystitis unspecified
Urinary Tract Infection	READ2	K152z	Other chronic cystitis NOS
Urinary Tract Infection	READ2	K153.	Trigonitis
Urinary Tract Infection	READ2	K1530	Acute trigonitis
Urinary Tract Infection	READ2	K1531	Chronic trigonitis

Urinary Tract Infection	READ2	K1532	Urethrotrigonitis
Urinary Tract Infection	READ2	K153z	Trigonitis NOS
Urinary Tract Infection	READ2	K154.	Cystitis in diseases EC
Urinary Tract Infection	READ2	K1540	Cystitis in actinomycosis
Urinary Tract Infection	READ2	K1541	Cystitis in amoebiasis
Urinary Tract Infection	READ2	K1542	Cystitis in bilharziasis
Urinary Tract Infection	READ2	K1543	Cystitis in echinococcus infestation
Urinary Tract Infection	READ2	K1544	Cystitis in diphtheria
Urinary Tract Infection	READ2	K1545	Cystitis in gonorrhoea
Urinary Tract Infection	READ2	K1546	Cystitis in moniliasis
Urinary Tract Infection	READ2	K1547	Cystitis in trichomoniasis
Urinary Tract Infection	READ2	K1548	Cystitis in tuberculosis
Urinary Tract Infection	READ2	K154z	Cystitis in diseases EC NOS
Urinary Tract Infection	READ2	K155.	Recurrent cystitis
Urinary Tract Infection	READ2	K15y.	Other specified cystitis
Urinary Tract Infection	READ2	K15y0	Cystitis cystica
Urinary Tract Infection	READ2	K15y1	Irradiation cystitis
Urinary Tract Infection	READ2	K15y2	Abscess of bladder
Urinary Tract Infection	READ2	K15y3	Malakoplakia of bladder
Urinary Tract Infection	READ2	K15yz	Other cystitis NOS
Urinary Tract Infection	READ2	K15z.	Cystitis NOS
Urinary Tract Infection	READ2	K190.	Urinary tract infection, site not specified
Urinary Tract Infection	READ2	K1900	Bacteriuria, site not specified
Urinary Tract Infection	READ2	K1901	Pyuria, site not specified
Urinary Tract Infection	READ2	K1902	Post operative urinary tract infection
Urinary Tract Infection	READ2	K1903	Recurrent urinary tract infection
Urinary Tract Infection	READ2	K1904	Chronic urinary tract infection
Urinary Tract Infection	READ2	K1905	Urinary tract infection
Urinary Tract Infection	READ2	K1906	Urosepsis
Urinary Tract Infection	READ2	K190X	Persistent proteinuria, unspecified
Urinary Tract Infection	READ2	K190z	Urinary tract infection, site not specified NOS
