# Supplementary material for: Incidence of Lower Respiratory Tract Infections and Atopic Conditions in Boys and Young Male Adults: Royal College of General Practitioners Research and Surveillance Centre Annual Report 2015-2016
Source: JMIR Public Health Surveill. 2018 Apr 30;4(2):e49. doi: 10.2196/publichealth.9307 (PMC5952117; doi:10.2196/publichealth.9307)
Supplement: Multimedia Appendix 3 [file publichealth_v4i2e49_app3.pdf]

| Total Denominator n=474,548 | Age band and gender | | Tonsillitis | Common cold | Allergic Rhinitis | Conjunctivitis | Asthma | Bronchitis | UTI | Otitis | Sinusitis | IID | ILI |
|---|---|---|---|---|---|---|---|---|---|---|---|---|---|
| **14,066 Patients** | **<1year M, ref=F** | Numerator | 98 | 11459 | 4 | 1144 | 1 | 723 | 35 | 251 | 1 | 152 | 9 |
| | | OR | 1.27 | 1.11 | N/A | 0.98 | N/A | 1.62 | N/A | 1.14 | N/A | 0.90 | 0.55 |
| | | 95%CI | 0.84-1.93 | 1.02-1.22 | N/A | 0.86-1.11 | N/A | 1.38-1.91 | N/A | 0.88-1.49 | N/A | 0.65-1.26 | 0.13-2.32 |
| | | p-value | .26 | .02 | N/A | .74 | N/A | <0.001 * | N/A | .32 | N/A | .56 | .42 |
| | | Adj. Probability | 0.14% | 11.11% | N/A | 4.83% | N/A | 4.23% | N/A | 0.99% | N/A | 0.68% | 0.16% |
| **75,011 Patients** | **1-4 years M, ref=F** | Numerator | 4144 | 15391 | 277 | 4592 | 368 | 3794 | 349 | 5093 | 25 | 1088 | 187 |
| | | OR | 1.08 | 0.98 | 1.35 | 1.08 | 1.44 | 1.15 | 0.3 | 1.09 | N/A | 0.96 | 1.06 |
| | | 95%CI | 1.01-1.15 | 0.95-1.02 | 1.05-1.74 | 1.01-1.15 | 1.16-1.79 | 1.08-1.23 | 0.24-0.39 | 1.03-1.16 | N/A | 0.85-1.09 | 0.78-1.44 |
| | | p-value | .03 | .43 | .02 | .02 | <0.001 * | <0.001 * | <0.001 * | .004 | N/A | .57 | .69 |
| | | Adj. Probability | 5.45% | 7.62% | 0.35% | 2.78% | 0.44% | 1.56% | 0.05% | 2.69% | N/A | 0.58% | 0.21% |
| **211,752 Patients** | **5-14 years M, ref=F** | Numerator | 5609 | 4598 | 1361 | 937 | 946 | 1699 | 1495 | 931 | 942 | 511 | 716 |
| | | OR | 0.8 | 0.92 | 1.51 | 1.04 | 1.29 | 1.18 | 0.14 | 0.92 | 0.90 | 1.05 | 1.21 |
| | | 95%CI | 0.76-0.85 | 0.88-0.96 | 1.36-1.66 | 0.94-1.15 | 1.15-1.45 | 1.08-1.30 | 0.11-0.18 | 0.86-0.98 | 0.69-1.19 | 0.90-1.23 | 0.98-1.49 |
| | | p-value | <0.001 * | <0.001 * | <0.001 * | .48 | <0.001 * | <0.001 * | <0.001 * | .01 | .47 | .50 | .07 |
| | | Adj. Probability | 4.92% | 1.82% | 0.52% | 0.52% | 0.47% | 0.47% | 0.04% | 0.94% | 0.11% | 0.14% | 0.28% |
| **173,719 Patients** | **15-24 years M, ref=F** | Numerator | 6031 | 9036 | 1773 | 1616 | 1243 | 1835 | 572 | 3468 | 221 | 700 | 380 |
| | | OR | 0.63 | 0.72 | 0.99 | 0.83 | 0.84 | 0.94 | 0.04 | 0.72 | 0.52 | 0.94 | 1.06 |
| | | 95%CI | 0.59-0.67 | 0.67-0.77 | 0.89-1.11 | 0.73-0.96 | 0.73-0.96 | 0.85-1.04 | 0.03-0.05 | 0.63-0.83 | 0.45-0.60 | 0.78-1.13 | 0.91-1.24 |
| | | p-value | <0.001 * | <0.001 * | .89 | .01 | .01 | .24 | <0.001 * | <0.001 * | <0.001 * | .51 | .47 |
| | | Adj. Probability | 2.76% | 0.78% | 0.52% | 0.15% | 0.28% | 0.40% | 0.03% | 0.26% | 0.18% | 0.10% | 0.41% |

* indicates statistically significant, p<0.001
