# Supplementary figures and images for: Incidence of Lower Respiratory Tract Infections and Atopic Conditions in Boys and Young Male Adults: Royal College of General Practitioners Research and Surveillance Centre Annual Report 2015-2016
Source: JMIR Public Health Surveill. 2018 Apr 30;4(2):e49. doi: 10.2196/publichealth.9307 (PMC5952117; doi:10.2196/publichealth.9307)

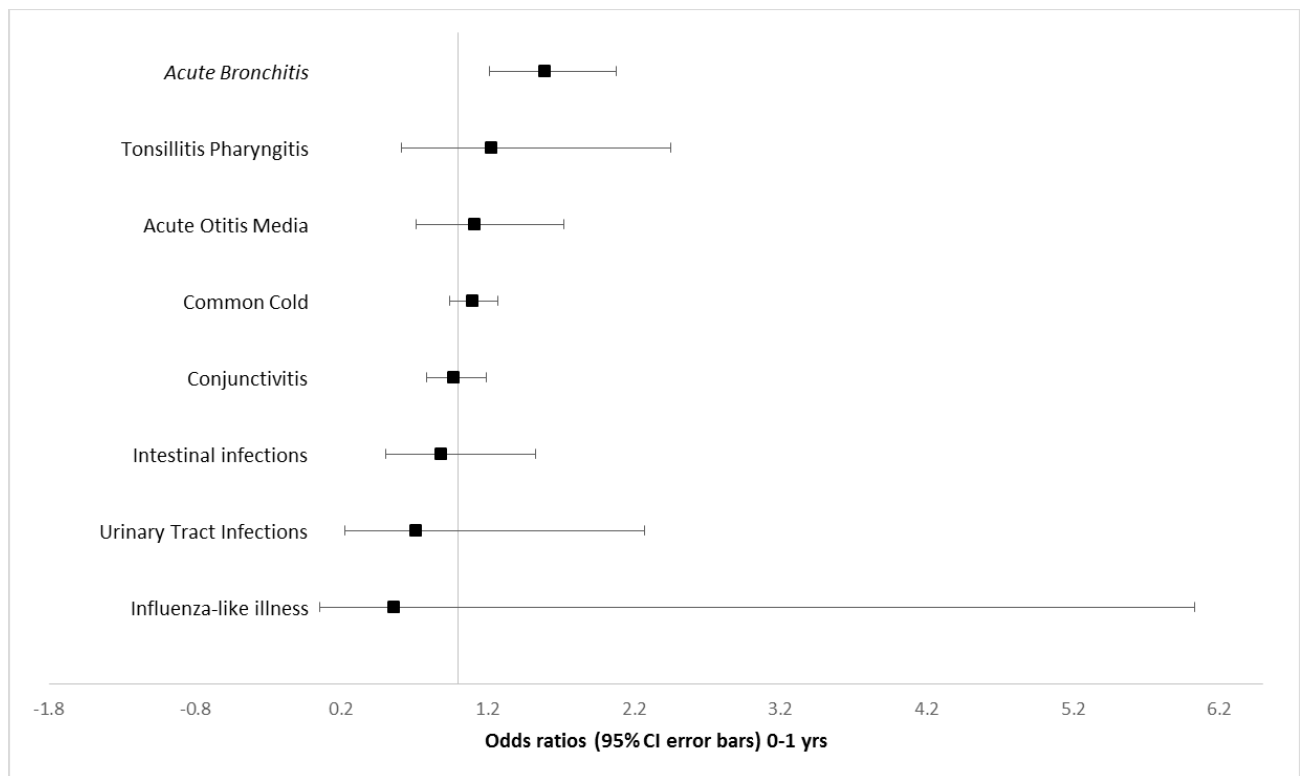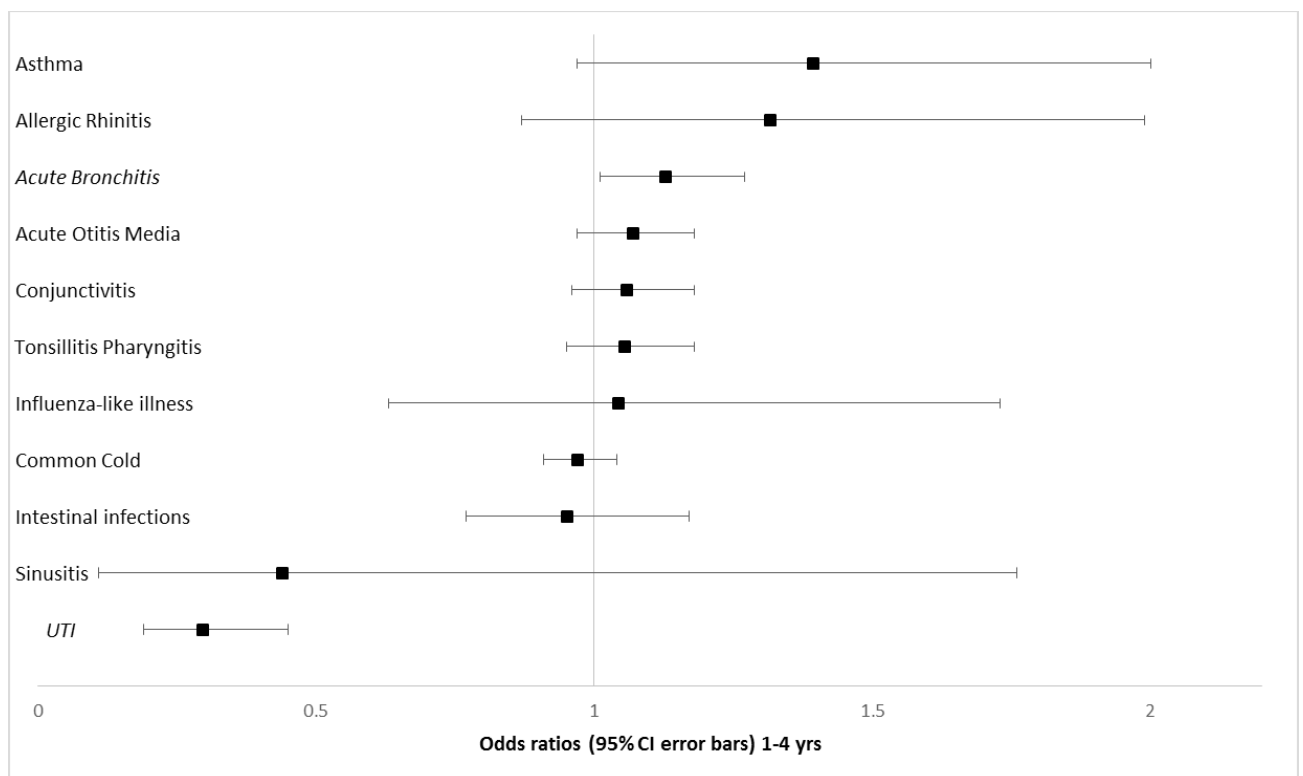

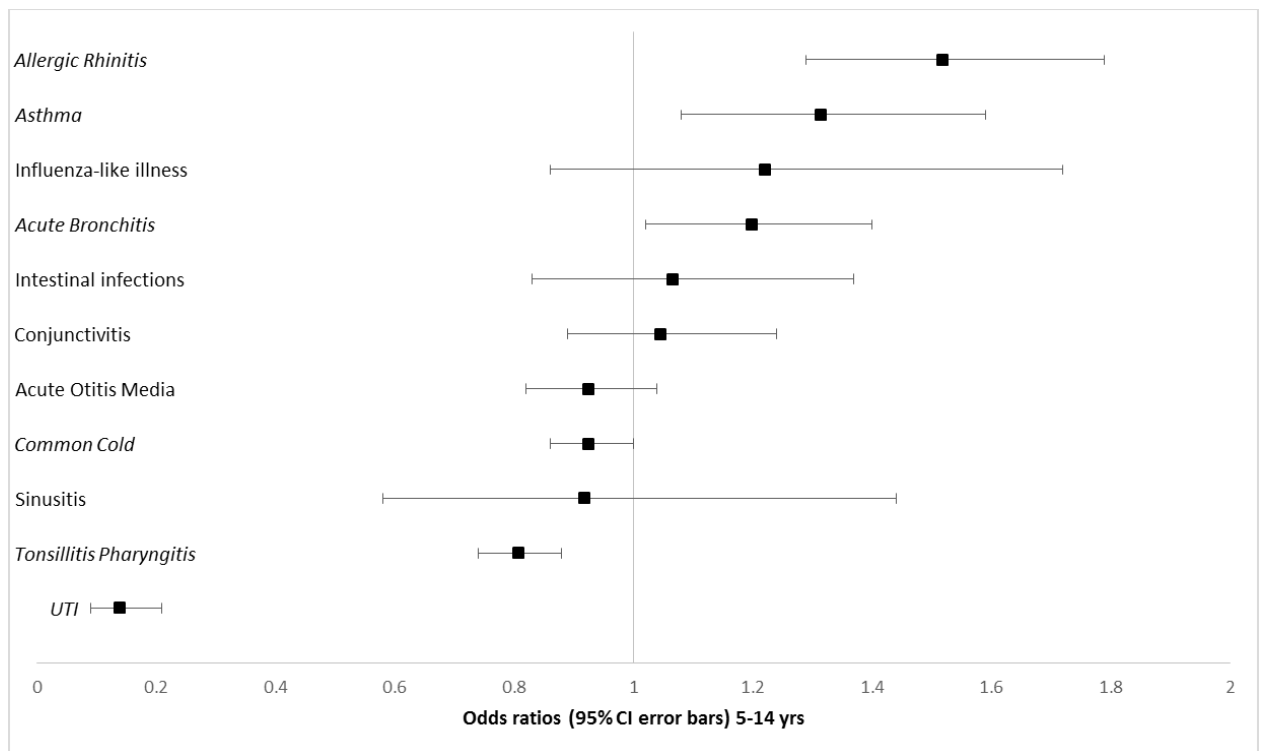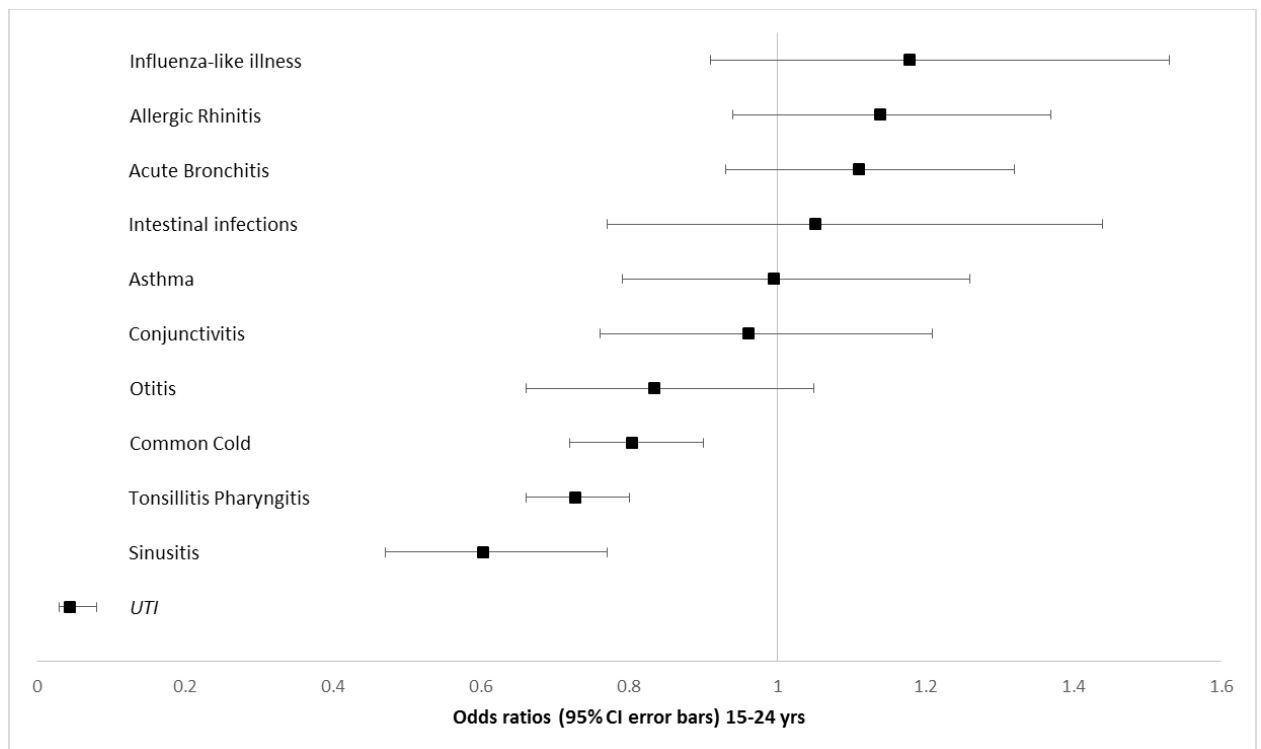

Supplement: Multimedia Appendix 4 [file publichealth_v4i2e49_app4.pdf]
